# Unlocking Catalyst Activation as a Critical Bottleneck in Cross‐Coupling Reactions: Room‐Temperature Couplings of Weak Nucleophiles Enabled by [Pd(1‐MeNAP)TFA]_2_ Precatalysts

**DOI:** 10.1002/anie.3359287

**Published:** 2026-05-13

**Authors:** Sourav Manna, Henric F. Janning, Nikolaos V. Tzouras, Jane Anto Simplica Sagayaraj, Fadil Faizal Mannighayil, Angelino Doppiu, Lukas J. Gooßen

**Affiliations:** ^1^ Chair of Organic Chemistry I, Faculty of Chemistry and Biochemistry Ruhr‐Universität Bochum Bochum Germany; ^2^ Precious Metals Chemistry Umicore AG & Co. KG Hanau‐Wolfgang Germany

**Keywords:** cross‐coupling, homogeneous catalysis, palladium catalysis, precatalysts, weak nucleophile

## Abstract

The trifluoroacetate‐bridged methylnaphthyl complex [Pd(1‐MeNAP)TFA]_2_ is introduced as a bench‐stable, highly soluble palladium source that consistently delivers exceptional catalytic activity. It reacts within minutes with even the most sterically demanding ligands to form monoligated Pd complexes and, upon exposure to nucleophiles, including weak, non‐reducing *N*‐nucleophiles, rapidly generates reactive Pd(0) species with the release of inert naphthalene derivatives. Across 15 representative transformations, catalysts generated in situ from [Pd(1‐MeNAP)TFA]_2_ enable substantial reductions in reaction temperature, often by 80°C, while preserving established ligands and reaction conditions, thereby providing a drop‐in solution to existing reactivity limitations. As a result, arylations of amides, carbamates, sulfonamides, amidines, ureas, cyclopropylamines, and trifluoroethylamines can be performed at room‐temperature with markedly improved functional‐group tolerance and compatibility with sensitive, coordinating heterocycles. Mechanistic studies reveal that catalyst activation, rather than catalytic turnover, has been the principal bottleneck in many cross‐coupling reactions.

## Graphical abstract

In many Pd‐catalyzed cross‐couplings, catalyst performance has been found to be limited by catalyst activation. Methylnaphthyl (MeNAP) palladium trifluoroacetate dimers are presented as easy‐to‐store and easy‐to‐handle precursors, which rapidly activate under reaction conditions, even when only weakly coordinating, non‐reducing nucleophiles are present. They elevate many challenging cross‐couplings to a new level of performance and application width.

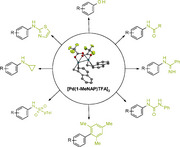

Over the last decades, palladium‐catalyzed cross‐coupling reactions have evolved into indispensable tools across synthetic chemistry [1, 2, 3]. Numerous reaction variants have enabled couplings of organohalides or pseudohalides with various carbon‐ and heteroatom‐based nucleophiles. These are generally understood to proceed via a catalytic cycle consisting of the oxidative addition of the electrophile to a coordinatively unsaturated Pd(0) complex, transmetallation of the nucleophile at the resulting Pd(II) complex, and the release of the product by reductive elimination, thereby regenerating the initial Pd(0) species [3]. Guided by mechanistic insights, steady ligand development has afforded increasingly electron‐rich and sterically demanding phosphine / *N*‐heterocyclic carbene (NHC) architectures. In modern catalyst systems, these ligands enforce monoligation, giving rise to coordinatively unsaturated Pd(0) species, often transiently stabilized through secondary Pd─H/C interactions [4, 5]. Such mono‐ligated complexes are highly electron‐rich, which enables oxidative insertions even into carbon─chlorine bonds, while their pronounced steric bulk facilitates the reductive elimination of the product [6].

The increasing ligand bulk has created a growing mismatch between modern ligand architectures and traditional palladium precursors, such as Pd(II) halides or carboxylates [7, 8, 9, 10, 11], or Pd_2_(dba)_3_ [12, 13]. In recent years, dimeric Pd(II) complexes bearing allyl, indenyl, benzyl, or 2‐aminobiphenyl substituents have been introduced as advantageous Pd(II) sources that allow the in situ generation of defined precatalysts [14, 15, 16, 17, 18, 19, 20, 21, 22, 23, 24, 25, 26, 27, 28]. Alternatively, the catalyst can be generated from Pd(0) complexes bearing N,N‐diaryldiazabutadiene, duroquinone (DQ), 1,5‐cyclooctadiene (COD), or other hemilabile ligands [29, 30, 31]. However, these precursors all have individual limitations with regard to synthetic accessibility, stability, or scope of application. Moreover, state‐of‐the‐art cross‐coupling ligands tend to be extremely bulky, and the formation of active, mono‐ligated species with the above precursors can be slower than the subsequent catalytic reaction [10, 12, 13, 32, 33, 34, 35, 36, 37, 38, 39, 40].

In this respect, pre‐formed monoligated Pd(II)‐precatalysts bearing *η* ^3^‐allyl and aryl substituents have proven to be superior, since they directly liberate catalytically active monoligated Pd(0) species when subjected to the conditions of the catalytic transformation [14, 15, 24, 25, 28, 34, 41, 42, 43, 44, 45, 46]. However, the reliance on pre‐formed complexes becomes impractical when conducting extensive ligand screening.

Catalytically active Pd(0) species are usually generated by liberation of hemilabile ligands from Pd(0) complexes or by reductive couplings of Pd(II) salts with nucleophiles. Palladacyclic precursors undergo reductive extrusion of the chelating ligand (e.g., carbazoles), whereas allyl‐ and aryl‐based precatalysts activate via (pseudo)halide substitution by the nucleophilic reaction partner, followed by reductive elimination of a substituted alkene (Scheme 1) [35].

**SCHEME 1:**
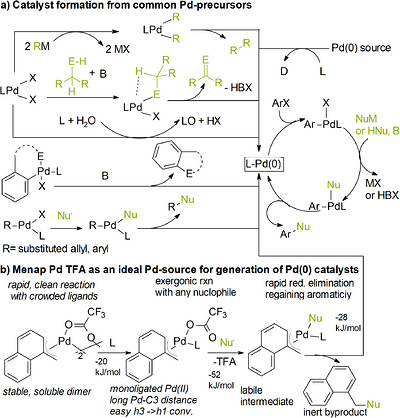
Generation of active catalysts from various Pd‐sources.

While many of the abovementioned systems perform reasonably well in couplings of strongly coordinating, reducing nucleophiles such as organometallic reagents or amines, they are markedly less effective for weak, non‐reducing nucleophiles such as amides. This may be rationalized with the low reactivity of these nucleophiles in the transmetallation step that precedes the reductive activation of Pd(II) precursors and their difficulty to compete for coordination sites at the palladium when strongly coordinating compounds such as dibenzylidene acetone (dba), alkenes, allyl compounds, or carbazoles are present [34, 35, 36, 47].

An optimal palladium precursor for reaction development and preparative chemistry should satisfy five criteria: (a) air‐stability and ease of storage and handling; (b) sufficient solubility to allow dispensing as a concentrated stock solution in an inert solvent; (c) rapid and clean in situ formation of mono‐ligated complexes, irrespective of ligand steric bulk; (d) instant activation upon contact with nucleophiles, including weak ones; and (e) conversion to mono‐ligated Pd(0) species without the formation of coordinating byproducts that could interfere with substrate binding.

To the best of our knowledge, no existing Pd precursor satisfies all these criteria. For instance, Pd(OAc)_2_ and Pd_2_(dba)_3_ react sluggishly with highly hindered ligands such as tBuBrettPhos, and the dba released from the latter is a well‐documented inhibitor in couplings of weak nucleophiles [7, 9, 10, 13, 37, 38, 47]. Buchwald's G3‐precursor reacts somewhat more rapidly but releases coordinating carbazoles upon activation [27, 34, 35, 36]. *η* ^3^‐Allyl and cinnamyl precatalysts also react only slowly with sterically encumbered ligands. Moreover, they tend to form off‐cycle Pd(I) *μ*‐allyl dimers and release coordinating olefins during activation [34, 36, 39, 40]. Oxidative‐addition complexes of aryl (pseudo)halides (e.g., G6 systems) arguably deliver the most efficient catalysts but cannot be generated in situ from one common Pd precursor and various phosphines [46].

We herein disclose a trifluoroacetate‐bridged methylnaphthyl palladium complex, [Pd(1‐MeNAP)TFA]_2_ (**Ia^TFA^,** Scheme 2), that satisfies criteria (a–e) and, as a consequence, consistently improves the efficiency of many cross‐couplings across electrophile and nucleophile classes, providing particularly dramatic improvements for weak, non‐reducing nucleophiles. The key advantage of the TFA dimers is their ability to rapidly generate soluble complexes upon reaction with phosphines, providing species with reactivity comparable to the corresponding triflate complexes. This high solubility is beneficial for all applications, where efficient in situ formation of active complexes from a single, soluble precursor is desired. However, the same property becomes a drawback when isolation of discrete complexes is targeted, as the resulting TFA complexes tend to crystallize poorly.

**SCHEME 2:**
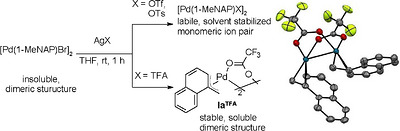
Pd‐source preparation. The X‐ray molecular structure of complex **Ia^TFA^** is presented, showing thermal displacement ellipsoids at the 50% probability level; hydrogen atoms are omitted for clarity (CCDC: 2526679) [49].

In contrast, triflate can be a suitable counterion for isolable complexes, as these species often crystallize more readily. In control experiments, we often found the reactivity of TFA and OTf complexes to be comparable [48]. However, there is no stable, soluble, and reactive dimeric precursor analogous to the TFA dimer. Instead, the OTf system leads to an air‐sensitive, unstable polymeric species, while OTs forms a labile, solvent‐stabilized monomeric ion pair. As a result, the TFA dimer uniquely combines stability, solubility, and rapid ligand substitution, making it particularly well suited as a precursor for the in situ generation of catalytically active complexes (Supporting Information).

Catalytic experiments from our group and that of Nolan had previously revealed that methylnaphthyl (MeNAP) bromide precatalysts perform better than, for example, allyl and cinnamyl complexes in a variety of cross‐coupling reactions [19, 37, 38, 50, 51]. The steric bulk of the MeNAP group promotes selective monoligation, the re‐aromatization of the naphthalene unit provides an additional driving force for ligand dissociation, and the released byproduct is an inert aromatic species rather than a coordinating alkene. However, even [Pd(1‐MeNAP)Br]_2_ reaches its performance limit in reactions that call for extremely bulky ligands. This is illustrated by the comparative study displayed in Scheme 3a. After stirring mixtures of precatalysts with AdBrettPhos for 5 min at room‐temperature, neither the G3‐dimer nor crotyl or cinnamyl complexes showed any conversion, and even [Pd(1‐MeNAP)Br]_2_ (**Ia^Br^)** gave only 30% of the desired monoligated complex. We reasoned that replacing the bromide with a less coordinating counterion would facilitate dissociation of the dimeric structure and thereby accelerate ligand coordination. To test this hypothesis, salt metathesis reactions of **Ia^Br^** with a range of silver salts bearing weakly coordinating anions were investigated (Scheme 2). However, these attempts mostly yielded unstable, poorly soluble, and/or structurally complex species, featuring solvent‐separated or ionic assemblies that are ill‐suited for catalytic applications. This is exemplified by the tosylate and the triflate derivatives (Scheme 2a).

**SCHEME 3:**
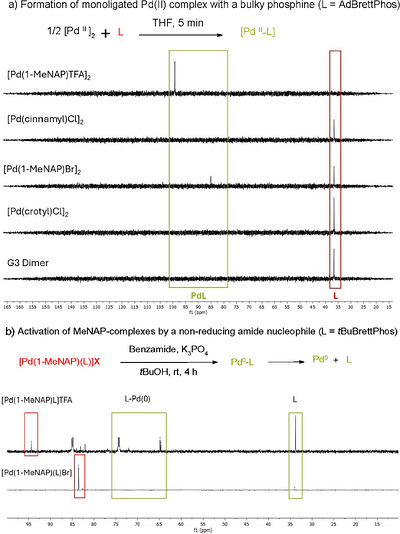
(a) ^31^P NMR of the mixture of AdBrettPhos and different palladium sources in THF after 5 min at rt. (b) Influence of the counter‐ion on the activation of MeNAP complexes.

In contrast, exchange of the bromide for a trifluoroacetate counterion afforded a trifluoroacetate‐bridged bimetallic complex [Pd(1‐MeNAP)TFA]_2_ (**Ia^TFA^**). This covalent, non‐ionic structure induces a remarkably high solubility in organic solvents. Even in inert toluene, **Ia^TFA^** reaches a solubility of 20 mg mL^−^^1^, which is sufficient for high‐throughput screening using liquid‐handling robotics. Moreover, **Ia^TFA^** is not only stable as a solid but also in solution: no visible decomposition was observed after 24 h exposure of solutions in various solvents to air (see Figure S9).

Crucially, however, the trifluoroacetate counterion does not merely improve the solubility and stability of the precatalyst but has a decisive impact on its reactivity. As shown in Scheme 3a, **Ia^TFA^** is quantitatively converted within minutes to monoligated complexes upon reaction with sterically demanding phosphines, including AdBrettPhos (buried volume, BV = 78.36 Å^3^), *t*BuBrettPhos (BV = 78.88 Å^3^), and Me_4_ *t*BuXPhos (BV = 105.57 Å^3^). These observations demonstrate the exceptional coordination efficiency of **Ia^TFA^** and its ability to generate catalytically active monoligated Pd species even with ligands with record‐setting buried volumes (see Figures S2–S8).

Analysis of the X‐ray molecular structure of **Ia^TFA^** revealed that the 1‐methylnaphthyl moiety features an unusually long Pd─C3 bond of 2.6 Å, indicative of a pronounced tendency of the *η* ^3^‐naphthyl unit to shift toward *η* ^1^ coordination. Such bond elongation has been identified by Nolan and coworkers as a predictor of high catalytic activity, as weakened Pd─C interactions correlate with accelerated salt exchange and reductive elimination processes [52].

While transmetallation steps involving nucleophilic amines proceed relatively fast, analogous reactions with weakly nucleophilic amides are typically sluggish and require elevated temperatures. Against this expectation, treatment of **Ia^TFA^** with weakly coordinating benzamide in the presence of the mild base K_3_PO_4_ resulted in near‐complete conversion to a labile, monoligated Pd(0) phosphine complex within four hours at room temperature, as observed by in situ ^3^ ^1^P NMR spectroscopy. GC–MS analysis confirms that this activation step generates the corresponding amide‐substituted methylnaphthalene byproduct, confirming productive amide transmetallation (Supporting Information).

Comparative studies demonstrate that this rapid activation is unique to the [Pd(1‐MeNAP)(*t*BuBrettPhos)]TFA system. Under identical conditions, [Pd(1‐MeNAP)(*t*BuBrettPhos)Br] does not generate monoligated Pd(0) species or corresponding decomposition products when stirred with K_3_PO_4_ and benzamide for 4 h at room‐temperature (Scheme 3b). In contrast, when the [Pd(1‐MeNAP)(*t*BuBrettPhos)]TFA system is subjected to the same conditions, in situ ^3^ ^1^P NMR spectroscopy shows that the signal of the preformed catalyst is almost completely consumed. Concomitantly, signals appear in the 60–75 ppm region that are likely to belong to one of the L─Pd(0) active species [37], along with a free ligand signal at 34 ppm arising from partial decomposition of the Pd(0)–ligand complex [53]. Since the lability of the putative Pd(0) complex prevents complete conversion of the precatalyst to this species, we sought to capture it through a rapid in situ trapping experiment. Consistent with the formation of a well‐defined L–Pd(0) species under these conditions, treatment of [Pd(1‐MeNAP)(tBuBrettPhos)]TFA with 4‐cyanobromobenzene in the presence of NaOt‐Am rapidly generated the corresponding oxidative addition complex in >90% yield within 10 min (Supporting Information). These observations underscore the crucial cooperative roles of both the 1‐methylnaphthyl scaffold and the trifluoroacetate counterion in enabling rapid catalyst activation without the release of strongly coordinating byproducts (Scheme 3b).

To gain further insight into the reactivity differences between [Pd(1‐MeNAP)TFA]_2_ (**Ia^TFA^**) and other precatalysts, we performed DFT calculations at the B3LYP/def2‐SVP/TZVP level with D3BJ dispersion correction, and CPCM solvent field (THF) using ORCA [54]. These revealed that the coordination of bulky *t*BuBrettPhos to the precatalyst **Ib^TFA^** is more exergonic for **Ia^TFA^** (−20 kJ mol^−^^1^), than for [Pd(allyl)TFA]_2_ (**IIa^TFA^**, −13 kJ mol^−^^1^), [Pd(cinnamyl)TFA]_2_ (**IIIa^TFA^**, −12 kJ mol^−^^1^), and [Pd(1‐MeNAP)Br]_2_ (**Ia^Br^**, −15 kJ mol^−^^1^). The subsequent nucleophilic attack of potassium amide on the monoligated Pd(II) complex is also significantly more favorable for **Ib^TFA^** (−52 kJ mol^−^^1^) than for **Ib^Br^** (−19 kJ mol^−^^1^), **IIb^TFA^** (−47 kJ mol^−^^1^), and **IIIb^TFA^** (−47 kJ mol^−^^1^). The reductive elimination of the methylnaphtylamide from **Ic^TFA^** is also more favorable (−28 kJ mol^−^^1^) than the corresponding reaction of **IIc^TFA^** (−17 kJ mol^−^^1^) and **IIIc^TFA^** (−10 kJ mol^−^^1^). Finally, we evaluated the binding strength of the reductive elimination products to the resulting Pd(0)–ligand complex because a strong coordination may block the oxidative addition or other steps of the catalytic cycle. Notably, the methylnaphthyl amide binds less strongly to Pd(0)–L than the corresponding allyl amide and cinnamyl amide (Δ*G* = −49 vs. −61 vs−70 kJ mol^−^^1^). This is a decisive factor, considering that the coordination of the aryl chloride to the Pd, the initiating step in the oxidative addition (**Ie** to **If**), is exergonic only by −25 kJ mol^−^^1^. Overall, the calculations confirm that all catalyst activation steps are facilitated for [Pd(1‐MeNAP)TFA]_2_, and that the naphthalene byproduct is less coordinating than allyl or cinnamyl species (Scheme 4).

**SCHEME 4:**
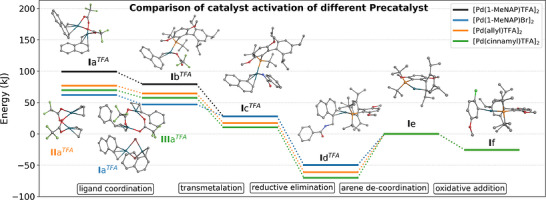
Energy diagram of [Pd(1‐MeNAP)TFA]_2_ (black) compared with related palladium catalysts: [Pd(1‐MeNAP)Br]_2_ (Blue), [Pd(allyl)TFA]_2_ (orange), and [Pd(cinnamyl)TFA]_2_ (green). The Pd(0) species was set to 0 kcal·mol^−1^ for comparative purposes.

Having thus established that the **Ia^TFA^** complex has all prerequisites to act as an effective precatalyst in cross‐couplings of weakly nucleophilic, non‐reducing nucleophiles, we investigated its activity in the challenging coupling of *p*‐chloroanisol with benzamide (Table 1) [55, 56, 57, 58, 59]. Even with state‐of‐the‐art *t*BuBrettPhos ligands, this widely used reaction requires a high temperature of 110°C when using Pd(OAc)_2_ as the precatalyst.

**TABLE 1:** Effects of Pd‐precursors on the catalytic arylation of amides.^a^

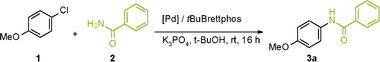			
#	[Pd]	Additive	3a(%)
1	Pd_2_(dba)_3_	−	<1
2	Pd(OAc)_2_	−	<1
3	[Pd(1‐Menap)TFA]_2_	−	>99
4	G3 Dimer	−	<1
5	[Pd(allyl)Cl]_2_	−	31
6	[Pd(cinnamyl)Cl]_2_	−	32
7	[Pd(1‐menap)Br]_2_	−	42
8	*t*BuBrettPhos Pd G6 bromide	−	60
9	[Pd(1‐Menap)TFA]_2_	dba	0
10	“	Styrenebenzamide	52
11	“	Allylbenzamide	0
12	“	Carbazole	45

^a^ Conditions: 0.25 mmol **1**, 1.20 equiv. **2**, 2.0 mol% [Pd], 2.0 mol % ligand, 1.40 equiv. K_3_PO_4_, 2.0 mL t‐BuOH, 25°C, 16 h, yields determined by GC analysis using *n*‐hexadecane as internal standard.

In accordance with literature reports, known catalyst systems generated from Pd_2_(dba)_3_ or Pd(OAc)_2_ and *t*BuBrettPhos were found to give no conversion within 16 h at room‐temperature (entries 1 and 2). In sharp contrast, a near‐quantitative yield of **3** was obtained when switching to **Ia^TFA^** as the precatalyst (entry 3). Under identical conditions, allyl‐, cinnamyl‐, and MeNAP‐halide precursors afforded only low yields, and the G3 precursor was ineffective (entries 4–7). Even a pre‐formed monoligated *t*BuBrettPhos–Pd(II) complex (G6 type) gave lower yields than the catalysts generated in situ from **Ia^TFA,^** which is remarkable, considering that the difficult catalyst activation steps are bypassed when using a one‐component catalyst (entry 8).

Further experiments probed the effect of compounds that are inevitably released during the activation steps from various catalyst precursors. The catalytic activity of the system generated in situ from **Ia^TFA^** was completely inhibited by the addition of dba or allyl benzamide and was markedly reduced by the addition of styrene benzamide or carbazoles—species that would be released during activation of allyl‐ or G3‐type palladium precursors (entries 9–12). In contrast, amide‐substituted methylnaphthalene, the activation byproduct of **Ia^TFA^**, had a much smaller effect on the reaction. To exclude contamination effects, reactions were conducted using new vials, stir bars, and spatulas. No decrease in yield was observed. In addition, control experiments performed in the absence of both Pd and ligand resulted in no detectable product formation. These observations indicate that the observed reactivity does not arise from trace Pd contamination (see Table S1).

A focused set of challenging cross‐couplings was used to evaluate the performance of the **Ia^TFA^**/*t*BuBrettPhos system in catalytic amidations of aryl chlorides (Table 2). In all cases, the reaction proceeded swiftly at room‐temperature rather than the 110°C required by the literature systems. Importantly, the mild conditions allow the conversion of substrates containing sensitive heterocycles and functional groups that were previously outside the scope of known protocols, for example, **3b**–**3s**.

**TABLE 2:** Selected couplings of aryl chlorides with amides.^a^

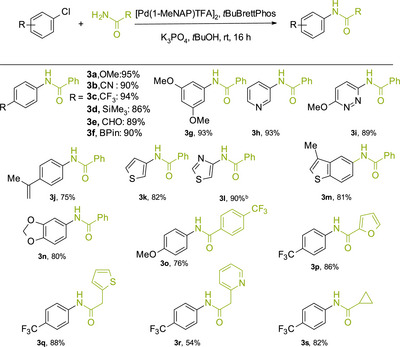

^a^Conditions: 0.50 mmol **1a**, 1.20 equiv. **2a**, 2.0 mol% [Pd], 2.0 mol % ligand, 1.40 equiv. K_3_PO_4_, 3.0 mL *t*‐BuOH, 25°C, 16 h, isolated yields.

^b^ Using the aryl bromide.

Building on the observations made in the development of this amide coupling, we reasoned that couplings of other weak, non‐reducing *N*‐nucleophiles might also be limited by catalyst formation and activation steps. And indeed, when replacing the Pd‐sources of the literature protocols with [Pd(1‐MeNAP)TFA]_2_, couplings of aryl chlorides with amidines [60, 61], ureas [58, 62, 63, 64, 65], carbamates [59, 66, 67], and even sulfonamides [59, 68, 69, 70] proceeded swiftly at room‐temperature. The direct comparison of **Ia^TFA^** with state‐of‐the‐art systems in Table 3a–d shows the magnitude of the precursor effect, enabling temperature reductions of up to 80°C.

**TABLE 3:** Room temperature arylations of non‐reducing *N*‐nucleophiles.^a^

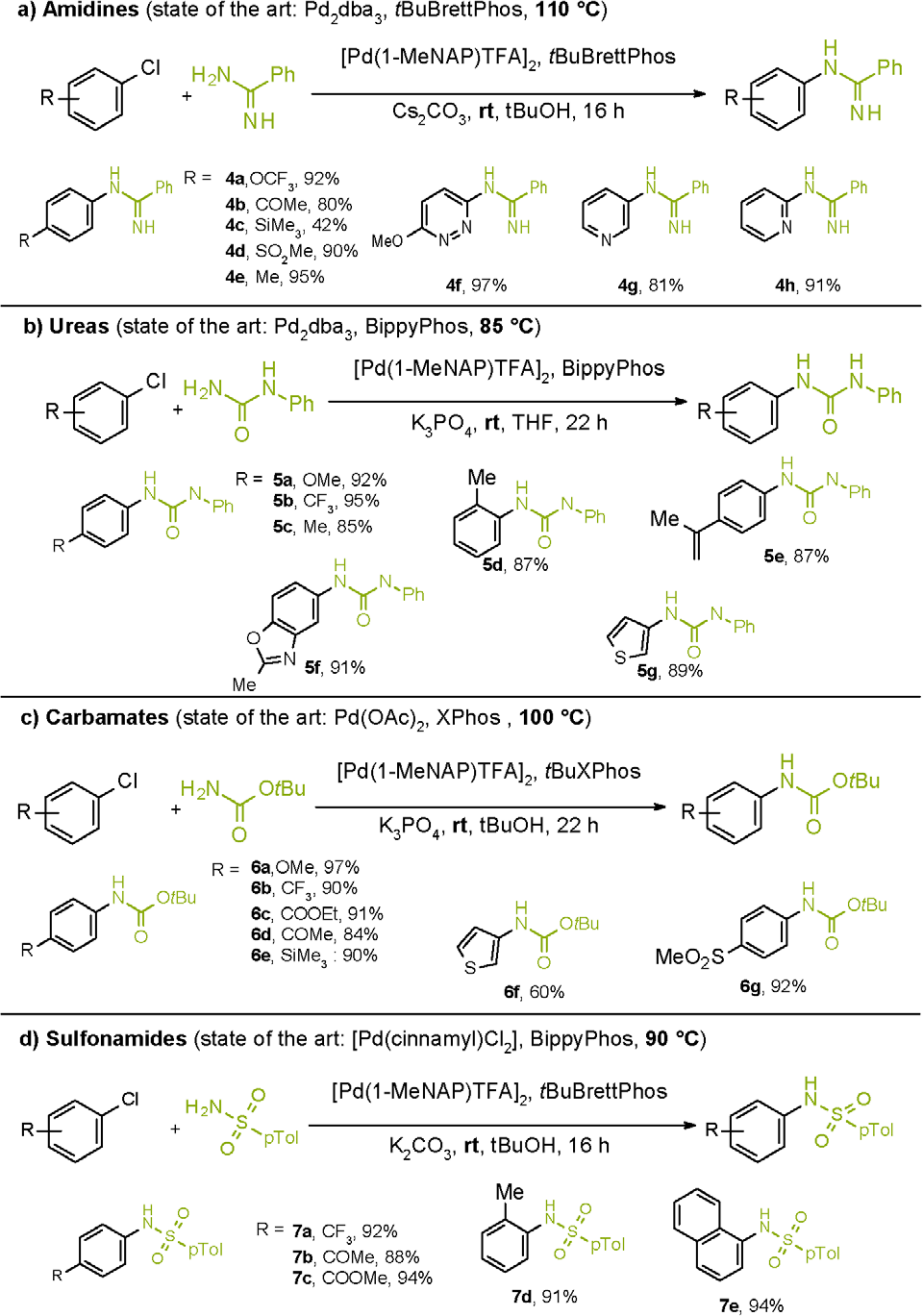

^a^ Conditions: 0.50 mmol ArCl, 1.1–1.2 equiv. *N*‐nucleophile, 0.5 mol% [Pd], 1.0 mol % ligand, 1.4–2.2 equiv base, 2.0 mL solvent, 25°C, 16–22 h, isolated yields. See the Supporting Information for details.

For all couplings, we used selected substrates from the literature tables to benchmark the performance of our catalyst system. Additionally, we applied it to compounds bearing temperature‐sensitive heterocycles or functional groups, for example, pyridazines, pyrimidines, thiophenes, or compounds bearing alkene, or methanesulfonyl groups, which have not previously been accessed via such couplings. These examples provide a glimpse of the opportunities this precatalyst opens for drug discovery and late‐stage functionalization.

In search for other reactions that may benefit from the use of **Ia^TFA^**, we investigated challenging amine arylations. And indeed, couplings of aryl chlorides with ammonia [37], cyclopropylamine [71], and trifluoroethylamine [72], which usually call for rather elevated temperatures, proceeded efficiently at room‐temperature using **Ia^TFA^**. The effect is again remarkable: temperature reductions of 60–80°C relative to standard protocols could be achieved. The reaction of aryl chlorides with ammonia equivalents had been carefully optimized in our group, and a pre‐formed [Pd(1‐MeNAP)tBuBrettPhos]Br complex was found to be most effective. However, still, it required heating to 80°C to achieve high yields. When using a catalyst conveniently generated in situ from **Ia^TFA^** and *t*BuBrettPhos (Table 4a), the transformation went to completion within only 6 h at room‐temperature. The superior performance of the **Ia^TFA^**‐derived catalyst highlights the dual role of the trifluoroacetate counterion in facilitating both the ligand coordination and the catalyst activation.

**TABLE 4:** Challenging aminations at room‐temperature.^a^

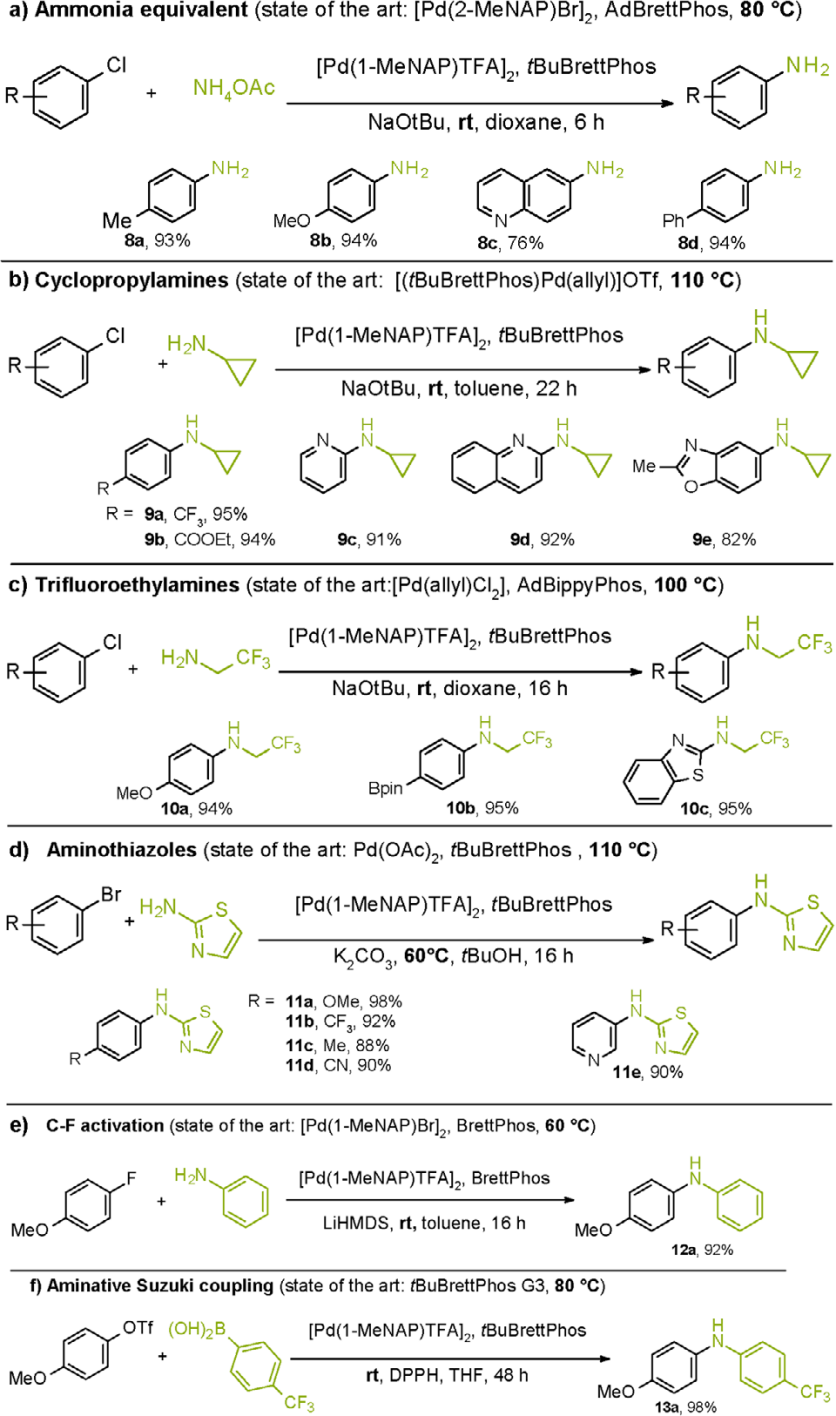

^a^ Conditions: 0.50 mmol aryl source, 1.2–3.0 equiv. nucleophile, 0.125–1.5 mol% [Pd], 0.25‐3 mol % ligand, 1.2–4 equiv. base, 2.0–5.0 mL solvent, 25°C, 6–48 h, isolated yields, see Supporting Information for details.

The arylations of cyclopropylamine and tri‐fluoroethylamine proceeded similarly well. The temperature of the 2‐aminothiazole coupling could be reduced by 50°C—from 110°C to 60°C (Table 4, b–d) [73].

When we recently demonstrated that the notoriously stable C─F bond of non‐activated aryl fluorides can be cleaved in Pd‐catalyzed aminations at only 60°C, this record seemed to be hard to beat [74]. However, with Pd(1‐MeNAP)TFA]_2_ as precatalyst and BrettPhos as the ligand, C─F aminations can now be achieved at room‐temperature (Table 4, e).

We also investigated the aminative Suzuki–Miyaura coupling of 4‐methoxy triflate with boronic acid [75]. Whereas the protocol by Liu, which is based on a pre‐formed *t*BuBrettPhosG3 catalyst calls for 80°C, a catalyst generated in situ from *t*BuBrettPhos and [Pd(1‐MeNAP)TFA]_2_ promotes the same reaction already at room temperature (Table 4, f).

In literature reports of several cross‐coupling reactions, problems with catalyst formation and activation become apparent from the rather cumbersome experimental procedures. For example, Buchwald explicitly stated in his experimental procedure for the coupling of aryl halides with imidazole [33] or triazole [76] with Pd_2_(dba)_3_ catalyst that the precatalyst must first be activated by heating (above 100°C) it with the ligand for 3–5 min. After cooling down the catalyst solution, the actual catalytic coupling is started, which proceeds well at 120°C. The use of **Ia^TFA^** as a catalyst precursor obviates the need for a cumbersome activation process. The reaction can be started simply by mixing **Ia^TFA^** with the ligand and the substrates and then heating up the reaction. The yields of this simplified procedure compare favorably with those achievable with the original protocol (Table 5a, b). In the cyanation of aryl chlorides with the cyanide source K_4_[Fe(CN)_6_]·3H_2_O, allyl‐type Pd‐precursors have been reported to be ineffective, while XPhos‐G3 performs well [77]. This has been rationalized with the latter being resistant to catalyst poisoning by excess cyanide. We were curious to find out whether the Menap catalyst would be similarly stable against catalyst poisoning and were pleased to find out that the catalyst generated in situ from **Ia^TFA^** is as efficient as the elaborate palladacycles of the original protocol (Table 5c).

**TABLE 5:** Bypassing complex catalyst preparation steps.

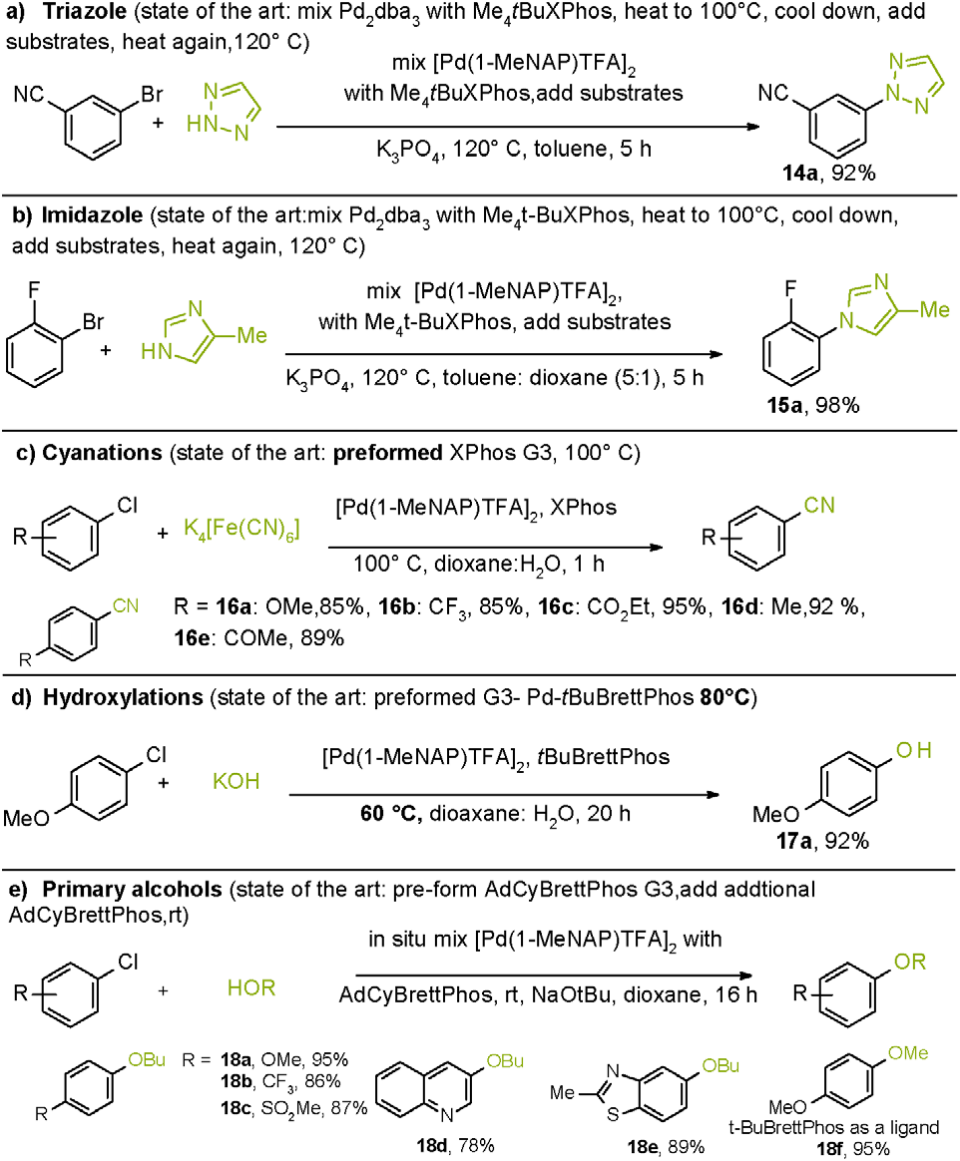

^a^ Conditions: 0.50 mmol aryl source, 0.5–2.0 equiv. Nucleophile, 0.2–2 mol% [Pd], 0.4–6 mol % ligand, 0.13–2 equiv. base, 1.5–2.0 mL solvent, 60 C–120°C, 6–48 h, isolated yields, see Supporting Information for details.

In various C─O coupling reactions, pre‐formed Pd‐phosphine complexes have been reported to perform better than in situ generated complexes. However, catalysts performed in situ from [Pd(1‐MeNAP)TFA]_2_ exhibited excellent activity under the previously reported conditions for the coupling of *n*‐butanol [78], methanol [79], and KOH [80] with aryl chlorides. In the case of KOH coupling, the reaction temperature could be lowered from 80 to 60°C. (Table 5d,e).

To ensure that [Pd(1‐MeNAP)TFA]_2_ is generally applicable to standard cross‐couplings, we applied it to numerous transformations. In all these tests, we did not encounter a single application case in which catalysts generated from **Ia^TFA^** fell behind that of an established system. Its consistent high reactivity is further illustrated by the example reactions in Table 6a–d, which are all challenging and represent the borderline of the possible in cross‐coupling chemistry. C─F bond‐forming reactions belong to the most challenging reactions in Pd‐chemistry [81]. With [Pd(1‐MeNAP)TFA]_2_, they proceed smoothly in reproducibly high yields. The coupling of bulky tert‐butyl amine with a sterically crowded aryl chloride gave a near quantitative yield at room‐temperature with commercially available Ruphos, which compares favorably to the literature report, in which a customized Pd(OAc)_2_ / 9‐[2‐(dicyclohexylphosphino)phenyl]‐2‐ethoxy‐9H‐carbazole/ system was used at 110°C [82]. Arylations of ketones were also found to proceed at lower temperatures than the best known systems [83]. In the Suzuki coupling of sterically crowded substrates, a catalyst generated from **Ia^TFA^** matches the activity of the best‐known systems [84].

**TABLE 6:** Assorted challenging cross‐couplings.

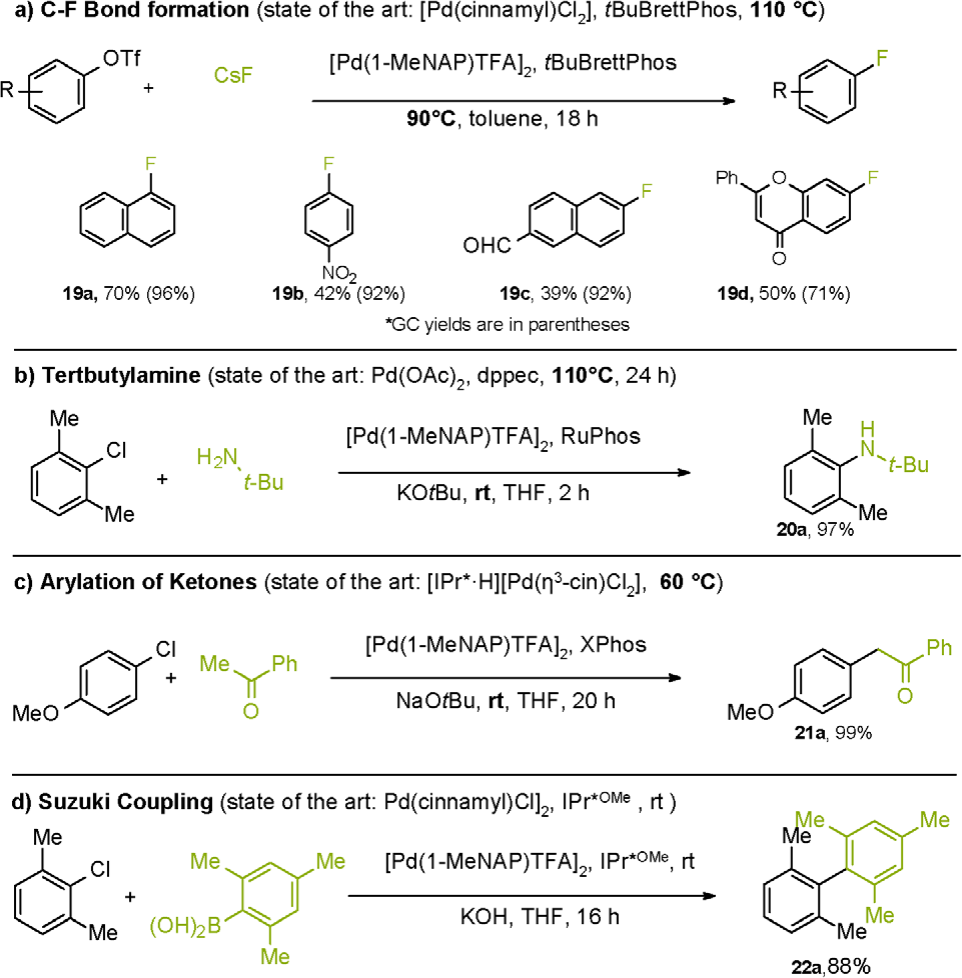

^a^ Conditions: 0.50 mmol aryl source, 1.05–2.0 equiv. Nucleophile, 0.5–2 mol% [Pd], 1–2 mol % ligand, 1.2–2 equiv. base, 1.0–2.0 mL solvent, 25 C–110°C, 2–16 h, isolated yields, see Supporting Information for details.

To probe the nature of the catalytically active species, several mechanistic experiments were performed (Supporting Information). The reaction proceeded unaffected in the presence of a mercury droplet, and filtration of the reaction mixture at partial conversion did not alter the reaction progress. Collectively, these observations argue against a colloidal or heterogeneous catalytic species, although such possibilities cannot be completely excluded.

Its easy handling, its high solubility in inert solvents, and its high catalytic efficiency make [Pd(1‐MeNAP)TFA]_2_ highly advantageous for catalyst screening and preparative applications. It is also ideal for automated systems, since it can be administered in an inert solvent by a liquid handler. The above results show that this can be done without concerns that the activity of the resulting in situ generated systems is inferior to established catalysts.

In conclusion, its solubility and stability in combination with unparalleled reactivity make **Ia^TFA^** an ideal precursor for manual or automated reaction discovery. It is swiftly converted into a catalytically active complex even when only weak, non‐coordinating nucleophiles are present. This elevates many cross‐couplings, especially those that have been limited by catalyst activation steps, to a new level of performance and application width. We are convinced that it will decisively facilitate reaction discovery, since it may give detectable yields already under conditions that are so far away from the optimum that traditional precursors would not have provided traces of product.

## Conflicts of Interest

UMICORE AG & Co. KG (A.D.) and S.M., H.F.J., N.V.T. and L.J.G. have filed a patent on the Pd‐methylnaphthyl complexes described in this manuscript.

## Supporting information




**Supporting File 1**: anie72355‐sup‐0001‐SuppMat.pdf.

## Data Availability

The data that support the findings of this study are openly available in the sciflection repository (https://identifiers.org/sciflection:b99b22cb-8ffd-4dd9-8e9d-1759968b0d52).
