# Supplementary material for: Unlocking Catalyst Activation as a Critical Bottleneck in Cross‐Coupling Reactions: Room‐Temperature Couplings of Weak Nucleophiles Enabled by [Pd(1‐MeNAP)TFA]2 Precatalysts
Source: Angew Chem Int Ed Engl. 2026 May 13;65(27):e3359287. doi: 10.1002/anie.3359287 (PMC13327566; doi:10.1002/anie.3359287)

Supporting Information

Unlocking Catalyst Activation as a Critical Bottleneck in Cross-Coupling Reactions: Room-Temperature Couplings of Weak Nucleophiles Enabled by [Pd(1-MeNAP)TFA]$_2$ Precatalysts

Sourav Manna,[a] Henric F. Janning,[a] Nikolaos V. Tzouras,[a] Jane Anto Simplica Sagayaraj,[a] Fadil Faizal Mannighayil,[a] Angelino Doppiu,[b] and Lukas J. Gooßen*[a]

[a]*Faculty for Chemistry and Biochemistry, Ruhr Universität Bochum, Universitätsstr. 150, 44801 Bochum, Germany*

[b]*Precious Metals Chemistry, Umicore AG & Co. KG, Rodenbacher Chaussee 4, 63457 Hanau-Wolfgang, Germany.*

*E-mail: lukas.goossen@rub.de

Supporting data are openly available in Sciflection.

Table of contents

1. General information **3**

2. General procedures **4**

2.1. General procedure of the C–N cross-coupling of Amides (GP A) *4*

2.2. General procedure of the C–N cross-coupling of Amidines (GP B) *4*

2.3. General procedure of the C–N cross-coupling of Urea (GP C) *5*

2.4. General procedure of the C–N cross-coupling of Carbamates (GP D) *5*

2.5. General procedure of the C–N cross-coupling of Sulfonamide (GP E) *5*

2.6. General procedure of the C–N cross-coupling of Ammonium acetate (GP F) *6*

2.7. General procedure of the C–N cross-coupling of Cyclopropylamine (GP G) *6*

2.8. General procedure of the C–N cross-coupling of Trifluoroethylamine (GP H) *6*

2.9. General procedure of the C–N cross-coupling of Aminothiazole (GP I) *7*

2.10. General procedure of the Aminative C-F activation coupling (GP J) *7*

2.11. General procedure of the Aminative Suzuki–Miyaura coupling (GP K) *7*

2.12. General procedure of the C–N cross-coupling of Triazole (GP L) *8*

2.13. General procedure of the C–N cross-coupling of Imidazole (GP M) *8*

2.14. General procedure of the C–CN cross-coupling of $K_3[Fe(CN)_6]$. (GP N) *8*

2.15. General procedure of the Coupling of KOH (GP O) *9*

2.16. General procedure of the C–O cross-coupling of n-Butanol (GP P) *9*

2.17. General procedure of the Coupling of Methanol (GP Q) *9*

2.18. General procedure of the C–F cross-coupling of CsF (GP R) 10
2.19. General procedure of the Coupling of Tertbutylamine (GP S) 10
2.20. General procedure of the α-Arylation of Ketones (GP T) 10
2.21. General procedure of the Coupling of Boronic acids (GP U) 11

3. Screening tables of the reaction conditions and additional experiments 12
3.1. Screening of the palladium source for the C–N cross-coupling of Amide 12
3.2. Screening of the palladium source for the C–N cross-coupling of Ammonium acetate 13

4. NMR Experiments 14
4.1. Preformation of Palladium(II) complexes incorporating sterically bulky ligands 14
4.2. Preformation of $[Pd(MeNAP)TFA]_2$ incorporating different ligands 15
4.3. Stability of $[Pd(MeNAP)TFA]_2$ in different solvents 22

5. Mechanistic studies 23
5.1. Catalyst Activation in the Buchwald-Hartwig amination of Amides 23
5.2. Catalyst Activation in the Buchwald-Hartwig amination of Ammonium acetate 23
5.3. ^{31}P NMR catalyst activation in the Buchwald-Hartwig amination of Amides 24
5.4 Pd(0) trap experiment 25
5.5 Mercury droplet Expeiment 26
5.6 Filtration Experiment 27

6. UV-VIS Solubility Experiments 28
6.1. Solubility determination for $[Pd(1\text{-}MeNAP)TFA]_2$ 28

7. Calculation Studies 31

8. Synthesis of Palladium Catalysts 32
8.1. Synthesis of $[Pd(1\text{-}MeNAP)TFA]_2$ 32
8.2. Synthesis of $[Pd(1\text{-}MeNAP)OTf]_2$ 33
8.3. Synthesis of $[Pd(1\text{-}MeNAP)OTs]_2$ 34

9. Synthesis of Palladium Precatalysts 35
9.1. Synthesis of [Pd(1-MeNAP)(tBuBrettPhos)TFA] 35
9.2. Synthesis of [Pd(1-MeNAP)(RuPhos)TFA] 36

10. Synthesis Procedures 37

11. References 80

12. Spectra 83

1.General information

All reactions were performed using standard Schlenk techniques or in a nitrogen-filled glovebox (GS Glovebox Systemtechnik). Heating and stirring of reactions were done using aluminum heating blocks with fitting cavities for 20 mL crimp cap vials stacked on top of an IKA RCT basic magnetic stirrer. Optimization reactions were monitored by GC analysis using *n*-hexadecane as internal standard. NMR spectra were recorded at ambient temperature on a Bruker neo-400 or Advance-III-300 spectrometer using $CDCl_3$, C_6D_6, DMSO-d^6, THF-d^6 or CD_2Cl_2 as solvent, with proton, carbon, and fluorine resonances at 400/300, 101/75 and 377 MHz, respectively. All NMR data are reported in parts per million (ppm,) and coupling constants in Hertz (Hz). The ^{1}H and ^{13}C NMR spectra were referenced to the solvent peak: $CDCl_3$ (7.26 ppm in ^{1}H and 77.0 ppm in ^{13}C), C_6D_6 (δ 7.16 ppm in ^{1}H and 128.4 ppm in ^{13}C), DMSO-d^6 (2.50 ppm in ^{1}H and 39.5 ppm in ^{13}C), THF-d^6 (1.73 ppm in ^{1}H and 25.4 ppm in ^{13}C),CD_2Cl_2 (δ 5.32 ppm in ^{1}H and 53.8 ppm in ^{13}C). Multiplicities are given with the following abbreviations: s = singlet, d = doublet, t=triplet, q = quartet, quin = quintett, m = multiplet, dd = doublet of doublets, ddd = doublet of doublets of doublets, br = broad signal. Elemental analyses were performed on an Elementar vario MICRO-cube elemental analyzer. Mass spectrometric data were acquired on an 8890 GC QTOF. The MS ionization was achieved by EI. HPLC-MS was measured on an HPLC Agilent 1100 combined with a MS Waters Micromass ZQ. The MS ionization was achieved by ESI. Infrared spectra were recorded on Bruker ALPHA FT-IR spectrometer. Bands are given in cm^{-1} with intensities (vs = very strong, s = strong, m = medium, w = weak). GC analyses were carried out using an HP-5 capillary column (Phenyl methyl siloxane, 30 m × 320 × 0.25, 100/2.3-30-300/3, 2 min at 60 °C, heating rate 30 °C/min, 10 min at 300 °C). Column chromatography was performed on a CombiFlash Companion (Isco) or on a Reveleris X2 (BUCHI) Flash Chromatography-System using Reveleris packed columns (12 g). Solvents were dried over molecular sieves or obtained from the solvent-drying system (Braun SPS System) and stored over 3 or 4 Å molecular sieves. Molecular sieves were activated in the microwave prior to use. All solvents and liquid reagents were degassed by bubbling nitrogen through the solvent. For improved reproducibility of the reaction, the hot plate temperature was carefully maintained at 27–29 °C, particularly when *t*-BuOH was used as the solvent. Commercial substrates were used as received unless otherwise stated. Unless otherwise mentioned, phosphine ligands, imidazolium salts, Pd sources, and well-defined Pd pre-catalysts were purchased from commercial sources or were donated by UMICORE.

2. General procedures

2.1. General procedure of the C–N cross-coupling of Amides (GP A)

A crimp cap vial equipped with a Teflon coated magnetic stirring bar was charged with [Pd(1-MeNAP)TFA]$_2$ (3.6 mg, 0.005 mmol, 1 mol%) and *t*BuBrettPhos (4.9 mg, 0.01 mmol, 2 mol%). Degassed and dry *t*BuOH (1.5 mL) was added and the solution was stirred for 5 minutes. A second oven-dried crimp cap vial equipped with a Teflon coated magnetic stirring bar was charged with the aryl chloride (if solid, 0.5 mmol, 1.0 equiv.) and amide (1.20 equiv.), The vial was entered inside a glovebox, where K_3PO_4 (149 mg, 0.7 mmol, 1.4 equiv.) was added. The vial was sealed, transferred out of the glovebox and aryl chloride (if liquid, 0.5 mmol, 1.0 equiv.) was added *via* syringe under nitrogen unless mentioned otherwise. The catalyst solution was then transferred into the second vial and the first vial was washed with *t*BuOH (1.5 mL). The mixture was allowed to stir at room temperature for 16 hours if not stated otherwise. The mixture was diluted with ethyl acetate (30 mL), and extracted with saturated Na_2CO_3 (30 mL), water (30 mL) and brine (30 mL). The organic phase was dried over Na_2SO_4 and purified by flash column chromatography (SiO_2, cyclohexane / ethyl acetate) to yield the Amide derivatives.

2.2. General procedure of the C–N cross-coupling of Amidines (GP B)

A crimp cap vial equipped with a Teflon coated magnetic stirring bar was charged with [Pd(1-MeNAP)TFA]$_2$ (1.8 mg, 0.0025 mmol, 0.5 mol%) and *t*BuBrettPhos (2.47 mg, 0.005 mmol, 1 mol%). Degassed and dry *t*BuOH (1 mL) was added and the solution was stirred for 5 minutes. A second oven-dried crimp cap vial equipped with a Teflon coated magnetic stirring bar was charged with the aryl chloride (if solid, 0.5 mmol, 1.0 equiv.) and benzamidine hydrochloride (86.8 mg, 0.55 mmol, 1.1 equiv.). The vial was entered inside a glovebox, where Cs_2CO_3(grinded before using) (424 mg, 1.3 mmol, 2.6 equiv.) was added. The vial was sealed, transferred out of the glovebox and aryl chloride (if liquid, 0.5 mmol, 1.0 equiv.) was added *via* syringe under nitrogen unless mentioned otherwise. The catalyst solution was then transferred into the second vial and the first vial was washed with *t*BuOH (1 mL). The mixture was allowed to stir at room temperature for 16 hours if not stated otherwise. The mixture was diluted with ethyl acetate (30 mL), and extracted with saturated Na_2CO_3 (30 mL), water (30 mL) and brine (30 mL). The organic phase was dried over Na2SO4 and purified by flash column chromatography (SiO_2, 1% NEt_3 in cyclohexane /1% NEt_3 and 10% iPrOH in ethyl acetate to yield the Amidine derivatives.

2.3. General procedure of the C–N cross-coupling of Urea (GP C)

A crimp cap vial equipped with a Teflon coated magnetic stirring bar was charged Pd(1-MeNAP)TFA]$_2$ (3.6 mg, 0.005 mmol, 1 mol%) and BippyPhos (5.2 mg, 0.01 mmol, 2 mol%). Degassed and dry THF (1 mL) was added and the solution was stirred for 5 minutes. A second oven-dried crimp cap vial equipped with a Teflon coated magnetic stirring bar was charged with the aryl chloride (if solid, 0.5 mmol, 1.0 equiv.) and Phenylurea (84.2 mg, 0.6 mmol, 1.2 equiv.). The vial was entered inside a glovebox, where K_3PO_4 (149 mg, 0.7 mmol, 1.4 equiv.) was added. The vial was sealed, transferred out of the glovebox and aryl chloride (if liquid, 0.5 mmol, 1.0 equiv.) was added *via* syringe under nitrogen unless mentioned otherwise. The catalyst solution was then transferred into the second vial and the first vial was washed with THF (1 mL). The mixture was allowed to stir at room temperature for 22 hours if not stated otherwise. The mixture was diluted with ethyl acetate (30 mL), and extracted with saturated Na_2CO_3 (30 mL), water (30 mL) and brine (30 mL). The organic phase was dried over Na_2SO_4 and purified by flash column chromatography (SiO_2, 1% NEt_3 in cyclohexane /1% NEt_3 and 10% iPrOH in ethyl acetate to yield the Urea derivatives.

2.4. General procedure of the C–N cross-coupling of Carbamates (GP D)

A crimp cap vial equipped with a Teflon coated magnetic stirring bar was charged Pd(1-MeNAP)TFA]$_2$ (3.6 mg, 0.005 mmol, 1 mol%) and *t*BuXPhos (4.3 mg, 0.01 mmol, 2 mol%). Degassed and dry *t*BuOH (1 mL) was added and the solution was stirred for 5 minutes. A second oven-dried crimp cap vial equipped with a Teflon coated magnetic stirring bar was charged with the aryl chloride (if solid, 0.5 mmol, 1.0 equiv.) and *tert*-Butyl carbamate (71.7 mg, 0.6 mmol, 1.2 equiv.). The vial was entered inside a glovebox, where K_3PO_4 (149 mg, 0.7 mmol, 1.4 equiv.) was added. The vial was sealed, transferred out of the glovebox and aryl chloride (if liquid, 0.5 mmol, 1.0 equiv.) was added *via* syringe under nitrogen unless mentioned otherwise. The catalyst solution was then transferred into the second vial and the first vial was washed with *t*BuOH (1 mL). The mixture was allowed to stir at room temperature for 22 hours if not stated otherwise. The mixture was diluted with ethyl acetate (30 mL), and extracted with saturated Na_2CO_3 (30 mL), water (30 mL) and brine (30 mL). The organic phase was dried over Na_2SO_4 and purified by flash column chromatography (SiO_2, cyclohexane / ethyl acetate to yield the Carbamates derivatives.

2.5. General procedure of the C–N cross-coupling of Sulfonamide (GP E)

A crimp cap vial equipped with a Teflon coated magnetic stirring bar was charged with [Pd(1-MeNAP)TFA]$_2$ (4.5 mg, 0.0063 mmol, 1.25 mol%) and *t*BuBrettPhos (12.1 mg, 0.025 mmol, 5 mol%). Degassed and dry *t*BuOH (1 mL) was added and the solution was stirred for 5 minutes. A second oven-dried crimp cap vial equipped with a Teflon coated magnetic stirring bar was charged with the aryl chloride (if solid, 0.5 mmol, 1.0 equiv.) and *p*-toluenesulfonamide (94.2 mg, 0.55 mmol, 1.1 equiv.). The vial was entered inside a glovebox, where K_2CO_3 (139 mg, 1.0 mmol, 2.0 equiv.) was added. The vial was sealed, transferred out of the glovebox and aryl chloride (if liquid, 0.5 mmol, 1.0 equiv.) was added *via* syringe under nitrogen unless mentioned otherwise. The catalyst solution was then transferred into the second vial and the first vial was washed with *t*BuOH (1.0 mL). The mixture was allowed to stir at room temperature for 16 hours if not stated otherwise. The mixture was diluted with ethyl acetate (30 mL), and extracted with saturated Na_2CO_3 (30 mL), water (30 mL) and brine (30 mL). The organic phase was dried over Na_2SO_4 and purified by flash column chromatography (SiO_2, cyclohexane / ethyl acetate) to yield the sulfonamide derivatives.

2.6. General procedure of the C–N cross-coupling of Ammonium acetate (GP F)

A crimp cap vial equipped with a Teflon coated magnetic stirring bar was charged with [Pd(1-MeNAP)TFA]$_2$ (1.8 mg, 0.0025 mmol, 0.5 mol%) and *t*BuBrettPhos (2.42 mg, 0.005 mmol, 1 mol%). Degassed and dry 1,4-Dioxane (2.5 mL) was added and the solution was stirred for 5 minutes. A second oven-dried crimp cap vial equipped with a Teflon coated magnetic stirring bar was charged with the aryl chloride (if solid, 0.5 mmol, 1.0 equiv.). The vial was entered inside a glovebox, where NaO*t*Bu (192 mg, 2 mmol, 4.0 equiv.), NH_4OAc (116 mg, 1.5 mmol, 3.0 equiv.) was added. The vial was sealed, transferred out of the glovebox and aryl chloride (if liquid, 0.5 mmol, 1.0 equiv.) was added *via* syringe under nitrogen unless mentioned otherwise. The catalyst solution was then transferred into the second vial and the first vial was washed with 1,4-Dioxane (2.5 mL). The mixture was allowed to stir at room temperature for 6 hours if not stated otherwise. The mixture was diluted with ethyl acetate (30 mL), and extracted with saturated Na_2CO_3 (30 mL), water (30 mL) and brine (30 mL). The organic phase was dried over Na_2SO_4 and purified by flash column chromatography (SiO_2, 1% NEt_3 in cyclohexane /1% NEt_3 in ethyl acetate) to yield the aniline derivatives.

2.7.General procedure of the C–N cross-coupling of Cyclopropylamine (GP G)

A crimp cap vial equipped with a Teflon coated magnetic stirring bar was charged with [Pd(1-MeNAP)TFA]$_2$ (1.8 mg, 0.0025 mmol, 0.5 mol%) and *t*BuBrettPhos (2.42 mg, 0.005 mmol, 1 mol%). Degassed and dry toluene (1 mL) was added and the solution was stirred for 5 minutes. A second oven-dried crimp cap vial equipped with a Teflon coated magnetic stirring bar was charged with the aryl chloride (if solid, 0.5 mmol, 1.0 equiv.). The vial was entered inside a glovebox, where NaO*t*Bu (64 mg, 0.65 mmol, 1.3 equiv.), Cyclopropylamine (35 mg, 42 µl, 0.6 mmol, 1.2 equiv.) was added. The vial was sealed, transferred out of the glovebox and aryl chloride (if liquid, 0.5 mmol, 1.0 equiv.) was added *via* syringe under nitrogen unless mentioned otherwise. The catalyst solution was then transferred into the second vial and the first vial was washed with toluene (1 mL). The mixture was allowed to stir at room temperature for 22 hours if not stated otherwise. The mixture was diluted with ethyl acetate (30 mL), and extracted with saturated Na_2CO_3 (30 mL), water (30 mL) and brine (30 mL). The organic phase was dried over Na_2SO_4 and purified by flash column chromatography (SiO_2, 1% NEt_3 in cyclohexane /1% NEt_3 in ethyl acetate) to yield the cyclopropylamine derivatives.

*2.8.*General procedure of the *C–N* cross-coupling of Trifluoroethylamine (GP H)

A crimp cap vial equipped with a Teflon coated magnetic stirring bar was charged with [Pd(1-MeNAP)TFA]$_2$ (3.61 mg, 0.005 mmol, 1 mol%) and *t*BuBrettPhos (4.85 mg, 0.01 mmol, 2 mol%). Degassed and dry 1,4-dioxane (1 mL) was added and the solution was stirred for 5 minutes. A second oven-dried crimp cap vial equipped with a Teflon coated magnetic stirring bar was charged with the aryl chloride (if solid, 0.5 mmol, 1.0 equiv.). The vial was entered inside a glovebox, where NaO*t*Bu (68.8 mg, 0.6 mmol, 1.2 equiv.), 2,2,2-trifluoroethylamine (99 mg, 80 µl, 1.0 mmol, 2.0 equiv.) was added. The vial was sealed, transferred out of the glovebox and aryl chloride (if liquid, 0.5 mmol, 1.0 equiv.) was added *via* syringe under nitrogen unless mentioned otherwise. The catalyst solution was then transferred into the second vial and the first vial was washed with 1,4-dioxane (1 mL). The mixture was allowed to stir at room temperature for 16 hours if not stated otherwise. The mixture was diluted with ethyl acetate (30 mL), and extracted with saturated Na_2CO_3 (30 mL), water (30 mL) and brine (30 mL). The organic phase was dried over Na_2SO_4 and purified by flash column chromatography (SiO_2, 1% NEt_3 in cyclohexane /1% NEt_3 in ethyl acetate) to yield the trifluoroethylamine derivatives.

2.9. General procedure of the C–N cross-coupling of Aminothiazole (GP I)

A crimp cap vial equipped with a Teflon coated magnetic stirring bar was charged Pd(1-MeNAP)TFA]$_2$ (2.7 mg, 0.00375 mmol, 0.75 mol%) and *t*BuBrettphos (3.6 mg, 0.0075 mmol, 1.5 mol%).. Degassed and dry *t*BuOH (1 mL) was added and the solution was stirred for 5 minutes. A second oven-dried crimp cap vial equipped with a Teflon coated magnetic stirring bar was charged with the aryl bromide (if solid, 0.5 mmol, 1.0 equiv.) and 2-Aminothiazol (61.9 mg, 0.6 mmol, 1.2 equiv.). The vial was entered inside a glovebox, where K_2CO_3 (97 mg, 0.7 mmol, 1.4 equiv.) was added. The vial was sealed, transferred out of the glovebox and aryl bromide (if liquid, 0.5 mmol, 1.0 equiv.) was added *via* syringe under nitrogen unless mentioned otherwise. The catalyst solution was then transferred into the second vial and the first vial was washed with *t*BuOH (1 mL). The mixture was allowed to stir at 60 °C for 16 hours if not stated otherwise. The mixture was diluted with ethyl acetate (30 mL), and extracted with saturated Na_2CO_3 (30 mL), water (30 mL) and brine (30 mL). The organic phase was dried over Na_2SO_4 and purified by flash column chromatography (SiO_2, cyclohexane / ethyl acetate to yield the Aminothiazole derivatives.

2.10. General procedure of the Aminative C-F activation coupling (GP J)

A crimp cap vial equipped with a Teflon coated magnetic stirring bar was charged with [Pd(1-MeNAP)TFA]$_2$ (1.8 mg, 0.0025 mmol, 1.0 mol%) and BrettPhos (2.68 mg, 0.005 mmol, 2 mol%). Degassed and dry toluene (1 mL) was added and the solution was stirred for 5 minutes. A second oven-dried crimp cap vial equipped with a Teflon coated magnetic stirring bar was charged with the aryl fluoride (if solid, 0.25 mmol, 1.0 equiv.) and amine (if solid, 0.3 mmol, 1.2 equiv.). The vial was entered inside a glovebox, where LiHMDS (129 mg, 0.75 mmol, 3.0 equiv.) was added. The vial was sealed, transferred out of the glovebox. aryl fluoride (if liquid, 0.25 mmol, 1.0 equiv.) and amine (if liquid, 0.3 mmol, 1.2 equiv.) was added *via* syringe under nitrogen unless mentioned otherwise. The catalyst solution was then transferred into the second vial and the first vial was washed with toluene (1 mL). The mixture was allowed to stir at rt for 16 hours if not stated otherwise. The reaction was monitored by GC analysis, with n-hexadecane (30 µL) added as an internal standard.

2.11. General procedure of the Aminative Suzuki–Miyaura coupling (GP K)

A crimp cap vial equipped with a Teflon coated magnetic stirring bar was charged with [Pd(1-MeNAP)TFA]$_2$ (5.41 mg, 0.0075 mmol, 1.5 mol%) and *t*BuBrettPhos (7.27 mg, 0.015 mmol, 3 mol%). Degassed and dry THF (1 mL) was added and the solution was stirred for 5 minutes. A second oven-dried crimp cap vial equipped with a Teflon coated magnetic stirring bar was charged with the aryl triflate (if solid, 0.5 mmol, 1.0 equiv.), 4-(Trifluoromethyl)phenylboronic acid (194 mg, 1 mmol, 2 equiv.), O-Diphenylphosphinylhydroxylamine (175 mg, 0.75 mmol, 1.5 equiv.). The vial was entered inside a glovebox, where K_3PO_4 (433 mg, 2.0 mmol, 4.0 equiv.) was added. The vial was sealed, transferred out of the glovebox. aryl trilfate (if liquid, 0.5 mmol, 1.0 equiv.) was added *via* syringe under nitrogen unless mentioned otherwise. The catalyst solution was then transferred into the second vial and the first vial was washed with THF (1 mL). The mixture was allowed to stir at rt for 48 hours if not stated otherwise. The mixture was diluted with ethyl acetate (30 mL), and extracted with saturated Na_2CO_3 (30 mL), water (30 mL) and brine (30 mL). The organic phase was dried over $MgSO_4$ and purified by flash column chromatography (SiO_2, cyclohexane / ethyl acetate to yield the aniline derivates.

2.12. General procedure of the C–N cross-coupling of Triazole (GP L)

A crimp cap vial equipped with a Teflon coated magnetic stirring bar was charged with [Pd(1-MeNAP)TFA]$_2$ (3.61 mg, 0.005 mmol, 1.0 mol%) and *Me$_4$t*BuXphos (4.91 mg, 0.01 mmol, 2 mol%). Degassed and dry toluene (1 mL) was added and the solution was stirred for 5 minutes. A second oven-dried crimp cap vial equipped with a Teflon coated magnetic stirring bar was charged with the aryl bromide (if solid, 0.5 mmol, 1.0 equiv.), 1*H*-1,2,3-Triazole (41.4 mg, 0.6 mmol, 1.2 equiv.). The vial was entered inside a glovebox, where K_3PO_4 (212 mg, 1.0 mmol, 2.0 equiv.) was added. The vial was sealed, transferred out of the glovebox. aryl bromide (if liquid, 0.5 mmol, 1.0 equiv.) was added *via* syringe under nitrogen unless mentioned otherwise. The catalyst solution was then transferred into the second vial and the first vial was washed with toluene (1 mL). The mixture was allowed to stir at 120 °C for 5 hours if not stated otherwise. The mixture was diluted with ethyl acetate (30 mL), and extracted with saturated Na_2CO_3 (30 mL), water (30 mL) and brine (30 mL). The organic phase was dried over $MgSO_4$ and purified by flash column chromatography (SiO_2, cyclohexane / ethyl acetate to yield the triazole derivatives.

2.13. General procedure of the C–N cross-coupling of Imidazole (GP M)

A crimp cap vial equipped with a Teflon coated magnetic stirring bar was charged with [Pd(1-MeNAP)TFA]$_2$ (2.7 mg, 0.004 mmol, 0.75 mol%) and *Me$_4$t*BuXphos (4.42 mg, 0.009 mmol, 1.8 mol%). Degassed and dry toluene/1,4-dioxane (1 mL, 5:1) was added and the solution was stirred for 5 minutes. A second oven-dried crimp cap vial equipped with a Teflon coated magnetic stirring bar was charged with the aryl bromide (if solid, 0.5 mmol, 1.0 equiv.), 4-Methylimidazole (50.3 mg, 0.6 mmol, 1.2 equiv.). The vial was entered inside a glovebox, where K_3PO_4 (212 mg, 1.0 mmol, 2.0 equiv.) was added. The vial was sealed, transferred out of the glovebox. aryl bromide (if liquid, 0.5 mmol, 1.0 equiv.) was added *via* syringe under nitrogen unless mentioned otherwise. The catalyst solution was then transferred into the second vial and the first vial was washed with toluene/1,4-dioxane (1 mL, 5:1). The mixture was allowed to stir at 120 °C for 5 hours if not stated otherwise. The mixture was diluted with ethyl acetate (30 mL), and extracted with saturated Na_2CO_3 (30 mL), water (30 mL) and brine (30 mL). The organic phase was dried over $MgSO_4$ and purified by flash column chromatography (SiO_2, cyclohexane / ethyl acetate to yield the imidazole derivatives.

2.14. General procedure of the C–CN cross-coupling of $K_3[Fe(CN)_6]$. (GP N)

A crimp cap vial equipped with a Teflon coated magnetic stirring bar was charged with [Pd(1-MeNAP)TFA]$_2$ (1.5 mg, 0.002 mmol, 0.2 mol%) and XPhos (2.0 mg, 0.004 mmol, 0.4 mol%). Degassed and dry 1,4-dioxane (0.5 mL) was added and the solution was stirred for 5 minutes. A second oven-dried crimp cap vial equipped with a Teflon coated magnetic stirring bar was charged with the aryl chloride (if solid, 1.0 mmol, 1.0 equiv.), $K_3[Fe(CN)_6]$ (212 mg, 0.5 mmol, 0.5 equiv.). The vial was entered inside a glovebox, where KOAc (12 mg, 0.125 mmol, 0.125 equiv.) was added. The vial was sealed, transferred out of the glovebox. aryl chloride (if liquid, 1.0 mmol, 1.0 equiv.) and degassed water (1 ml) was added *via* syringe under nitrogen unless mentioned otherwise. The catalyst solution was then transferred into the second vial and the first vial was washed with 1,4-dioxane (0.5 mL). The mixture was allowed to stir at 100 °C for 1 hours if not stated otherwise. The mixture was diluted with ethyl acetate (30 mL), and extracted with saturated Na_2CO_3 (30 mL), water (30 mL) and brine (30 mL). The organic phase was dried over $MgSO_4$ and purified by flash column chromatography (SiO_2, cyclohexane / ethyl acetate to yield the Nitrile derivatives.

2.15. General procedure of the Coupling of KOH (GP O)

A crimp cap vial equipped with a Teflon coated magnetic stirring bar was charged with [Pd(1-MeNAP)TFA]$_2$ (3.6 mg, 0.005 mmol, 1 mol%) and tBuBrettPhos (4.95 mg, 0.01 mmol, 2 mol%). Degassed and dry dioxane (1 mL) was added and the solution was stirred for 5 minutes. A second oven-dried crimp cap vial equipped with a Teflon coated magnetic stirring bar was charged with the aryl chloride (if solid, 0.5 mmol, 1.0 equiv.). The vial was entered inside a glovebox, where KOH (84 mg, 1.5 mmol, 3 equiv.) was added. The vial was sealed, transferred out of the glovebox. aryl chloride (if liquid, 0.5 mmol, 1.0 equiv.) and degassed water (180 µL, 10 mmol, 20 equiv.) was added *via* syringe under nitrogen unless mentioned otherwise. The catalyst solution was then transferred into the second vial and the first vial was washed with dry dioxane (1 mL) The mixture was allowed to stir at 60 for 20 hours if not stated otherwise. The reaction was monitored by GC analysis, with n-hexadecane (20 µL) added as an internal standard. The mixture was then acidified with aqueous HCl solution (1 M, 1 mL). The resulting reaction mixture in the capped test tube was agitated until all of the solid was dissolved into the reaction mixture. The reaction mixture was then neutralized with saturated NaHCO3 solution (2 mL). After that, open caps dilute it with EA. 1 ml of org. Phase into workup vial (3 ml water, 3ml EtOAc), wash org. phase and then take 0.5 ml of org. phase, filter through glass pipette with cotton, silica and $MgSO_4$ into GC vial.

2.16. General procedure of the C–O cross-coupling of n-Butanol (GP P)

A crimp cap vial equipped with a Teflon coated magnetic stirring bar was charged with [Pd(1-MeNAP)TFA]$_2$ (3.6 mg, 0.005 mmol, 1 mol%) and AdCyBrettPhos (5.9 mg, 0.01 mmol, 2 mol%). Degassed and dry 1,4-dioxane (0.5 mL) was added and the solution was stirred for 5 minutes. A second oven-dried crimp cap vial equipped with a Teflon coated magnetic stirring bar was charged with the aryl chloride (if solid, 0.5 mmol, 1.0 equiv.). The vial was entered inside a glovebox, where NaO*t*Bu (58 mg, 0.6 mmol, 1.2 equiv.) was added. The vial was sealed, transferred out of the glovebox, dry 1-Butanol (74.1 mg, 92 µl, 1.0 mmol, 2.0 equiv.) and aryl chloride (if liquid, 0.5 mmol, 1.0 equiv.) was added *via* syringe under nitrogen unless mentioned otherwise. The catalyst solution was then transferred into the second vial and the first vial was washed with 1,4-dioxane (0.5 mL). The mixture was allowed to stir at room temperature for 16 hours if not stated otherwise. The mixture was diluted with ethyl acetate (30 mL), and extracted with saturated Na_2CO_3 (30 mL), water (30 mL) and brine (30 mL). The organic phase was dried over $MgSO_4$ and purified by flash column chromatography (SiO_2, cyclohexane / ethyl acetate to yield the Alcohol derivatives.

2.17. General procedure of the Coupling of Methanol (GP Q)

A crimp cap vial equipped with a Teflon coated magnetic stirring bar was charged with [Pd(1-MeNAP)TFA]$_2$ (1.8 mg, 0.0025 mmol, 0.5 mol%) and tBuBrettPhos (2.47 mg, 0.005 mmol, 1 mol%). Degassed and dry dioxane (0.5 mL) was added and the solution was stirred for 5 minutes. A second oven-dried crimp cap vial equipped with a Teflon coated magnetic stirring bar was charged with the aryl chloride (if solid, 0.5 mmol, 1.0 equiv.). The vial was entered inside a glovebox, where NaO*t*Bu (67.3 mg, 0.7 mmol, 1.4 equiv.) was added. The vial was sealed, transferred out of the glovebox. aryl chloride (if liquid, 0.5 mmol, 1.0 equiv.) and dry methanol (101 µL, 2.5 mmol, 5 equiv.) was added *via* syringe under nitrogen unless mentioned otherwise. The catalyst solution was then transferred into the second vial and the first vial was washed with dry dioxane (0.5 mL) The mixture was allowed to stir at rt for 20 hours if not stated otherwise. The reaction was monitored by GC analysis, with n-hexadecane (20 µL) added as an internal standard.

2.18. General procedure of the C–F cross-coupling of CsF (GP R)

A crimp-cap vial equipped with a Teflon-coated magnetic stirring bar was charged with [Pd(1-MeNAP)TFA]$_2$ (7.2 mg, 0.01 mmol, 2 mol%) and *tBu*BrettPhos (14.8 mg, 0.03 mmol, 6 mol%). A second oven-dried crimp-cap vial equipped with a Teflon-coated magnetic stirring bar was charged with the aryl triflate (0.5 mmol, 1.0 equiv.). The vial was transferred into a glovebox, where CsF (152 mg, 1.0 mmol, 2.0 equiv.) was added. Degassed and dry toluene (1.5 mL) was added to the first vial containing the catalyst inside the glovebox, and the solution was stirred for 5 min. The catalyst solution was then transferred to the second vial, and the first vial was washed with additional toluene (1.0 mL). The vial was sealed and removed from the glovebox. The reaction mixture was stirred at 90 °C for 18 h unless stated otherwise. After cooling to room temperature, the mixture was diluted with ethyl acetate (30 mL) and washed successively with saturated Na_2CO_3 (30 mL), water (30 mL), and brine (30 mL). The organic layer was dried over $MgSO_4$, filtered, and concentrated under reduced pressure. The crude product was purified by flash column chromatography on silica gel (SiO_2) using cyclohexane/ethyl acetate as eluent to afford the fluorinated aromatic products.

2.19. General procedure of the Coupling of Tertbutylamine (GP S)

A crimp cap vial equipped with a Teflon coated magnetic stirring bar was charged with [Pd(1-MeNAP)TFA]$_2$ (1.8 mg, 0.0025 mmol, 0.5 mol%) and RuPhos (2.41 mg, 0.005 mmol, 1 mol%). Degassed and dry THF (1 mL) was added and the solution was stirred for 5 minutes. A second oven-dried crimp cap vial equipped with a Teflon coated magnetic stirring bar was charged with the aryl chloride (if solid, 0.5 mmol, 1.0 equiv.). The vial was entered inside a glovebox, where KOtBu (95 mg, 0.75 mmol, 1.6 equiv.) was added. The vial was sealed, transferred out of the glovebox. aryl chloride (if liquid, 0.5 mmol, 1.0 equiv.) and tertbutylamine (59 µL, 0.55 mmol, 1.1 equiv.) was added *via* syringe under nitrogen unless mentioned otherwise. The catalyst solution was then transferred into the second vial and the first vial was washed with dry THF (1 mL) The mixture was allowed to stir at rt for 2 hours if not stated otherwise. The reaction was monitored by GC analysis, with n-undecane (30 µL) added as an internal standard.

2.20. General procedure of the α-Arylation of Ketones (GP T)

A crimp cap vial equipped with a Teflon coated magnetic stirring bar was charged with [Pd(1-MeNAP)TFA]$_2$ (3.6 mg, 0.005 mmol, 1 mol%) and XPhos (4.9 mg, 0.01 mmol, 2 mol%). Degassed and dry THF (1 mL) was added and the solution was stirred for 5 minutes. A second oven-dried crimp cap vial equipped with a Teflon coated magnetic stirring bar was charged with the aryl chloride (if solid, 0.5 mmol, 1.0 equiv.). The vial was entered inside a glovebox, where KOtBu (115 mg, 1.0 mmol, 2.0 equiv.) was added. The vial was sealed, transferred out of the glovebox. aryl chloride (if liquid, 0.5 mmol, 1.0 equiv.) and Acetophenon (66 µL, 0.55 mmol, 1.1 equiv.) was added *via* syringe under nitrogen unless mentioned otherwise. The catalyst solution was then transferred into the second vial and the first vial was washed with dry THF (1 mL) The mixture was allowed to stir at rt for 20 hours if not stated otherwise. The reaction was monitored by GC analysis, with n-tetradecane (50 µL) added as an internal standard.

2.21. General procedure of the Coupling of Boronic acids (GP U)

A crimp cap vial equipped with a Teflon coated magnetic stirring bar was charged with [Pd(1-MeNAP)TFA]$_2$ (3.6 mg, 0.005 mmol, 1 mol%) and IPr*OMe (9.65 mg, 0.01 mmol, 2 mol%). Degassed and dry THF (1 mL) was added and the solution was stirred for 5 minutes. A second oven-dried crimp cap vial equipped with a Teflon coated magnetic stirring bar was charged with the aryl chloride (if solid, 0.5 mmol, 1.0 equiv.) and boronic acid (0.75 mmol, 1,5 equiv.). The vial was entered inside a glovebox, where KOH (32 mg, 1.0 mmol, 2.0 equiv.) was added. The vial was sealed, transferred out of the glovebox. aryl chloride (if liquid, 0.5 mmol, 1.0 equiv.) was added *via* syringe under nitrogen unless mentioned otherwise. The catalyst solution was then transferred into the second vial and the first vial was washed with dry THF (1 mL) The mixture was allowed to stir at rt for 16 hours if not stated otherwise. The reaction was monitored by GC analysis, with n-undecane (50 µL) added as an internal standard.

3. Screening tables of the reaction conditions and additional experiments

3.1. Screening of the palladium source for the C–N cross-coupling of Amide

Following the general procedure (GP A), optimization reactions were monitored by GC analysis, with n-hexadecane (20 µL) added as an internal standard to a freshly prepared stock solution of *t*-BuOH and the corresponding aryl chloride. For reactions with additives, 2 mol% was added. *N*-Allylbenzamide[1], Styrenebenzaamide[2] were isolated according to literature procedure.

Table S1: Screening of palladium sources for the C–N cross-coupling of Amide[a]

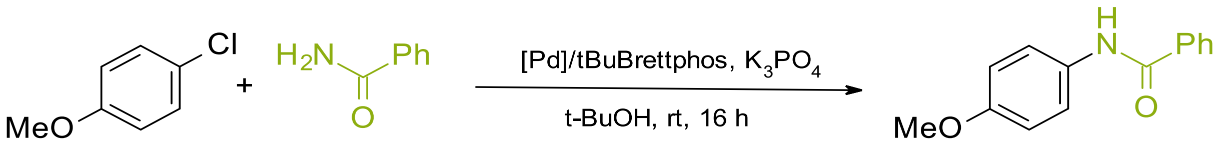

1 2 3a

Entry	Pd source	Additive	Yield 3a [%]
**1**	$Pd_2(dba)_3$	-	<1
**2**	$Pd(OAc)_2$	-	<1
**3**	$[Pd(1\text{-}Menap)TFA]_2$	-	>99
**4**	G3 Dimer	-	<1
**5**	$[Pd(allyl)Cl]_2$	-	31
**6**	$[Pd(cinnamyl)Cl]_2$	-	32
**7**	$[Pd(1\text{-}MeNAP)Br]_2$	-	42
**11**	t-BuBrettPhos Pd G6 bromide	-	60
**14**	$[Pd(1\text{-}Menap)TFA]_2$	dba	0
**15**	"	Styrenebenzaamide	52
**16**	"	Allylbenzamide	0
**17**	"	carbazole	45
**18**	With out Pd source	-	0
**19**	With out Pd and ligand	-	0
**20**	$[Pd(1\text{-}Menap)TFA]_2$	-	99[b]

[a]Reaction conditions: **1** (0.5 mmol), **Benzamide** (1.2 equiv.), **[Pd]** (1 mol%), *t*BuBrettPhos (2 mol%), K_3PO_4 (1.4 equiv.), *t*BuOH (3 mL). Yields were determined by GC using *n*-hexadecane as internal standard. 2 mol% Additive.[b]The reaction was performed using a new vial and a new stir bar, and the solids were weighed using a new spatula to avoid contamination with palladium.

3.2.Screening of the palladium source for the C–N cross-coupling of Ammonium acetate

Following the general procedure (GP F), optimization reactions were monitored by GC analysis, with *n*-tetradecane (30 µL) added as an internal standard to a freshly prepared stock solution of 1,4-Dioxane and the corresponding aryl chloride.

Table S2: Screening of palladium sources for the C–N cross-coupling of Ammonium acetate[a]

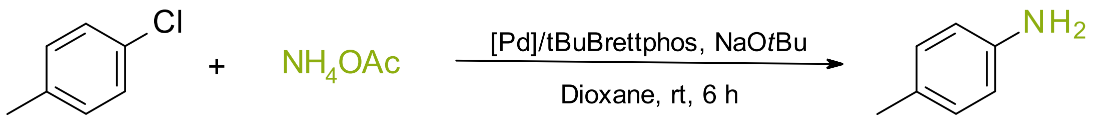

S1 **S2** **8a**

**Entry**	**Pd source**	**Yield 8a** [%]
1	$Pd_2(dba)_3$	0
2	$Pd(OAc)_2$	0
3	$[Pd(1\text{-menap) Br}]_2$	0
4	$[Pd(cinnamyl)Cl]_2$	0
5	$[Pd(allyl)Cl]_2$	0
6	G3 dimer	0
7	$[Pd(1\text{-MeNAP) TFA}]_2$	99
10	tBuBrettphos Pd G3	19

[a]Reaction conditions: **S1** (0.5 mmol), **NH_4OAc** (3.0 equiv.), [**Pd**] (0.5 mol%), *t*BuBrettPhos (1 mol%), NaO*t*Bu (4.0 equiv.), 1,4-Dioxane (5 mL). Yields were determined by GC using *n*-hexadecane as internal standard.

4.NMR Experiments

4.1.Preformation of Palladium(II) complexes incorporating sterically bulky ligands

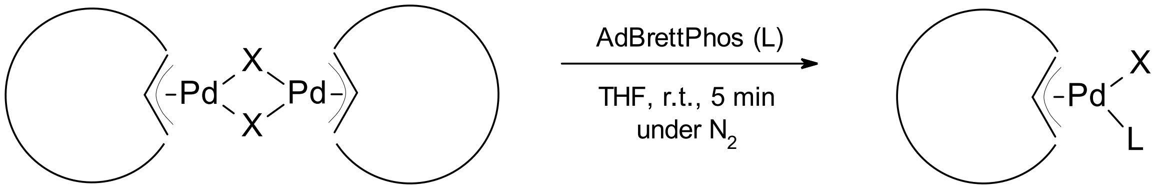

Inside a nitrogen-filled glovebox, an NMR tube was charged with [Pd] (0.125 mmol, 1 equiv) and AdBrettPhos (16.9 mg, 0.250 mmol, 2 equiv) and dry and degassed THF (0.4 mL) was added. The tube was then sealed and removed from the glovebox. After 5 minutes, a ^{31}P NMR spectrum was recorded.

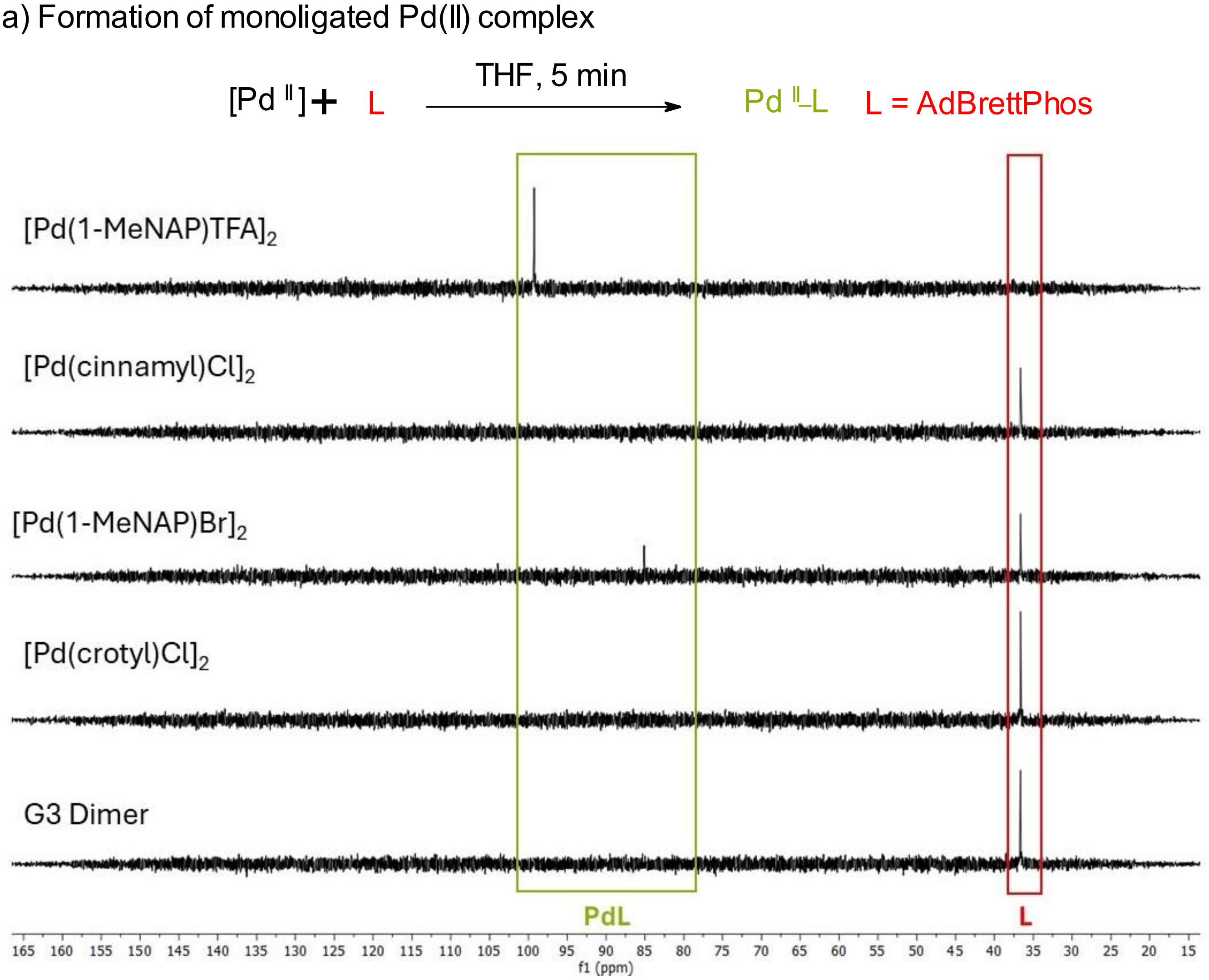

Figure S1: ^{31}P NMR spectra of preformed [Pd(AdBrettPhos)] complexes recorded 5 min after removal from a nitrogen-filled glovebox, prepared from different palladium precursors.

4.2.Preformation of [Pd(MeNAP)TFA]2 incorporating different ligands

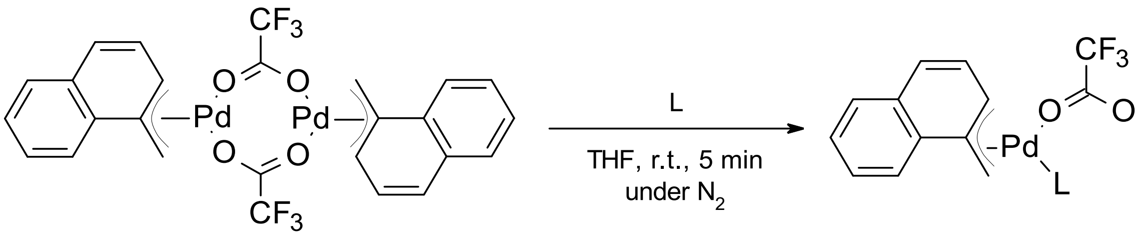

Inside a nitrogen-filled glovebox, an NMR tube was charged with [Pd(MeNAP)TFA]2 (9.0 mg, 0.125 mmol, 1 equiv) and Ligand (0.250 mmol, 2 equiv) and dry and degassed THF (0.4 mL) was added.The tube was then sealed and removed from the glovebox. After 5 minutes, a ^{31}P NMR spectrum was recorded.

L:

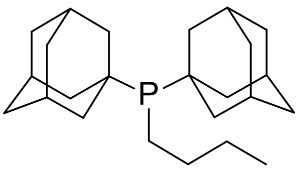

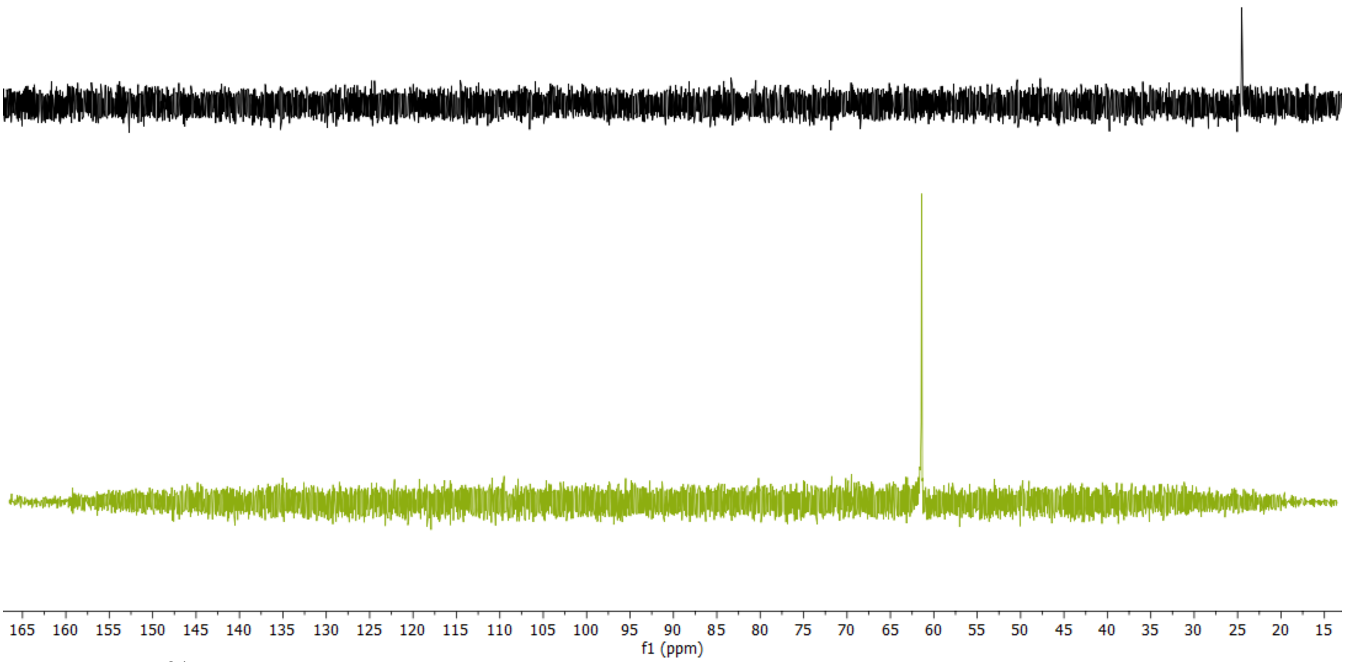

Figure S2: ^{31}P NMR spectra of cataCXium® A (Top) and [Pd(cataCXium® A)] complex (bottom) recorded 5 min after removal from a nitrogen-filled glovebox.

L:

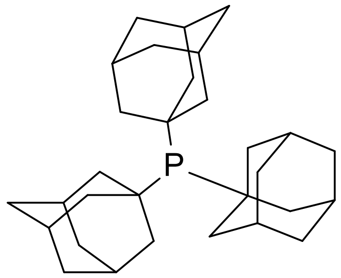

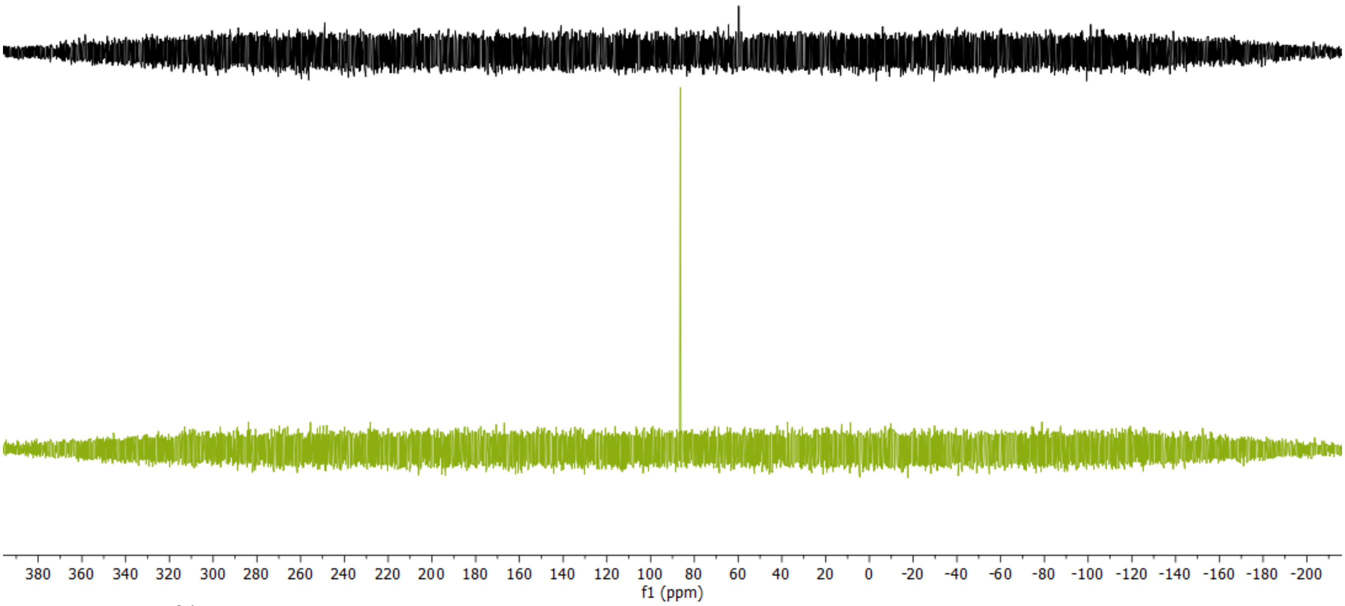

Figure S3: ^{31}P NMR spectra of PAd_3 (Top) and $[Pd(PAd_3)]$ complex (bottom) recorded 5 min after removal from a nitrogen-filled glovebox.

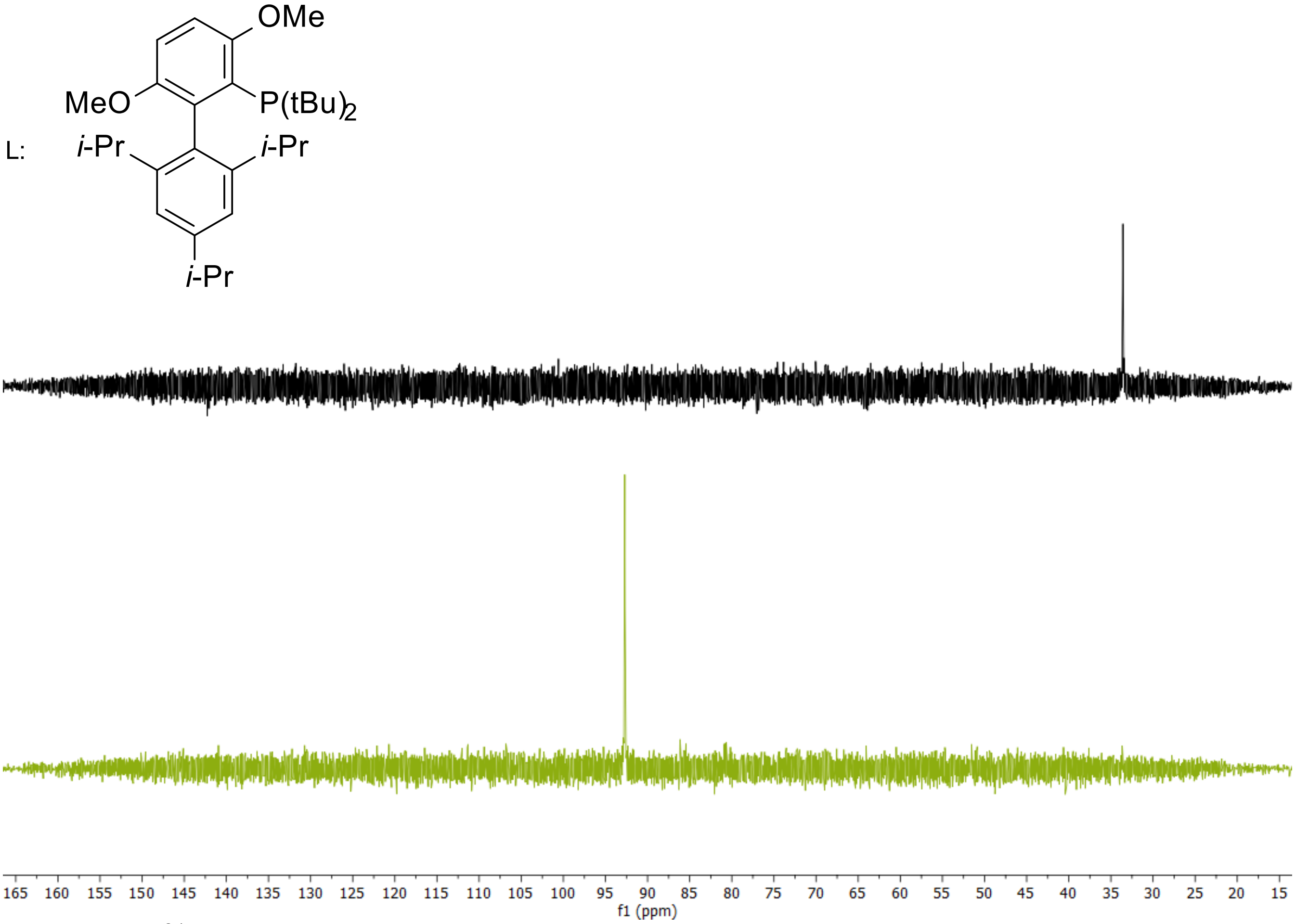

Figure S4: ^{31}P NMR spectra of *t*BuBrettPhos (Top) and [Pd(*t*BuBrettPhos)] complex (bottom) recorded 5 min after removal from a nitrogen-filled glovebox.

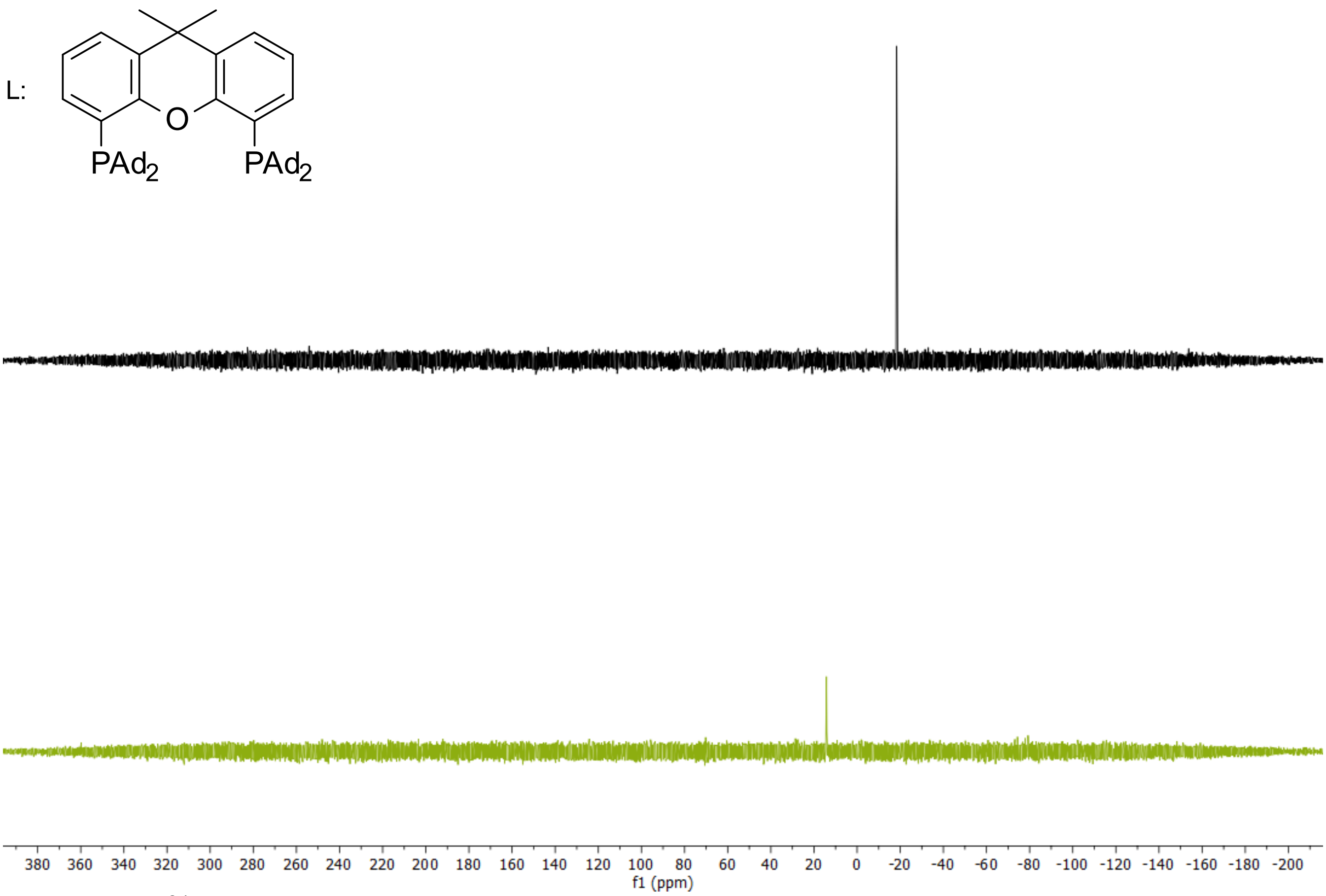

Figure S5: ^{31}P NMR spectra of XantPhos (Top) and [Pd(XantPhos)] complex (bottom) recorded 5 min after removal from a nitrogen-filled glovebox.

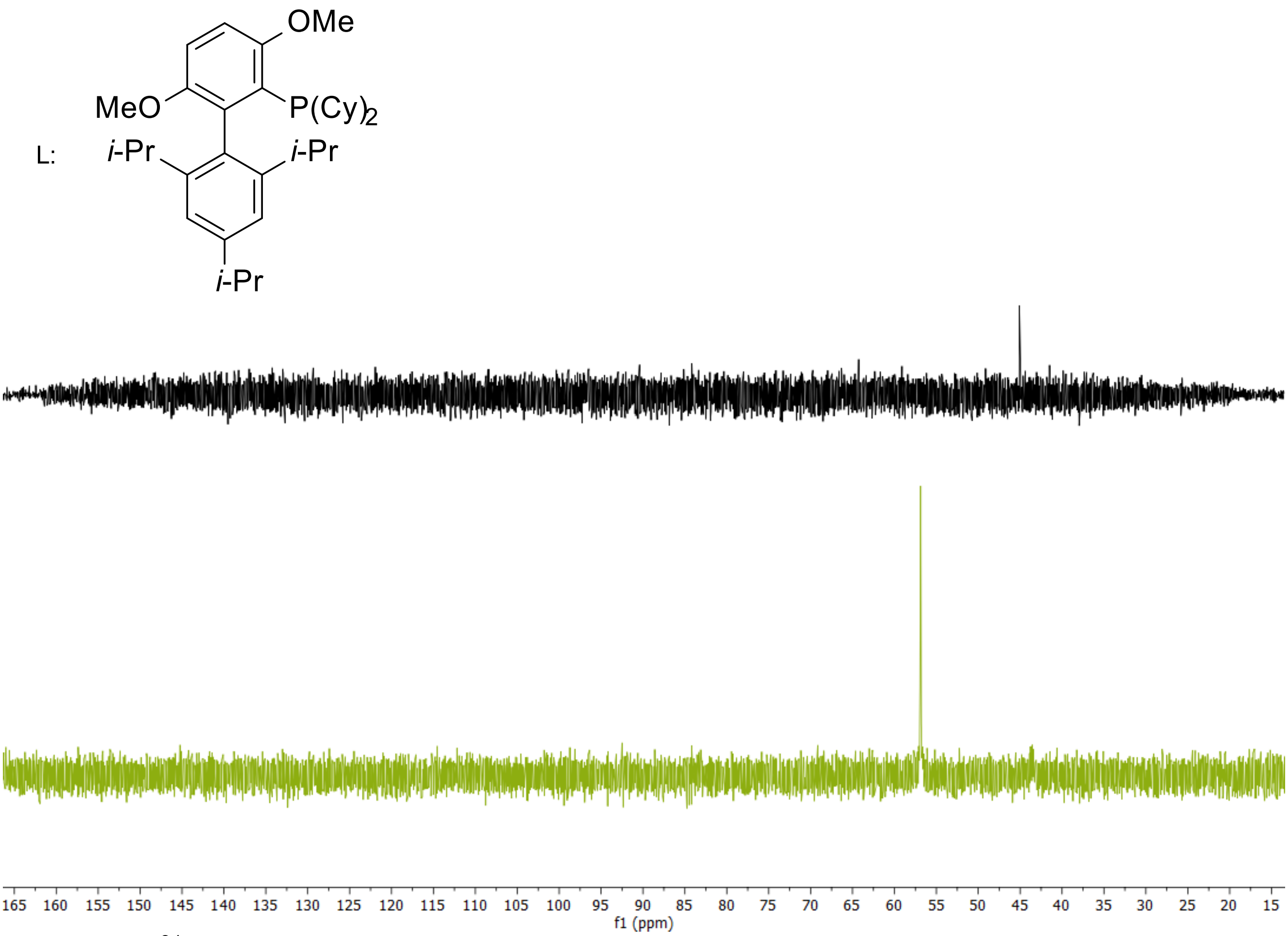

Figure S6: ^{31}P NMR spectra of BrettPhos (Top) and [Pd(BrettPhos)] complex (bottom) recorded 5 min after removal from a nitrogen-filled glovebox.

L:

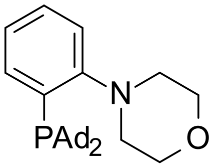

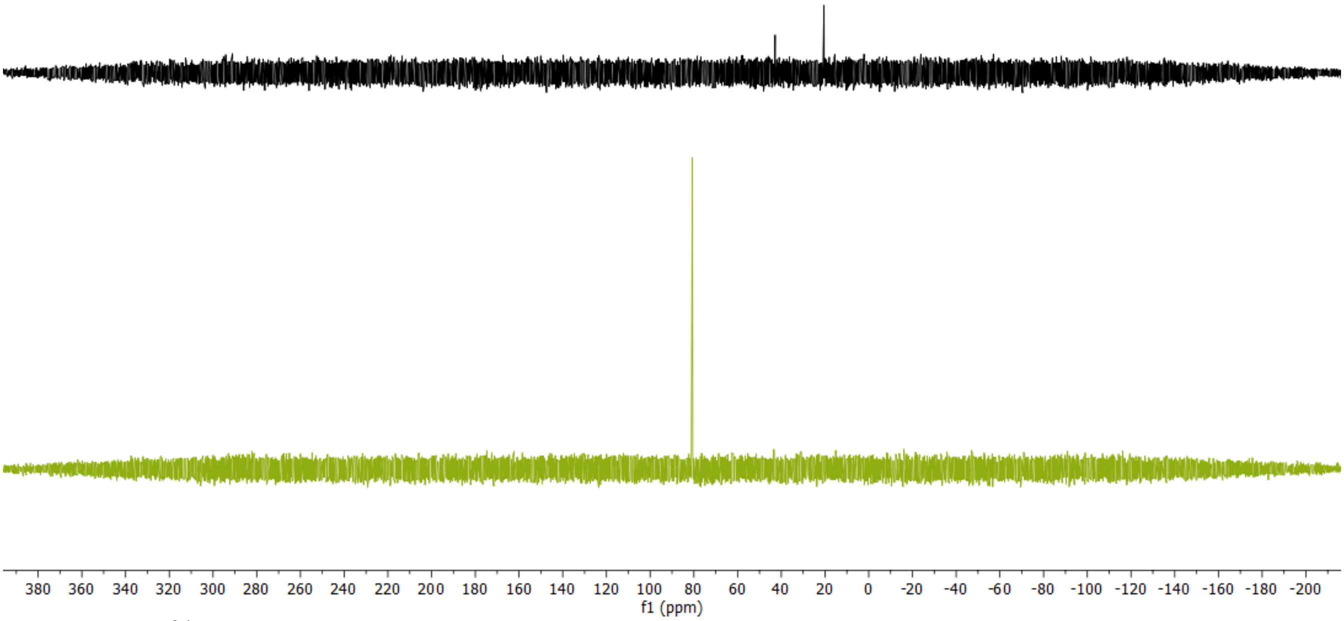

Figure S7: ^{31}P NMR spectra of MorDalPhos (Top) and [Pd(MorDalPhos)] complex (bottom) recorded 5 min after removal from a nitrogen-filled glovebox.

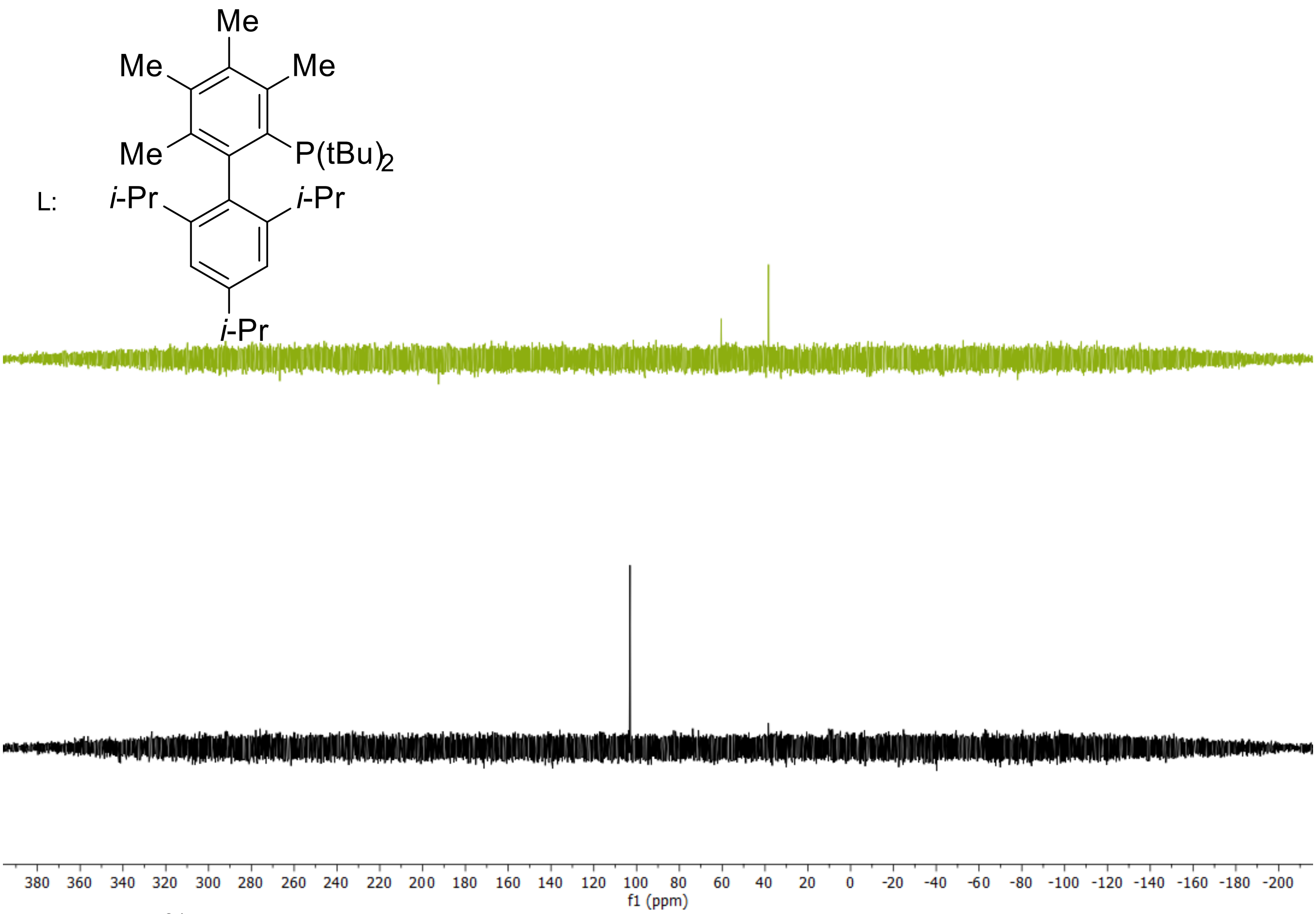

Figure S8: ^{31}P NMR spectra of Me4tButylXphos (Top) and [Pd(Me4tButylXphos)] complex (bottom) recorded 5 min after removal from a nitrogen-filled glovebox.

4.3. Stability of [Pd(MeNAP)TFA]$_2$ in different solvents

An NMR tube was charged with [Pd(MeNAP)TFA]$_2$ (7.2 mg, 0.010 mmol, 1.0 equiv) and methyl 3,5-dinitrobenzoate (2.3 mg, 0.010 mmol, 1.0 equiv, internal standard). Deuterated solvent (0.4 mL) was then added under air. The mixture was briefly shaken to ensure homogeneity. Quantitative ^{1}H NMR spectra were recorded at regular intervals (every few hours) to monitor the decomposition process.

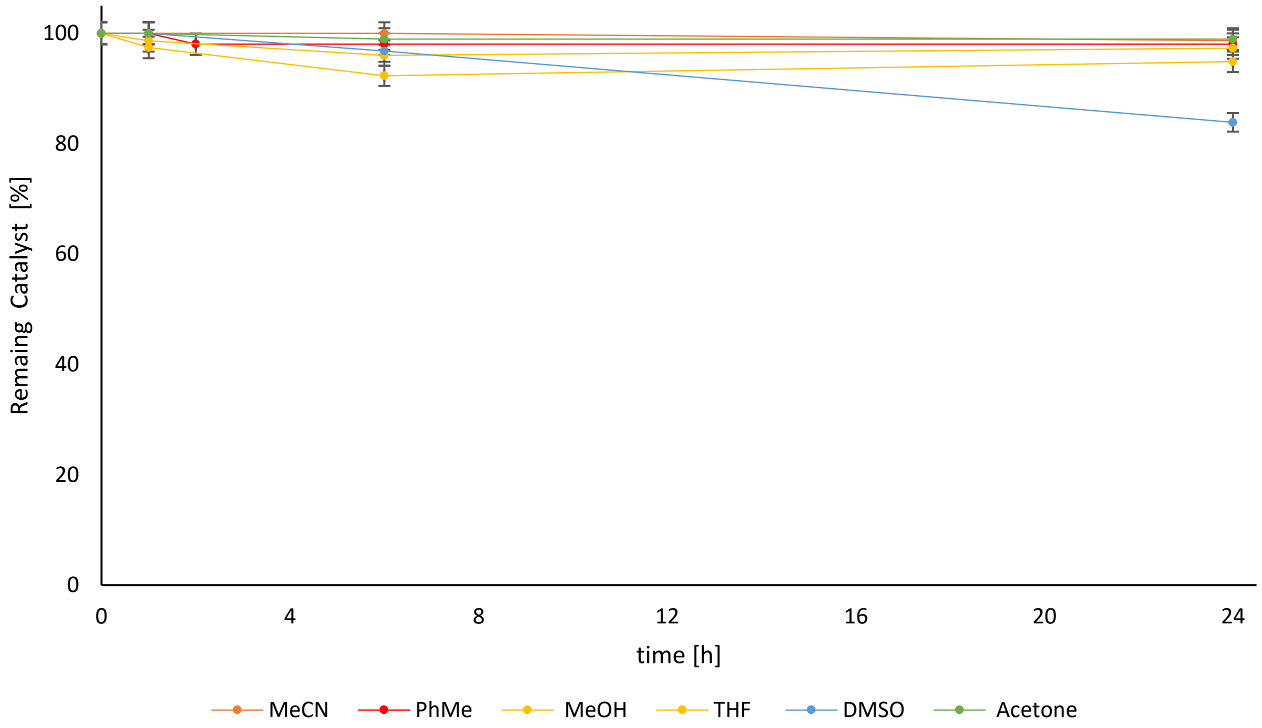

Figure S9: Time-resolved ^{1}H NMR monitoring of [Pd(MeNAP)TFA]$_2$ decomposition in various deuterated solvents under air.

5.Mechanistic studies

To a 20 mL vial charged with 0.05 mmol of [Pd(MeNAP)TFA]2, Nucleophile (5.0 equiv; Benzamid or Ammonium Acetate), *t*BuBrettphos (2.0 equiv), Base (6.0 equiv; K_3PO_4 or NaO*t*Bu) were added in the glovebox. 1.0 mL of the respective solvent (*t*BuOH or 1,4-Dioxane) and 20 µL of n-hexadecane were added and the reaction mixture was stirred outside the glovebox for 4 hours at room temperature. The mixture was then diluted with ethyl acetate, washed with Na_2CO_3 solution, filtered over celite/Mg_2SO_4 and analysed via GC and GCMS.

5.1.Catalyst Activation in the Buchwald-Hartwig amination of Amides

To shed some light on the mechanism of catalyst activation in the Buchwald Hartwig amination, we performed a reaction of [Pd(MeNAP)TFA]2 with ligand, base and excess amount of Benzamide. The amination product was formed as major product, whereas the methylnaphthalene was found as minor product in a ratio of 1:1.5 and the methylnaphthyl bromide was not detected at all.

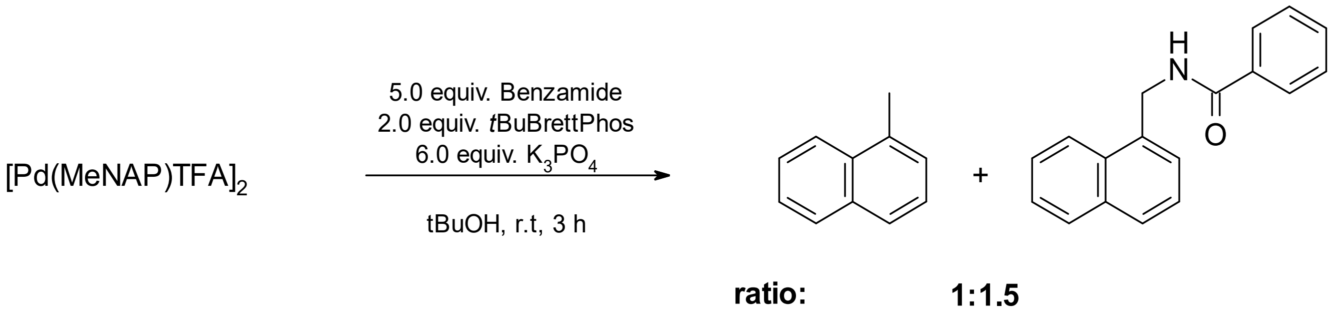

5.2.Catalyst Activation in the Buchwald-Hartwig amination of Ammonium acetate

In order to elucidate the catalyst activation in the Buchwald Hartwig amination, we performed a reaction of [Pd(MeNAP)TFA]2 with ligand, base and excess amount of Ammoniumacetet. The amination product was formed as major product, whereas the methylnaphthalene was found as minor product in a ratio of 1:3.9 and the methylnaphthyl bromide was not detected at all.

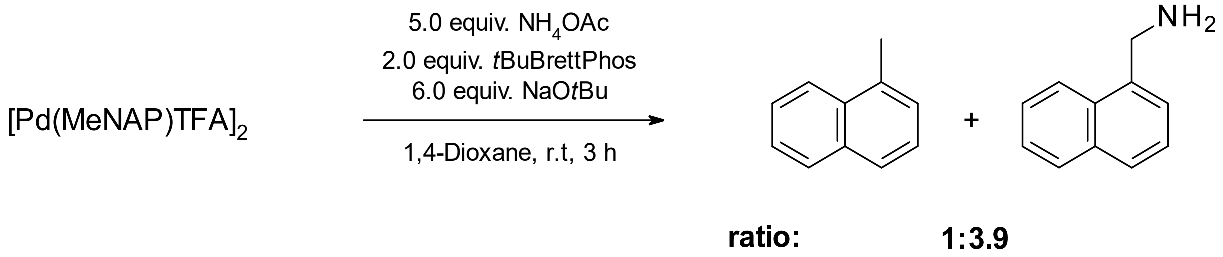

5.3. ^{31}P NMR catalyst activation in the Buchwald-Hartwig amination of Amides

To a 20 mL vial charged with 0.04 mmol of $[Pd(MeNAP)TFA]_2$, Nucleophile (5.0 equiv; Benzamid), preformed complex (1.0 equiv), Base (6.0 equiv; K_3PO_4) were added in the glovebox. 1.5 mL of *t*BuOH were added and the reaction mixture was stirred outside the glovebox for 4 hours at room temperature. The mixture was then diluted with tetrahydrofuran inside the glovebox and a ^{31}P NMR spectrum was recorded.

b) Activation of different Pd precursor

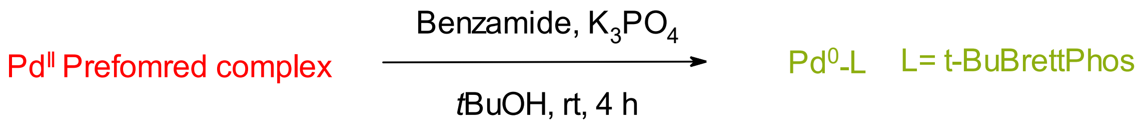

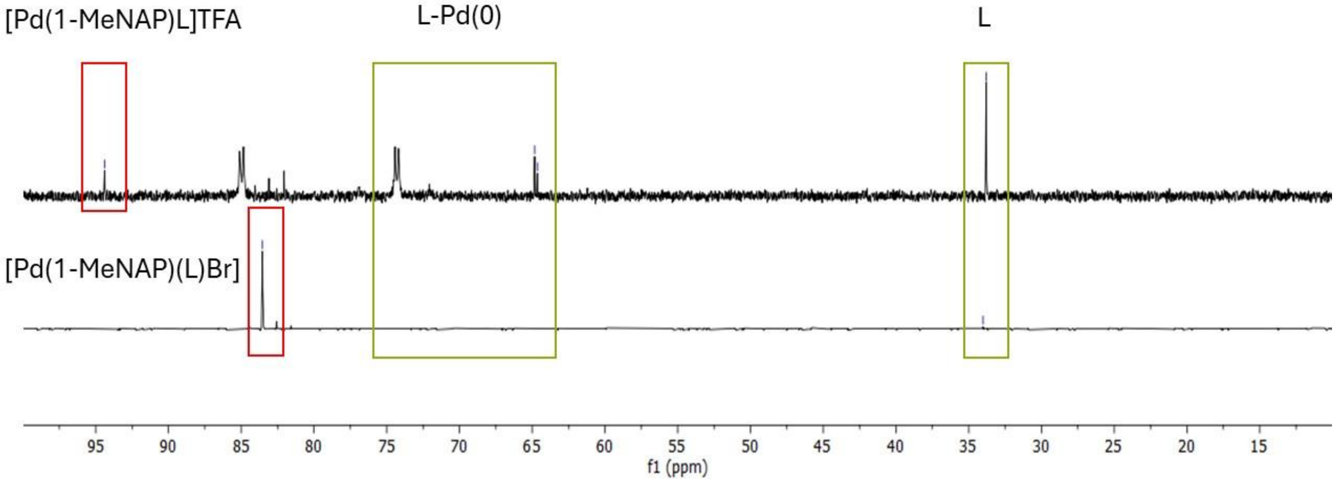

Figure S10: ^{31}P NMR spectra of [Pd(1-MeNAP)(tBubrettphos)Br] (Top) and [Pd(1-MeNAP)(tBubrettphos)TFA] (bottom) recorded 4 hours after stirring with base and nucleophile at room temperature.

5.4 Pd(0) trap experiment

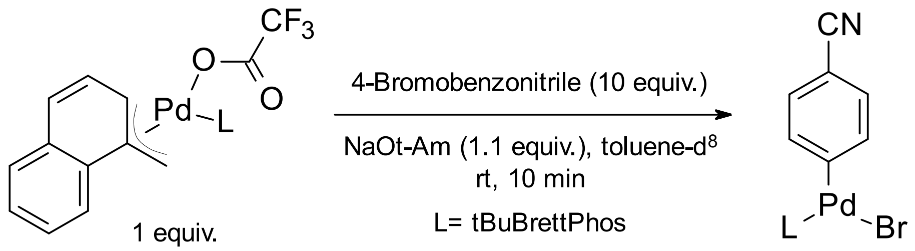

In an oven- dried vial, [Pd(1- MeNAP) tBuBrettPhos] TFA (1.0 equiv) was weighed outside the glovebox. The vial was then transferred into a glovebox, where NaOtAm (1.1 equiv) and 4-bromobenzonitrile (10 equiv) were added sequentially. Toluene-d_8 was subsequently introduced, and the resulting mixture was allowed to stand for 10 min. An NMR sample was then prepared inside the glovebox, and the reaction was analyzed by ^{31}P-NMR spectroscopy. The yield was determined by relative integration of the ^{31}P-NMR signals.

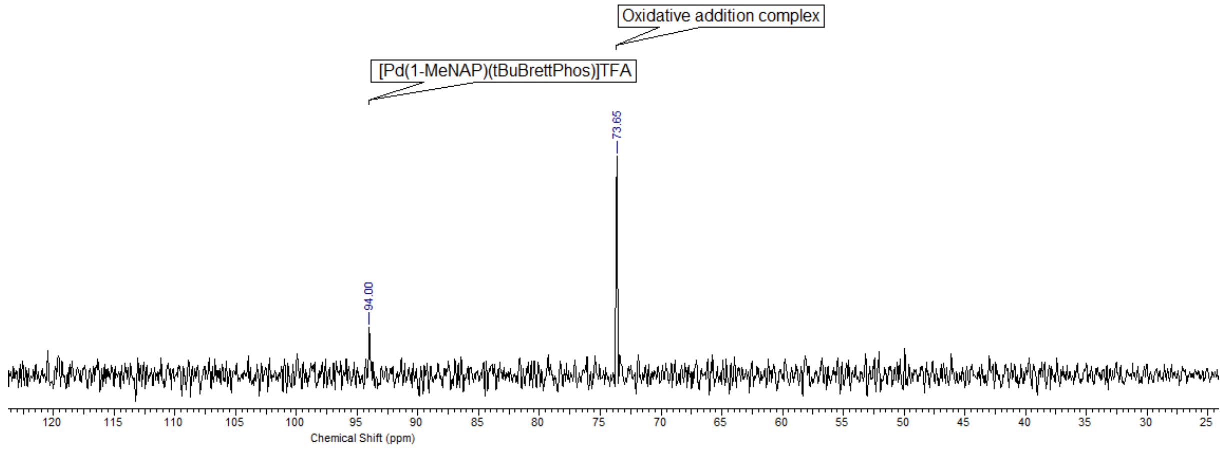

Figure S11: ^{31}P NMR spectra of the Pd(0)-trapping experiment following the addition of ArBr.

5.5 Mercury droplet Expeiment

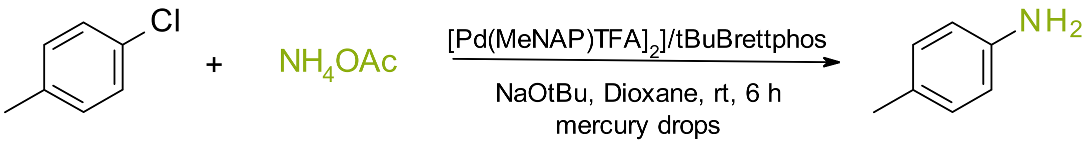

S1 **S2** **8a**

A crimp cap vial equipped with a Teflon coated magnetic stirring bar was charged with [Pd(1-MeNAP)TFA]$_2$ (1.8 mg, 0.0025 mmol, 0.5 mol%) and tBuBrettPhos (2.42 mg, 0.005 mmol, 1 mol%). Degassed and dry 1,4-Dioxane (2.5 mL) was added and the solution was stirred for 5 minutes. A second oven-dried crimp cap vial equipped with a Teflon coated magnetic stirring bar was charged with the aryl chloride (if solid, 0.5 mmol, 1.0 equiv.). The vial was entered inside a glovebox, where NaOtBu (192 mg, 2 mmol, 4.0 equiv.), NH_4OAc (116 mg, 1.5 mmol, 3.0 equiv.) was added. The vial was sealed, transferred out of the glovebox and aryl chloride (if liquid, 0.5 mmol, 1.0 equiv.) was added via syringe under nitrogen. The catalyst solution was then transferred into the second vial and the first vial was washed with 1,4-Dioxane (2.5 mL). The mixture was allowed to stir at room temperature for 0.50 hours after that one droplet of liquid mercury was added to vial (inside Glovebox). The resulting mixture was stirred at rt for 6 h. The reaction was afterwards analyzed by GC-FID.

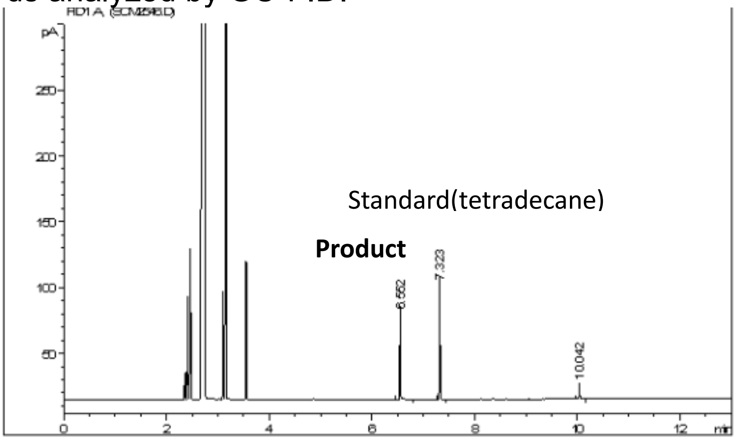

Area Percent Report

Sorted By : Signal
Multiplier : 1.0000
Dilution : 1.0000
Use Multiplier & Dilution Factor with ISTDs

Signal 1: FID1 A,

Peak #	RetTime [min]	Type	Width [min]	Area [pA*s]	Height [pA]	Area %
1	6.552	VB	0.0176	64.53773	64.80177	38.78676
2	7.323	VV	0.0176	86.52008	87.36459	51.99801
3	10.042	VB	0.0213	15.33334	11.67651	9.21524

Figure S12: GC-FID chromatogram of the reaction mixture from the mercury droplet experiment. The peak at 6.552 ppm corresponds to the product, and the peak at 7.323 ppm corresponds to the internal standard (tetradecane).

5.6 Filtration Experiment

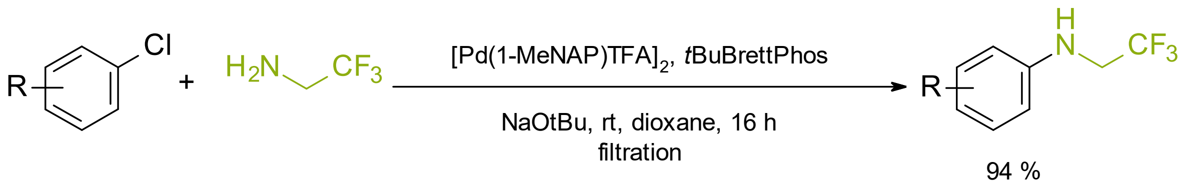

A crimp cap vial equipped with a Teflon coated magnetic stirring bar was charged with [Pd(1-MeNAP)TFA]$_2$ (3.61 mg, 0.005 mmol, 1 mol%) and tBuBrettPhos (4.85 mg, 0.01 mmol, 2 mol%). Degassed and dry 1,4-dioxane (1 mL) was added and the solution was stirred for 5 minutes. A second oven-dried crimp cap vial equipped with a Teflon coated magnetic stirring bar was charged with the aryl chloride (if solid, 0.5 mmol, 1.0 equiv.). The vial was entered inside a glovebox, where NaOtBu (68.8 mg, 0.6 mmol, 1.2 equiv.), 2,2,2-trifluoroethylamine (99 mg, 80 µl, 1.0 mmol, 2.0 equiv.) was added. The vial was sealed, transferred out of the glovebox and aryl chloride (if liquid, 0.5 mmol, 1.0 equiv.) was added via syringe under nitrogen. The catalyst solution was then transferred into the second vial and the first vial was washed with 1,4-dioxane (1 mL). The mixture was allowed to stir at room temperature for 0.50 hours after that the solution was filtered in the Glovebox. The resulting mixture was stirred at rt for 16 h. The reaction was afterwards analyzed by GC-FID.

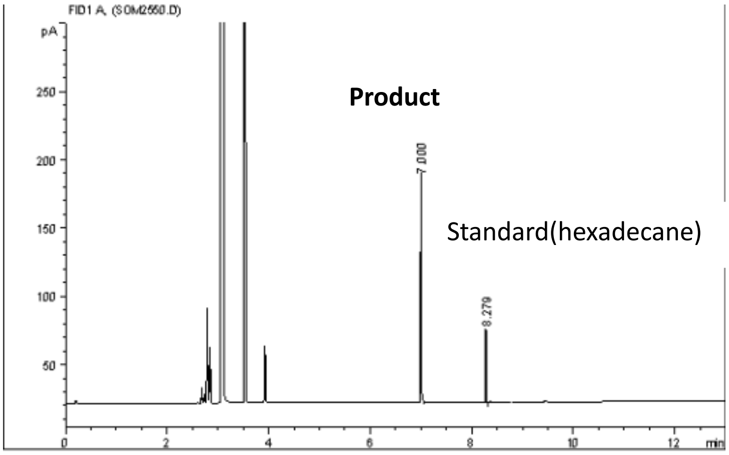


```
=====================================================================
                     Area Percent Report
=====================================================================

Sorted By             :      Signal
Multiplier            :      1.0000
Dilution              :      1.0000
Use Multiplier & Dilution Factor with ISTDs

Signal 1: FID1 A,

Peak RetTime Type  Width     Area      Height     Area
  #   [min]        [min]   [pA*s]      [pA]        %
----|-------|----|-------|----------|----------|--------|
   1   7.000 BB    0.0154  161.69438  164.96953 76.25845
   2   8.279 BB    0.0151   50.34032   52.82225 23.74155

Totals :                   212.03470  217.79178
```

Figure S13: GC-FID chromatogram of the reaction mixture from the mercury droplet experiment. The peak at 7.000 ppm corresponds to the product, and the peak at 8.279 ppm corresponds to the internal standard (hexadecane).

6.UV-VIS Solubility Experiments

6.1.Solubility determination for [Pd(1-MeNAP)TFA]₂

A solution of [Pd(1-MeNAP)TFA]$_2$ (30.0 mg) in THF (1.5 mL) was generated (20.0 mg/mL, 27.73 mM). Serial dilutions were performed to make four additional solutions of [Pd(1-MeNAP)TFA]$_2$ in THF: 0.0691 mM (0.0499 mg/mL), 0.0346 mM (0.0249 mg/mL), 0.0173 mM (0.0125 mg/mL), 0.00867 mM (0.00625 mg/mL) solutions in THF. UV/Vis absorbance spectra were recorded from 200-800 nm on a Implen NanoPhotometer P300 (**Figure S11**). These data were used to create calibration curves and to determine the molar extinction coefficients for the absorbances at 250 nm (**Figure S12**).

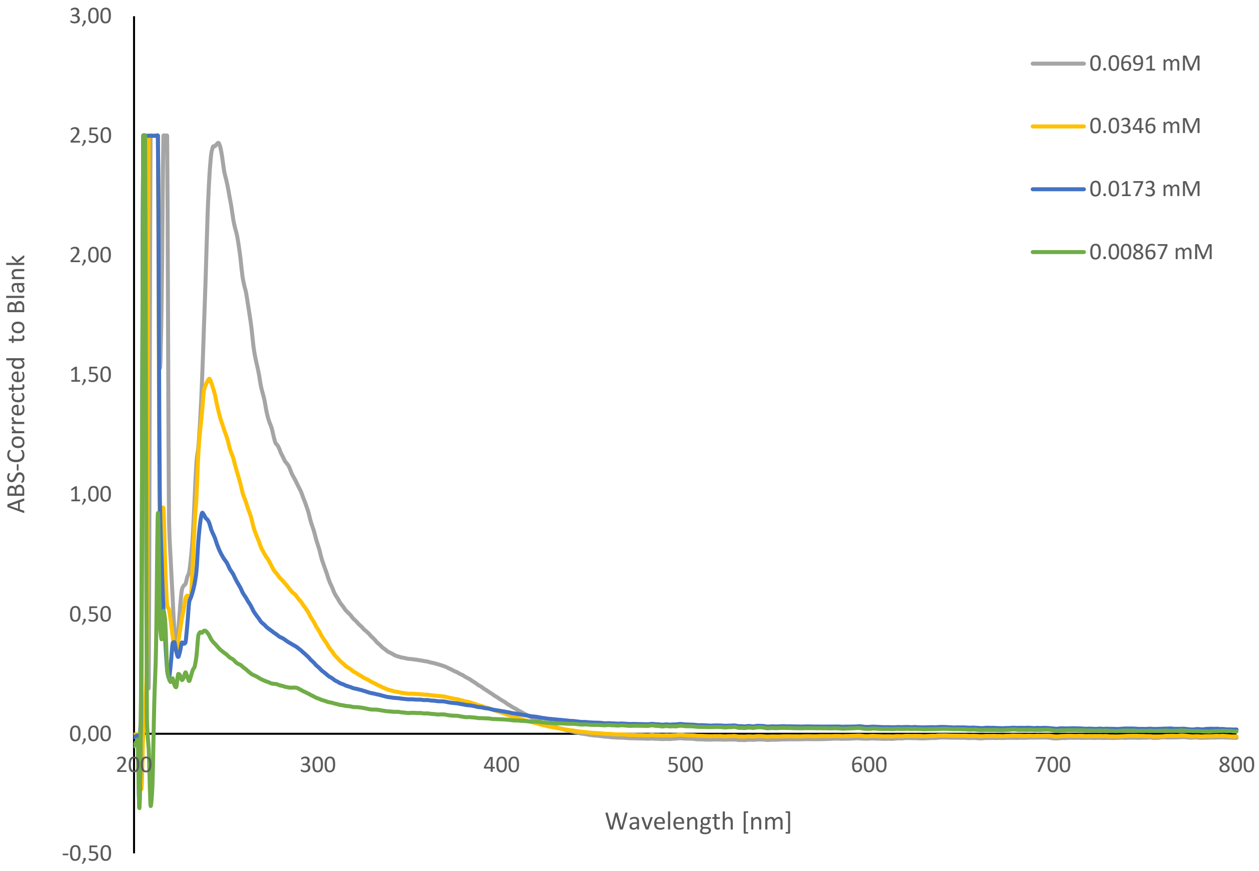

Figure S14: UV-Vis absorbance spectra for at [Pd(1-MeNAP)TFA]$_2$ at 0.07 mM – 0.009 mM concentration (THF solution).

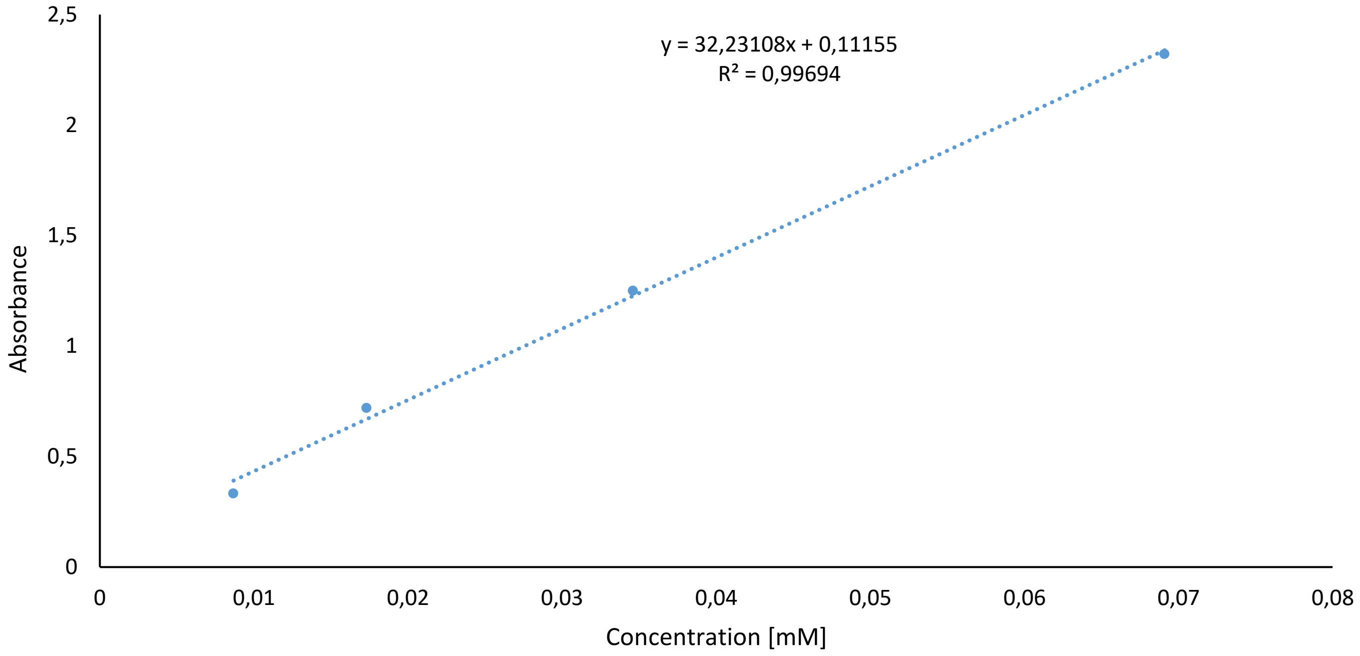

Figure S15: Concentration/absorbance calibration curve for peak at 250 nm.

To determine the solubility of [Pd(1-MeNAP)TFA]$_2$ in a variety of organic solvents, [Pd(1-MeNAP)TFA]$_2$ (20.0 mg) was dissolved/ suspended in 1.0 mL of each solvent. After thoroughly mixing at room temperature, a quantitative aliquot (30 µL) of the supernatant was diluted in a known amount of THF (12 mL). and subsequently analyzed by UV/Vis absorbance spectroscopy. The solubility (mg/mL) was calculated using the calibration curve generated for the peak at 250 nm using the following equation.

$$Solubility\ (mg/mL) = \left(\frac{A - b}{m}\right) \times \frac{V_{diluted}}{V_{aliquot}}$$

Symbol	Meaning
A	Measured absorbance of the diluted solution
b	Y-intercept of the calibration curv
m	Slope of the calibration curve
$V_{diluted}$	Total volume of the diluted solution (mL)
$V_{aliquot}$	Volume of the aliquot taken from the original sample (mL)

Using this relationship, the solubility in mg/mL can be determined for each solvent after correcting for the applied dilution factor.

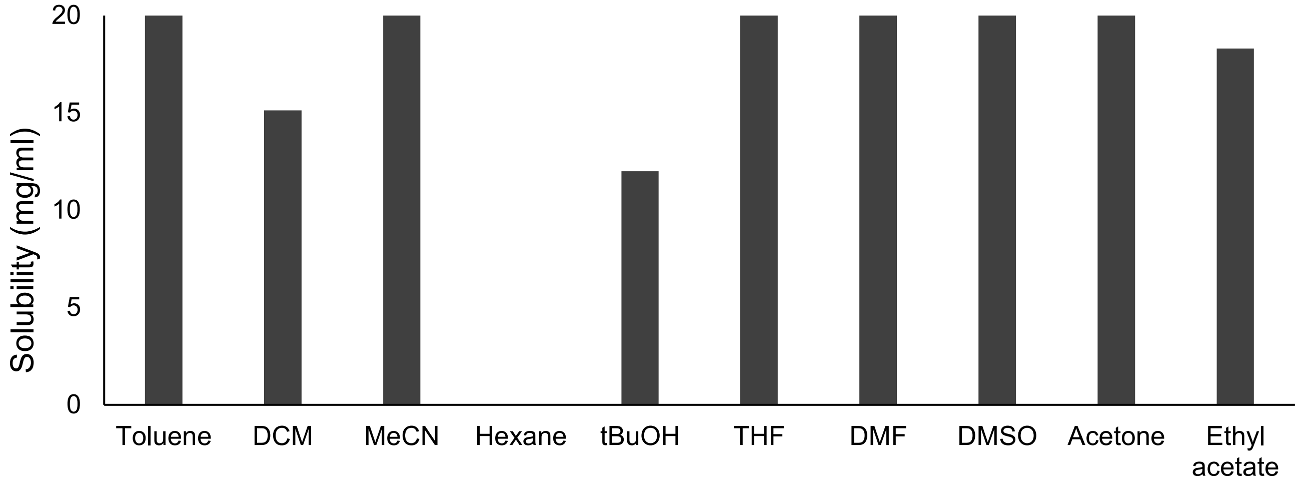

Figure S16: Solubility of $[Pd(1\text{-}MeNAP)TFA]_2$ determined by UV/Vis absorbance spectroscopy

7.Calculation Studies

Density functional theory (DFT) calculations were carried out using the ORCA program package (version 6.0).[3] Geometry optimizations were performed at the B3LYP/def2-SVP level of theory, including D3BJ dispersion corrections and a CPCM solvent model (THF). Single-point energy calculations on the optimized geometries were subsequently performed using the def2-TZVP basis set.For the energy diagram, the Gibbs free energy in solution (ΔGsolv) was used, calculated according to the following formula ($\Delta G^{TZVP} = \Delta E^{TZVP} + (\Delta G^{SVP} - \Delta E^{SVP})$).The corresponding XYZ coordinate files are provided in a ZIP archive. The energy diagram was generated using the ChemDraw Energy Diagram Plotter.[4]

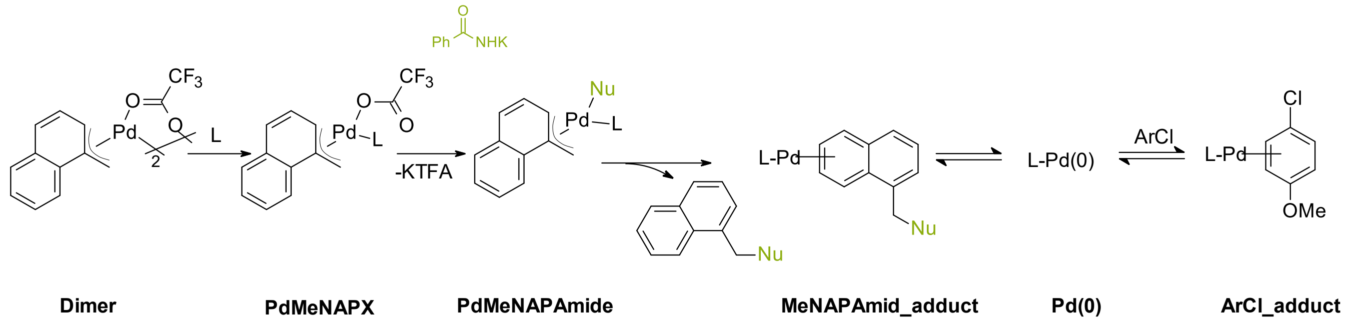

Figure S17: Representative reaction equation for [Pd(1-MeNAP)TFA] with L = tBuBrettPhos to visualize the different calculated step. The calculated intermediates were the same for the other Pd sources studied, differing only by exchange of the respective moiety from MeNAP to allyl, cinnamyl, or trifluoroacetate to bromide

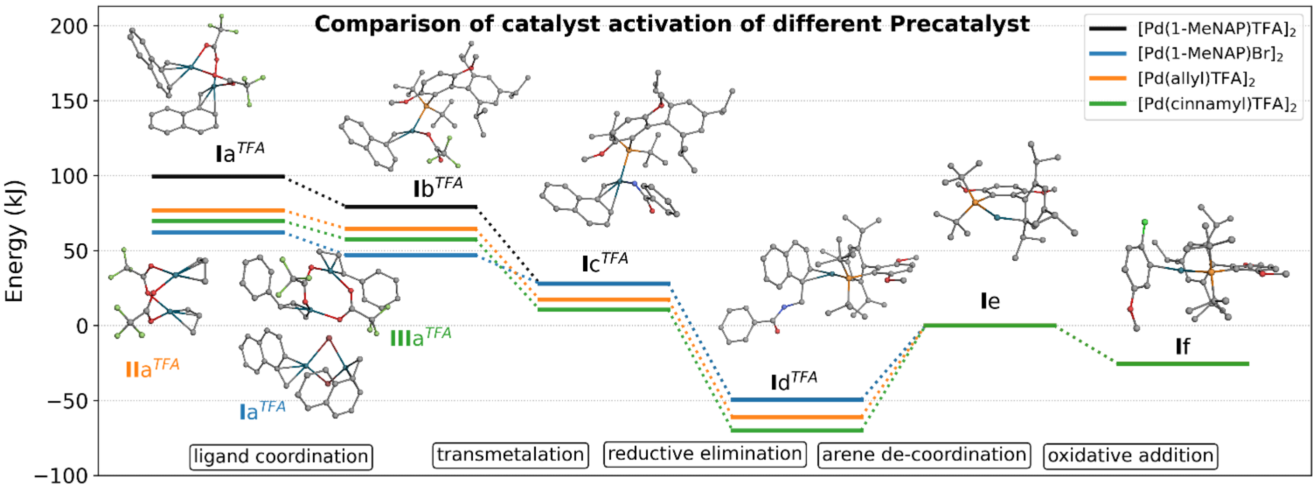

Figure S18: Energy diagram of $[Pd(1\text{-}MeNAP)TFA]_2$ (black) compared with related palladium catalysts: $[Pd(1\text{-}MeNAP)Br]_2$ (Blue), $[Pd(allyl)TFA]_2$ (orange), and $[Pd(cinnamyl)TFA]_2$ (green). The Pd(0) species was set to 0 kcal ·mol− 1 for comparative purposes. All optimized structures are provided in the attached ZIP file (.xyz format).

8.Synthesis of Palladium Catalysts

8.1.Synthesis of [Pd(1-MeNAP)TFA]$_2$

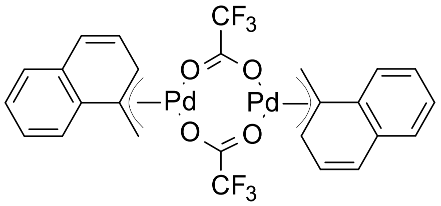

A brown crimp cap vial equipped with a Teflon coated magnetic stirring bar was charged with [Pd(1-MeNAP)Br]$_2$ (1310 mg, 2.0 mmol, 1 equiv.). The vial was entered inside a nitrogen filled glovebox, where silver trifluoroacetate (884 mg, 4 mmol, 2 equiv.) and dry and degassed THF (24 mL) was added. The mixture was allowed to stir at room temperature for 1 hour inside the glovebox, avoiding exposure to light. The reaction solution was microfiltered into a 100 mL flask using a syringe filter the flask was transferred out of the glovebox and the solvent was removed under reduced pressure. The remaining solid was washed with pentane (2 x 40 mL), the supernatant was removed by decantation under air. The solid was dried under reduced pressure for 16 hours to afforded the the title compound as an yellowish/greenish solid (1403 mg, 1.95 mmol, 97%).

^{1}H NMR (300 MHz, DMSO-d^6): δ = 8.35 (d, J=8.3 Hz, 2 H) 7.90 - 7.99 (m, 4 H) 7.54 - 7.69 (m, 4 H) 7.33 (dd, J=8.2, 6.9 Hz, 2 H) 7.21 (d, J=6.3Hz, 2 H) 3.81 (s, 4 H) ppm.

^{13}C NMR (75 MHz, DMSO-d^6): δ = 159.5 (q, J=33.8 Hz), 137.6, 133.8, 130.9, 128.1, 126.8, 126.7,126.5, 125.8, 125.1, 118.9, 116.3 (q, J=291.7 Hz), 30.5 ppm.

^{19}F NMR (76 MHz, DMSO-d$_6$, $C_6H_4F_2$): δ = -75.4 ppm

Elemental analysis: calcd (%) for $C_{26}H_{18}F_6O_4Pd_2$: C, 43.30, H, 2.52; N, 0.00; found: C, 42.98 H, 2.51 N,0.00.

8.2.Synthesis of [Pd(1-MeNAP)OTf]₂

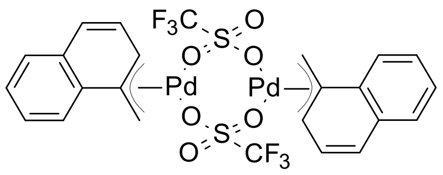

A brown crimp cap vial equipped with a Teflon coated magnetic stirring bar was charged with $[Pd(1\text{-}MeNAP)Br]_2$ (81.9 mg, 0.125 mmol, 1 equiv.). The vial was entered inside a nitrogen filled glovebox, where silver trifluoromethanesulfonate (65.5 mg, 0.25 mmol, 2 equiv.) and dry and degassed THF (3 mL) was added. The mixture was allowed to stir at room temperature for 1 hour inside the glovebox, avoiding exposure to light. The reaction solution was microfiltered into a 20 mL vial using a syringe filter the flask was transferred out of the glovebox and the solvent was removed under reduced pressure. The remaining solid was washed with pentane (2 x 40 mL), the supernatant was removed by decantation inside a nitrogen filled glovebox. The solid was dried under reduced pressure for 16 hours to afford the title compound as an yellowish/greenish solid (99.8 mg, 0.125 mmol, 99%). The solid was stored inside a nitrogen-filled glovebox, as it rapidly decomposes when exposed to air. Vapor diffusion of pentane into a saturated solution of the compound in dichloromethane under inert atmosphere led to crystals (red plates) that were studied by X-ray diffraction analysis (Cu Kα radiation, 100 K) leading to the establishment of connectivity in the solid state.

^{1}H NMR (400 MHz, THF-d^8): δ = 8.06 (d, *J*=7.9 Hz, 2 H), 7.97 (d, *J*=9.0 Hz, 2 H), 7.82 (dd, *J*=7.8, 1.4 Hz, 2 H), 7.56 - 7.71 (m, 4 H), 7.23 (dd, *J*=9.0, 6.1 Hz, 2 H), 5.53 (d, *J*=6.1 Hz, 2 H), 4.90 (br. s., 2 H), 2.85 (br. s., 2 H) ppm.

^{13}C NMR (101 MHz, THF-d^8): δ = 135.6, 132.8, 131.8, 131.1, 129.6, 129.2, 125.8, 118.3, 75.0, 68.4, 44.2, 26.5 ppm.

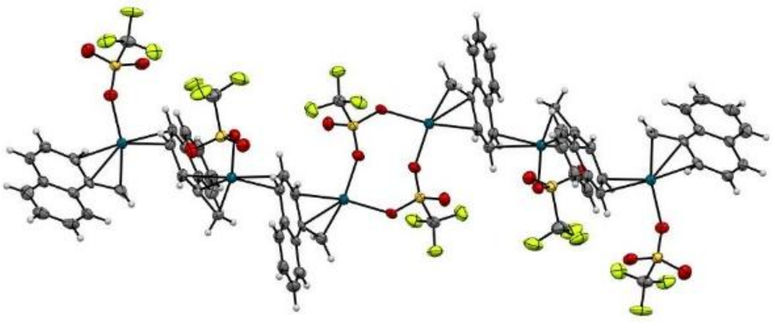

Figure 19: The X-ray molecular structure of $[Pd(1\text{-}MeNAP)OTf]_2$ is presented, showing thermal displacement ellipsoids at the 50% probability level. The established connectivity confirms the absence of stabilizing solvent molecules in the solid state and shows the dimeric structure which includes two trimeric subunits. Further structural/crystallographic information such as bond lengths and bond angles are not presented due to the low quality of the crystal, heavy atom (Pd) effects in combination with Cu Kα radiation, and the unstable nature of the complex.

8.3.Synthesis of [Pd(1-MeNAP)OTs]$_2$

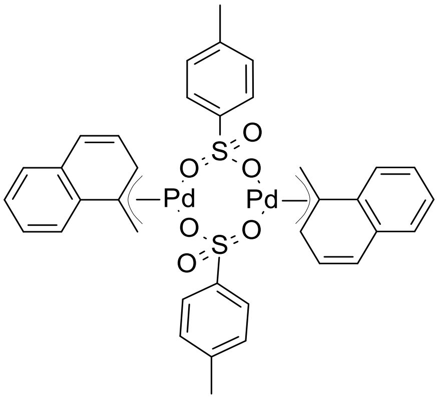

A brown crimp cap vial equipped with a Teflon coated magnetic stirring bar was charged with [Pd(1-MeNAP)Br]$_2$ (81.9 mg, 0.125 mmol, 1 equiv.). The vial was entered inside a nitrogen filled glovebox, where silver p-toluenesulfonate (69.8 mg, 0.25 mmol, 2 equiv.) and dry and degassed THF (3 mL) was added. The mixture was allowed to stir at room temperature for 1 hour inside the glovebox, avoiding exposure to light. The reaction solution was microfiltered into a 20 mL vial using a syringe filter the flask was transferred out of the glovebox and the solvent was removed under reduced pressure. The remaining solid was washed with pentane (2 x 40 mL), the supernatant was removed by decantation under air. The solid was dried under reduced pressure for 16 hours to afford the title compound as an yellowish/greenish solid (100.0 mg, 0.119 mmol, 96%). The solid is moisture-sensitive; therefore, it was stored in a nitrogen-filled glovebox. Attempts to grow single crystals of the complex were either unsuccessful or led to structures incorporating water molecules (*vide infra*).

^{1}H NMR (300 MHz, THF-d^8): δ = 8.11 (d, J=7.9 Hz, 2 H), 7.92 (d, J=9.0 Hz, 2 H), 7.80 - 7.87 (m, 2 H), 7.55 - 7.74 (m, 8H), 7.25 (dd, J=9.0, 6.1 Hz, 2 H), 7.07 (d, J=7.9 Hz, 4H), 5.31 (d, J=5.9 Hz, 2 H), 4.74 - 5.11 (br. s, 2 H), 2.58 - 2.91 (br. s, 2 H), 2.31 (s, 6 H) ppm.

^{13}C NMR (101 MHz, THF-d^8): δ = 140.7, 135.6, 132.6, 131.4, 130.7, 129.4, 129.3, 129.2, 129.1, 127.5, 125.9, 117.6, 68.4, 43.1, 26.5, 21.5 ppm.

Vapor diffusion of hexane into a solution of the compound in acetone at 5 degrees celsius led to single crystals (yellow needles) of suitable quality for X-ray diffraction studies. However, the determined molecular structures includes two water molecules.

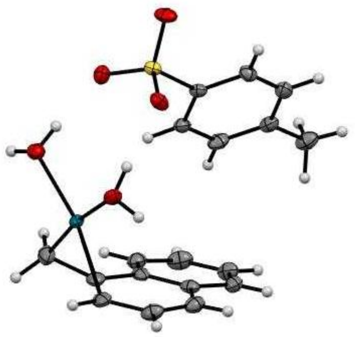

Figure S20: The X-ray molecular structure of [Pd(1-MeNAP)(H_2O)$_2$] [OTs] is presented, showing thermal displacement ellipsoids at the 50% probability level (CCDC: 2526680).

9.Synthesis of Palladium Precatalysts

A crimp cap vial equipped with a Teflon coated magnetic stirring bar was charged with [Pd(1-MeNAP)TFA]$_2$ (108 mg, 0.15 mmol, 1.0 equiv.) and Ligand (0.3 mmol, 2.0 equiv.). The vial was entered inside a nitrogen filled glovebox, where dry and degassed THF (3 mL) was added. The mixture was allowed to stir at room temperature for 1 hour inside the glovebox. The vial was transferred out of the glovebox and the solvent was removed under reduced pressure. The remaining solid was washed with pentane (2 x 10 mL), the supernatant was removed by decantation. The solid was dried under reduced pressure for 16 hours to afford the Precatalyts.

9.1.Synthesis of [Pd(1-MeNAP)(tBuBrettPhos)TFA]

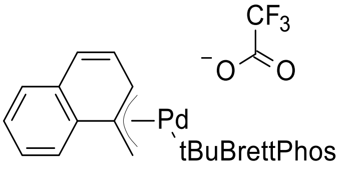

Following general procedure for the synthesis of precatalyst and starting from *t*BuBrettPhos the title compound was obtained as a yellow/greenish solid (102.5 mg, 0.12 mmol, 81%).

^{1}H NMR (400 MHz, DCM-d^2): δ = 8.19 (dd, J=8.4, 4.3 Hz, 1 H), 7.92 - 7.99 (m, 1 H), 7.82 - 7.89 (m, 1 H), 7.65 - 7.74 (m, 3 H), 7.22 (br. s., 1 H), 7.03 (dd, J=8.9, 2.9 Hz, 2 H), 6.91 (d, J=8.9 Hz, 2 H), 6.56 - 6.64 (m, 1 H), 6.31 (br. s., 1 H), 5.71 (t, J=6.4 Hz, 1 H), 4.93 (br. s., 1 H), 3.86 (s, 4 H), 3.47 (dt, J=13.7, 6.9 Hz, 1 H), 3.27 (s, 3 H), 2.87 (br. s., 1 H), 2.67 (br. s., 1 H), 2.42 (d, J=7.9 Hz, 1 H), 1.15 - 1.85 (m, 27 H), 0.83 - 1.00 (m, 4 H), 0.40 (br. s., 3 H), -0.11 (br. s., 3 H) ppm.

^{13}C NMR (101 MHz, DCM-d^2): 157.3, 155.3, 154.9, 154.9, 152.2, 152.0, 149.7, 137.5, 137.3, 135.8, 135.8, 135.2, 135.1, 130.1, 129.9, 129.8, 129.5, 127.9, 127.9, 126.4, 126.1, 124.3, 124.1, 123.3, 115.6, 115.5, 115.4, 115.3, 112.7, 112.7, 112.5, 112.5, 111.5, 111.4, 68.3, 54.5, 54.3, 53.7, 53.5, 43.0, 42.9, 41.9, 34.7, 34.1, 32.8, 32.3, 31.4, 26.1, 25.0, 24.5, 23.7, 23.3, 22.9, 14.4 ppm.

^{19}F NMR (376 MHz, DCM-d^2, $C_6H_4F_2$): δ = -75.7 ppm.

^{31}P NMR (162 MHz, DCM-d^2): δ = 94.3 ppm.

IR (ATR): ṽ = 3046 (m), 2959 (m), 2867 (m), 1689 (vs), 1599 (w), 1458 (m), 1423 (m), 1298 (s), 1193 (s), 1148 (s), 1049 (s). 781 (s), 713 (s), 476 (w) cm^{-1}

Elemental analysis: calcd (%) for $C_{44}H_{58}F_3O_4PPd$: C, 62.52, H, 6.92; N, 0.00; found: C, 62.09 H, 6,30 N,0.19. (0.62% off for Hydrogen)

9.2.Synthesis of [Pd(1-MeNAP)(RuPhos)TFA]

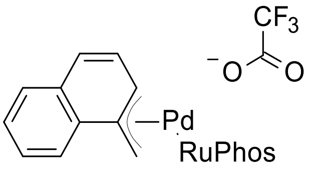

Following general procedure for the synthesis of precatalyst and starting from RuPhos the title compound was obtained as a yellow/greenish solid (110.8 mg, 0.13 mmol, 89%).

^{1}H NMR (400 MHz, DCM-d^2): 8.44 (dd, *J*=7.9, 3.8 Hz, 1 H), 7.90 - 8.00 (m, 1 H), 7.75 - 7.84 (m, 1 H), 7.57 - 7.70 (m, 4 H), 7.43 (br. s., 2 H), 6.88 (t, *J*=7.3 Hz, 1 H), 6.64 - 6.74 (m, 1 H), 6.04 (d, *J*=7.0 Hz, 2 H), 5.91 (t, *J*=6.1 Hz, 1 H), 4.14 (br. s., 2 H), 3.62 (br. s., 2 H), 2.32 - 2.51 (m, 2 H), 2.23 (br. s., 1 H), 1.96 (br. s., 2 H), 1.75 - 1.91 (m, 8 H), 1.16 - 1.49 (m, 11 H), 0.83 (d, *J*=6.0 Hz, 6 H), 0.69 (br. s., 5 H) ppm.

^{13}C NMR (101 MHz, DCM-d^2): δ = 159.3, 145.7, 135.4, 135.1, 135.1, 135.0, 133.0, 132.9, 132.5, 132.2, 131.8, 131.7, 131.6, 130.6, 130.0, 129.8, 129.0, 128.2, 128.1, 127.7, 127.6, 123.5, 115.2, 104.0, 71.8, 54.2, 53.9, 37.5, 37.4, 37.4, 37.1, 30.5, 30.4, 30.0, 27.3, 27.2, 27.0, 26.6, 21.9, 21.1, 14.4 ppm.

^{19}F NMR (376 MHz, DCM-d^2, $C_6H_4F_2$): δ = -75.7 ppm.

^{31}P NMR (162 MHz, DCM-d^2): δ = 60.36 ppm.

IR (ATR): ṽ = 3052 (w), 2962 (m), 2853 (w), 1685 (vs), 1461 (w), 1446 (w), 1194 (vs), 1124 (s), 837 (s), 795 (s), 767 (s), 723 (s), 527 (w), 509 (w) cm^{-1}

Elemental analysis: calcd (%) for $C_{45}H_{52}F_3O_4PPd$: C, 62.43, H, 6.34; N, 0.00; found: C, 61.85 H, 5.65 N,0.14. (0.58% off for Carbon, 0.69% off for Hydrogen).

10. Synthesis **Procedures**

10.1.Synthesis of 4-Methoxybenzanilide (3a) [CAS 7472-54-0]

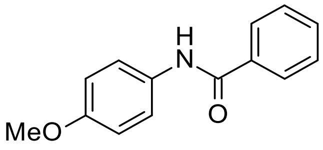

Following general procedure (GP A) and starting from 4-Chloranisol the title compound was obtained as a white solid (108,3 mg, 0.477 mmol, 95%). 0–30% ethyl acetate in cyclohexane.

The NMR data are in agreement with those reported in literature.[5]

^{1}H NMR (300 MHz, $CDCl_3$): δ = 7.82 - 7.93 (m, 2 H), 7.74 (br. s., 1 H), 7.44 - 7.61 (m, 5 H), 6.88 - 6.97 (m, 2 H), 3.83 (s, 3 H) ppm.

^{13}C NMR (75 MHz, , $CDCl_3$): δ = 157.0, 135.5, 132.1, 131.4, 129.2, 127.4, 122.5, 114.7, 77.8, 55.9 ppm.

IR (ATR): ṽ = 3328 (w), 2963 (w), 2837 (w), 2228 (w), 2053 (w), 1645 (m), 1408 (m), 1324 (m), 715 ,, 671 (m), 526 (m), 494 (w), 411 (w) cm^{-1}

GC-HRMS (EI-QTOF): [M]+calcd. 222.0795 for $C_{14}H_{10}N_2O$; found: 222.0793.

*10.2.Synthesis of N-(3-methylbenzothiophen-5-yl)benzamide (**3b**) [CAS 10278-46-3]*

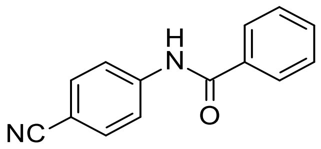

Following general procedure (GP A) and starting from 4-Chlorobenzonitrile the title compound was obtained as a white solid (99.0 mg, 0.445 mmol, 89%). 0–30% ethyl acetate in cyclohexane.

The NMR data are in agreement with those reported in literature.[6]

^{1}H NMR (300 MHz, $CDCl_3$): δ = 7.96 (m, 1 H), 7.86 - 7.92 (m, 2 H), 7.78 - 7.84 (m, 2 H), 7.66 - 7.72 (m, 2 H), 7.49 - 7.65 (m, 3 H) ppm.

^{13}C NMR (75 MHz, , $CDCl_3$): δ = 165.9, 142.0, 134.1, 133.3, 132.5, 129.0, 127.1, 119.9, 118.8, 107.4 ppm.

IR (ATR): ṽ = 3351 (w), 2226 (w), 1659 (m), 1590 (m), 1505 (m), 1407 (m), 1318 (m), 1246 (w), 1176 (w), 831 (w), 716 (w), 545 (w) cm^{-1}

GC-HRMS (EI-QTOF): [M]+calcd. 222.0795 for $C_{14}H_{10}N_2O$; found: 222.0793

*10.3.Synthesis of N-[4-(trifluoromethyl)phenyl]benzamide (**3c**) [CAS 350-98-1]*

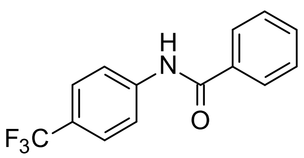

Following general procedure (GP A) and starting from 4-Chlorobenzotrifluoride the title compound was obtained as a white solid (124.3 mg, 0.467 mmol, 94%). 0–20% ethyl acetate in cyclohexane.

The NMR data are in agreement with those reported in literature.[7]

^{1}H NMR (300 MHz, $CDCl_3$): δ = 10.58 (s, 1 H), 7.93 - 8.08 (m, 4 H), 7.74 (d, J=0.5 Hz, 2 H), 7.50 - 7.65 (m, 3 H)ppm.

^{13}C NMR (101 MHz, , DMSO-d^6): 166.1, 142.8, 134.5, 131.9, 128.5, 127.8, 125.9 (q) 123.8, 123.4, 120.1 ppm.

^{19}F NMR (76 MHz, DMSO-d^6, $C_6H_4F_2$): -62.2 ppm.

IR (ATR): ṽ = 3289 (w), 2971 (m), 2359 (m), 1654 (m), 1598 (m), 1513 (m), 1403 (m), 1070 (m), 775 (m), 592 (m) cm^{-1}.

GC-HRMS (EI-QTOF): $[M]^+$calcd. 265.0715 for $C_{14}H_{10}F_3NO$; found: 265.0716.

*10.4.Synthesis of N-(4-trimethylsilylphenyl)benzamide (**3d**)*

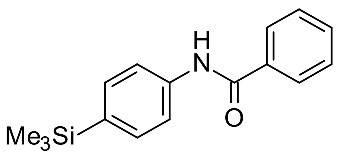

Following general procedure (GP A) and starting from (4-Chlorophenyl)(trimethyl)silane and Benzamide the title compound was obtained as a white solid (115.6 mg, 0.429 mmol, 86%). 0–20% ethyl acetate in cyclohexane.

^{1}H NMR (300 MHz, $CDCl_3$): δ = 7.85 - 7.93 (m, 2 H), 7.76 - 7.83 (m, 1 H), 7.62 - 7.69 (m, 2 H), 7.45 - 7.58 (m, 5 H), 0.24 - 0.33 (m, 9 H) ppm.

^{13}C NMR (75 MHz, $CDCl_3$): δ = 165.7, 138.4, 136.4, 135.0, 134.2, 131.8, 128.7, 127.0, 119.4, -1.1 ppm.

IR (ATR): ṽ = 3309 (m), 2954 (m), 2896 (w), 1650 (s), 1576 (s), 1505 (s), 1244 (s), 1076 (w), 816 (s), 522 (m) cm^{-1}

GC-HRMS (EI-QTOF): [M]+calcd 269.1236 for $C_{16}H_{19}NOSi$; found: 269.1238.

*10.5.Synthesis of N-(4-formylphenyl)benzamide (**3e**) [CAS 65854-93-5]*

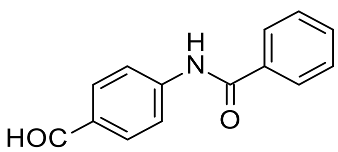

Following general procedure (GP A) and starting from 4-Chlorbenzaldehyd and Benzamide the title compound was obtained as a white solid (100.7 mg, 0.447 mmol, 89%). 0–40% ethyl acetate in cyclohexane.

The NMR data are in agreement with those reported in literature.[8]

^{1}H NMR (300 MHz, $CDCl_3$): δ = 9.97 (s, 1 H), 8.05 (br s, 1 H), 7.83 - 7.95 (m, 6 H), 7.49 - 7.65 (m, 3 H) ppm.

^{13}C NMR (75 MHz, $CDCl_3$): δ = 191.1, 166.0, 143.5, 134.3, 132.5, 132.4, 131.2, 129.0, 127.1, 119.7, 116.0 ppm.

IR (ATR): ṽ = 3334 (m), 2969 (w), 2831 (w), 1692 (s), 1655 (s), 1518 (s), 1315 (m), 1115 (s), 825 (m), 510 (m) cm^{-1}

GC-HRMS (EI-QTOF): [M]+calcd 225.0790 for $C_{14}H_{11}NO_2$; found: 225.0790.

*10.6.Synthesis of N-[4-(4,4,5,5-tetramethyl-1,3,2-dioxaborolan-2-yl)phenyl]benzamide (**3f**) [CAS 935660-75-6]*

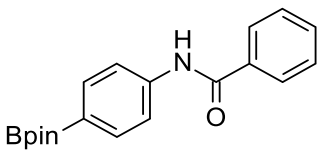

Following general procedure (GP A) and starting from 2-(4-Chlorophenyl)-4,4,5,5-tetramethyl-1,3,2-dioxaborolane and Benzamide the title compound was obtained as a white solid (145.8 mg, 0.451 mmol, 90%). 0–20% ethyl acetate in cyclohexane.

The NMR data are in agreement with those reported in literature.[9]

^{1}H NMR (300 MHz, $CDCl_3$): δ = 7.81 - 7.92 (m, 5 H), 7.68 (d, J=8.5 Hz, 2 H), 7.46 - 7.57 (m, 3 H), 1.36 (s, 12 H) ppm.

^{13}C NMR (75 MHz, $CDCl_3$): 165.6, 140.6, 135.9, 131.9, 129.1, 128.8, 127.0, 120.1, 118.8, 83.8 24.9 (s) ppm.

IR (ATR): ṽ = 3310 (m), 2980 (m), 1649 (s), 1650 (s), 1515 (s), 1399 (m), 1360 (s), 1320 (m), 828 (m), 641 (s) cm^{-1}

GC-HRMS (EI-QTOF): [M]+calcd 322.1729 for $C_{19}H_{22}BNO_3$; found: 322.1727.

*10.7.Synthesis of N-(3,5-Dimethoxyphenyl)benzamide (**3g**) [CAS 94088-74-1]*

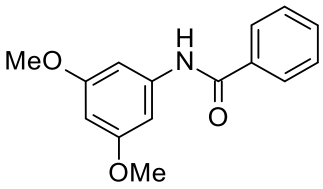

Following general procedure (GP A) and starting from 1-Chloro-3,5-dimethoxybenzene and Benzamide the title compound was obtained as a white solid (121.5 mg, 0.472 mmol, 94%). 0–20% ethyl acetate in cyclohexane.

The NMR data are in agreement with those reported in literature.[10]

^{1}H NMR (300 MHz, $CDCl_3$): δ = 7.80 - 7.89 (m, 2 H), 7.45 - 7.60 (m, 3 H), 6.92 (d, *J*=2.2 Hz, 2 H), 6.29 (t, *J*=2.2 Hz, 1 H), 3.81 (s, 6 H) ppm.

^{13}C NMR (75 MHz, $CDCl_3$): δ = 165.7, 161.1, 139.7, 134.9, 131.9, 128.8, 126.9, 98.3, 97.1, 55.4 ppm.

IR (ATR): ṽ = 3244 (m), 3140 (w), 3071 (w), 1651 (s), 1508 (s), 1597 (m), 1451 (m), 1206 (s), 1053 (m), 707 (m) cm^{-1}

GC-HRMS (EI-QTOF): [M]+calcd 257.1052 for $C_{15}H_{15}NO_3$; found: 257.1051.

*10.8.Synthesis of N-(3-pyridyl)benzamide (**3h**) [CAS 5221-40-9]*

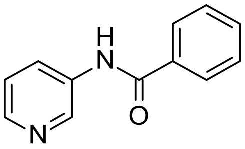

Following general procedure (GP A) and starting from 3-Chlorpyridine the title compound was obtained as a white solid (92.0 mg, 0.464 mmol, 93%). 0–100% ethyl acetate in cyclohexane.

The NMR data are in agreement with those reported in literature.[5]

^{1}H NMR (400 MHz, $CDCl_3$): δ = 8.95 (m, 1 H), 8.76 (m., 1 H), 8.58 (d, *J*=8.3 Hz, 1 H), 8.36 (d, *J*=4.4 Hz, 1 H), 7.99 (d, *J*=7.3 Hz, 2 H), 7.39 - 7.62 (m, 5 H), 4.26 (br. s., 1 H) ppm.

^{13}C NMR (101 MHz, , $CDCl_3$): δ = 166.4, 143.0, 139.9, 136.1, 133.8, 132.4, 132.0, 129.3, 128.5, 127.4, 127.3, 124.4 ppm.

IR (ATR): ṽ = 3366 (w), 3173 (m), 3063 (m), 1679 (m), 1579 (m), 1472 (m), 1322 (m), 925 (m), 809 (m), 685 (m) cm^{-1}.

GC-HRMS (EI-QTOF): $[M]^+$calcd. 198.0793 for $C_{12}H_{10}N_2O$; found: 198.0793.

*10.9.Synthesis of N-(6-Methoxy-3-pyridazinyl)benzamide (**3i**) [CAS 39614-86-3]*

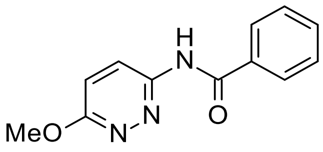

Following general procedure (GP A) and starting from 3-Chloro-6-methoxypyridazine and Benzamide the title compound was obtained as a white solid (101,7 mg, 0.444 mmol, 89%). 0–30% ethyl acetate in cyclohexane.

^{1}H NMR (300 MHz, *DMSO-d*$_6$): δ = 11.25 (s, 1 H), 8.20 - 8.30 (m, 1 H), 8.00 - 8.09 (m, 2 H), 7.47 - 7.67 (m, 3 H), 7.30 (d, *J*=9.5 Hz, 1 H), 4.02 (s, 3 H) ppm.

^{13}C NMR (75 MHz, *DMSO-d*$_6$): δ = 166.3, 162.6, 152.2, 133.5, 132.2, 128.4, 128.1, 124.7, 118.6, 54.4 ppm.

IR (ATR): ṽ = 3207 (m), 3093 (w), 3020 (w), 1663 (s), 1518 (s), 1461 (s), 1254 (w), 1013 (s), 709 (s), 582 (m) cm^{-1}

HPLC-HRMS (ESI): $[M+H]^+$ calcd. 230.0924 for $C_{12}H_{11}N_3O_2$; found: 230.0936.

*10.10.Synthesis of N-[4-(1-methylethenyl)phenyl]benzamide (**3j**) [CAS 2966931-58-6]*

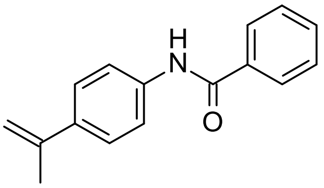

Following general procedure (GP A) and starting from 4-Chloro-α-methylstyrene and Benzamide the title compound was obtained as a white solid (89.1 mg, 0.376 mmol, 75%). 0–20% ethyl acetate in cyclohexane.

^{91}**H NMR** (300 MHz, $CDCl_3$): δ = 7.81 - 7.92 (m,3 H), 7.59 - 7.66 (m, 2 H), 7.46 - 7.58 (m, 5 H), 5.38 (s, 1 H), 5.08 (t, *J*=1.4 Hz, 1 H), 2.17 (s, 3 H) ppm.

^{13}C NMR (75 MHz, $CDCl_3$): δ = 165.6, 142.4, 137.4, 137.2, 135.0, 131.9, 128.8, 127.0, 126.2, 119.8, 111.9, 21.8 ppm.

IR (ATR): ṽ = 3300 (m), 3094 (w), 3050 (w), 1653 (s), 1589 (s), 1408 (s), 1076 (w), 879 (s), 831 (s), 714 (m) cm^{-1}

GC-HRMS (EI-QTOF): [M]+calcd 237.1154 for $C_{16}H_{15}NO$; found: 237.1158.

*10.11.Synthesis of N-3-Thienylbenzamide (**3k**) [CAS 79128-75-9]*

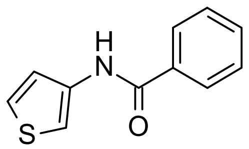

Following general procedure (GP A) and starting from 3-Chlorothiophene and Benzamide the title compound was obtained as a white solid (82,9 mg, 0.408 mmol, 92%). 0–50% ethyl acetate in cyclohexane.

The NMR data are in agreement with those reported in literature.[11]

^{1}H NMR (300 MHz, $CDCl_3$): δ = 8.14 (br. s., 1 H), 7.83 - 7.91 (m, 2 H), 7.71 - 7.76 (m, 3 H), 7.44 - 7.60 (m, 2 H), 7.14 (dd, *J*=5.2, 1.3 Hz, 1 H) ppm.

^{13}C NMR (75 MHz, $CDCl_3$): δ = 165.0, 135.6, 134.4, 131.9, 128.8, 126.9, 124.7, 121.2, 110.7 ppm.

IR (ATR): ṽ = 3331 (m), 3291 (w), 3144 (w), 1650 (s), 1567 (m), 1395 (m), 1174 (m), 829 (s), 769 (m), 614 (m) cm^{-1}

GC-HRMS (EI-QTOF): [M]+calcd 203.0405 for $C_{11}H_9NOS$; found: 203.0408.

*10.12.Synthesis of N-4-Thiazolylbenzamide (**3l**) [CAS 71168-41-7]*

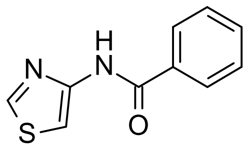

Following general procedure (GP A) and starting from 4-Bromothiazole and Benzamide the title compound was obtained as a white solid (91,6 mg, 0.448 mmol, 90%). 0–50% ethyl acetate in cyclohexane.
^{1}H NMR (300 MHz, $CDCl_3$): δ = 9.76 (br. s., 1 H), 8.37 (d, *J*=2.3 Hz, 1 H), 7.88 - 7.98 (m, 3 H), 7.46 - 7.63 (m, 3 H) ppm.
^{13}C NMR (75 MHz, $CDCl_3$): δ = 165.2, 150.7, 148.4, 134.0, 132.1, 128.8, 127.3, 101.8 ppm.

IR (ATR): ṽ = 3263 (m), 3061 (w), 2971 (w), 1664 (s), 1508 (s), 1403 (m), 1227 (m), 966 (s), 838 (m), 682 (m) cm^{-1}

GC-HRMS (EI-QTOF): [M]+calcd 204.0357 for $C_{10}H_8N_2OS$; found: 204.0361.

*10.13.Synthesis of N-(3-methylbenzothiophen-5-yl)benzamide (**3m**)*

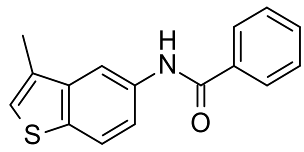

Following general procedure (GP A) and starting from 5-Chloro-3-methyl-benzo[b]thiophene the title compound was obtained as a white solid (108.0 mg, 0.404 mmol, 81%). 0–30% ethyl acetate in cyclohexane.

^{1}H NMR (300 MHz, $CDCl_3$): δ = 8.21 (d, J=2.0 Hz, 1 H), 7.90 - 8.02 (m, 3 H), 7.82 (d, J=8.6 Hz, 1 H), 7.44 - 7.62 (m, 4 H), 7.08 - 7.17 (m, 1 H), 2.46 (d, J=1.1 Hz, 3 H) ppm.

^{13}C NMR (75 MHz, , $CDCl_3$): δ = 140.3, 135.0, 134.5, 132.3, 131.8, 128.8, 127.0, 123.1, 122.8, 117.7, 113.3, 14.0 ppm.

IR (ATR): ṽ = 3296 (w), 2227 (w), 1341 (m), 1254 (m), 1154 (m), 1086 (m), 982 (m), 793 (w), 772 (w), 718 (m), 650 (w) cm^{-1}

GC-HRMS (EI-QTOF): [M]+calcd. 267.0719 for $C_{16}H_{13}NOS$; found: 267.0719.

*10.14.Synthesis of N-1,3-Benzodioxol-5-ylbenzamide (**3n**) [CAS 97631-47-5]*

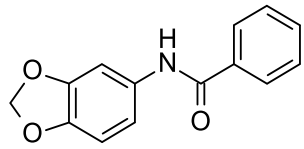

Following general procedure (GP A) and starting from 5-Chloro-1,3-benzodioxole the title compound was obtained as a white solid (97.0 mg, 0.402 mmol, 80%). 0–40% ethyl acetate in cyclohexane.

The NMR data are in agreement with those reported in literature.[12]

^{1}H NMR (400 MHz, $CDCl_3$): δ =7.86 (d, *J*=7.3 Hz, 2 H), 7.74 (br. s., 1 H), 7.45 - 7.59 (m, 3 H), 7.37 (s, 1 H), 6.88 - 6.93 (m, 1 H), 6.80 (d, *J*=8.3 Hz, 1H), 5.99 (s, 2 H) ppm.

^{13}C NMR (75 MHz, , $CDCl_3$): δ = 165.7, 147.8, 144.5, 134.8, 132.1, 131.7, 128.7, 127.0, 113.6, 108.1, 103.2, 101.3 ppm.

IR (ATR): ṽ = 3310 (w), 2227 (w), 1664 (m), 1344 (m), 1294 (m), 1279 (m), 936 (m), 922 (m), 784 (m), 688 (m), 614 (m), 458 (w), 433 (w) cm^{-1}

GC-HRMS (EI-QTOF): [M]+calcd. 241.0741 for $C_{14}H_{11}NO_3$; found: 241.0739.

*10.15.Synthesis of N-(4-Methoxyphenyl)-4-(trifluoromethyl)benzamide (**3o**) [CAS 447431-35-8]*

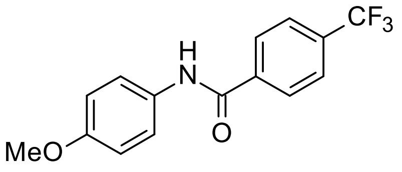

Following general procedure (GP A) and starting from 4-Chloranisol and 4-(Trifluoromethyl)benzamide the title compound was obtained as a white solid (108,3 mg, 0.477 mmol, 95%). 0–30% ethyl acetate in cyclohexane.

The NMR data are in agreement with those reported in literature.[13]

^{1}H NMR (300 MHz, *DMSO-d*$_6$): δ = 10.34 (s, 1 H), 8.14 (d, *J*=8.1 Hz, 2 H), 7.90 (d, *J*=8.2 Hz, 2 H), 7.64 - 7.71 (m, 2 H), 6.89 - 7.00 (m, 2 H), 3.75 (s, 3 H) ppm.

^{13}C NMR (75 MHz, *DMSO-d*$_6$): δ = 162.6, 145.0, 139.0 133.4, 119.5, 116.6, 110.4, 102.0 ppm.

^{19}F NMR (76 MHz, *DMSO-d*$_6$, $C_6H_4F_2$): δ = -63.1 ppm.

IR (ATR): ṽ = 3338 (m), 3028 (w), 2844 (w), 1648 (m), 1510 (m), 1125 (m), 1164 (m), 550 (w), 474 (w) cm^{-1}

GC-HRMS (EI-QTOF): [M]+calcd. 295.0820 for $C_{15}H_{12}F_3NO_2$; found: 295.0822.

*10.16.Synthesis of N-[4-(Trifluoromethyl)phenyl]-2-furancarboxamide (**3p**) [CAS 25617-46-3]*

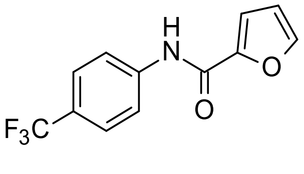

Following general procedure (GP A) and starting from 4-Chlorobenzotrifluoride and 2-Furamidethe title compound was obtained as a white solid (110,0 mg, 0.431 mmol, 86%). 0–30% ethyl acetate in cyclohexane.

The NMR data are in agreement with those reported in literature.[14]

^{1}H NMR (300 MHz, $CDCl_3$): δ = 8.21 (br. s, 1 H), 7.80 (d, *J*=8.6 Hz, 2 H), 7.63 (d, *J*=8.6 Hz, 2 H), 7.53 - 7.56 (m, 1 H), 7.29 (d, *J*=3.6 Hz, 1 H), 6.60 (dd, *J*=3.5, 1.7 Hz, 1 H) ppm.

^{13}C NMR (75 MHz, $CDCl_3$): δ = 156.1, 147.3, 144.5, 140.5, 126.4 (q), 125.9 (q), 119.5, 116.0 112.8 ppm.

^{19}F NMR (76 MHz, $CDCl_3$, $C_6H_4F_2$): -63.5 ppm.

IR (ATR): ṽ = 3661 (w), 2980 ,, 2885 (w), 2362 (w), 1663 (w), 1600 (w), 1471 (w), 832 (m), 751 (m), 592 (w) cm^{-1}

GC-HRMS (EI-QTOF): [M]+calcd. 255.0507 for $C_{12}H_8F_3NO_2$; found: 255.0511.

*10.17.*Synthesi**s** *of N-[4-(Trifluoromethyl)phenyl]-2-thiopheneacetamide (**3q**) [CAS 639810-25-6]*

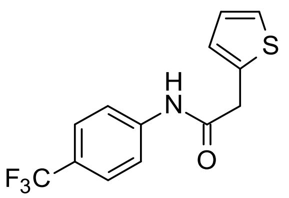

Following general procedure (GP A) and starting from 4-Chlorobenzotrifluoride and thiophene-2-acetamide title compound was obtained as a white solid (125.2 mg, 0.439 mmol, 88%). 0–30% ethyl acetate in cyclohexane.

The NMR data are in agreement with those reported in literature.[15]

^{1}H NMR (300 MHz, $CDCl_3$): δ = 7.52 - 7.61 (m, 4 H), 7.46 (br. s., 1 H), 7.34 (dd, *J*=4.8, 1.6 Hz, 1 H), 7.03 - 7.11 (m, 2 H), 3.98 (s, 2 H) ppm.

^{13}C NMR (75 MHz, $CDCl_3$): δ = 168.0, 140.4, 135.0, 128.0, 127.8, 126.4, 126.1 - 126.3 (q), 119.4, 38.6 ppm.

^{19}F NMR (76 MHz, $CDCl_3$, $C_6H_4F_2$): -63.2 ppm.

IR (ATR): ṽ = 3267 (w), 3199 (vw), 3131 (vw), 3065 (vw), 1661 (m), 1604 (m), 1541 (m),1408 (m), 1108 (m), 1068 (m), 1013 (w), 837 (m), 701 (m), 551 (w), 510 (w) cm^{-1}

GC-HRMS (EI-QTOF): [M]+calcd. 285.0435 for $C_{13}H_{10}F_3NOS$; found: 285.0439.

*10.18.*Synthesis *of N-[4-(Trifluoromethyl)phenyl]-2-pyridineacetamide (**3r**) [CAS 1790280-85-1]*

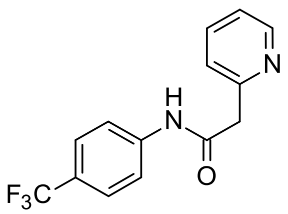

Following general procedure (GP A) and starting from 4-Chlorobenzotrifluoride and 2-(Pyridin-2-yl)acetamide title compound was obtained as a white solid (75.0 mg, 0.268 mmol, 54%). 0–80% ethyl acetate in cyclohexane.

The NMR data are in agreement with those reported in literature.[16]

^{1}H NMR (300 MHz, $CDCl_3$): δ = 10.40 (br. s, 1 H), 8.58 - 8.67 (m, 1 H), 7.64 - 7.76 (m, 3 H), 7.53 (d, *J*=8.5 Hz, 2 H), 7.21 - 7.36 (m, 2 H), 3.90 (s, 2 H) ppm.

^{13}C NMR (75 MHz, $CDCl_3$): δ = 167.5, 155.0, 148.8, 141.2, 137.7, 126.0 (q), 124.9 - 125.9 (m), 124.4, 122.5, 119.3, 45.5 ppm.

^{19}F NMR (76 MHz, $CDCl_3$, $C_6H_4F_2$): -62.1 ppm.

IR (ATR): ṽ = 3250 (vw), 2970 (vw), 2921 (vw), 1659 (m), 1605 (m), 1476 (m), 1265 (w), 998 (w), 968 (m), 791 (m), 657 (m), 422 (vw), 415 (vw) cm^{-1}

GC-HRMS (EI-QTOF): [M]+calcd. 280.0824 for C14H11F3N_2O; found: 280.0823.

*10.19.*Synthesis *of N-[4-(Trifluoromethyl)phenyl]cyclopropanecarboxamide (**3s**) [CAS 23845-06-9]*

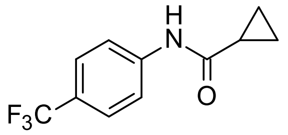

Following general procedure (GP A) and starting from 4-Chlorobenzotrifluoride and cyclopropanecarboxamide title compound was obtained as a white solid (94.3 mg, 0.411 mmol, 82%). 0–30% ethyl acetate in cyclohexane.

^{1}H NMR (300 MHz, DMSO-d_6): δ = 10.55 (s, 1 H), 7.79 (d, *J*=8.5 Hz, 2 H), 7.65 (d, *J*=8.7 Hz, 2 H), 1.80 (quin, *J*=6.2 Hz, 1 H), 0.83 (d, *J*=6.2 Hz, 4 H) ppm.

^{13}C NMR (75 MHz, DMSO-d_6): δ = 172.7, 141.9, 125.8 (q) 124.5 - 125.2 (m), 119.0, 39.1 - 40.3 (m), 15.2, 7.9 ppm.

^{19}F NMR (76 MHz, DMSO-d_6, $C_6H_4F_2$): -62.0 ppm.

IR (ATR): ṽ = 3301 (w), 2228 (w), 1661 (m), 1601 (m), 1528 (m), 1321 (m), 1156 (m), 1101 (vs), 1063 (vs), 837 (vs), 699 (m), 546 (w), 511 (w) cm^{-1}

GC-HRMS (EI-QTOF): [M]+calcd. 229.0715 for $C_{11}H_{10}F_3NO$; found: 229.0717.

*10.20.Synthesis of N-[4-(Trifluoromethoxy)phenyl]benzenecarboximidamide (**4a**) [CAS 1266333-17-8]*

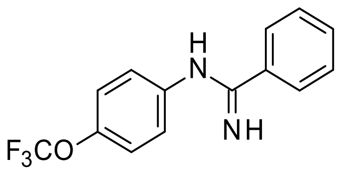

Following general procedure (GP C) and starting from 1-Chloro-4-(trifluoromethoxy)benzene the title compound was obtained as a off white solid (128.5 mg, 0.458 mmol, 91%). 0–30% (1% NEt_3 and 10% *i*PrOH in ethyl acetate) in (1% NEt_3 in cyclohexane).

The NMR data are in agreement with those reported in literature.[17]

^{1}H NMR (300 MHz, *DMSO-d*$_6$): δ = 7.97 (d, *J*=6.5 Hz, 2 H), 7.35 - 7.54 (m, 3 H), 7.27 (d, *J*=8.3 Hz, 2 H), 6.93 (d, *J*=7.8 Hz, 2 H), 6.45 (br. s., 2 H) ppm.

^{13}C NMR (75 MHz, *DMSO-d*$_6$): δ = 154.3, 150.1, 143.0, 135.7, 130.1, 128.0, 127.1, 123.0, 122.0, 118.6, ppm.

^{19}F NMR (76 MHz, DMSO-*d*$_6$, $C_6H_4F_2$): δ = - 57.7 ppm.

IR (ATR): ṽ = 3337 (w), 1918 (vw), 1698 (vw), 1448 (m), 1383 (m), 1267 (m), 1196 (m), 1097 (vs), 1097 (m), 869 (m), 693 (m) cm^{-1}

GC-HRMS (EI-QTOF): [M]+calcd. 280.0824 for $C_{14}H_{11}F_3N_2O$; found: 280.0822.

*10.21.Synthesis of N-(4-Acetylphenyl)benzenecarboximidamide (**4b**) [CAS 2366186-66-3]*

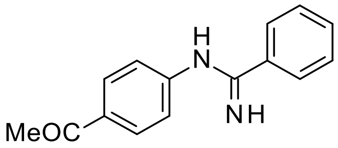

Following general procedure (GP C) and starting from 4-Chloroacetophenone the title compound was obtained as a off white solid (95.2 mg, 0.400 mmol, 80%). 0–30% (1% NEt_3 and 10% *i*PrOH in ethyl acetate) in (1% NEt_3 in cyclohexane).

^{1}H NMR (300 MHz, *DMSO-d*$_6$): δ = 7.91 (d, *J*=8.4 Hz, 4 H), 7.38 - 7.54 (m, 2 H), 6.93 (d, *J*=8.1 Hz, 2 H), 6.57 (br. s., 2 H), 2.50 - 2.55 (m, 3 H) ppm.

^{13}C NMR (75 MHz, *DMSO-d*$_6$): δ = 196.5, 156.1, 154.3, 135.4, 130.6, 130.2, 129.8, 128.0, 127.3, 121.8, 26.4 ppm.

IR (ATR): ṽ = 3313 (w), 3196 (w), 1637 (m), 1503 (vs), 1386 (m), 1239 (m), 842 (m), 871 (m), 572 (w), 508 (w) cm^{-1}

GC-HRMS (EI-QTOF): [M]+calcd. 238.1106 for $C_{15}H_{14}N_2O$; found: 238.1109.

*10.22.Synthesis of N-(4-trimethylsilylphenyl)benzamidine (**4c**)*

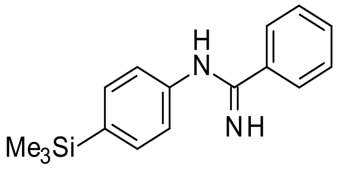

Following general procedure (GP C) and starting from (4-Chlorophenyl)trimethylsilane the title compound was obtained as a off white solid (56.1 mg, 0.209 mmol, 42%). 0–40% (1% NEt_3 and 10% *i*PrOH in ethyl acetate) in (1% NEt_3 in cyclohexane).

^{1}H NMR (300 MHz, *DMSO-d*$_6$): δ = 7.96 (d, *J*=6.4 Hz, 2 H), 7.38 - 7.54 (m, 5 H), 6.87 (d, *J*=6.8 Hz, 2 H), 6.29 (br. s., 2 H), 0.24 (s, 9 H) ppm.

^{13}C NMR (75 MHz, *DMSO-d*$_6$): δ = 153.5, 151.5, 135.8, 134.2, 131.6, 130.0, 127.9, 127.0, 121.2, -0.8 ppm.

IR (ATR): ṽ = 3484 (w), 3443 (w), 3374 (w), 2954 (w), 1627 (w), 1586 (m), 1370 (m), 1182 (m), 1079 (m), 832 (vs), 695 (vs), 630 (m), 476 (m) cm^{-1}

GC-HRMS (EI-QTOF): [M]$^+$ calcd. 268.1396 for $C_{16}H_{20}N_2Si$; found: 268.1393.

*10.23.Synthesis of N-(4-Methylsulfonylphenyl)benzenecarboximidamide (**4d**) [CAS 177662-40-7]*

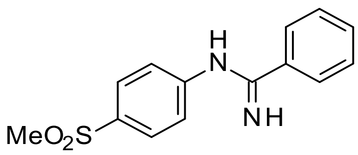

Following general procedure (GP C) and starting from 4-Chlorophenyl methyl sulfone the title compound was obtained as a off white solid (122.5 mg, 0.447 mmol, 89%). 0–60% (1% NEt_3 and 10% *i*PrOH in ethyl acetate) in (1% NEt_3 in cyclohexane).

^{1}H NMR (300 MHz, *DMSO-d*$_6$): δ = 7.93 (d, *J*=6.8 Hz, 2 H), 7.80 (d, *J*=8.5 Hz, 3 H), 7.40 - 7.53 (m, 2 H), 7.02 (d, *J*=8.3 Hz, 2 H), 6.67 (br. s.,2 H), 3.14 - 3.19 (m, 3 H) ppm.

^{13}C NMR (75 MHz, *DMSO-d*$_6$): δ = 156.3, 135.3, 133.2, 130.3, 128.4, 128.1, 127.3, 122.3, 44.0 ppm.

IR (ATR): ṽ = 3656 (w), 3462 (w), 3386 (w), 2981 (w), 2888 (m), 2360 (w), 1281 (m), 1134 (w), 865 (w), 548 (w) cm^{-1}

GC-HRMS (EI-QTOF): [M]+calcd. 74.0776 for $C_{14}H_{14}N_2O_2S$; found: 274.0775.

*10.24.Synthesis of N-p-Tolylbenzamidine (**4e**) [CAS 1859-00-3]*

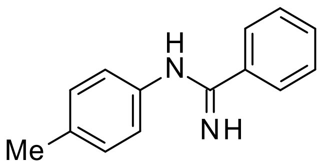

Following general procedure (GP B) and starting from 1-Chloro-4-methylbenzene the title compound was obtained as a white solid (99.6 mg, 0.474 mmol, 95%). 0–30% (1% NEt_3 and 10% *i*PrOH in ethyl acetate) in (1% NEt_3 in cyclohexane).

The NMR data are in agreement with those reported in literature.[18]

^{1}H NMR (300 MHz, *DMSO-d*$_6$): δ = 7.96 (d, *J*=6.6 Hz, 2 H), 7.34 - 7.52 (m, 3 H), 7.12 (d, *J*=7.8 Hz, 2 H), 6.78 (d, *J*=7.3 Hz, 2 H), 2.28 (s, 3 H) ppm

^{13}C NMR (75 MHz, *DMSO-d*$_6$): δ = 153.8, 143.0, 136.0, 130.3, 129.9, 129.7, 127.9, 127.0, 121.4, 20.5 ppm.

IR (ATR): ṽ = 3448 (w), 3291 (vw), 3119 (vw), 1632 (s), 1599 (s), 1232 (s), 1023 (w), 864 (s), 756(m), 497 (m), 437 (m) cm^{-1}

*10.25.Synthesis of N-(6-methoxypyridazin-3-yl)benzamidine (**4f**) [CAS 39931-61-8]*

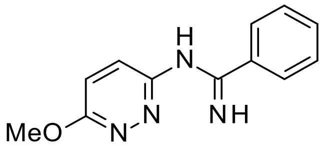

Following general procedure (GP B) and starting from 3-Chloro-6-methoxypyridazine the title compound was obtained as a off white solid (110.8 mg, 0.485 mmol, 97%). 0–30% (1% NEt_3 and 10% *i*PrOH in ethyl acetate) in (1% NEt_3 in cyclohexane).

^{1}H NMR (300 MHz, *DMSO-d*$_6$): δ = 8.03 - 8.11 (m, 2 H), 7.43 - 7.55 (m, 3 H), 7.33 (d, *J*=9.3 Hz, 1 H), 7.16 (d, *J*=9.2 Hz, 1 H), 3.99 (s, 3 H) ppm.

^{13}C NMR (75 MHz, *DMSO-d*$_6$): δ = 162.1, 160.7, 158.0, 135.8, 131.1, 130.6, 128.2, 127.3, 119.4, 54.1 ppm.

IR (ATR): ṽ = 3753 (w), 3650 (w), 2369 (w), 2207 (m), 1608 (m), 1402 (m), 1184 (w), 846 (w), 434 (vs) cm^{-1}

GC-HRMS (EI-QTOF): [M]+calcd. 227.0933 for $C_{12}H_{11}N_4O$; found: 227.0941.

*10.26. Synthesis of N-(3-Pyridyl)benzamidine (**4g**) [CAS 19673-11-1]*

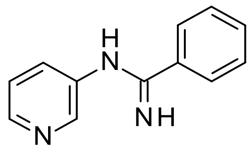

Following general procedure (GP B) and starting from 3-Chloropyridine the title compound was obtained as a off white solid (79.5 mg, 0.403 mmol, 81%). 0–100% (1% NEt_3 and 10% *i*PrOH in ethyl acetate) in (1% NEt_3 in cyclohexane).

The NMR data are in agreement with those reported in literature.[19]

^{1}H NMR (300 MHz, *DMSO-d$_6$*): δ = 8.07 - 8.27 (m, 2 H), 7.98 (d, *J*=6.6 Hz, 2 H), 7.36 - 7.56 (m, 3 H), 7.19 - 7.36 (m, 2 H), 6.57 (br. s., 2 H) ppm.

^{13}C NMR (75 MHz, *DMSO-d$_6$*): δ = 155.0, 146.9, 143.6, 142.7, 135.6, 130.2, 128.7, 128.0, 127.1, 123.9, 40.3, 40.1, 39.8, 39.2, 39.0, 38.7 ppm.

IR (ATR): ṽ = 3377 (w), 3320 (w), 2920 (m), 1645 (m), 1608 (vs), 1569 (vs), 1500 (vs), 1407 (vs), 1187 (vs), 1100 (m), 970 (m), 691 (vs), 477 (w), 448 (w) cm^{-1}

GC-HRMS (EI-QTOF): [M]+calcd. 197.0953 for $C_{12}H_{11}N_3$; found: 197.0952.

*10.27. Synthesis of N-(2-Pyridyl)benzamidine (**4h**) [CAS 4931-08-2]*

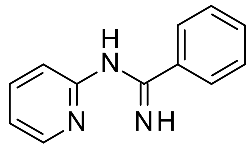

Following general procedure (GP B) and starting fom 2-Chloropyridine the title compound was obtained as a off white solid (89,3 mg, 0.453 mmol, 91%). 0–100% (1% NEt_3 and 10% *i*PrOH in ethyl acetate) in (1% NEt_3 in cyclohexane).

The NMR data are in agreement with those reported in literature.[20]

^{1}H NMR (300 MHz, *DMSO-d$_6$*): δ = 8.34 (dd, *J*=5.0, 1.4 Hz, 1 H), 8.05 (dd, *J*=7.7, 1.8 Hz, 2 H), 7.71 (ddd, *J*=8.1, 7.3, 2.1 Hz, 1 H), 7.42 - 7.53 (m, 3 H), 7.11 (d, *J*=8.2 Hz, 1 H), 6.98 (ddd, *J*=7.2, 5.0, 1.1 Hz, 1 H) ppm.

^{13}C NMR (75 MHz, *DMSO-d$_6$*): δ = 162.9, 157.9, 146.1, 137.6, 136.4, 130.4, 128.1, 127.2, 121.8, 117.3 ppm.

IR (ATR): ṽ = 2980 (w), 2866 (w), 2156 (m), 1621 (m), 1383 (m), 1252 (m), 954 (m), 776 (m), 697 (vw), 489 (vw) cm^{-1}

HPLC-HRMS (ESI): $[M+H]^+$ calcd. 198.1026 for $C_{12}H_{11}N_3$; found: 198.1019.

*10.28.Synthesis of 3-(4-Methoxyphenyl)-1-phenylurea (**5a**) [CAS 232597-42-1]*

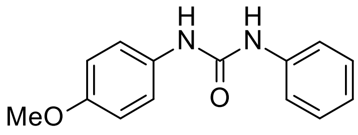

Following general procedure (GP C) and starting from 4-Chloranisol the title compound was obtained as a off white solid (110.8 mg, 0.457 mmol, 92%). 0–60% (1% NEt_3 and 10% *i*PrOH in ethyl acetate) in (1% NEt_3 in cyclohexane).

The NMR data are in agreement with those reported in literature.[21]

^{1}H NMR (300 MHz, *DMSO-d*$_6$): δ = 8.55 (s, 1 H), 8.44 (s, 1 H), 7.39 - 7.46 (m, 2 H), 7.31 - 7.38 (m, 2 H), 7.26 (t, *J*=7.9 Hz, 2 H), 6.91 - 6.98 (m, 1 H), 6.82 - 6.90 (m, 2 H), 3.71 (s, 3 H) ppm.

^{13}C NMR (75 MHz, *DMSO-d*$_6$): δ = 154.4, 152.7, 139.9, 132.7, 128.7, 121.6, 120.0, 118.1, 114.0, 55.2 ppm.

IR (ATR): ṽ = 3288 (w), 3068 (vw), 2834 (vw), 1507 (vw), 1442 (vw), 1244 (m), 1179 (w), 1154 (vs), 1028 (vs), 499 (m), 453 (m) cm^{-1}

GC-HRMS (EI-QTOF): [M]$^+$calcd. 242.1055 for $C_{14}H_{14}N_2O_2$; found: 242.1056.

*10.29.Synthesis of 1-Phenyl-3-[4-(trifluoromethyl)phenyl]urea (**5b**) [CAS 23750-69-8]*

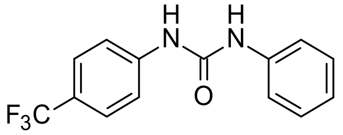

Following general procedure (GP C) and starting from 4-Chlorobenzotrifluoride the title compound was obtained as a off white solid (132.6 mg, 0.473 mmol, 95%). 0–40% (1% NEt_3 and 10% *i*PrOH in ethyl acetate) in (1% NEt_3 in cyclohexane).

The NMR data are in agreement with those reported in literature.[22]

^{1}H NMR (300 MHz, *DMSO-d*$_6$): δ = 9.08 (s, 1 H), 8.78 (s, 1 H), 7.60 - 7.68 (m, 4 H), 7.46 (d, *J*=7.6 Hz, 2 H), 7.29 (t, *J*=7.9 Hz, 2 H), 6.96 - 7.04 (m, 1 H) ppm.

^{13}C NMR (75 MHz, *DMSO-d*$_6$): δ = 163.9, 155.8, 138.8, 131.9, 128.5, 125.3 (q), 122.0, 113.8, 55.2 ppm.

^{19}F NMR (76 MHz, DMSO-*d*$_6$, $C_6H_4F_2$): δ = - 61.9 ppm.

IR (ATR): ṽ = 3304 (w), 1688 (m), 1447 (m), 1181 (m), 1066 (m), 989 (vs), 896 (vs), 637 (s), 499 (w), 409 (vw) cm^{-1}

GC-HRMS (EI-QTOF): [M]+calcd. 280.0824for $C_{14}H_{11}F_3N_2O$; found: 280.08200.

*10.30. Synthesis of 1-(4-Methylphenyl)-3-phenylurea (**5c**) [CAS 4300-33-8]*

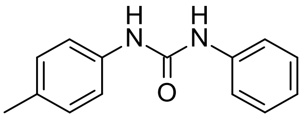

Following general procedure (GP C) and starting from 4-chlorotoluene the title compound was obtained as a off white solid (107 mg, 0.473 mmol, 95%). 0–40% (1% NEt_3 and 10% *i*PrOH in ethyl acetate) in (1% NEt_3 in cyclohexane).

The NMR data are in agreement with those reported in literature.[23]

^{1}H NMR (300 MHz, *DMSO-d*6): δ = 8.59 (s, 1 H), 8.52 (s, 1 H), 7.41 - 7.46 (m, 2 H), 7.33 (d, *J*=8.4 Hz, 2 H), 7.27 (t, *J*=7.9 Hz, 2 H), 7.08 (d, *J*=8.3 Hz, 2 H), 6.92 - 6.99 (m, 1 H), 2.24 (s, 3 H) ppm.

^{13}C NMR (75 MHz, *DMSO-d*6): δ = 152.5, 139.8, 137.1, 130.6, 129.1, 128.7, 121.7, 118.2, 118.1, 20.3 ppm.

IR (ATR): ṽ = 3292 (m), 2855 ,, 2726 (w), 1590 (m), 1560 (m), 1442 (m), 1376 (m), 787 (m), 731 (m), 691 (m), 507 (m) cm^{-1}

GC-HRMS (EI-QTOF): $[M]^+$ calcd. 226.1106 for $C_{14}H_{14}N_2O$; found: 226.1106

*10.31. Synthesis of 1-(2-Methylphenyl)-3-phenylurea (**5d**) [CAS 23750-69-8]*

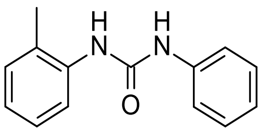

Following general procedure (GP C) and starting from 2-chlorotoluene the title compound was obtained as a off white solid (98.2 mg, 0.434 mmol, 87%). 0–40% (1% NEt_3 and 10% *i*PrOH in ethyl acetate) in (1% NEt_3 in cyclohexane).

The NMR data are in agreement with those reported in literature.[24]

^{1}H NMR (300 MHz, *DMSO-d*6): δ = 9.01 (s, 1 H), 7.91 (s, 1 H), 7.84 (d, *J*=7.5 Hz, 1 H), 7.42 - 7.51 (m, 2 H), 7.24 - 7.33 (m, 2 H), 7.09 - 7.20 (m, 2 H), 6.90 - 7.00 (m, 2 H), 2.24 (s, 3 H) ppm.

^{13}C NMR (75 MHz, *DMSO-d*6): δ = 152.6, 139.9, 137.4, 130.2, 128.8, 127.5, 126.1, 122.6, 121.7, 121.0, 118.0, 17.9 ppm.

IR (ATR): ṽ = 3291 (w), 3034 (vw), 1592 (vs), 1309 (m), 565 (m), 447 (w), 410 (w) cm^{-1}

GC-HRMS (EI-QTOF): $[M]^+$ calcd. 226.1106 for $C_{14}H_{14}N_2O$; found: 226.1105.

*10.32. Synthesis of 1-(4-isopropenylphenyl)-3-phenyl-urea (**5e**)*

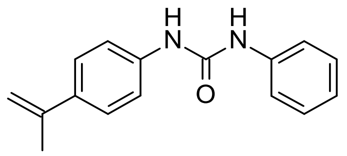

Following general procedure (GP C) and starting from 4-Chloro-α-methylstyrene the title compound was obtained as a white solid (110.0 mg, 0.436 mmol, 87%). 0–40% (1% NEt_3 and 10% *i*PrOH in ethyl acetate) in (1% NEt_3 in cyclohexane).

^{1}H NMR (300 MHz, *DMSO-d_6*): δ = 8.60 - 8.75 (m, 2 H), 7.40 - 7.48 (m, 6 H), 7.22 - 7.33 (m, 2 H), 6.92 - 7.01 (m, 1 H), 5.33 - 5.39 (m, 1 H), 5.00 (t, J=1.4 Hz, 1 H), 2.08 (s, 3 H) ppm.

^{13}C NMR (75 MHz, *DMSO-d_6*): 152.4, 141.9, 139.6, 139.2, 133.7, 128.8, 125.7, 121.8 (s) 118.2, 117.8, 110.7, 21.5 ppm.

IR (ATR): ṽ = 3322 (w), 2955 (m), 1618 (m), 1511 (s), 1322 (s),1014 (m), 889 (s), 693 (s), 504 (m) cm^{-1}

HPLC-HRMS (ESI): $[M+Na]^+$ calcd. 275.1155 for $C_{12}H_{11}N_3O_2$; found: 275.1146.

*10.33. Synthesis of N-(2-Methyl-5-benzoxazolyl)-N-phenylurea (**5f**) [CAS : 1787872-65-4]*

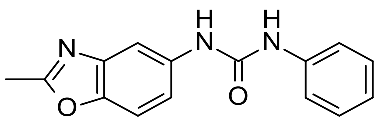

Following general procedure (GP C) and starting from 5-Chloro-2-methylbenzoxazole the title compound was obtained as a off white solid (121.7 mg, 0.456 mmol, 91%). 0–50% (1% NEt_3 and 10% *i*PrOH in ethyl acetate) in (1% NEt_3 in cyclohexane).

^{1}H NMR (300 MHz, *DMSO-d_6*): δ = 8.75 (s, 1 H), 8.65 (s, 1 H), 7.85 (d, *J*=2.0 Hz, 1 H), 7.54 (d, *J*=8.8 Hz, 1 H), 7.41 - 7.49 (m, 2 H), 7.23 - 7.32 (m, 3 H), 6.93 - 7.01 (m, 1 H), 2.58 (s, 3 H) ppm.

^{13}C NMR (75 MHz, *DMSO-d_6*): δ = 164.4, 152.7, 145.8, 141.5, 139.7, 136.3, 128.8, 121.8, 118.2, 115.8, 111.1, 108.5, 14.1 ppm.

IR (ATR): ṽ = 3753 (vw), 3286 (vw), 1481 (w), 1639 (vs), 1255 (s),1178 (m), 793 (m), 661 (m), 416 (vw) cm^{-1}

GC-HRMS (EI-QTOF): $[M]^+$ calcd. 267.1008 for $C_{15}H_{13}N_3O_2$; found: 267.1008.

*10.34.Synthesis of N-Phenyl-N'-3-thienylurea (**5g**) [CAS :2361877-12-3]*

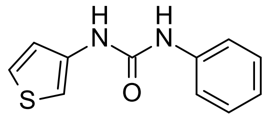

Following general procedure (GP C) and starting from 3-Chlorothiophene the title compound was obtained as a white solid (96.8 mg, 0.443 mmol, 89%). 0–100% (1% NEt_3 and 10% *i*PrOH in ethyl acetate) in (1% NEt_3 in cyclohexane).

The NMR data are in agreement with those reported in literature.

^{1}H NMR (300 MHz, *DMSO-d$_6$*): δ = 8.48 (s, 1 H), 7.33 - 7.43 (m, 2 H), 7.14 - 7.27 (m, 2 H), 6.82 - 6.94 (m, 1 H), 5.81 (s, 1 H) ppm.

^{13}C NMR (75 MHz, *DMSO-d$_6$*): δ = 156.0, 140.5, 128.6, 121.1, 117.7 ppm.

IR (ATR): ṽ = 3419 (m), 3310 (s), 3036 (w), 1650 (s), 1590 (s),1353 (m),1113 (m), 750 (s), 694 cm^{-1}

*10.35.Synthesis of tert-Butyl N-(4-methoxyphenyl)carbamate (**6a**) [CAS 18437-68-8]*

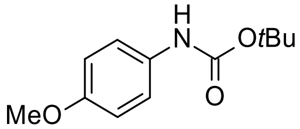

Following general procedure (GP D) and starting from 4-Chloranisol the title compound was obtained as a off white solid (107.8 mg, 0.483 mmol, 97%). 0–30% ethyl acetate in cyclohexane.

The NMR data are in agreement with those reported in literature.[25]

^{1}H NMR (300 MHz, $CDCl_3$): δ = 7.13 - 7.24 (m, 2 H), 6.71 - 6.79 (m, 2 H), 6.31 (br. s, 1 H), 3.70 (s, 3 H), 1.43 (s, 9 H) ppm.

^{13}C NMR (75 MHz, $CDCl_3$): δ = 155.6, 153.2, 131.4, 120.6, 114.1, 80.2, 55.5, 28.3 ppm.

IR (ATR): ṽ = 3364 (m), 1692 (s), 1233 (m), 1024 (m), 790 (m), 772 (m), 622 (m), 560(m) cm^{-1}

GC-HRMS (EI-QTOF): $[M]^+$calcd. 223.1208 for $C_{12}H_{17}NO_3$; found: 223.1211.

10.36.Synthesis of tert-Butyl 4-trifluoromethylphenylcarbamate ***(6b)*** *[CAS 141940-37-6]*

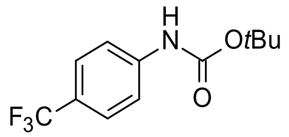

Following general procedure (GP D) and starting from 4-Chlorobenzotrifluoride the title compound was obtained as a off white solid (118.1 mg, 0.452 mmol, 90%). 0–10% ethyl acetate in cyclohexane.

The NMR data are in agreement with those reported in literature.[26]

^{1}H NMR (300 MHz, $CDCl_3$): δ = 7.52 - 7.58 (m, 2 H), 7.45 - 7.51 (m, 2 H), 6.63 (br. s., 1H), 1.54 (s, 9 H) ppm.

^{13}C NMR (75 MHz, $CDCl_3$): δ = 152.3, 141.5, 126.2 (q) 125.0, 124.6, 117.8, 81.3, 28.2 ppm.

^{19}F NMR (76 MHz, $CDCl_3$, $C_6H_4F_2$): δ = -63.3 ppm.

IR (ATR): ṽ = 3364 (w), 3013(w), 2985 (w), 2940 (vw), 1702 (vw), 1505 (m), 1331 (m), 1154 (m), 1112 (m), 1069 (m), 610 (w), 507 (vw) cm^{-1}

GC-HRMS (EI-QTOF): [M]+calcd. 261.0977 for $C_{12}H_{14}F_3NO_2$; found: 261.0980.

*10.37.*Synthesis *of Ethyl 4-((tert-butoxycarbonyl)amino)benzoate (**6c**) [CAS 110969-44-3]*

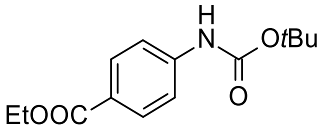

Following general procedure (GP D) and starting from Ethyl 4-chlorobenzoate the title compound was obtained as a off white solid (120.3 mg, 0.453 mmol, 91%). 0–10% ethyl acetate in cyclohexane.

The NMR data are in agreement with those reported in literature.[27]

^{1}H NMR (300 MHz, $CDCl_3$): δ = 7.99 (d, J=8.7 Hz, 2 H), 7.43 (d, J=8.7 Hz, 2 H), 6.65 (br. s., 1H), 4.36 (q, J=7.1 Hz, 2 H), 1.54 (s, 9 H), 1.39 (t, J=7.1 Hz, 3 H) ppm.

^{13}C NMR (75 MHz, $CDCl_3$): δ = 166.3, 152.2, 142.6, 130.8, 124.7, 117.3, 81.2, 60.7, 28.3, 14.3 ppm.

IR (ATR): ṽ = 3322 (w), 2980 ,, 2935 (vw), 1698 (m), 1596 (m), 1529 (m), 1414 (m), 1362 (m), 1050 (vs) cm^{-1}

GC-HRMS (EI-QTOF): $[M]^+$calcd. 265.1314 for $C_{14}H_{19}NO_4$; found: 265.1315

*10.38.Synthesis of tert-*Butyl *(4-acetylphenyl)carbamate (**6d**) [CAS 232597-42-1]*

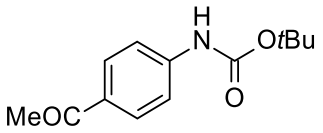

Following general procedure (GP D) and starting from 4-Chloroacetophenone the title compound was obtained as a off white solid (98.7 mg, 0.458 mmol, 84%). 0–40% ethyl acetate in cyclohexane.

The NMR data are in agreement with those reported in literature.[28]

^{1}H NMR (300 MHz, $CDCl_3$): δ = 7.92 (d, *J*=8.8 Hz, 2 H), 7.46 (d, *J*=8.8 Hz, 2 H), 6.69 (br. s., 1 H), 2.57 (s, 3 H), 1.54 (s, 9 H) ppm.

^{13}C NMR (75 MHz, $CDCl_3$): δ = 196.9, 152.1, 142.9, 131.8, 130.9 - 133.6 (m) 129.8, 117.4, 81.3, 28.2, 26.3 ppm.

IR (ATR): ṽ = 3281 (w), 2981 (vw), 1717 (vw), 1670 (vs), 1597 (m), 1530 (m), 1155 (vs), 1074 (vs), 832 (m), 586 (m), 494 (m), 459 (vw) cm^{-1}

GC-HRMS (EI-QTOF): $[M]^+$calcd. 235.1208 for $C_{13}H_{17}NO_3$; found: 235.1207.

*10.39.Synthesis of tert-butyl N-(4-trimethylsilylphenyl)carbamate (**6e**) [CAS 2231316-68-8]*

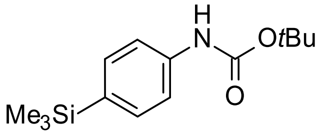

Following general procedure (GP D) and starting from (4-Chlorophenyl)(trimethyl)silane the title compound was obtained as a white solid (127.7 mg, 0.480 mmol, 69%). 0–20% ethyl acetate in cyclohexane.

The NMR data are in agreement with those reported in literature.[29]

^{1}H NMR (300 MHz, *DMSO-d*$_6$): δ = 9.32 (s, 1 H), 7.33 - 7.48 (m, 4 H), 1.47 (s, 9 H), 0.20 (s, 9 H) ppm.

^{13}C NMR (75 MHz, *DMSO-d*$_6$): δ = 152.7, 140.2, 133.6, 132.2, 117.5, 79.0, 28.1, -1.0 ppm.

IR (ATR): ṽ = 3319 (m), 2977 (m), 2931 (s), 1691 (s), 1587 (s), 1392 (s), 1321 (s), 819 (s), 519 (s) cm^{-1}

*10.40.Synthesis of 1,1-Dimethylethyl N-3-thienylcarbamate (**6f**) [CAS 19228-91-2]*

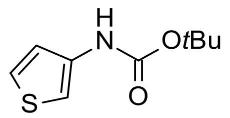

Following general procedure (GP D) and starting from 3-Chlorothiophene the title compound was obtained as a off white solid (75.8 mg, 0.380 mmol, 76%). 0–20% ethyl acetate in cyclohexane.

The NMR data are in agreement with those reported in literature.[30]

^{1}H NMR (300 MHz, *DMSO-d*$_6$): δ = 9.61 (br. s, 1 H), 7.38 (dd, J=5.1, 3.2 Hz, 1 H), 7.16 (br. s, 1 H), 6.98 (dd, J=5.1, 1.0 Hz, 1 H), 1.46 (s, 9 H) ppm.

^{13}C NMR (101 MHz, *DMSO-d*$_6$): δ = 152.7, 137.3, 124.6, 121.1, 105.7, 78.9, 28.1 ppm.

IR (ATR): ṽ = 3311 (m), 3143 (m), 1687 (s), 1575 (s), 1390 (m), 1150 (s), 858 (s), 664 (m), 460 (w) cm^{-1}

*10.41.Synthesis of (4-Methanesulfonyl-phenyl)carbamic acid tert-butyl ester (**6g**) [CAS 1096711-96-4]*

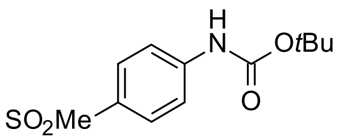

Following general procedure (GP D) and starting from 4-Chlorophenyl methyl sulfone the title compound was obtained as a off white solid (124.3 mg, 0.458 mmol, 92%). 0–40% ethyl acetate in cyclohexane.

^{1}H NMR (300 MHz, $CDCl_3$): δ = 7.84 - 7.90 (m, 2 H), 7.52 - 7.61 (m, 2 H), 6.74 (br. s., 1 H), 3.04 (s, 3 H), 1.54 (s, 9 H) ppm.

^{13}C NMR (75 MHz, $CDCl_3$): δ = 152.0, 143.5, 134.0, 128.8, 118.0, 81.7, 44.7, 28.2 ppm.

IR (ATR): ṽ =3368 (w), 3005 (vw), 2935 (vw), 1729 (m), 1522 (m), 1404 (vw), 1049 (vs), 898 (m), 753 (m), 530 (vs) cm^{-1}

HPLC-HRMS (ESI): $[M+H]^+$ calcd. 272.0951 for $C_{12}H_{18}NO_4S$; found: 272.0944.

*10.42.Synthesis of 4-Methyl-N-[4-(trifluoromethyl)phenyl]benzenesulfonamide (**7a**) [CAS 107491-54-3]*

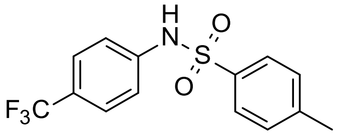

Following general procedure (GP E) and starting from 4-Chlor-trifluortoluolthe title compound was obtained as a white solid (145.4 mg, 0.461 mmol, 92%). 0–30% ethyl acetate in cyclohexane.

The NMR data are in agreement with those reported in literature. [5]

^{1}H NMR (300 MHz, $CDCl_3$): *δ* = 7.67 (d, *J*=8.4 Hz, 2 H), 7.40 (d, *J*=8.6 Hz, 2 H), 7.15 - 7.21 (m, 2 H), 7.12 (d, *J*=8.6 Hz, 2 H), 2.31 (s, 3 H) ppm.

^{13}C NMR (75 MHz, $CDCl_3$): *δ* = 144.5, 140.0, 135.7, 129.9, 129.8, 127.2, 126.6 (q), 125.2, 122.5, 119.6, 21.5 ppm.

*10.43.Synthesis of N-(4-acetylphenyl)-4-methyl-benzenesulfonamide (**7b**) [CAS 5317-94-2]*

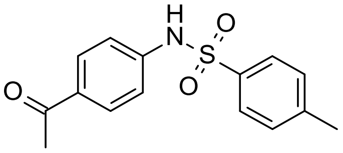

Following general procedure (GP E) and starting from 4-Chloroacetophenone the title compound was obtained as a white solid (127.0 mg, 0.439 mmol, 88%). 0–50% ethyl acetate in cyclohexane.

The NMR data are in agreement with those reported in literature.[31]

^{1}H NMR (300 MHz, $CDCl_3$): δ = 7.83 - 7.89 (m, 2 H), 7.75 - 7.75 (m, 2 H), 7.73 (d, *J*=8.3 Hz, 2 H), 7.27 (m, 3 H), 7.12 - 7.20 (m, 2 H), 6.90 (s, 1 H), 2.54 (s, 3 H), 2.40 (s, 3 H) ppm

^{13}C NMR (75 MHz, $CDCl_3$): δ = 144.5, 141.0, 135.8, 133.3, 130.0, 129.9, 127.2, 119.0, 26.4, 21.6 ppm.

IR (ATR): ṽ = 3217 (m), 2949 (m), 2853 (m), 1691 (vs), 1433 (vs), 1297 (vs), 1162 (vs), 1092 (s), 916 8s), 765 (w), 668 (w), 546 (m) cm^{-1}.

GC-HRMS (EI-QTOF): [M]+calcd. 289.0773 for $C_{15}H_{15}NO_3S$; found: 289.0774.

*10.44.Synthesis of methyl 4-(p-tolylsulfonylamino)benzoate (**7c**) [CAS 158038-67-6]*

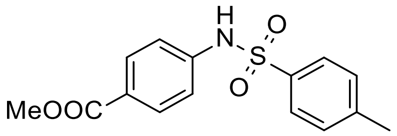

Following general procedure (GP E) and starting from Methyl 4-chlorobenzoatethe title compound was obtained as a white solid (133.0 mg, 0.436 mmol, 87%). 0–30% ethyl acetate in cyclohexane.

The NMR data are in agreement with those reported in literature.[32]

^{1}H NMR (300 MHz, $CDCl_3$): δ = 10.80 (br. s., 1 H), 7.78 - 7.85 (m, 2 H), 7.70 (d, *J*=8.3 Hz, 2 H), 7.35 (d, *J*=8.1 Hz, 2 H), 7.16 - 7.26 (m, 2 H), 3.77 (s, 3 H), 2.32 (s, 3 H) ppm.

^{13}C NMR (75 MHz, DMSO-d^6): 165.6, 143.6, 142.6, 136.4, 130.6, 129.8, 126.7, 124.2, 118.1, 51.9, 20.9 ppm.

IR (ATR): ṽ = 3217 (m), 2949 (m), 2923 (m), 2853 (vw), 1691 (w), 1608 (m), 1433 (m), 1405 (m), 1297 (m), 1162 (vs), 916 (m), 668 (m) cm-1

GC-HRMS (EI-QTOF): [M]+calcd. 305.0722 for $C_{15}H_{15}NO_4S$; found: 305.0722.

*10.45.Synthesis of 4-methyl-N-(o-tolyl)benzenesulfonamide (**7d**) [CAS 80-28-4]*

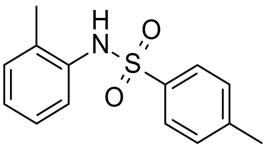

Following general procedure (GP E) and starting from 2-Chlorotoluenethe title compound was obtained as a white solid (119.0 mg, 0.455 mmol, 91%). 0–30% ethyl acetate in cyclohexane.

The NMR data are in agreement with those reported in literature.[33]

^{1}H NMR (300 MHz, $CDCl_3$): δ = 7.61 (d, *J*=8.3 Hz, 2 H), 7.32 (d, *J*=7.7 Hz, 1 H), 7.23 (d, *J*=8.0 Hz, 2 H), 7.05 - 7.19 (m, 3 H), 6.22 (s, 1 H), 2.40 (s, 3 H), 2.01 (s, 3 H) ppm.

^{13}C NMR (75 MHz, $CDCl_3$): δ = 143.7, 136.7, 134.5, 131.5, 130.7, 129.5, 127.1, 126.8, 126.1, 124.3, 21.5, 17.5 ppm.

IR (ATR): ṽ = 3267 (m),1581 (w), 1379 (m), 1287 (w), 1090 (w), 907 (m), 754 (w), 602 (m), 569 (m), 494 (m), 451 (m), 427 (w) cm^{-1}

GC-HRMS (EI-QTOF): [M]+calcd. 261.0824 for $C_{14}H_{15}NO_2S$; found: 261.0829

*10.46.Synthesis of 4-methyl-N-(1-naphthyl)benzenesulfonamide (**7e**) [CAS 18271-17-5]*

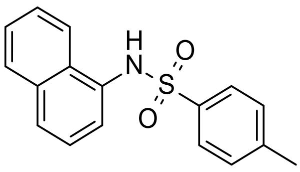

Following general procedure (GP E) and starting from 1-Chloronaphthalenethe title compound was obtained as a white solid (149.0 mg, 0.471 mmol, 94%). 0–30% ethyl acetate in cyclohexane.

The NMR data are in agreement with those reported in literature.[34]

^{1}H NMR (300 MHz, $CDCl_3$): δ = 7.68 - 7.87 (m, 3 H), 7.60 - 7.68 (m, 2 H), 7.33 - 7.51 (m, 4 H), 7.12 - 7.24 (m, 2 H), 6.77 (s, 1H), 2.35 (s, 3 H) ppm.

^{13}C NMR (75 MHz, $CDCl_3$): 143.7, 136.3, 134.2, 131.5, 129.6, 129.5, 128.9, 128.3, 127.3, 127.2, 127.1, 126.5, 126.2, 125.3, 122.6, 121.6, 21.4 ppm.

IR (ATR): ṽ = 3262 (w), 3065 (w), 2980 (w), 1679 (m), 1513 (m), 1413 (vs), 1341 (vs), 1155 (vs), 1018 (m), 796 (m), 660 , cm^{-1}.

GC-HRMS (EI-QTOF): [M]+calcd. 261.0824 for $C_{14}H_{15}NO_2S$; found: 261.0829.

*10.47.Synthesis of 4-Methylaniline (**8a**) [CAS 106-49-0]*

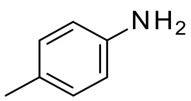

Following general procedure (GP F) and starting from 4-Chlorotoluen the title compound was obtained as a brown solid (49.8 mg, 0.465 mmol, 93%). 0–40% (1% NEt3 in ethyl acetate) in (1% NEt3 in cyclohexane).

The NMR data are in agreement with those reported in literature.[35]

^{1}H NMR (300 MHz, $CDCl_3$): δ = 6.98 (d, J=8.3 Hz, 2 H), 6.59 - 6.67 (m, 2 H), 2.23 - 2.28 (m, 3 H) ppm.

^{13}C NMR (75 MHz, $CDCl_3$): δ = 143.8, 129.7, 127.8, 115.2, 20.4 ppm.

IR (ATR): ṽ = 3416 (m), 3335 (m), 3053 (w), 3009 (m), 1267 (m), 1177 (m), 810 (m), 602 (m), 503 (m) cm^{-1}

GC-HRMS (EI-QTOF): $[M]^+$ calcd. 107.0735 for C_7H_9N; found: 107.0735.

*10.48.Synthesis of 4-Methoxyaniline_(**8b**) [CAS 104-94-9]*

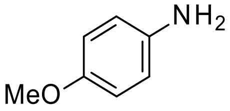

Following general procedure (GP F) and starting from 4-Chloranisol the title compound was obtained as a brown solid (57.6 mg, 0.468 mmol, 94%). 0–100% (1% NEt_3 in ethyl acetate) in (1% NEt_3 incyclohexane).

The NMR data are in agreement with those reported in literature.[36]

^{1}H NMR (300 MHz, $CDCl_3$): δ = 6.72 - 6.79 (m, 2 H), 6.62 - 6.70 (m, 2 H), 3.76 (s, 3 H), 3.42 (br. s., 2 H) ppm.

^{13}C NMR (75 MHz, $CDCl_3$): δ = 152.8, 139.9, 116.4, 114.8, 55.7 ppm

IR (ATR): ṽ = 3382 (w), 3214 (w), 3002 (vw), 2835 (vw), 1507 (m), 1229 (m), 1179 (m), 1032 (m), 822 (m), 715 (m), 513 (m), 430 (vw) cm^{-1}

GC-HRMS (EI-QTOF): $[M]^+$ calcd. 123.0684 for C_7H_9NO; found: 123.0686

*10.49.Synthesis of Quinolin-6-amine (**8c**) [CAS 580-15-4]*

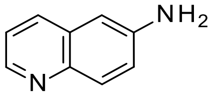

F

Following general procedure (GP F) and starting from 6-Chloroquinoline the title compound was obtained as a brown solid (49.7 mg, 0.38 mmol, 76%). 0–100% (1% NEt_3 in ethyl acetate) in (1% NEt_3 in cyclohexane).

The NMR data are in agreement with those reported in literature.[36]

^{1}H NMR (300 MHz, $CDCl_3$): δ = 8.64 (dd, *J*=4.2, 1.6 Hz, 2 H), 7.79 - 7.95 (m, 2 H), 7.24 (dd, *J*=8.3, 4.2 Hz, 2 H), 7.13 (dd, *J*=8.9, 2.6 Hz, 2 H), 6.86 (d, *J*=2.5 Hz, 2 H), 3.82 - 4.18 (m, 2 H) ppm.

^{13}C NMR (75 MHz, $CDCl_3$): δ = 146.7, 144.6, 143.3, 133.6, 130.4, 129.7, 121.5, 121.3, 107.3 ppm.

IR (ATR): ṽ = 3392 (vw), 3314 (vw), 3179 (w), 3052 (w), 2925 (w), 1467 (m), 1280 (m), 1121 (m), 824 (m), 713 (m), 539 (m), 470 (m) cm^{-1}

GC-HRMS (EI-QTOF): $[M]^+$ calcd. 144.0688 for $C_9H_8N_2$; found: 144.0690

*10.50.Synthesis of 4-Aminobiphenyl (**8d**) [CAS 92-67-1]*

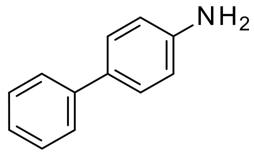

Following general procedure (GP F) and starting from 4-Chlorobiphenyl the title compound was obtained as a brown solid (57.6 mg, 0.468 mmol, 94%). 0–50% (1% NEt_3 in ethyl acetate) in (1% NEt_3 in cyclohexane).

The NMR data are in agreement with those reported in literature.[35]

^{1}H NMR (300 MHz, $CDCl_3$): δ = 7.57 - 7.64 (m, 2 H), 7.41 - 7.52 (m, 4 H), 7.29 - 7.38 (m, 1 H), 6.75 - 6.84 (m, 2 H), 3.74 (br. s., 2 H) ppm.

^{13}C NMR (75 MHz, $CDCl_3$): δ = 145.8, 141.1, 131.4, 128.6, 127.9, 126.3, 126.2, 115.3 ppm.

IR (ATR): ṽ = 3666 (vw), 3424 (vw), 3187 (w), 2981 (w), 1599 (m), 1518 (vs), 1418 (vs), 1256 (vs), 1078 (m), 831 (m), 759 (m), 692 (m) cm^{-1}.

GC-HRMS (EI-QTOF): $[M]^+$ calcd. 169.0892 for $C_{12}H_{11}N$; found: 169.0893.

*10.51.Synthesis of N-cyclopropyl-4-(trifluoromethyl)aniline (**9a**) [CAS 1249999-66-3]*

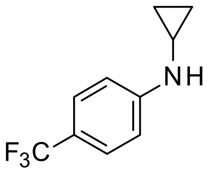

Following general procedure (GP G) and from 4-Chlorobenzotrifluoride the title compound was obtained as a colorless oil (60.7 mg, 0.477 mmol, 95%). 0–10% (1% NEt_3 in ethyl acetate) in (1% NEt_3 in cyclohexane).

The NMR data are in agreement with those reported in literature.[37]

^{1}H NMR (300 MHz, $CDCl_3$): δ = 7.43 (d, J=8.5 Hz, 2 H), 6.80 (d, J=8.5 Hz, 2 H), 4.45 (br. s., 1 H), 2.47 (tt, J=6.4, 3.4 Hz, 1 H), 0.74 - 0.87 (m, 2 H), 0.48 - 0.62 (m, 2 H) ppm.

^{13}C NMR (75 MHz, $CDCl_3$): δ = 151.1, 126.5 (q),119.5, 116.5 (m), 112.4, 24.9, 7.5 ppm

^{19}F NMR (76 MHz, $CDCl_3$, $C_6H_4F_2$): δ = -62.3 ppm.

IR (ATR): ṽ = 3382 (m), 3317 (w), 3214 (w), 3002 (w), 2914 (w), 2835 (w), 1507 (m), 1229 (m), 822 (m), 430 (w) cm^{-1}

GC-HRMS (EI-QTOF): $[M]^+$ calcd. 201.0765 for $C_{10}H_{10}F_3N$; found: 201.0764.

*10.52. Synthesis of Ethyl 4-(cyclopropylamino)benzoate (**9b**) [CAS 112033-48-4]*

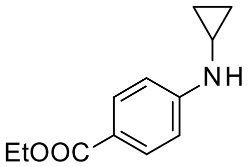

Following general procedure (GP G) and starting from Ethyl 4-Chlorobenzoate the title compound was obtained as a colorless oil (78.7 mg, 0.383 mmol, 77%). 0–10% (1% NEt_3 in ethyl acetate) in (1% NEt_3 in cyclohexane).

The NMR data are in agreement with those reported in literature.[38]

^{1}H NMR (300 MHz, $CDCl_3$): δ = 7.85 - 7.93 (m, 2 H), 6.70 - 6.78 (m, 2 H), 4.53 (br. s., 1 H), 4.33 (q, *J*=7.2 Hz, 2 H), 2.42 - 2.55 (m, 1 H), 1.37 (t, *J*=7.1 Hz, 3 H), 0.76 - 0.85 (m, 2 H), 0.50 - 0.59 (m, 2 H) ppm.
^{13}C NMR (75 MHz, $CDCl_3$): δ = 166.9, 152.4, 131.3, 119.3, 112.0, 60.2, 24.8, 14.4, 7.6 ppm.

IR (ATR): ṽ = 3351 (m), 2979 (m), 1675 (m), 1601 (m), 1276 (m), 1167 (m), 1021 (w), 840 (m), 770 (m) cm^{-1}

GC-HRMS (EI-QTOF): $[M]^+$ calcd. 205.1103 for $C_{12}H_{15}NO_2$; found: 205.1103.

*10.53. Synthesis of N-cyclopropylpyridin-2-amine (**9c**) [CAS 950577-07-8]*

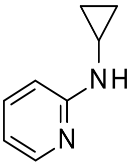

Following general procedure (GP G) and starting from 2-Chloropyridine the title compound was obtained as a off white solid (60.7 mg, 0.452 mmol, 91%). 0–40% (1% NEt_3 in ethyl acetate) in (1% NEt_3 in cyclohexane).

The NMR data are in agreement with those reported in literature.[39]

^{1}H NMR (300 MHz, $CDCl_3$): δ = 8.01 - 8.14 (m, 1 H), 7.42 - 7.54 (m, 1 H), 6.74 (d, *J*=8.3 Hz, 1 H), 6.57 - 6.67 (m, 1 H), 5.12 (br. s., 1 H), 2.50 (m, *J*=6.7, 1 H), 0.72 - 0.84 (m, 2 H), 0.49 - 0.62 (m, 2 H) ppm.

^{13}C NMR (75 MHz, $CDCl_3$): δ = 159.8, 148.2, 137.6, 113.4, 106.1, 24.0, 7.6 ppm.

IR (ATR): ṽ = 3223 (w), 3085 (w), 2980 (m), 1575 (m), 1438 (m), 1362 (m), 1149 (m), 981 (m), 769 (m), 607 (m), 526 (m) cm^{-1}.

GC-HRMS (EI-QTOF): $[M]^+$ calcd. 134.0844 for $C_8H_{10}N_2$; found: 134.0843.

*10.54.Synthesis of N-cyclopropylquinolin-2-amine (**9d**) [CAS 855760-19-9]*

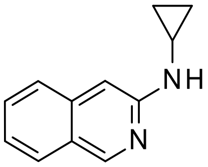

Following general procedure (GP G) and starting from 2-Chloroquinoline the title compound was obtained as a colorless oil (85.0 mg, 0.461 mmol, 88%). 0–50% (1% NEt_3 in ethyl acetate) in (1% NEt_3 in cyclohexane).

^{1}H NMR (300 MHz, $CDCl_3$): δ = 7.94 (d, *J*=8.9 Hz, 1 H), 7.61 - 7.68 (m, 2 H), 7.51 - 7.59 (m, 8 H), 7.20 - 7.27 (m, 2 H), 7.09 (d, *J*=9.0 Hz, 1 H), 5.38 (br. s., 1 H), 2.69 (ttd, *J*=6.7, 3.5, 1.5 Hz, 1H), 0.81 - 0.90 (m, 2 H), 0.57 - 0.69 (m, 2 H) ppm.

^{13}C NMR (75 MHz, $CDCl_3$): δ = 158.4, 147.9, 137.8, 129.6, 127.5, 125.9, 123.7, 122.1, 109.6, 24.0, 8.0 ppm.

IR (ATR): ṽ = 3250 (w), 2890 (vw), 1750 (m), 1590 (m), 1482 (m), 1350 (m), 1000 (vw), 890 (vw), 850 (vw), 790 (vw), 700 (vw), 510 (vw) cm^{-1}

GC-HRMS (EI-QTOF): $[M]^+$calcd. 184.1000 for $C_{12}H_{12}N_2$; found: 184.0998.

*10.55.Synthesis of N-Cyclopropyl-2-methyl-5-benzoxazolamine (**9e**) [CAS 1509662-56-9]*

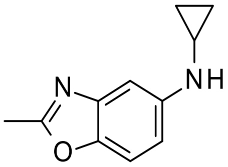

Following general procedure (GP G) and starting from Ethyl 4-Chlorobenzoate the title compound was obtained as a colorless oil (82.3 mg, 0.437 mmol, 87%). 0–60% (1% NEt_3 in ethyl acetate) in (1% NEt_3 in cyclohexane).

The NMR data are in agreement with those reported in literature.[40]

^{1}H NMR (300 MHz, $CDCl_3$): δ = 7.30 (s, 1 H), 7.15 (d, *J*=2.3 Hz, 1H), 6.72 (dd, *J*=8.7, 2.3 Hz, 1 H), 4.25 (br. s., 1 H), 2.60 - 2.65 (s, 3 H), 2.44 - 2.53 (m, 1 H), 0.75 - 0.84 (m, 2 H), 0.53 - 0.61 (m, 2 H) ppm.

^{13}C NMR (75 MHz, $CDCl_3$): δ = 164.0, 146.2, 144.5, 142.6, 111.3, 110.0, 102.3, 26.9, 25.9, 14.6, 7.4 ppm.

IR (ATR): ṽ = 3299 (m), 2976 (w), 1337 (m), 1292 (m), 1220 (m), 1178 (m), 1115 (m), 800 , 664 (m), 591 (w), 533(w), 436(w) cm^{-1}

GC-HRMS (EI-QTOF): $[M]^+$calcd. 188.0950 for $C_{11}H_{12}N_2O$; found: 188.0947.

*10.56.Synthesis of 4-Methoxy-N-(2,2,2-trifluoroethyl)benzenamine (**10a**) [CAS 62158-95-6]*

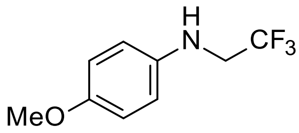

Following general procedure (GP H) and starting from 4-Chloranisol the title compound was obtained as a off white solid (89.0 mg, 0.434 mmol, 89%). 0–20% (1% NEt_3 ethyl acetate) in (1% NEt_3 in cyclohexane).

The NMR data are in agreement with those reported in literature.[41]

^{1}H NMR (400 MHz, $CDCl_3$): δ = 6.78 - 6.85 (m, 2 H), 6.63 - 6.71 (m, 2 H), 3.77 (s, 3 H), 3.67 - 3.74 (m, 2 H) pm.

^{13}C NMR (101 MHz, $CDCl_3$): δ = 153.2, 140.2, 126.5, 123.7, 114.9, 114.7, 55.7, 46.5 - 48.0 (m) 28.2 ppm.

^{19}F NMR (76 MHz, $CDCl_3$, $C_6H_4F_2$): δ = - 73.7 ppm.

10.57.Synthesis of 4-(4,4,5,5-tetramethyl-1,3,2-dioxaborolan-2-yl)-N-(2,2,2-trifluoroethyl)aniline

*(**10b**)*

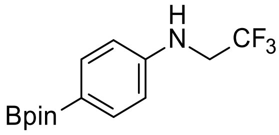

Following general procedure (GP H) and starting from 2-(4-Chlorophenyl)-4,4,5,5-tetramethyl-1,3,2-dioxaborolane the title compound was obtained as a off white solid (110 mg, 0.474 mmol, 95%). 0–40% (1% NEt_3 ethyl acetate) in (1% NEt_3 in cyclohexane).

^{1}H NMR (400 MHz, $CDCl_3$): δ = 7.62 - 7.74 (m, 2 H), 6.62 - 6.73 (m, 2 H), 4.15 (br s, 1 H), 3.74 - 3.91 (m, 2 H), 1.33 (s, 12 H) ppm.

^{13}C NMR (75 MHz, $CDCl_3$): δ = 148.7, 136.4, 126.7, 123.0, 112.1, 83.4, 43.2 - 47.3 (q), 24.8 ppm.

^{19}F NMR (76 MHz, $CDCl_3$, $C_6H_4F_2$): δ = - 73.7 ppm.

IR (ATR): ṽ = 3377 (m), 2981 (w), 2924 (w), 2854 (w), 1605 (s), 1353 (s), 1313 (s), 1136 (s), 1082 (s), 946 (m), 856 (m), 814 (m), 672 (s) cm^{-1}

HPLC-HRMS (ESI): $[M+H]^+$ calcd. 302.1534 for $C_{14}H_{19}BF_3NO_2$; found: 302.1534.

*10.58.Synthesis of N-(2,2,2-Trifluoroethyl)-2-benzothiazolamine (**10c**) [CAS 1036568-13-4]*

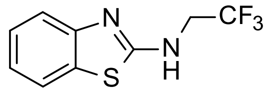

Following general procedure (GP H) and starting from 2-Chlorobenzothiazole the title compound was obtained as a off white solid (110 mg, 0.474 mmol, 95%). 0–60% (1% NEt_3 ethyl acetate) in (1% NEt_3 in cyclohexane).

^{1}H NMR (400 MHz, $CDCl_3$): δ = 8.60 (t, *J*=6.1 Hz, 1 H), 7.72 (d, *J*=7.4 Hz, 1 H), 7.46 (d, *J*=7.9 Hz, 1 H), 7.23 - 7.30 (m, 1 H), 7.05 - 7.13 (m, 1 H), 4.21 - 4.34 (m, 2 H) ppm.

^{13}C NMR (101 MHz, $CDCl_3$): δ = 166.1, 151.6, 130.6, 129.1, 126.3, 125.7, 123.5, 121.7, 121.2, 118.7, f44.2 (q) ppm.

^{19}F NMR (76 MHz, *DMSO-d*$_6$, $C_6H_4F_2$): δ = - 73.4 ppm.

IR (ATR): ṽ = 3240 (w), 3097 (m), 2916 (s), 1625 (s), 1575 (s), 1425 (s), 1395 (s), 1260 (s), 1113 (s), 932 (s), 884 (m), 753 (m), 723 (s), 674 (s), 523 (m), 433 (s) cm^{-1}

GC-HRMS (EI-QTOF): $[M]^+$ calcd. for $C_9H_7F_3N_2S$: 232.0282; found: 232.0282.

*10.59.Synthesis of N-(4-Methoxyphenyl)-2-thiazolamine (**11a**) [CAS 63615-92-9]*

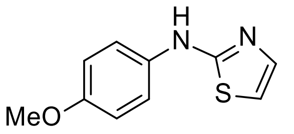

Following general procedure (GP I) and starting from 4-Bromanisol the title compound was obtained as a off white solid (100.8 mg, 0.489 mmol, 98%). 0–50% ethyl acetate in cyclohexane.

The NMR data are in agreement with those reported in literature.[42]

^{1}H NMR (300 MHz, $CDCl_3$): δ = 8.61 (br. s, 1 H), 7.28 - 7.34 (m, 2 H), 7.24 (d, *J*=3.7 Hz, 1 H), 6.89 - 6.96 (m, 2 H), 6.54 (d, *J*=3.7 Hz, 1 H), 3.82 (s, 3 H) ppm.

^{13}C NMR (75 MHz, , $CDCl_3$): δ = 168.1, 156.2, 138.6, 134.1, 121.6, 114.7, 106.6, 55.5 ppm.

IR (ATR): ṽ = 3262 (w), 2981 (w), 2157 (w), 1596 (w), 1155 (w), 1089 (m), 914 (w), 661 (w), 561 (w), 422 (w) cm^{-1}

GC-HRMS (EI-QTOF): [M]+calcd. 206.0514 for $C_{10}H_{10}N_2OS$; found: 206.0519.

*10.60.Synthesis of N-[4-(Trifluoromethyl)phenyl]-2-thiazolamine (**11b**) [CAS 1148107-42-9]*

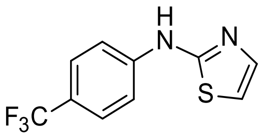

Following general procedure (GP I) and starting from 4-Bromobenzotrifluoride the title compound was obtained as a off white solid (112.6 mg, 0.4610 mmol, 92%). 0–40% ethyl acetate in cyclohexane.

^{1}H NMR (300 MHz, $CDCl_3$): δ = 7.58 - 7.64 (m, 2 H), 7.46 - 7.51 (m, 2 H), 7.37 (d, *J*=3.6 Hz, 1 H), 6.77 (d, *J*=3.7 Hz, 1 H) ppm.

^{13}C NMR (75 MHz, $CDCl_3$): δ = 164.26, 143.4, 138.4, 126.7 (q)

, 123.9, 116.7, 108.7 ppm.

^{19}F NMR (76 MHz, $CDCl_3$, $C_6H_4F_2$): -63.2 ppm.

IR (ATR): ṽ = 2952 (w), 2087 (vw), 1611 (m), 1499 (m), 1326 (m), 1100 (vs), 1060 (vs), 1007 (w), 874 (w), 599 (m), 503 (m), 423 (m) cm^{-1}

GC-HRMS (EI-QTOF): $[M]^+$calcd. 244.0282 for $C_{10}H_7F_3N_2S$; found: 244.0275.

*10.61.Synthesis of N-(4-Methylphenyl)-2-thiazolamine (**11c**) [CAS 112584-15-3]*

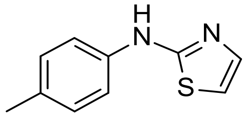

Following general procedure (GP I) and starting from 4-Bromotoluene the title compound was obtained as a off white solid (83.9 mg, 0.441 mmol, 88%). 0–40% ethyl acetate in cyclohexane.

The NMR data are in agreement with those reported in literature.[43]

^{1}H NMR (300 MHz, $CDCl_3$): δ = 7.25 - 7.31 (m, 3 H), 7.16 - 7.22 (m, 2 H), 6.59 (d, *J*=3.7 Hz, 1 H), 2.35 (s, 3 H) ppm.

^{13}C NMR (75 MHz, $CDCl_3$): δ = 167.1, 138.2, 132.7, 130.0, 118.7, 106.5, 20.8 ppm.

IR (ATR): ṽ = 2911 (w), 2228 (vw), 1436 (m), 1407 (m), 1385 (m), 1258 (m), 1057 (vs), 950 (m), 682 (m), 495 (vs) cm^{-1}

GC-HRMS (EI-QTOF): $[M]^+$calcd. 190.0565 for $C_{10}H_{10}N_2S$; found: 190.0560.

*10.62.Synthesis of 4-(2-Thiazolylamino)benzonitrile (**11d**) [CAS 959928-72-4]*

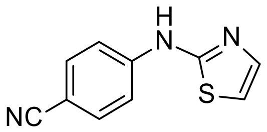

Following general procedure (GP I) and starting from 4-Brombenzonitrilthe title compound was obtained as a off white solid (87.0 mg, 0.432 mmol, 87%). 0–60% ethyl acetate in cyclohexane.

The NMR data are in agreement with those reported in literature.[44]

^{1}H NMR (300 MHz, *DMSO-d*$_6$): δ = 10.74 (s, 1 H), 7.69 - 7.83 (m, 4 H), 7.35 (d, *J*=3.7 Hz, 1 H), 7.07 (d, *J*=3.7 Hz, 1 H) ppm.

^{13}C NMR (75 MHz, *DMSO-d*$_6$): δ = 162.6, 145.0, 139.0, 133.4, 119.5, 116.6, 110.4, 101.9 ppm.

IR (ATR): ṽ = 3422 (w), 3243 (w), 2219 (m), 1550 (m), 1427 (m), 1330 (m), 1162 (vs), 718 (vs), 507 (vs), 472 (w) cm^{-1}

GC-HRMS (EI-QTOF): [M]$^{+}$calcd. 201.0361for $C_{10}H_7N_3S$; found: 201.0355.

*10.63.Synthesis of N-2-Thiazolyl-3-pyridinamine (**11e**) [CAS 58061-59-9]*

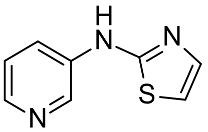

Following general procedure (GP I) and starting from 3-Brompyridine the title compound was obtained as a off white solid (83.9 mg, 0.441 mmol, 88%). 0–40% ethyl acetate in cyclohexane.

^{1}H NMR (300 MHz, *DMSO-d*$_6$): δ = 10.38 (s, 1 H), 8.74 (d, *J*=2.7 Hz, 1 H), 8.09 - 8.26 (m, 2 H), 7.24 - 7.38 (m, 2 H), 6.97 (d, *J*=3.7 Hz, 1 H) ppm.

^{13}C NMR (75 MHz, *DMSO-d*$_6$): δ = 163.4, 141.8, 138.9, 138.7, 137.9, 123.7, 123.0, 109.4 ppm.

IR (ATR): ṽ = 3653 (w), 2980 ,, 2885 (w), 2358 (m), 1627 (w), 1590 (w), 1382 (m), 954 (m), 805 (w), 632 (w), 605 (w) cm^{-1}

GC-HRMS (EI-QTOF): [M]$^{+}$calcd. 177.0361 for $C_8H_7N_3S$; found: 177.0354.

*10.64.Synthesis of 4-Methoxydiphenylamin (**12a**) [CAS 1208-86-2]*

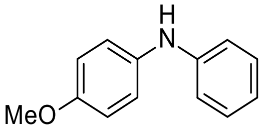

Following the general procedure (GP J), starting from 4-Fluoroanisole, a yield of 92% was determined by GC analysis for the title compound using a five-point calibrated response factor. The reaction was carried out with the addition of *n*-hexadecane (30 µL) as an internal standard.

*10.65.Synthesis of N-(4-Methoxyphenyl)-4-(trifluoromethyl)benzenamine (**13a**) [CAS 53451-88-0]*

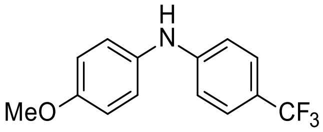

Following general procedure (GP K) and starting from 4-Methoxyphenyl trifluoromethanesulfonate the title compound was obtained as white solid (123.1 mg, 0.46 mmol, 92%). 0–10% ethyl acetate in cyclohexane.

The NMR data are in agreement with those reported in literature.[45]

^{1}H NMR (300 MHz, $CDCl_3$): δ = 7.43 (d, *J*=8.6 Hz, 2 H), 7.10 - 7.18 (m, 2 H), 6.84 - 6.96 (m, 4 H), 5.73 (br. s., 1 H), 3.84 (s, 3 H) ppm.

^{13}C NMR (75 MHz, $CDCl_3$): 126.6, 126.3 - 126.9 (m), 124.3, 122.9, 120.0 - 120.8 (m), 114.8, 113.7, 55.5 ppm.

^{19}F NMR (76 MHz, $CDCl_3$, $C_6H_4F_2$): -62.6 ppm.

IR (ATR): ṽ = 3392 (m), 3026 (w), 2964 (w), 2843 (w), 1609 (m), 1510 (s), 1318 (s), 1098 (s), 810 (s), 657 (m). 510 (m) cm^{-1}.

*10.66.Synthesis of 3-(1H-1,2,3-Triazol-1-yl)benzonitrile (**14a**) [CAS 85862-70-0]*

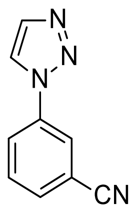

Following general procedure (GP L) and starting from 3-Bromobenzonitrile the title compound was obtained as white solid (78.1 mg, 0.459 mmol, 92%). 0–50% ethyl acetate in cyclohexane.

The NMR data are in agreement with those reported in literature.[46]

^{1}H NMR (400 MHz, $CDCl_3$): δ = 8.43 (s, 1 H), 8.36 (dt, *J*=7.5, 2.0 Hz, 1 H), 7.87 (s, 2 H), 7.59 - 7.67 (m, 2 H) ppm.

^{13}C NMR (101 MHz, $CDCl_3$): δ = 136.4, 130.8, 130.3, 122.9, 122.3, 117.9, 113.6 ppm.

IR (ATR): ṽ = 3137 (w), 3122 (w), 2233 (s), 1584 (s), 1478 (s), 1442 (s), 1408 (s), 1375 (s), 1185 (m), 953 (s), 835 (s), 800 (s), 672 (m), 622 (m) cm^{-1}

GC-HRMS (EI-QTOF): $[M]^+$calcd. 170.0593 for $C_9H_6N_4$; found: 170.0594.

*10.67.Synthesis of 1-(2-Fluorophenyl)-4-methyl-1H-imidazole (**15a**) [CAS 1351990-67-4]*

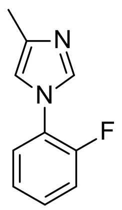

Following general procedure (GP M) and starting from 1-Bromo-2-fluorobenzene the title compound was obtained as white solid (86.2 mg, 0.489 mmol, 98%). 0–50% ethyl acetate in cyclohexane.

^{1}H NMR (400 MHz, $CDCl_3$): δ = 7.73 (t, *J*=1.6 Hz, 1 H), 7.30 - 7.41 (m, 2 H), 7.21 - 7.30 (m, 2 H), 6.99 (dt, *J*=1.8, 1.1 Hz, 1 H), 2.30 - 2.33 (m, 3 H) ppm.

^{13}C NMR (101 MHz, $CDCl_3$): δ = 156.1, 153.6, 138.9, 136.2, 136.2, 128.5, 128.5, 125.7, 125.0, 125.0, 124.8, 117.3, 117.1, 115.9, 115.8 , 77.2, 13.6 ppm.

^{19}F NMR (76 MHz, $CDCl_3$, $C_6H_4F_2$): - 116.4 ppm.

IR (ATR): ṽ = 3103 (vw), 3001 (w), 2983 (w), 2944 (w), 1619 (m), 1515 (s), 1440 (w), 1323 (s), 1110 (m), 1063 (s), 970 (m), 813 (s), 748 (s). 625 (s) cm^{-1}

GC-HRMS (EI-QTOF): $[M]^+$calcd. 176.0750 for $C_{10}H_9FN_2$; found: 176.0750.

*10.68.Synthesis of Anisonitrile (**16a**) [CAS 874-90-8]*

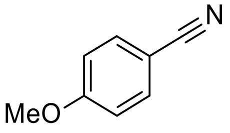

Following general procedure (GP N) and starting from 4-Chloranisol title compound was obtained as a off-white solid (112.7 mg, 0.846 mmol, 85%). 0–40% ethyl acetate in cyclohexane.

The NMR data are in agreement with those reported in literature.[47]

^{1}H NMR (400 MHz, $CDCl_3$): δ = 7.55 - 7.62 (m, 2 H), 6.96 (d, J=8.5 Hz, 2 H), 3.86 (s, 3 H) ppm.

^{13}C NMR (101 MHz, $CDCl_3$): δ = 162.8 , 133.9 , 119.2 , 114.7 , 103.9 , 55.5 ppm.

IR (ATR): ṽ = 2976 (w), 2941 (m), 2969 (w), 2216 (m), 1603 (s), 1507 (s), 1173 (m), 1021 (m), 826 (w), 681(m), 544 (w) cm^{-1}.

GC-HRMS (EI-QTOF): **)**: $[M]^+$calcd. 133.0528 for C_8H_7NO; found: 133.0530.

*10.69.Synthesis of 4-(Trifluoromethyl)benzonitrile (**16b**) [CAS 455-18-5]*

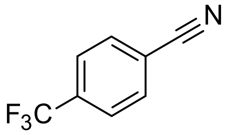

Following general procedure (GP N) and starting from 4-Chlorobenzotrifluoride title compound was obtained as a off-white solid (145.0 mg, 0.847 mmol, 85%). 0–30% ethyl acetate in cyclohexane.

The NMR data are in agreement with those reported in literature.[48]

^{1}H NMR (300 MHz, $CDCl_3$): δ = 7.79 (q, *J*=8.5 Hz, 4 H) ppm.

^{13}C NMR (75 MHz, $CDCl_3$): δ = 34.8, 134.3, 132.7, 126.1 (q), 124.8, 121.2, 117.4, 116.0 ppm.

^{19}F NMR (76 MHz, $CDCl_3$, $C_6H_4F_2$): δ = -64.9 ppm.

IR (ATR): ṽ = 2236 (m), 1622 (m), 1414 (w), 1106 (w), 1058 (w), 1017 (w), 779 (m), 596 (m), 546 (m) cm^{-1}

GC-HRMS (EI-QTOF): $[M]^+$calcd. 171.0296 for $C_8H_4F_3N$; found: 171.0292.

*10.70.Synthesis of Ethyl 4-cyanobenzoate (**16c**) [CAS 7153-22-2]*

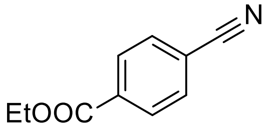

Following general procedure (GP N) and starting from ethyl 4-Chlorobenzoate the title compound was obtained as a off-white solid (167.1 mg, 0.954 mmol, 95%). 0–30% ethyl acetate in cyclohexane.

The NMR data are in agreement with those reported in literature.[49]

^{1}H NMR (300 MHz, $CDCl_3$): δ = 8.15 (d, *J*=8.3 Hz, 2 H), 7.75 (d, *J*=8.3 Hz, 2 H), 4.42 (q, *J*=7.2 Hz, 2 H), 1.42 (t, *J*=7.2 Hz, 3 H) ppm.

^{13}C NMR (75 MHz, $CDCl_3$): δ = 164.9, 134.3, 132.1, 130.0, 118.0, 116.2, 61.8, 14.2 ppm.

IR (ATR): **)**: ṽ = 3262 (m), 3066 (w), 2980 (m), 2883 (m), 2158 (m), 1965 (m), 1596 (s), 1090 (m), 968 (m) cm^{-1}

GC-HRMS (EI-QTOF): $[M]^+$calcd. 175.0633 for $C_{10}H_9NO_2$; found: 175.0635.

*10.71.Synthesis of p-Tolunitrile (**16d**) [CAS 104-85-8]*

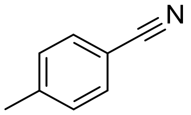

Following general procedure (GP N) and starting from 4-chlorotoluene title compound was obtained as a off-white solid (145.0 mg, 0.847 mmol, 85%). 0–30% ethyl acetate in cyclohexane.

The NMR data are in agreement with those reported in literature.[50]

^{1}H NMR (300 MHz, $CDCl_3$): δ = 7.52 (d, *J*=8.2 Hz, 2 H), 7.23 - 7.29 (m, 2 H), 2.41 (s, 3 H) ppm.

^{13}C NMR (75 MHz, $CDCl_3$): δ = 143.7, 132.0, 129.8, 119.1, 109.3, 21.8 ppm.

IR (ATR): ṽ = 2925 (w), 2227 (ml, 1177 (m), 1607 (s), 1508 (m), 814 (w) cm^{-1}

GC-HRMS (EI-QTOF): $[M]^+$calcd. 117.0579 for C_8H_7N; found: 117.0577.

*10.72.Synthesis of 4-Acetylbenzonitril (**16e**) [CAS 1443-80-7]*

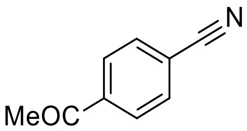

Following general procedure (GP N) and starting from 4-Chloroacetophenonethe title compound was obtained as a off-white solid (129.1 mg, 0.889 mmol, 89%). 0–30% ethyl acetate in cyclohexane.

The NMR data are in agreement with those reported in literature.[49]

^{1}H NMR (300 MHz, $CDCl_3$): δ = 8.04 (dd, J=8.4, 1.1 Hz, 2 H), 7.74 - 7.81 (m, 2 H), 2.64 (s, 3 H) ppm.

^{13}C NMR (75 MHz, $CDCl_3$): δ = 196.5, 139.8, 132.4, 128.6, 117.8, 116.3, 26.7 ppm.

IR (ATR): ṽ = 3262 (m), 3066 (w), 2980 (w), 2883 (m), 2158 (m), 1965 (m), 1596 (m), 1090 (m), 968 (m) cm^{-1}

GC-HRMS (EI-QTOF): $[M]^+$calcd. 145.0531 for C_9H_7NO; found: 145.0528.

*10.73.Synthesis of 4-Hydroxyanisol (**17a**) [CAS 150-76-5]*

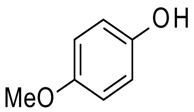

Following the general procedure (GP N), starting from 4-Chloranisol, a yield of 92% was determined by GC analysis for the title compound using a five-point calibrated response factor. The reaction was carried out with the addition of *n*-hexadecane (20 µL) as an internal standard.

*10.74.Synthesis of 1-Butoxy-4-methoxybenzene (**18a**) [CAS 20743-95-7]*

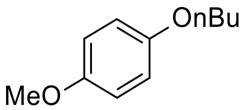

Following the general procedure (GP P), starting from 4-chloroanisole, a yield of 95% was determined by GC analysis for the title compound using a five-point calibrated response factor. The reaction was carried out with the addition of *n*-hexadecane (20 µL) as an internal standard.

*10.75.Synthesis of 1-Butoxy-4-trifluoromethylbenzene (**18b**) [CAS 944584-50-3]*

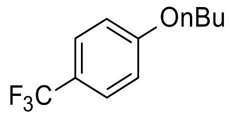

Following general procedure (GP P) and starting from 4-Chlorobenzotrifluoride the title compound was obtained as a colorless liquid (93.3 mg, 0.428 mmol, 86%). 0–10% ethyl acetate in cyclohexane.

The NMR data are in agreement with those reported in literature.[51]

^{1}H NMR (300 MHz, $CDCl_3$): δ = 7.55 (d, *J*=8.6 Hz, 2 H), 6.97 (d, *J*=8.6 Hz, 2 H), 4.02 (t, *J*=6.5 Hz, 2 H), 1.74 - 1.87 (m, 2 H), 1.48 - 1.61 (m, 2 H), 1.01 (t, *J*=7.3 Hz, 3 H) ppm.

^{13}C NMR (75 MHz, $CDCl_3$): δ = 161.6, 126.83 (q), 126.3, 122.7 (m), 122.4, 114.4, 67.9, 32.0, 31.1, 29.7, 29.7, 29.4, 22.7, 19.2, 14.1, 13.7 ppm.

^{19}F NMR (76 MHz, $CDCl_3$, $C_6H_4F_2$): δ = -62.8 ppm.

IR (ATR): ṽ = 3248 (w), 3192 (w), 3055 (w), 1660 (m), 1161 (m), 1110 (m), 1093 (m) cm^{-1}

GC-HRMS (EI-QTOF): $[M]^+$calcd. 218.0919 for $C_{11}H_{13}F_3O$; found: 218.0918.

*10.76.Synthesis of 1-Butoxy-4-(methanesulfonyl)benzene (**18c**) [CAS 345222-98-2]*

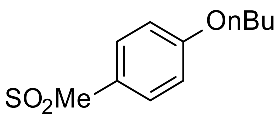

Following general procedure (GP P) and starting from 4-Chlorophenyl Methyl Sulfone the title compound was obtained as a yellow viscous liquid (99.1 mg, 0.434 mmol, 87%). 0–60% ethyl acetate in cyclohexane.

^{1}H NMR (300 MHz, $CDCl_3$): δ = 7.80 - 7.88 (m, 2 H), 6.96 - 7.04 (m, 2 H), 4.03 (t, *J*=6.5 Hz, 2 H), 3.02 (s, 3 H), 1.73 - 1.85 (m, 2 H), 1.44 - 1.55 (m, 2 H), 0.98 (t, *J*=7.4 Hz, 3 H) ppm.

^{13}C NMR (75 MHz, $CDCl_3$): δ = 163.3, 131.9, 129.5, 114.9, 68.2, 44.8, 31.0, 19.1, 13.7 ppm.

IR (ATR): ṽ = 1645 (m), 1618 (m), 1625 (m), 1281 (m), 1572 (w), 900 (w) cm^{-1}

GC-HRMS (EI-QTOF): $[M]^+$calcd. 228.0820 for $C_{11}H_{16}O_3S$; found: 228.0820.

*10.77.Synthesis of 3-Butoxyquinoline (**18d**) [CAS 1239478-93-3]*

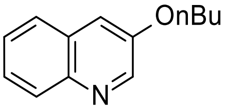

Following general procedure (GP P) and starting from 3-Chloroquinoline the title compound was obtained as a off white solid (78.8 mg, 0.392 mmol, 78%). 0–30% ethyl acetate in cyclohexane.

The NMR data are in agreement with those reported in literature.[52]

^{1}H NMR (300 MHz, $CDCl_3$): δ = 8.68 (d, *J*=2.8 Hz, 1 H), 8.05 (d, *J*=8.3 Hz, 1 H), 7.69 - 7.75 (m, 1 H), 7.47 - 7.59 (m, 2 H), 7.38 (d, *J*=2.8 Hz, 1 H), 4.10 (t, *J*=6.5 Hz, 2 H), 1.79 - 1.92 (m, 2 H), 1.56 (dq, *J*=15.0, 7.4 Hz, 2 H), 1.02 (t, *J*=7.4 Hz, 3 H) ppm.

^{13}C NMR (75 MHz, $CDCl_3$): δ = 152.5, 144.7, 143.3, 129.0, 128.8, 126.9, 126.5, 126.4, 112.7, 77.4, 76.6, 67.9, 31.0, 19.1, 13.7 ppm.

GC-HRMS (EI-QTOF): $[M]^+$calcd. 201.1154 for $C_{13}H_{15}NO$; found: 201.1158.

*10.78.Synthesis of 5-Butoxy-2-methylbenzothiazole (**18e**) [CAS 1351403-41-2]*

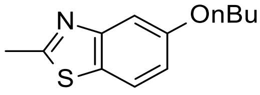

Following general procedure (GP P) and starting from 5-Chloro-2-methylbenzothiazole the title compound was obtained as a off white solid (98.9 mg, 0.447 mmol, 89%). 0–60% ethyl acetate in cyclohexane.

The NMR data are in agreement with those reported in literature.[52]

^{1}H NMR (300 MHz, $CDCl_3$): δ = 7.66 (d, J=8.8 Hz, 1 H), 7.45 (d, J=2.4 Hz, 1 H), 7.00 (dd, J=8.7, 2.5 Hz, 1 H), 4.04 (t, J=6.5 Hz, 2 H), 1.74 - 1.89 (m, 3 H), 1.47 - 1.62 (m, 2 H), 1.00 (t, J=7.4 Hz, 3 H) ppm.

^{13}C NMR (75 MHz, $CDCl_3$): δ = 168.2, 158.4, 154.3, 127.0, 121.5, 115.2, 105.8, 68.1, 31.2, 20.1, 19.3, 13.8 ppm.

IR (ATR): ṽ = 3656 (w), 3262 (m), 2981 (m), 2360 (m), 1597 (w), 1513 (w), 1245 (m), 1155 (m), 1089 (w), 835 (w), 435 (w) cm^{-1}

GC-HRMS (EI-QTOF): [M]+calcd. 221.0874 for $C_{12}H_{15}NOS$; found: 221.0876.

*10.79.Synthesis of 1,4-Dimethoxybenzene (**18f**) [CAS 150-78-7]*

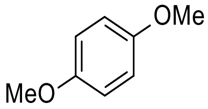

Following the general procedure (GP Q), starting from 4-Chloranisol, a yield of 95% was determined by GC analysis for the title compound using a five-point calibrated response factor. The reaction was carried out with the addition of *n*-hexadecane (20 µL) as an internal standard.

*10.80.Synthesis of 1-Fluoronaphthalene (**19a**) [CAS 321-38-0]*

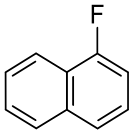

Following general procedure (GP R) and starting from Naphthalen-1-yl trifluoromethanesulfonate the title compound was obtained as white solid (51.2 mg, 0.350 mmol, 70%, analytical yield: 96%). 0–10% ethyl acetate in cyclohexane.

The NMR data are in agreement with those reported in literature.[53]

^{1}H NMR (400 MHz, $CDCl_3$): δ = 8.34 (d, *J*=8.3 Hz, 1 H), 7.93 (d, *J*=7.8 Hz, 1 H), 7.49 - 7.68 (m, 3 H), 7.38 (t, *J*=7.9 Hz, 1 H), 6.93 (d, *J*=7.3 Hz, 1 H) ppm.

^{13}C NMR (101 MHz, $CDCl_3$): δ = 153.5, 135.0, 127.8, 126.6, 126.5, 126.0, 125.9, 123.2, 122.0, 113.1ppm.

^{19}F NMR (76 MHz, $CDCl_3$, $C_6H_4F_2$): δ = -117.2 ppm.

IR (ATR): ṽ = 3059 (w), 1600 (m), 1391 (m), 1228 (m), 1074 (m), 1012 (w), 791 (m), 762 (w), 708 (m), 564 (w) cm^{-1}

GC-HRMS (EI-QTOF): $[M]^+$calcd. 146.0532 for $C_{10}H_7F$; found: 146.0531.

*10.81.Synthesis of 1-Fluor-4-nitrobenzol (**19b**) [CAS 350-46-9]*

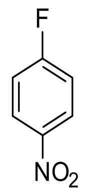

Following general procedure (GP R) and starting from 4-Nitrophenyl trifluoromethanesulfonate the title compound was obtained as white solid (29.5 mg, 0.209 mmol, 42%, analytical yield: 92%). 0–10% ethyl acetate in cyclohexane.

The NMR data are in agreement with those reported in literature.[54]

^{1}H NMR (400 MHz, $CDCl_3$): δ = 8.29 - 8.34 (m, 2 H), 7.14 - 7.22 (m, 2 H) ppm.

^{13}C NMR (101 MHz, $CDCl_3$): δ =160.6, 144.2, 126.2, 119.3 ppm.

^{19}F NMR (76 MHz, $CDCl_3$, $C_6H_4F_2$): -103.4 ppm

IR (ATR): ṽ = 3089 (w), 1592 (m), 1520 (m), 1493 (m), 1343 (m), 1228 (m), 1110 (m), 851 (m), 748 (w) cm^{-1}

GC-HRMS (EI-QTOF): $[M]^+$calcd. 141.0226 for $C_6H_4FNO_2$; found: 141.0226.

*10.82.Synthesis of 6-Fluoro-2-naphthaldehyde (**19c**) [CAS 721968-77-0]*

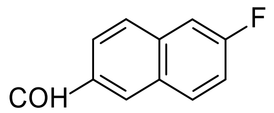

Following general procedure (GP R) and starting from 6-(Trifluoromethanesulfonyloxy)naphthalene-2-carboxaldehyde the title compound was obtained as white solid (33.8 mg, 0.194 mmol, 39%, analytical yield: 92%). 0–40% ethyl acetate in cyclohexane.

The NMR data are in agreement with those reported in literature.[55]

^{1}H NMR (400 MHz, $CDCl_3$): δ =10.16 (s, 1 H), 8.35 (s, 1 H), 7.96 - 8.06 (m, 2 H), 7.86 - 7.92 (m, 1 H), 7.54 (dd, J=9.5, 2.4 Hz, 1 H), 7.38 (td, J=8.6, 2.5 Hz, 1 H) ppm.

^{13}C NMR (101 MHz, $CDCl_3$): δ =191.8, 161.5 (d), 137.6 (d) 134.2 (d) 133.6 (d) 132.1 (d) 129.6, 128.4 (d) 124.0, 117.6 (d) 111.5 (d) ppm.

^{19}F NMR (76 MHz, $CDCl_3$, $C_6H_4F_2$): δ =-110.4 ppm

IR (ATR): ṽ = 3050 (w), 1684 (m), 1627 (s), 1473 (m), 1333 (m), 1255 (m), 1143 (m), 870 (m), 802 (m), 477 (w) cm^{-1}

GC-HRMS (EI-QTOF): $[M]^+$calcd. 174.0481 for $C_{11}H_7FO$; found: 174.0478.

*10.83.Synthesis of 7-Fluoro-2-phenylchromone (**19d**) [CAS 2331212-10-1]*

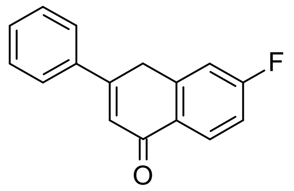

Following general procedure (GP R) and starting from 5-oxo-7-phenyl-5,8-dihydronaphthalen-2-yl trifluoromethanesulfonate the title compound was obtained as white solid (57.3 mg, 0.239 mmol, 48%, analytical yield: 71%). 0–50% ethyl acetate in cyclohexane.

The NMR data are in agreement with those reported in literature.[53]

^{1}H NMR (400 MHz, $CDCl_3$): δ = 8.29 - 8.33 (m, 1 H), 7.89 - 7.94 (m, 2 H), 7.49 - 7.58 (m, 3 H), 7.19 - 7.24 (m, 2 H), 6.84 (s, 1 H) ppm.

^{13}C NMR (101 MHz, $CDCl_3$): δ = 177.5, 163.6, 160.4, 157.5, 131.7, 131.5, 129.1, 128.1, 126.2, 120.6, 117.3, 107.7, 107.4, 100.0.

IR (ATR): ṽ = 3062 (w), 1637 (w), 1367 (m), 1166 (m), 1028 (m), 967 (m), 768 (m), 668 (m), 461 (m) cm^{-1}

*10.84.Synthesis of N-(1,1-Dimethylethyl)-2,6-dimethylbenzenamine (**20a**) [CAS 395116-77-5]*

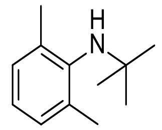

Following the general procedure (GP S), starting from 2-Chlor-m-xylol yield of 97% was determined by GC analysis for the title compound using a five-point calibrated response factor. The reaction was carried out with the addition of *n*-undecane (30 µL) as an internal standard.

*10.85.Synthesis of 2-(4-Methoxyphenyl)-1-phenylethanone (**21a**) [CAS 24845-40-7]*

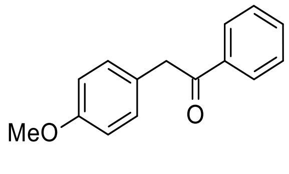

Following the general procedure (GP T), starting from 4-Chloranisol yield of 99% was determined by GC analysis for the title compound using a five-point calibrated response factor. The reaction was carried out with the addition of *n*-tetradecane (50 µL) as an internal standard.

*10.86. Synthesis of 2,2',4,6,6'-Pentamethyl-1,1'-biphenyl (**22a**) [CAS 76411-12-6]*

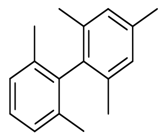

Following the general procedure (GP U), starting from 2-Chlor-m-xylol yield of 89% was determined by GC analysis for the title compound using a five-point calibrated response factor. The reaction was carried out with the addition of *n*-undecane (50 µL) as an internal standard.

11. References

[1] A. Gilbert, X. Bertrand, J.-F. Paquin, "Silver-Promoted Synthesis of 5-[(Pentafluorosulfanyl)methyl]-2-oxazolines" *Org. Lett.* **2018**, *20*, 7257–7260.

[2] X. Zhang, Z. Zhou, H. Xu, X. Xu, X. Yu, W. Yi, "Cobalt-Catalyzed Allylation of Amides with Styrenes Using DMSO as Both the Solvent and the α-Methylene Source" *Org. Lett.* **2019**, *21*, 7248–7253.

[3] F. Neese, "Software Update: The ORCA Program System—Version 6.0" *WIREs Comput. Mol. Sci.* **2025**, *15*, e70019.

[4] Y. Li **2023**, DOI 10.5281/ZENODO.7634466.

[5] S. M. Crawford, C. B. Lavery, M. Stradiotto, "BippyPhos: A Single Ligand With Unprecedented Scope in the Buchwald–Hartwig Amination of (Hetero)aryl Chlorides" *Chem. – Eur. J.* **2013**, *19*, 16760–16771.

[6] S. Pandey, S. Kumar, V. Singh, V. Srivastava, S. Singh, "Photocatalytic Approach Toward the Synthesis of Amides via S–C Cleavage: A Mild Approach" *J. Org. Chem.* **2025**, *90*, 6423–6433.

[7] R. P. S. Rajan, C. E. Hong, K. Park, S. Lee, "Photocatalytic Synthesis of Diaryl Amides via Direct Coupling of Methyl Arenes and Nitroarenes" *Org. Lett.* **2025**, *27*, 4580–4585.

[8] M. J. Cotnam, N. Martinek, M. Stradiotto, "Probing the Impact of Added $Al(OTf)_3$ on DalPhos/Nickel-Catalyzed *N* -Arylations of Amides with (Hetero)aryl Chlorides" *Eur. J. Org. Chem.* **2023**, *26*, e202301004.

[9] C. Liu, C. Ji, X. Hong, M. Szostak, "Palladium-Catalyzed Decarbonylative Borylation of Carboxylic Acids: Tuning Reaction Selectivity by Computation" *Angew. Chem. Int. Ed.* **2018**, *57*, 16721–16726.

[10] G. Pandey, S. Koley, R. Talukdar, P. K. Sahani, "Cross-Dehydrogenating Coupling of Aldehydes with Amines/R-OTBS Ethers by Visible-Light Photoredox Catalysis: Synthesis of Amides, Esters, and Ureas" *Org. Lett.* **2018**, *20*, 5861–5865.

[11] A. Klapars, X. Huang, S. L. Buchwald, "A General and Efficient Copper Catalyst for the Amidation of Aryl Halides" *J. Am. Chem. Soc.* **2002**, *124*, 7421–7428.

[12] X. Deng, F. Jiang, X. Wang, "Asymmetric Deoxygenative Functionalization of Secondary Amides with Vinylpyridines Enabled by a Triple Iridium-Photoredox-Chiral Phosphoric Acid System" *Org. Lett.* **2024**, *26*, 2483–2488.

[13] A. H. Dardir, P. R. Melvin, Ryan. M. Davis, N. Hazari, M. Mohadjer Beromi, "Rapidly Activating Pd-Precatalyst for Suzuki–Miyaura and Buchwald–Hartwig Couplings of Aryl Esters" *J. Org. Chem.* **2018**, *83*, 469–477.

[14] T. Roy, K. Mondal, P. Halder, A. Sengupta, P. Das, "CuF_2 /DTBP-Catalyzed Chan-Lam Coupling of Oxazolidinones with Arylboronic Acid Pinacol Ester: Scope and Application" *J. Org. Chem.* **2025**, *90*, 6219–6232.

[15] G. Song, Q. Li, D. Nong, J. Song, G. Li, C. Wang, J. Xiao, D. Xue, "Ni-Catalyzed Photochemical C−N Coupling of Amides with (Hetero)aryl Chlorides" *Chem. – Eur. J.* **2023**, *29*, e202300458.

[16] D. Sustac Roman, V. Poiret, G. Pelletier, A. B. Charette, "Direct Arylation of Imidazo[1,5- *a*]azines Through Ruthenium and Palladium Catalysis" *Eur. J. Org. Chem.* **2015**, *2015*, 67–71.

[17] M. Cortes-Salva, C. Garvin, J. C. Antilla, "Ligand-Free Copper-Catalyzed Arylation of Amidines" *J. Org. Chem.* **2011**, *76*, 1456–1459.

[18] W. Li, M. Xue, F. Xu, J. Tu, Y. Zhang, Q. Shen, "Synthesis, characterization of bridged bis(amidinate) lanthanide amides and their application as catalysts for addition of amines to nitriles for monosubstituted N-arylamidines" *Dalton Trans.* **2012**, *41*, 8252.

[19] A. D. Redhouse, R. J. Thompson, B. J. Wakefield, J. A. Wardell, "Synthesis of amidines and attempted synthesis of imidazoazines by reactions of lithiated β-aminoazines with nitriles" *Tetrahedron* **1992**, *48*, 7619–7628.

[20] L. Song, X. Tian, Z. Lv, E. Li, J. Wu, Y. Liu, W. Yu, J. Chang, "I_2 /KI-Mediated Oxidative N–N Bond Formation for the Synthesis of 1,5-Fused 1,2,4-Triazoles from *N* -Aryl Amidines" *J. Org. Chem.* **2015**, *80*, 7219–7225.

[21] E. Chetankumar, C. Srinivasulu, G. Periyasamy, V. V. Sureshbabu, "Tf_2 O-Promoted Synthesis of Ureas, Carbamates and Thiocarbamate via Lossen Rearrangement: A Mechanistic Insight" *Eur. J. Org. Chem.* **2024**, *27*, e202400028.

[22] J. Zhao, Z. Li, S. Yan, S. Xu, M.-A. Wang, B. Fu, Z. Zhang, "Pd/C Catalyzed Carbonylation of Azides in the Presence of Amines" *Org. Lett.* **2016**, *18*, 1736–1739.

[23] B. J. Kotecki, D. P. Fernando, A. R. Haight, K. A. Lukin, "A General Method for the Synthesis of Unsymmetrically Substituted Ureas via Palladium-Catalyzed Amidation" *Org. Lett.* **2009**, *11*, 947–950.

[24] J. Bao, D. Kuik, G. K. Tranmer, "An efficient one-pot synthesis of N,N′-disubstituted phenylureas and N-aryl carbamates using hydroxylamine-O-sulfonic acid" *Tetrahedron* **2018**, *74*, 5546–5553.

[25] F. Du, Q. Zhou, Y. Fu, H. Zhao, Y. Chen, G. Chen, "*tert* -Butyl(3-cyano-4,6-dimethylpyridin-2-yl)carbonate as a green and chemoselective *N-tert* -butoxycarbonylation reagent" *New J. Chem.* **2019**, *43*, 6549–6554.

[26] L. R. Reddy, S. Kotturi, Y. Waman, V. Ravinder Reddy, C. Patel, A. Kobarne, S. Kuttappan, "*N* -Arylation of Carbamates through Photosensitized Nickel Catalysis" *J. Org. Chem.* **2018**, *83*, 13854–13860.

[27] F. Ma, X. Xie, L. Zhang, Z. Peng, L. Ding, L. Fu, Z. Zhang, "Palladium-Catalyzed Amidation of Aryl Halides Using 2-Dialkylphosphino-2′-alkoxyl-1,1′-binaphthyl as Ligands" *J. Org. Chem.* **2012**, *77*, 5279–5285.
[28] M. Krumb, L. M. Kammer, R. Forster, C. Grundke, T. Opatz, "Visible-Light-Induced Cleavage of C−S Bonds in Thioacetals and Thioketals with Iodine as a Photocatalyst" *ChemPhotoChem* **2020**, *4*, 101–104.
[29] M. P. Wiesenfeldt, T. Knecht, C. Schlepphorst, F. Glorius, "Silylarene Hydrogenation: A Strategic Approach that Enables Direct Access to Versatile Silylated Saturated Carbo- and Heterocycles" *Angew. Chem. Int. Ed.* **2018**, *57*, 8297–8300.
[30] D. Yoo, T. Hasegawa, M. Ashizawa, T. Kawamoto, H. Masunaga, T. Hikima, H. Matsumoto, T. Mori, "N-Unsubstituted thienoisoindigos: preparation, molecular packing and ambipolar organic field-effect transistors" *J. Mater. Chem. C* **2017**, *5*, 2509–2512.
[31] E. Torti, S. Protti, D. Merli, D. Dondi, M. Fagnoni, "Photochemistry of *N* -Arylsulfonimides: An Easily Available Class of Nonionic Photoacid Generators (PAGs)" *Chem. – Eur. J.* **2016**, *22*, 16998–17005.
[32] Y.-Y. Liu, D. Liang, L.-Q. Lu, W.-J. Xiao, "Practical heterogeneous photoredox/nickel dual catalysis for C–N and C–O coupling reactions" *Chem. Commun.* **2019**, *55*, 4853–4856.
[33] F. Sánchez-Cantalejo, J. D. Priest, P. W. Davies, "A Gold Carbene Manifold to Prepare Fused γ-Lactams by Oxidative Cyclisation of Ynamides" *Chem. – Eur. J.* **2018**, *24*, 17215–17219.
[34] W. Chen, W. Yang, R. Wu, D. Yang, "Water-promoted synthesis of fused bicyclic triazolines and naphthols from oxa(aza)bicyclic alkenes and transformation *via* a novel ring-opening/rearrangement reaction" *Green Chem.* **2018**, *20*, 2512–2518.
[35] B. Xue, J. Shen, S. Manna, A. Doppiu, L. J. Gooßen, "Selective Monoarylation of Ammonium Triflate with Aryl Chlorides Catalyzed by [Pd(β-MeNAP)Br]$_2$ and AdBrettPhos" *Adv. Synth. Catal.* **2023**, *365*, 3473–3477.
[36] C. W. Cheung, D. S. Surry, S. L. Buchwald, "Mild and Highly Selective Palladium-Catalyzed Monoarylation of Ammonia Enabled by the Use of Bulky Biarylphosphine Ligands and Palladacycle Precatalysts" *Org. Lett.* **2013**, *15*, 3734–3737.
[37] S. Maity, M. Zhu, R. S. Shinabery, N. Zheng, "Intermolecular [3+2] Cycloaddition of Cyclopropylamines with Olefins by Visible-Light Photocatalysis" *Angew. Chem. Int. Ed.* **2012**, *51*, 222–226.
[38] H. Luo, G. Wang, Y. Feng, W. Zheng, L. Kong, Y. Ma, S. Matsunaga, L. Lin, "Photoinduced Nickel-Catalyzed Carbon–Heteroatom Coupling**" *Chem. – Eur. J.* **2023**, *29*, e202202385.
[39] P. Hong, X. Zhu, X. Lai, Z. Gong, M. Huang, Y. Wan, "Room-Temperature CuI-Catalyzed *N* -Arylation of Cyclopropylamine" *J. Org. Chem.* **2024**, *89*, 57–67.
[40] N. Kaiser, L. Räkow, J. Handelmann, V. H. Gessner, "Palladium-Catalyzed Monoarylation of Cyclopropylamine" *J. Org. Chem.* **2025**, *90*, 12153–12162.
[41] A. T. Brusoe, J. F. Hartwig, "Palladium-Catalyzed Arylation of Fluoroalkylamines" *J. Am. Chem. Soc.* **2015**, *137*, 8460–8468.
[42] D. Li, X. Lan, A. Song, Md. M. Rahman, C. Xu, F. Huang, R. Szostak, M. Szostak, F. Liu, "Buchwald-Hartwig Amination of Coordinating Heterocycles Enabled by Large-but-Flexible Pd-BIAN-NHC Catalysts**" *Chem. – Eur. J.* **2022**, *28*, e202103341.
[43] M. A. McGowan, C. Z. McAvoy, S. L. Buchwald, "Palladium-Catalyzed N-Monoarylation of Amidines and a One-Pot Synthesis of Quinazoline Derivatives" *Org. Lett.* **2012**, *14*, 3800–3803.
[44] J. S. K. Clark, M. J. Ferguson, R. McDonald, M. Stradiotto, "PAd2-DalPhos Enables the Nickel-Catalyzed C–N Cross-Coupling of Primary Heteroarylamines and (Hetero)aryl Chlorides" *Angew. Chem. Int. Ed.* **2019**, *58*, 6391–6395.
[45] T. Seo, K. Kubota, H. Ito, "Dual Nickel(II)/Mechanoredox Catalysis: Mechanical-Force-Driven Aryl-Amination Reactions Using Ball Milling and Piezoelectric Materials" *Angew. Chem. Int. Ed.* **2023**, *62*, e202311531.
[46] L. Wu, B. Yan, G. Yang, Y. Chen, "Green synthesis of 1-monosubstituted 1,2,3-triazoles via 'click chemistry' in water" *hc* **2013**, *19*, 397–400.
[47] T. D. Senecal, W. Shu, S. L. Buchwald, "A General, Practical Palladium-Catalyzed Cyanation of (Hetero)Aryl Chlorides and Bromides" *Angew. Chem. Int. Ed.* **2013**, *52*, 10035–10039.
[48] J. Zheng, J.-H. Lin, X.-Y. Deng, J.-C. Xiao, "1,8-Diazabicyclo[5.4.0]undec-7-ene (DBU)-Promoted Decomposition of Difluorocarbene and the Subsequent Trifluoromethylation" *Org. Lett.* **2015**, *17*, 532–535.
[49] D. T. Cohen, S. L. Buchwald, "Mild Palladium-Catalyzed Cyanation of (Hetero)aryl Halides and Triflates in Aqueous Media" *Org. Lett.* **2015**, *17*, 202–205.
[50] C. Zhu, H. Liu, Y. Wu, R. Meng, L. Ma, X. Wang, C. Wang, "Aerobic oxidative dehydrogenation of amines to nitriles catalyzed by Co@CsETS-10 catalyst" *Catal. Sci. Technol.* **2025**, *15*, 2670–2676.
[51] L. Yang, H. Lu, C. Lai, G. Li, W. Zhang, R. Cao, F. Liu, C. Wang, J. Xiao, D. Xue, "Light-Promoted Nickel Catalysis: Etherification of Aryl Electrophiles with Alcohols Catalyzed by a Ni^{II} -Aryl Complex" *Angew. Chem. Int. Ed.* **2020**, *59*, 12714–12719.

[52] X. Wu, B. P. Fors, S. L. Buchwald, “A Single Phosphine Ligand Allows Palladium-Catalyzed Intermolecular C⍰O Bond Formation with Secondary and Primary Alcohols” *Angew. Chem. Int. Ed.* **2011**, *50*, 9943–9947.
[53] D. A. Watson, M. Su, G. Teverovskiy, Y. Zhang, J. García-Fortanet, T. Kinzel, S. L. Buchwald, “Formation of ArF from LPdAr(F): Catalytic Conversion of Aryl Triflates to Aryl Fluorides” *Science* **2009**, *325*, 1661–1664.
[54] R. Calvo, K. Zhang, A. Passera, D. Katayev, “Facile access to nitroarenes and nitroheteroarenes using N-nitrosaccharin” *Nat. Commun.* **2019**, *10*, 3410.
[55] K. J. Schwarz, C. Yang, J. W. B. Fyfe, T. N. Snaddon, “Enantioselective α-Benzylation of Acyclic Esters Using π-Extended Electrophiles” *Angew. Chem. Int. Ed.* **2018**, *57*, 12102–12105.

12. Spectra

[Pd(MeNAP)TFA]2

^{1}H NMR (300 MHz, DMSO-d^6):

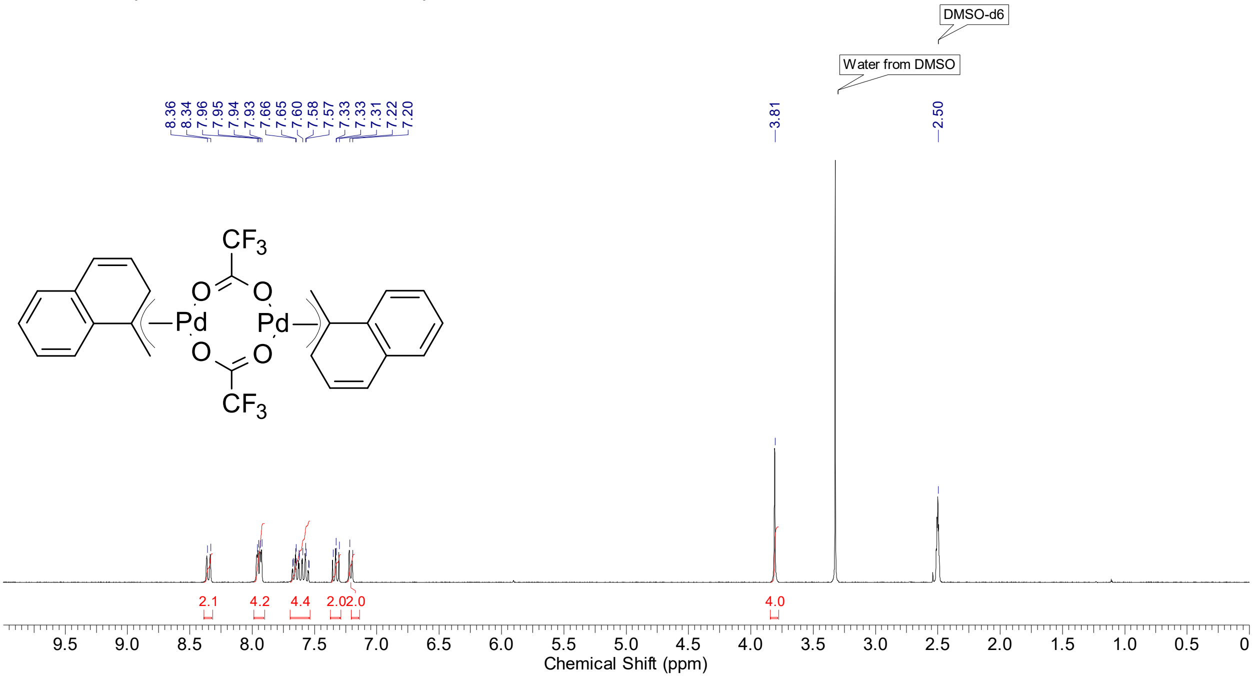

^{13}C NMR (75 MHz, DMSO-d^6):

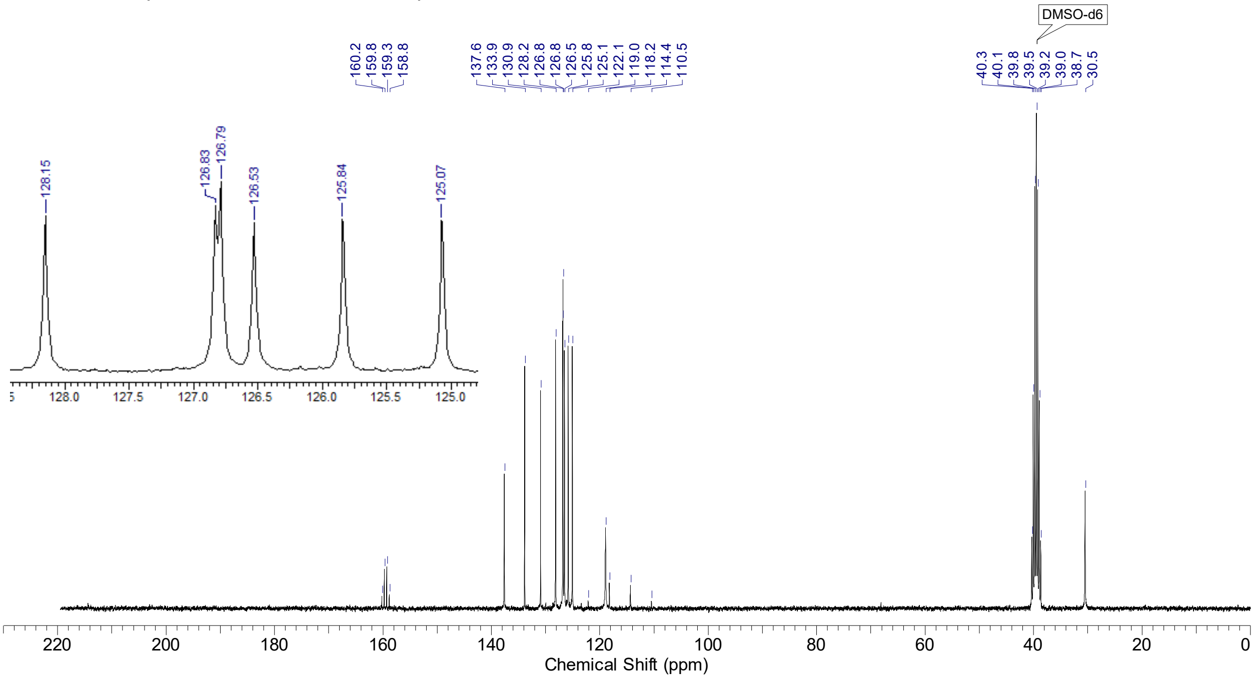

^{19}F NMR (76 MHz, DMSO-d_6, $C_6H_4F_2$):

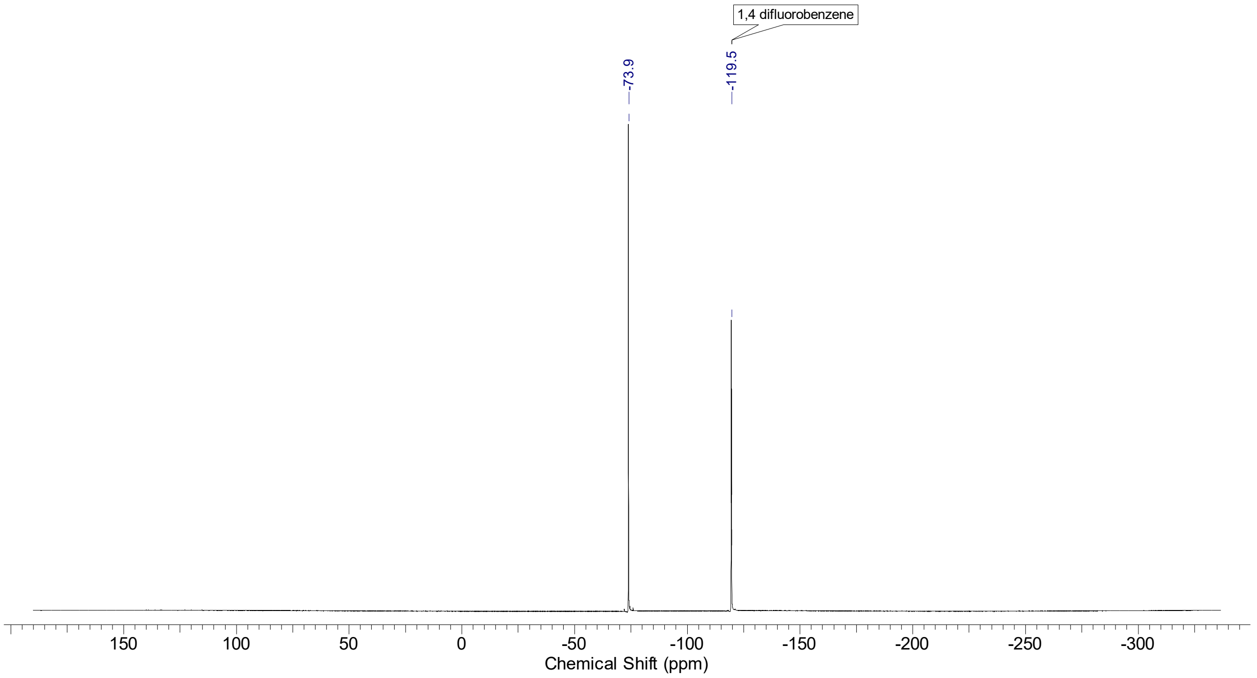

<u>[Pd(MeNAP)OTF]$_2$</u>

^{1}H NMR (300 MHz, THF-d^8):

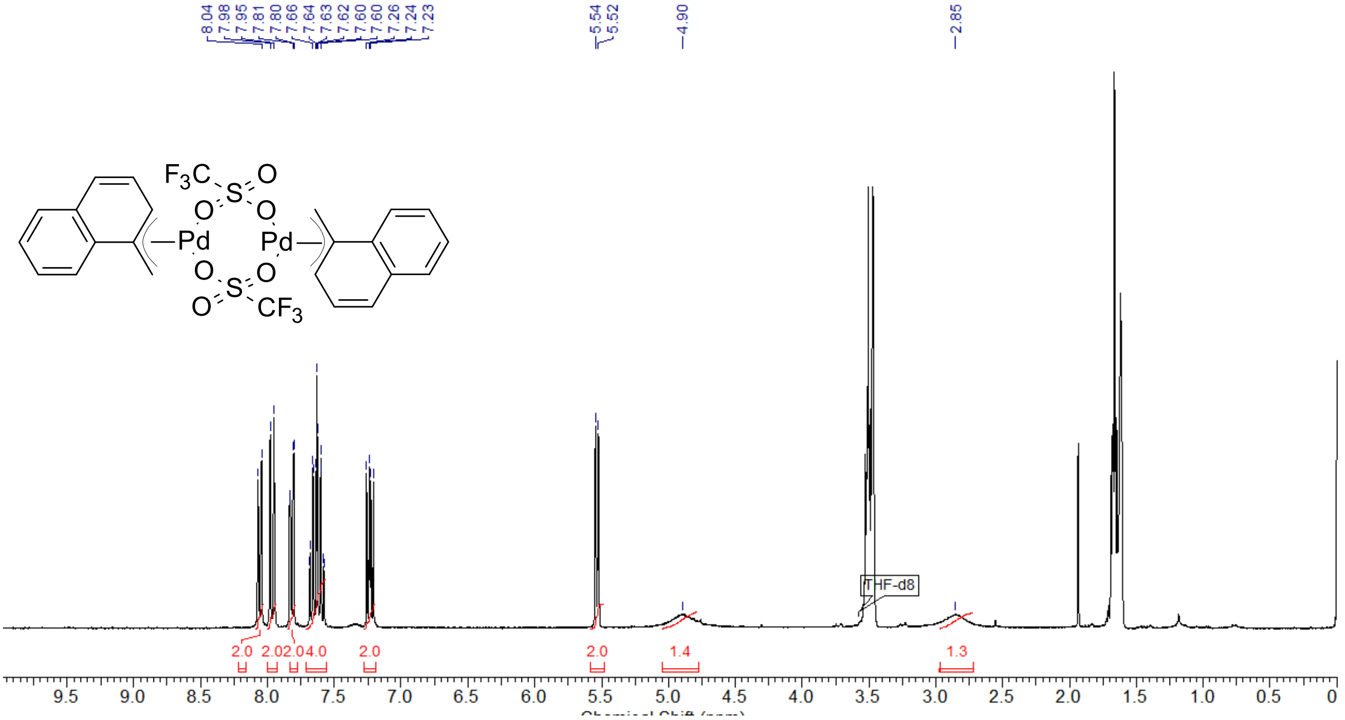

^{13}C NMR (75 MHz, THF-d^8):

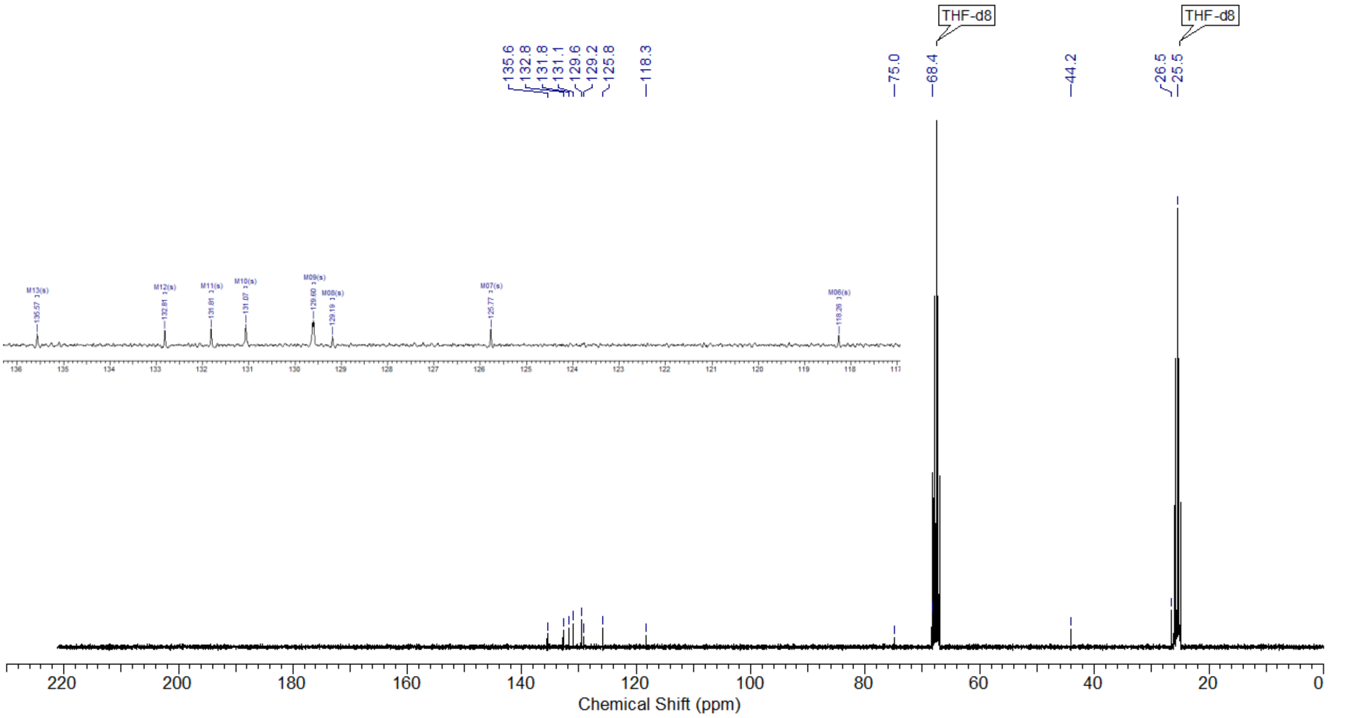

[Pd(MeNAP)OTs]$_2$

^{1}H NMR (300 MHz, DCM-d^2):

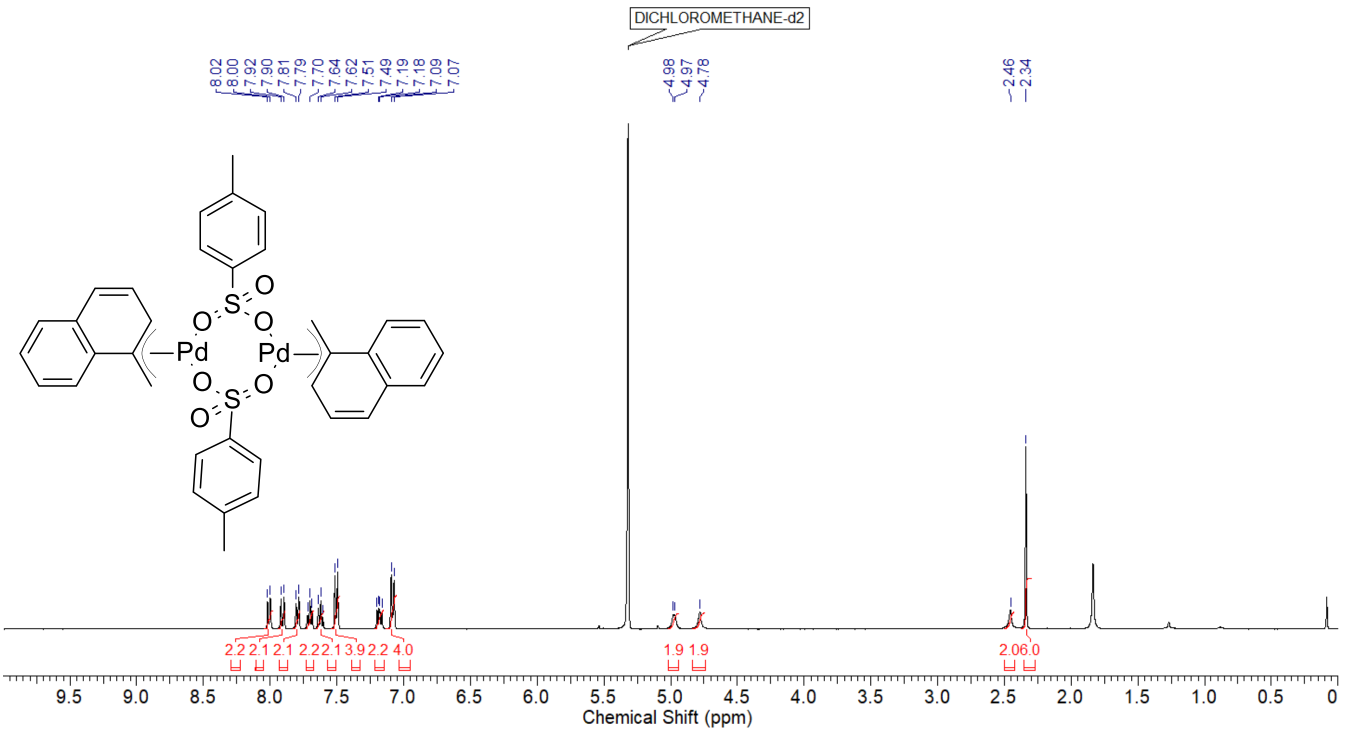

^{13}C NMR 75 MHz, THF-d^8):

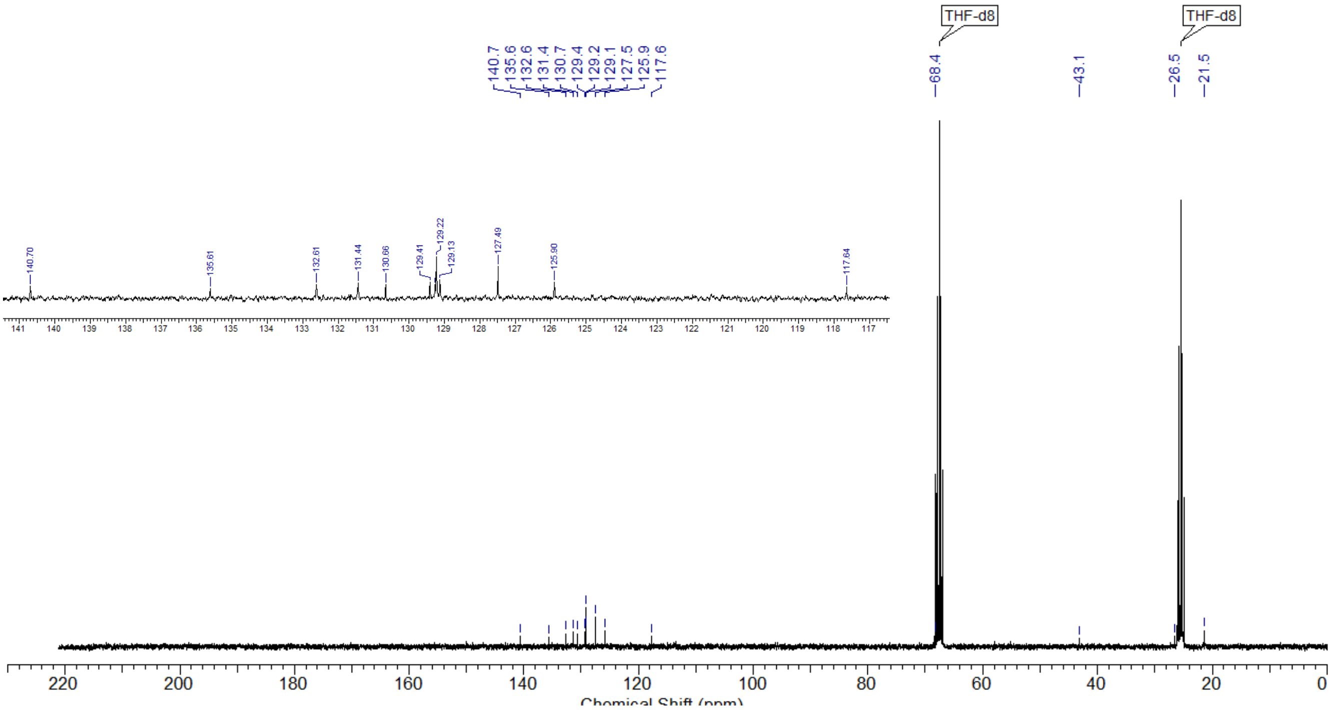

[Pd(1-MeNAP)(tBuBrettPhos)TFA]

^{1}H NMR (400 MHz, DCM-d^{2}):

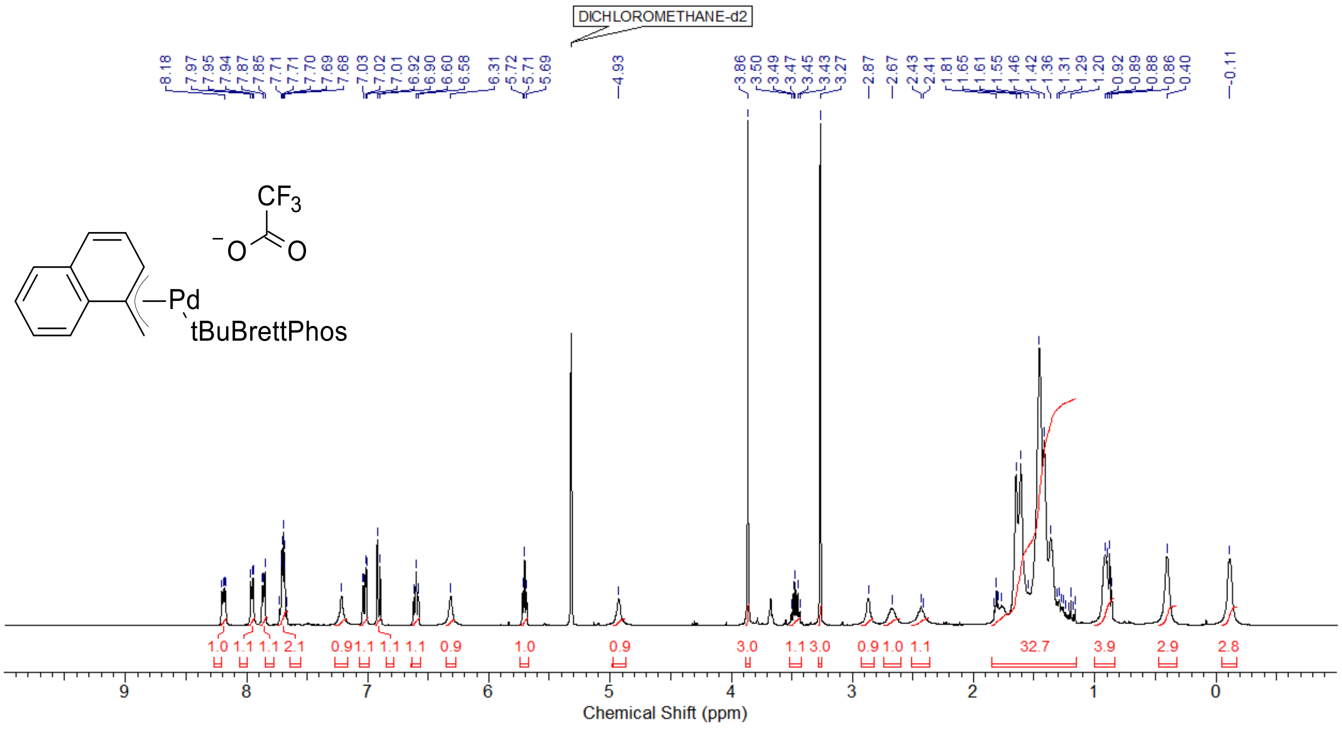

^{13}C NMR (101 MHz, DCM-d^{2}):

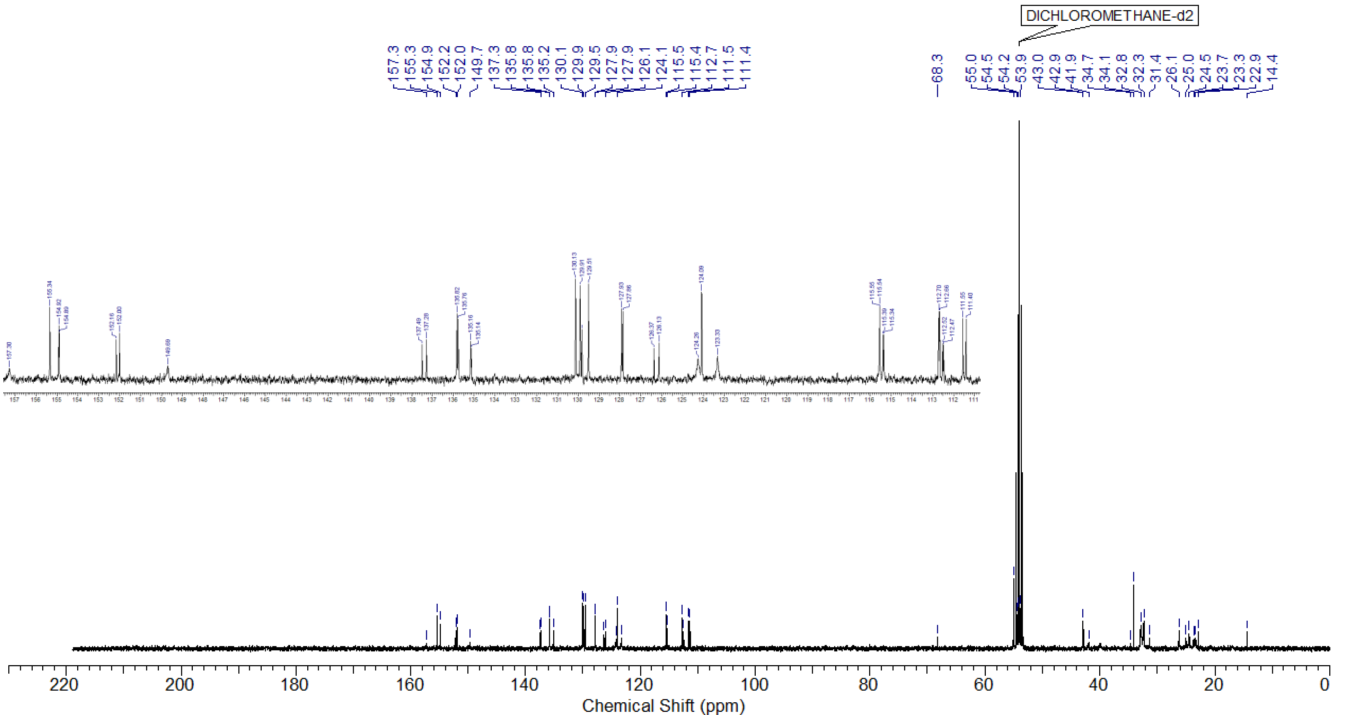

^{19}F NMR (76 MHz, DCM-d^2, $C_6H_4F_2$):

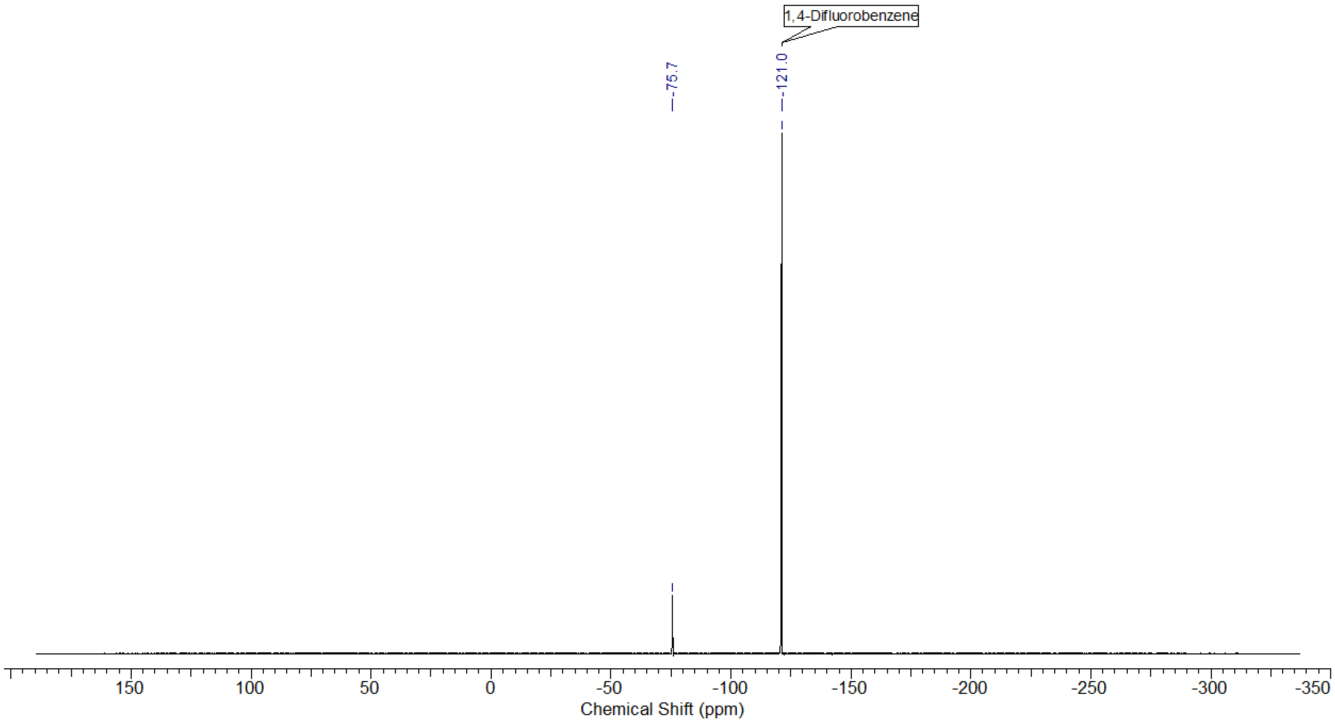

^{31}P NMR (162 MHz, DCM-d^2):

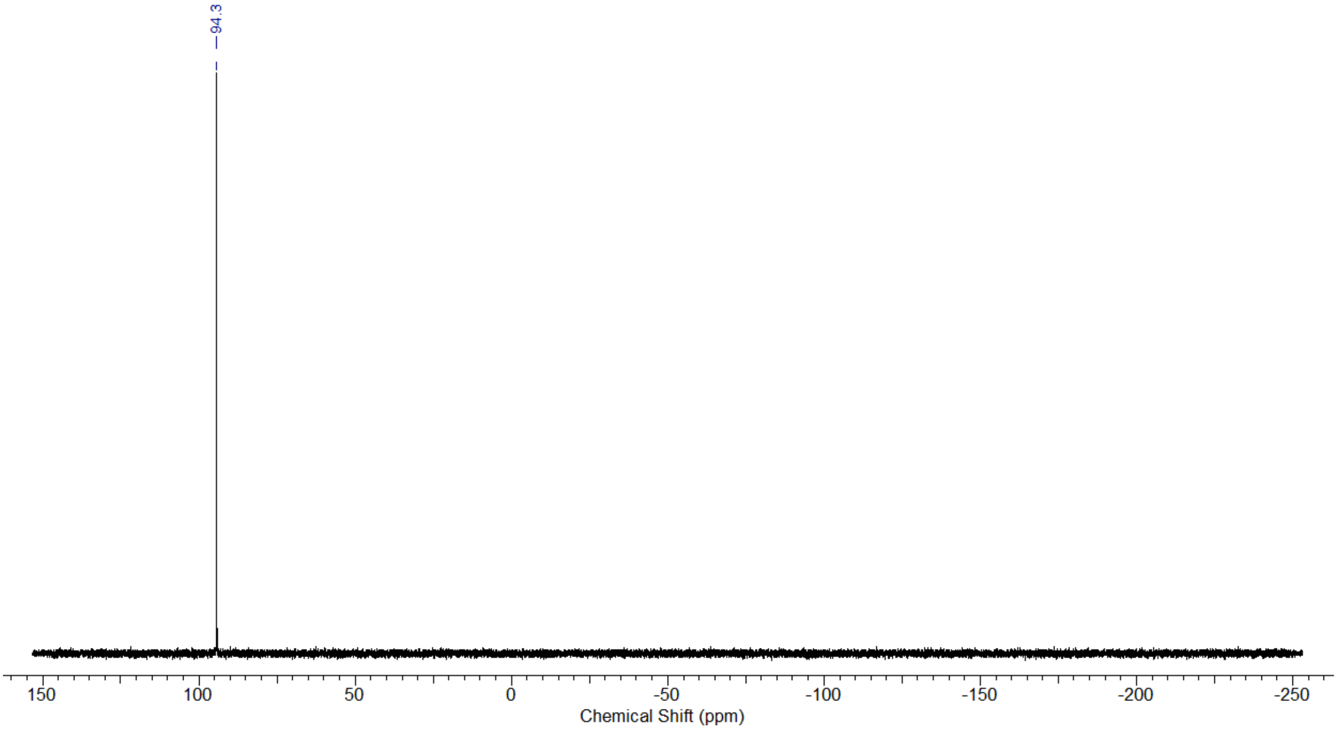

[Pd(1-MeNAP)(RuPhos)TFA]

^{1}H NMR (400 MHz, DCM-d^2):

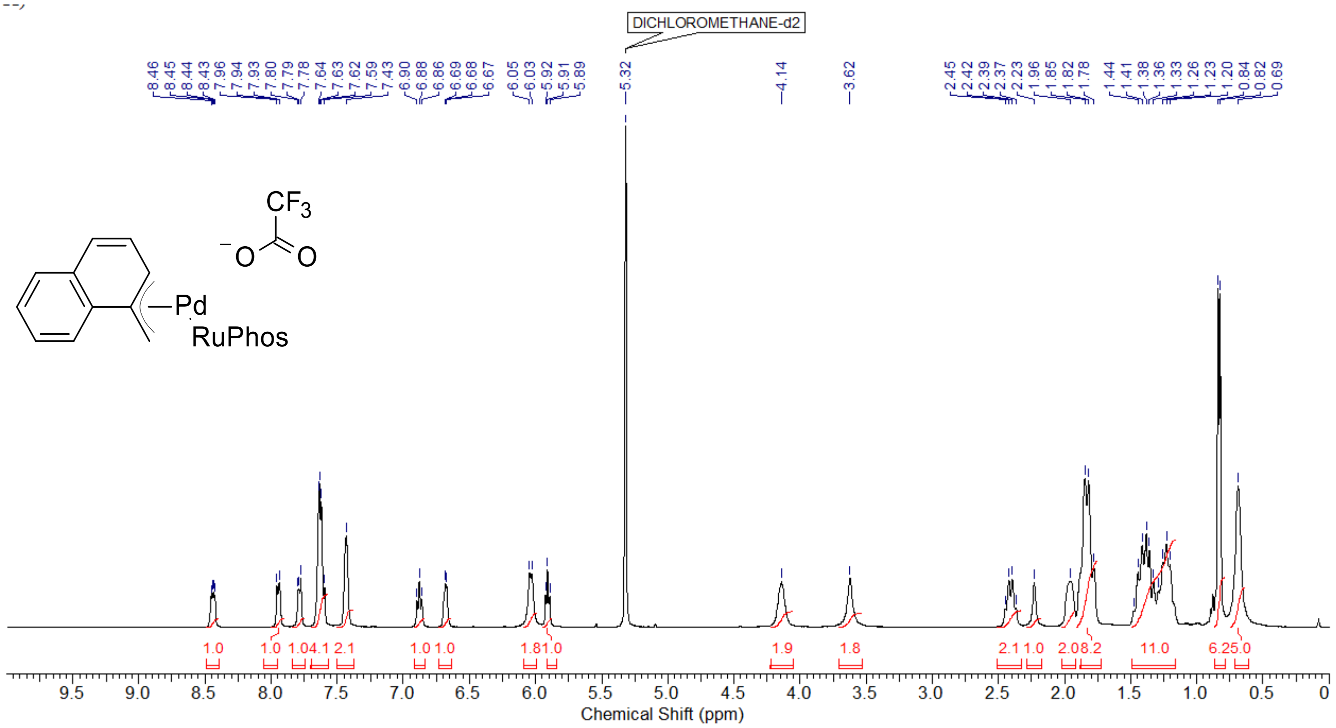

^{13}C NMR (101 MHz, DCM-d^2):

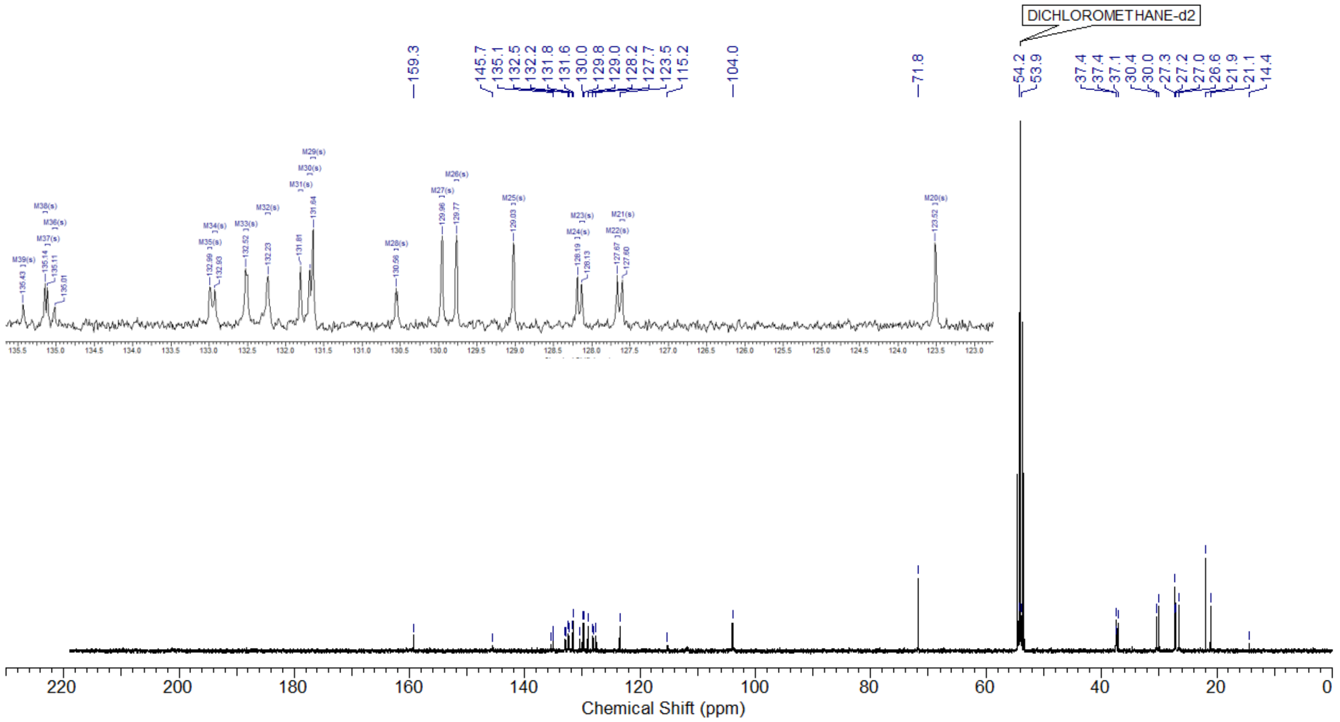

^{19}F NMR (76 MHz, DCM-d^2, $C_6H_4F_2$):

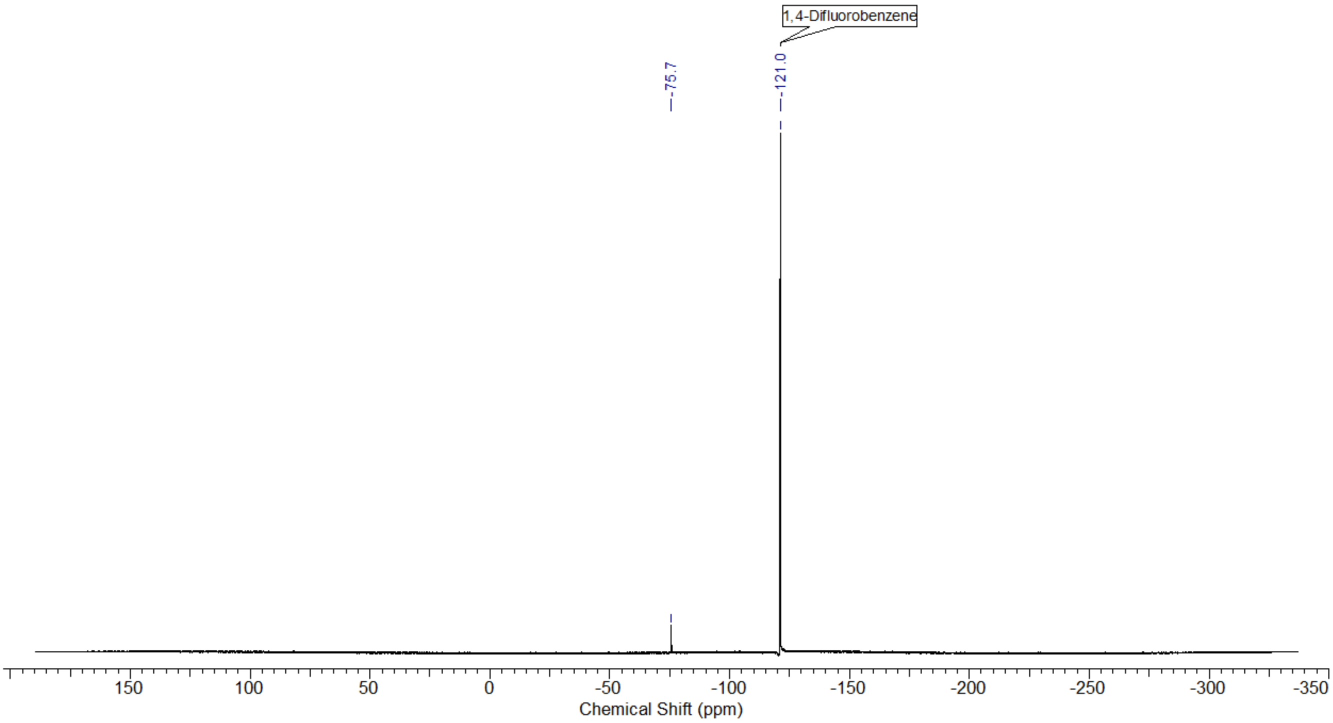

^{31}P NMR (162 MHz, DCM-d^2):

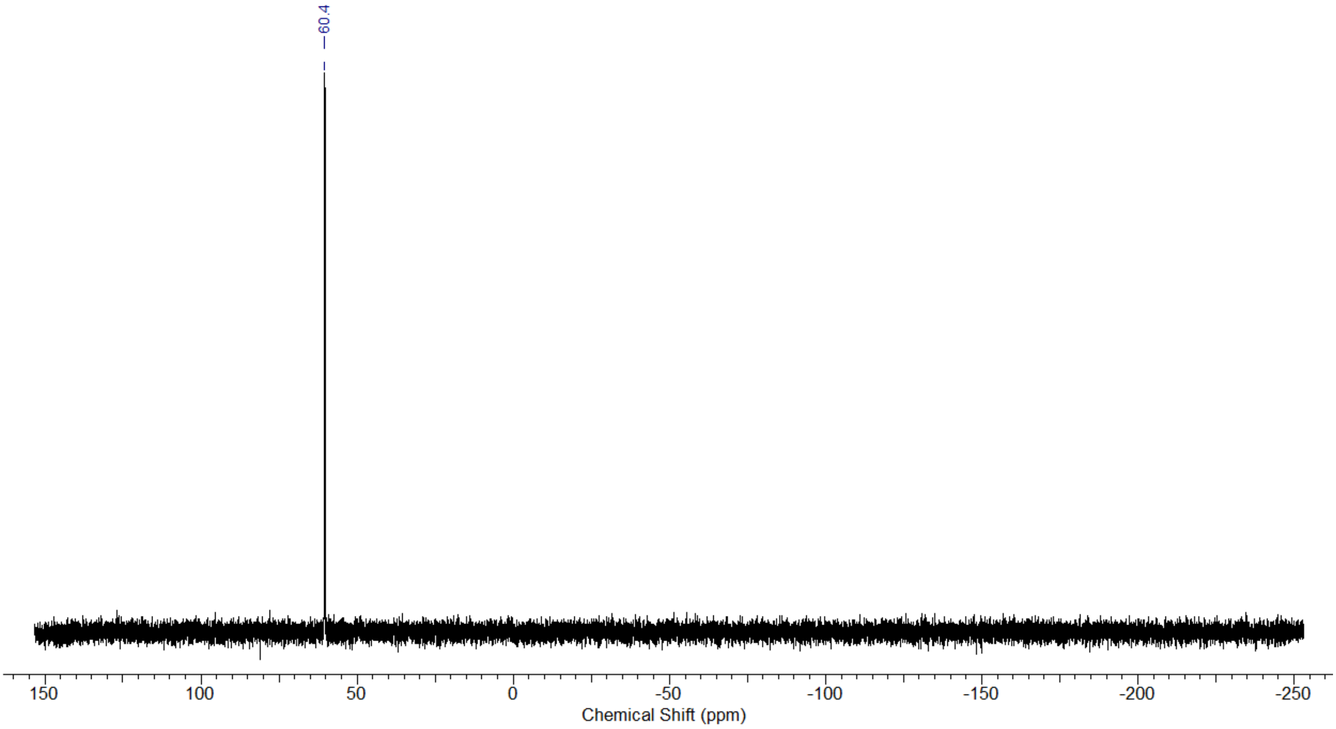

4-Methoxybenzanilide (**3a**) [CAS 7472-54-0]

^{1}H NMR (300 MHz, $CDCl_3$):

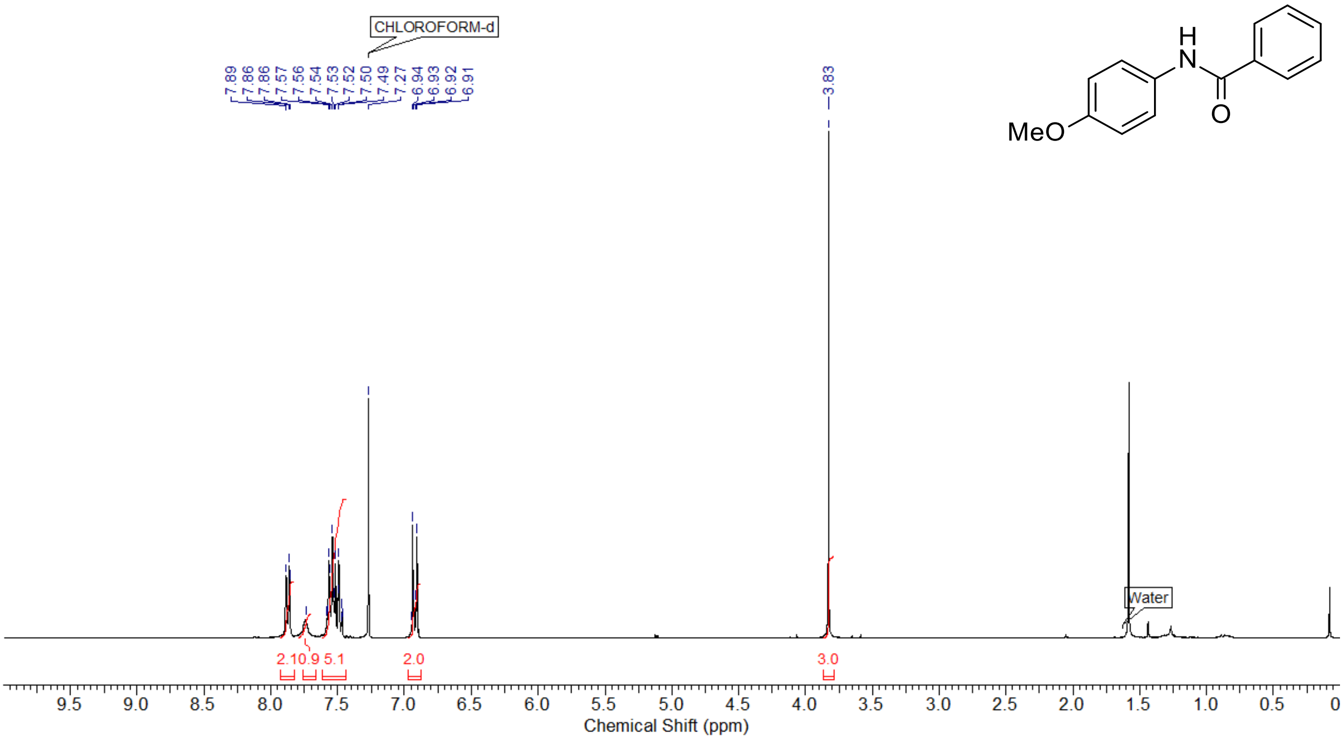

^{13}C NMR (75 MHz, $CDCl_3$):

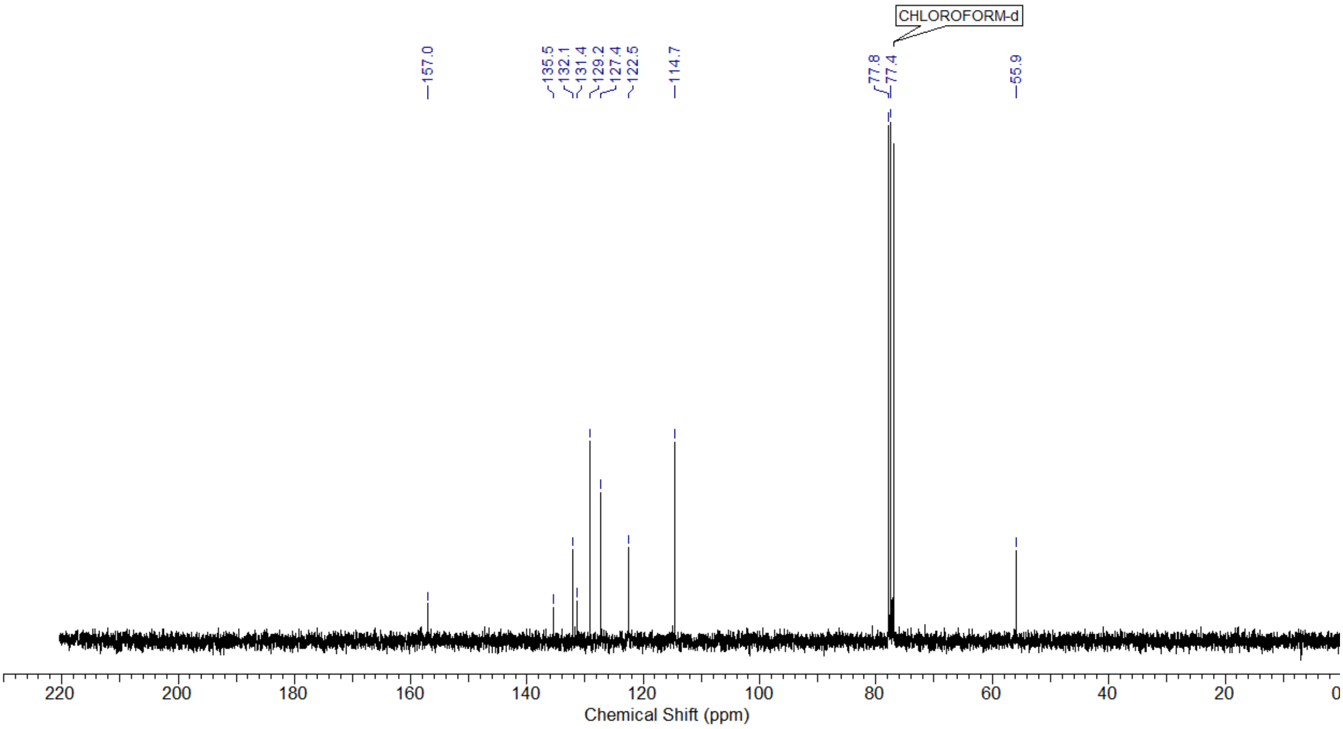

N-(3-methylbenzothiophen-5-yl)benzamide (**3b**) [CAS 10278-46-3]

^{1}H NMR (300 MHz, $CDCl_3$):

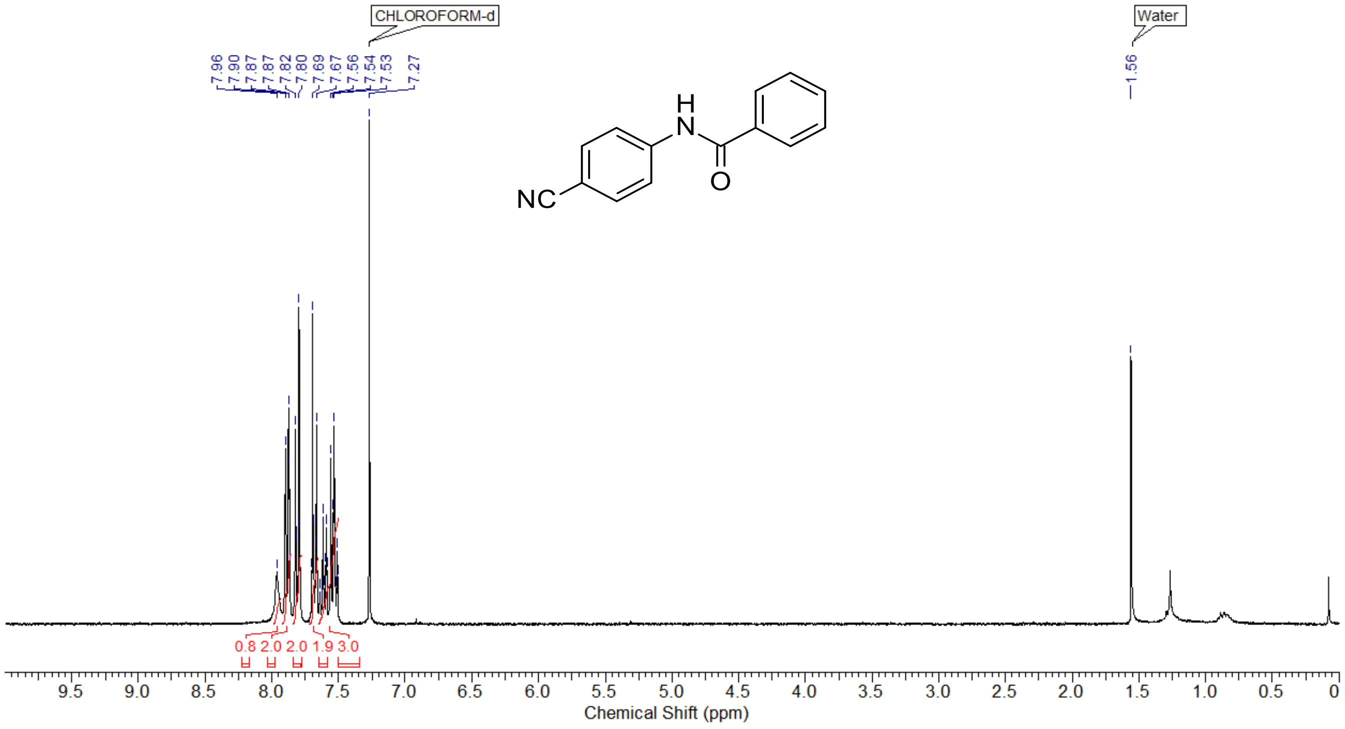

^{13}C NMR (75 MHz, $CDCl_3$):

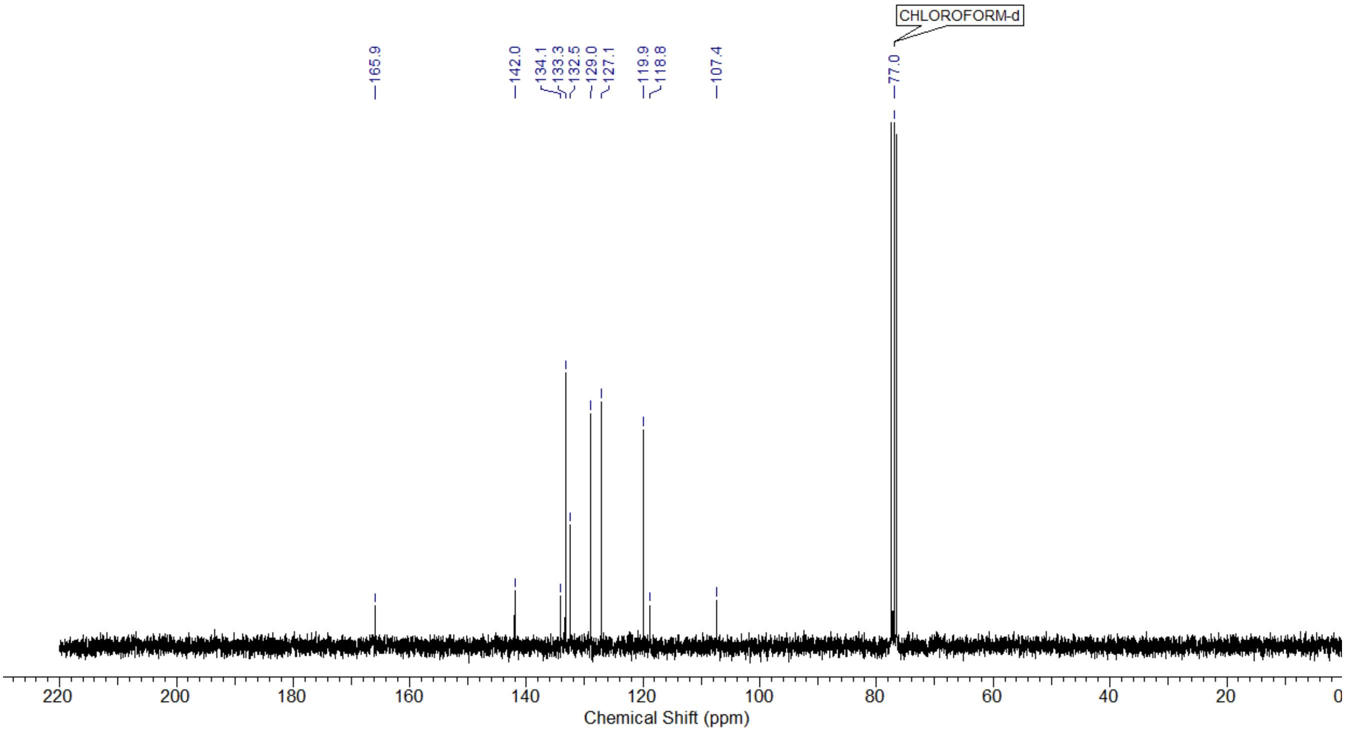

N-[4-(trifluoromethyl)phenyl]benzamide (**3c**) [CAS 350-98-1]

^{1}H NMR (300 MHz, DMSO-d^{6}):

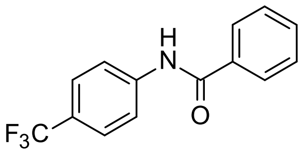

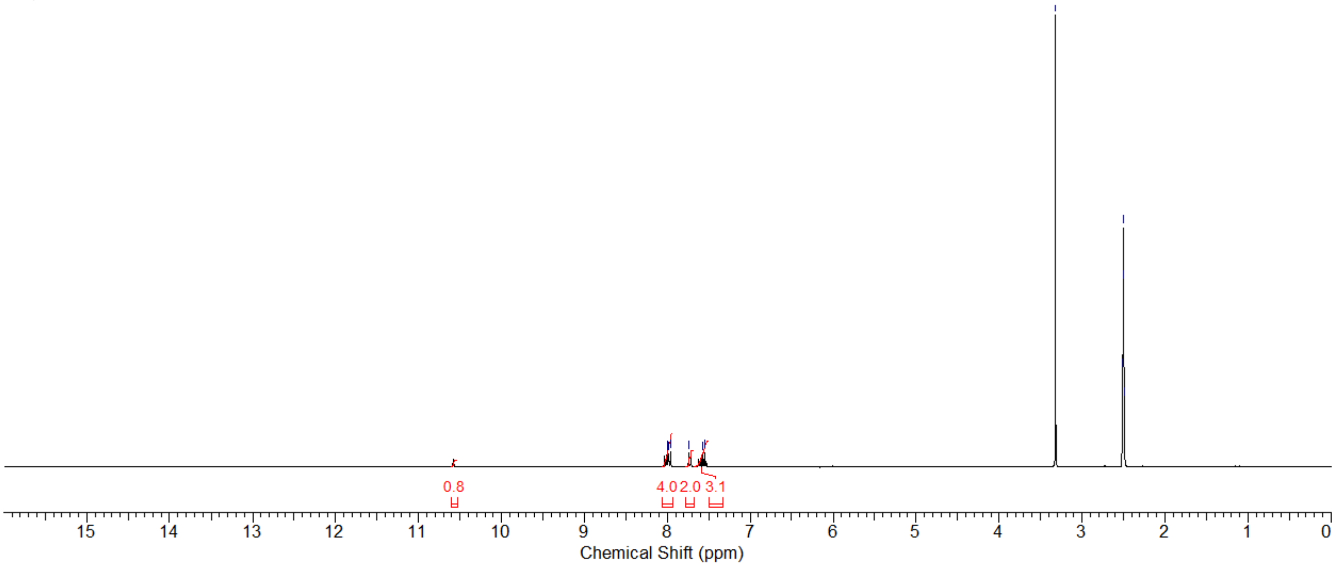

^{13}C NMR (101 MHz, DMSO-d^{6}):

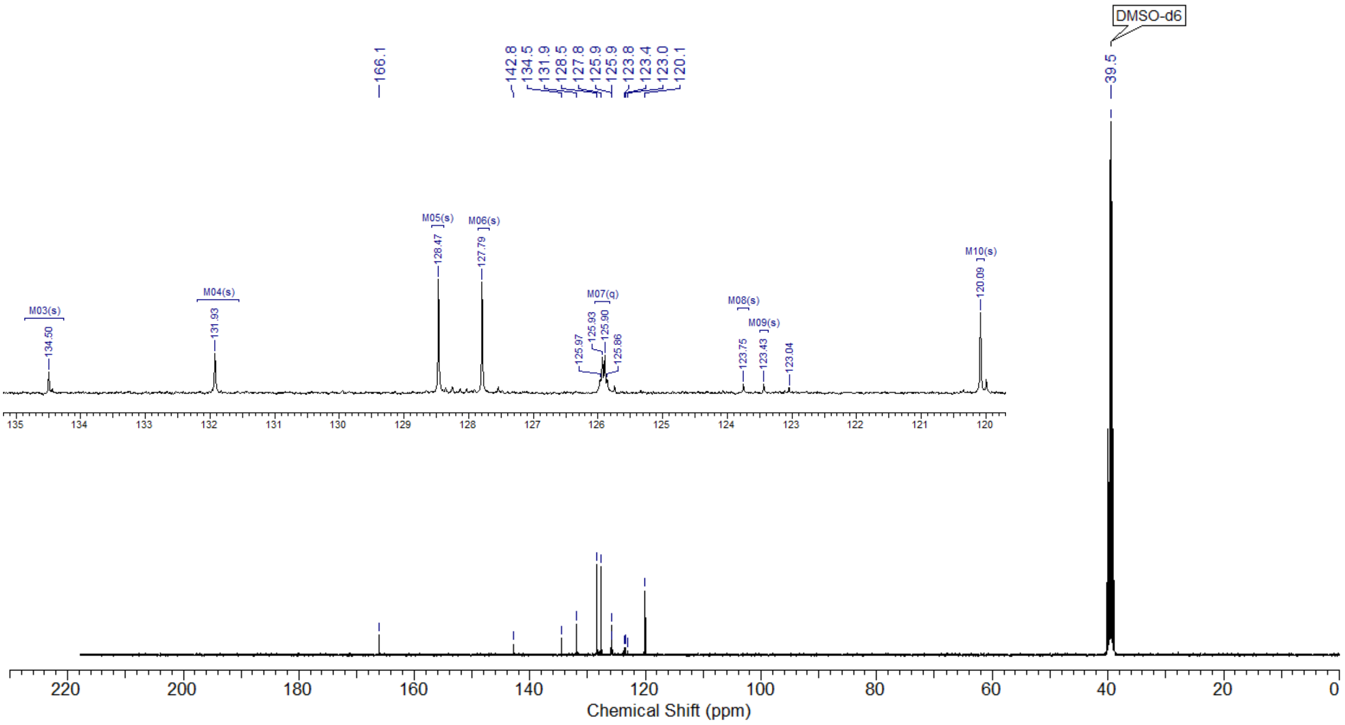

^{19}F NMR (76 MHz, $CDCl_3$, DMSO-d^6):

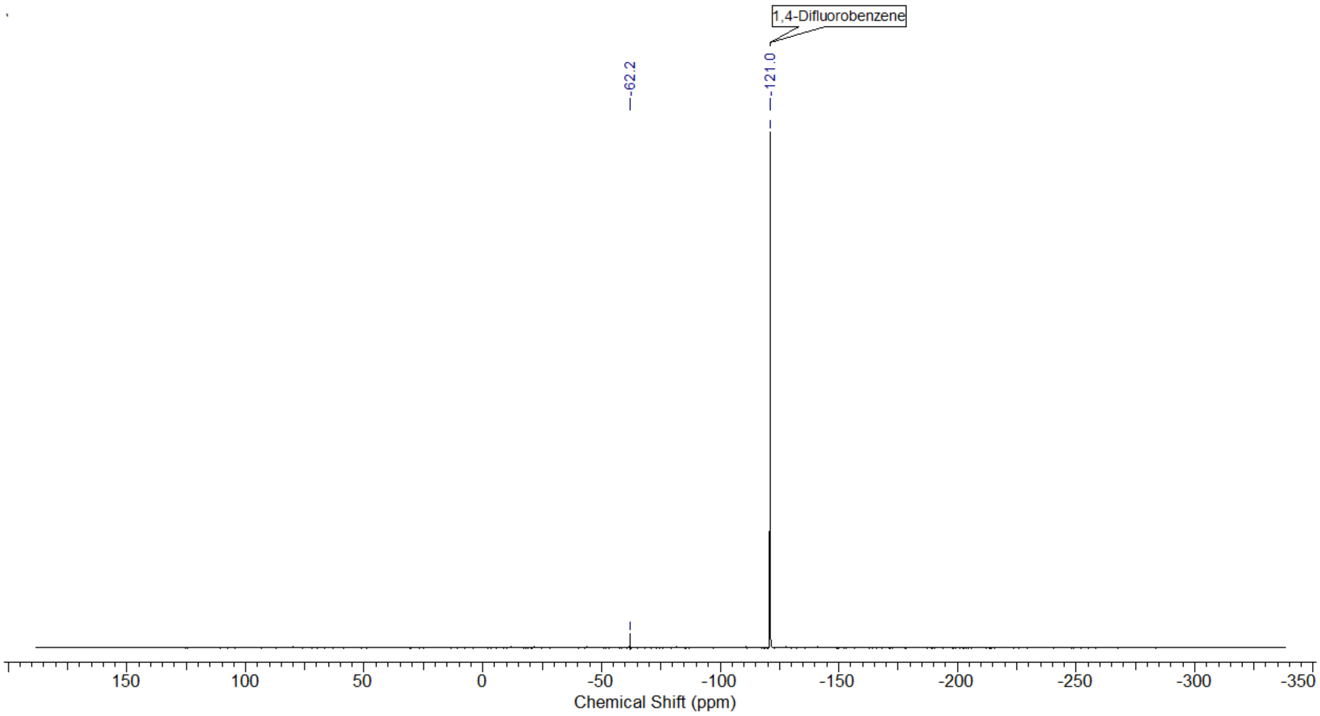

N-(4-trimethylsilylphenyl)benzamide (**3d**)

^{1}H NMR (300 MHz, $CDCl_3$):

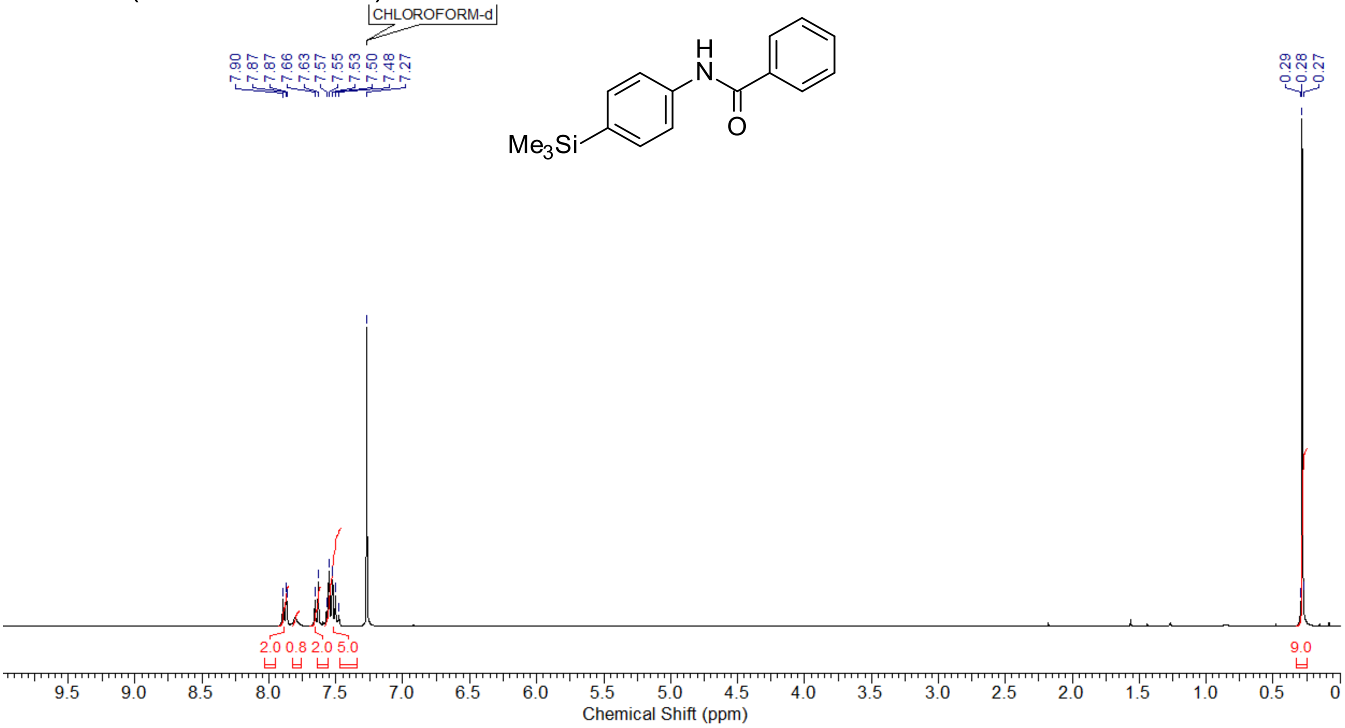

^{13}C NMR (75 MHz, $CDCl_3$):

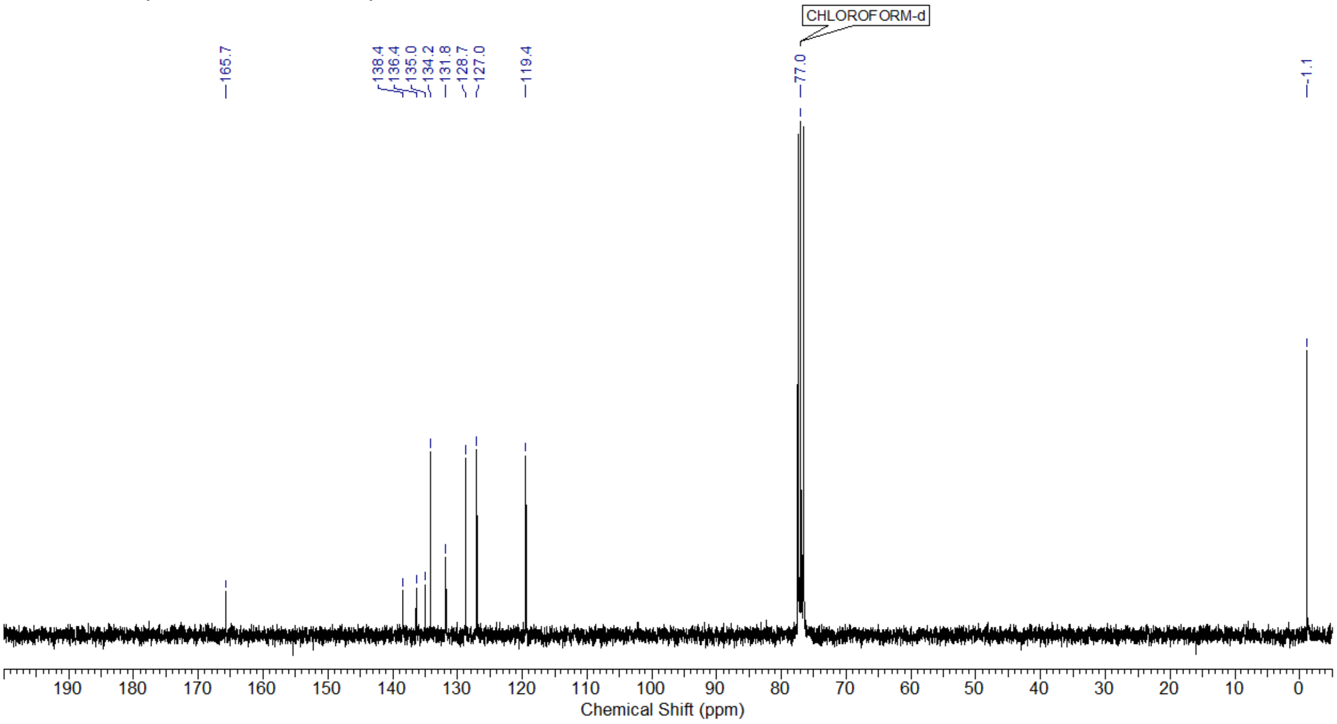

N-(4-formylphenyl)benzamide (**3e**) [CAS 65854-93-5]

^{1}H NMR (300 MHz, $CDCl_3$):

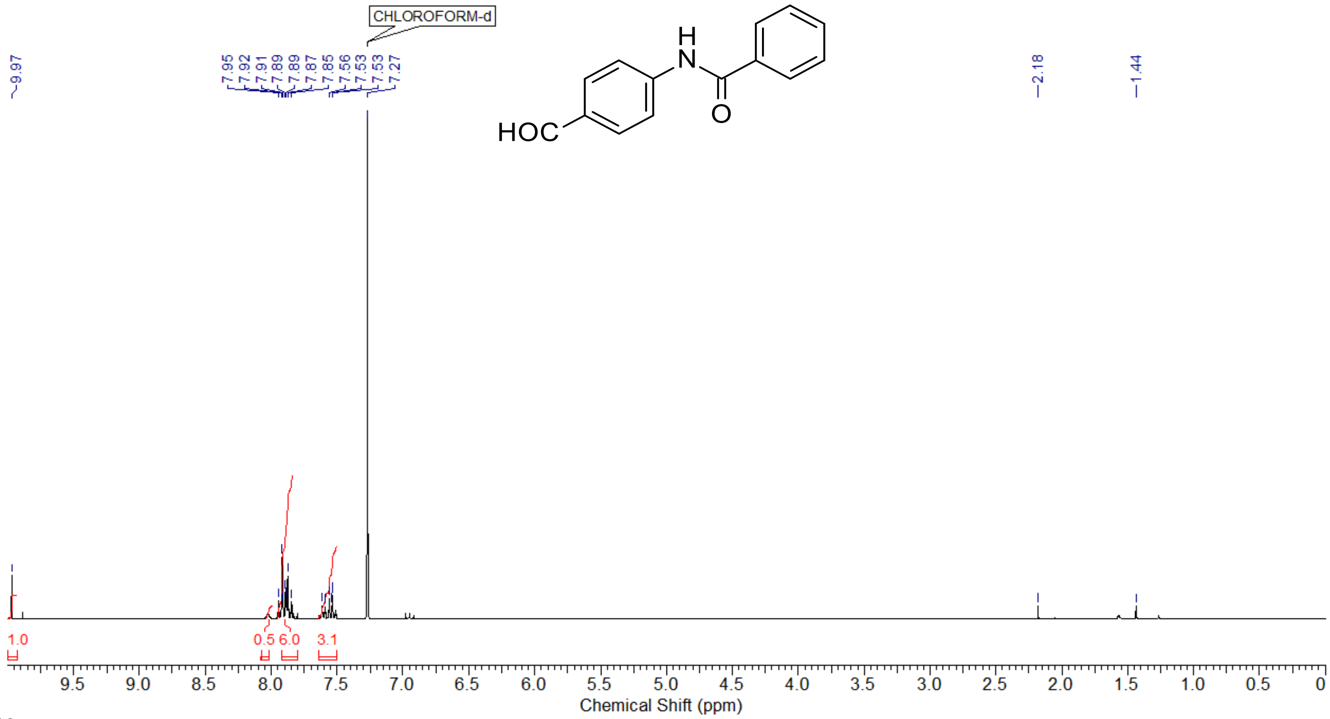

^{13}C NMR (75 MHz, $CDCl_3$):

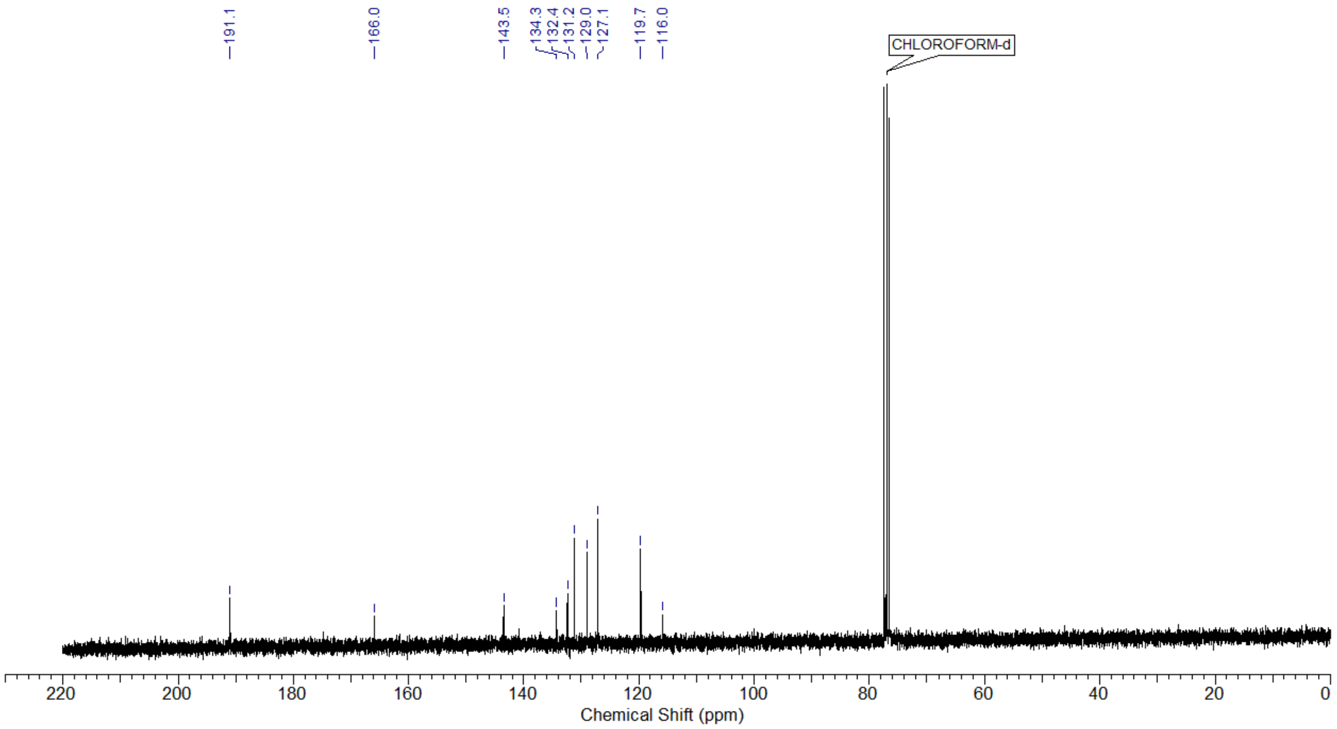

N-[4-(4,4,5,5-tetramethyl-1,3,2-dioxaborolan-2-yl)phenyl]benzamide (**3f**) [CAS 935660-75-6]

^{1}H NMR (300 MHz, $CDCl_3$):

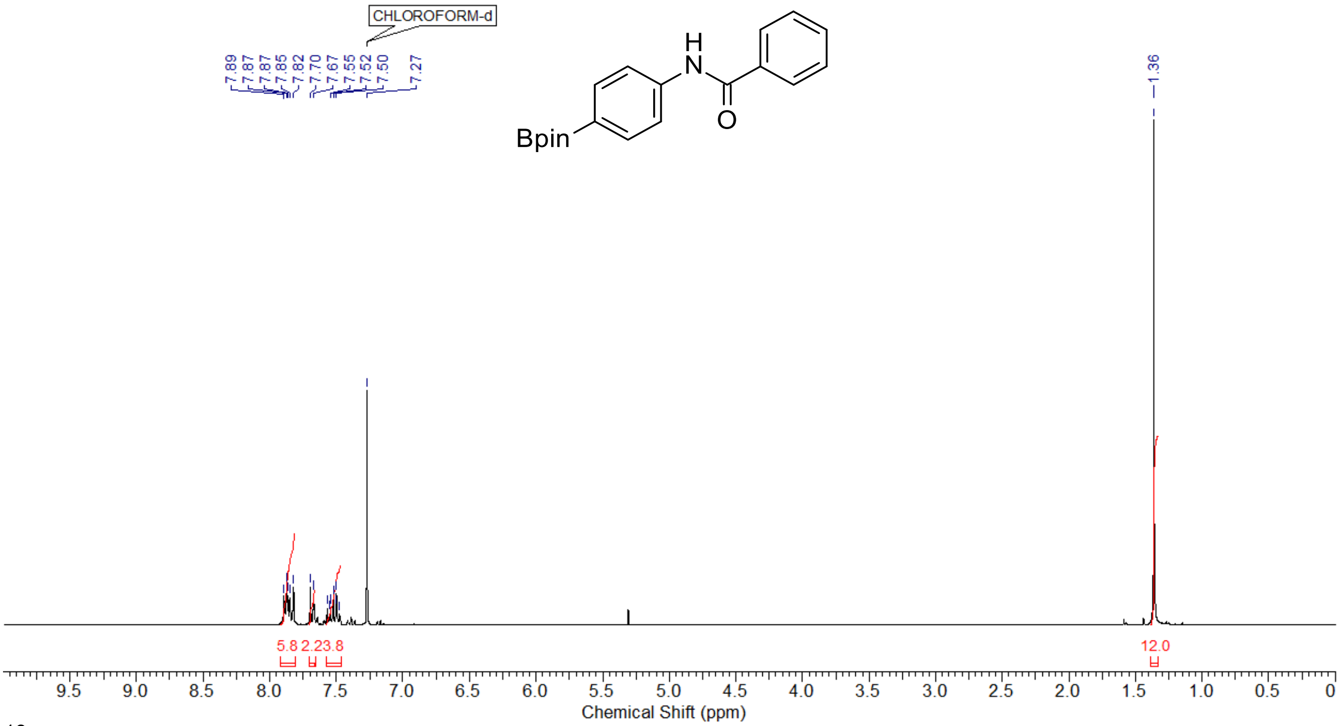

^{13}C NMR (75 MHz, $CDCl_3$):

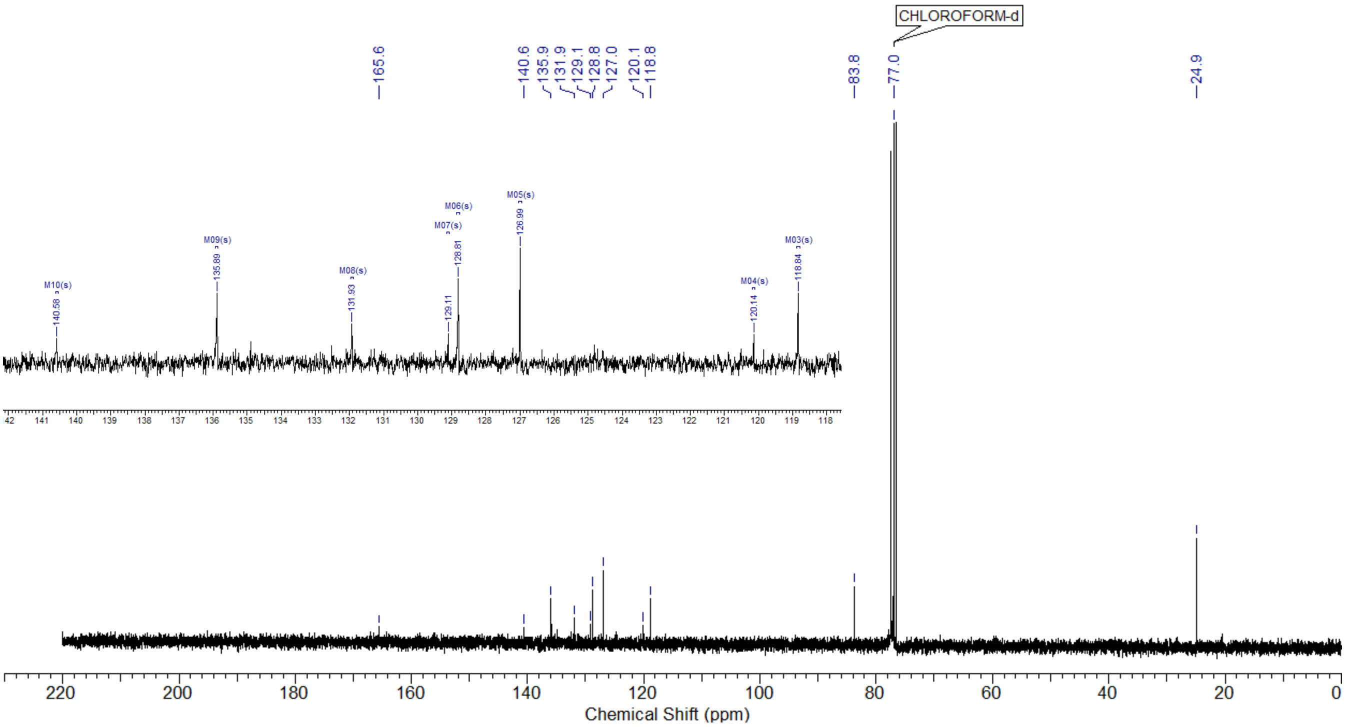

N-(3,5-Dimethoxyphenyl)benzamide (**3g**) [CAS 94088-74-1]

^{1}H NMR (300 MHz, $CDCl_3$):

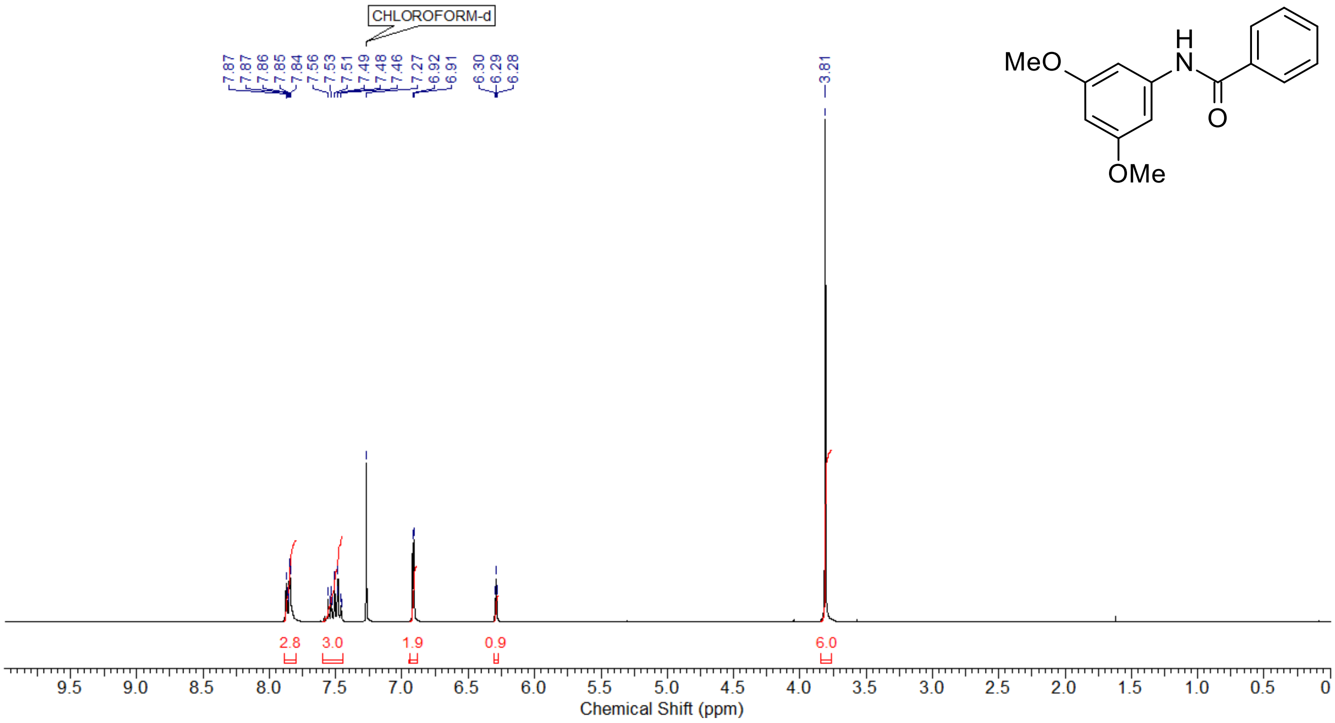

^{13}C NMR (75 MHz, $CDCl_3$):

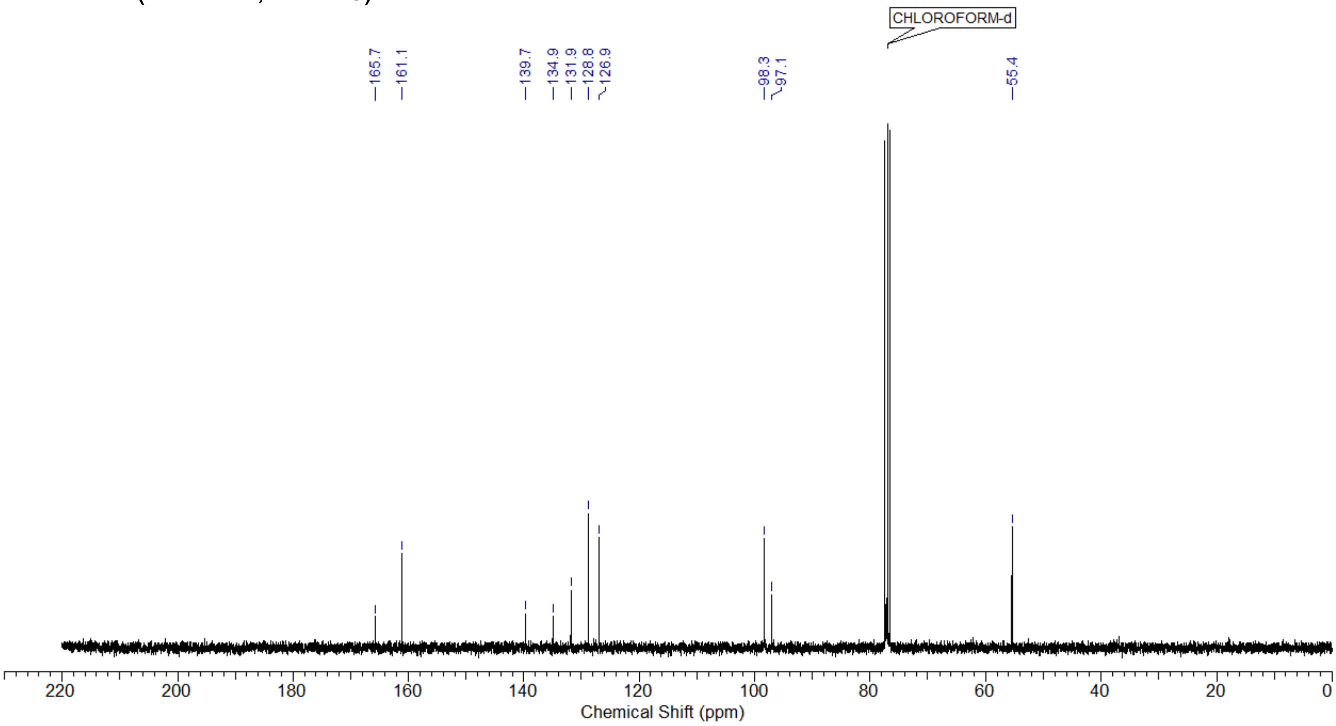

N-(3-pyridyl)benzamide (**3h**) [CAS 5221-40-9]

^{1}H NMR (300 MHz, $CDCl_3$):

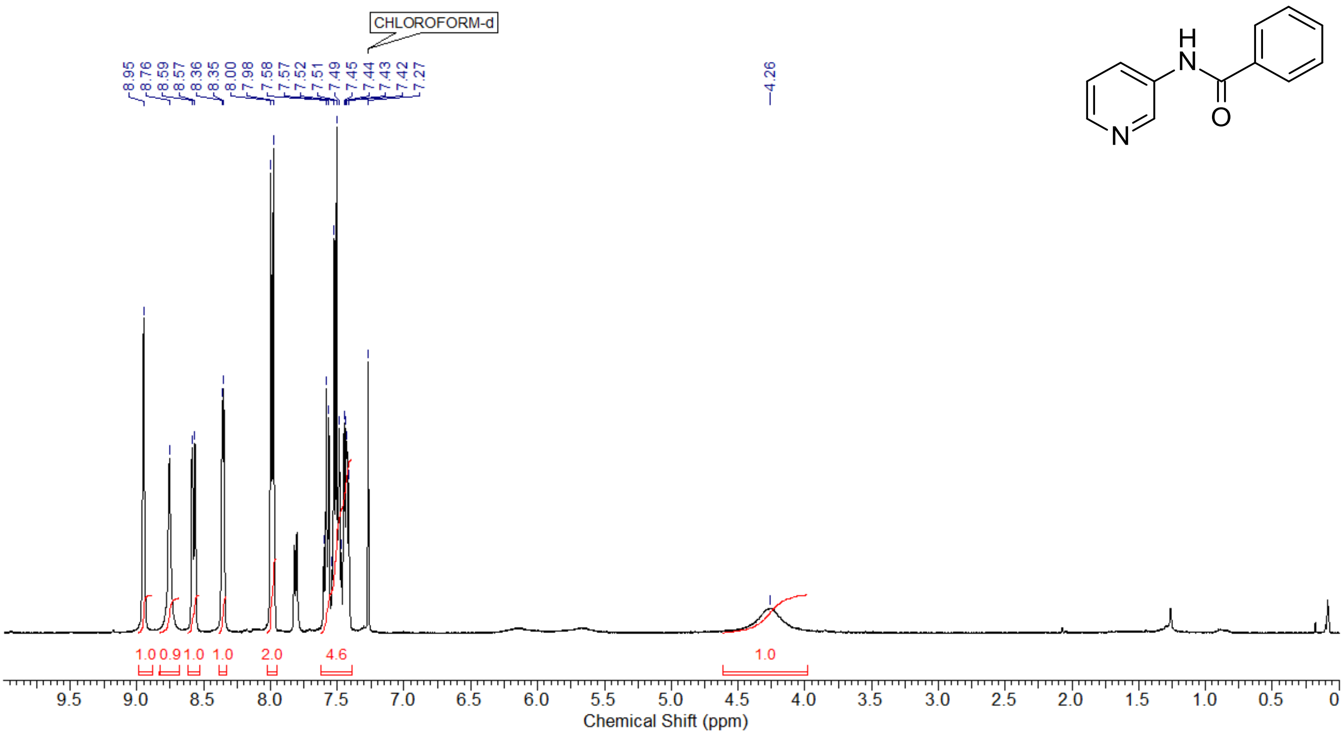

^{13}C NMR (75 MHz, $CDCl_3$):

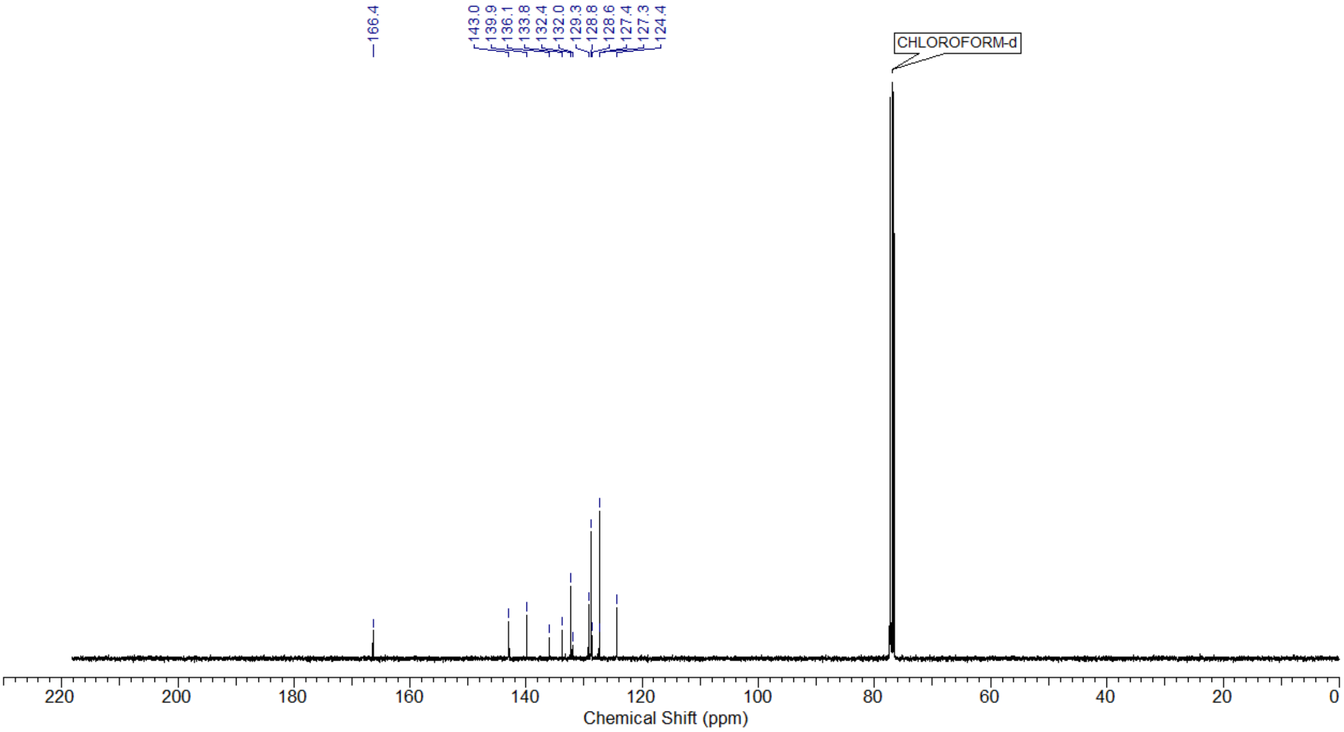

*N-(6-Methoxy-3-pyridazinyl)benzamide (**3i**) [CAS 39614-86-3]*

^{1}H NMR (300 MHz, *DMSO-d6*):

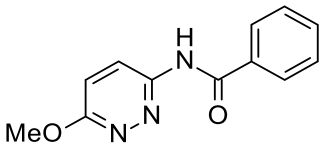

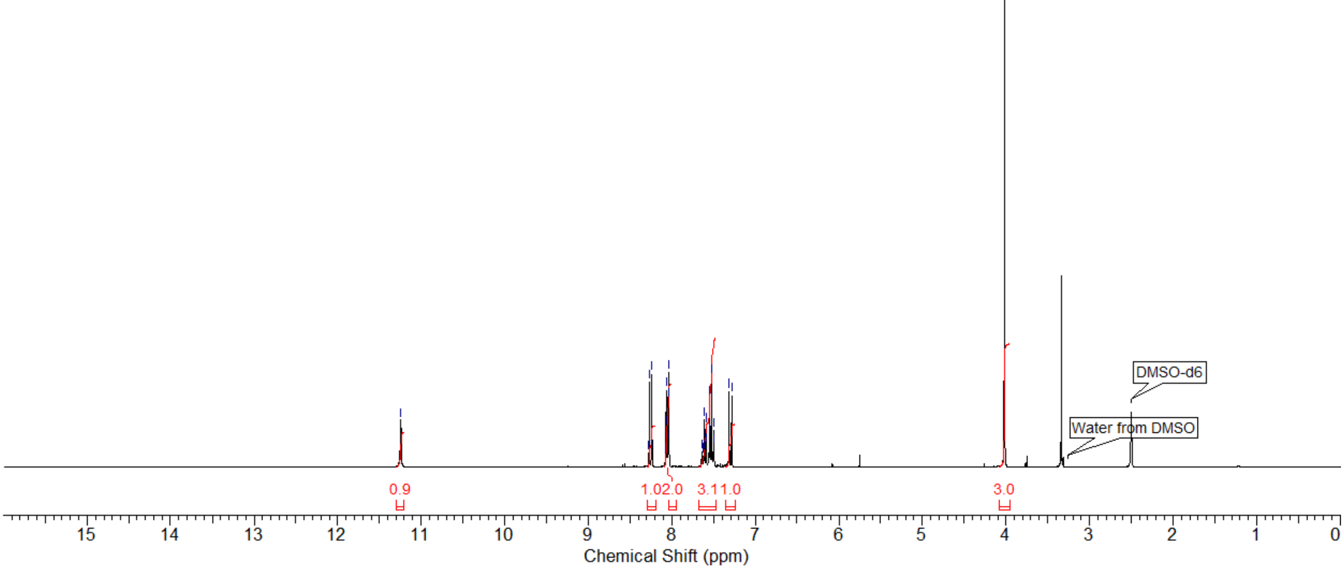

^{13}C NMR (75 MHz, *DMSO-d6*):

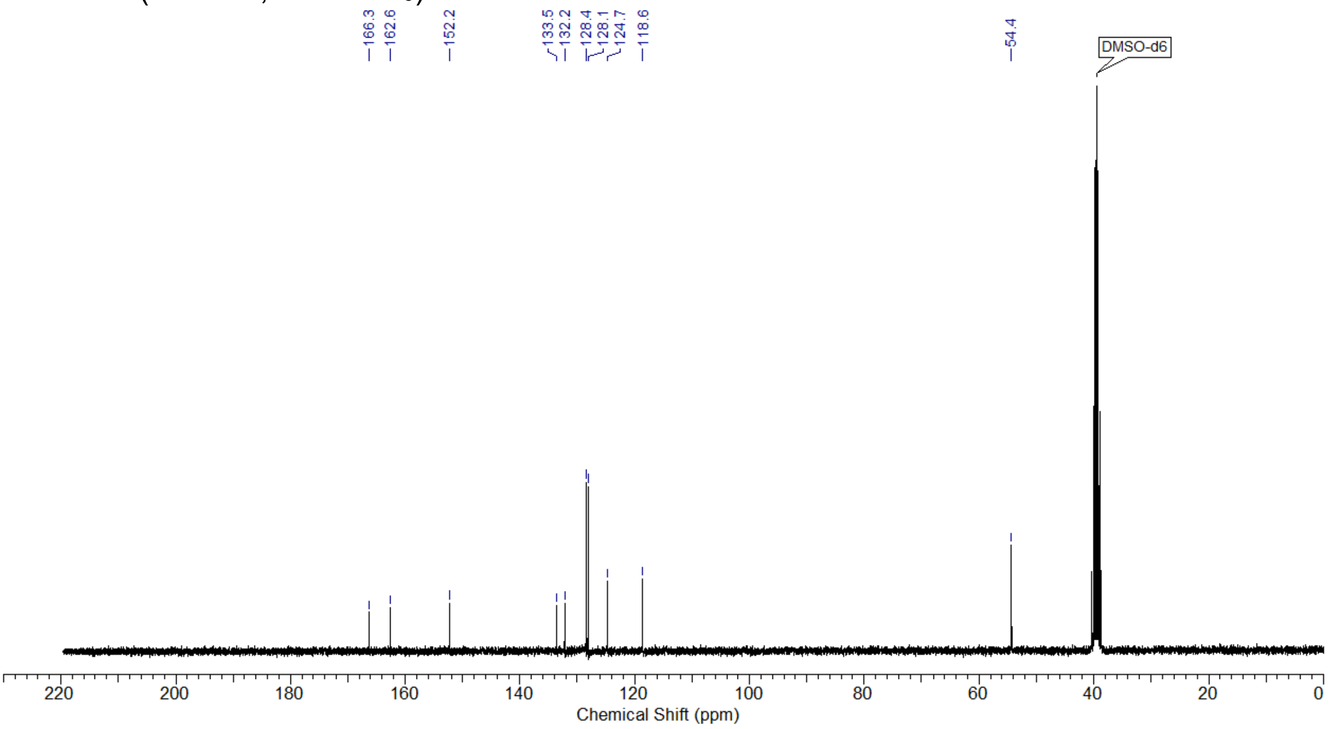

N-[4-(1-methylethenyl)phenyl]benzamide (**3j**) [CAS 2966931-58-6]

^{1}H NMR (300 MHz, $CDCl_3$):

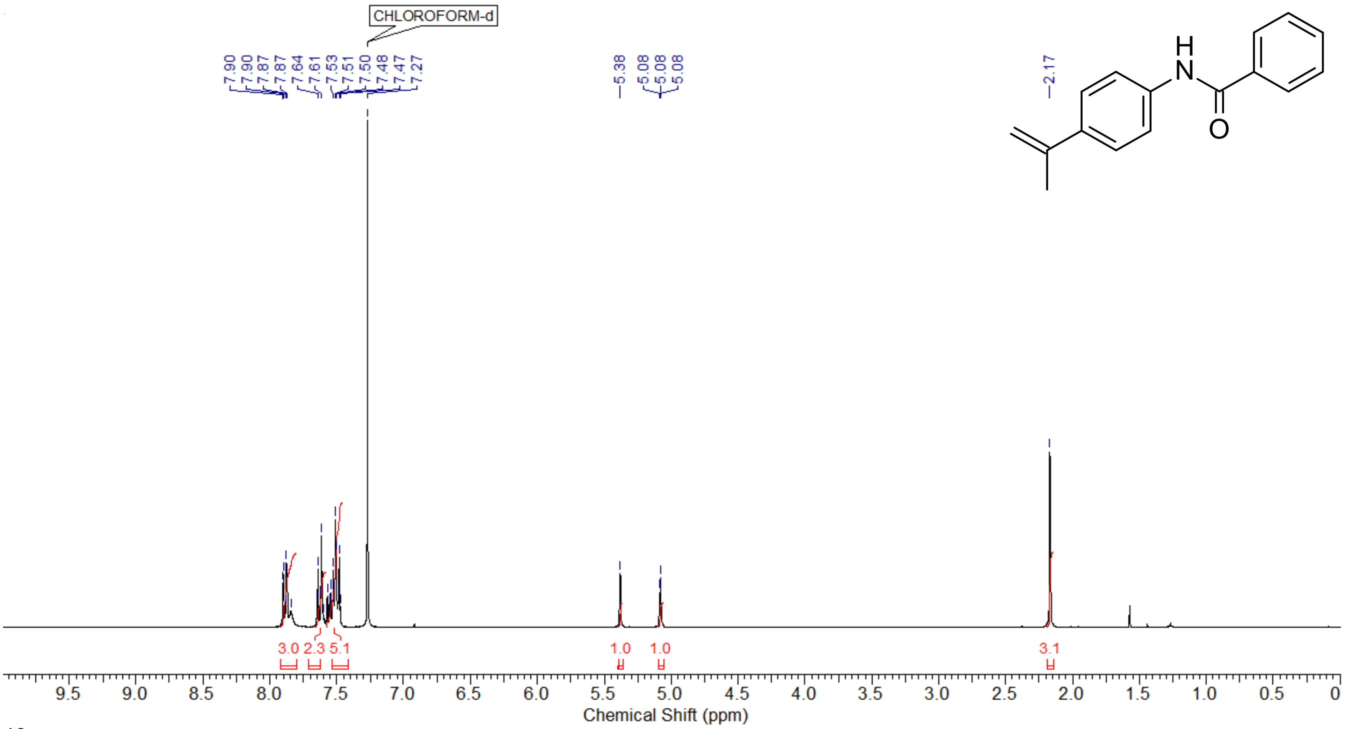

^{13}C NMR (75 MHz, $CDCl_3$):

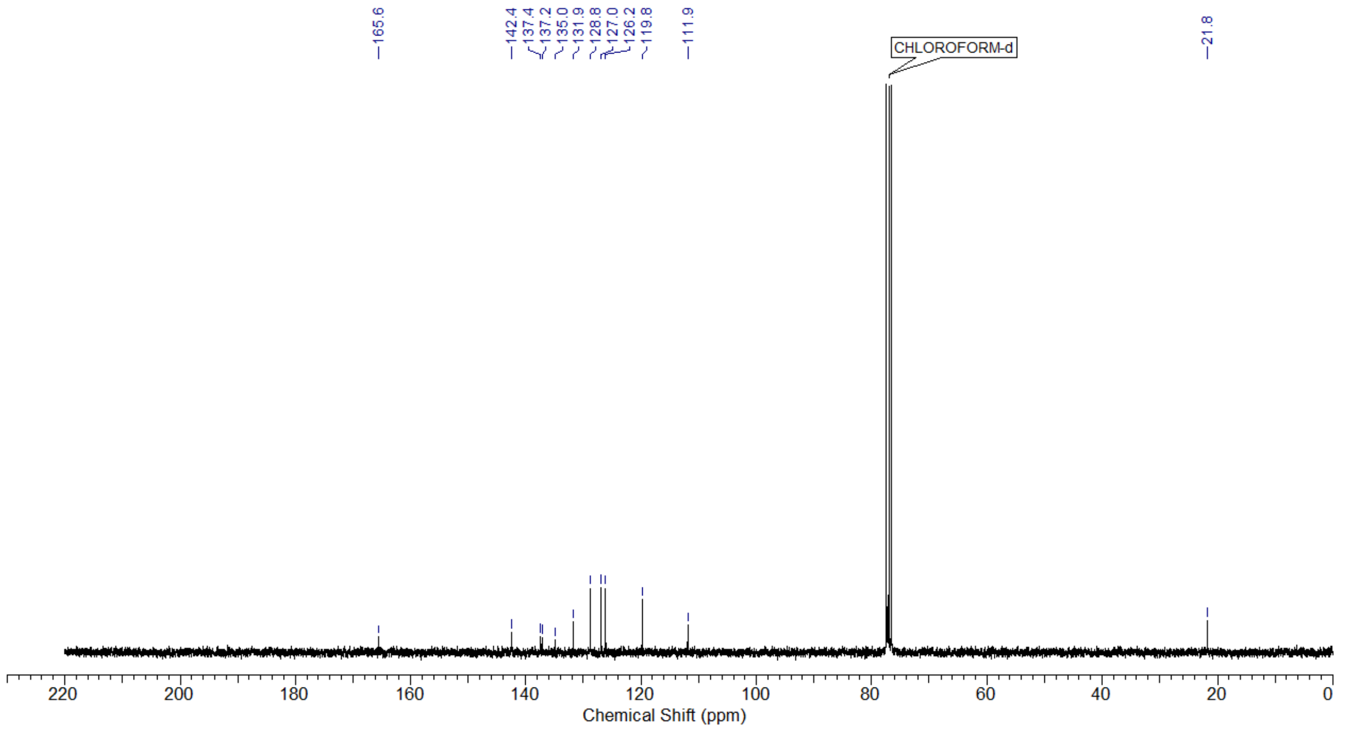

N-3-Thienylbenzamide (**3k**) [CAS 79128-75-9]

^{1}H NMR (300 MHz, $CDCl_3$):

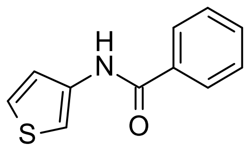

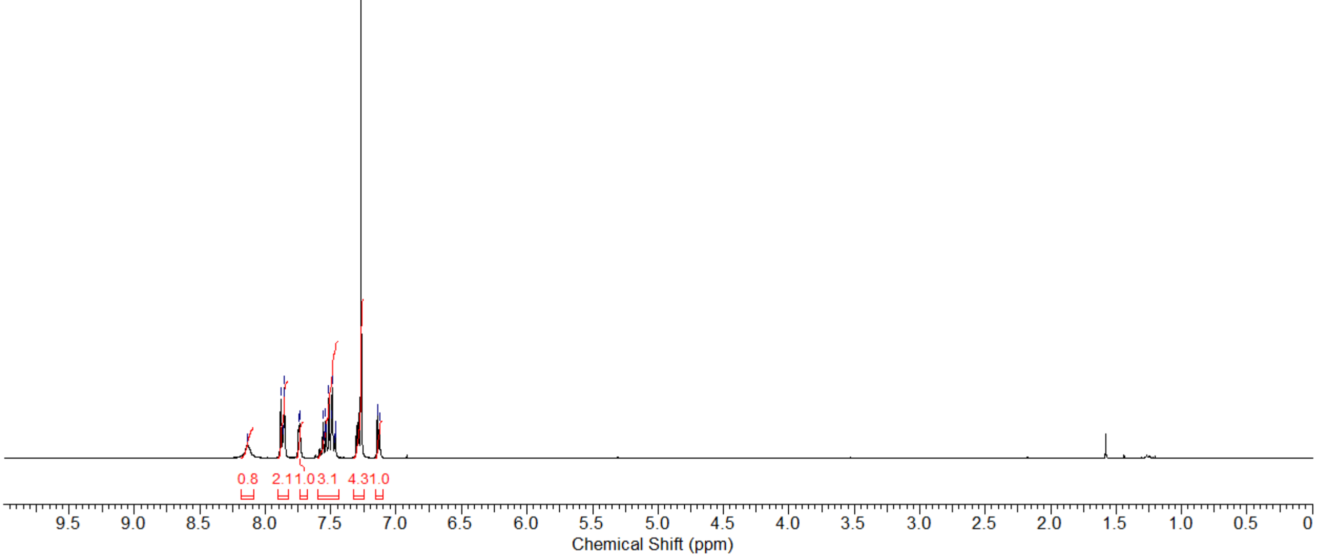

^{13}C NMR (75 MHz, $CDCl_3$):

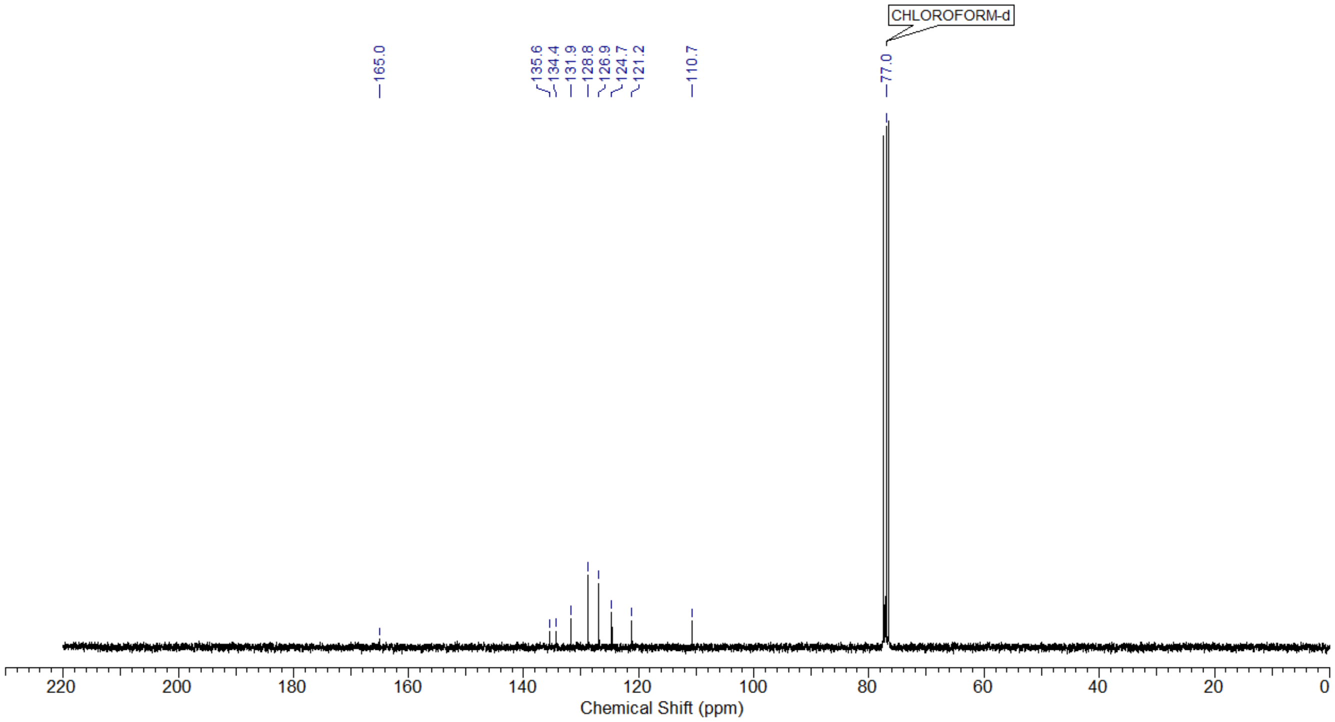

N-4-Thiazolylbenzamide (**3l**) [CAS 71168-41-7]

^{1}H NMR (300 MHz, $CDCl_3$):

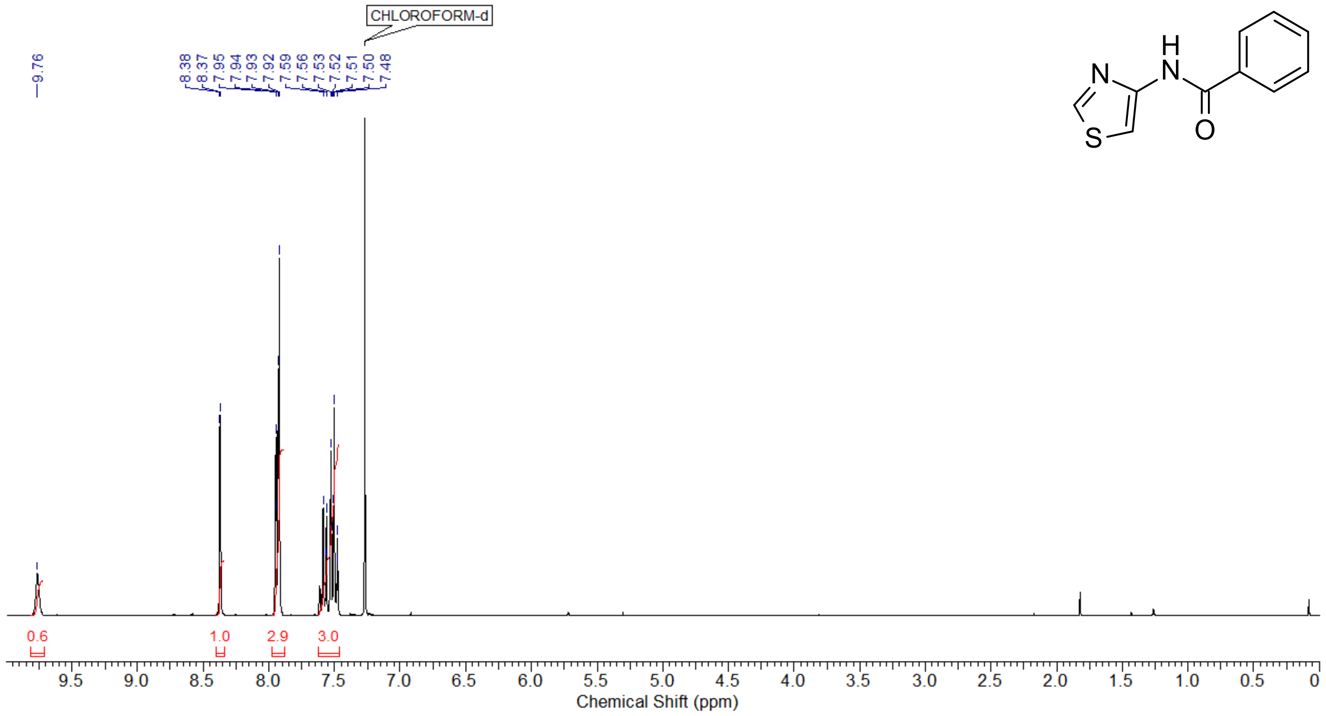

^{13}C NMR (75 MHz, $CDCl_3$):

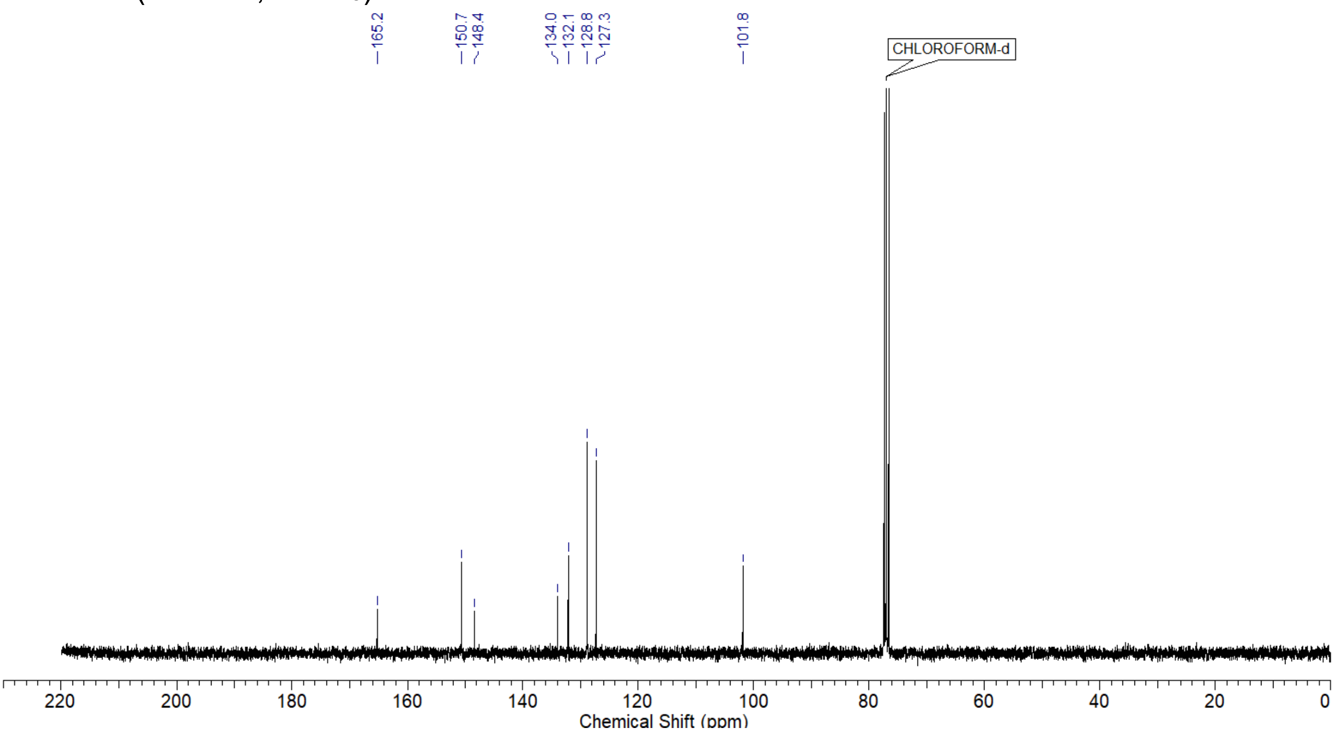

N-(3-methylbenzothiophen-5-yl)benzamide (**3m**)

^{1}H NMR (300 MHz, $CDCl_3$):

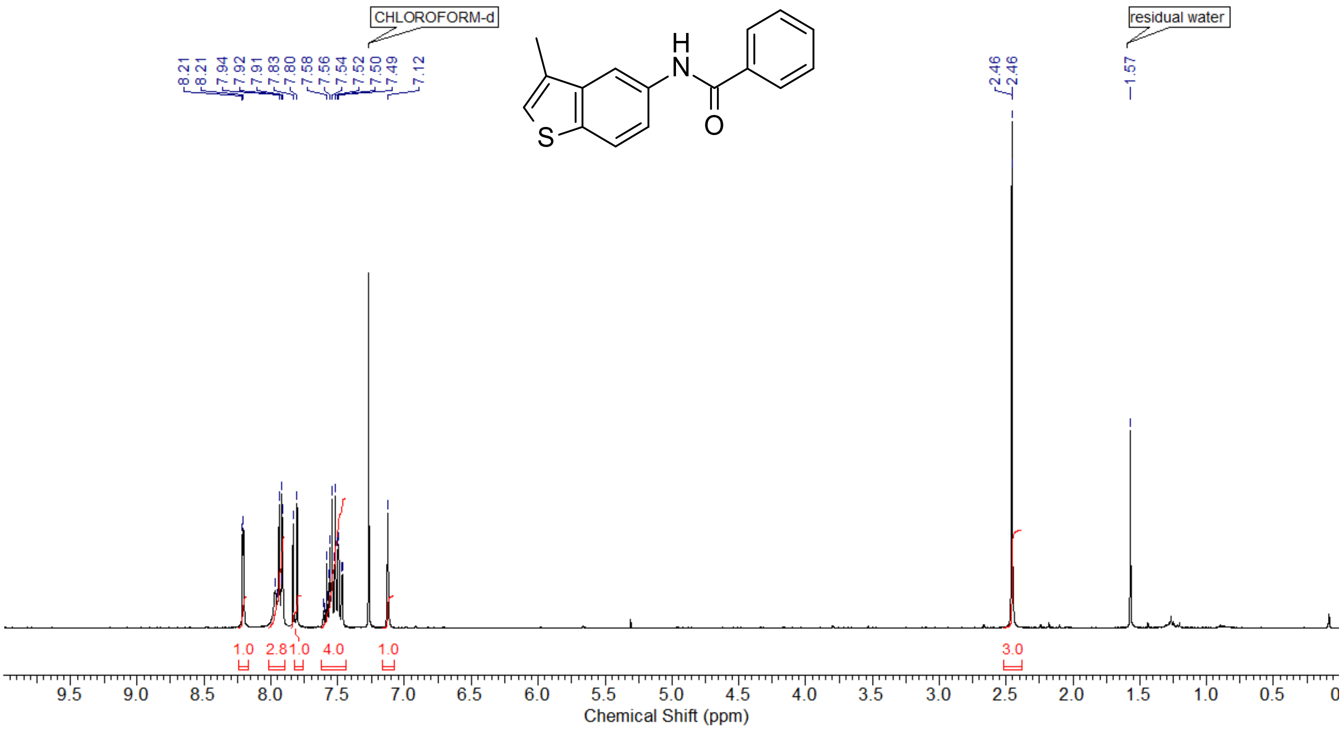

^{13}C NMR (75 MHz, $CDCl_3$):

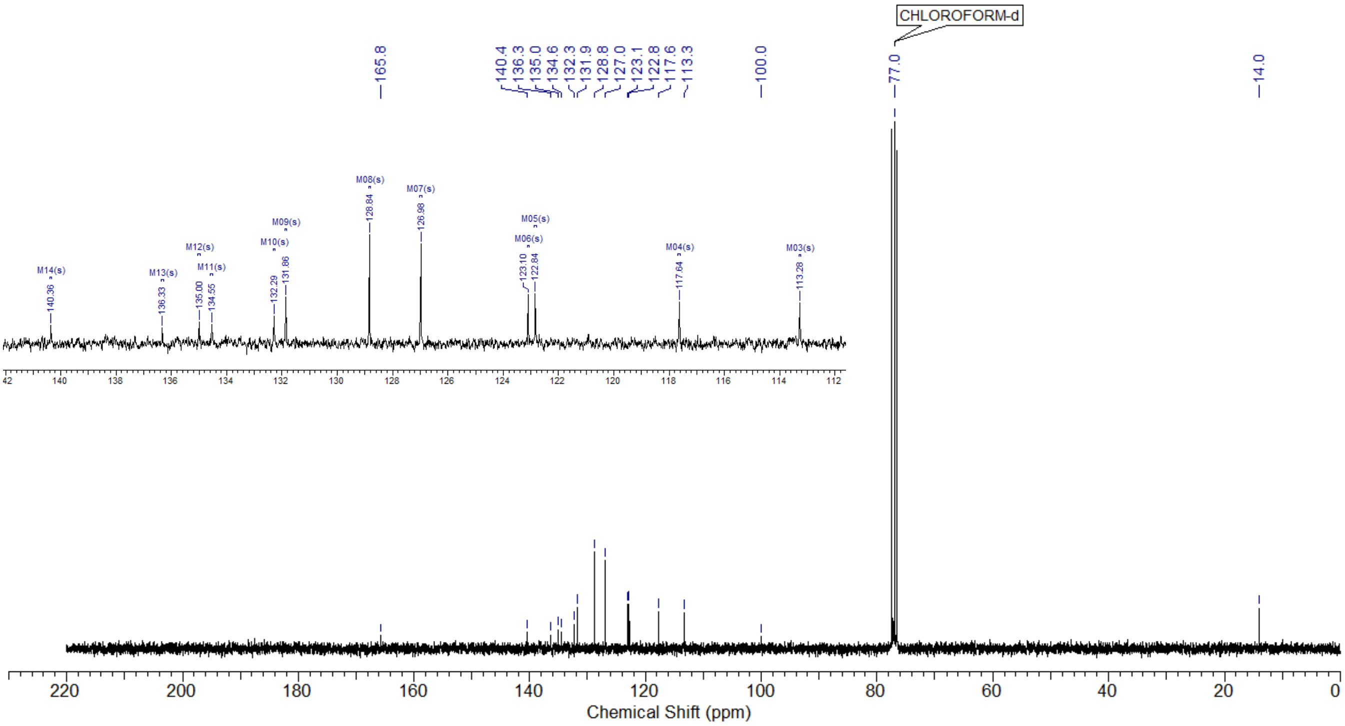

N-1,3-Benzodioxol-5-ylbenzamide (**3n**) [CAS 97631-47-5]

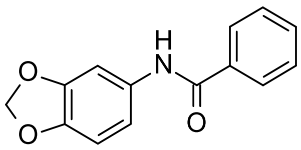

^{1}H NMR (300 MHz, $CDCl_3$):

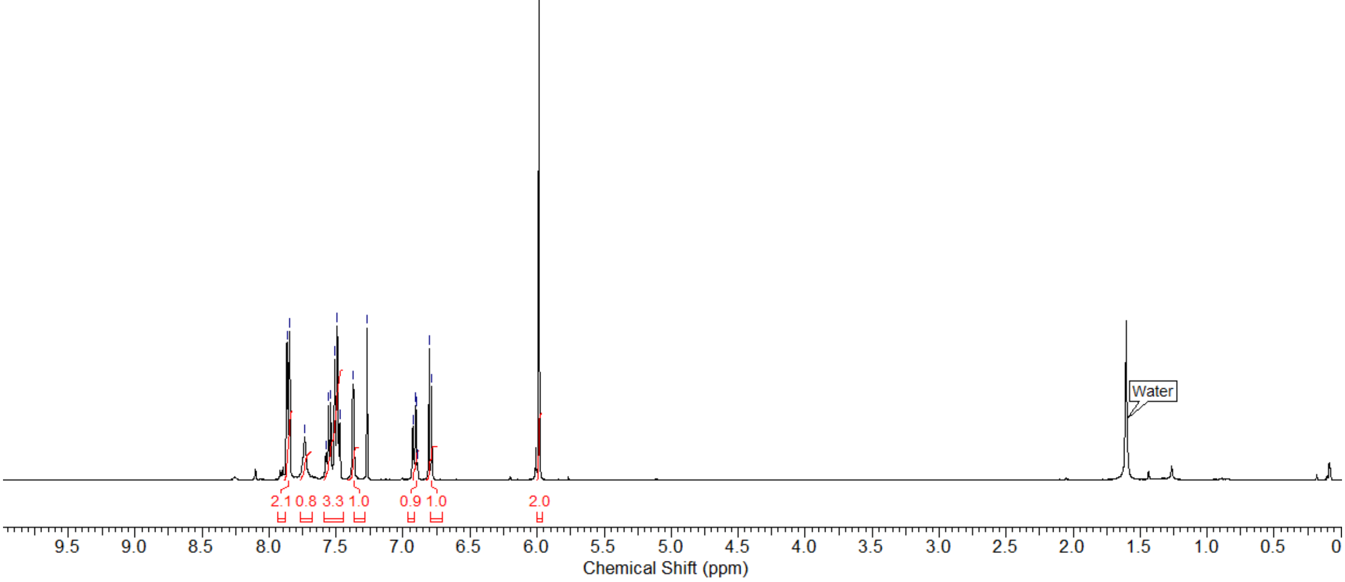

^{13}C NMR (75 MHz, $CDCl_3$):

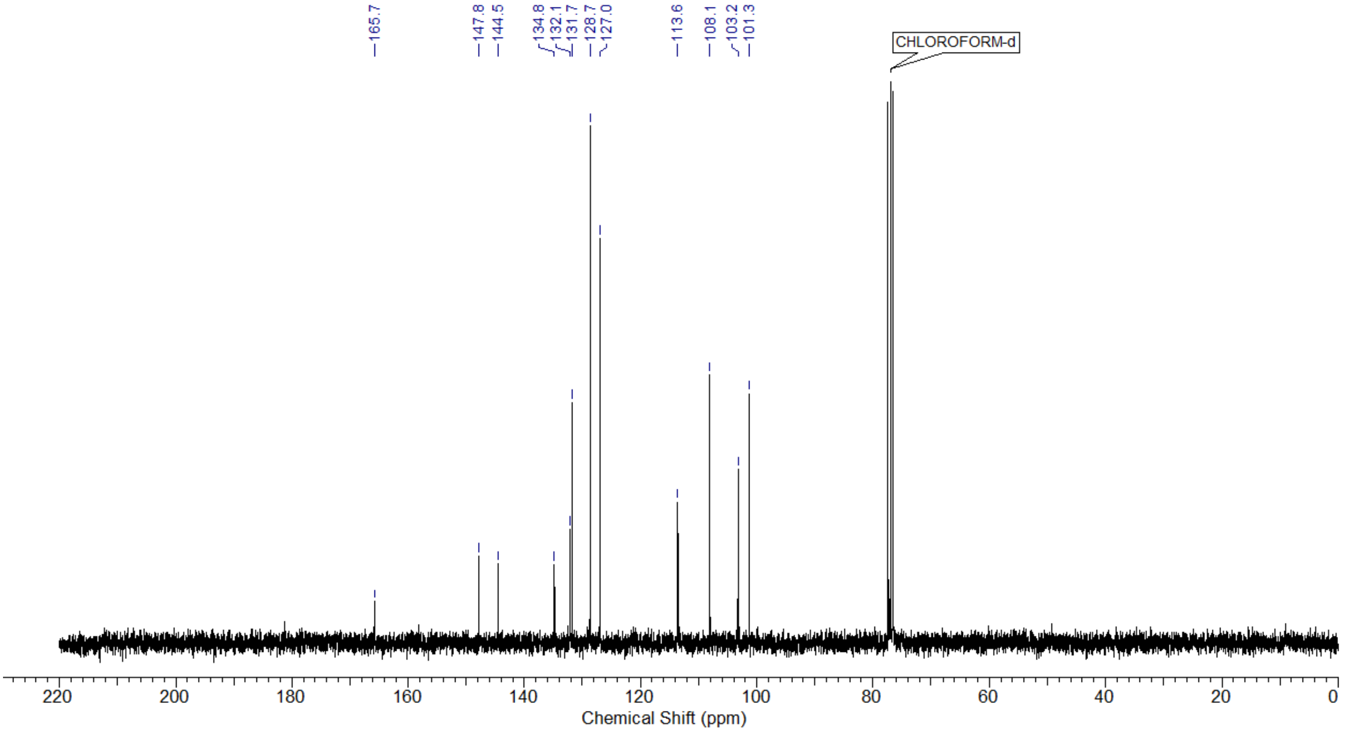

N-(4-Methoxyphenyl)-4-(trifluoromethyl)benzamide (**3o**) *[CAS 447431-35-8]*

^{1}H NMR (300 MHz, DMSO-d_6):

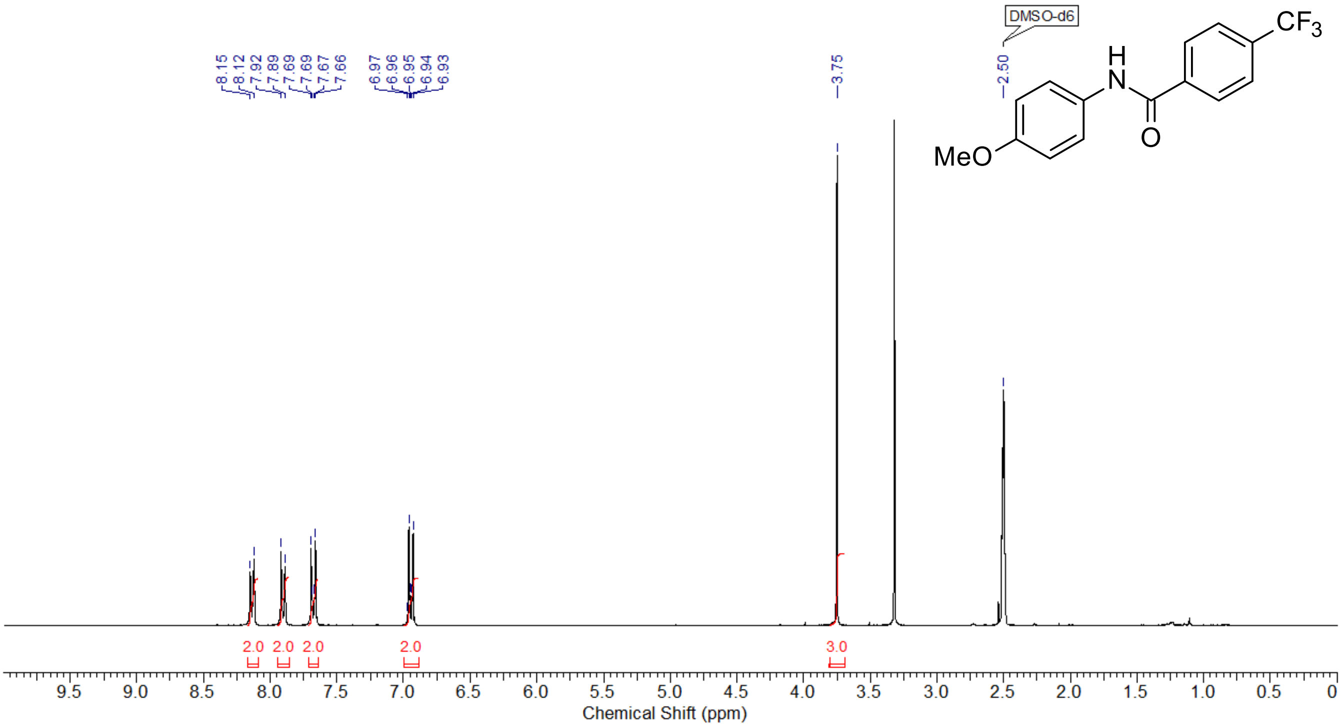

^{13}C NMR (75 MHz, DMSO-d_6):

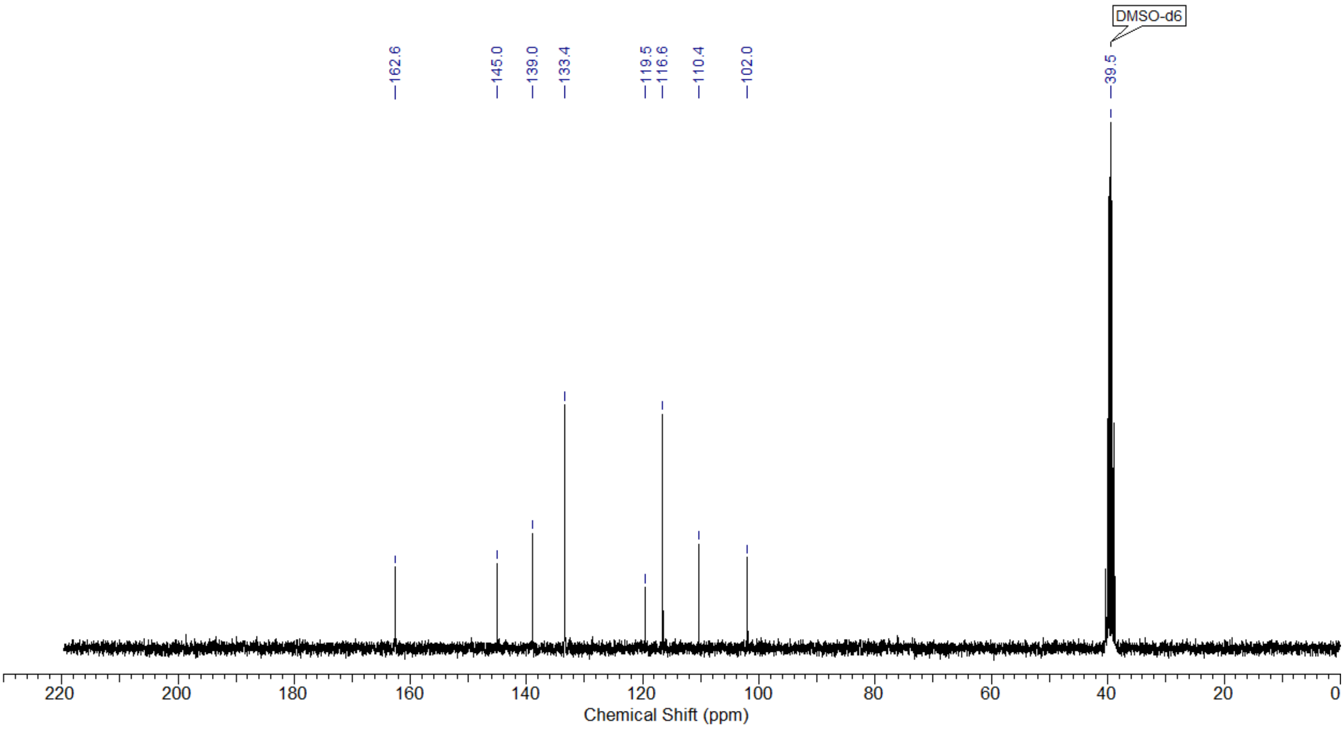

^{19}F NMR (76 MHz, DMSO-d6, $C_6H_4F_2$):

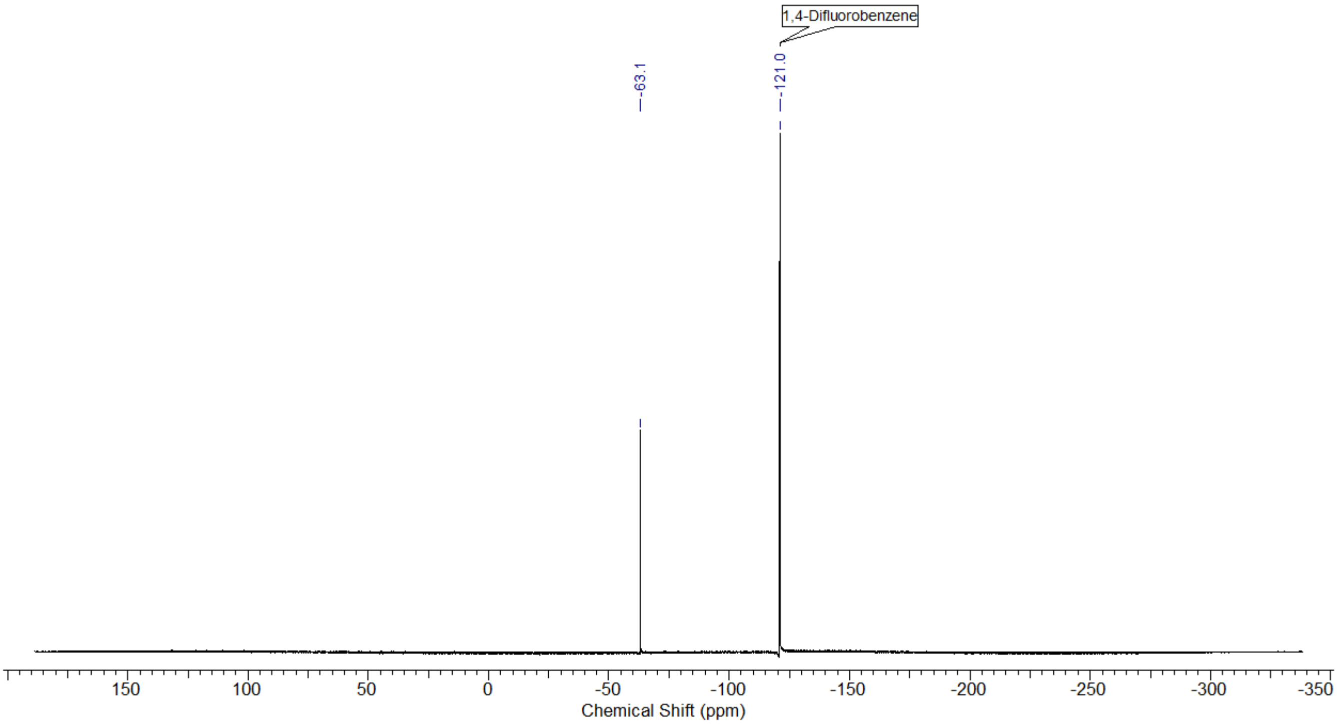

<u>*N*-[4-(Trifluoromethyl)phenyl]-2-furancarboxamide (**3p**)</u> [CAS 25617-46-3]

^{1}H NMR (300 MHz, $CDCl_3$):

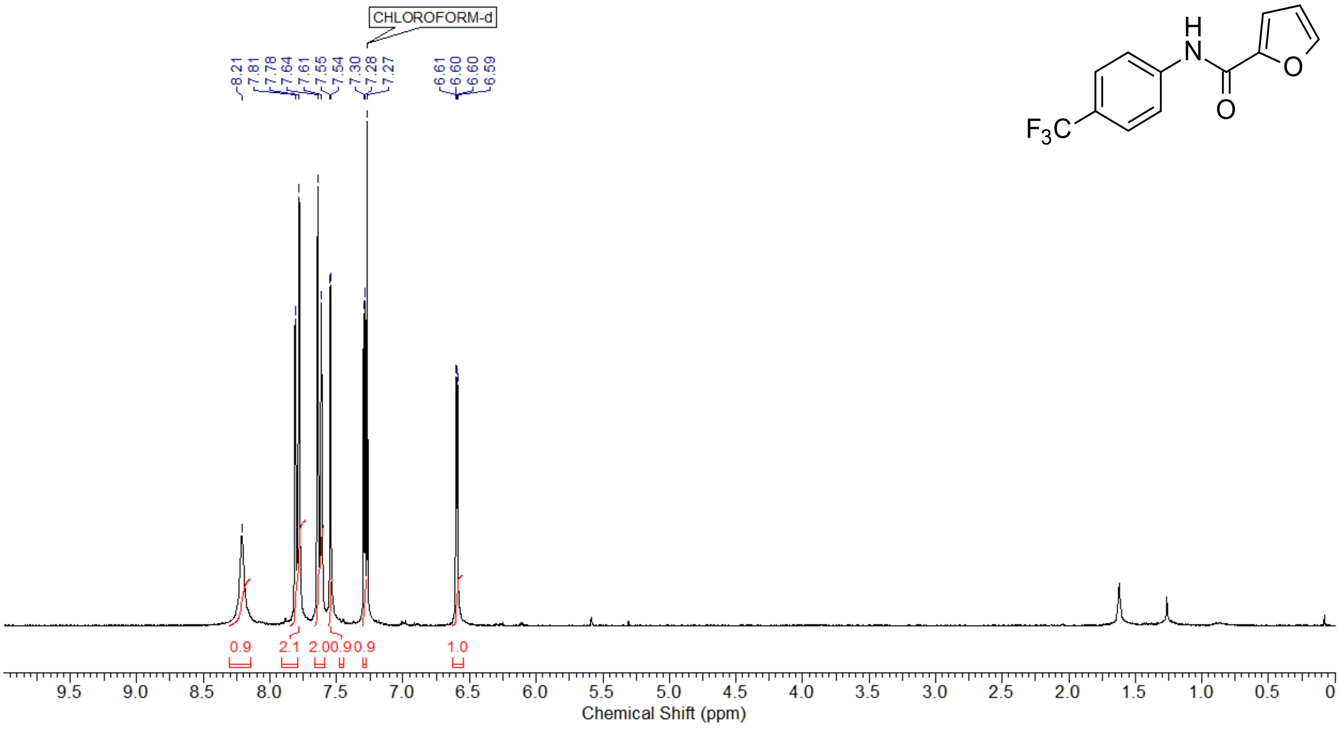

^{13}C NMR (75 MHz, $CDCl_3$):

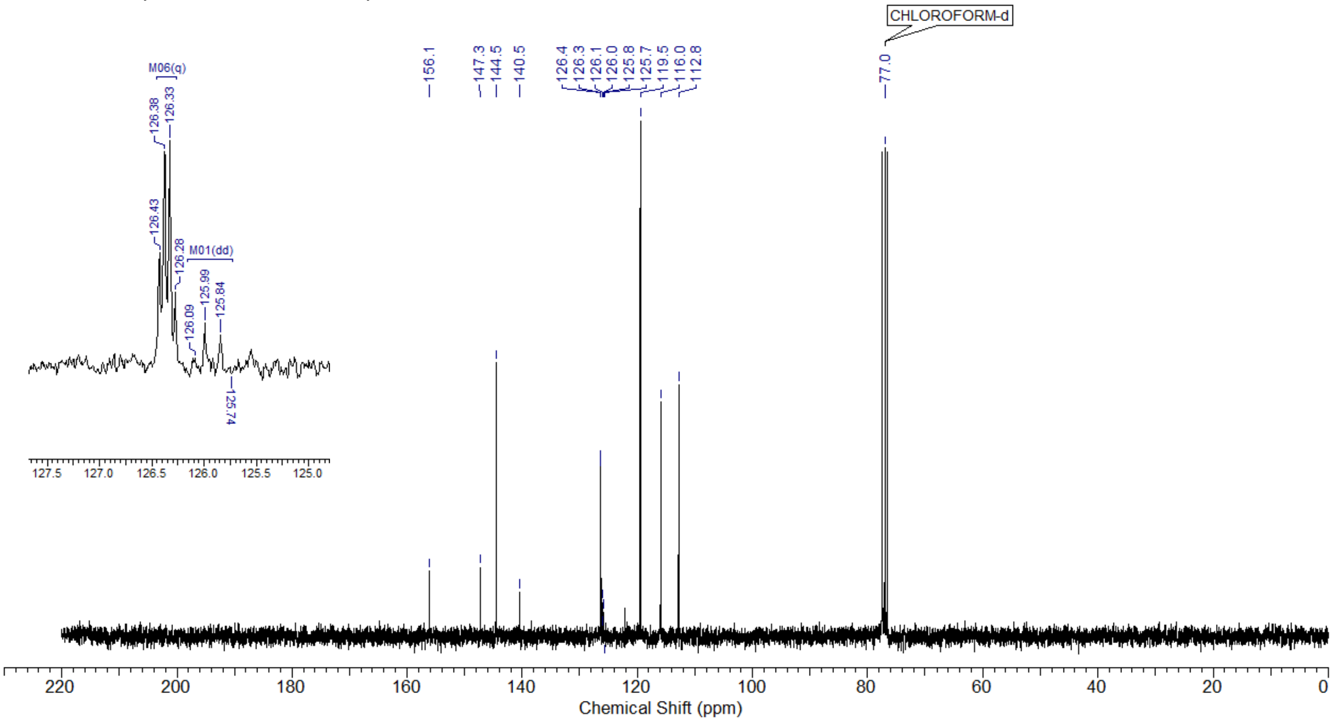

^{19}F NMR (76 MHz, $CDCl_3$, $C_6H_4F_2$):

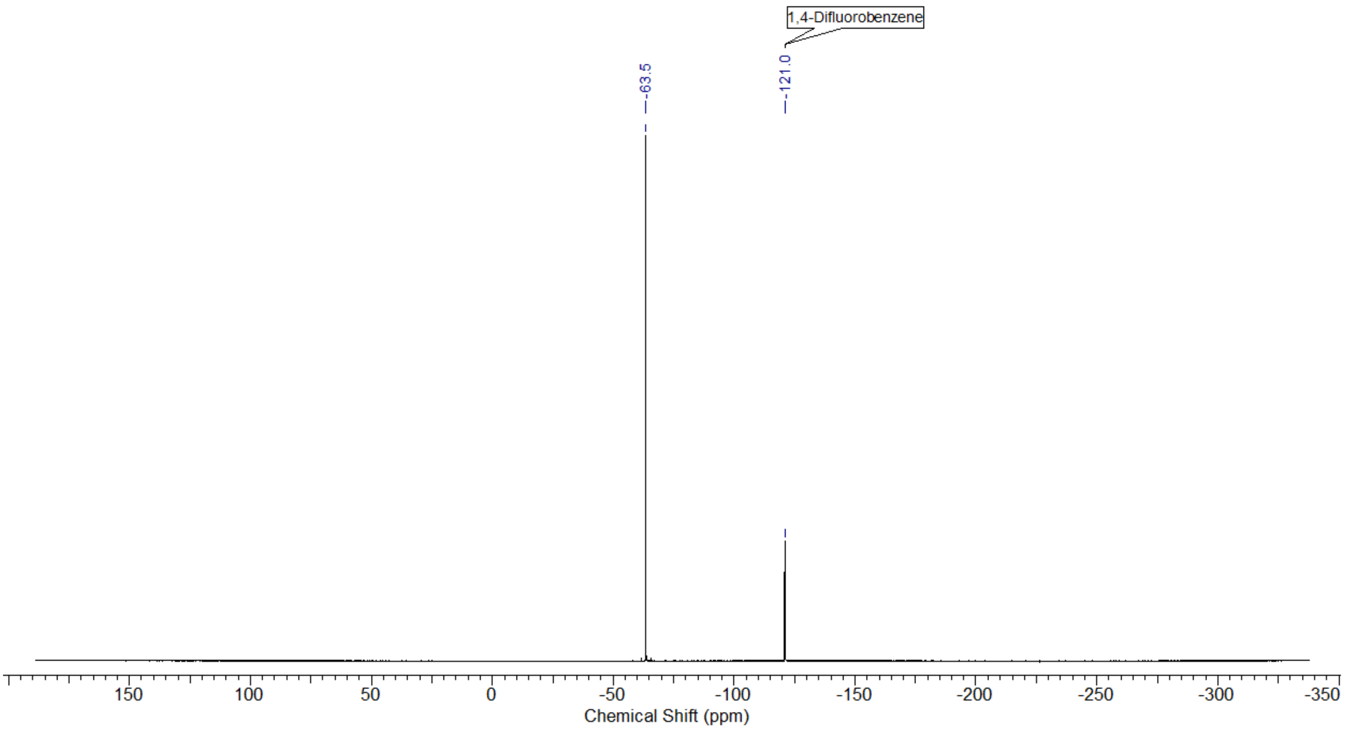

N-[4-(Trifluoromethyl)phenyl]-2-thiopheneacetamide (**3q**) [CAS 639810-25-6]

^{1}H NMR (300 MHz, $CDCl_3$):

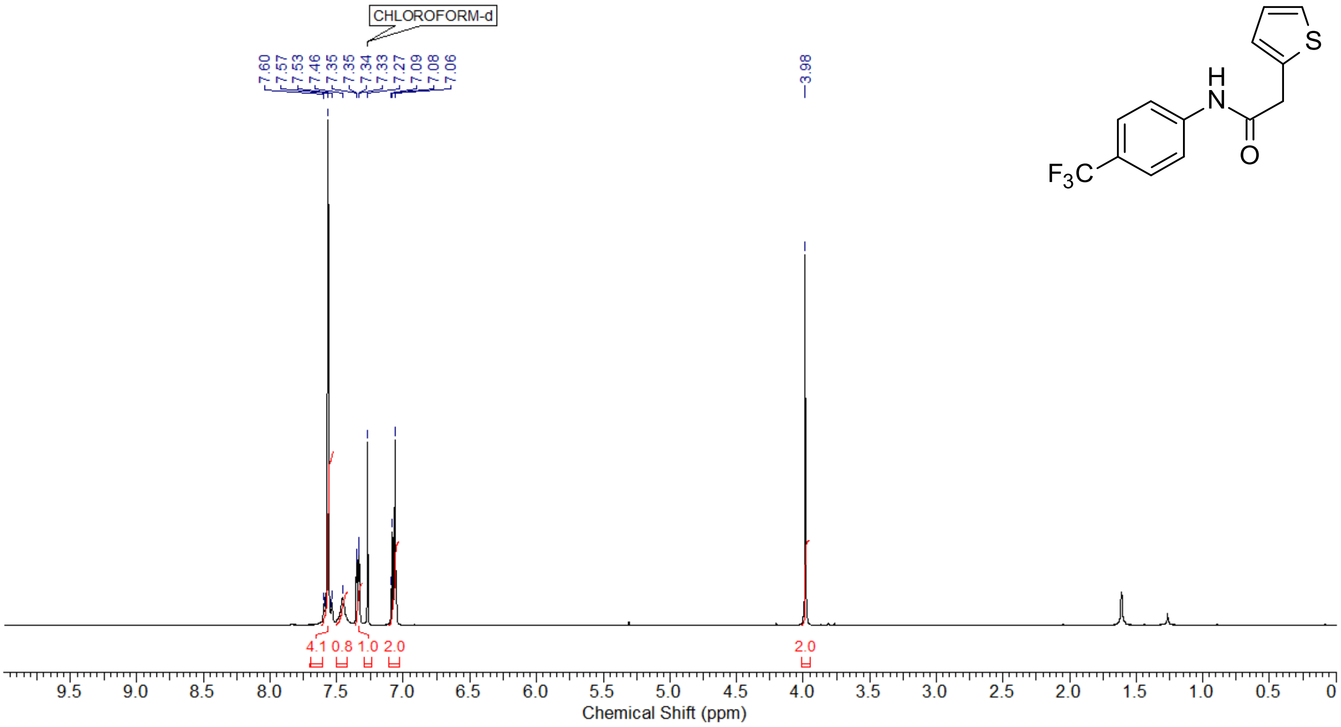

^{13}C NMR (75 MHz, $CDCl_3$):

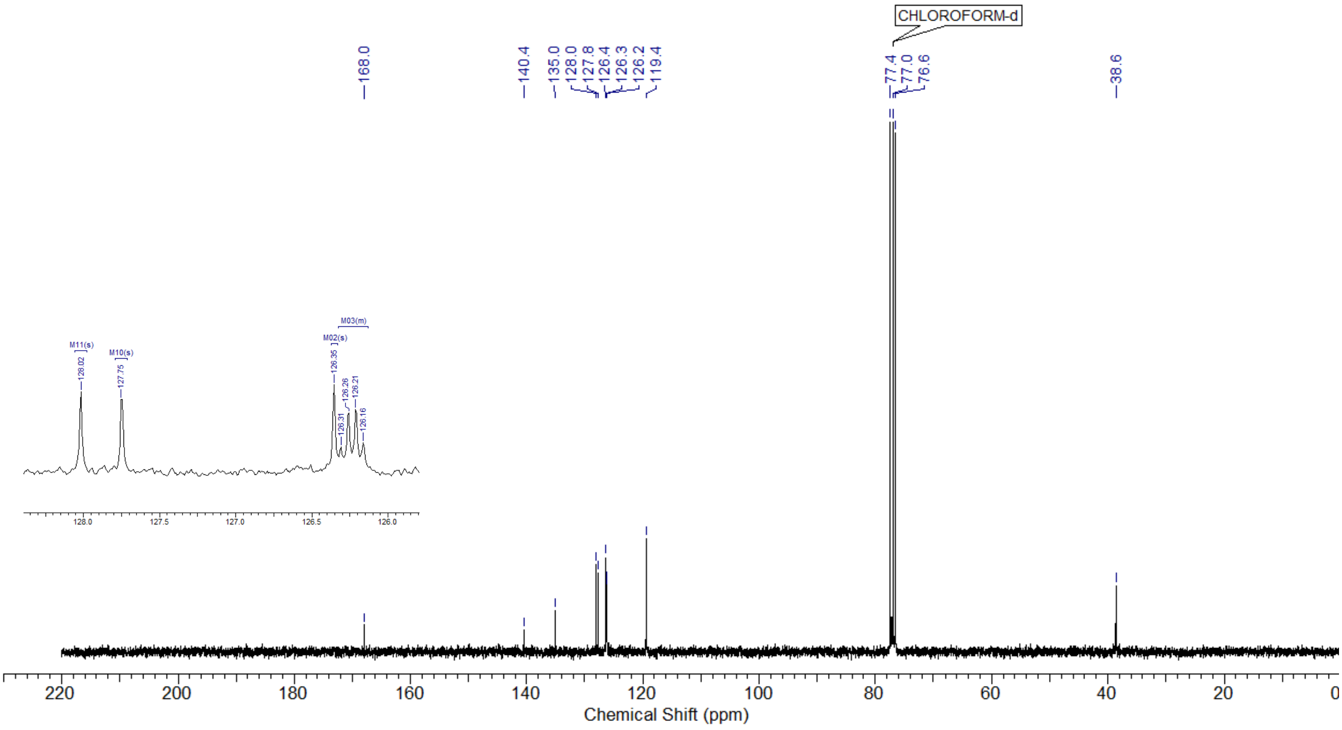

^{19}F NMR (76 MHz, $CDCl_3$,$C_6H_4F_2$):

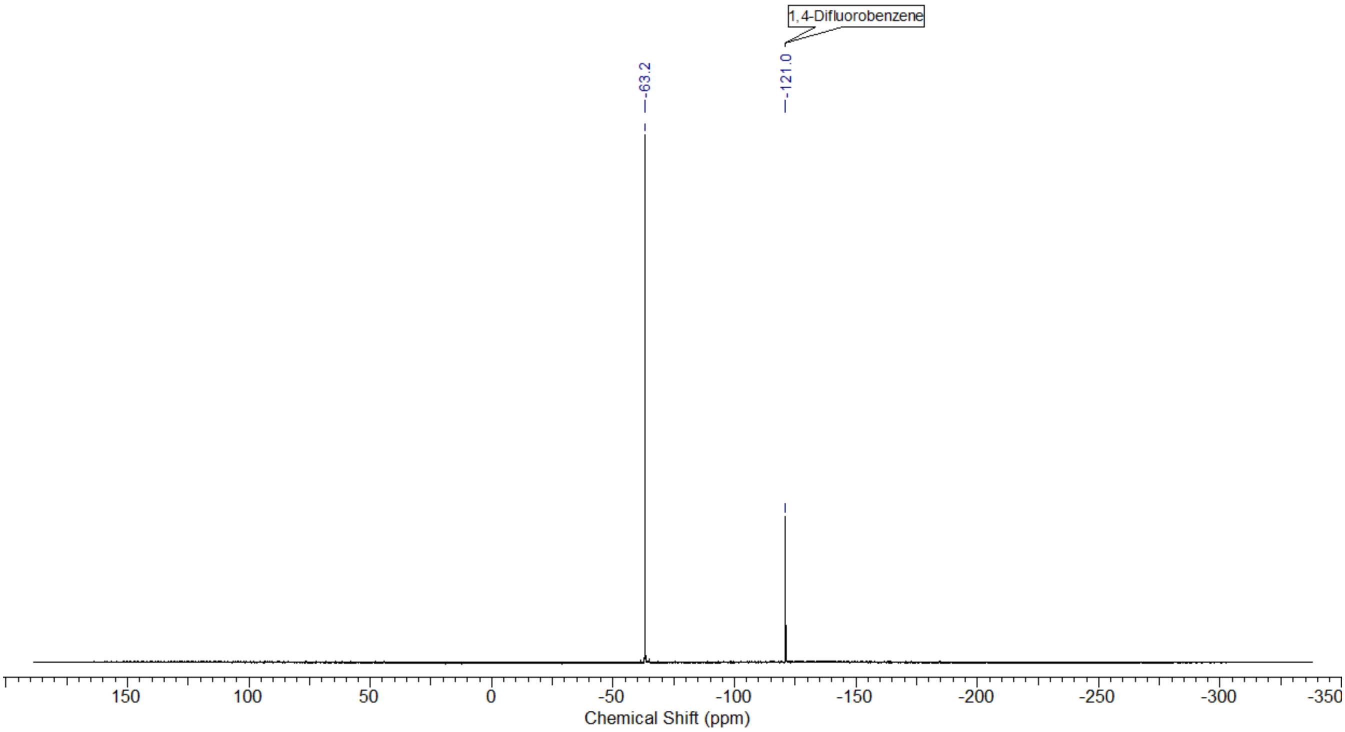

N-[4-(Trifluoromethyl)phenyl]-2-pyridineacetamide (**3r**) [CAS 1790280-85-1]

^{1}H NMR (300 MHz, $CDCl_3$):

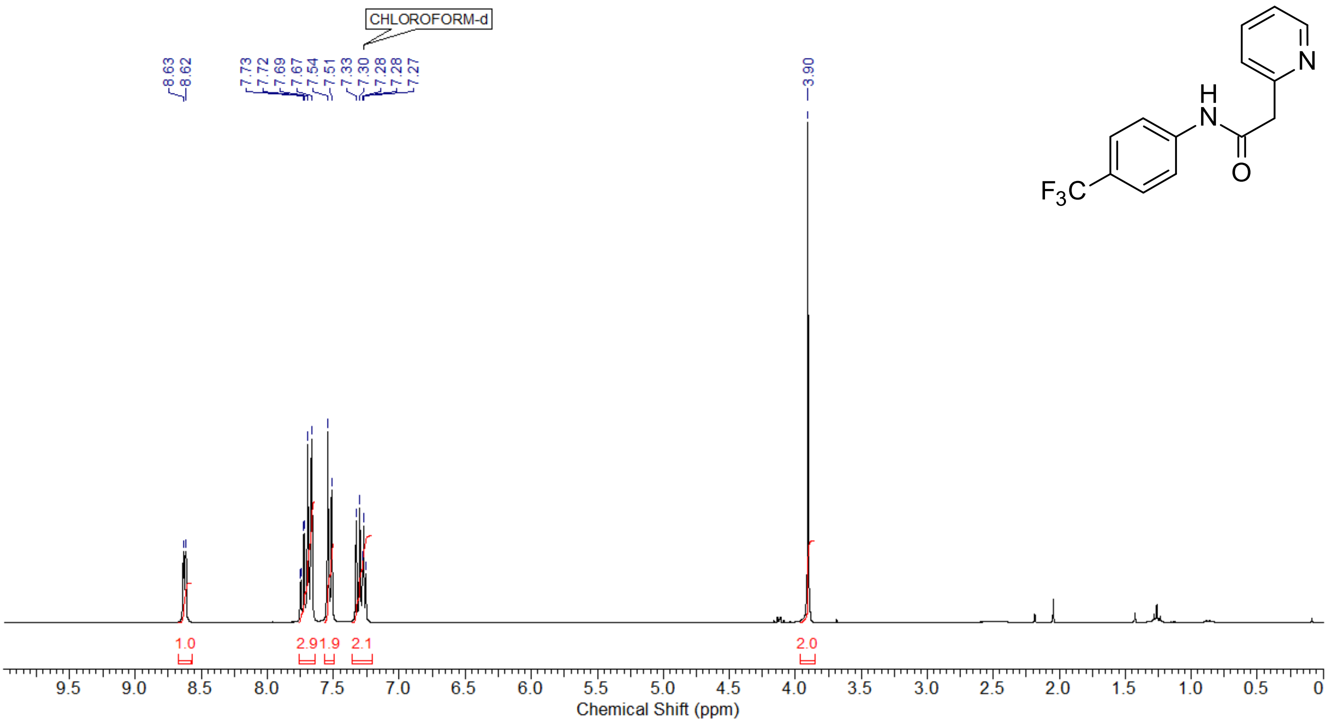

^{13}C NMR (75 MHz, $CDCl_3$):

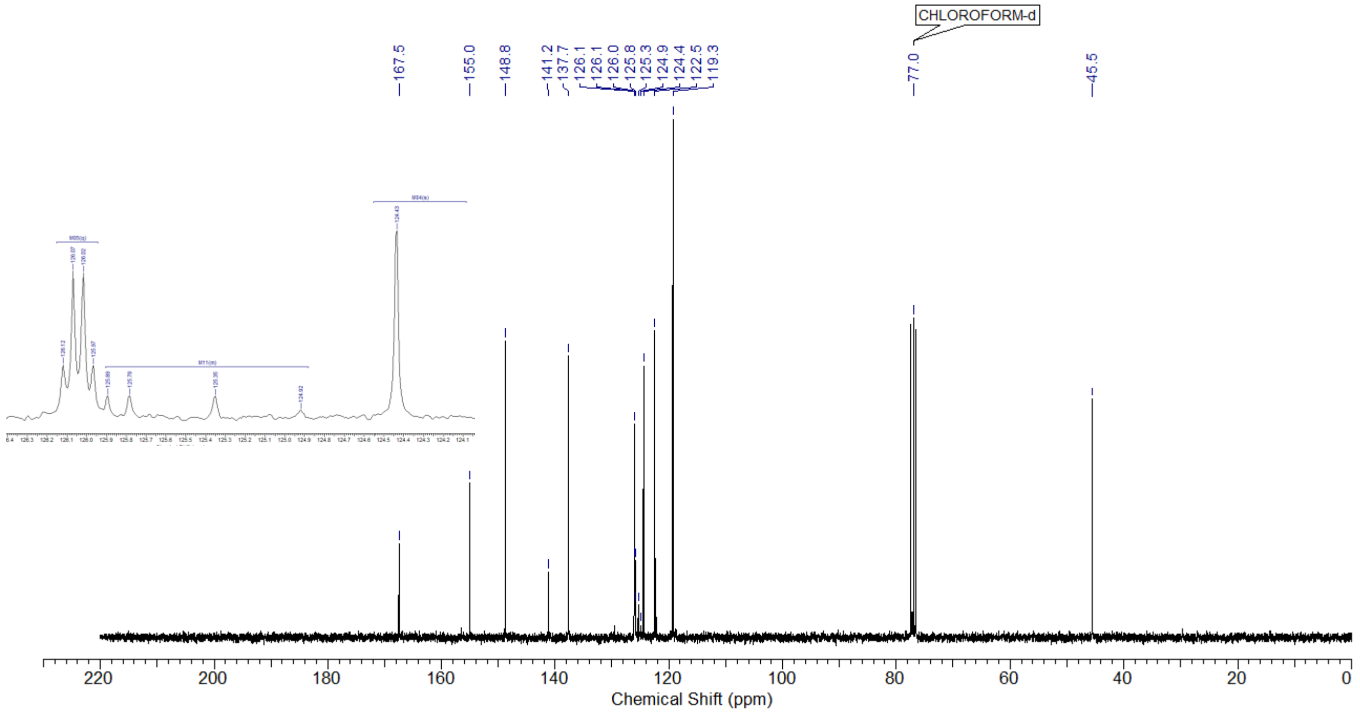

^{19}F NMR (76 MHz, $CDCl_3$, $C_6H_4F_2$):

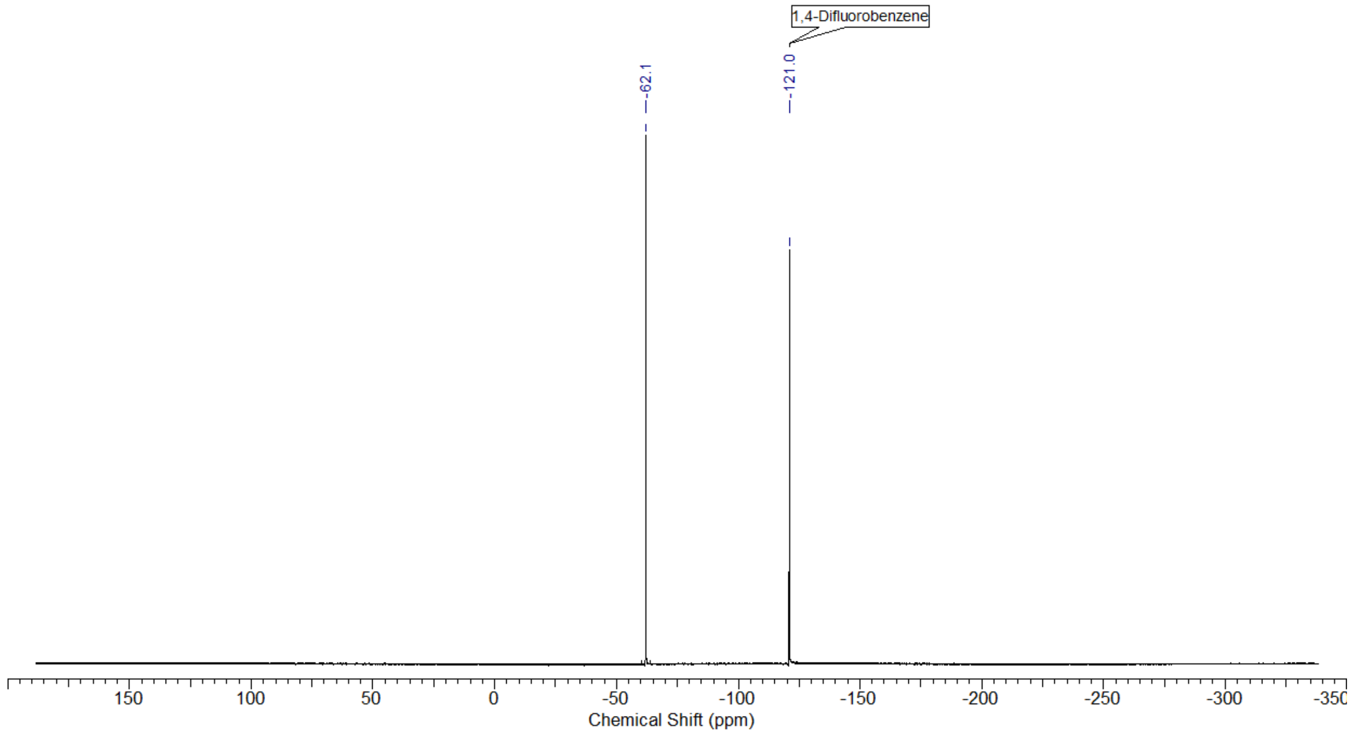

N-[4-(Trifluoromethyl)phenyl]cyclopropanecarboxamide *(***3s***) [CAS 23845-06-9]*

^{1}H NMR (300 MHz, DMSO-d$_6$):

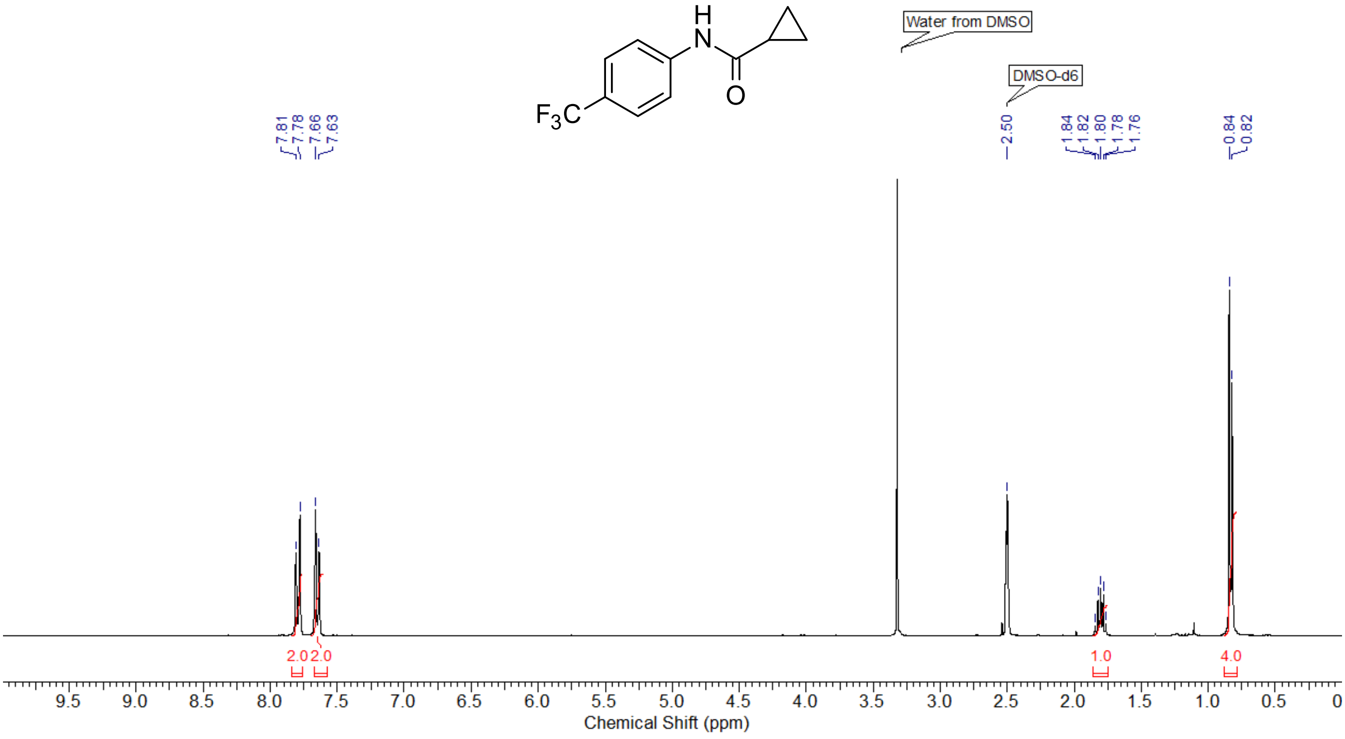

^{13}C NMR (75 MHz, DMSO-d$_6$):

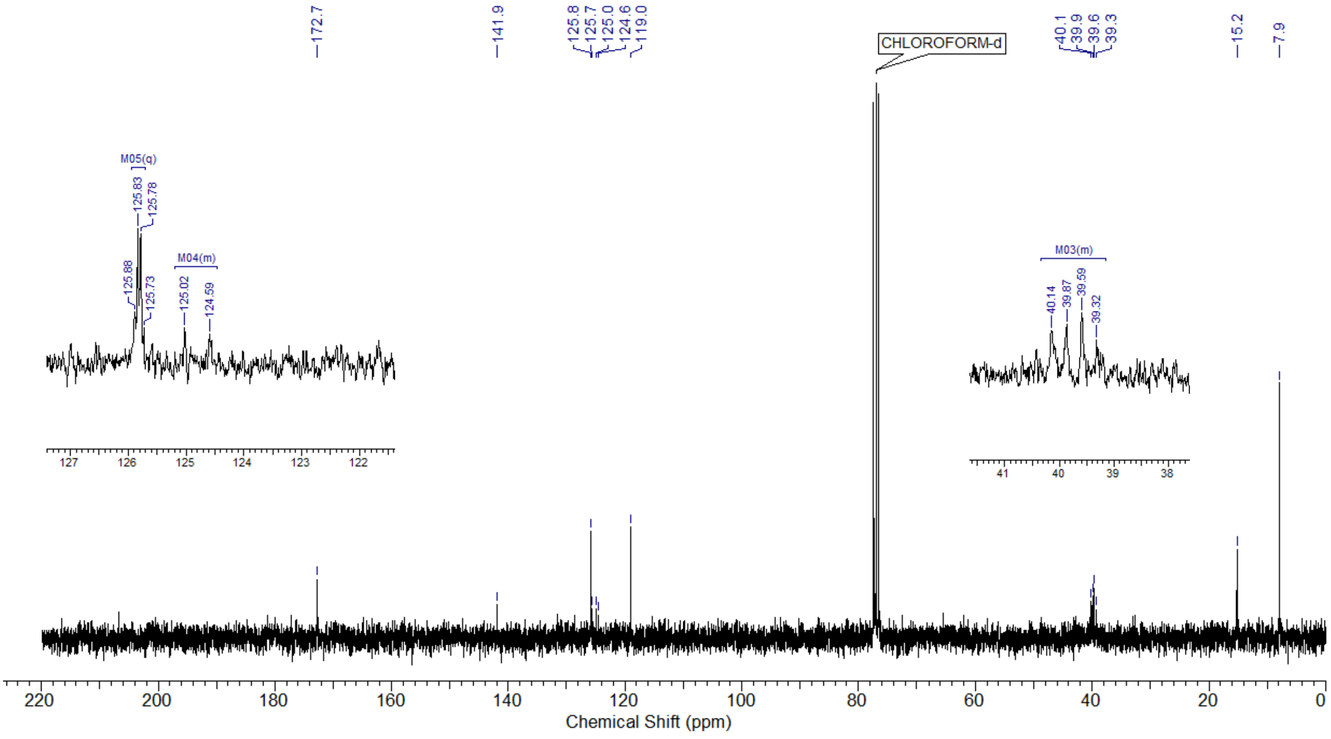

^{19}F NMR (76 MHz, DMSO-d$_6$,$C_6H_4F_2$):

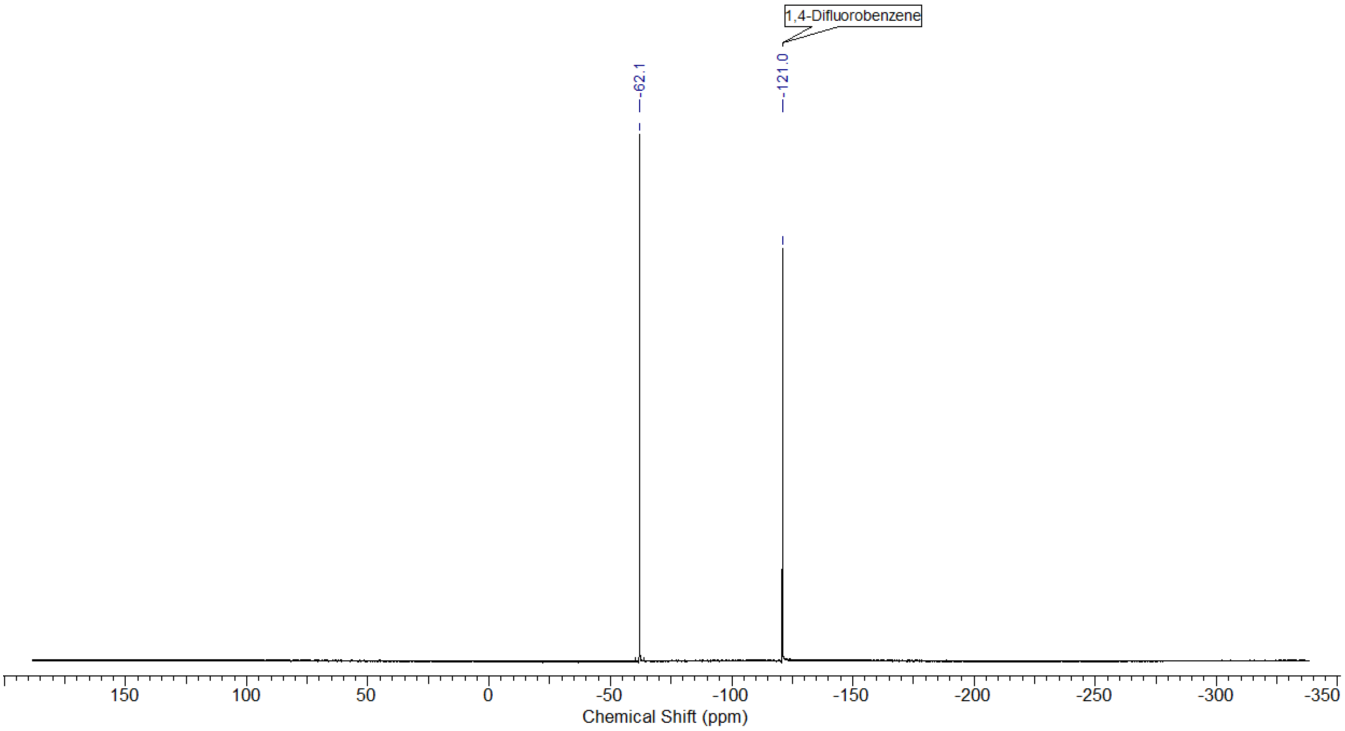

N-[4-(Trifluoromethoxy)phenyl]benzenecarboximidamide (**4a**) [CAS 1266333-17-8]

^{1}H NMR (300 MHz, *DMSO-d*$_6$):

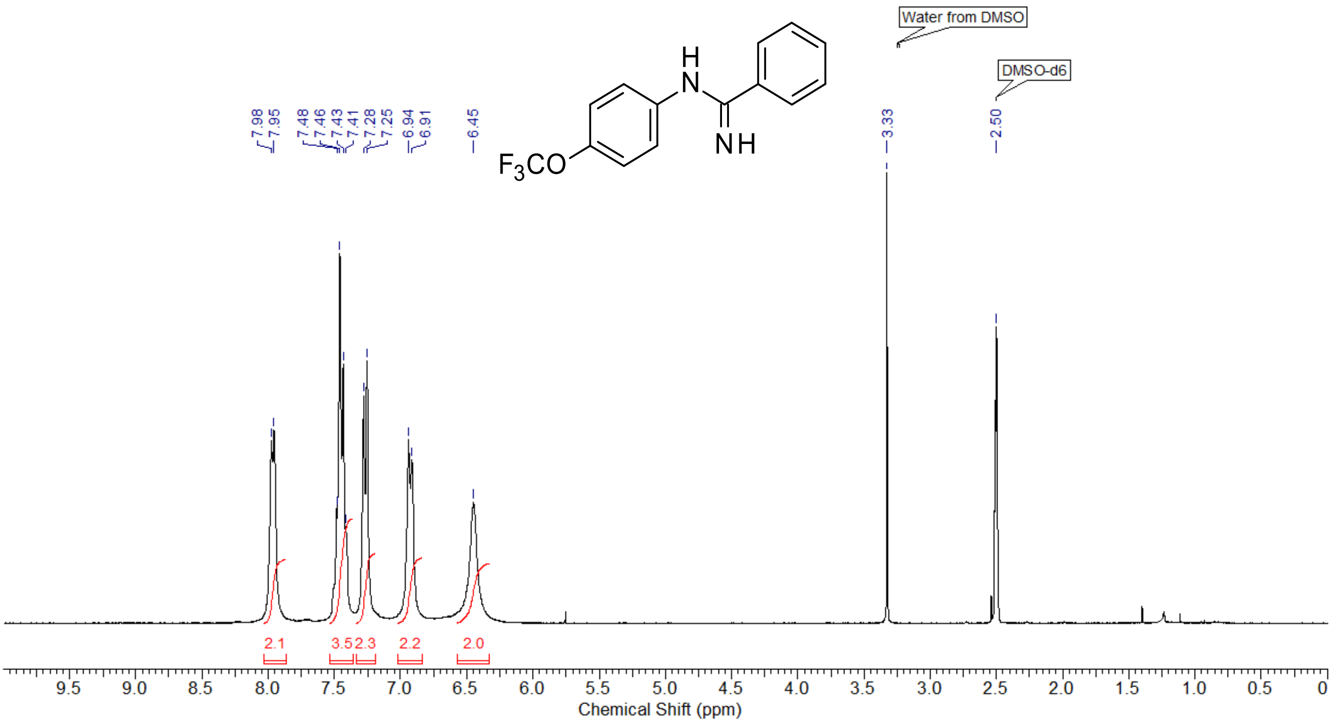

^{13}C NMR (75 MHz, DMSO-*d*$_6$):

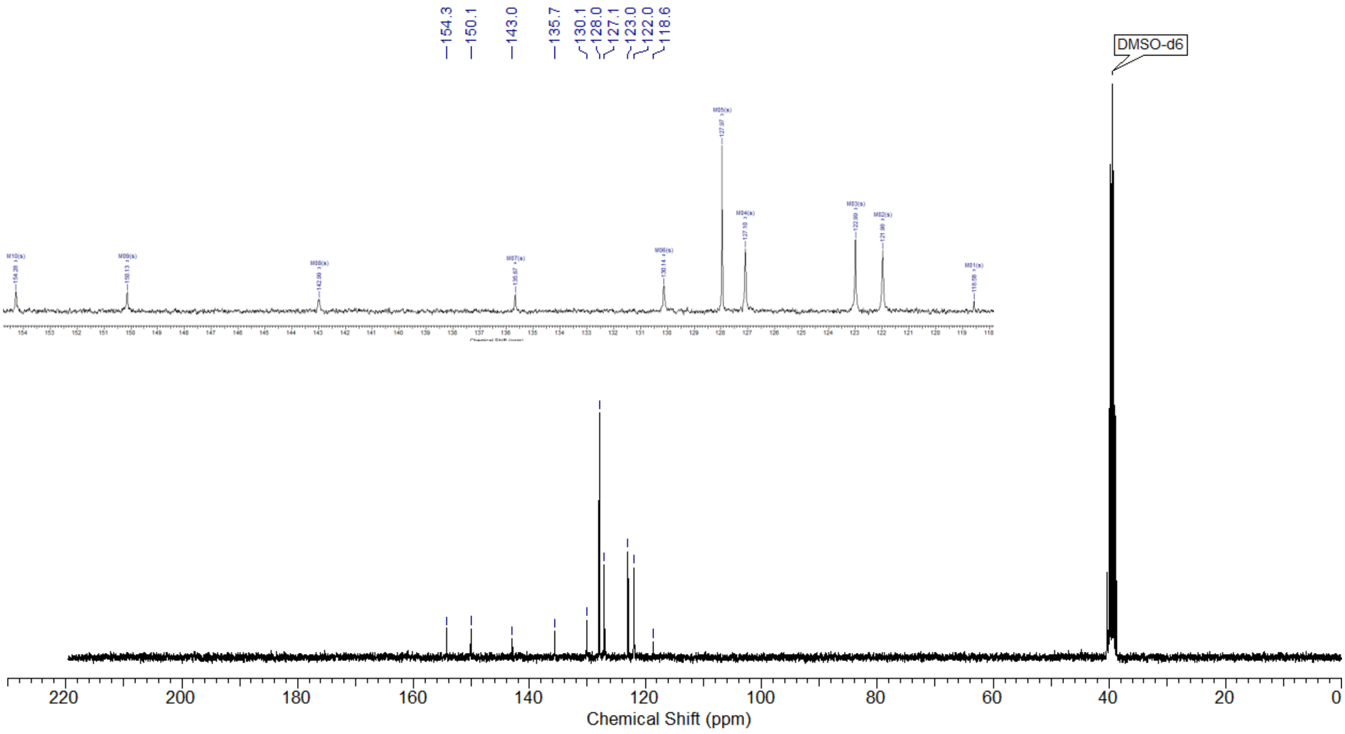

^{19}F NMR (76 MHz, DMSO-d_6, $C_6H_4F_2$):

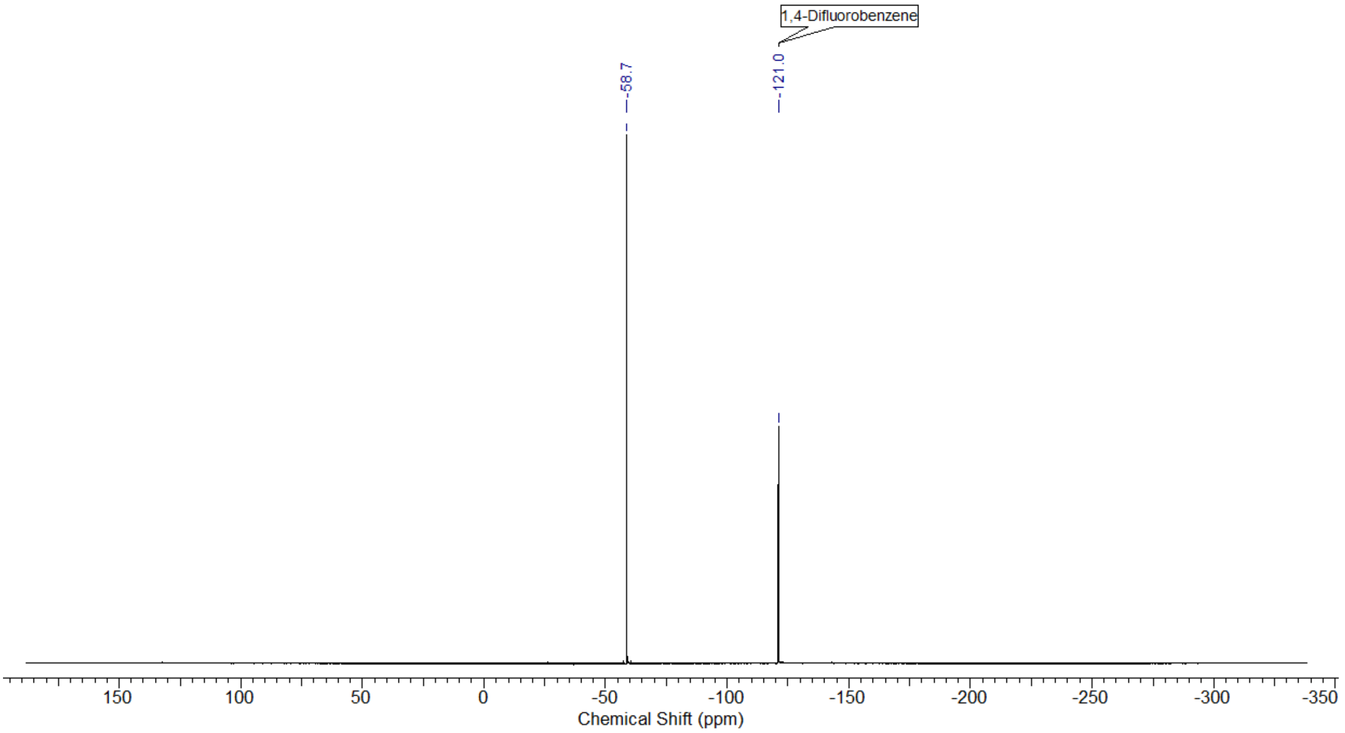

<u>*N*-(4-Acetylphenyl)benzenecarboximidamide</u> *(**4b**) [CAS 2366186-66-3]*

^{1}H NMR (300 MHz, *DMSO-d*$_6$):

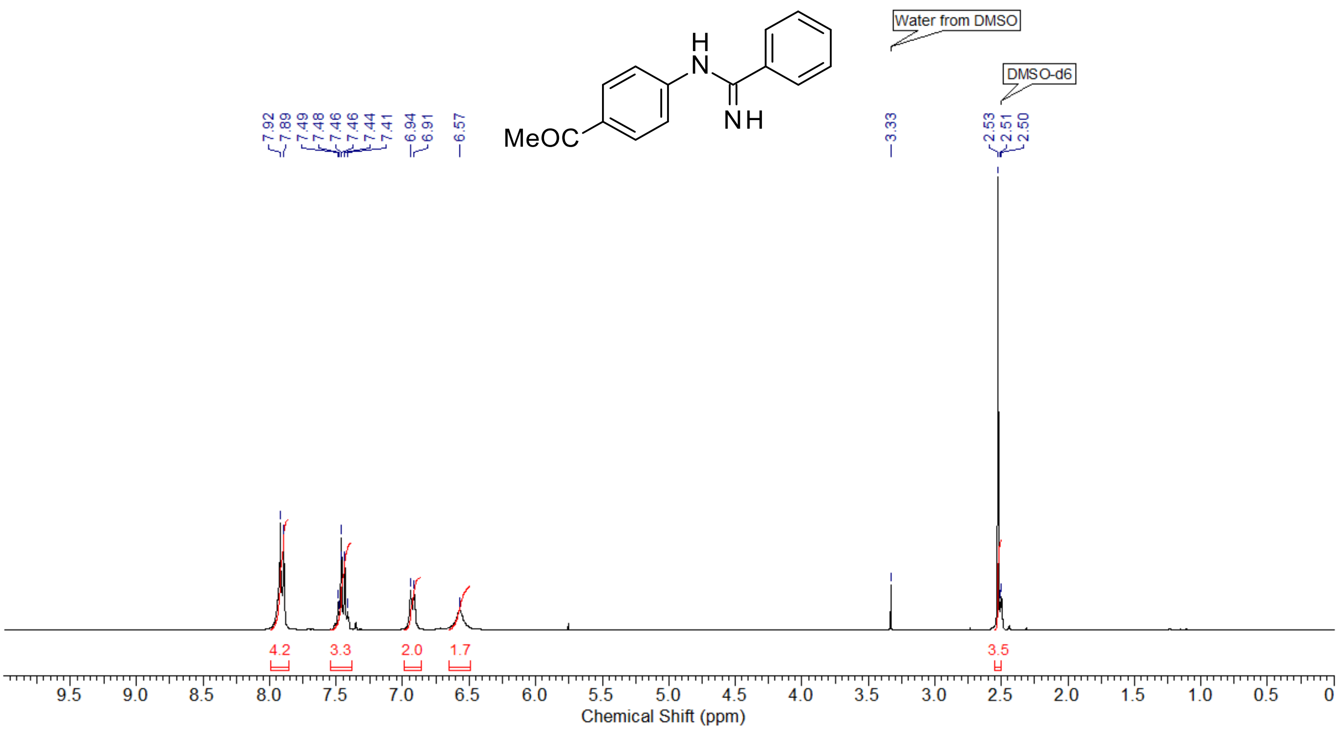

^{13}C NMR (75 MHz, DMSO-*d*$_6$):

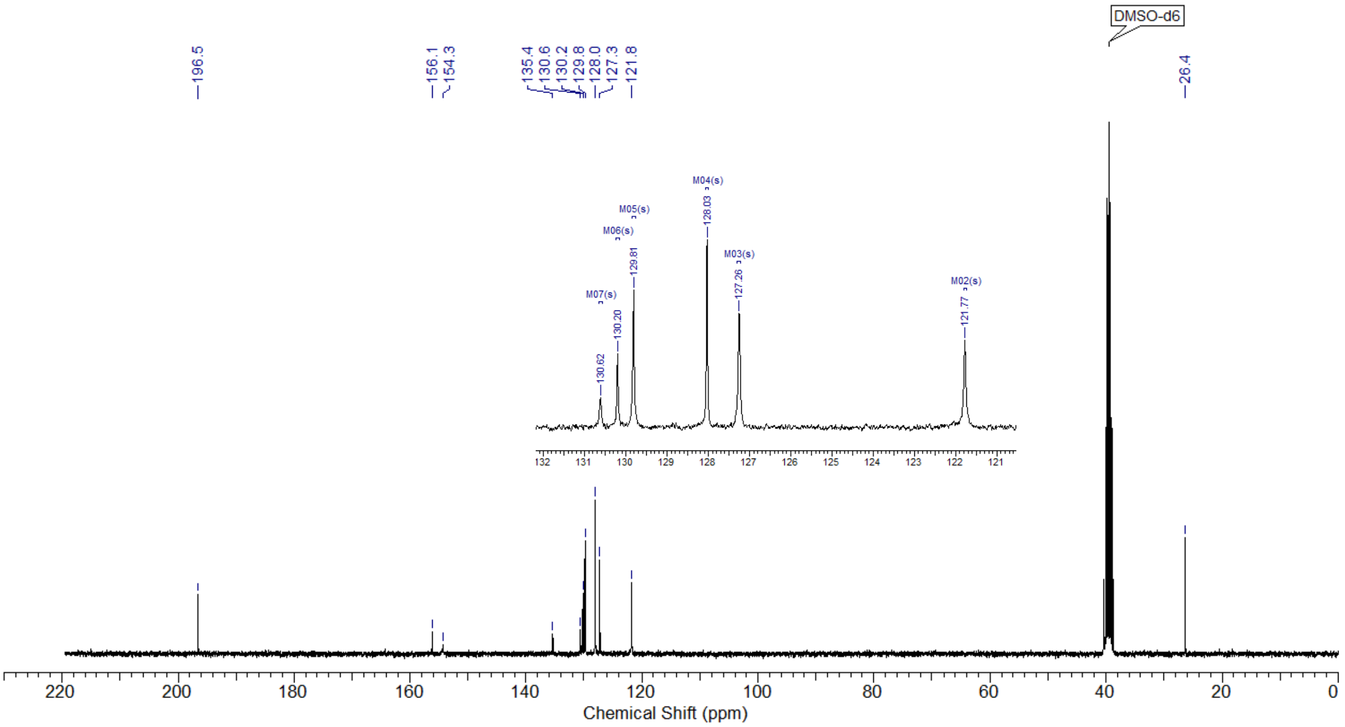

<u>*N*-(4-trimethylsilylphenyl)benzamidine</u> (**4c**)

^{1}H NMR (300 MHz, *DMSO-d*6):

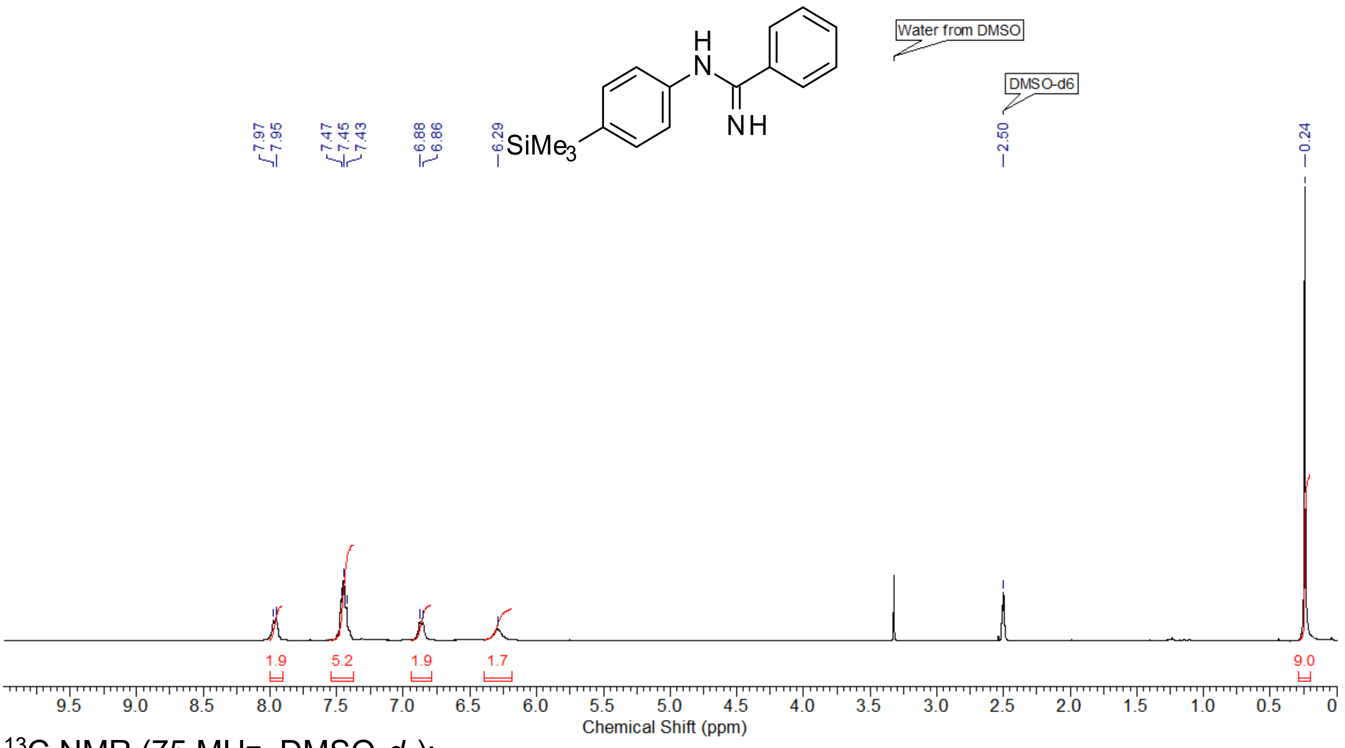

^{13}C NMR (75 MHz, DMSO-*d*6):

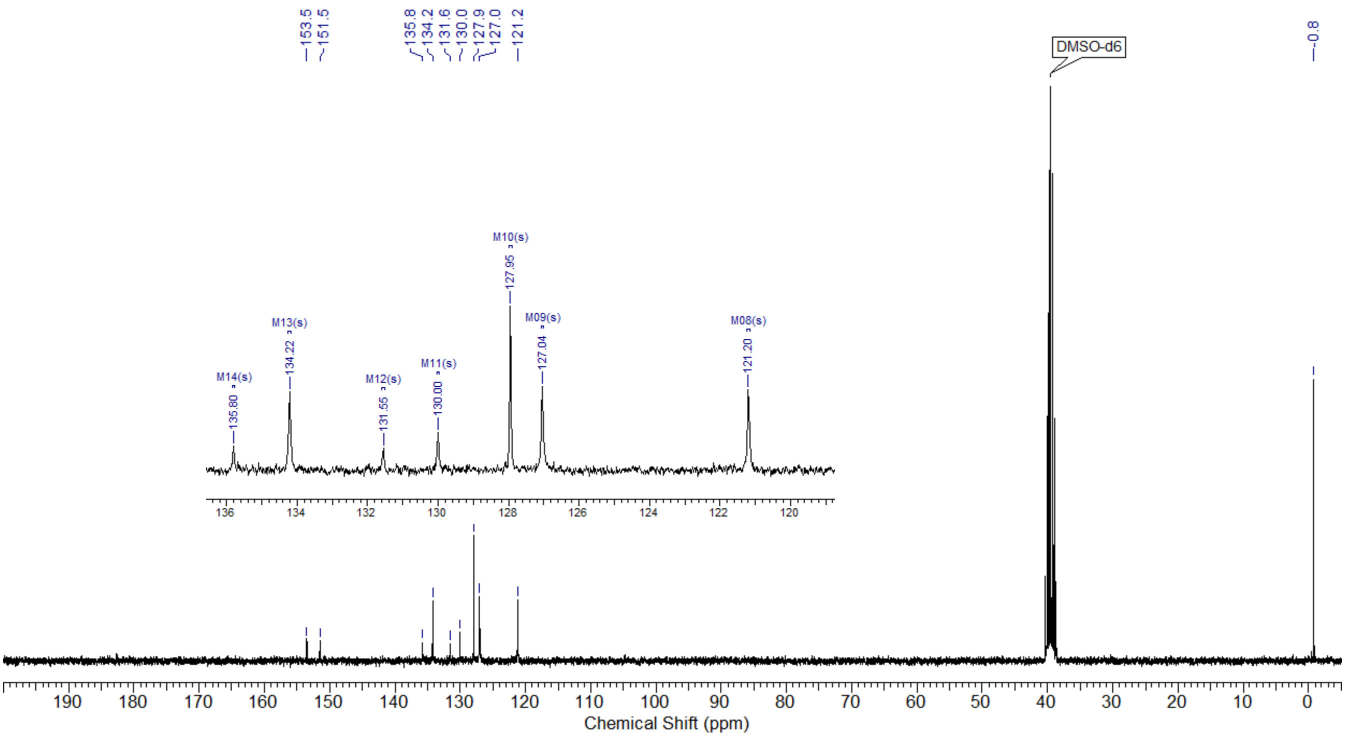

<u>*N*-(4-Methylsulfonylphenyl)benzenecarboximidamide</u> (**4d**) *[CAS 177662-40-7]*

^{1}H NMR (300 MHz, *DMSO-d*$_6$):

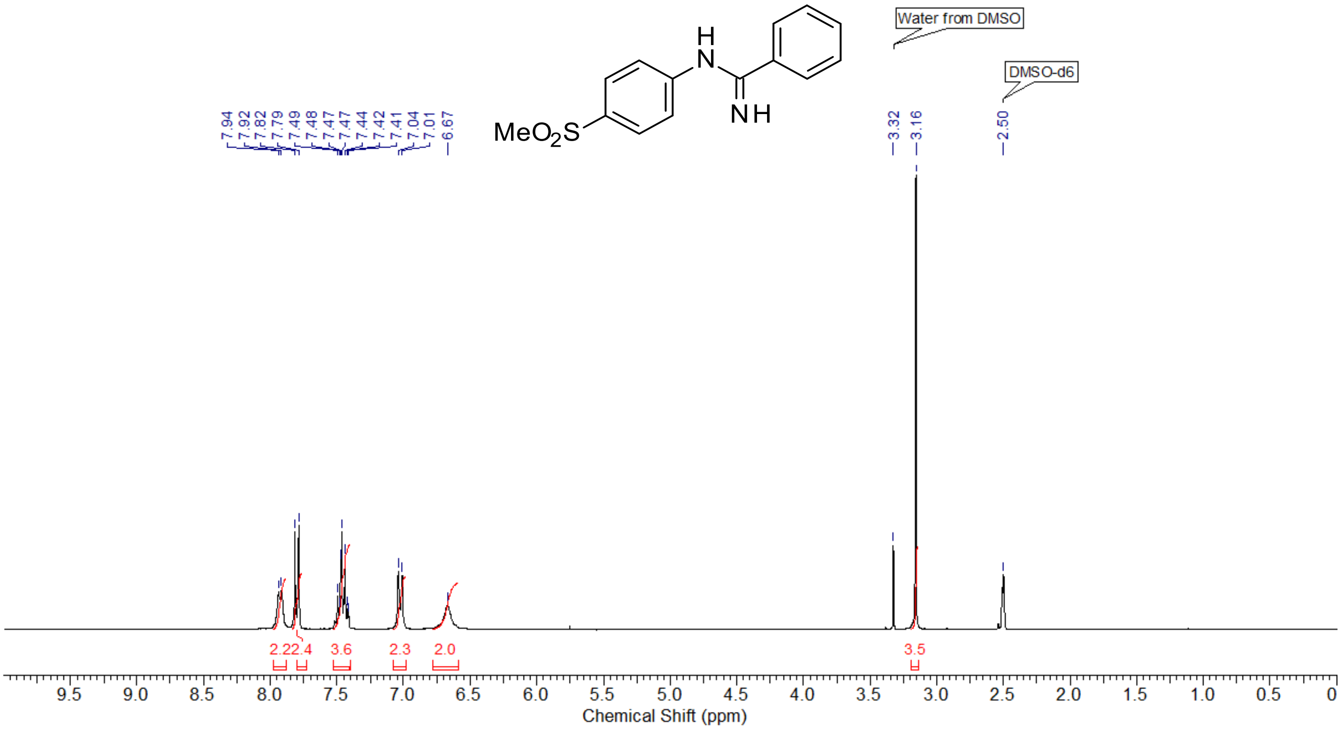

^{13}C NMR (75 MHz, DMSO-*d*$_6$):

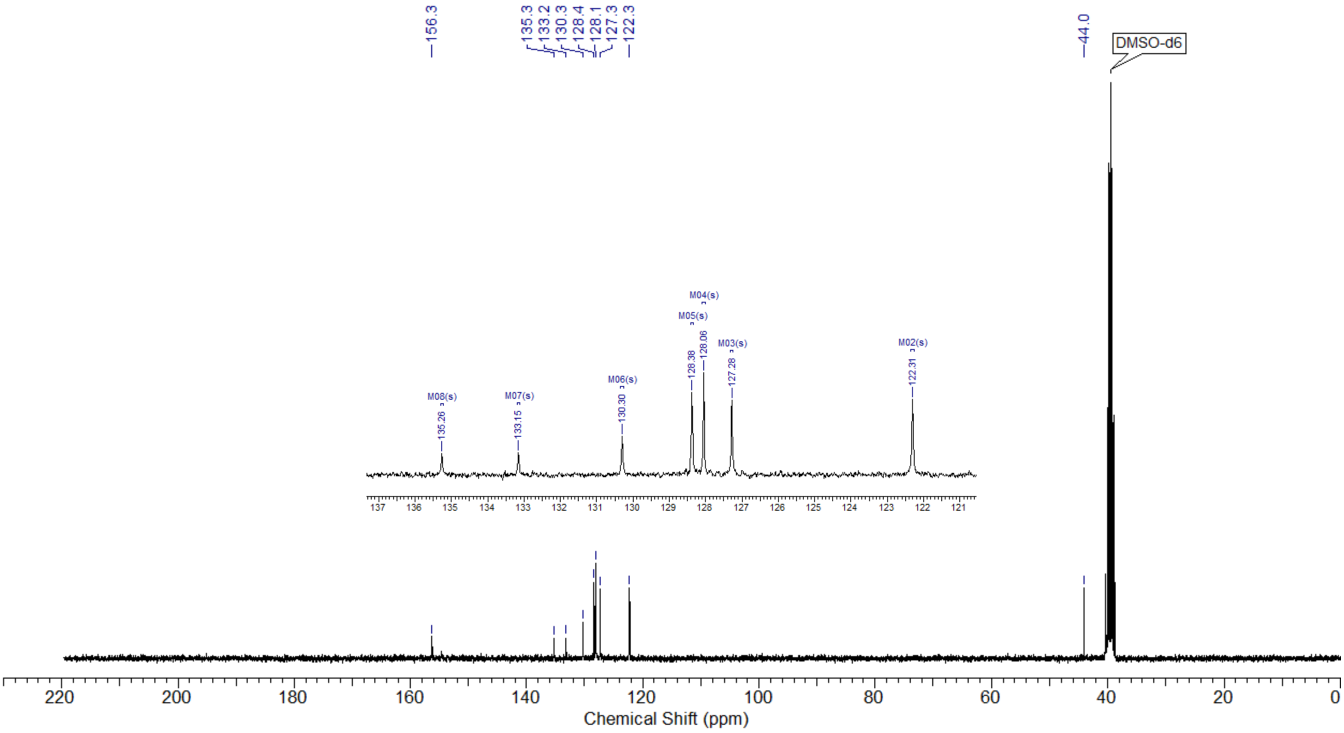

N-p-Tolylbenzamidine (**4e**) [CAS 1859-00-3]

^{1}H NMR (300 MHz, *DMSO-d*$_6$):

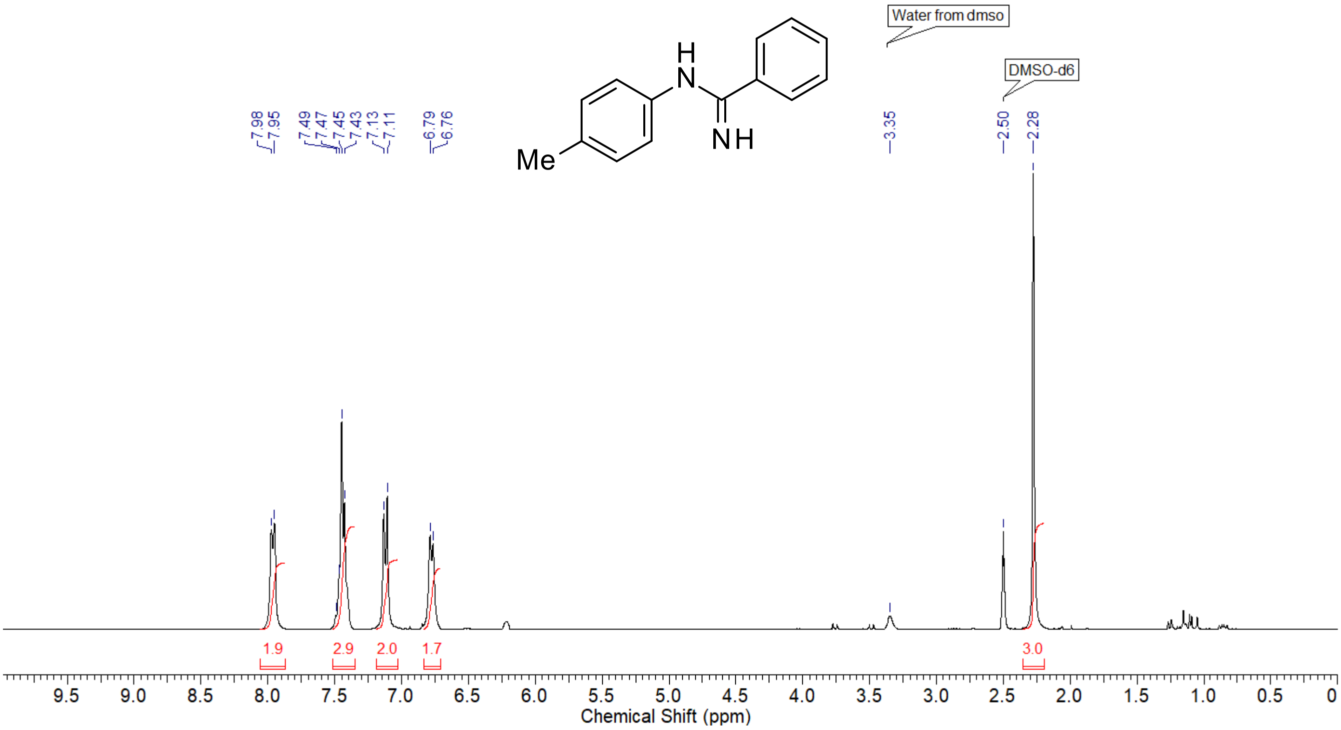

^{13}C NMR (75 MHz, DMSO-*d*$_6$):

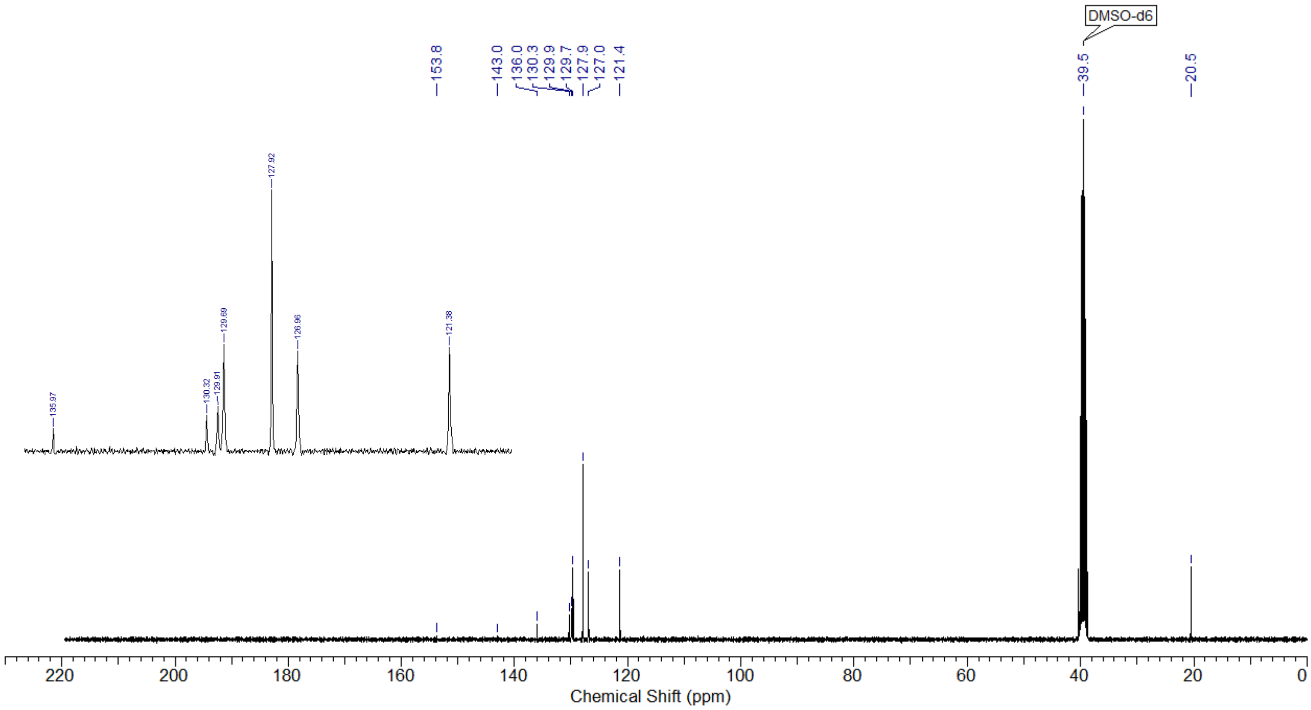

N-(6-methoxypyridazin-3-yl)benzamidine (**4f**) [CAS 39931-61-8]

^{1}H NMR (300 MHz, *DMSO-d*$_6$):

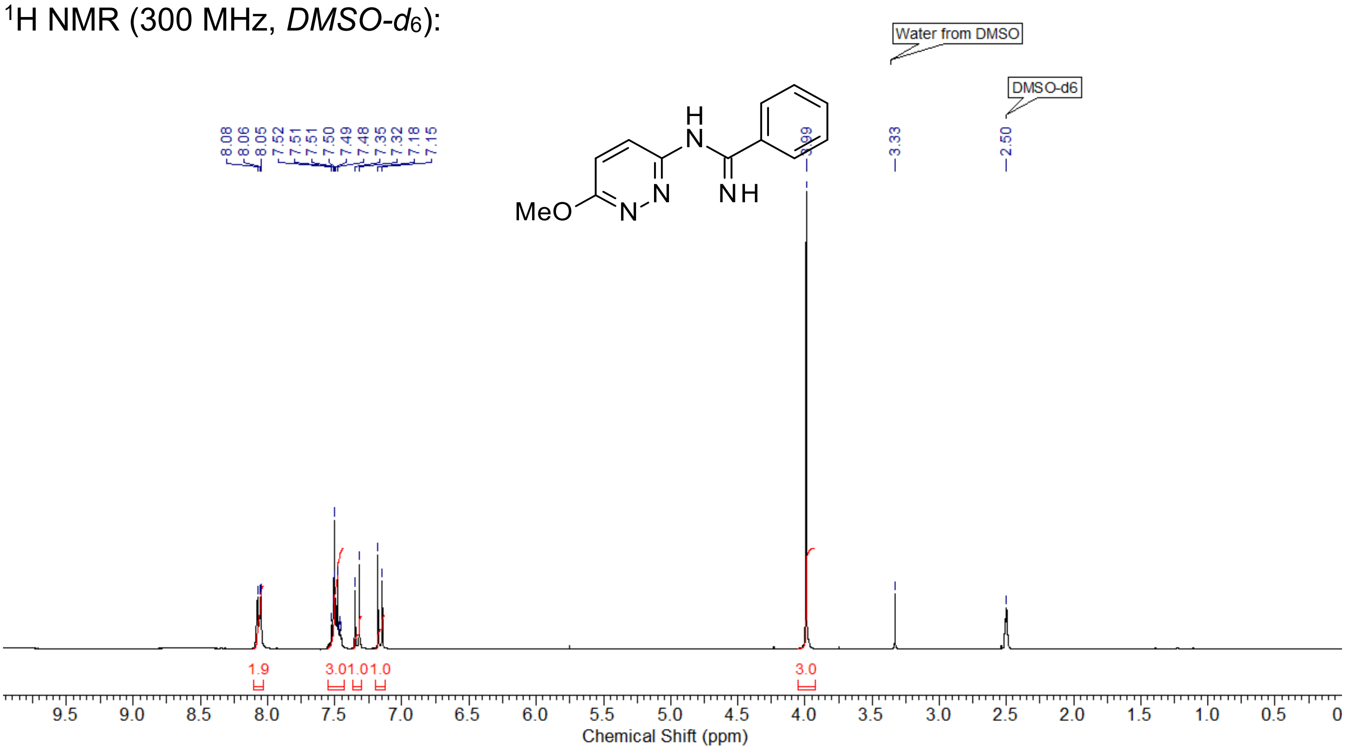

^{13}C NMR (75 MHz, DMSO-*d*$_6$):

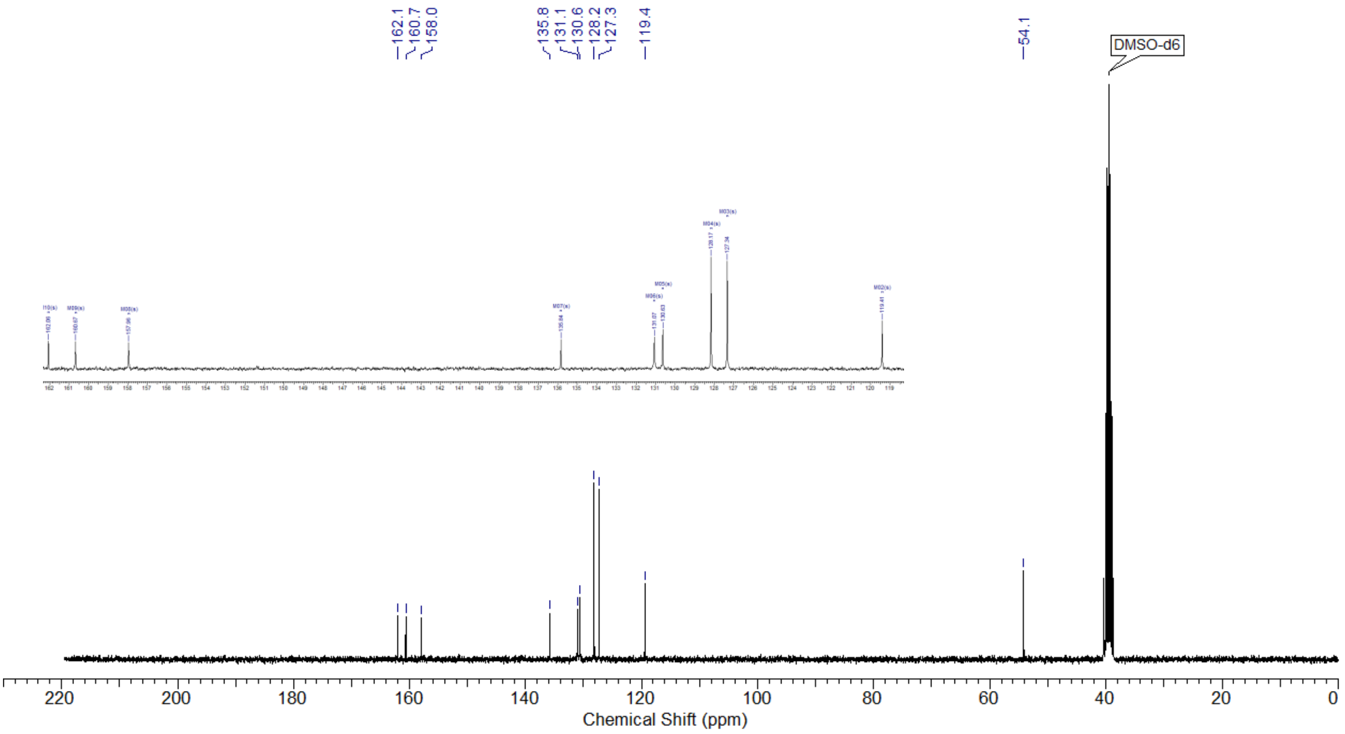

<u>*N*-(3-Pyridyl)benzamidine</u> (**4g**) [CAS 19673-11-1]

^{1}H NMR (300 MHz, *DMSO-d6*):

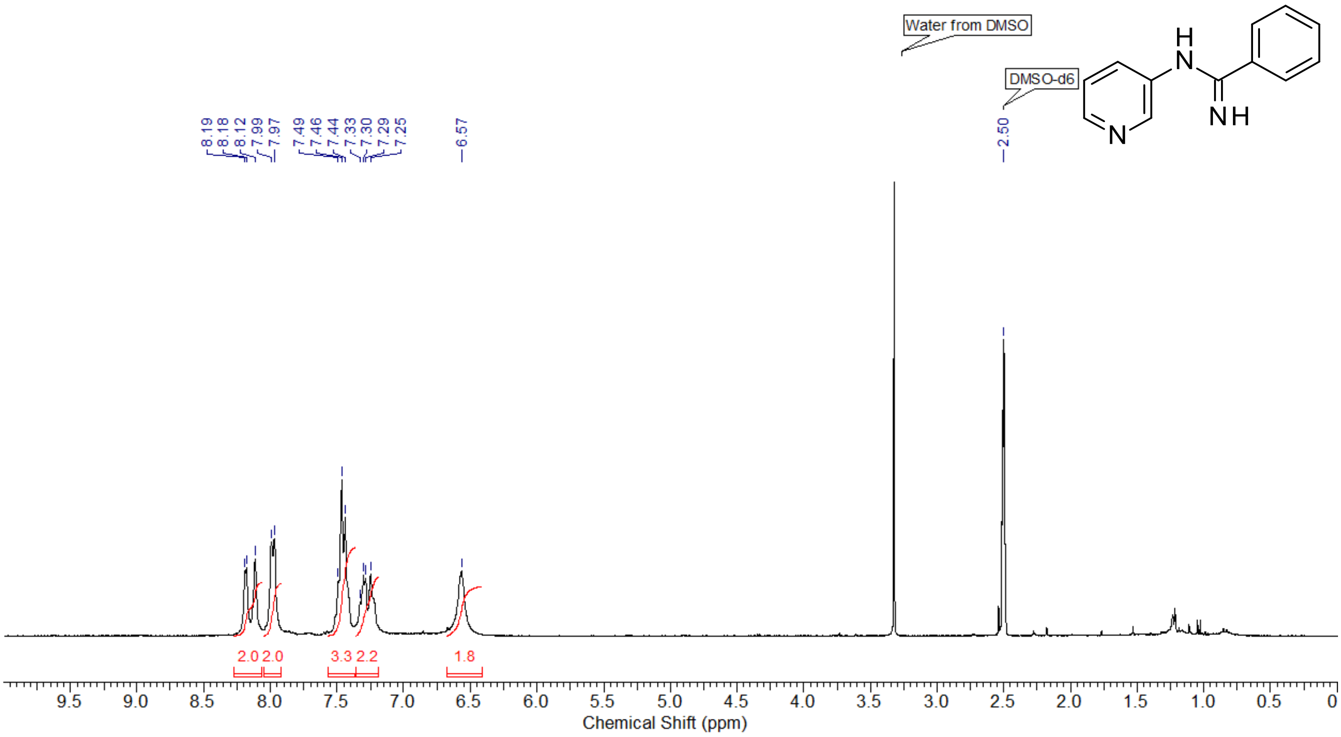

^{13}C NMR (75 MHz, DMSO-*d6*):

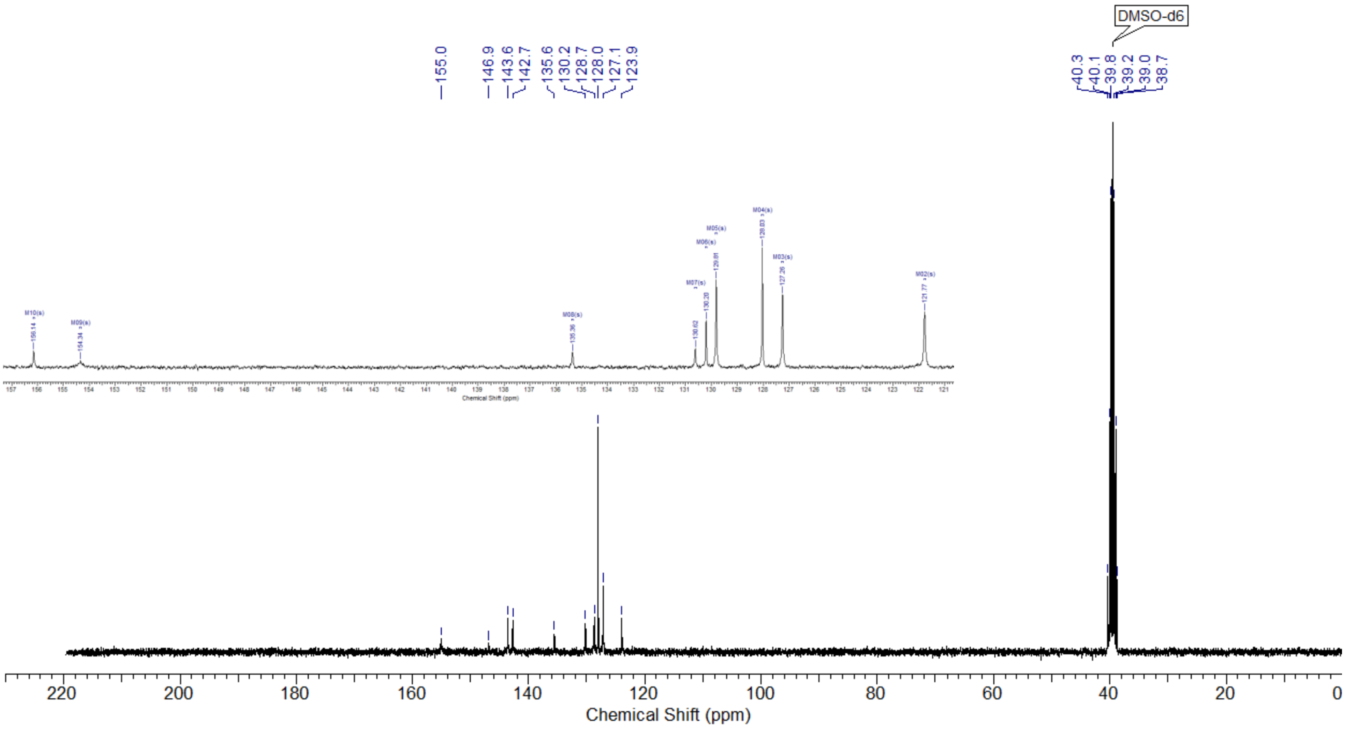

N-(2-Pyridyl)benzamidine (**4h**) [CAS 4931-08-2]

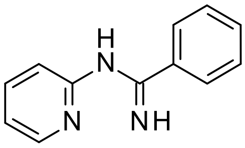

^{1}H NMR (300 MHz, *DMSO-d*$_6$):

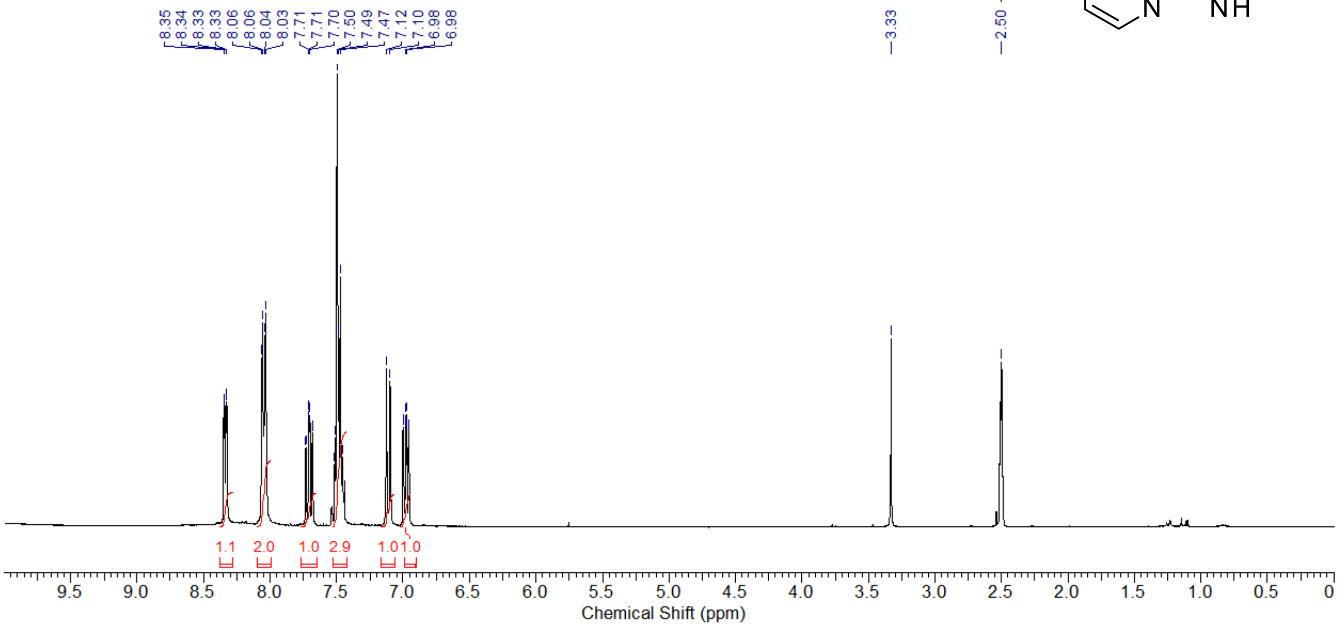

^{13}C NMR (75 MHz, DMSO-*d*$_6$):

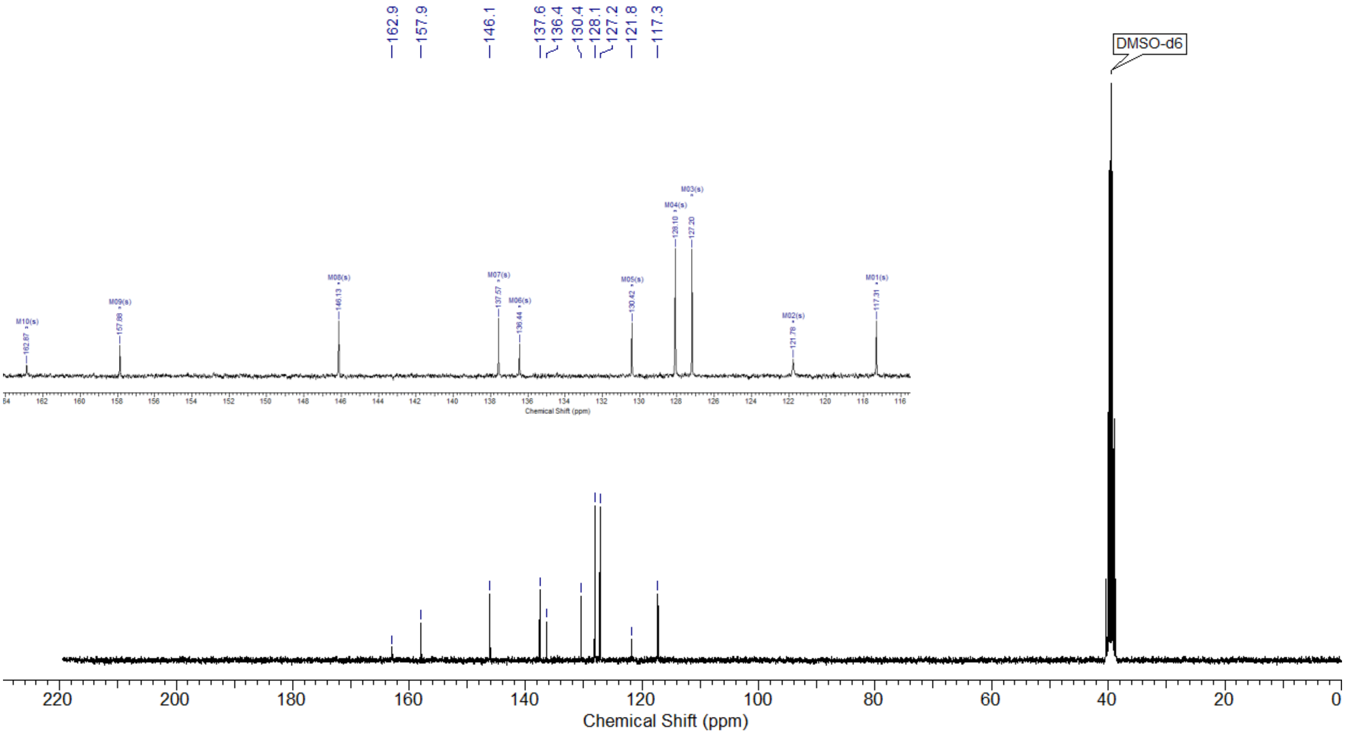

3-(4-Methoxyphenyl)-1-phenylurea (**5a**) [CAS 232597-42-1]

^{1}H NMR (300 MHz, DMSO-d_6):

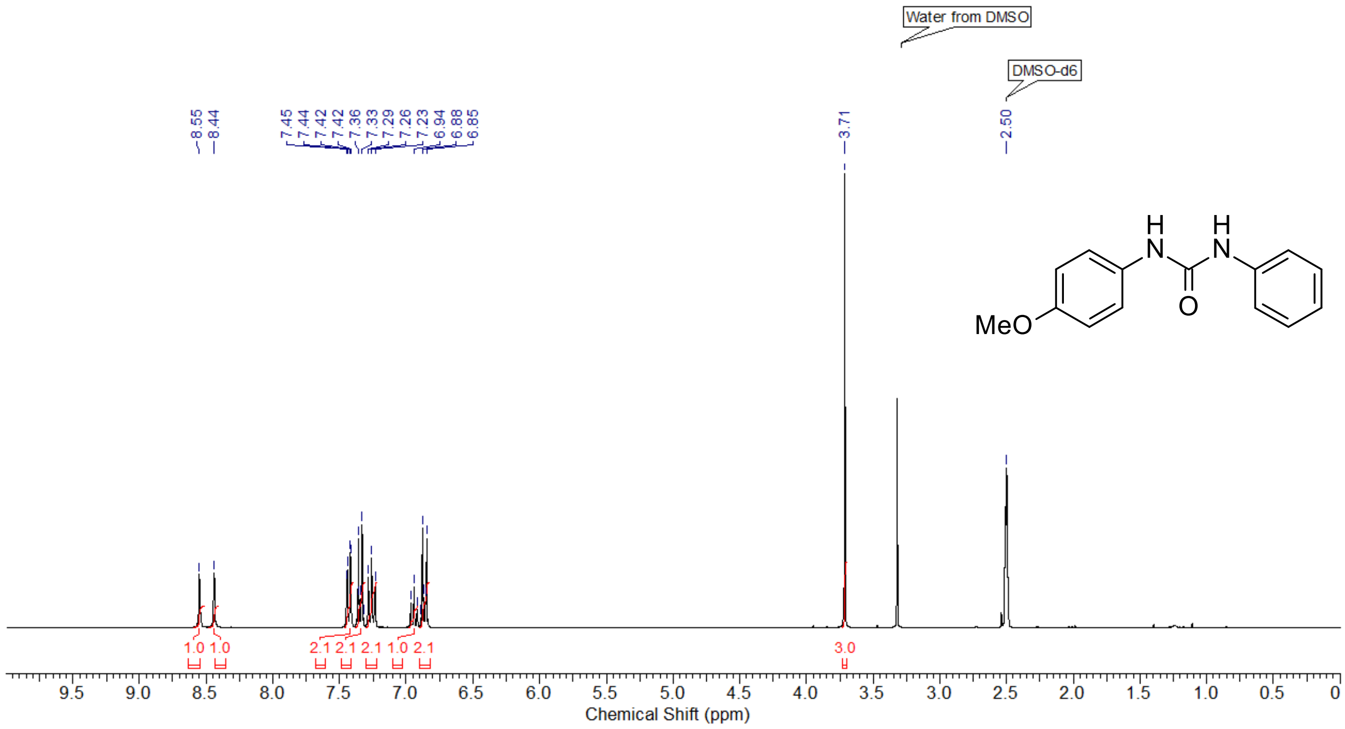

^{13}C NMR (75 MHz, DMSO-d_6):

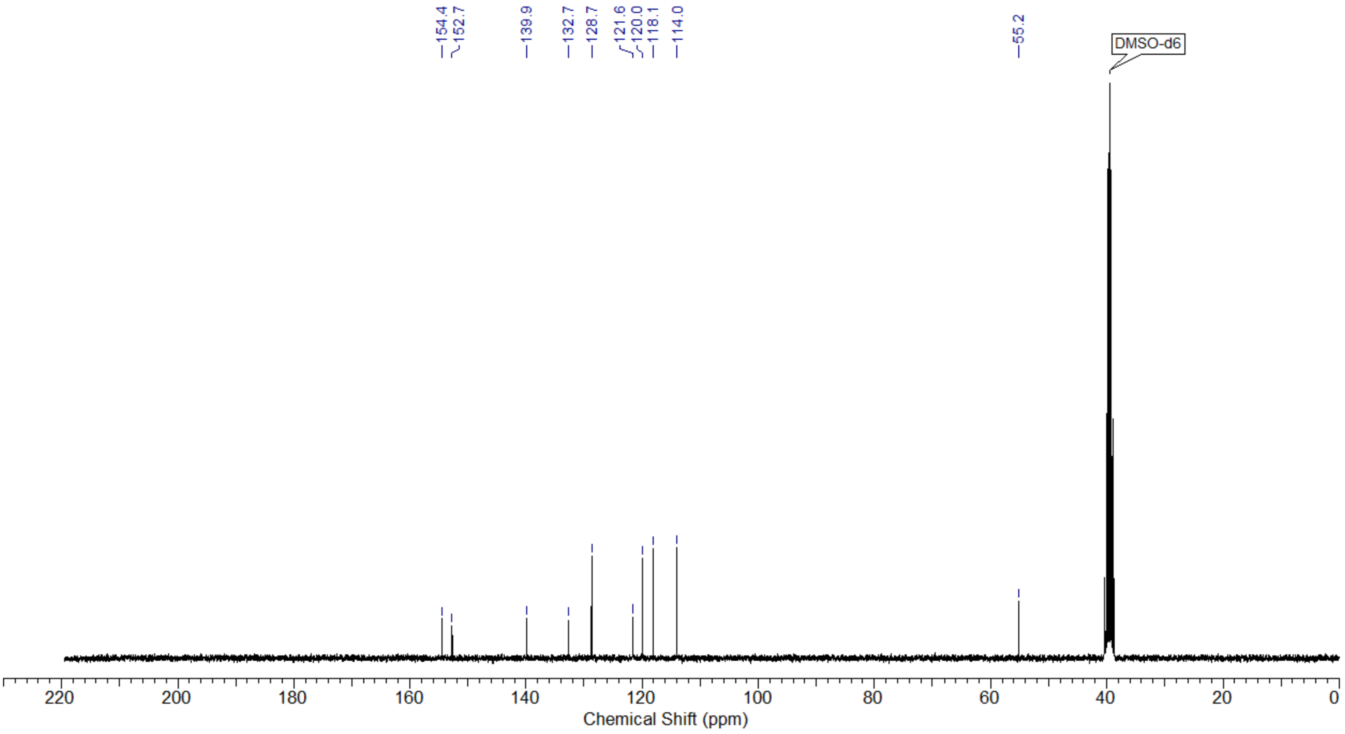

1-Phenyl-3-[4-(trifluoromethyl)phenyl]urea (**5b**) [CAS 23750-69-8]

^{1}H NMR (300 MHz, DMSO-d_6):

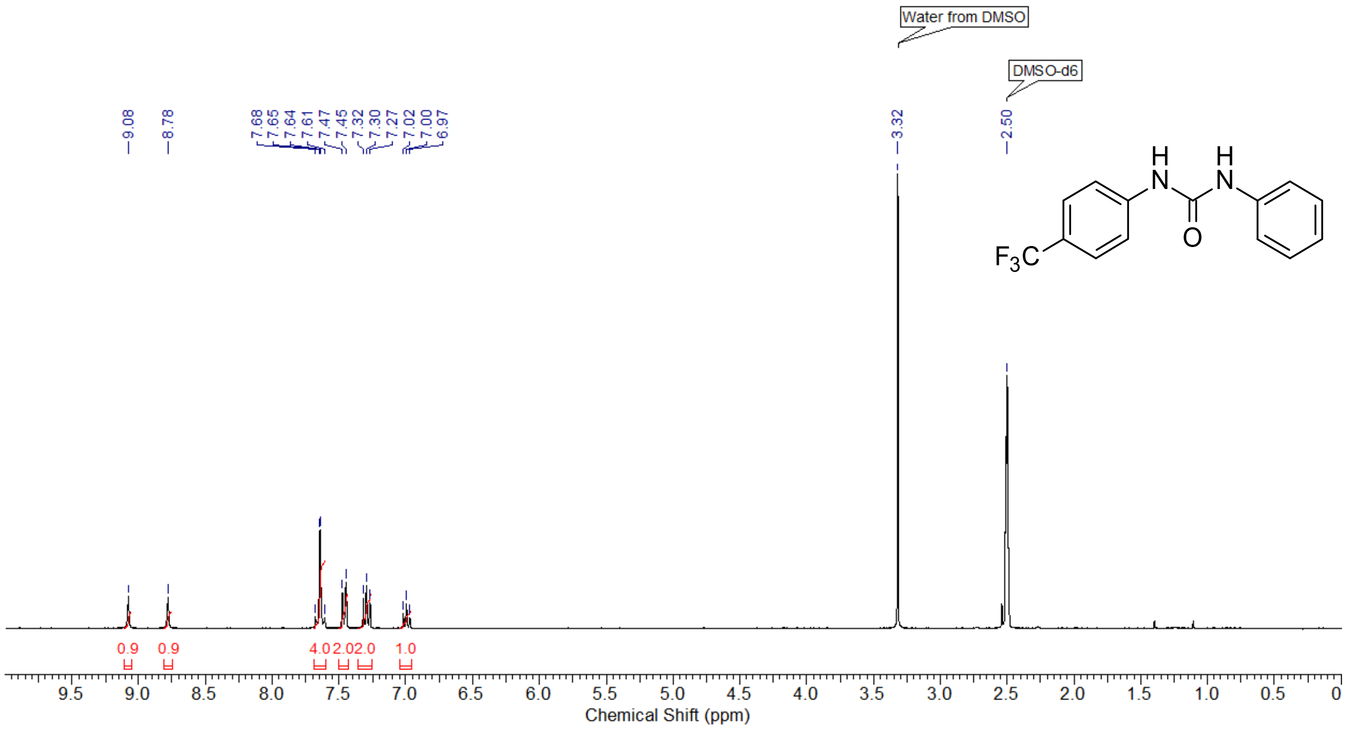

^{13}C NMR (75 MHz, DMSO-d_6):

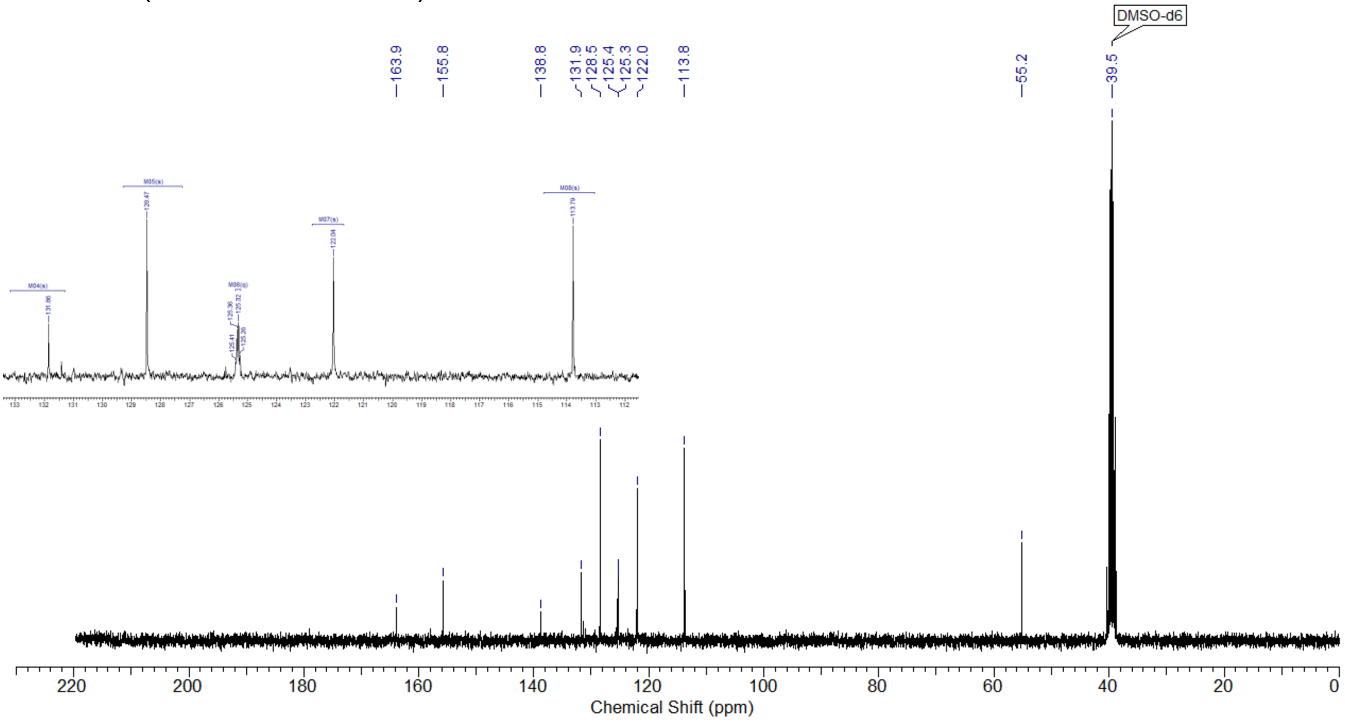

^{19}F NMR (76 MHz, DMSO-d_6, $C_6H_4F_2$):

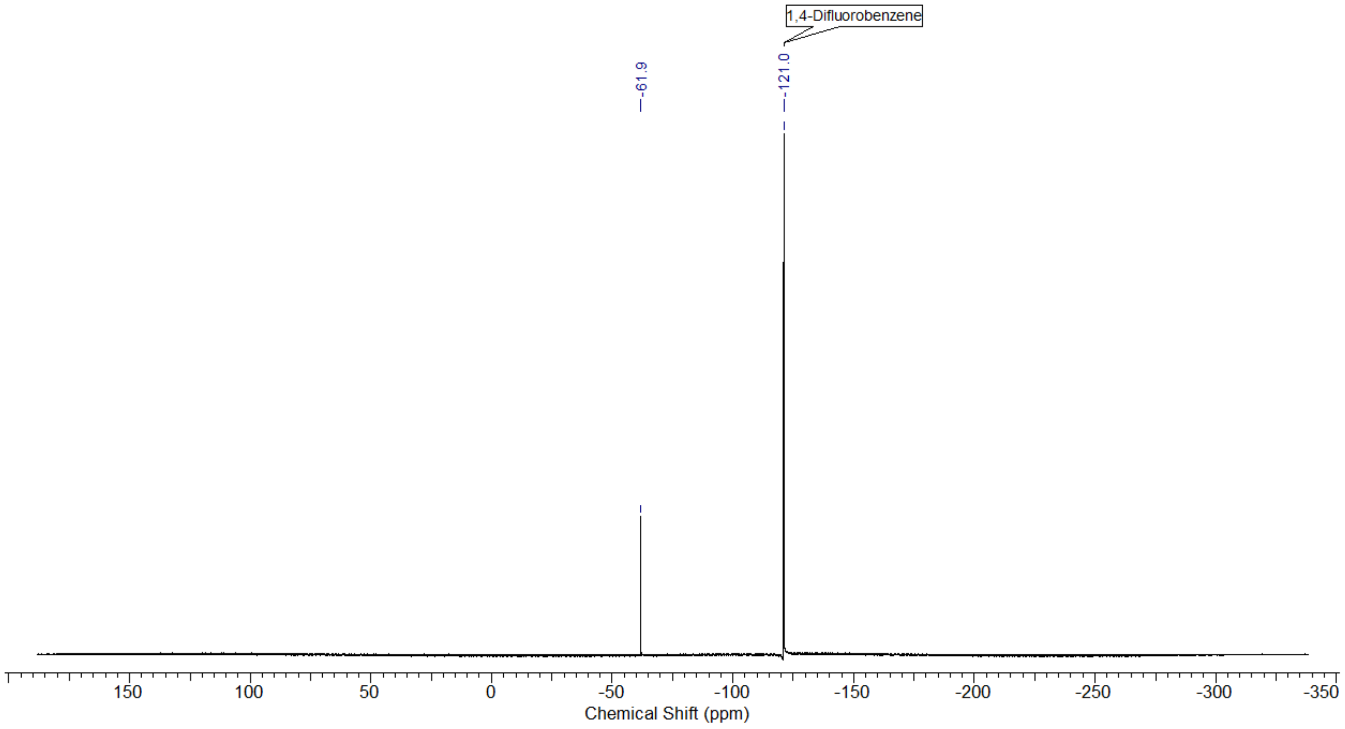

1-(4-Methylphenyl)-3-phenylurea (**5c**) [CAS 4300-33-8]

^{1}H NMR (300 MHz, DMSO-d_6):

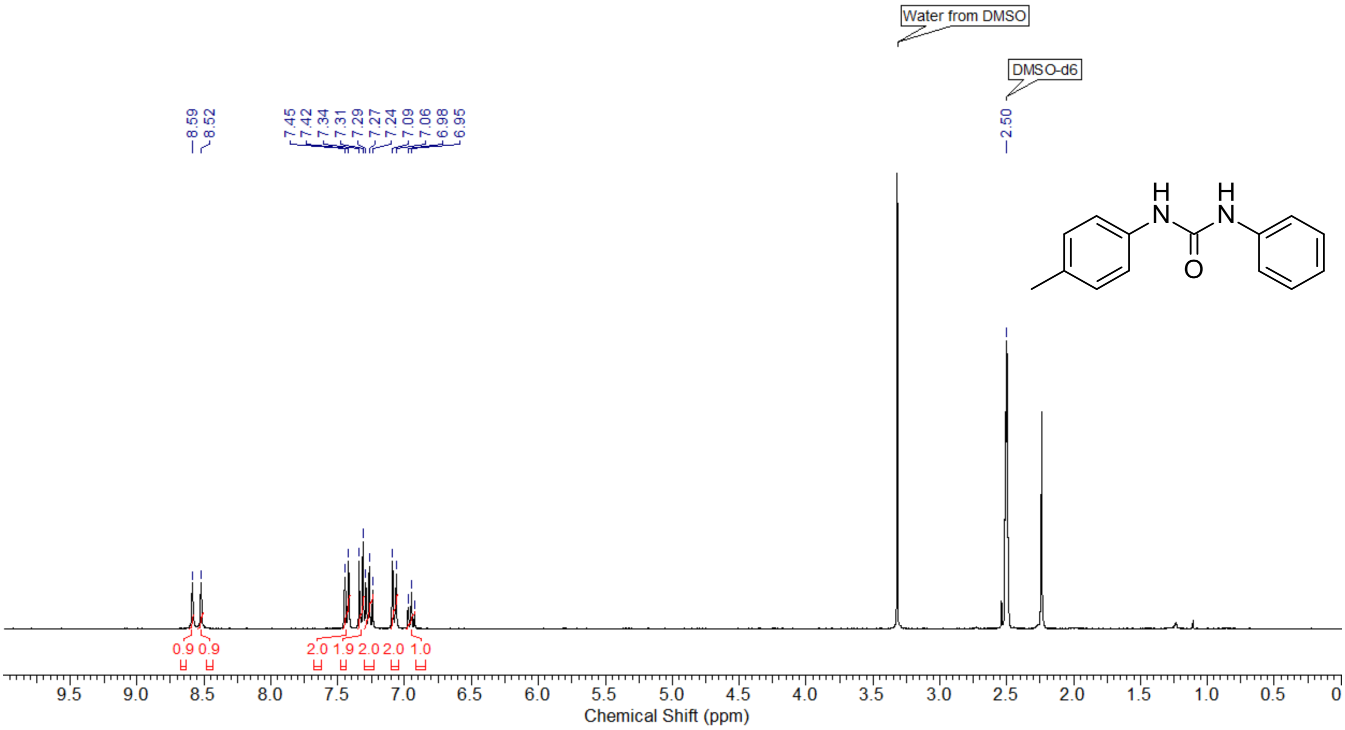

^{13}C NMR (75 MHz, DMSO-d_6):

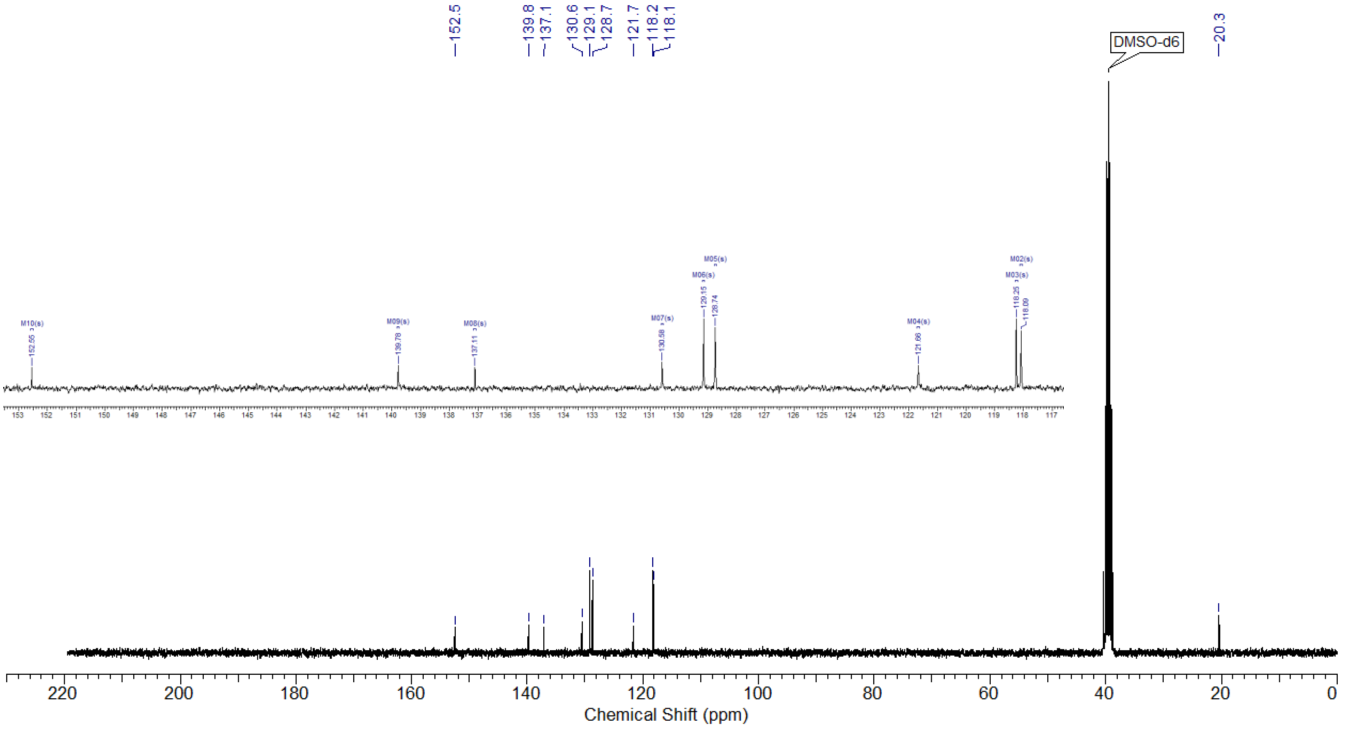

1-(2-Methylphenyl)-3-phenylurea (**5d**) [CAS 23750-69-8]

^{1}H NMR (300 MHz, DMSO-d_6):

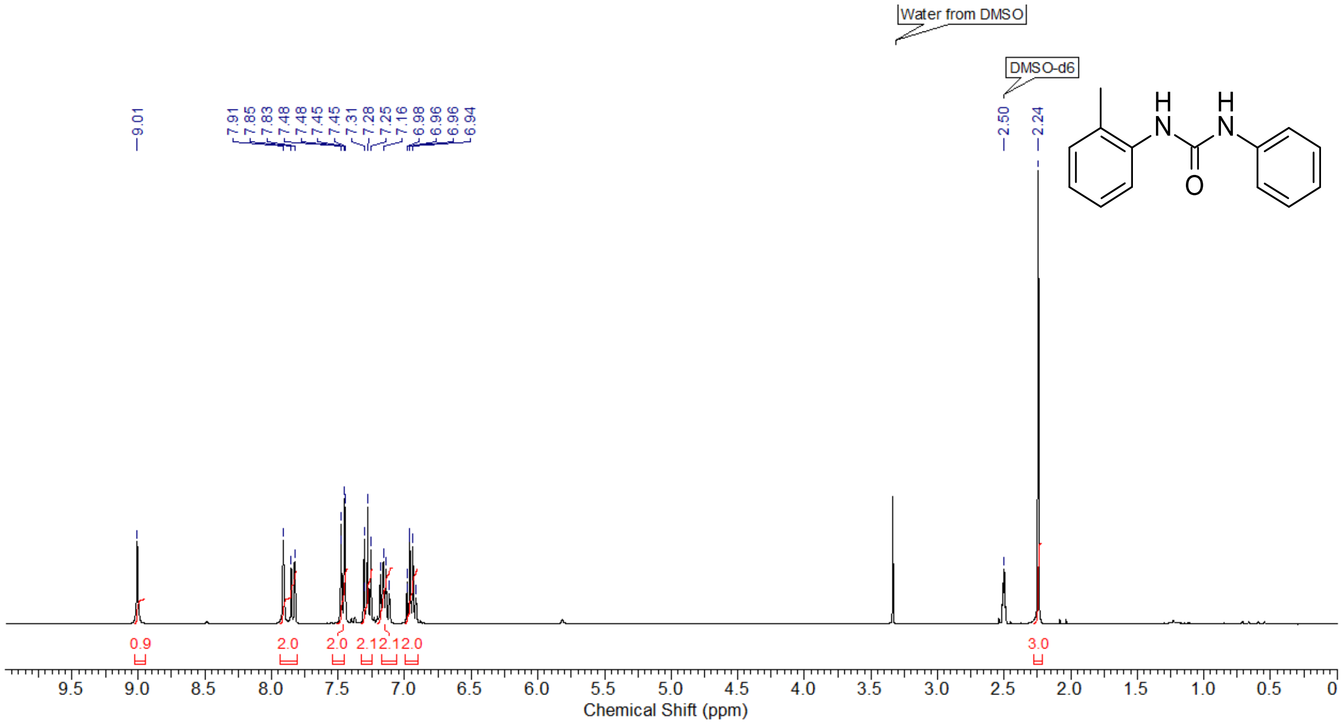

^{13}C NMR (75 MHz, DMSO-d_6):

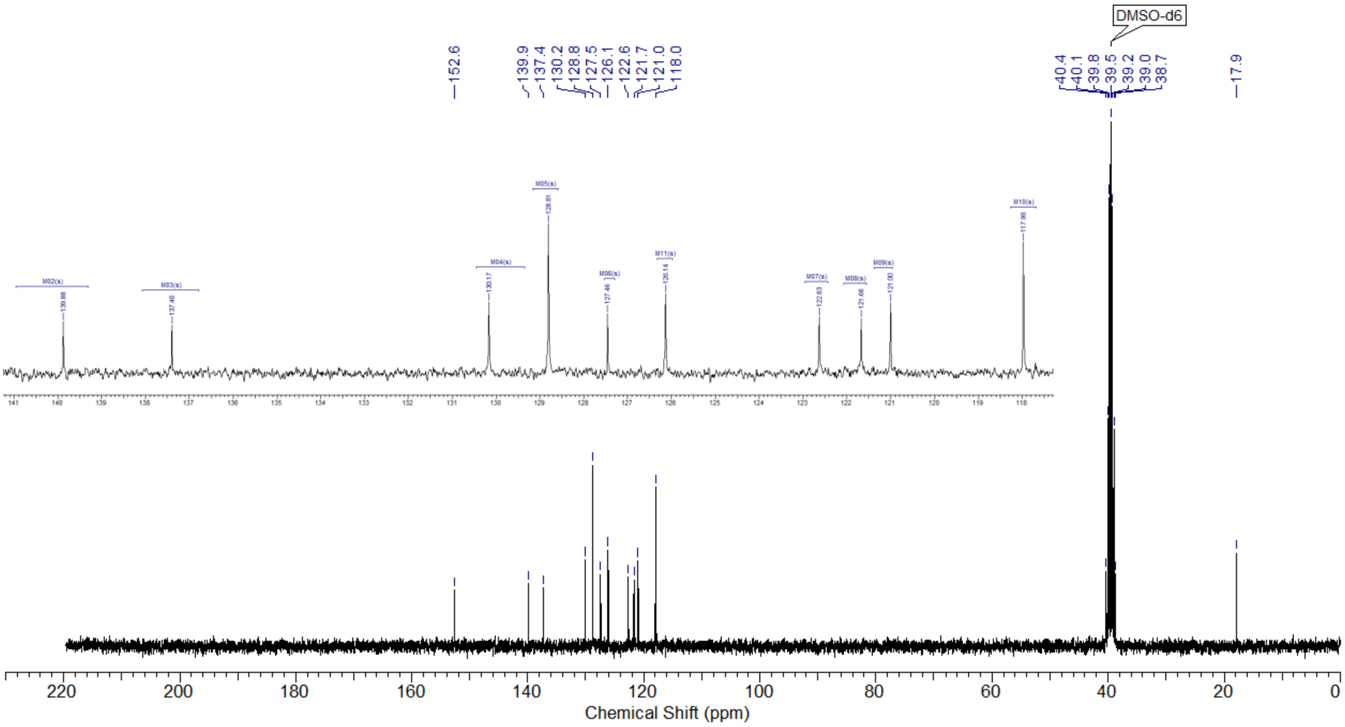

1-(4-isopropenylphenyl)-3-phenyl-urea (**5e**)

^{1}H NMR (300 MHz, *DMSO-d*$_6$):

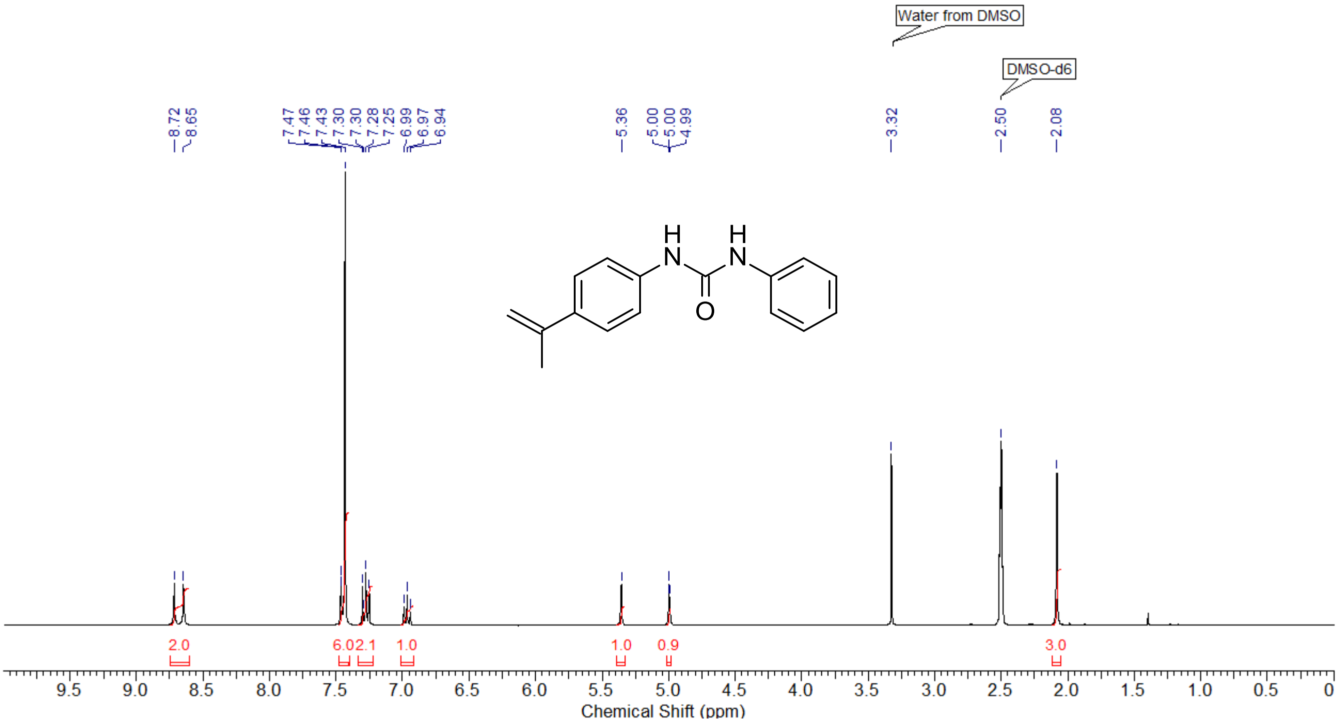

^{13}C NMR (75 MHz, *DMSO-d*$_6$):

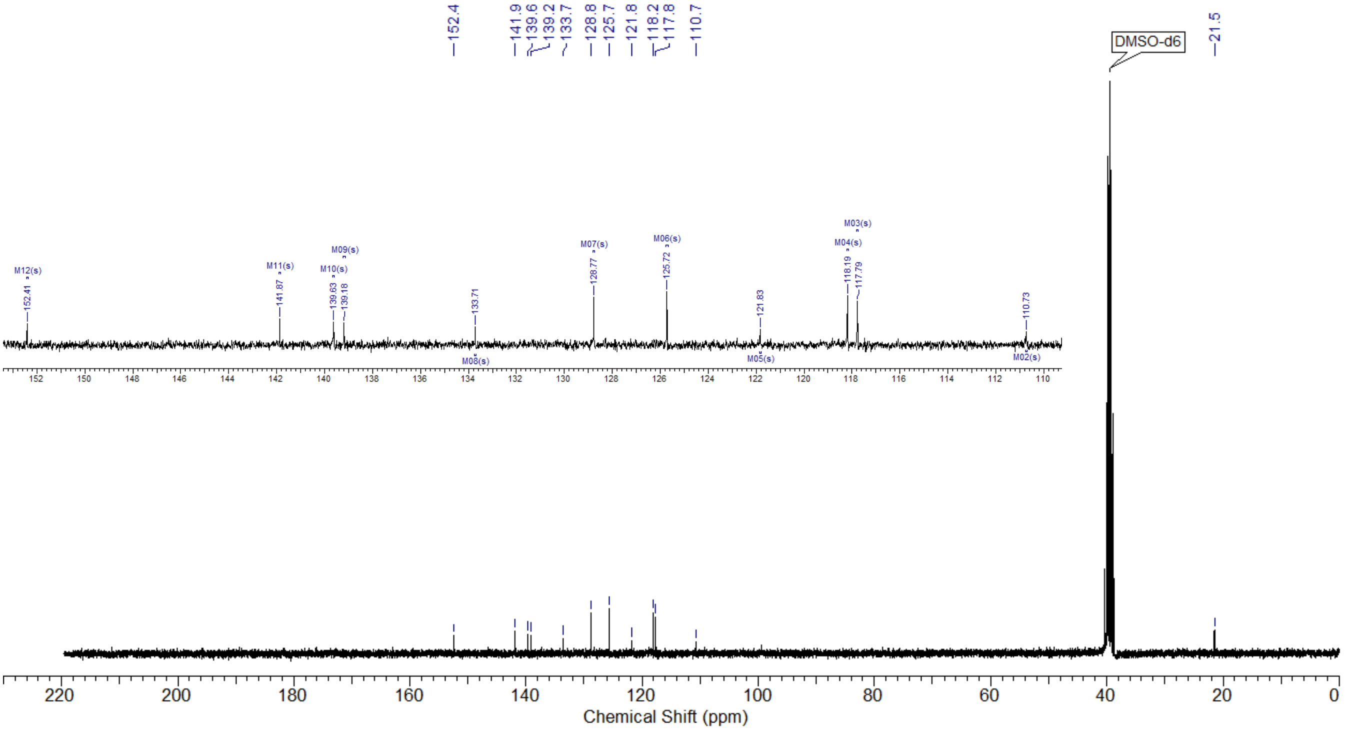

N-(2-Methyl-5-benzoxazolyl)-N-phenylurea (**5f**) [CAS : 1787872-65-4]

^{1}H NMR (300 MHz, DMSO-d_6):

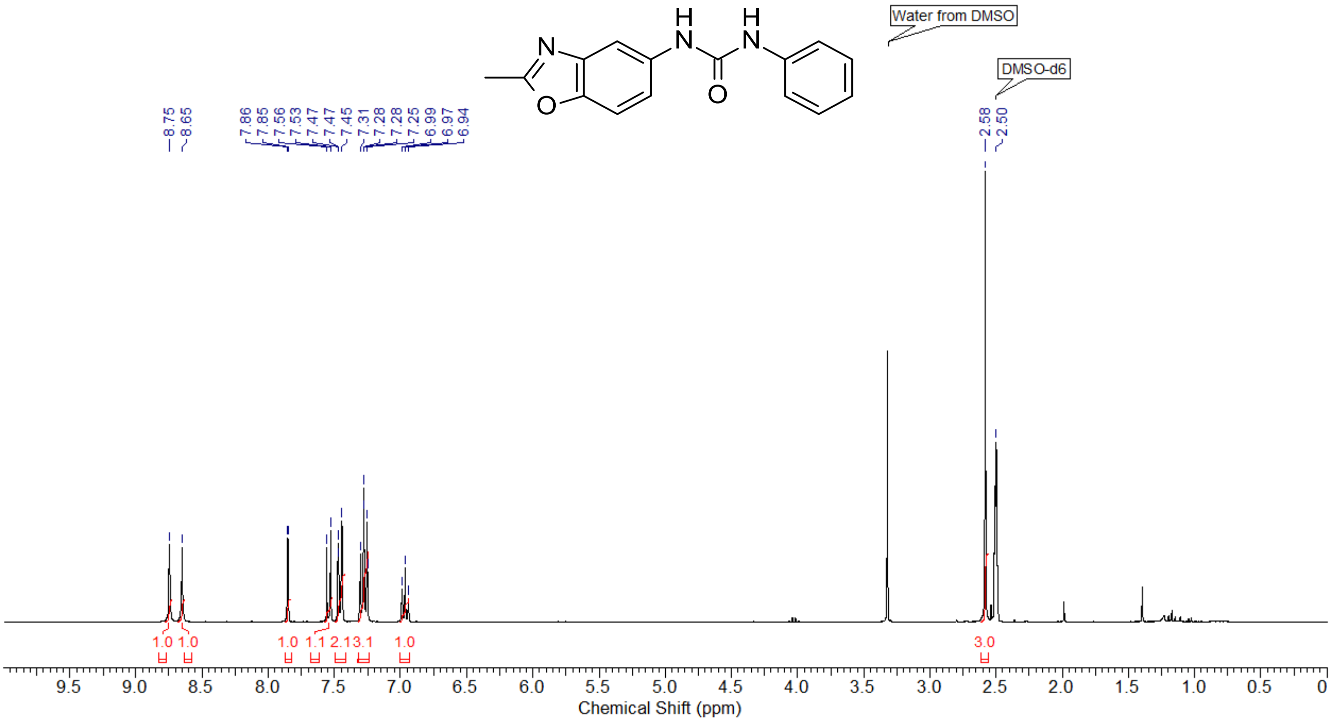

^{13}C NMR (75 MHz, DMSO-d_6):

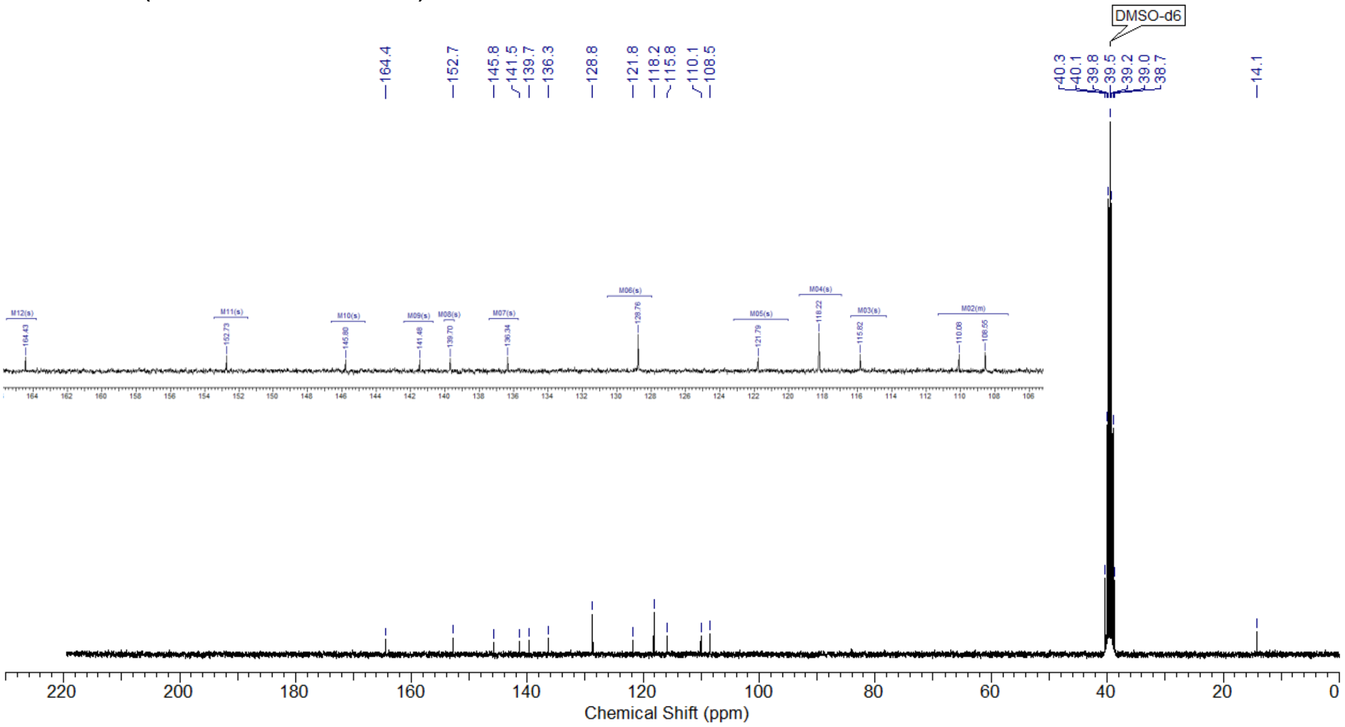

N-Phenyl-N′-3-thienylurea (**5g**) [CAS :2361877-12-3]

^{1}H NMR (300 MHz, *DMSO-d*6):

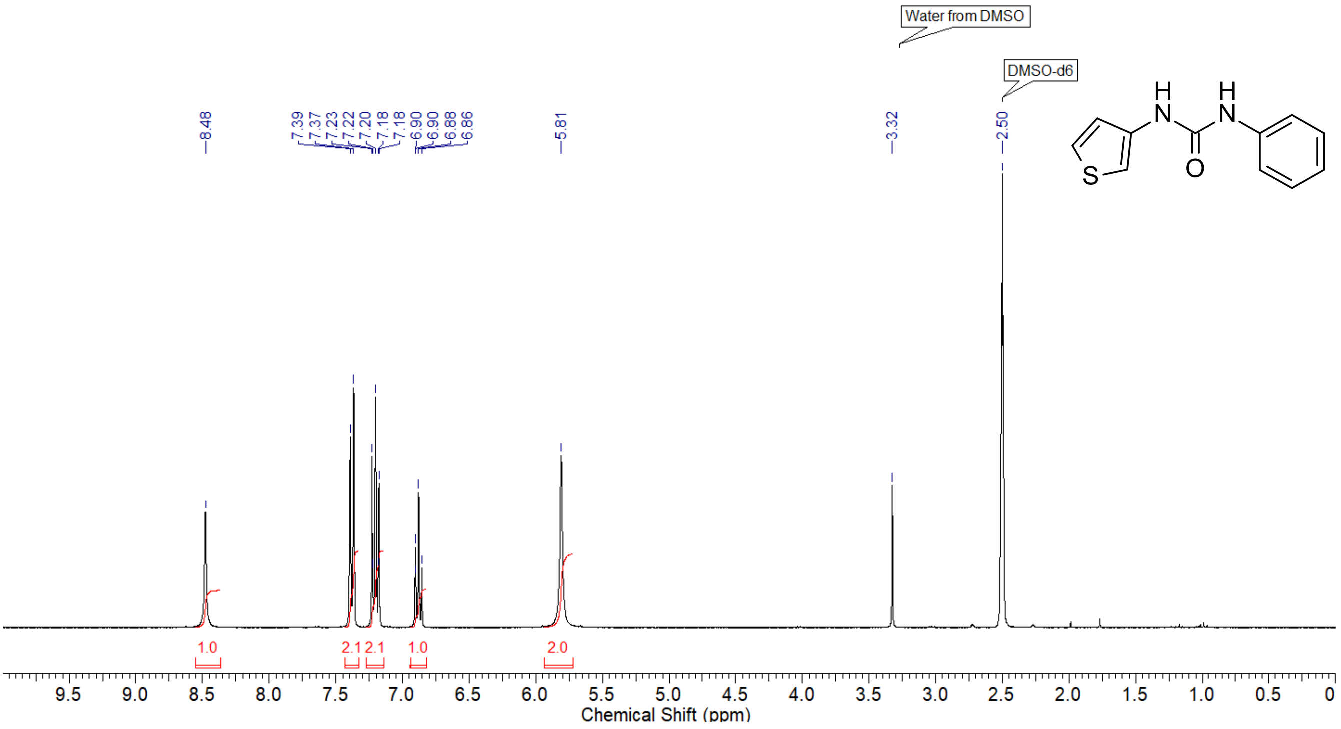

^{13}C NMR (75 MHz, *DMSO-d*6):

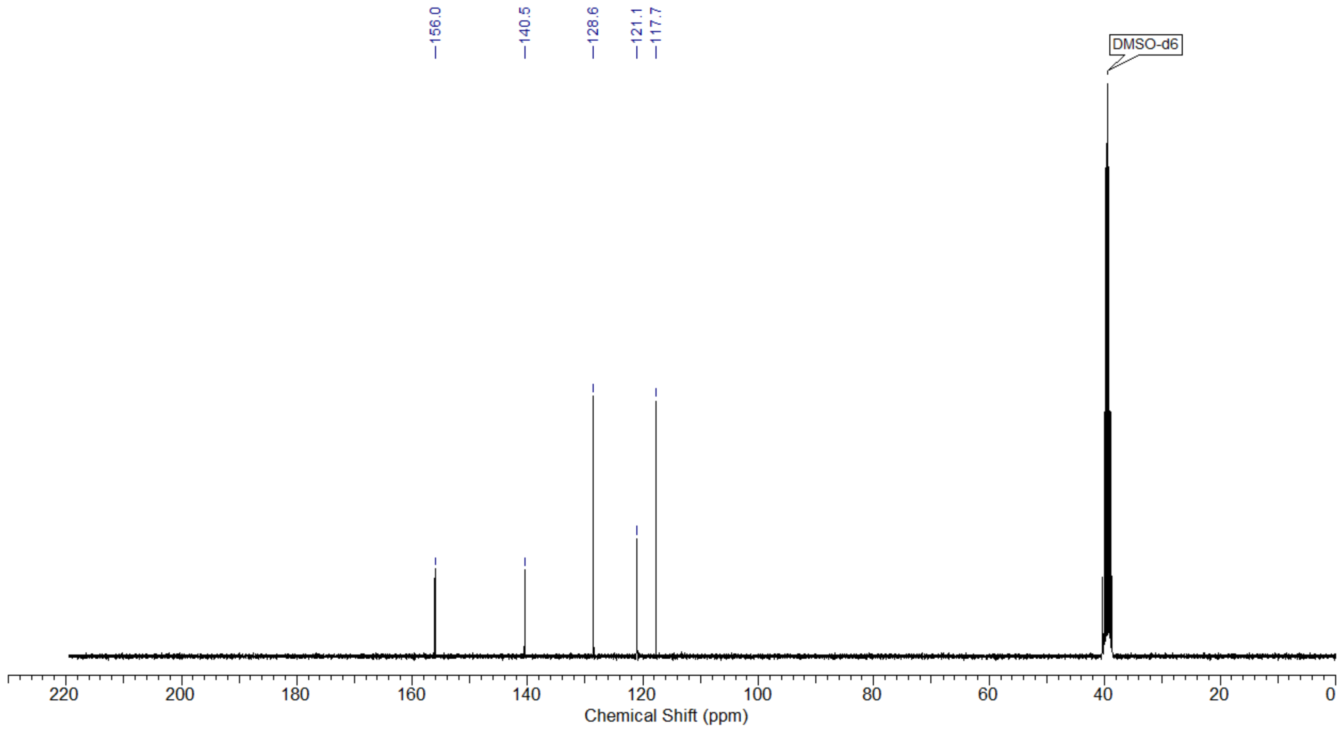

tert-Butyl N-(4-methoxyphenyl)carbamate (**6a**) [CAS 18437-68-8]

^{1}H NMR (300 MHz, $CDCl_3$):

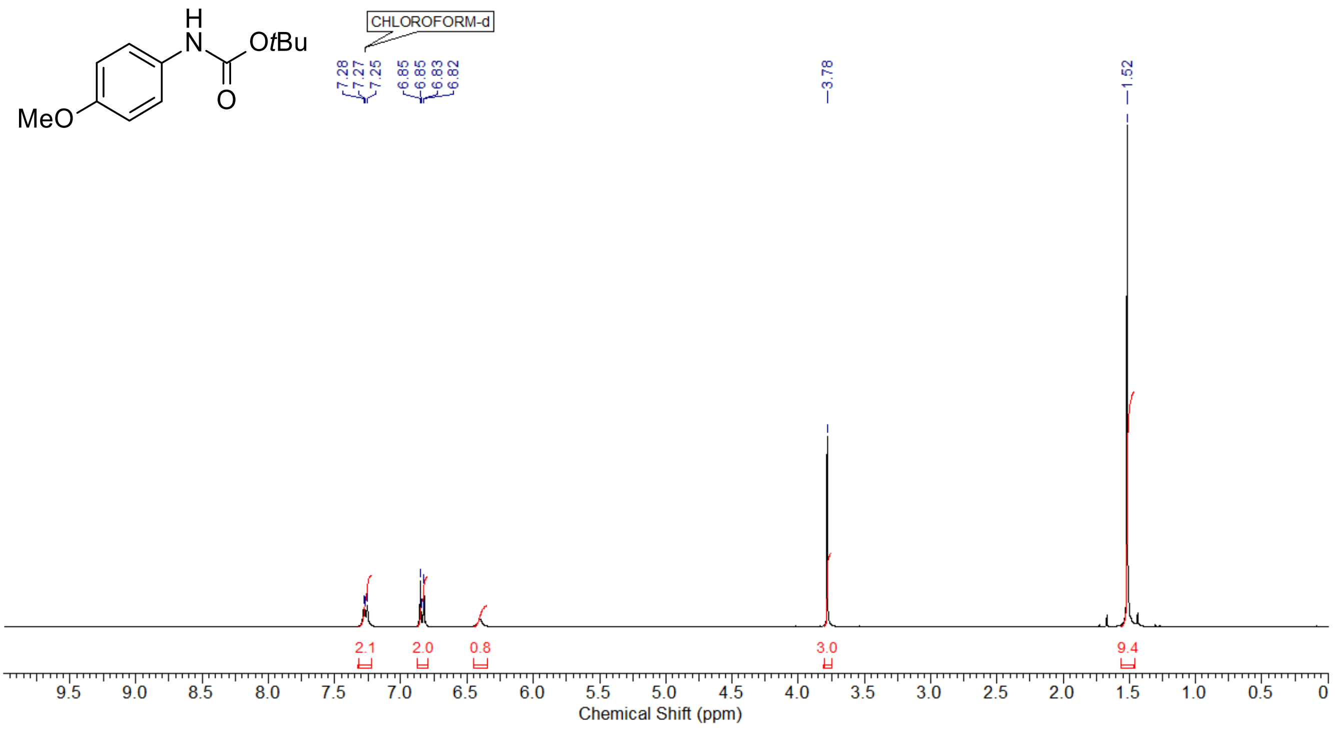

^{13}C NMR (75 MHz, $CDCl_3$):

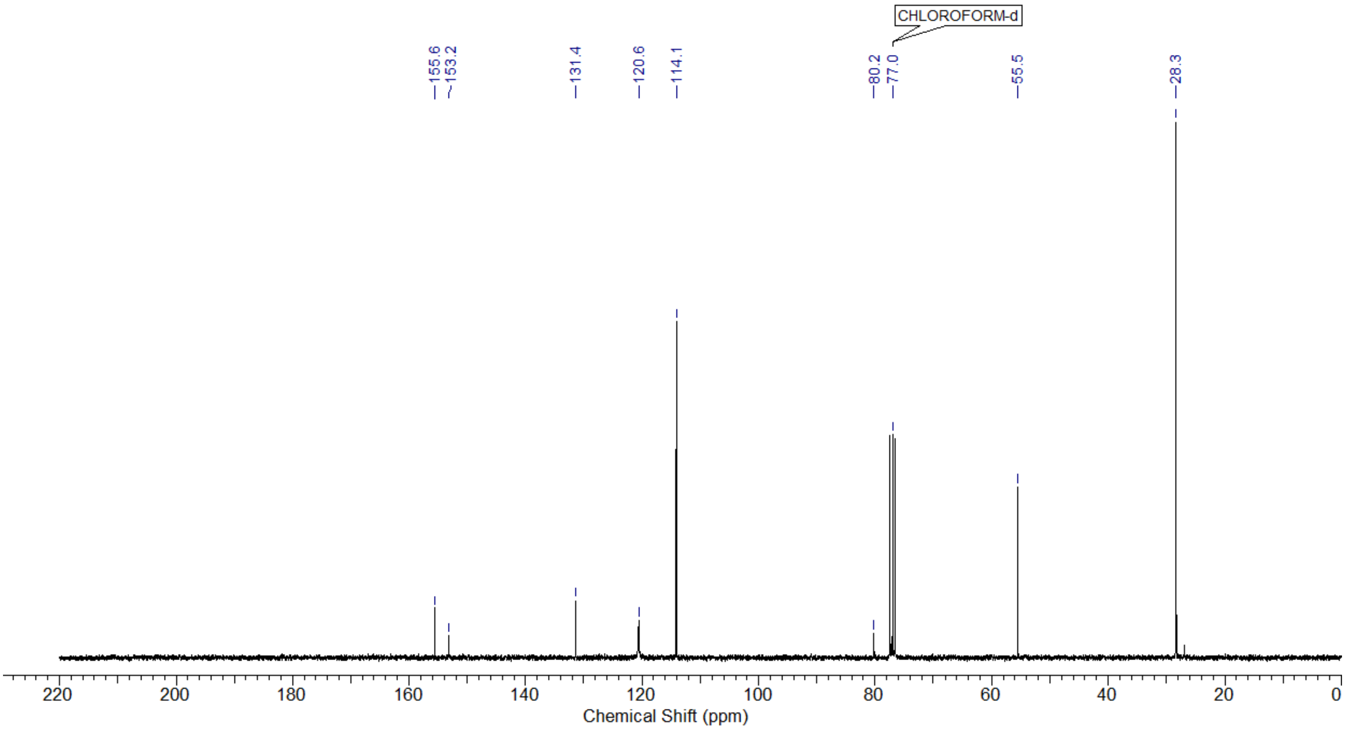

<u>*tert*-Butyl 4-trifluoromethylphenylcarbamate</u> (**6b**) [CAS 141940-37-6]

^1H NMR (300 MHz, $CDCl_3$):

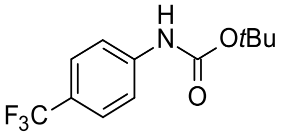

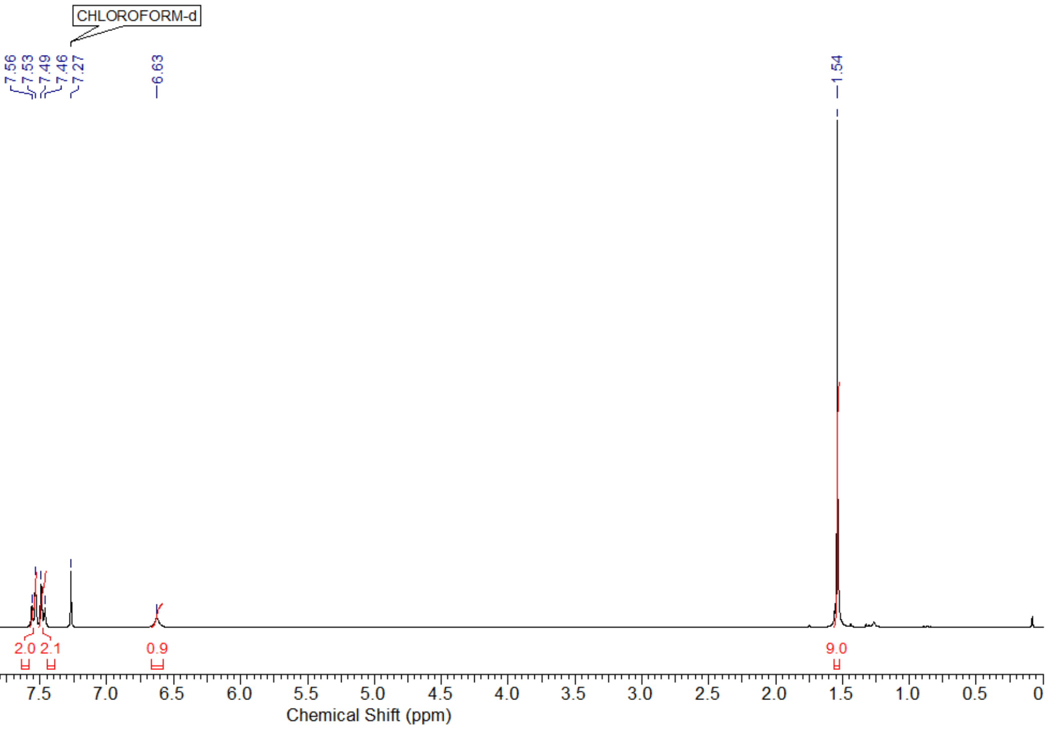

^{13}C NMR (75 MHz, $CDCl_3$):

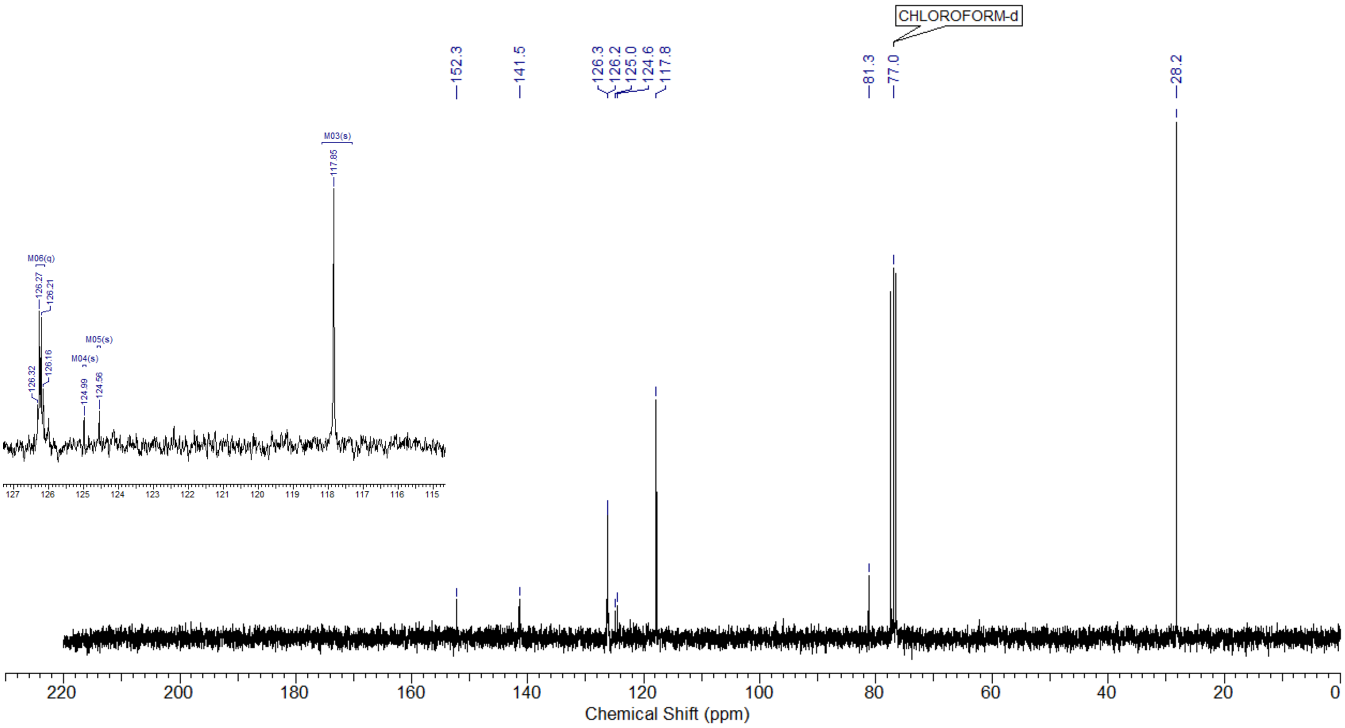

^{19}F NMR (76 MHz, $CDCl_3$, $C_6H_4F_2$):

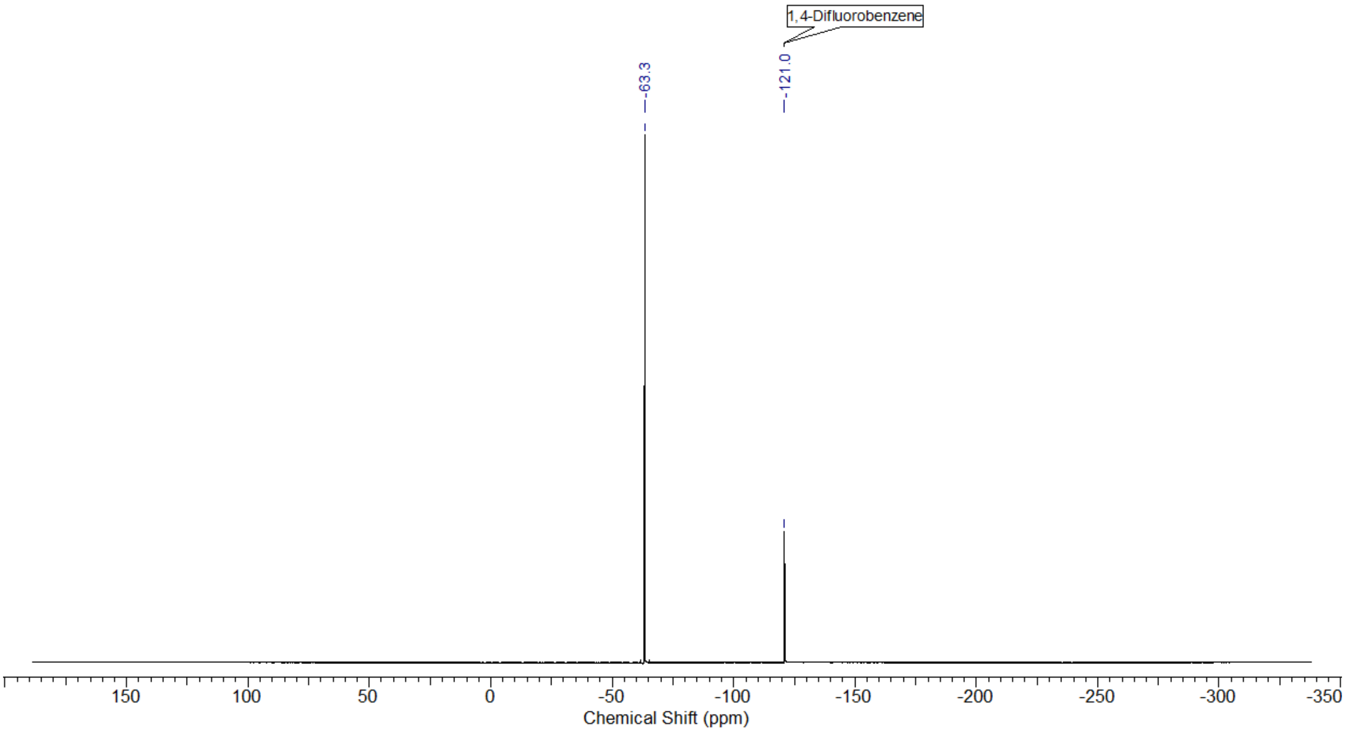

Ethyl 4-((tert-butoxycarbonyl)amino)benzoate (**6c**) [CAS 110969-44-3]

^{1}H NMR (300 MHz, $CDCl_3$):

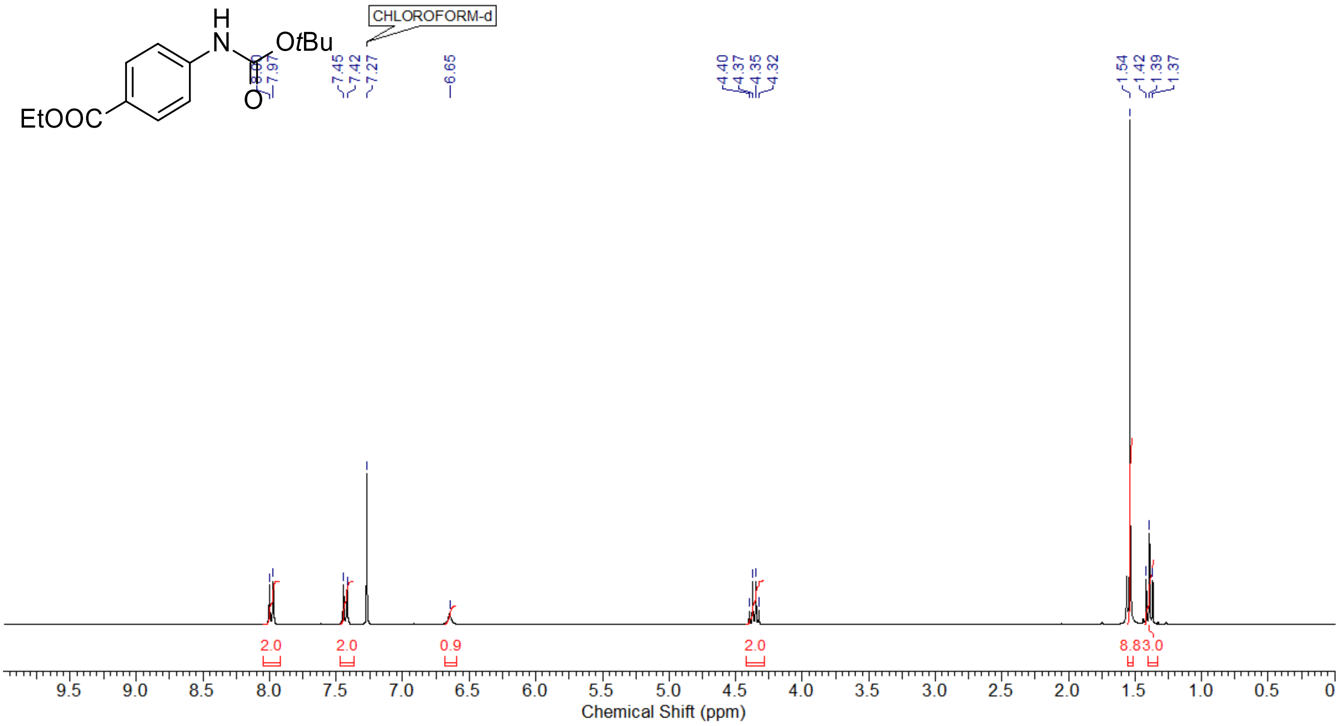

^{13}C NMR (75 MHz, $CDCl_3$):

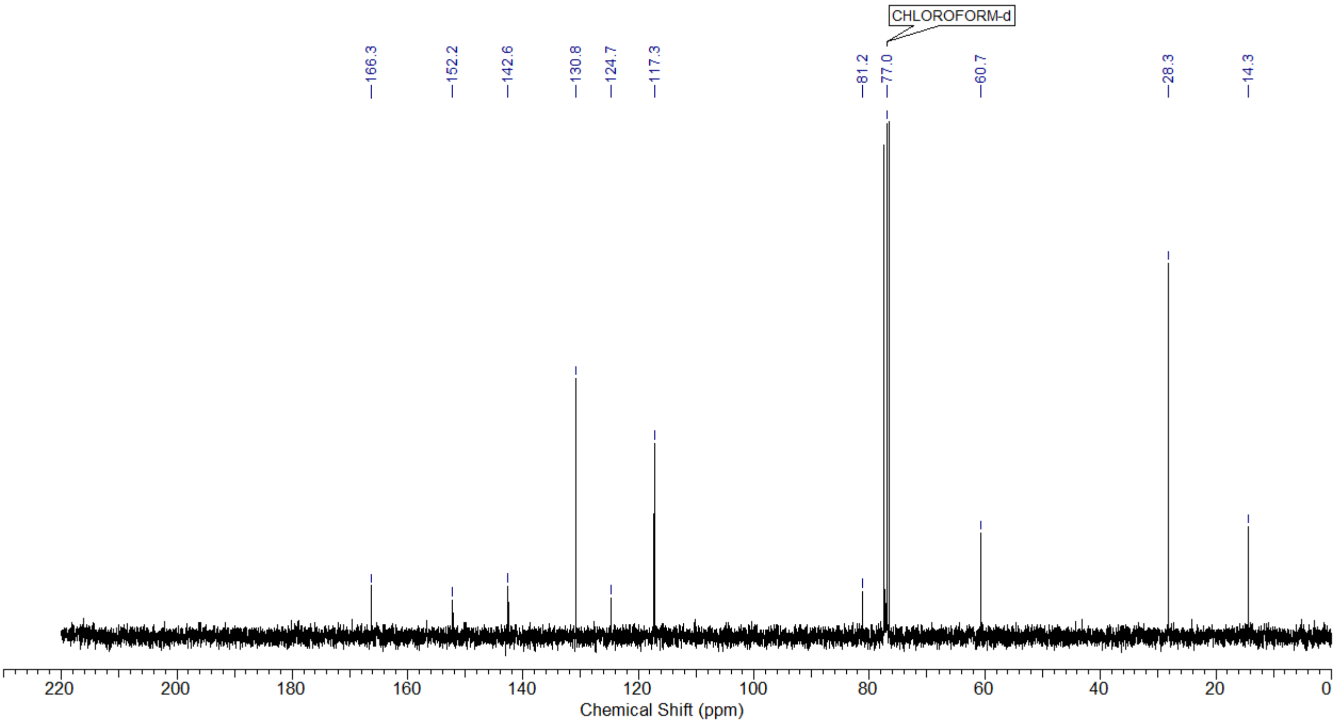

tert-Butyl (4-acetylphenyl)carbamate (**6d**) [CAS 232597-42-1]

^{1}H NMR (300 MHz, $CDCl_3$):

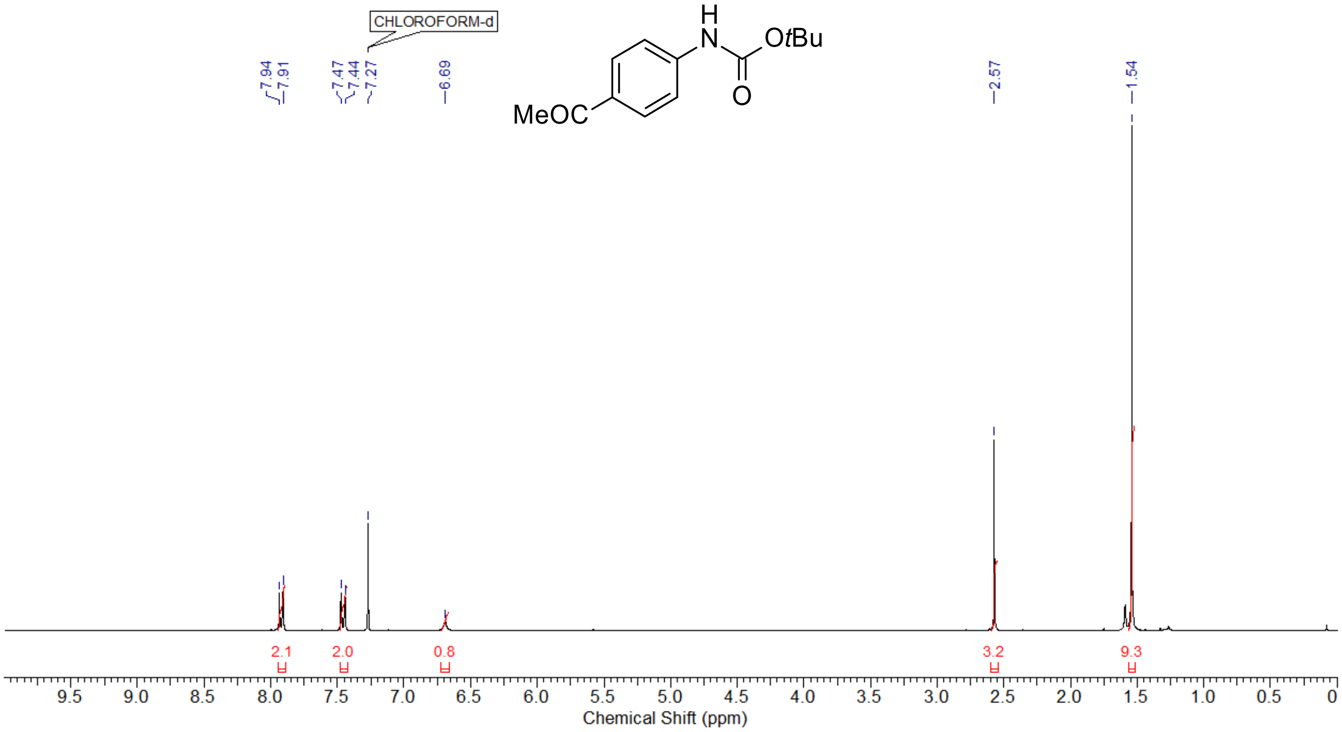

^{13}C NMR (75 MHz, $CDCl_3$):

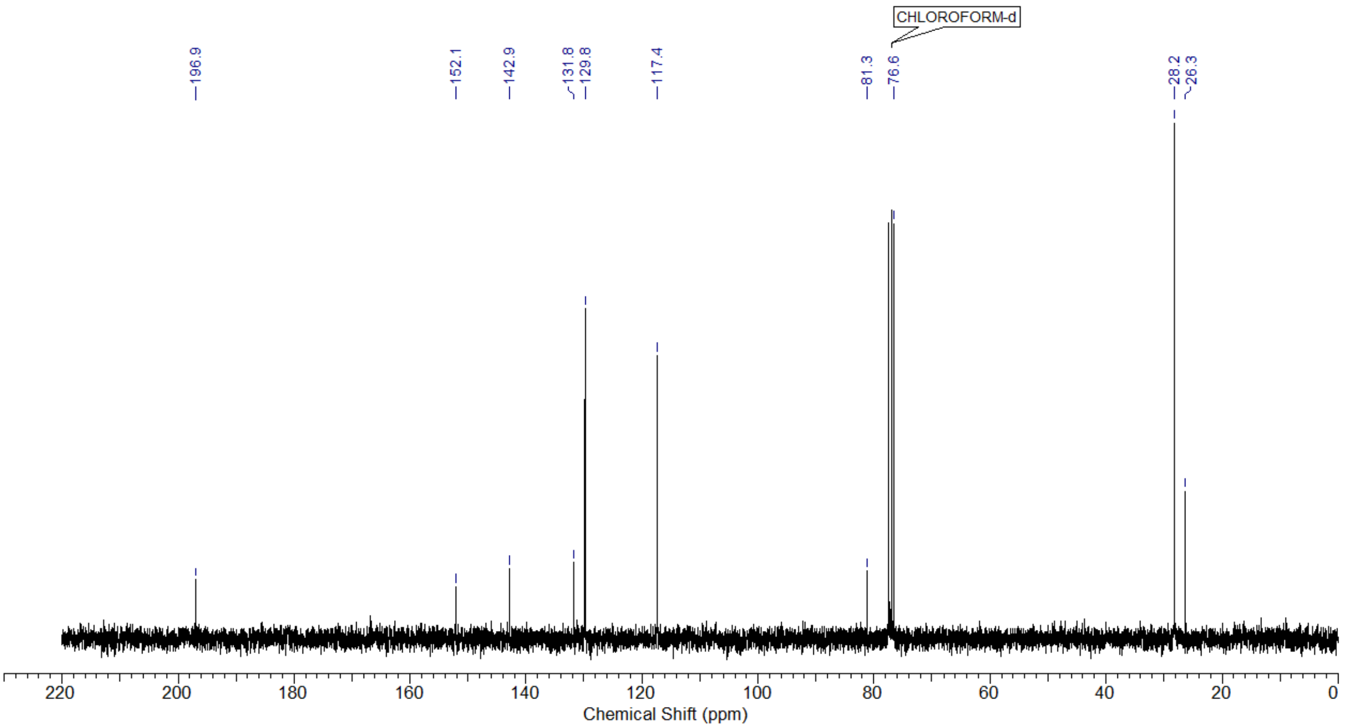

tert-butyl N-(4-trimethylsilylphenyl)carbamate (**6e**) [CAS 2231316-68-8]

^{1}H NMR (300 MHz, *DMSO-d*$_6$):

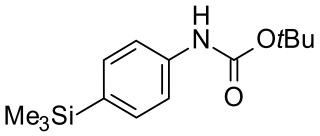

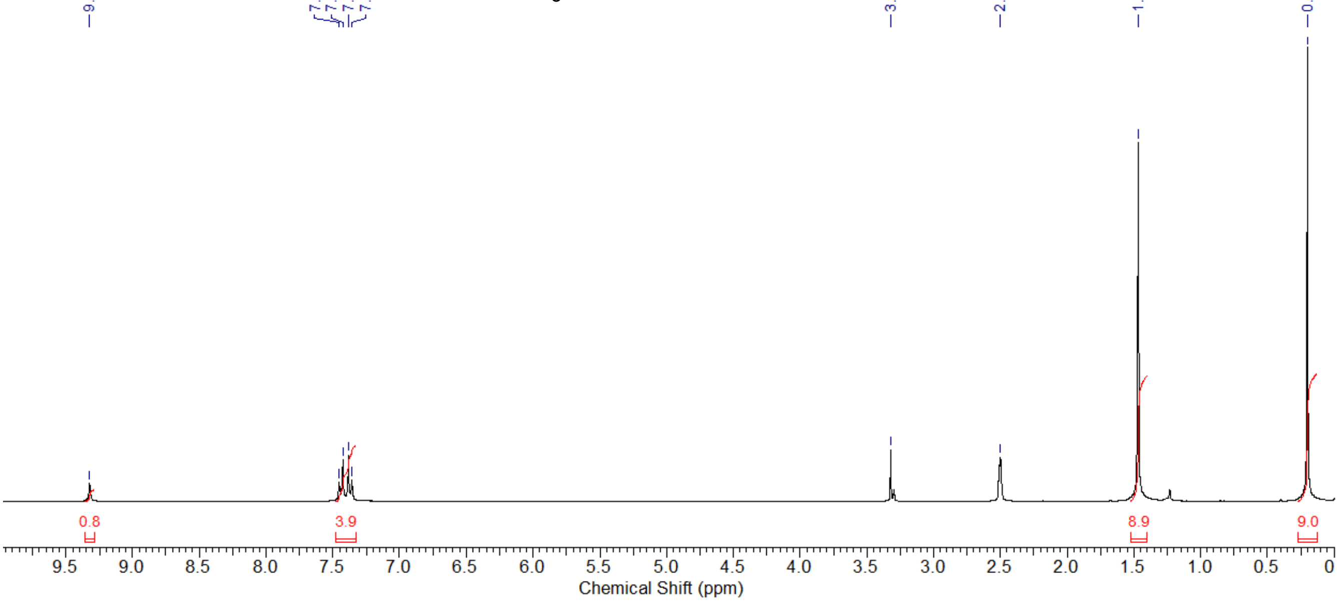

^{13}C NMR (75 MHz, *DMSO-d*$_6$):

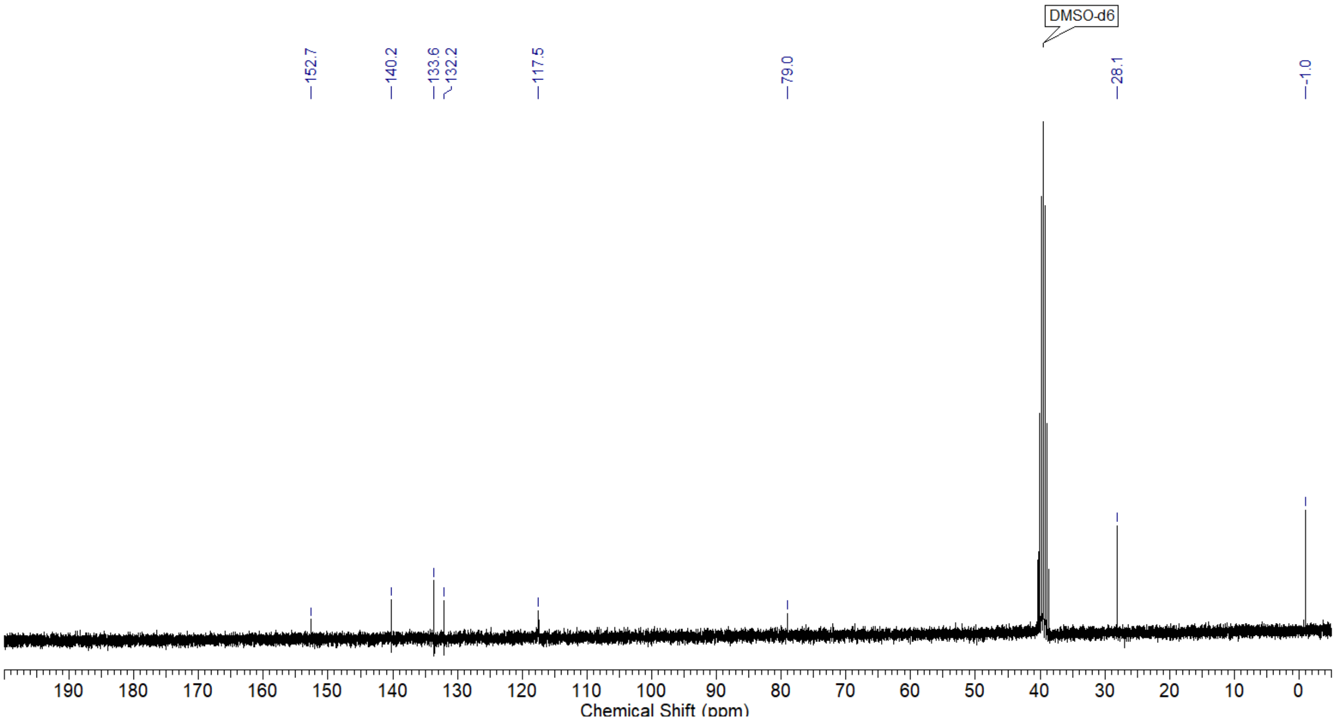

1,1-Dimethylethyl N-3-thienylcarbamate (**6f**) [CAS 19228-91-2]

^{1}H NMR (300 MHz, *DMSO-d*$_6$):

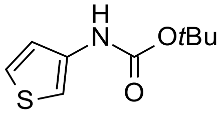

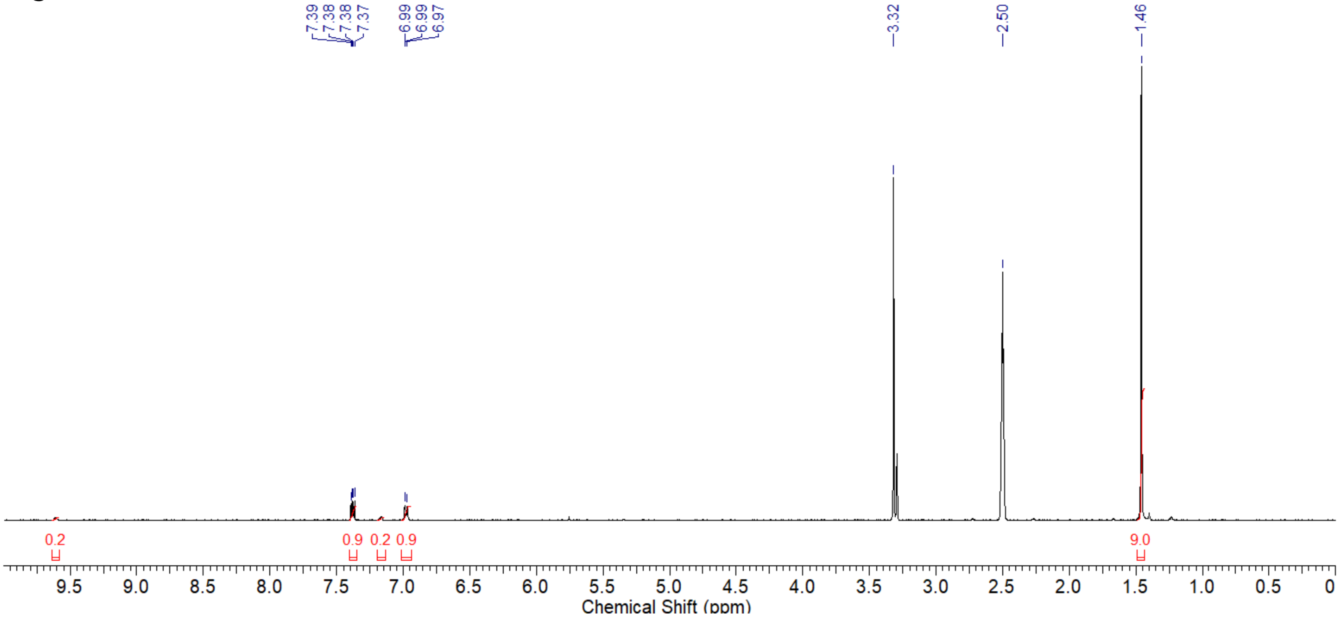

^{13}**C NMR** (101 MHz, *DMSO-d*$_6$)

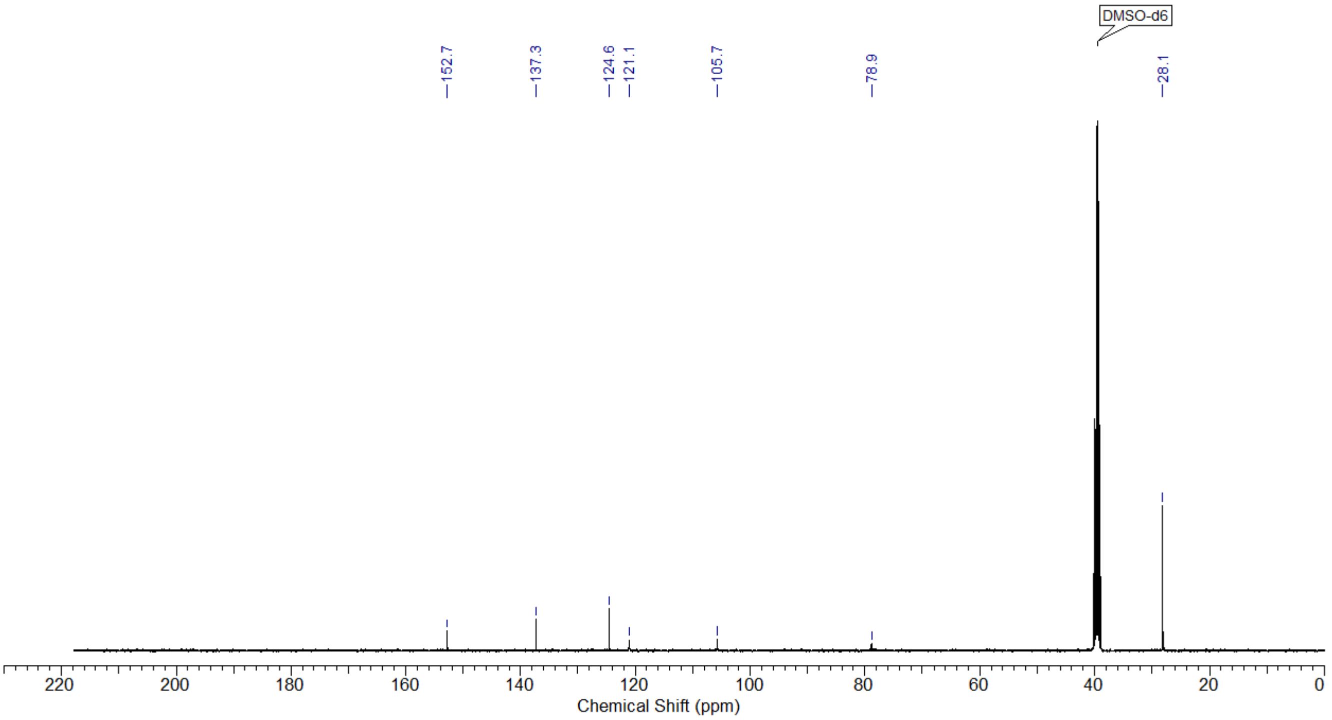

:

(4-Methanesulfonyl-phenyl)carbamic acid tert-butyl ester (**6g**) [CAS 1096711-96-4]

^{1}H NMR (300 MHz, $CDCl_3$):

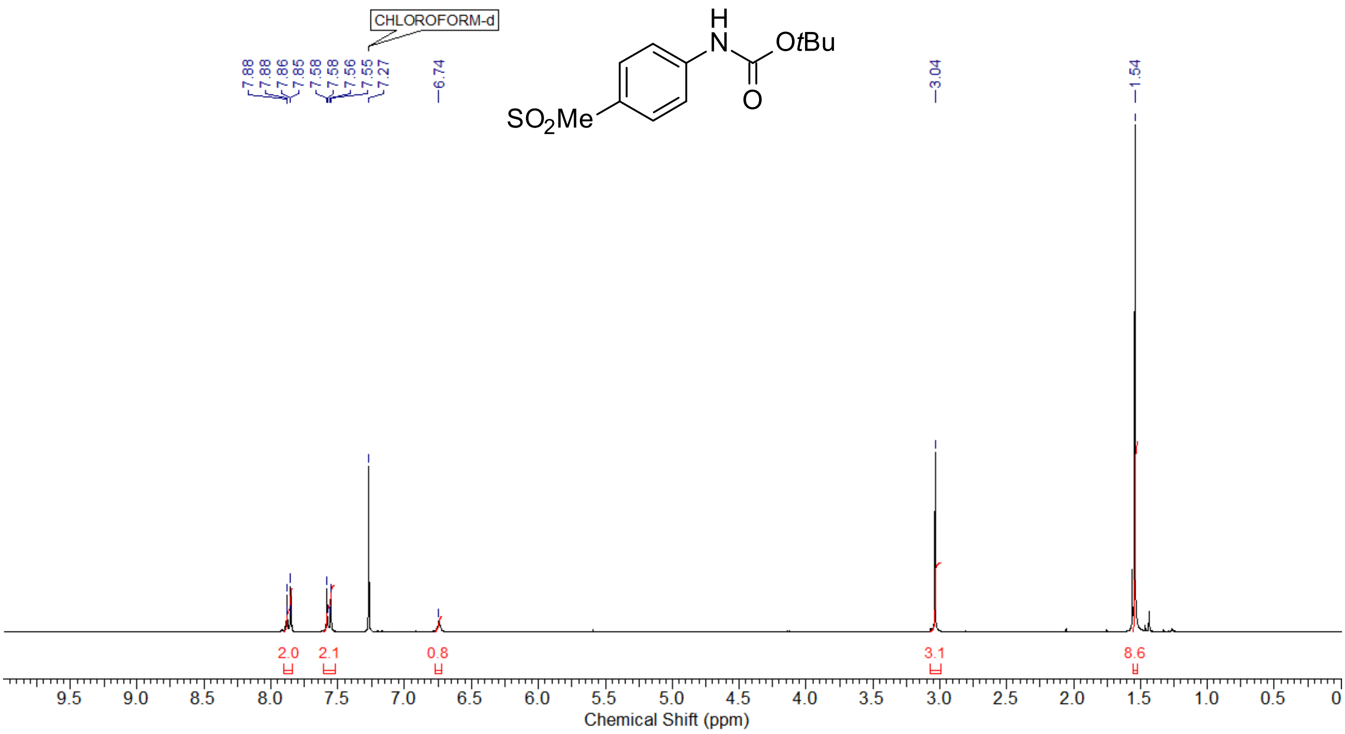

^{13}C NMR (75 MHz, $CDCl_3$):

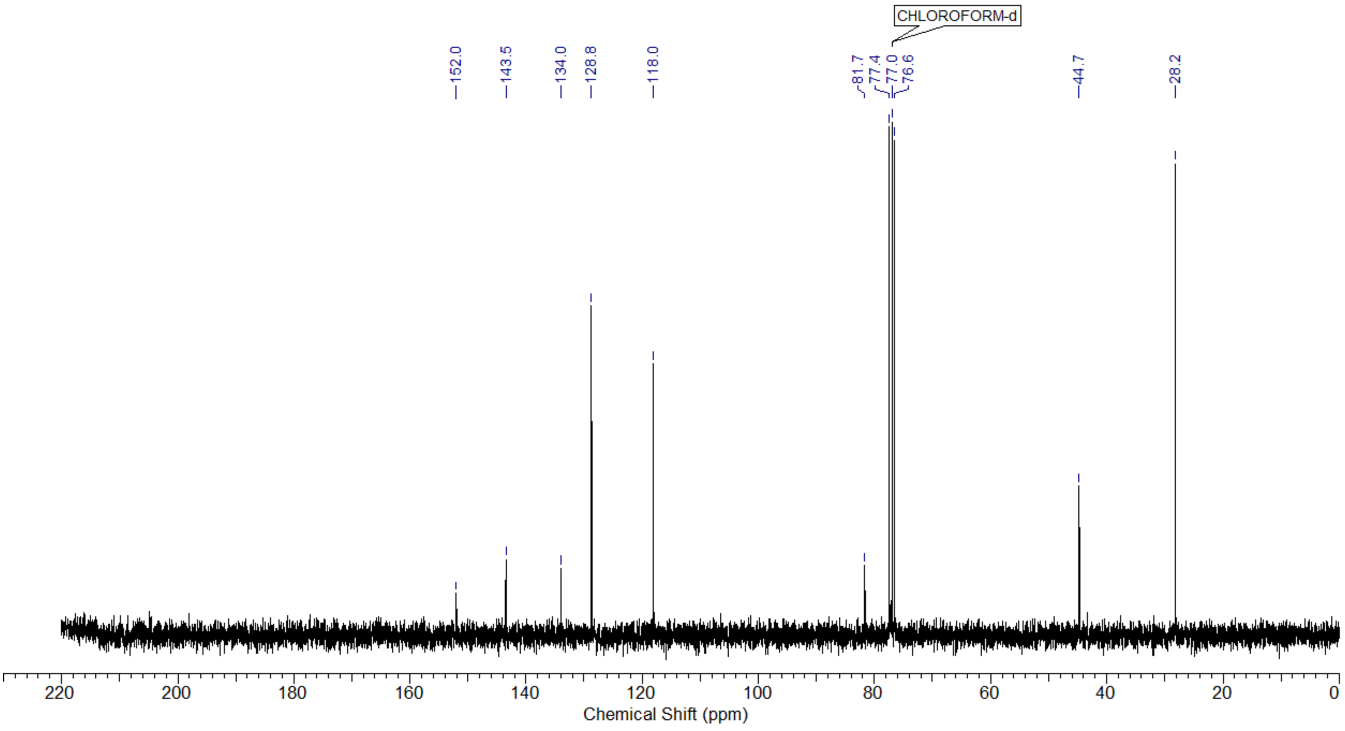

4-Methyl-N-[4-(trifluoromethyl)phenyl]benzenesulfonamide (**7a**) [CAS 107491-54-3]

^{1}H NMR (300 MHz, $CDCl_3$):

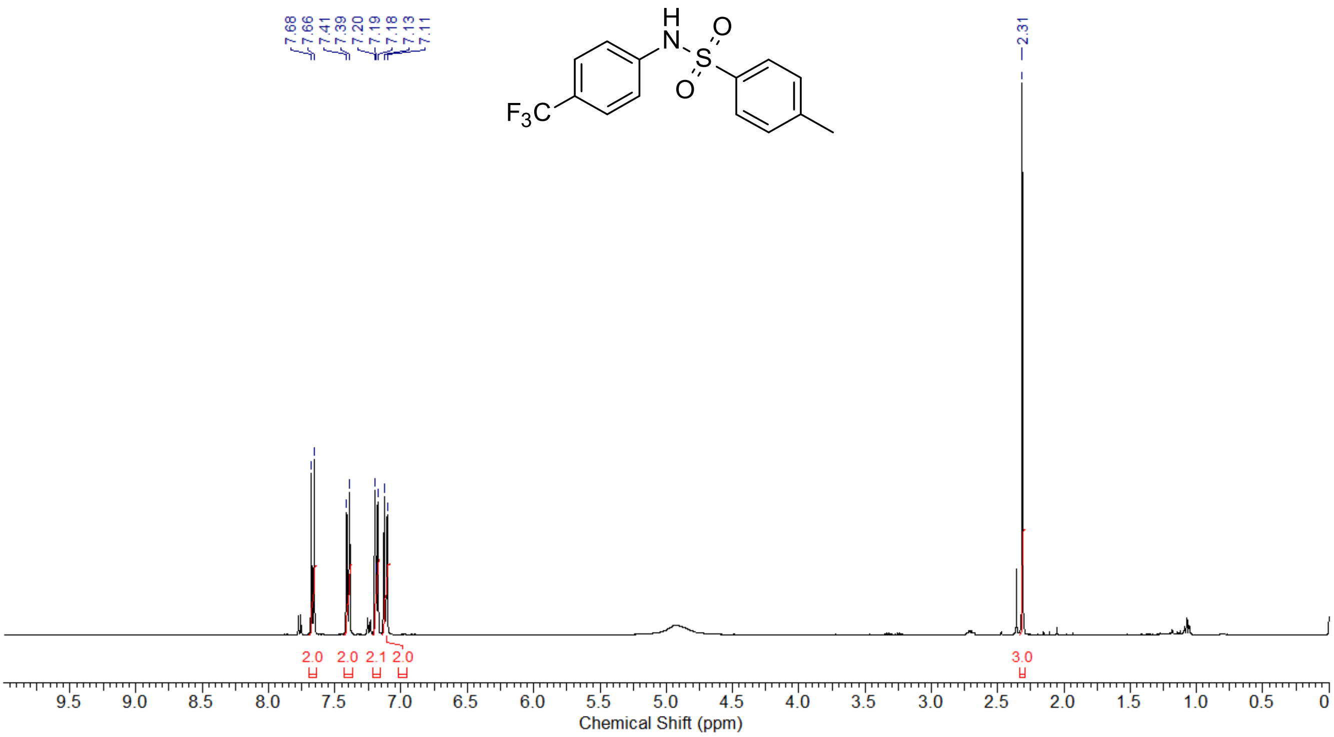

^{13}C NMR (75 MHz, $CDCl_3$):

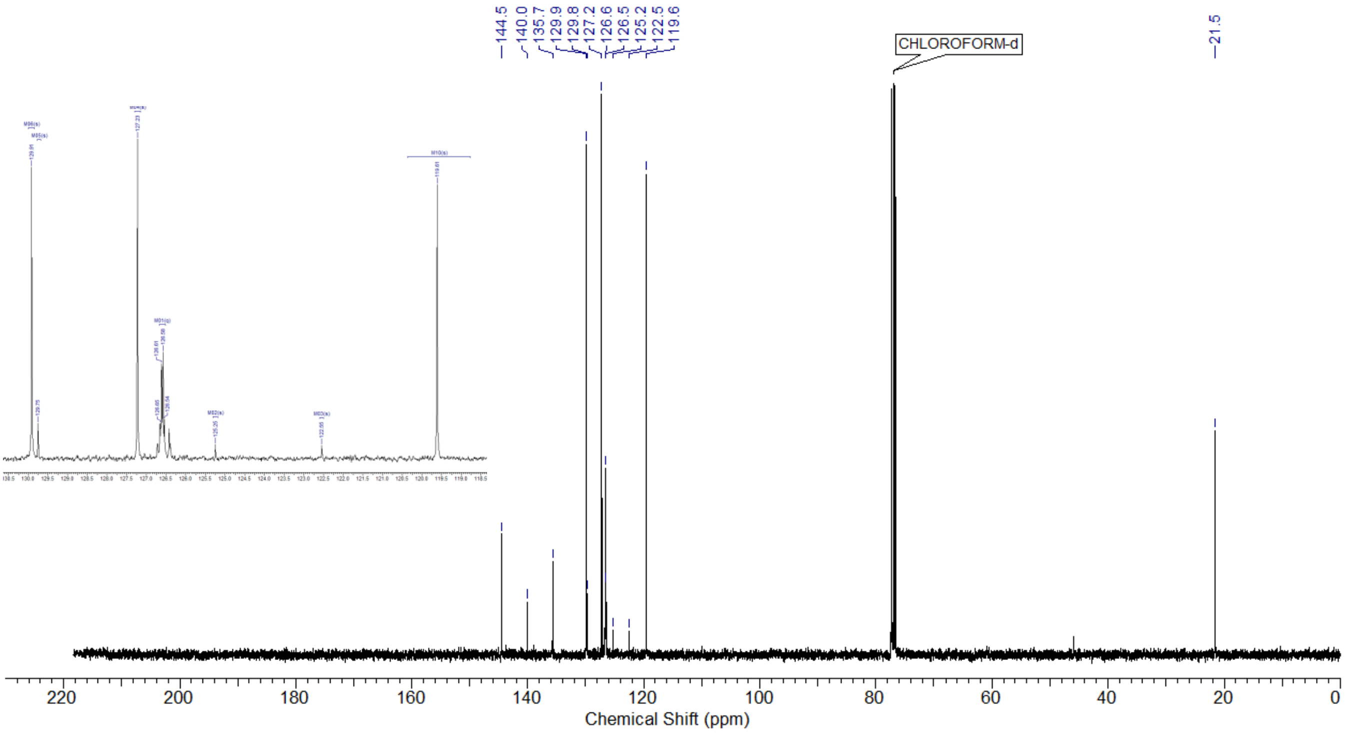

N-(4-acetylphenyl)-4-methyl-benzenesulfonamide (**7b**) [CAS 5317-94-2]

^{1}H NMR (300 MHz, $CDCl_3$):

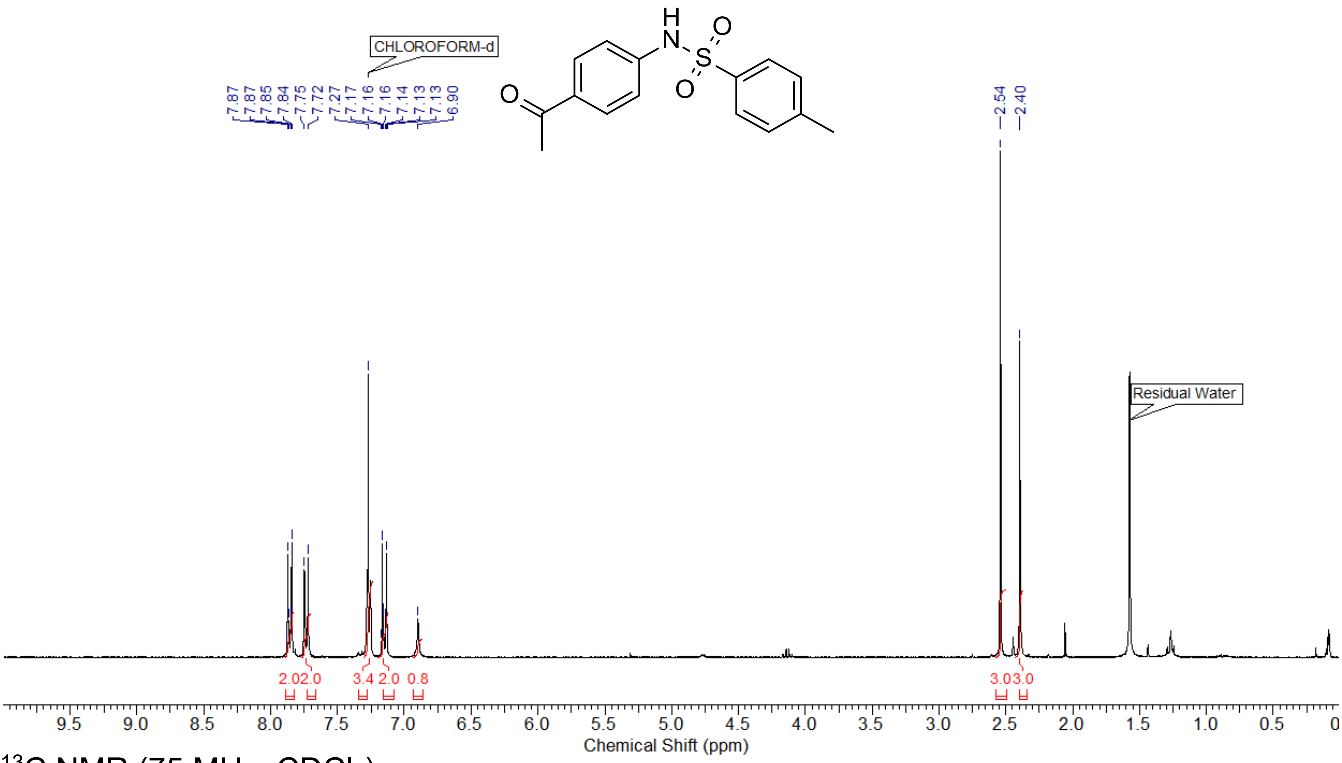

^{13}C NMR (75 MHz, $CDCl_3$):

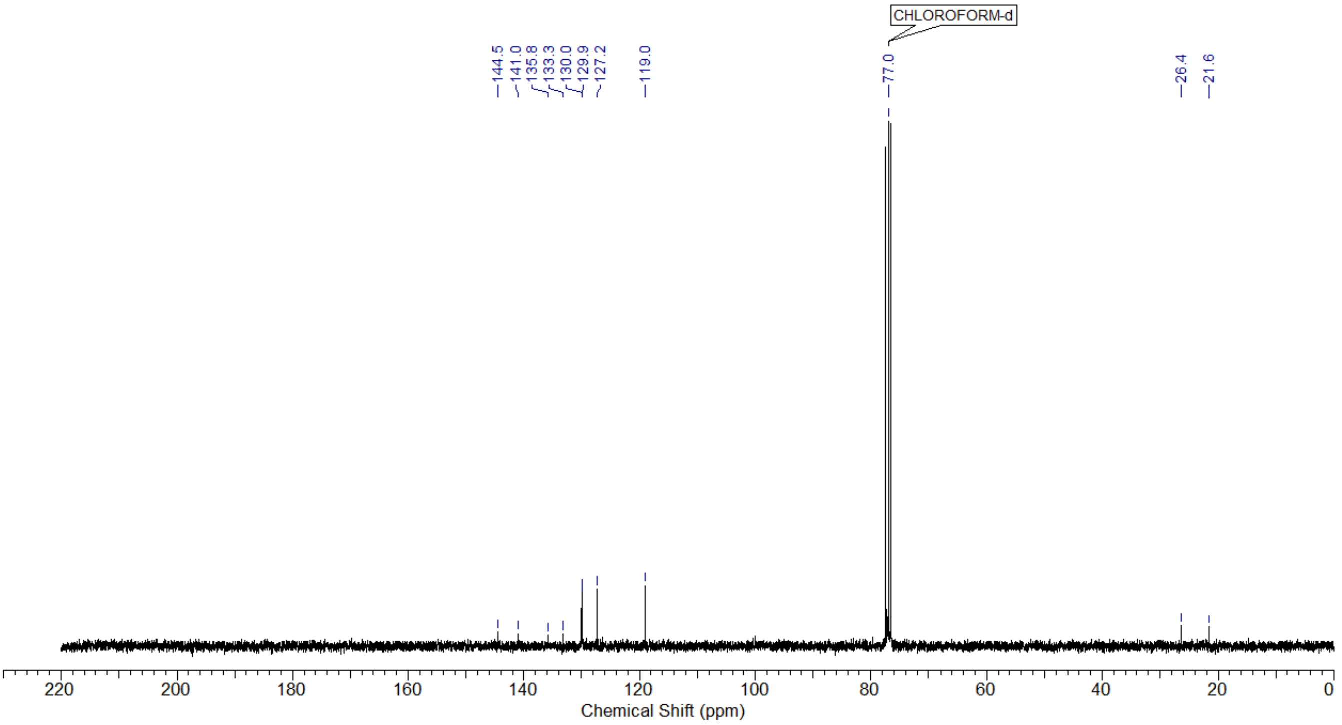

Methyl 4-(p-tolylsulfonylamino)benzoate (**7c**) [CAS 158038-67-6]

^{1}H NMR (300 MHz, DMSO-d^6):

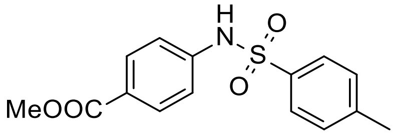

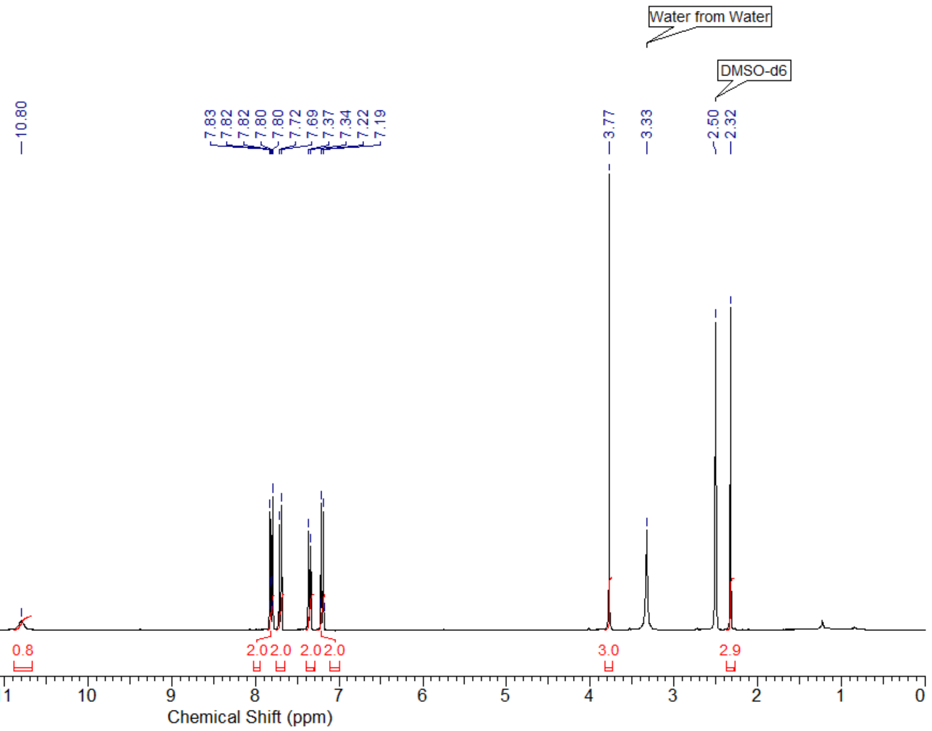

^{13}C NMR (75 MHz, DMSO-d^6):

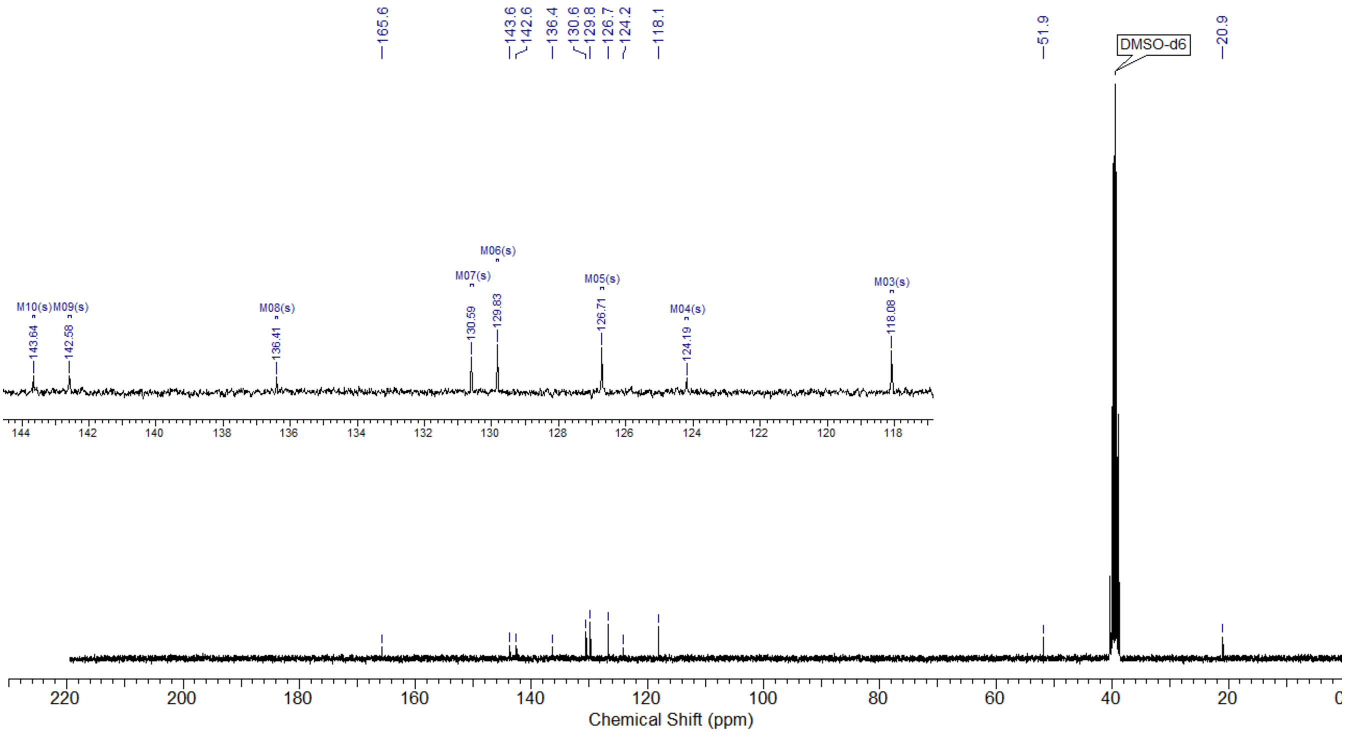

4-methyl-*N*-(*o*-tolyl)benzenesulfonamide (**7d**) [CAS 80-28-4]

^{1}H NMR (300 MHz, $CDCl_3$):

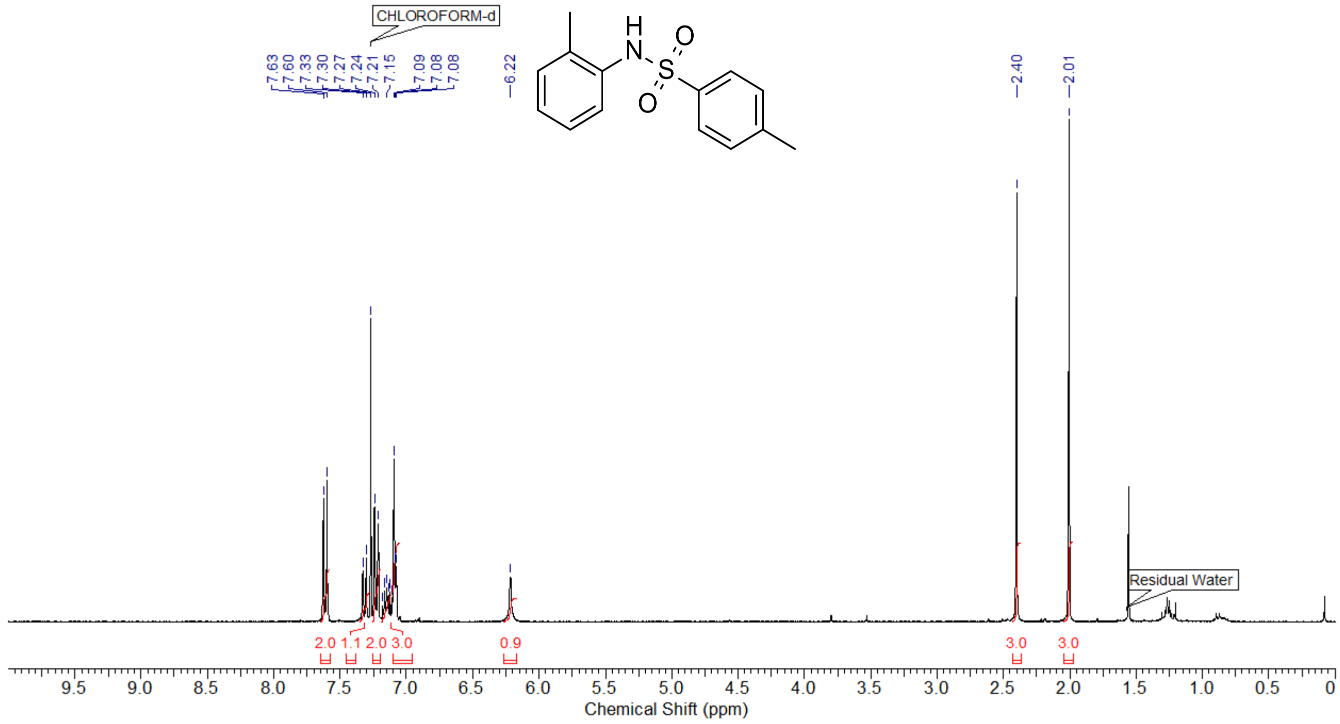

^{13}C NMR (75 MHz, $CDCl_3$):

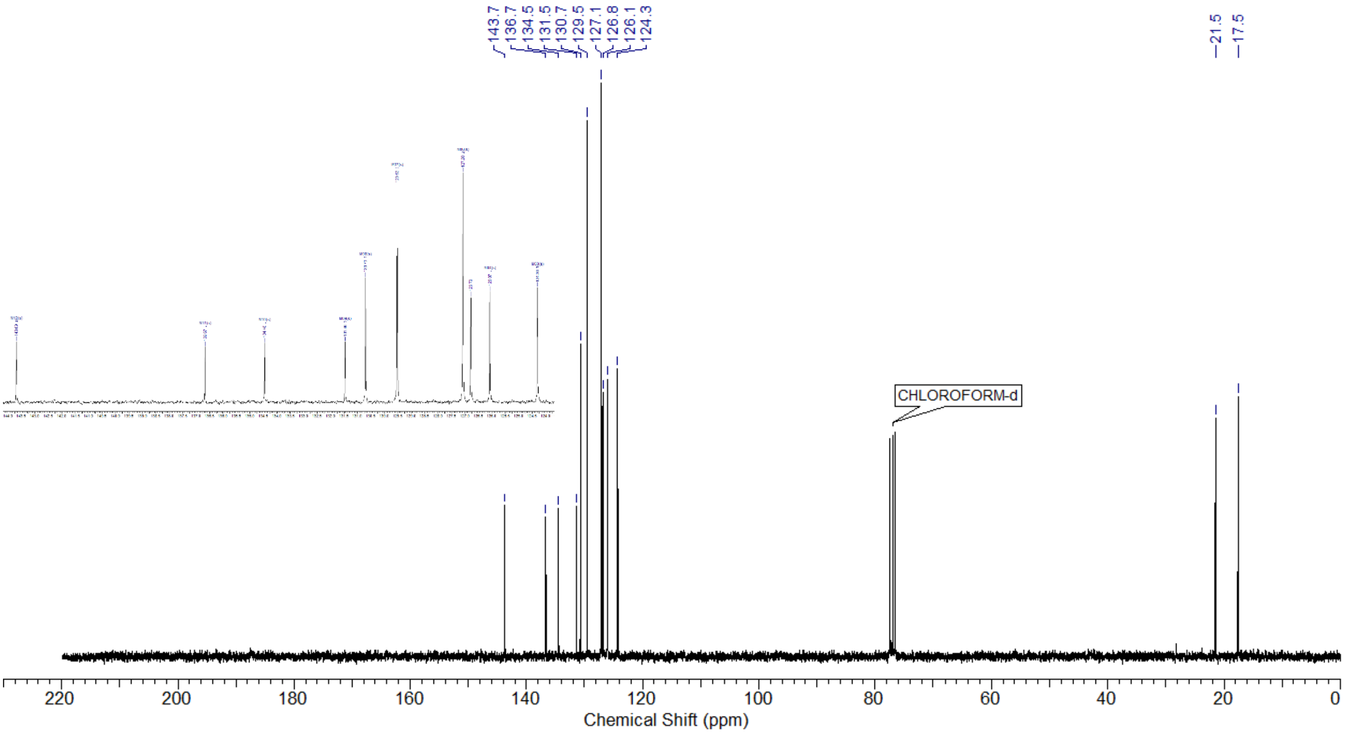

4-methyl-N-(1-naphthyl)benzenesulfonamide (**7e**) [CAS 18271-17-5]

^1H NMR (300 MHz, $CDCl_3$):

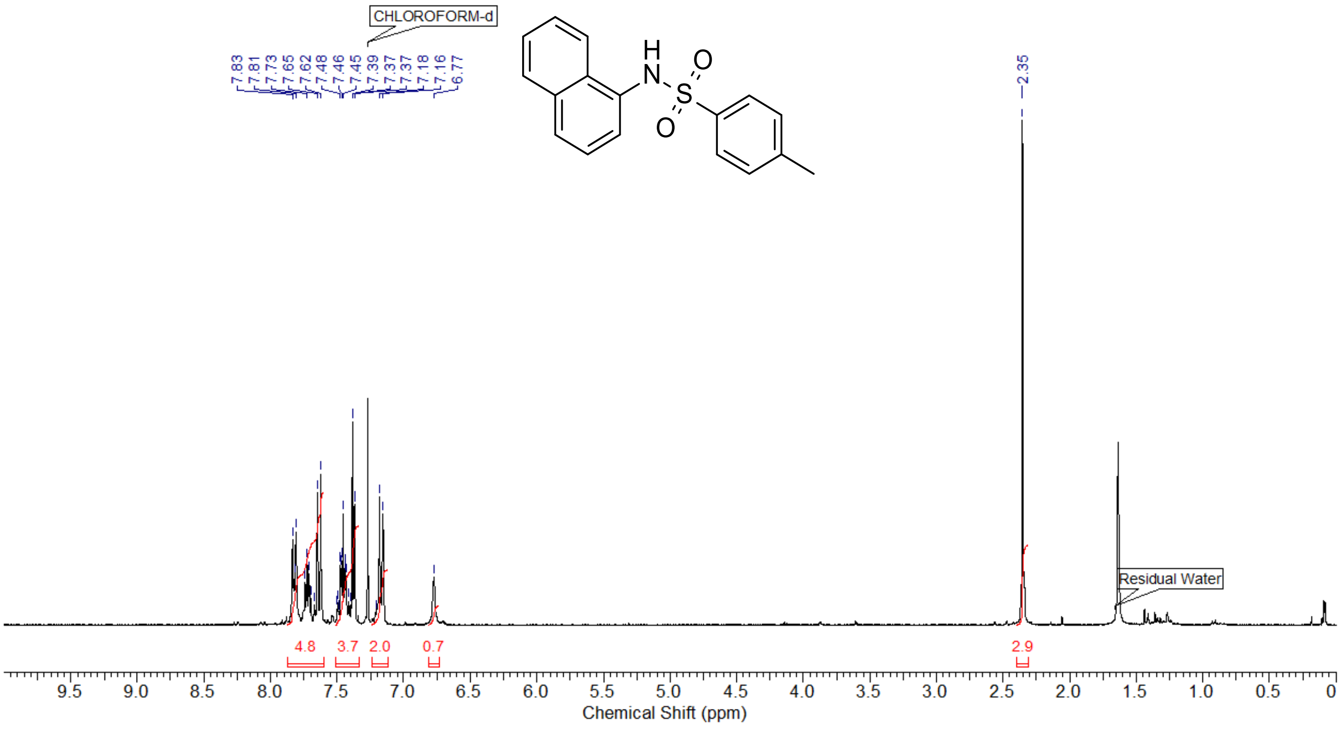

^{13}C NMR (75 MHz, $CDCl_3$):

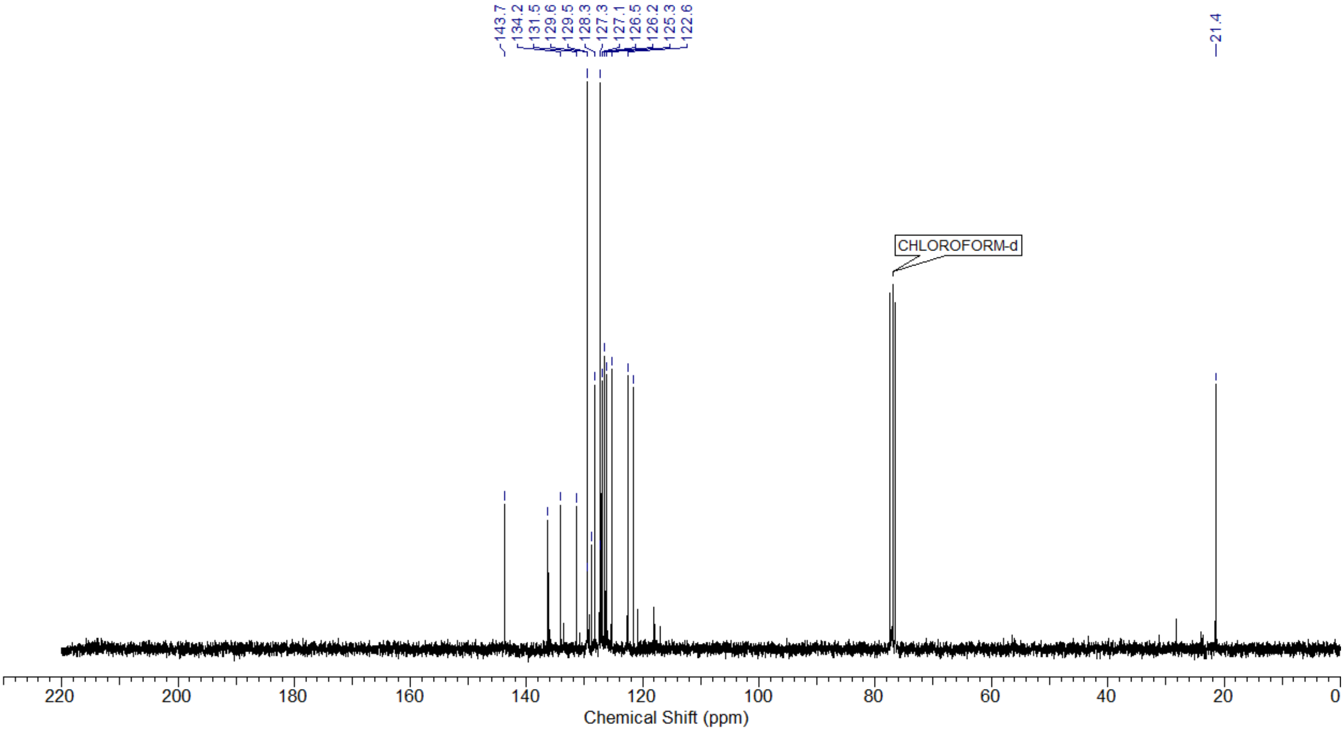

4-Methylaniline (**8a**) [CAS: 106-49-0]

^{1}H NMR (300 MHz, $CDCl_3$):

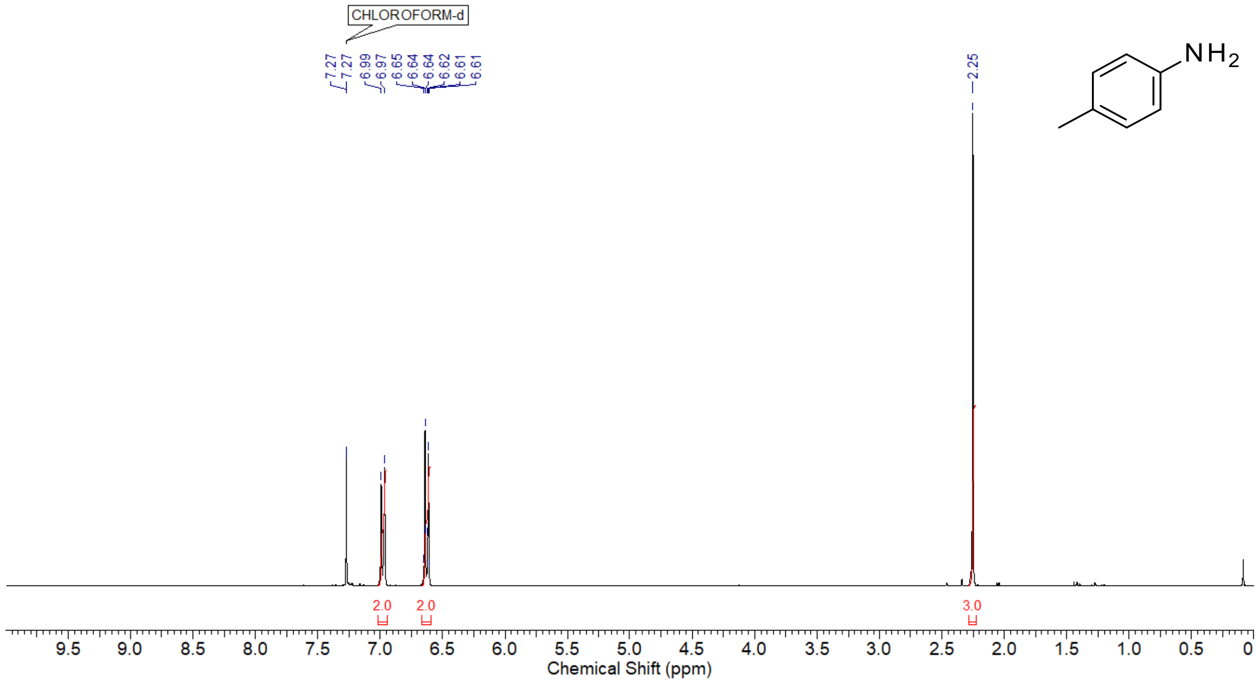

^{13}C NMR (75 MHz, $CDCl_3$):

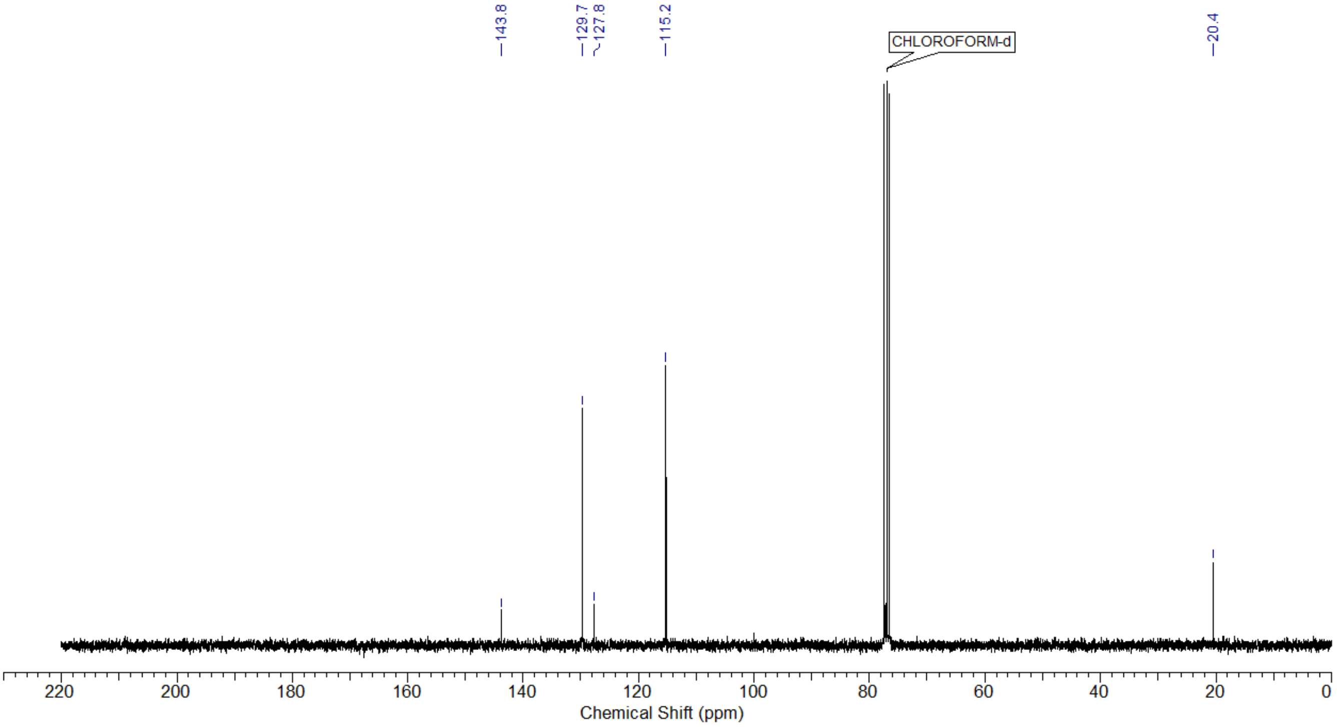

4-Methoxyaniline (**8b**) [CAS: 104-94-9]

^{1}H NMR (300 MHz, $CDCl_3$):

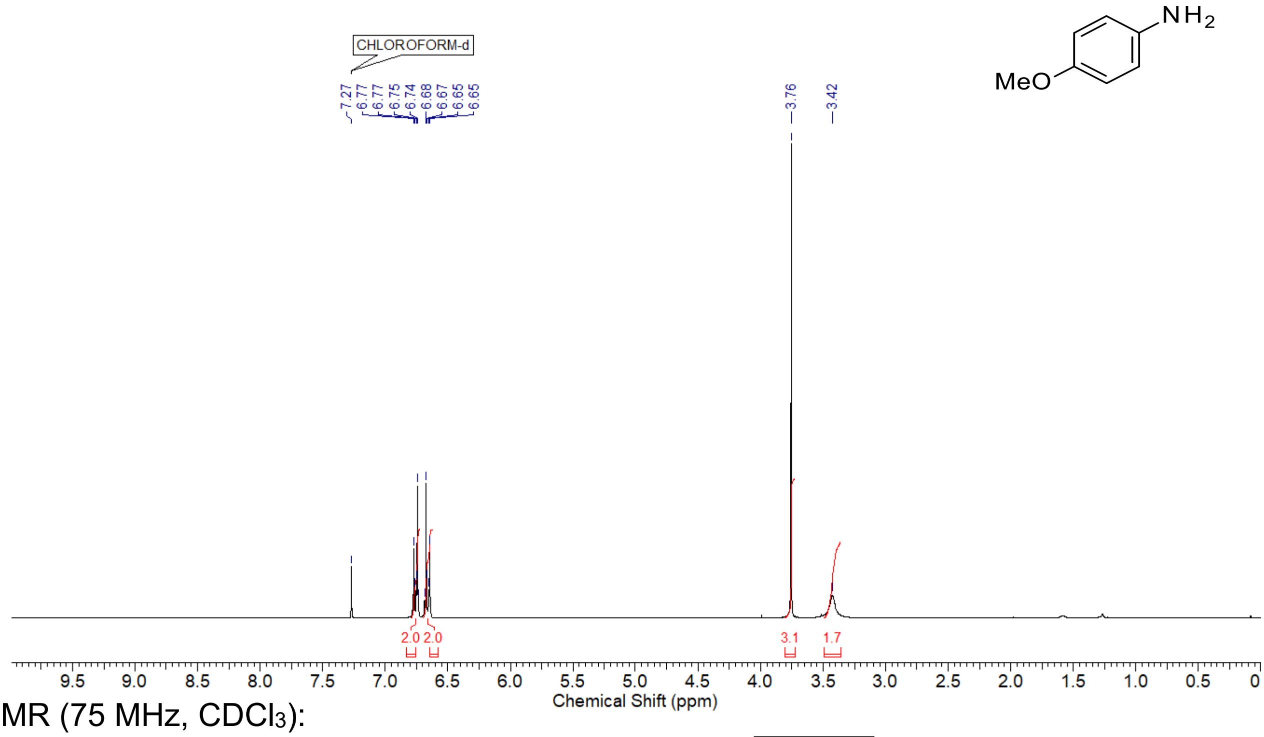

^{13}C NMR (75 MHz, $CDCl_3$):

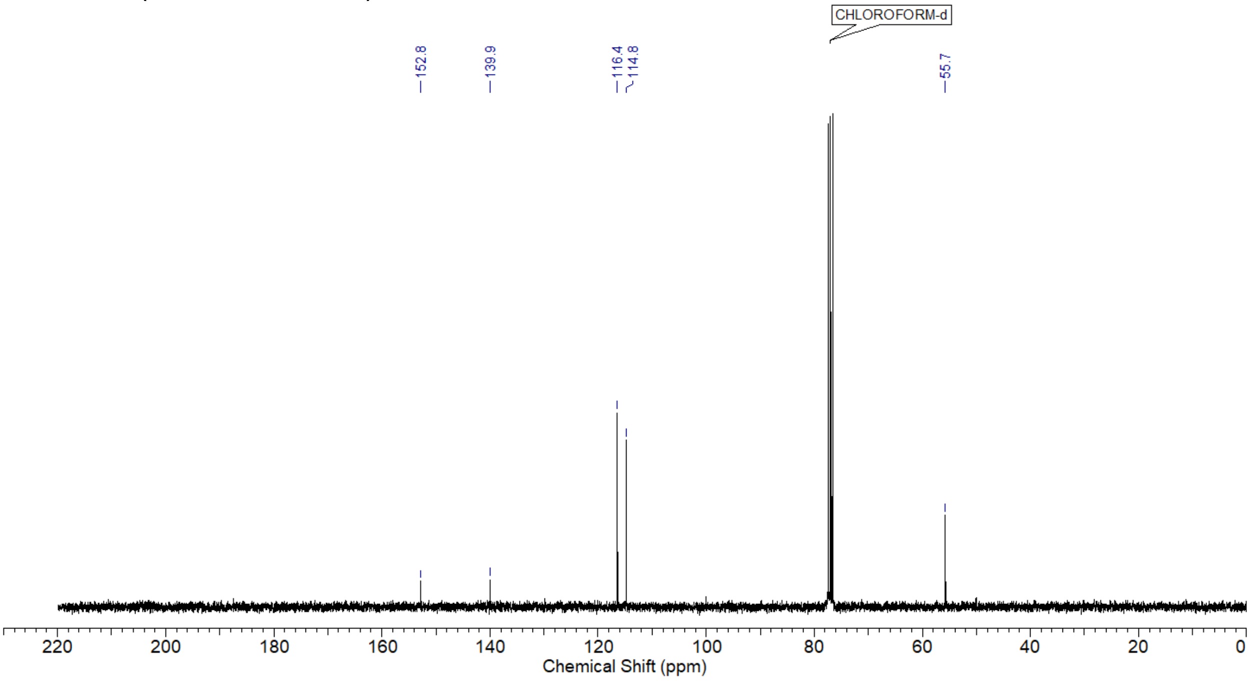

Quinolin-6-amine (**8c**) [CAS: 580-15-4]:

^{1}H NMR (300 MHz, $CDCl_3$):

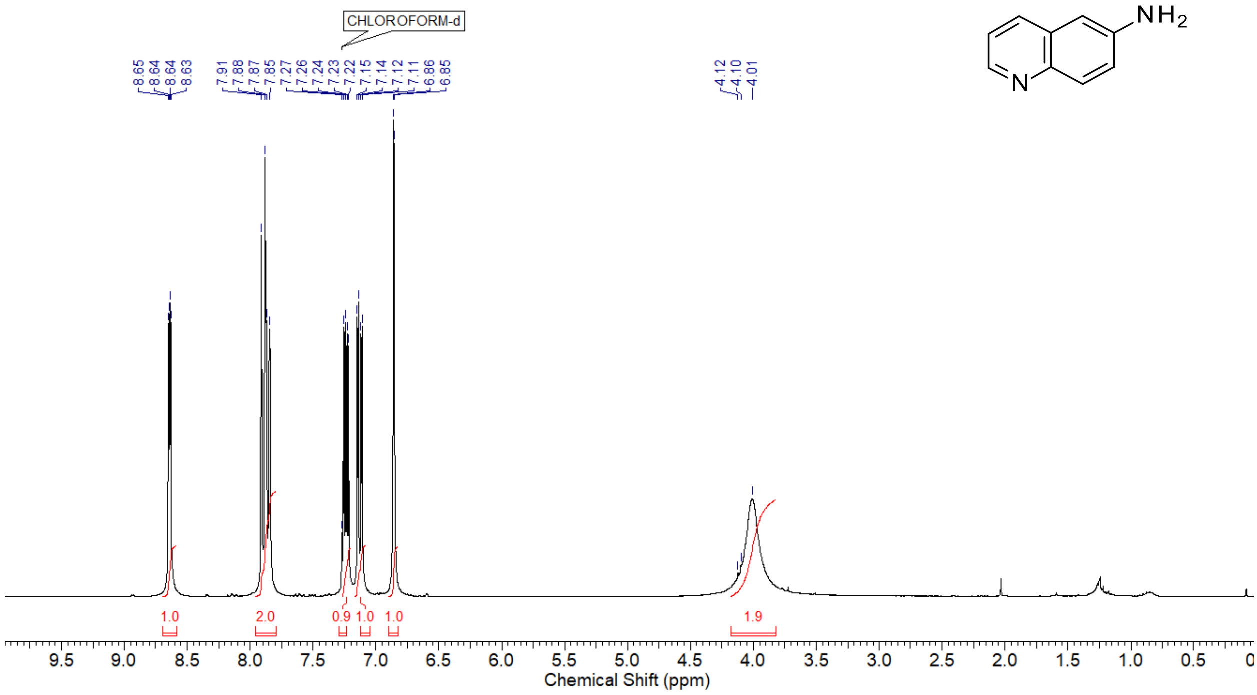

^{13}C NMR (75 MHz, $CDCl_3$):

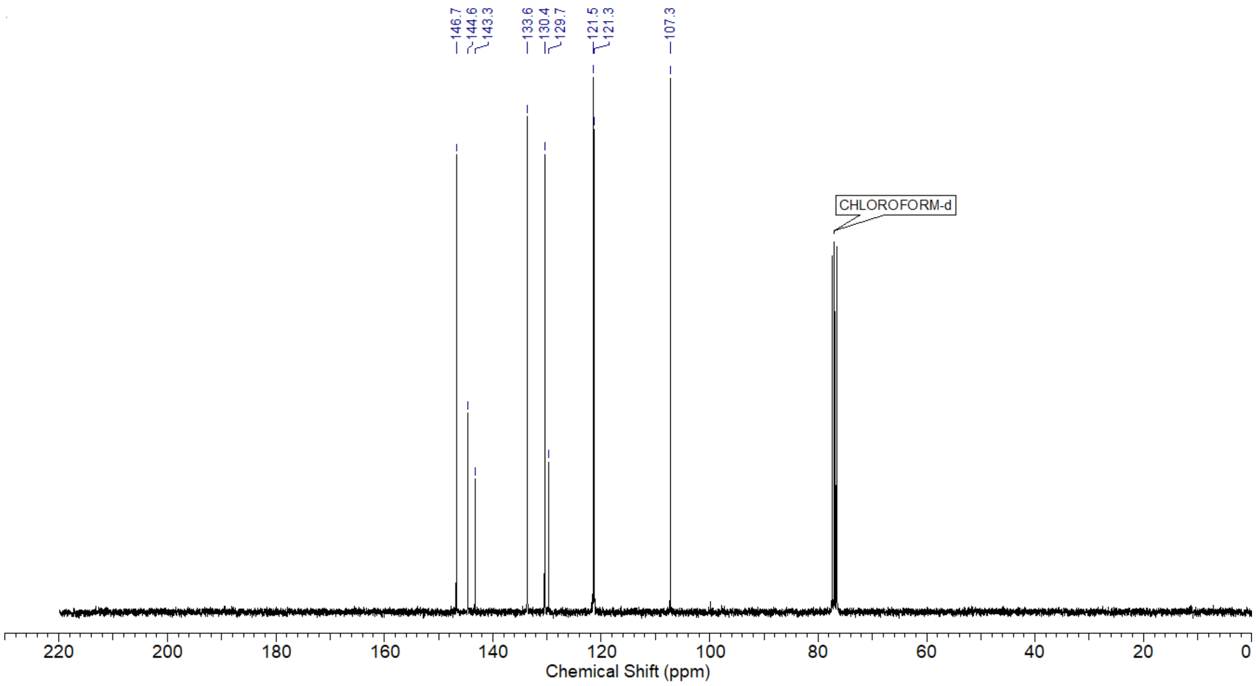

4-Aminobiphenyl (**8d**) [CAS: 92-67-1]

^{1}H NMR (300 MHz, $CDCl_3$):

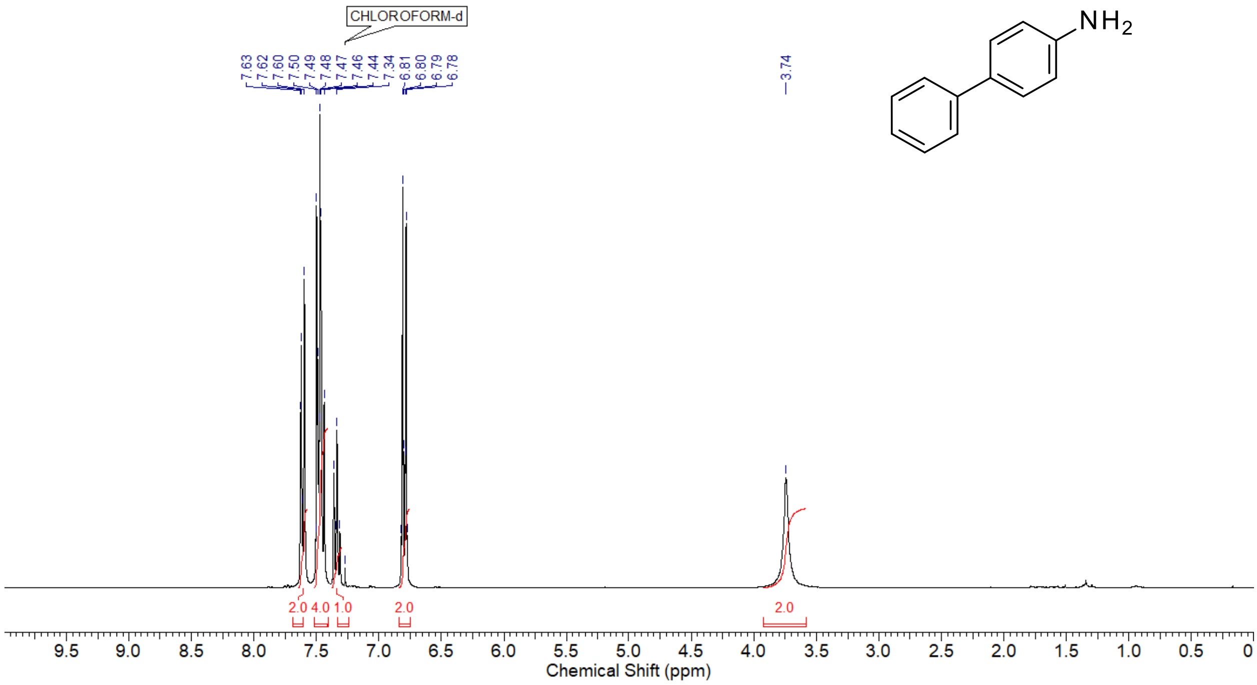

^{13}C NMR (75 MHz, $CDCl_3$):

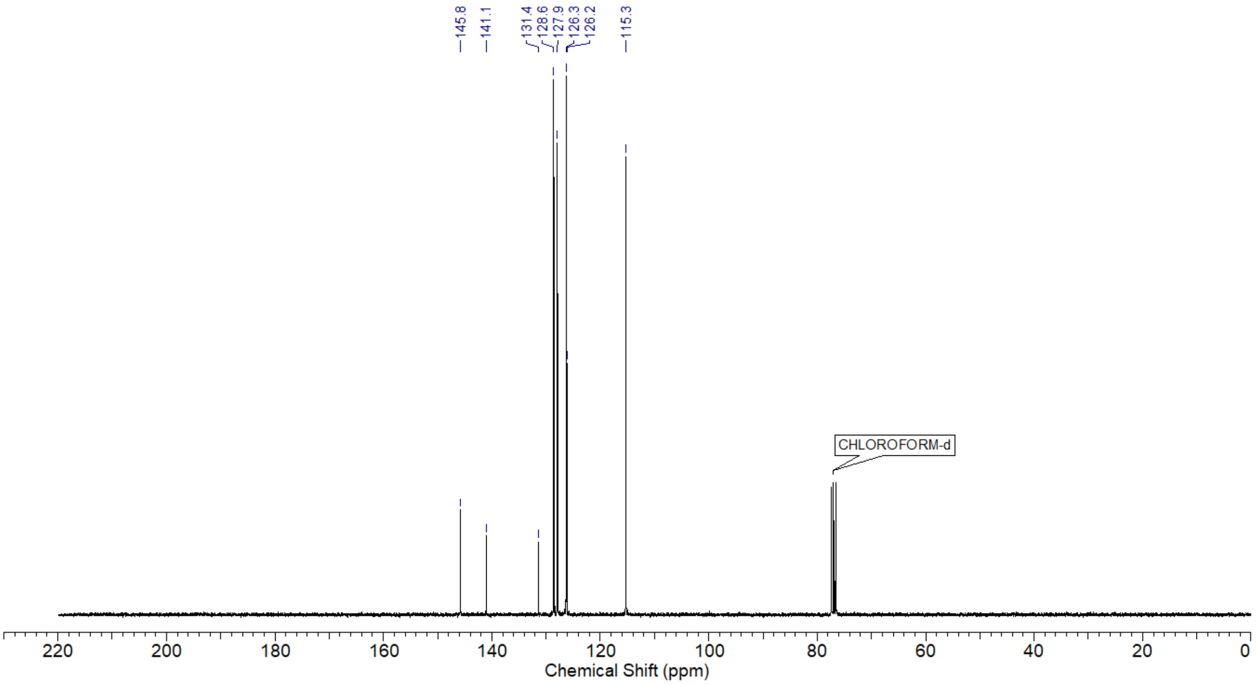

<u>*N*-cyclopropyl-4-(trifluoromethyl)aniline</u> (**9a**) [CAS 1249999-66-3]

^{1}H NMR (300 MHz, $CDCl_3$):

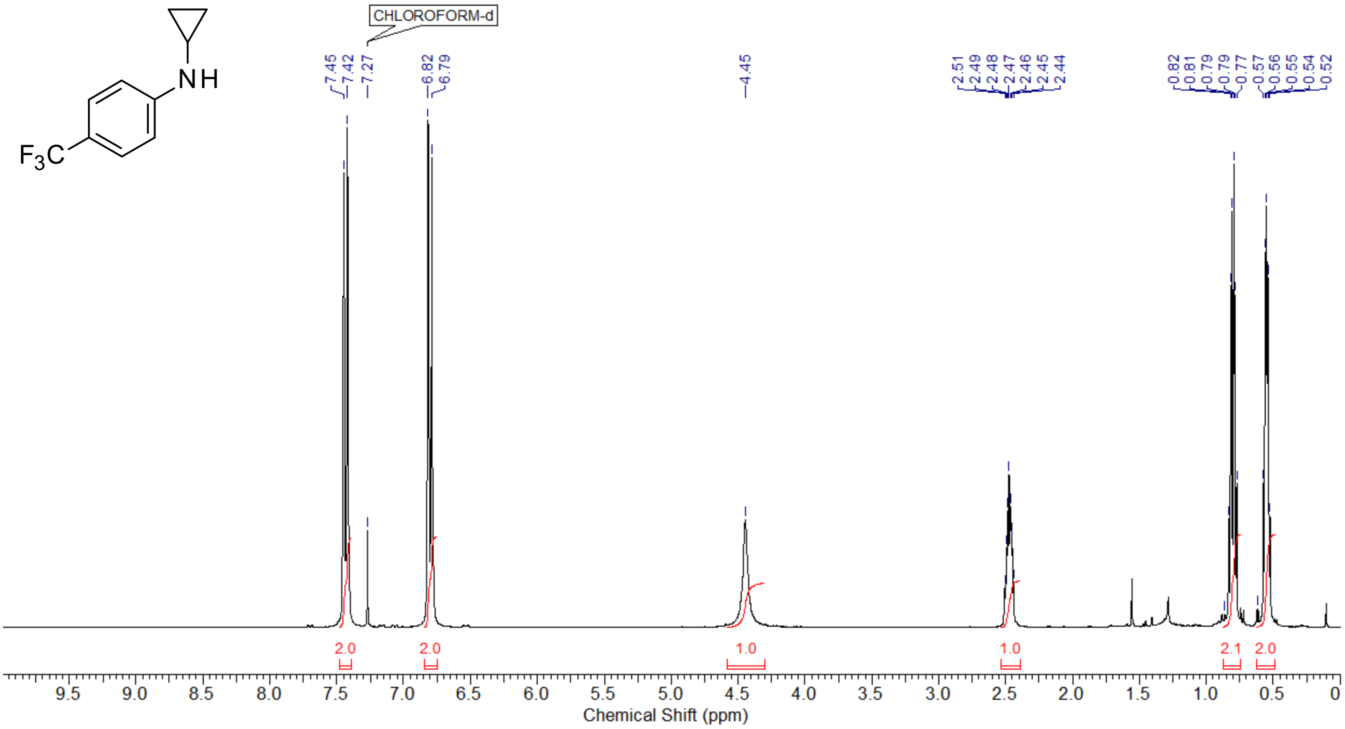

^{13}C NMR (75 MHz, $CDCl_3$):

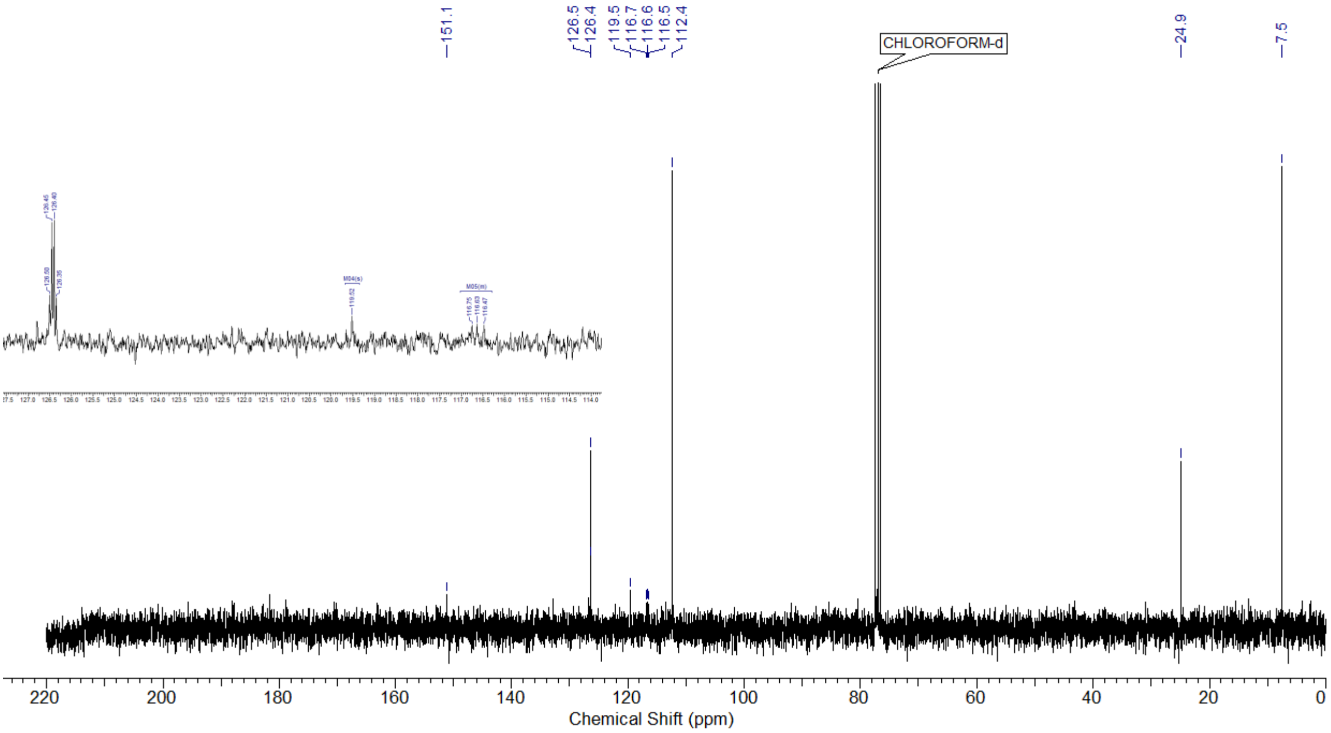

^{19}F NMR (76 MHz, $CDCl_3$, $C_6H_4F_2$):

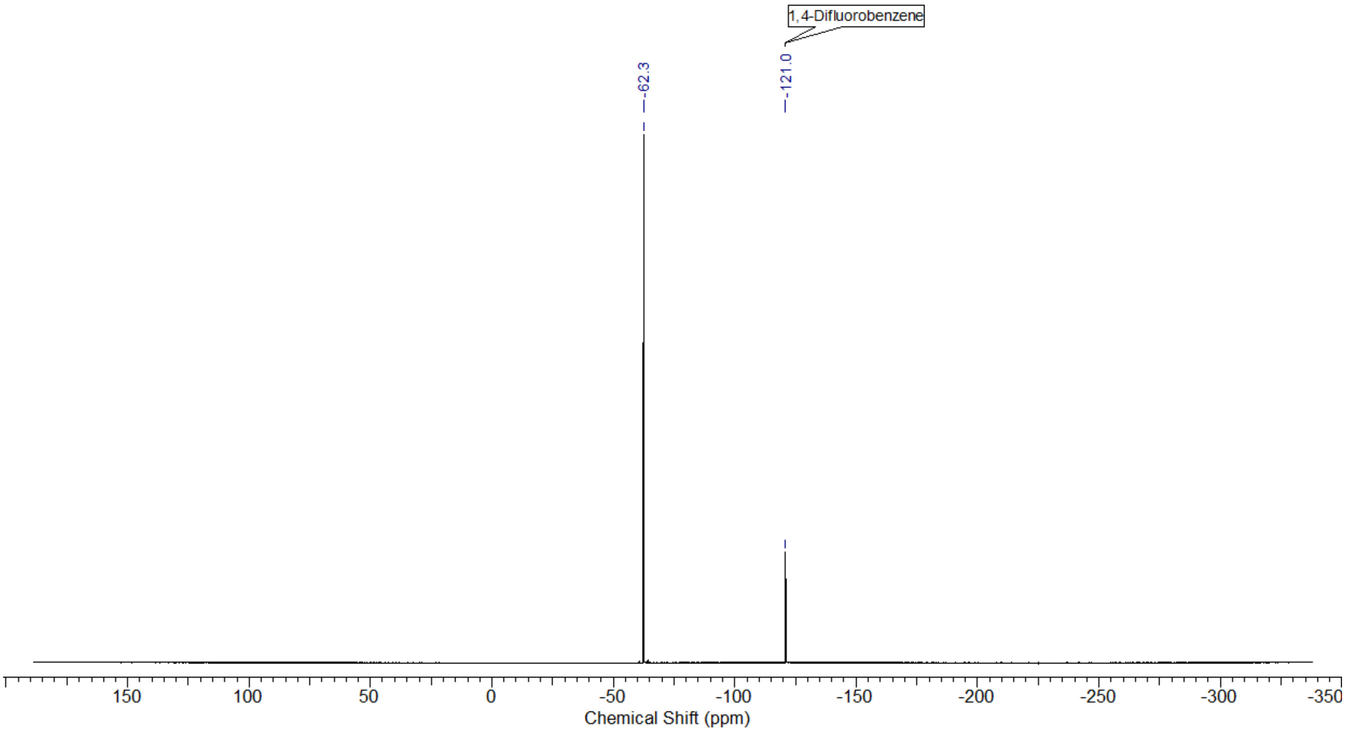

Ethyl 4-(cyclopropylamino)benzoate (**9b**) [CAS 112033-48-4]

^{1}H NMR (300 MHz, $CDCl_3$):

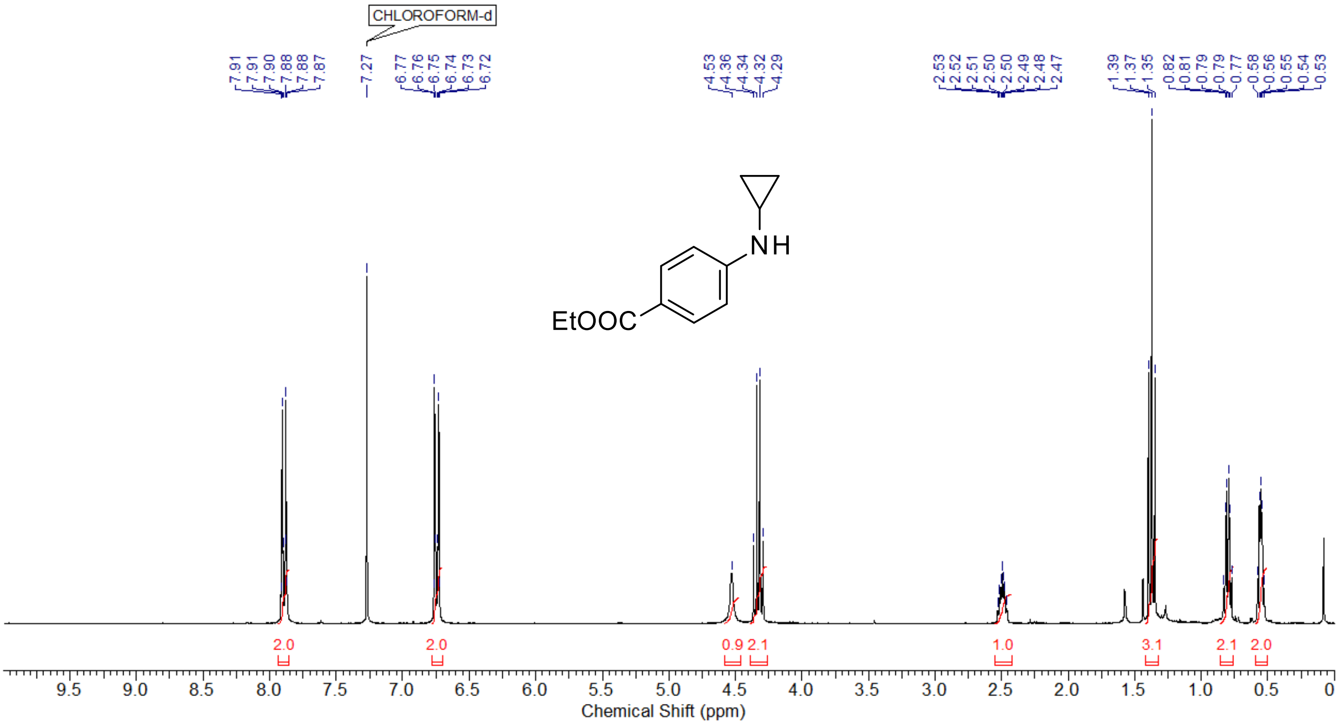

^{13}C NMR (75 MHz, $CDCl_3$):

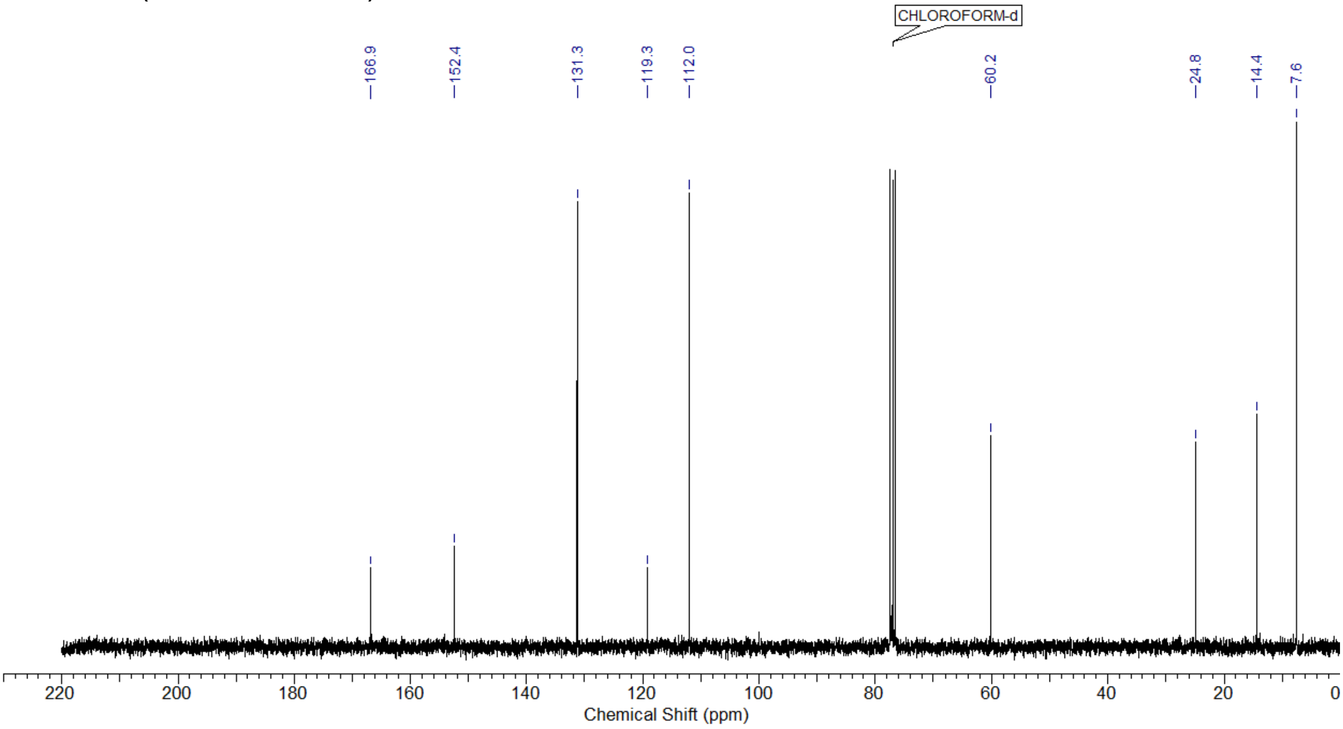

<u>*N*-cyclopropylpyridin-2-amine</u> (**9c**) [CAS 950577-07-8]

^{1}H NMR (300 MHz, $CDCl_3$):

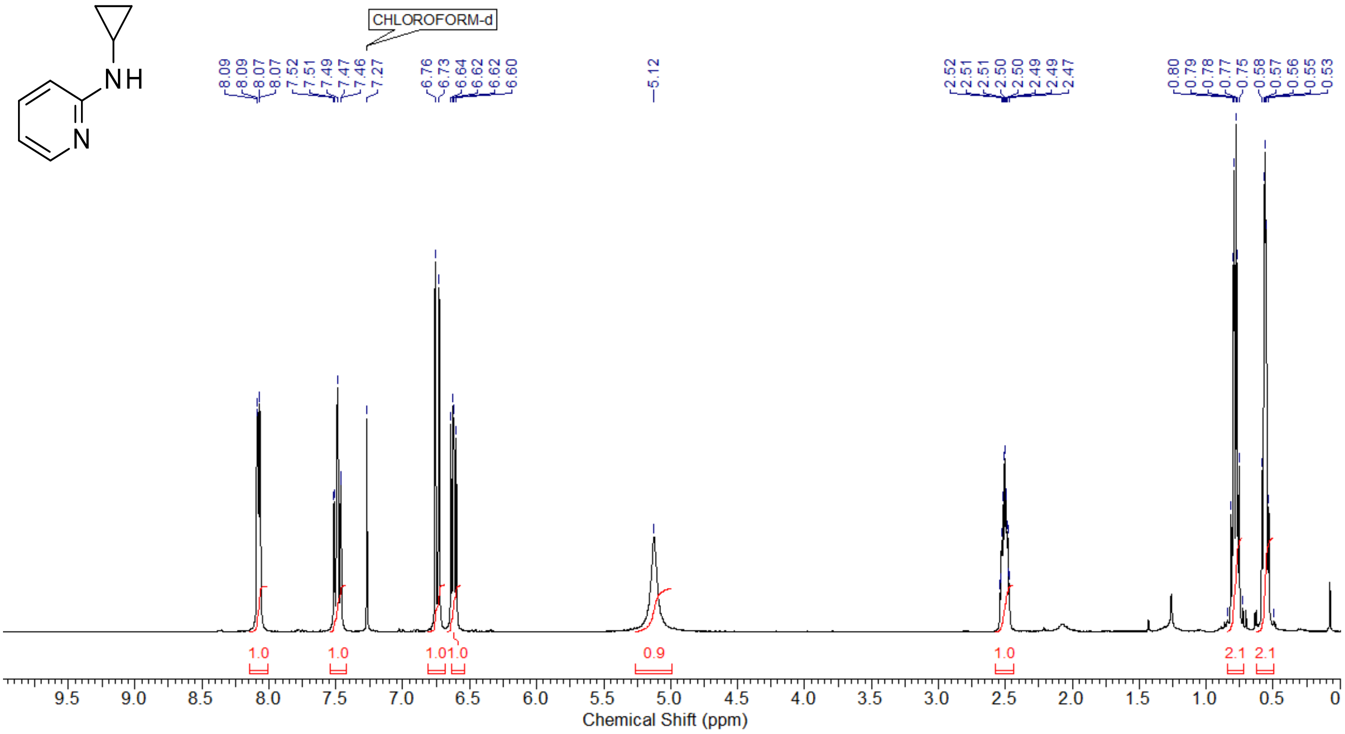

^{13}C NMR (75 MHz, $CDCl_3$):

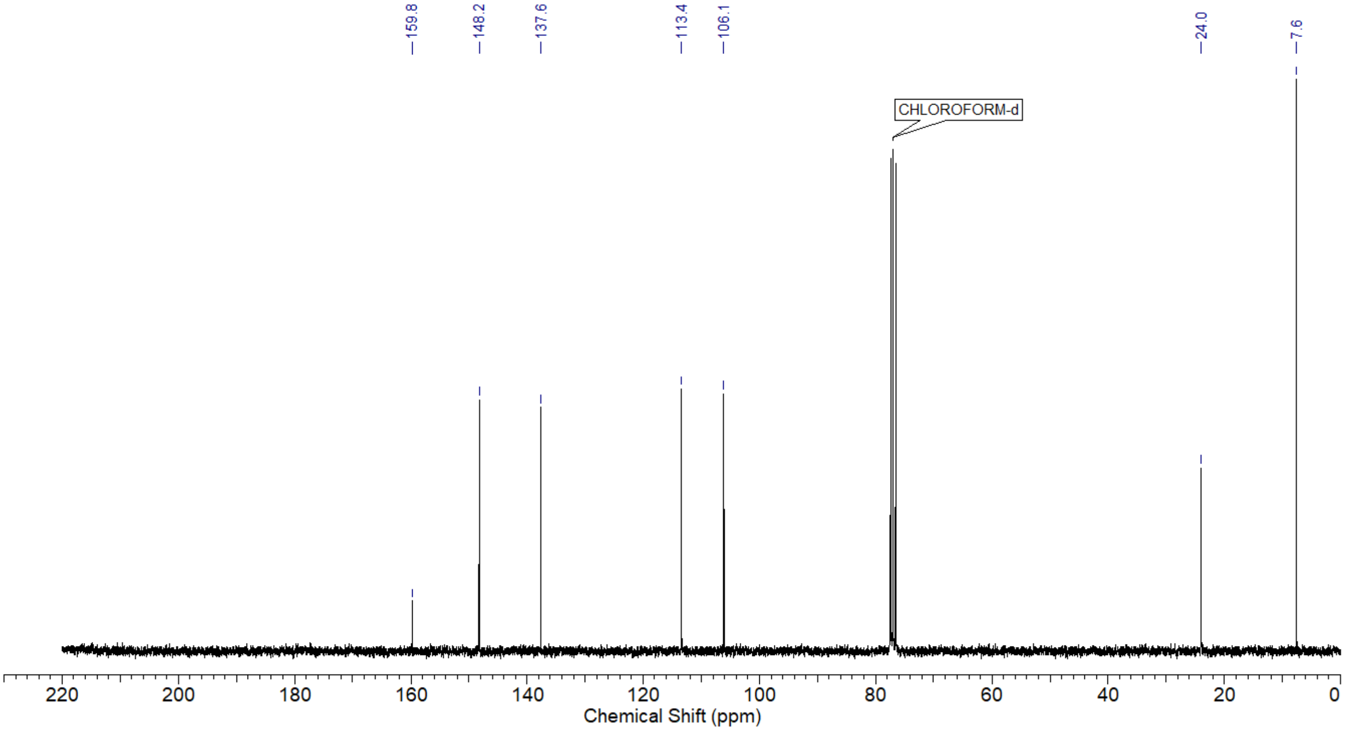

N-cyclopropylquinolin-2-amine (**9d**) [CAS 855760-19-9]

^{1}H NMR (300 MHz, $CDCl_3$):

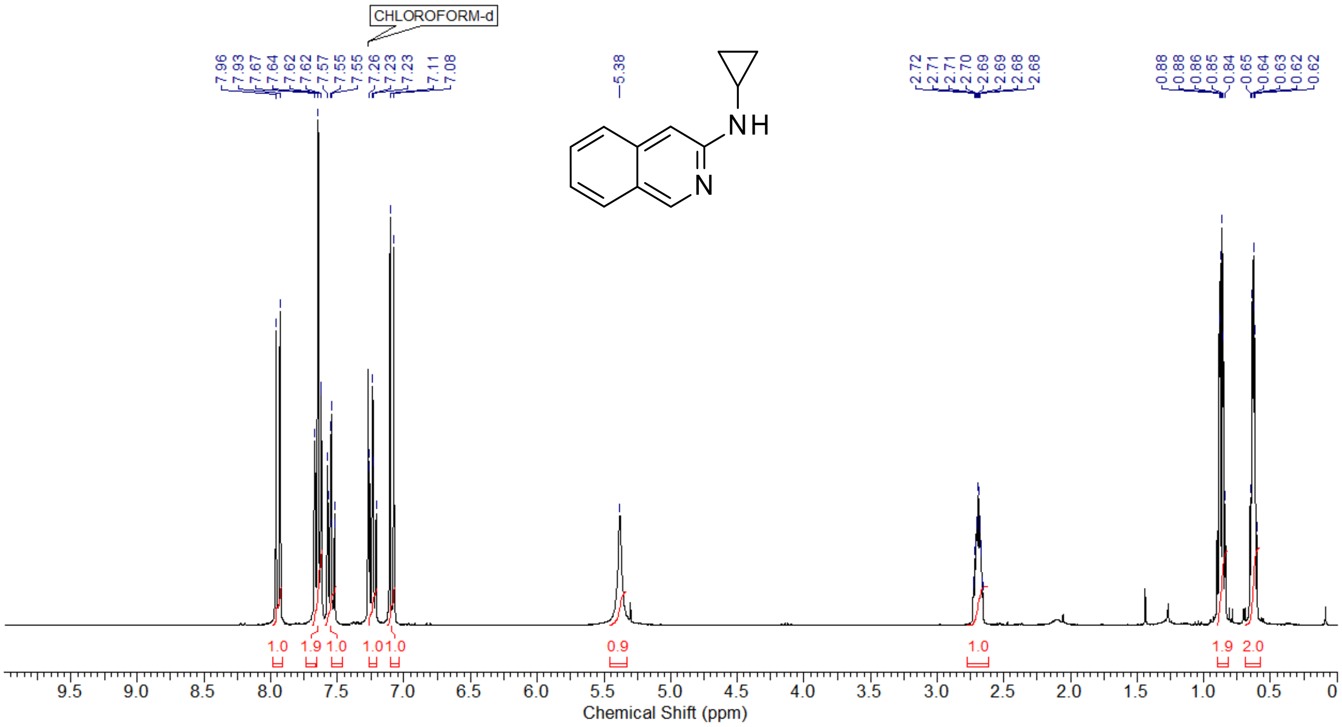

^{13}C NMR (75 MHz, $CDCl_3$):

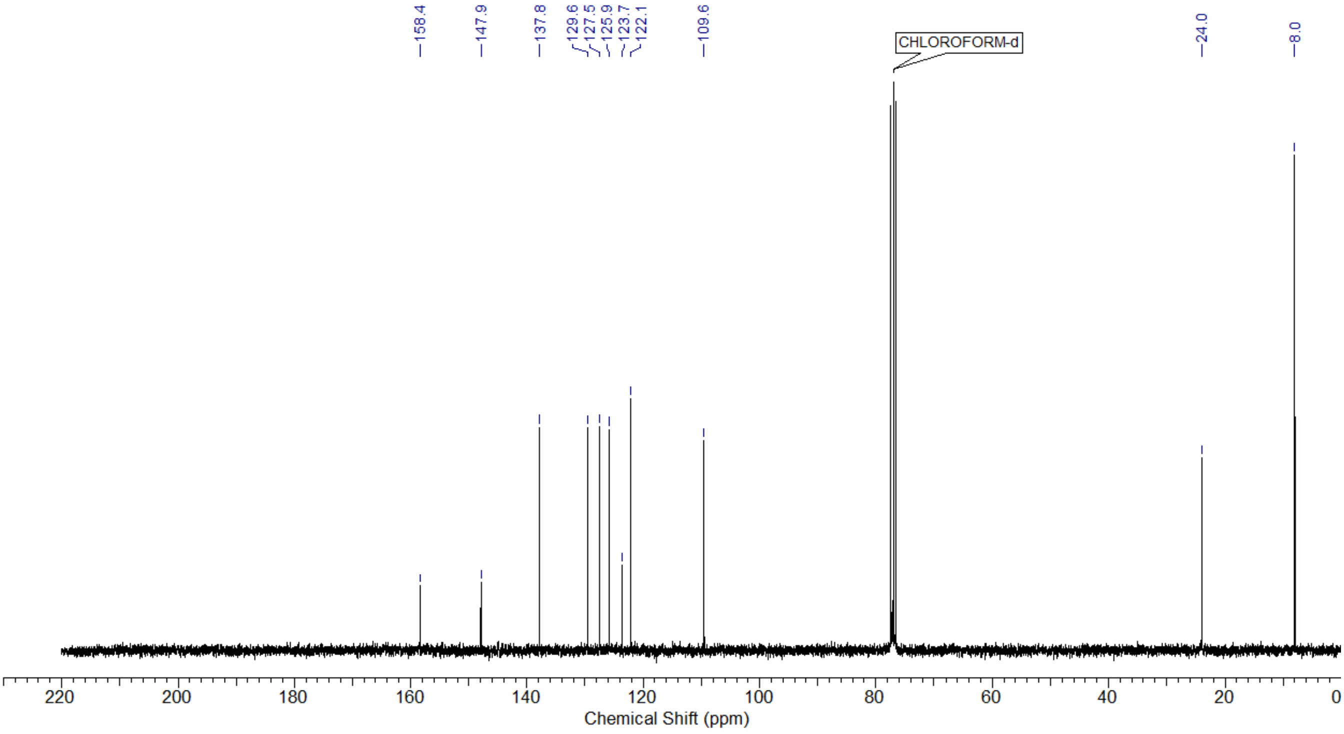

N-Cyclopropyl-2-methyl-5-benzoxazolamine (**9e**) *[CAS 1509662-56-9]*

^{1}H NMR (300 MHz, $CDCl_3$)

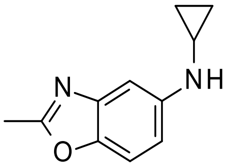

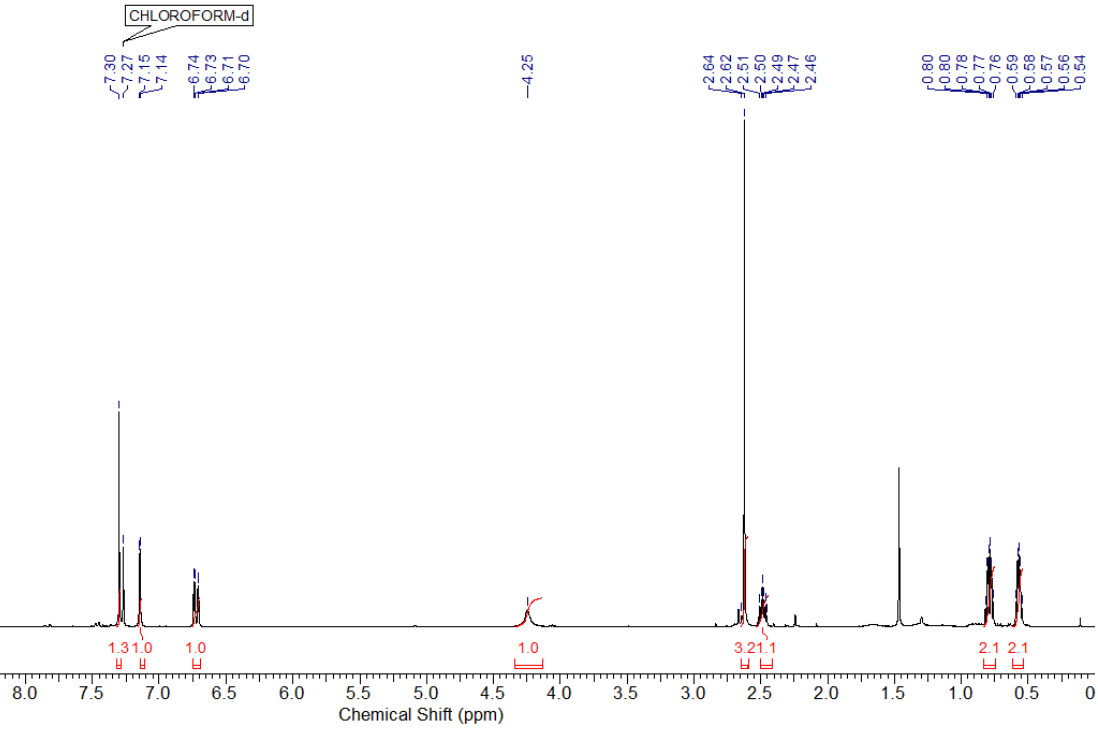

^{13}C NMR (75 MHz, $CDCl_3$):

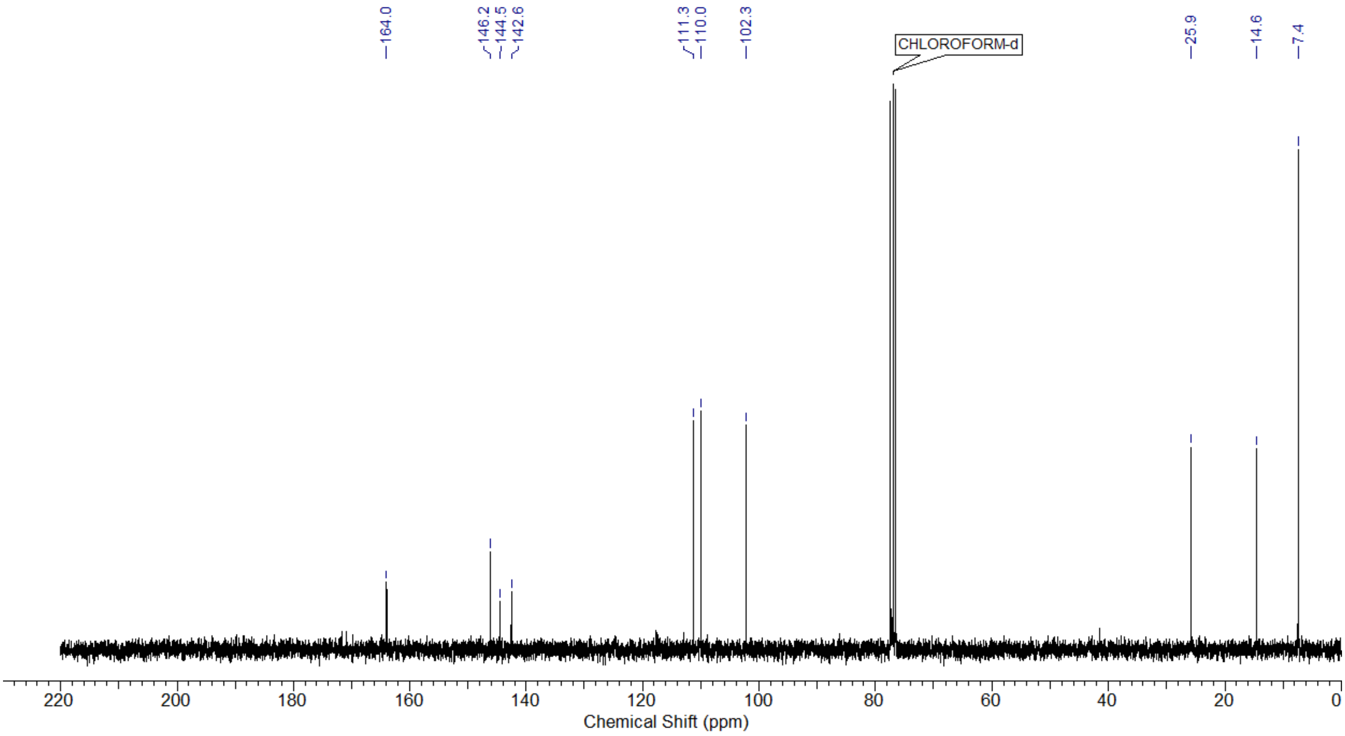

-Methoxy-N-(2,2,2-trifluoroethyl)benzenamine (**10a**) [*CAS 62158-95-6]*

^{1}H NMR (300 MHz, $CDCl_3$):

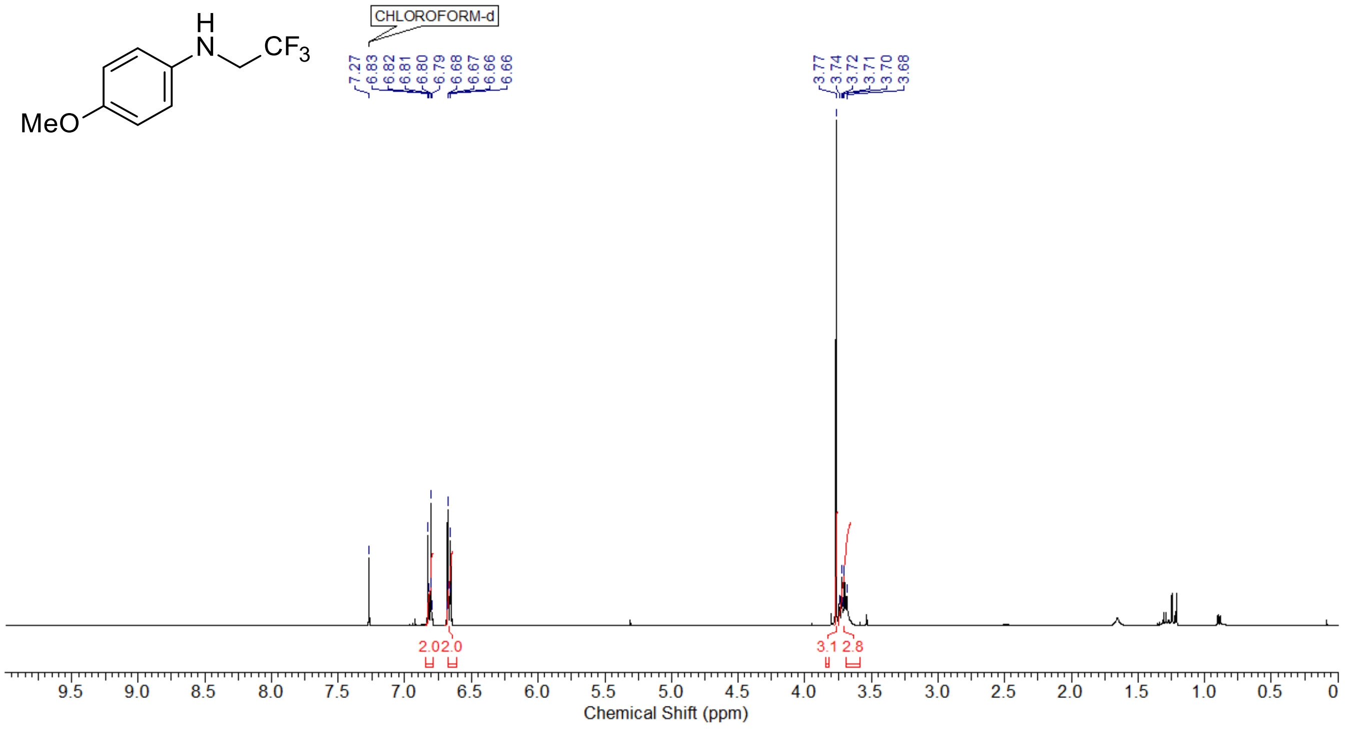

^{13}C NMR (75 MHz, $CDCl_3$):

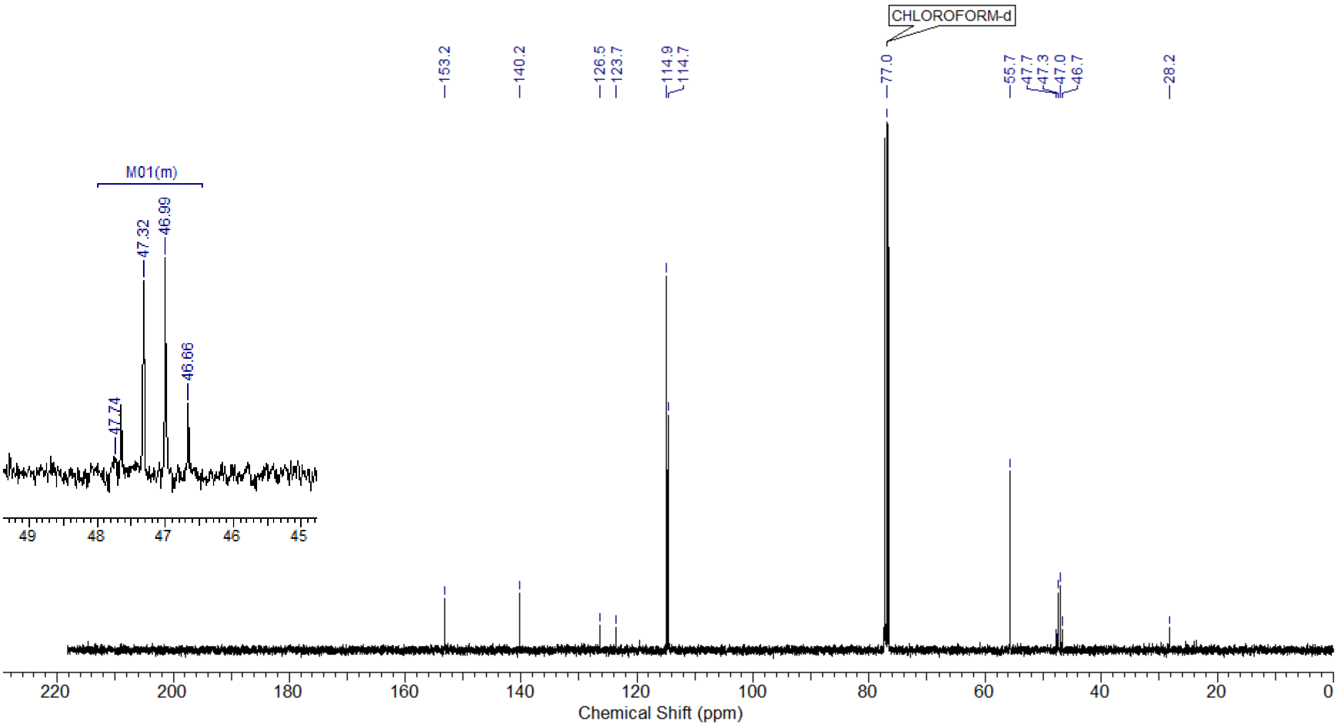

^{19}F NMR (76 MHz, $CDCl_3$, $C_6H_4F_2$):

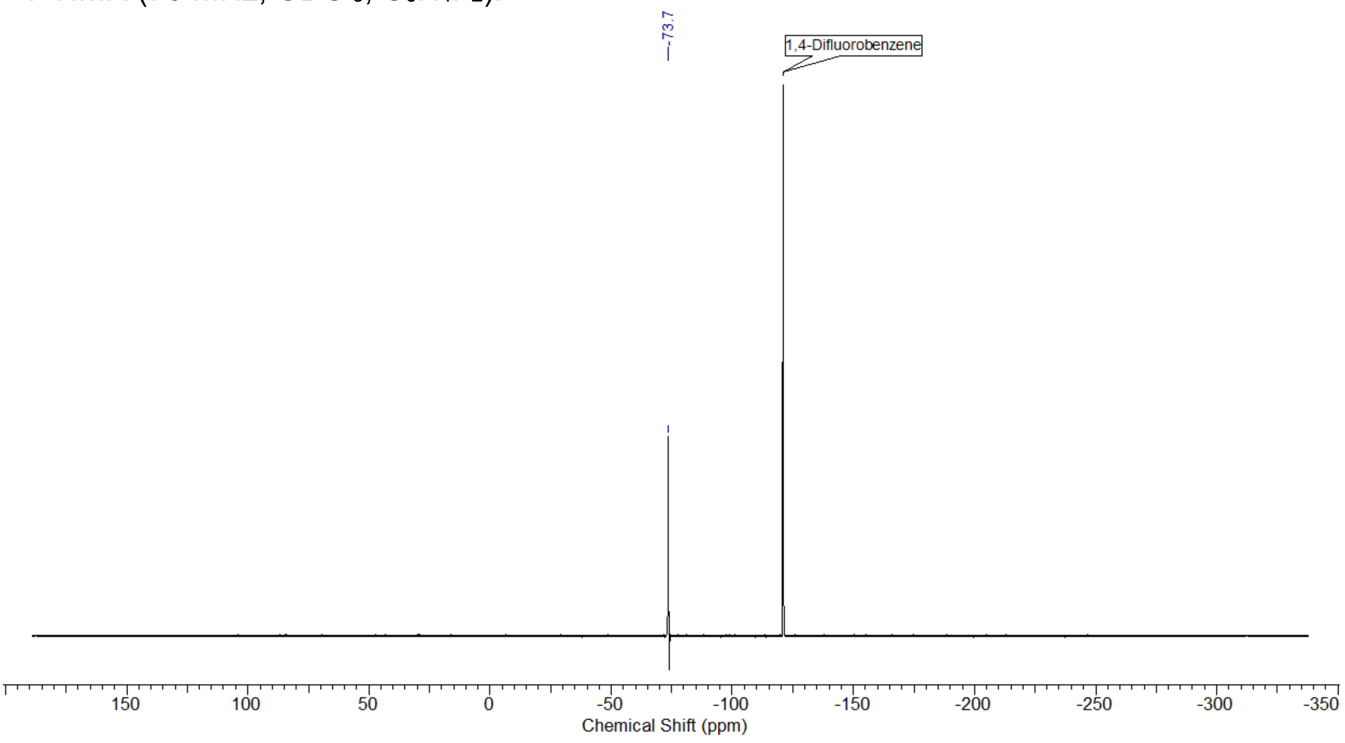

4-(4,4,5,5-tetramethyl-1,3,2-dioxaborolan-2-yl)-N-(2,2,2-trifluoroethyl)aniline (**10b**)

^{1}H NMR (300 MHz, $CDCl_3$):

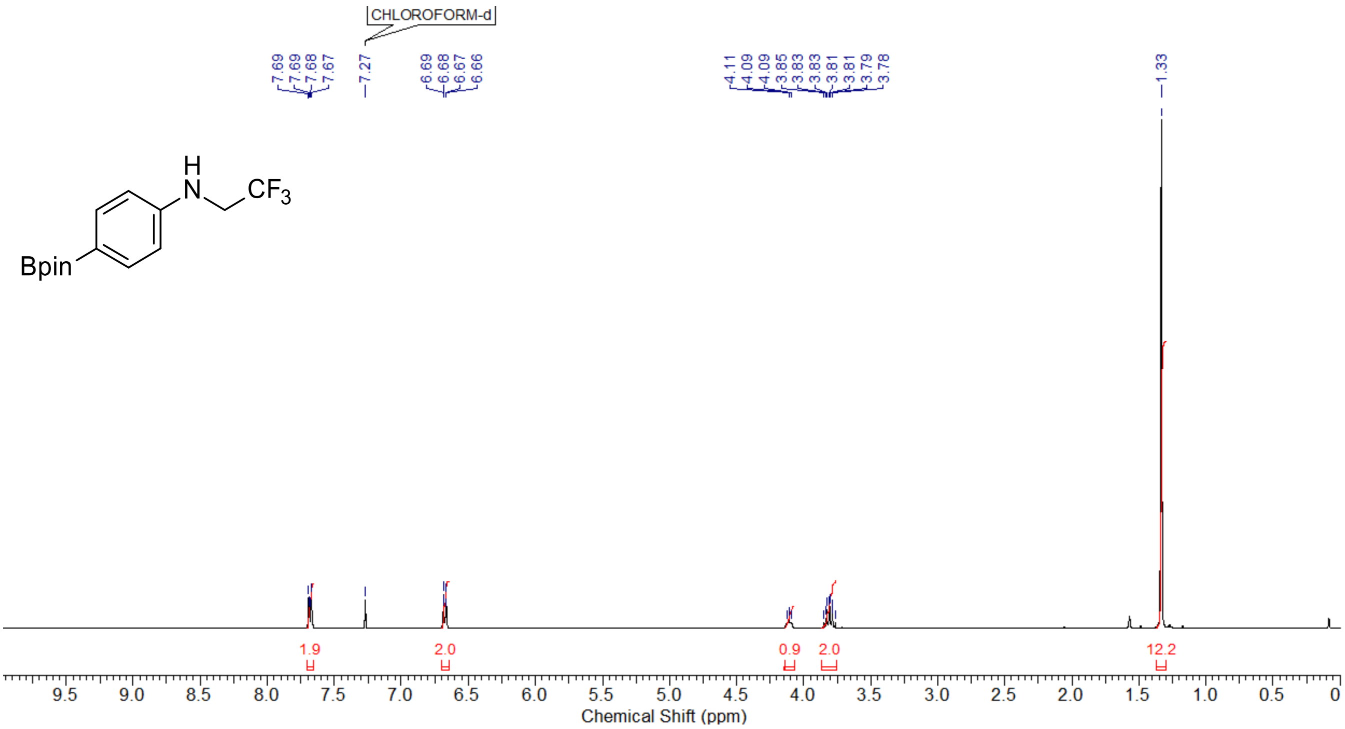

^{13}C NMR (75 MHz, $CDCl_3$):

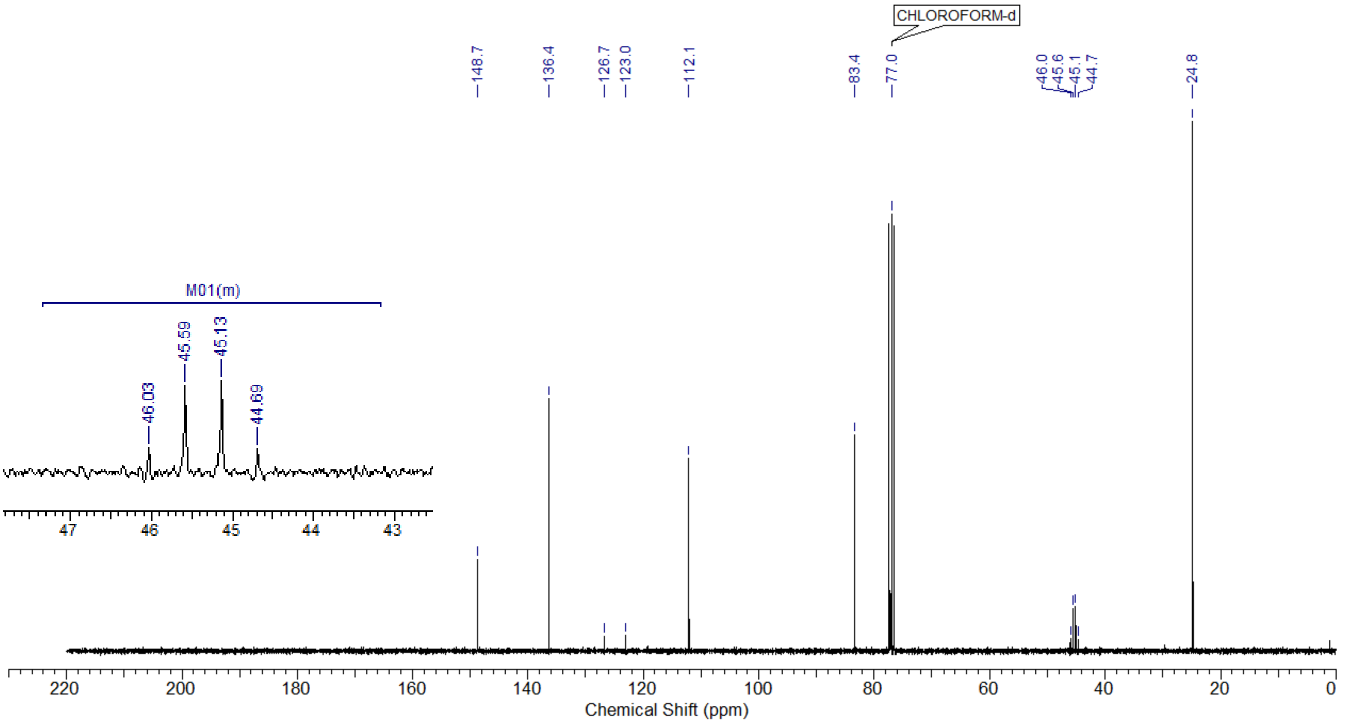

^{19}F NMR (76 MHz, $CDCl_3$, $C_6H_4F_2$):

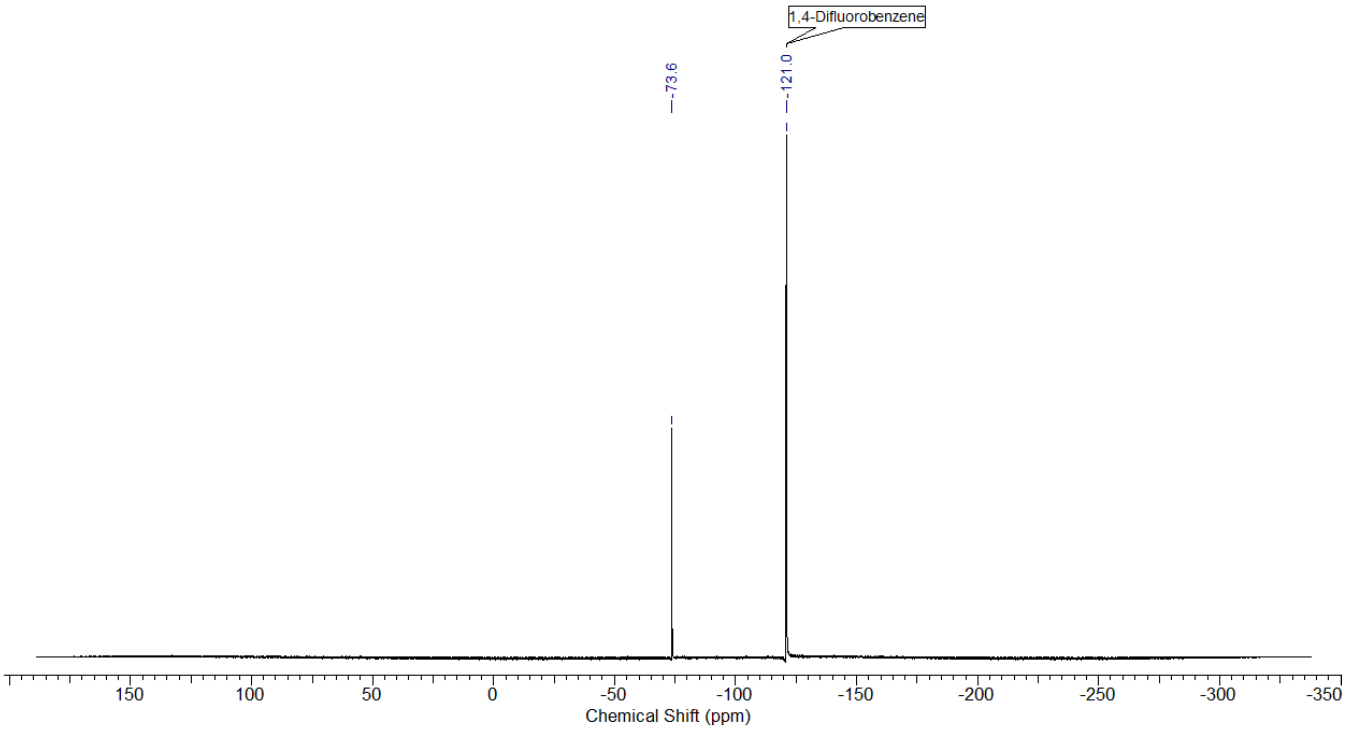

N-(2,2,2-Trifluoroethyl)-2-benzothiazolamine (**10c**) [CAS 1036568-13-4]

^{1}H NMR (400 MHz, DMSO-d$_6$):

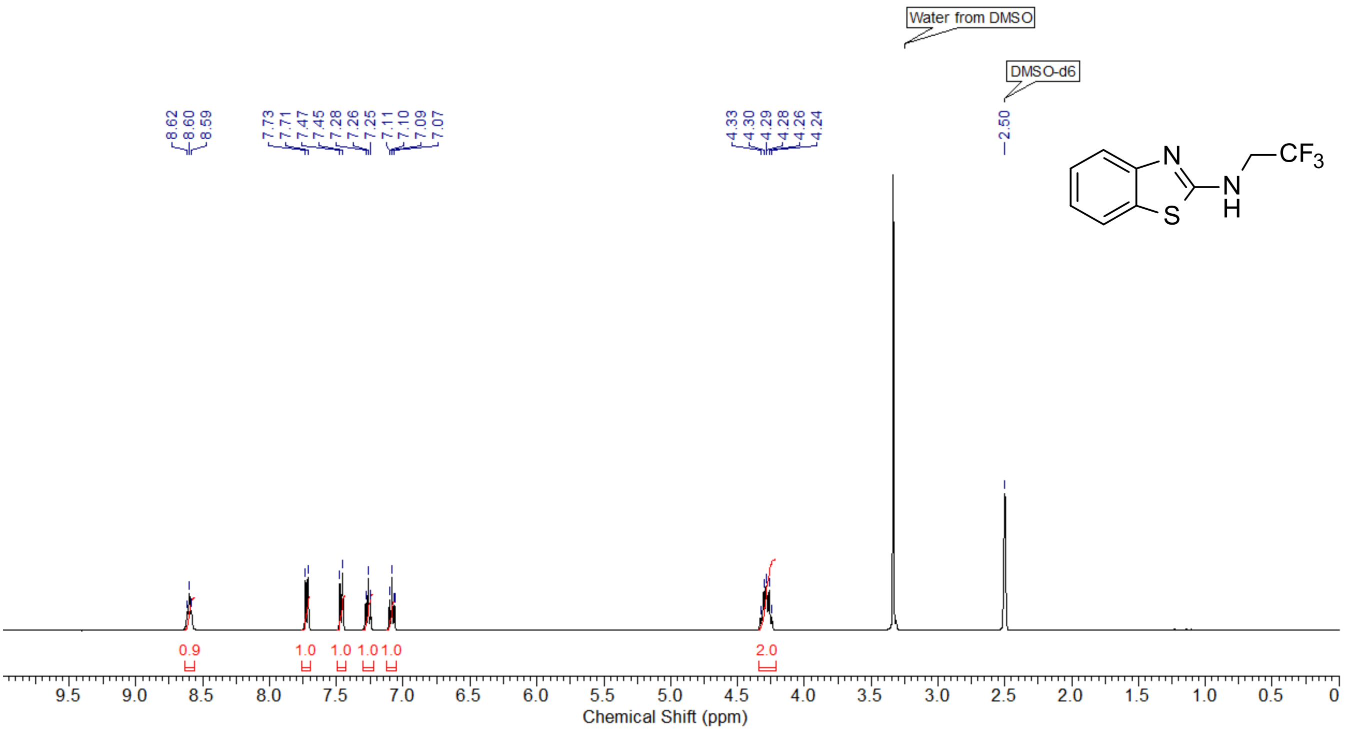

^{13}C NMR (101 MHz, DMSO-d$_6$):

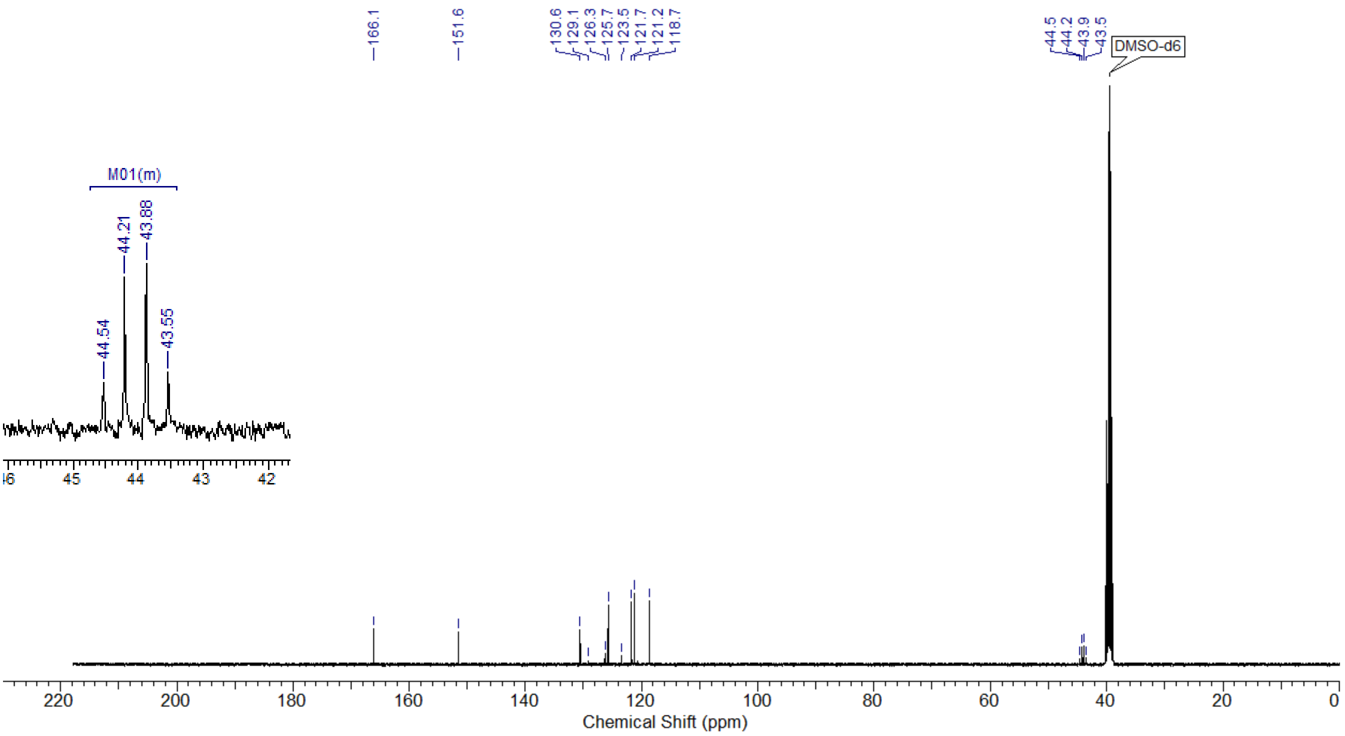

^{19}F NMR (76 MHz, DMSO-d$_6$, $C_6H_4F_2$):

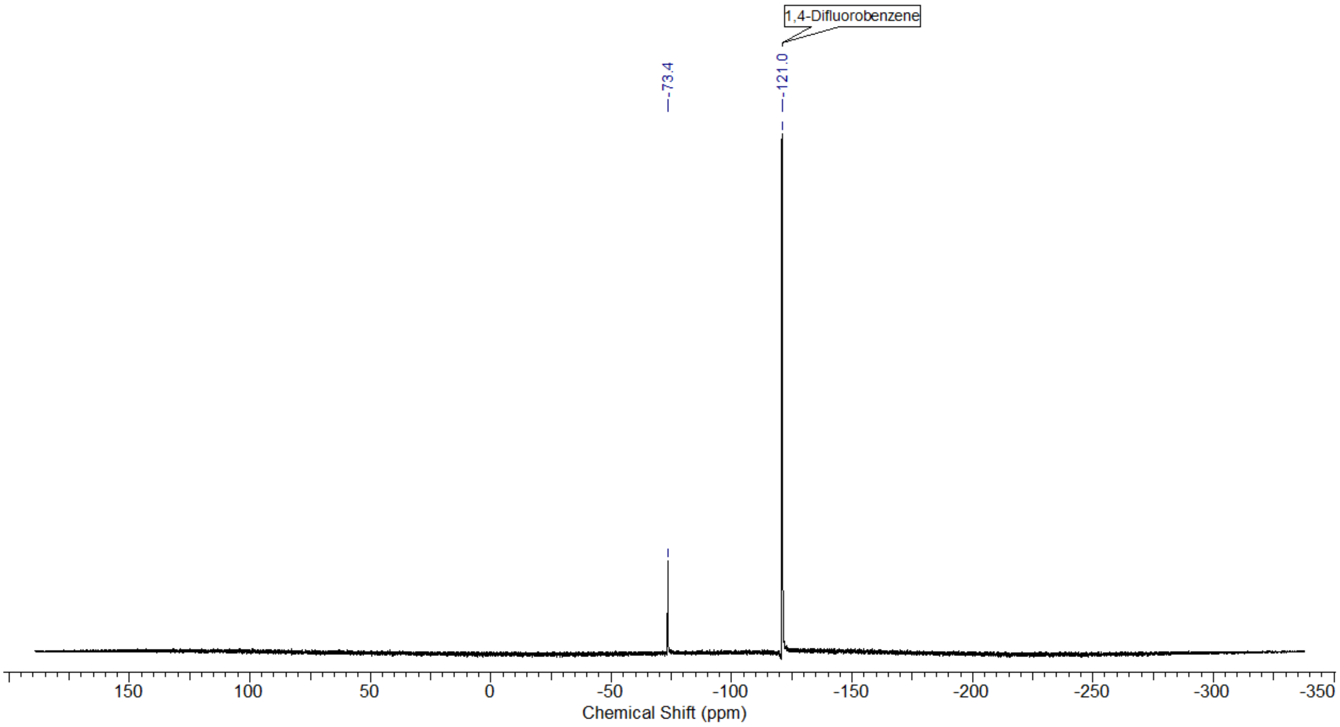

N-(4-Methoxyphenyl)-2-thiazolamine (**11a**) [CAS 63615-92-9]

^{1}H NMR (300 MHz, $CDCl_3$):

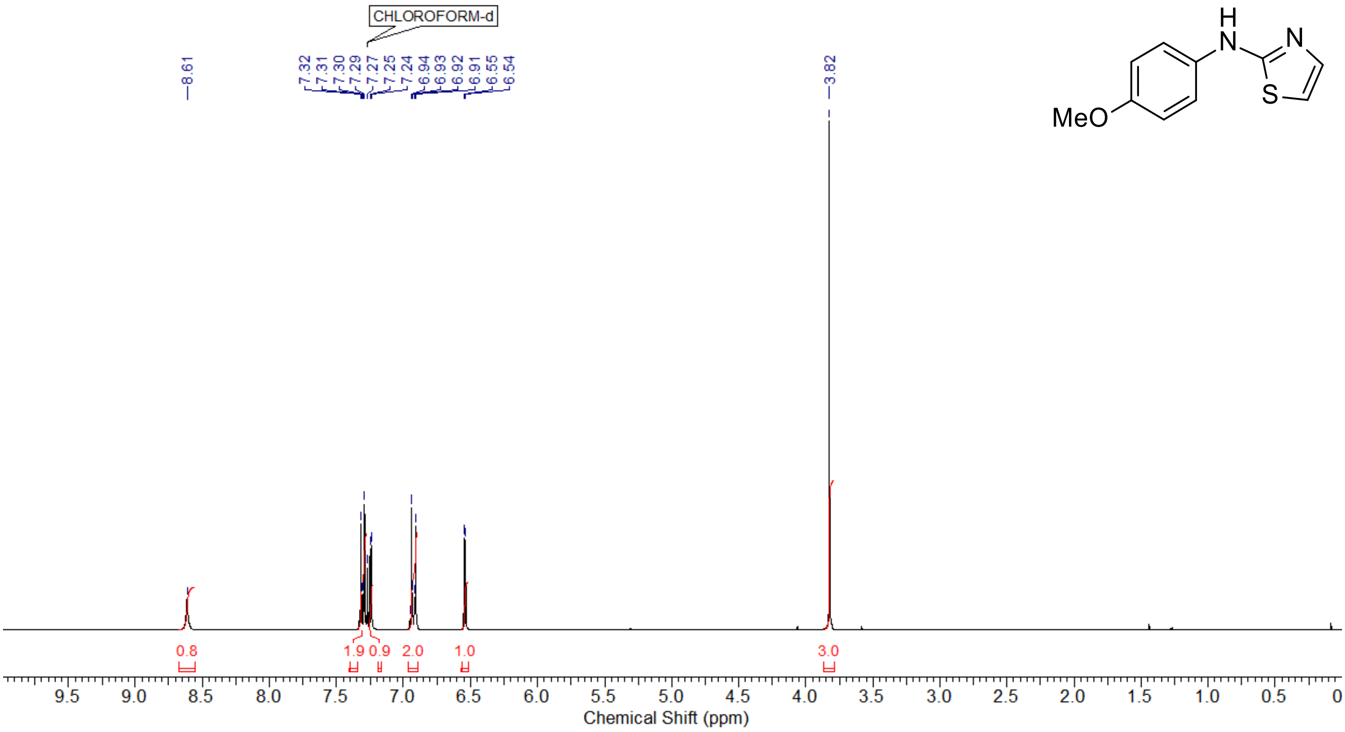

^{13}C NMR (75 MHz, $CDCl_3$):

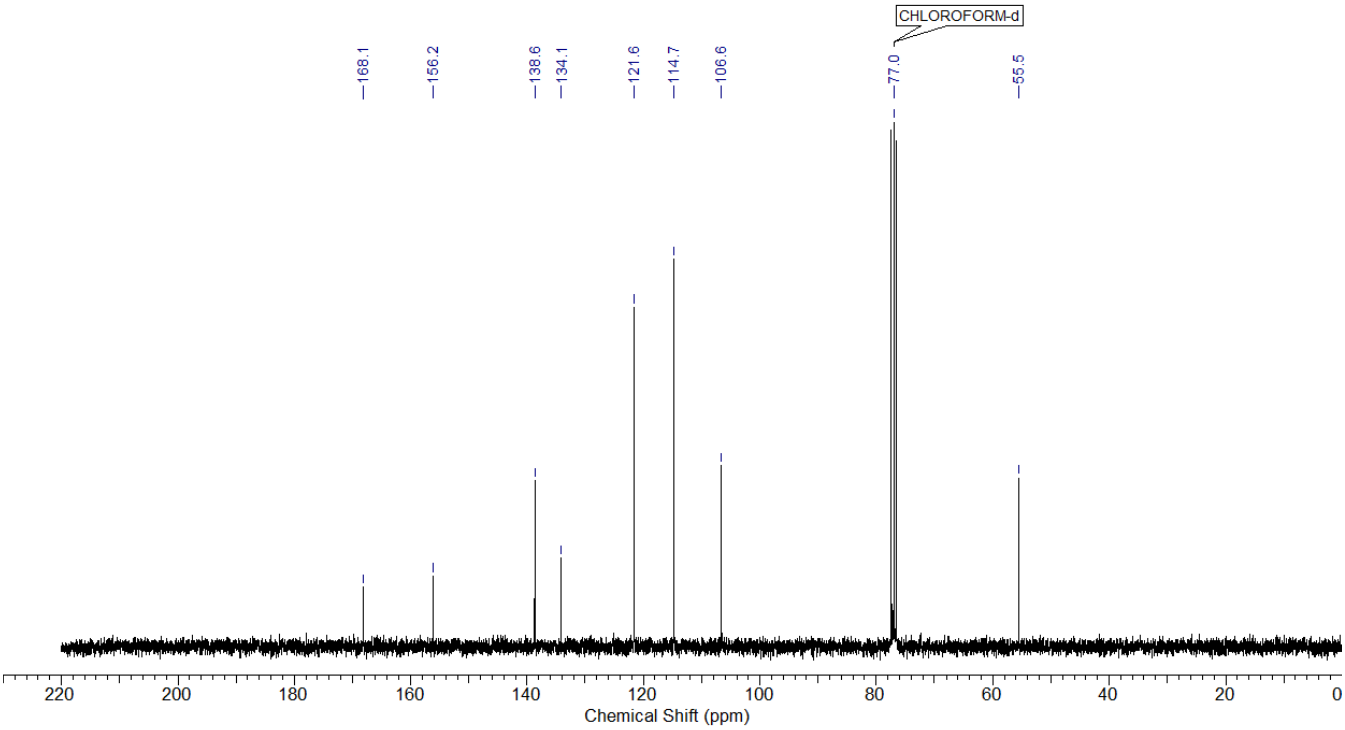

N-[4-(Trifluoromethyl)phenyl]-2-thiazolamine (**11b**) [CAS 1148107-42-9]

^{1}H NMR (300 MHz, $CDCl_3$):

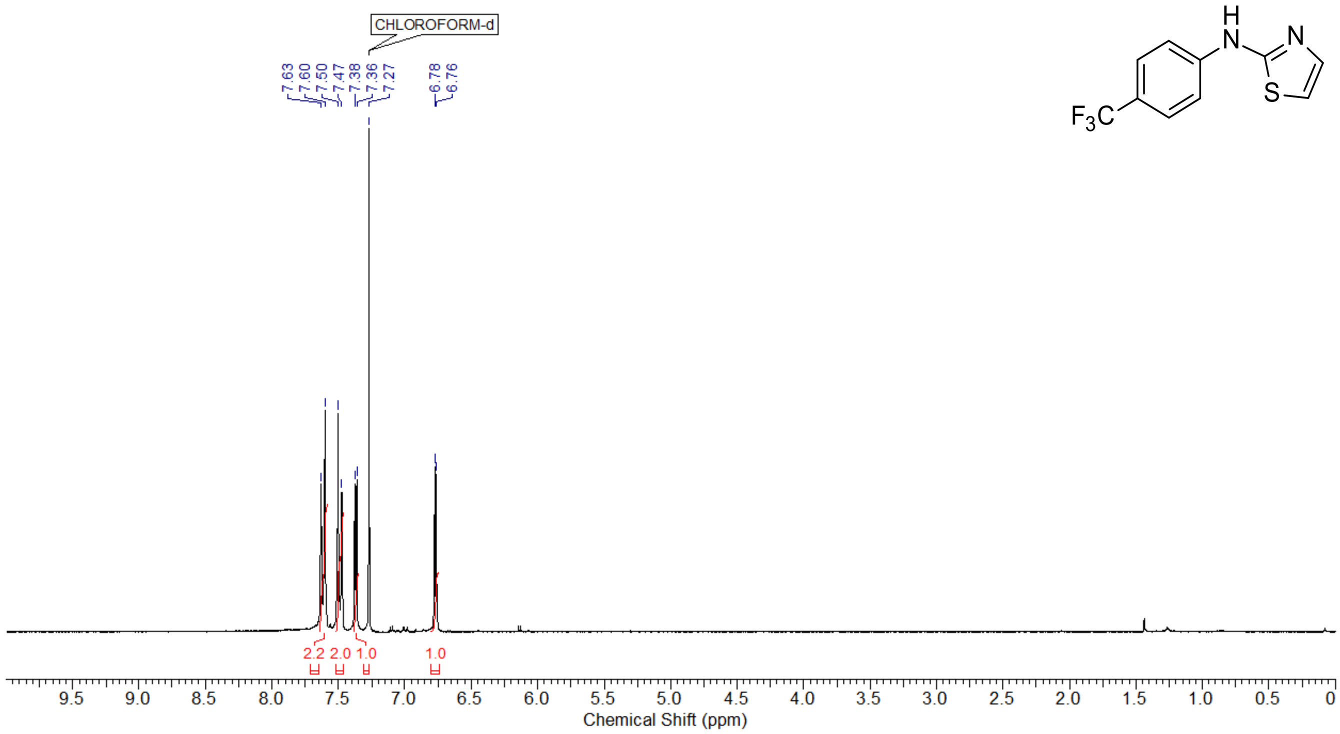

^{13}C NMR (75 MHz, $CDCl_3$):

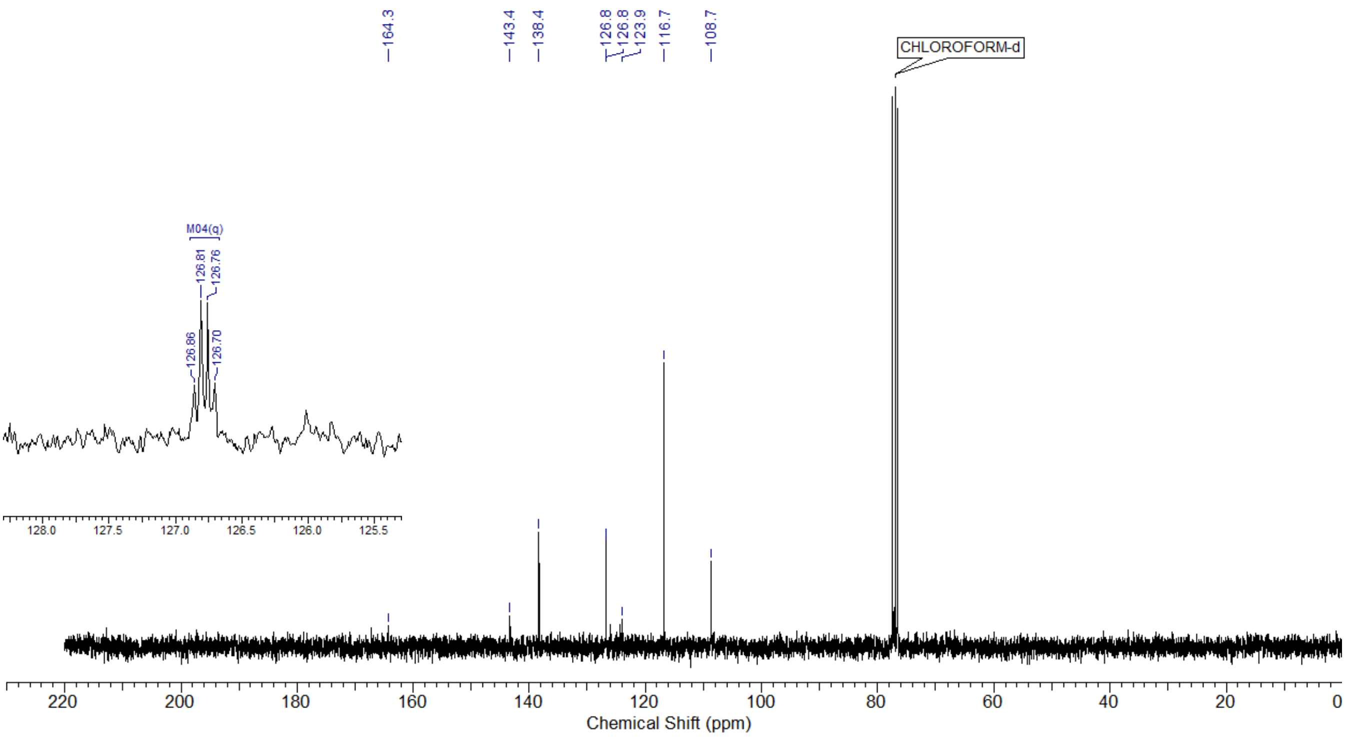

^{19}F NMR (76 MHz, $CDCl_3$, $C_6H_4F_2$):

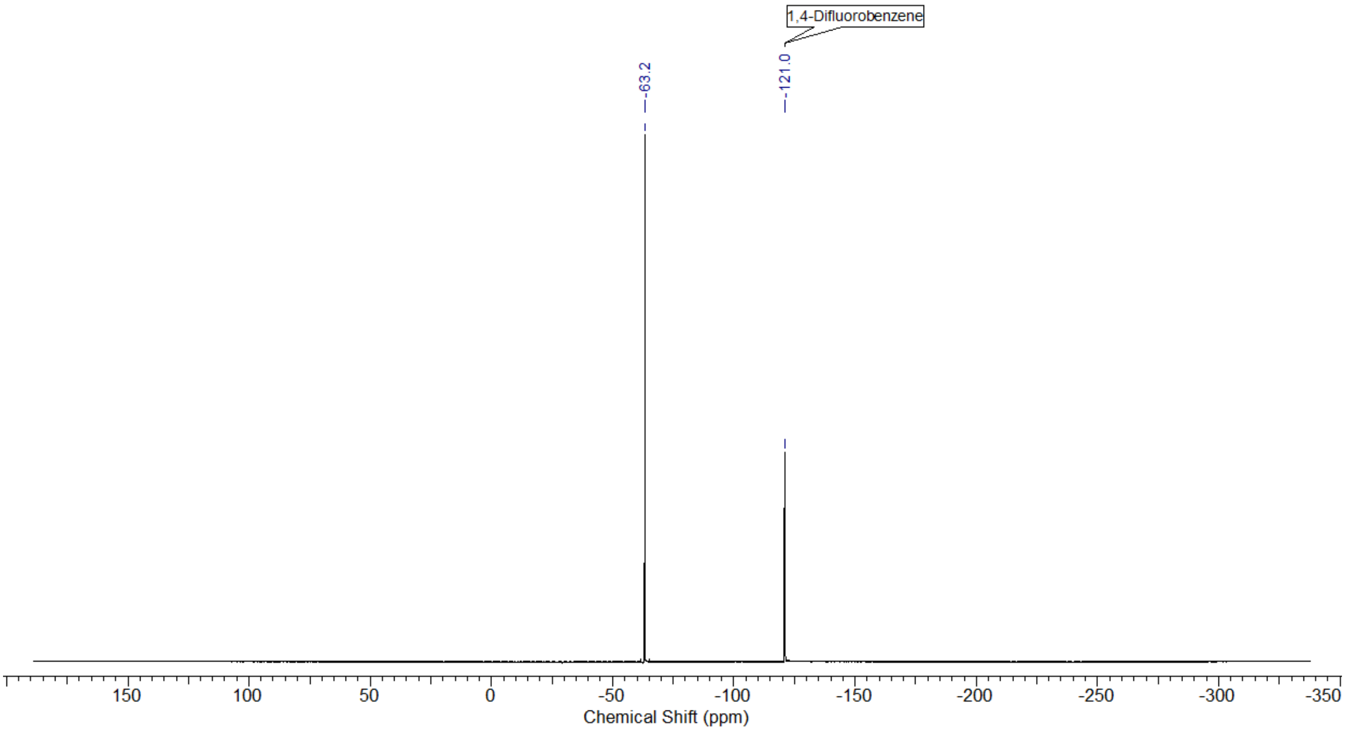

<u>*N*-(4-Methylphenyl)-2-thiazolamine</u> (**11c**) [CAS 112584-15-3]

^{1}H NMR (300 MHz, $CDCl_3$):

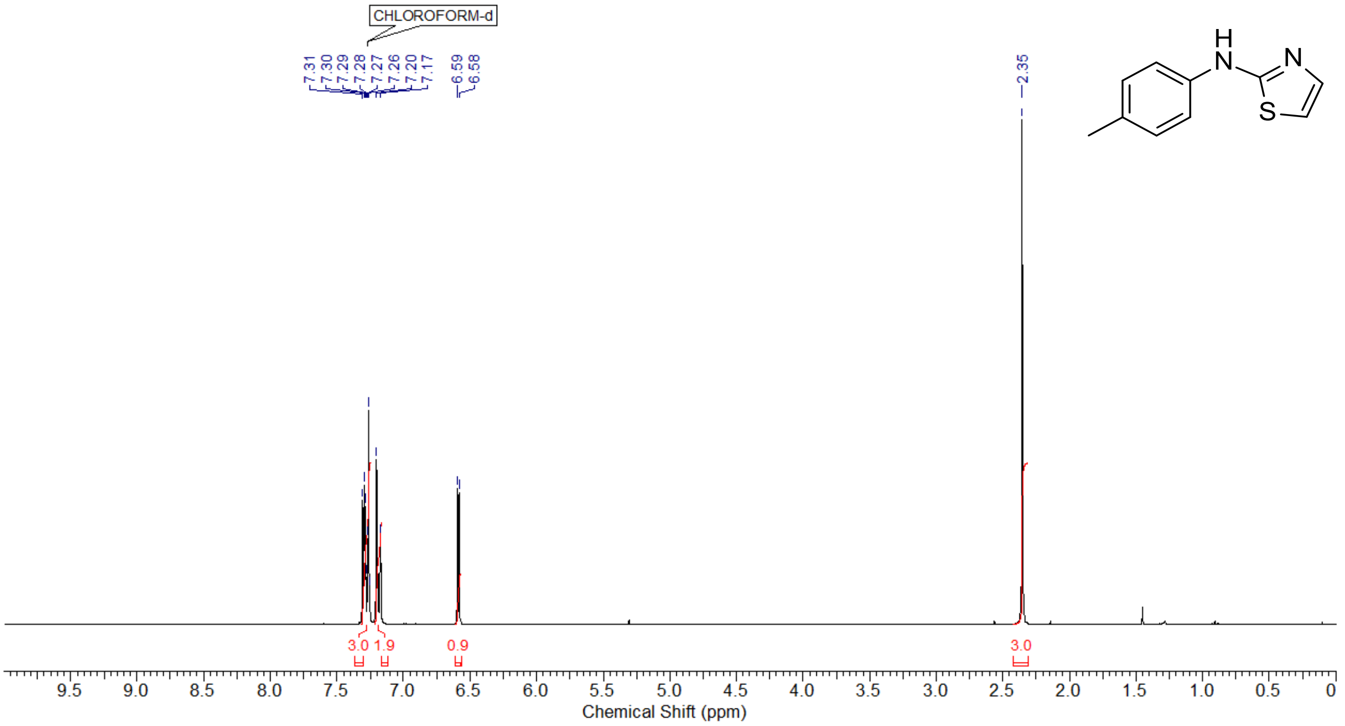

^{13}C NMR (75 MHz, $CDCl_3$):

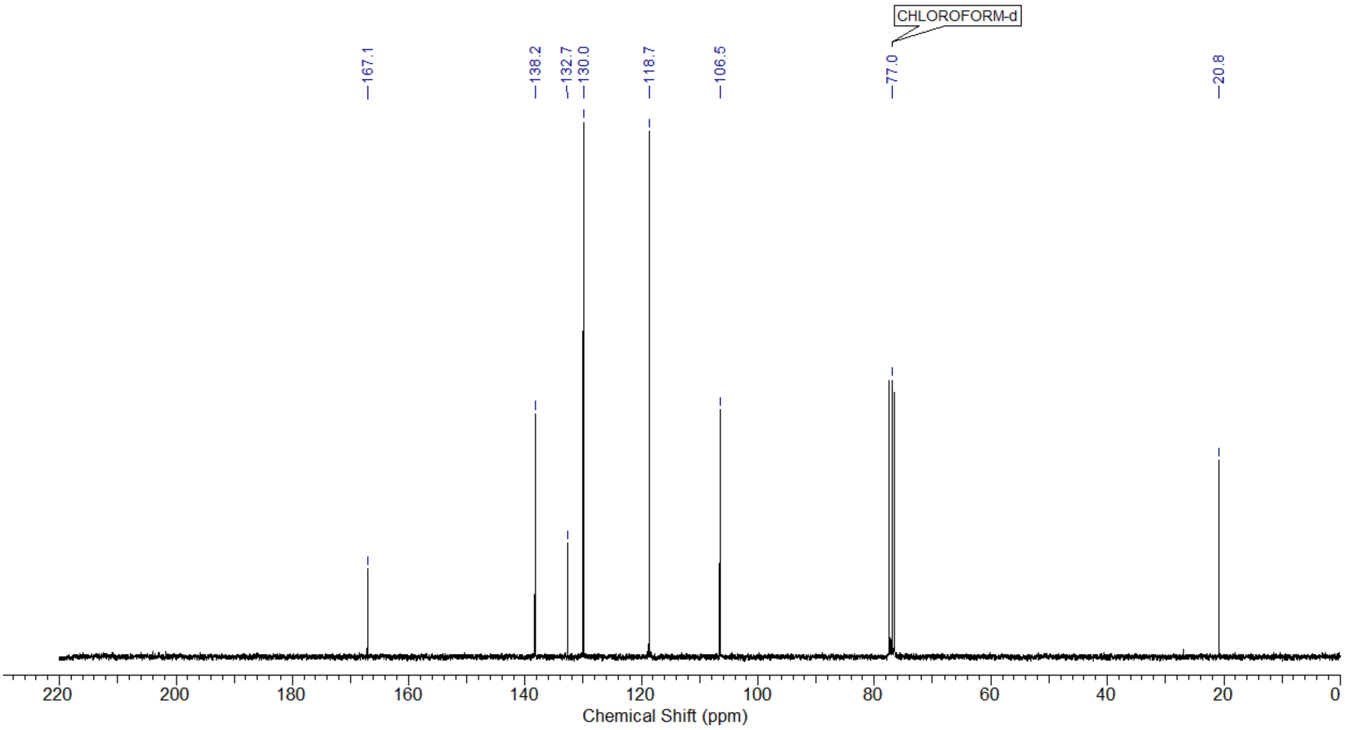

4-(2-Thiazolylamino)benzonitrile (**11d**) [CAS 959928-72-4]

^{1}H NMR (300 MHz, *DMSO-d*$_6$):

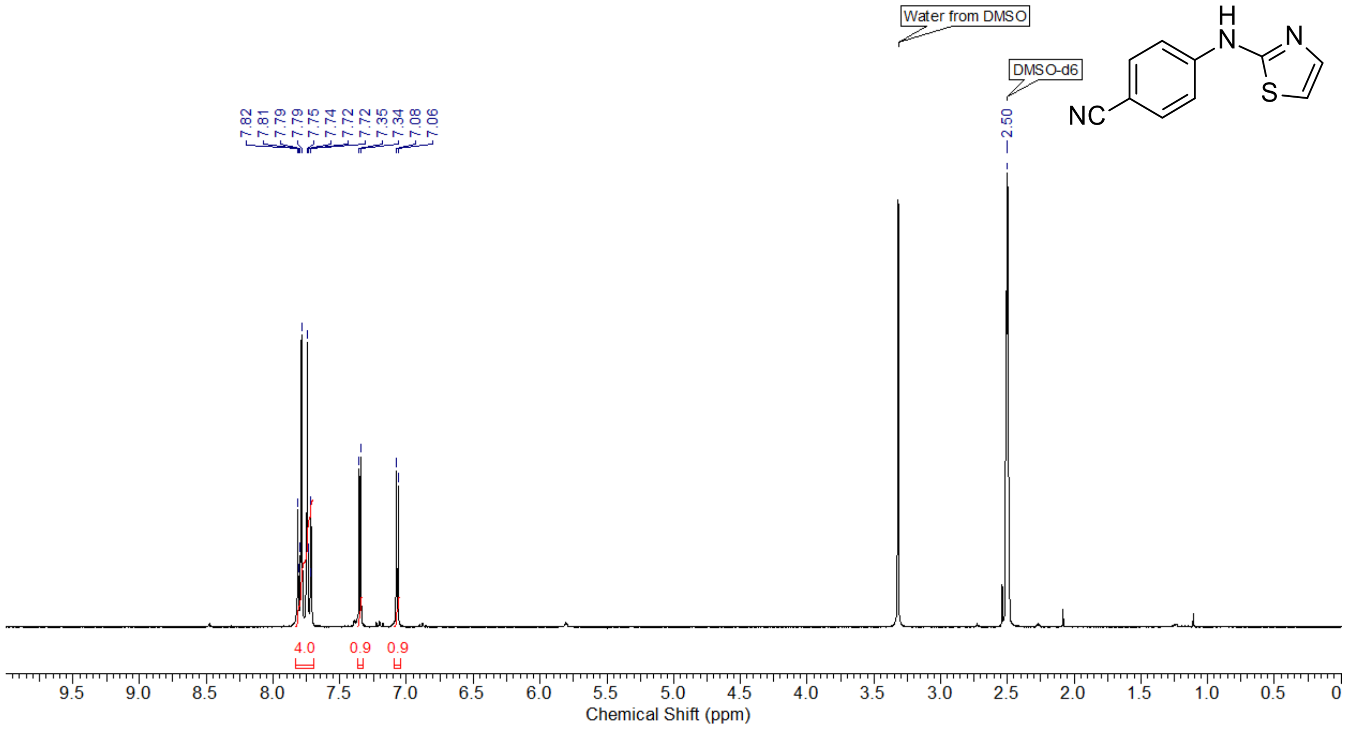

^{13}C NMR (75 MHz, *DMSO-d*$_6$):

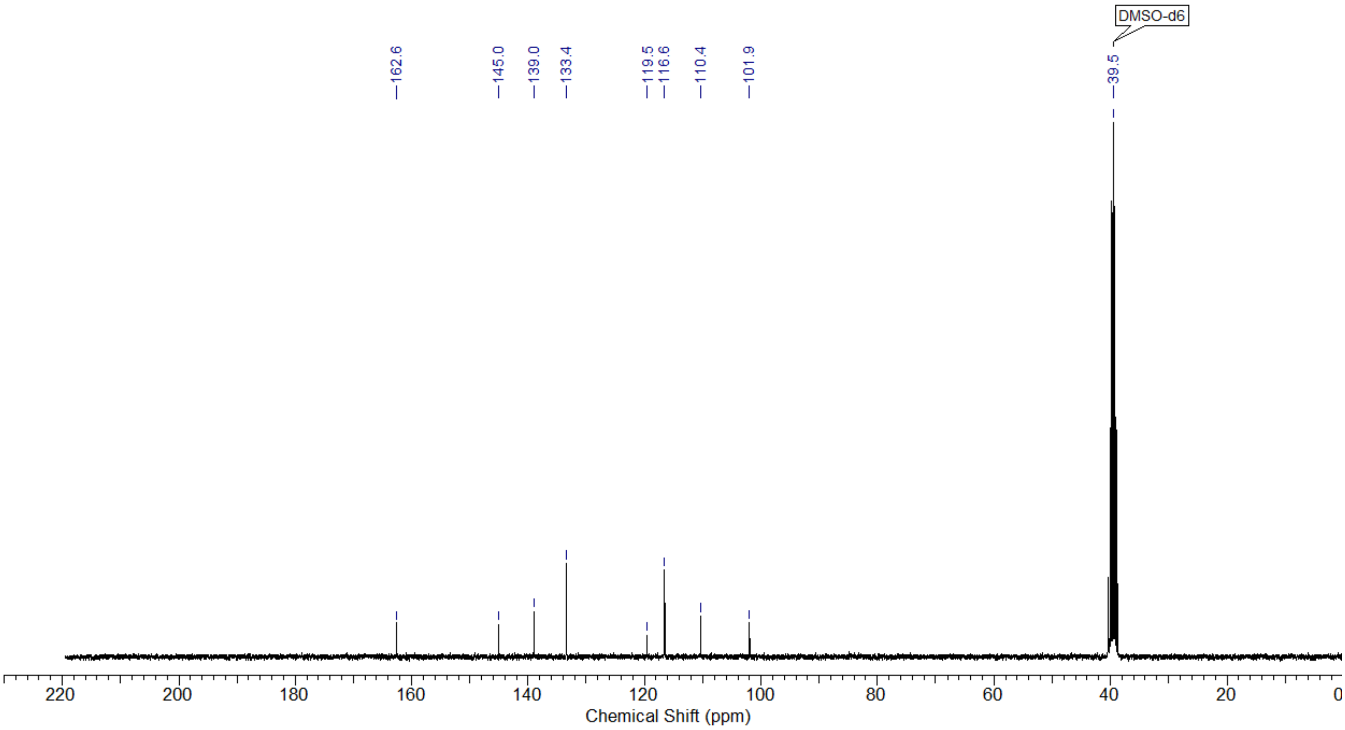

<u>*N*-2-Thiazolyl-3-pyridinamine</u> (**11e**) [CAS 58061-59-9]

^{1}H NMR (300 MHz, *DMSO-d*$_6$):

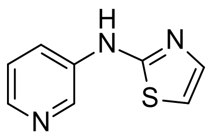

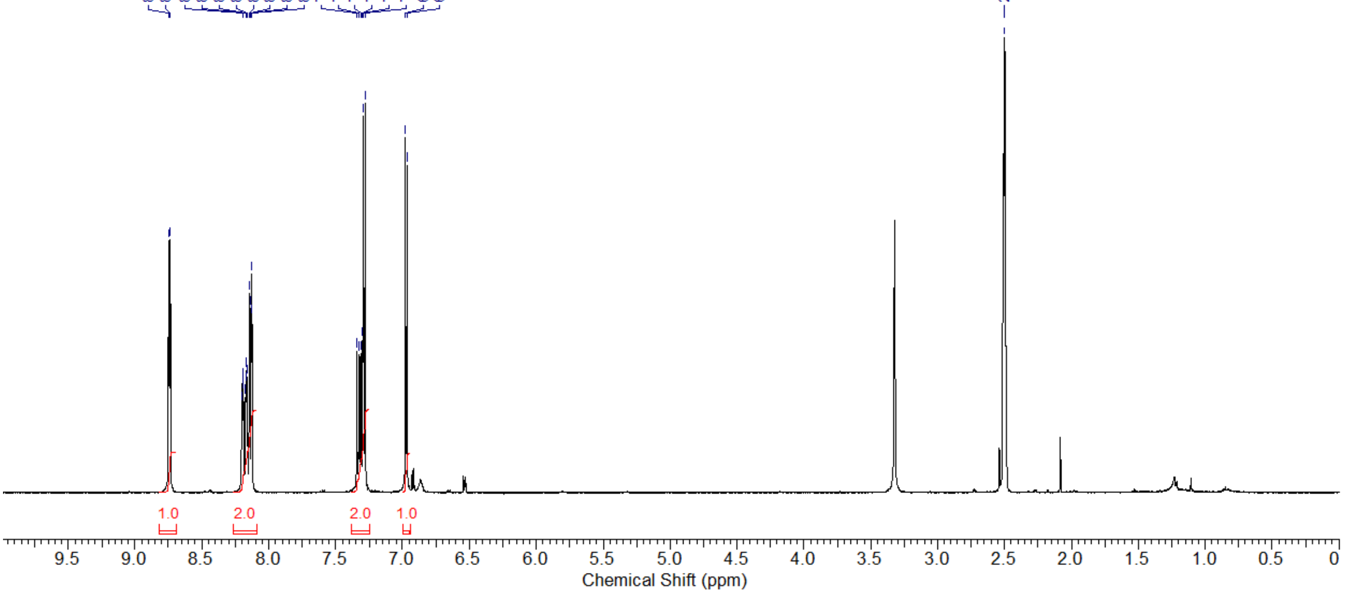

^{13}C NMR (75 MHz, *DMSO-d*$_6$):

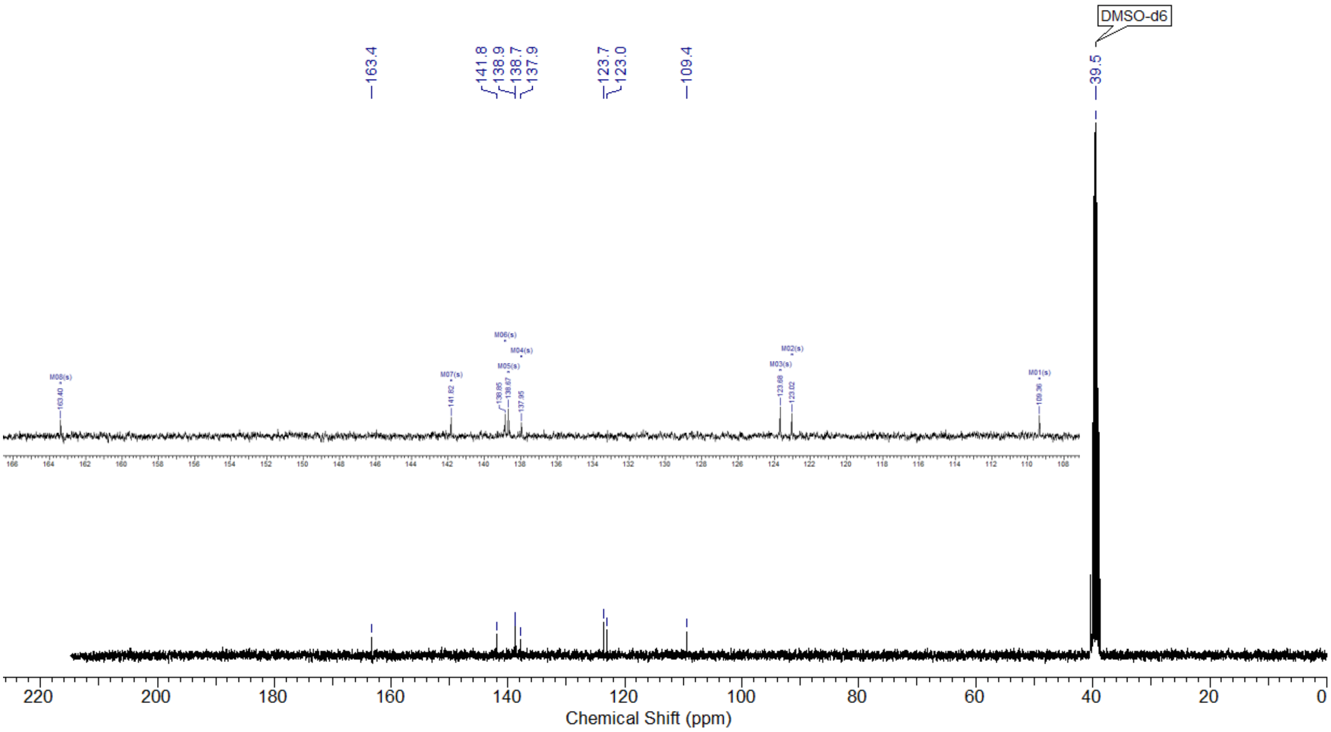

N-(4-Methoxyphenyl)-4-(trifluoromethyl)benzenamine (**13a**) [CAS 53451-88-0]

^{1}H NMR (300 MHz, $CDCl_3$):

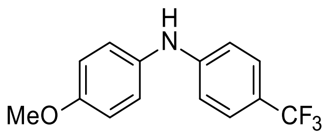

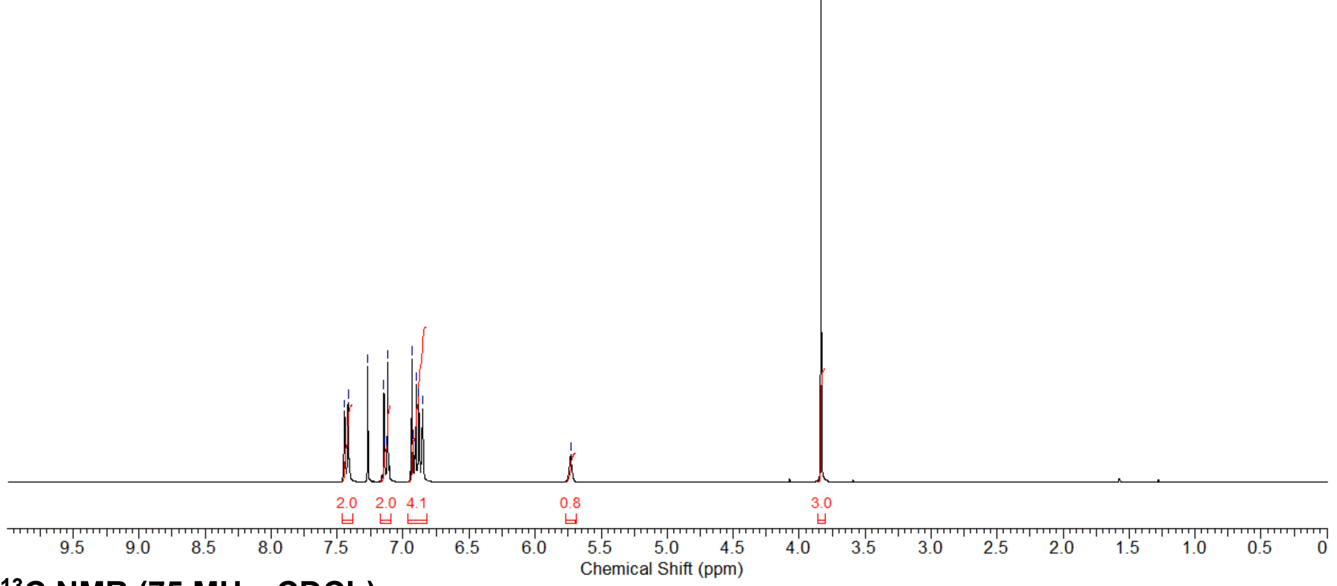

^{13}C NMR (75 MHz, $CDCl_3$):

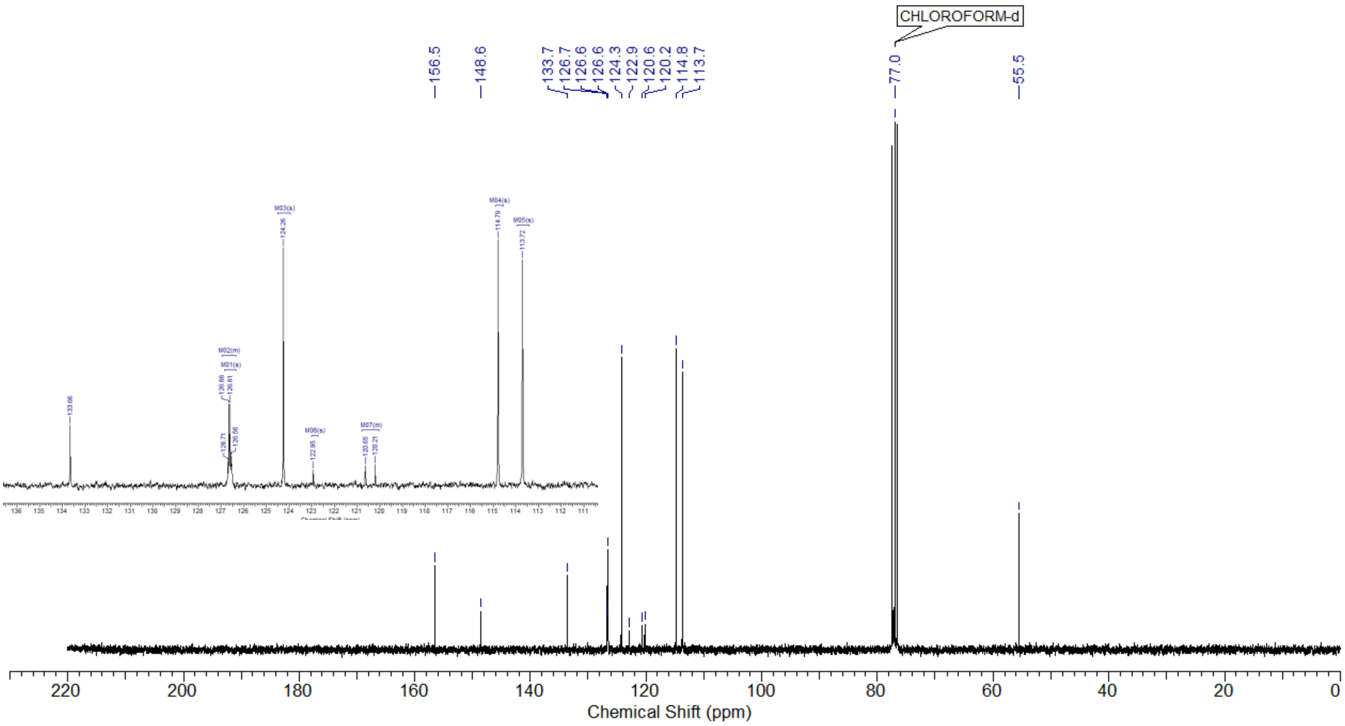

^{19}F NMR (76 MHz, $CDCl_3$, $C_6H_4F_2$):

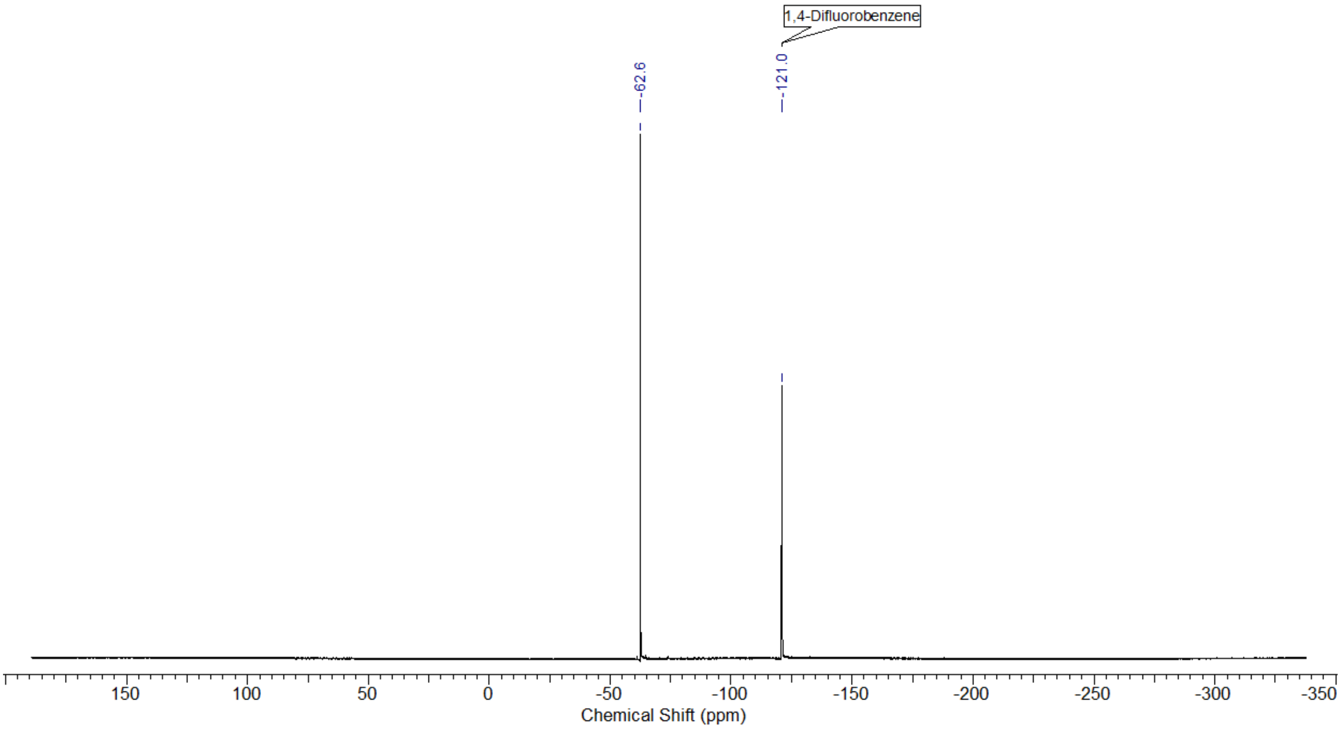

3-(1H-1,2,3-Triazol-1-yl)benzonitrile (14a) [CAS 85862-70-0]

^{1}H NMR (400 MHz, $CDCl_3$):

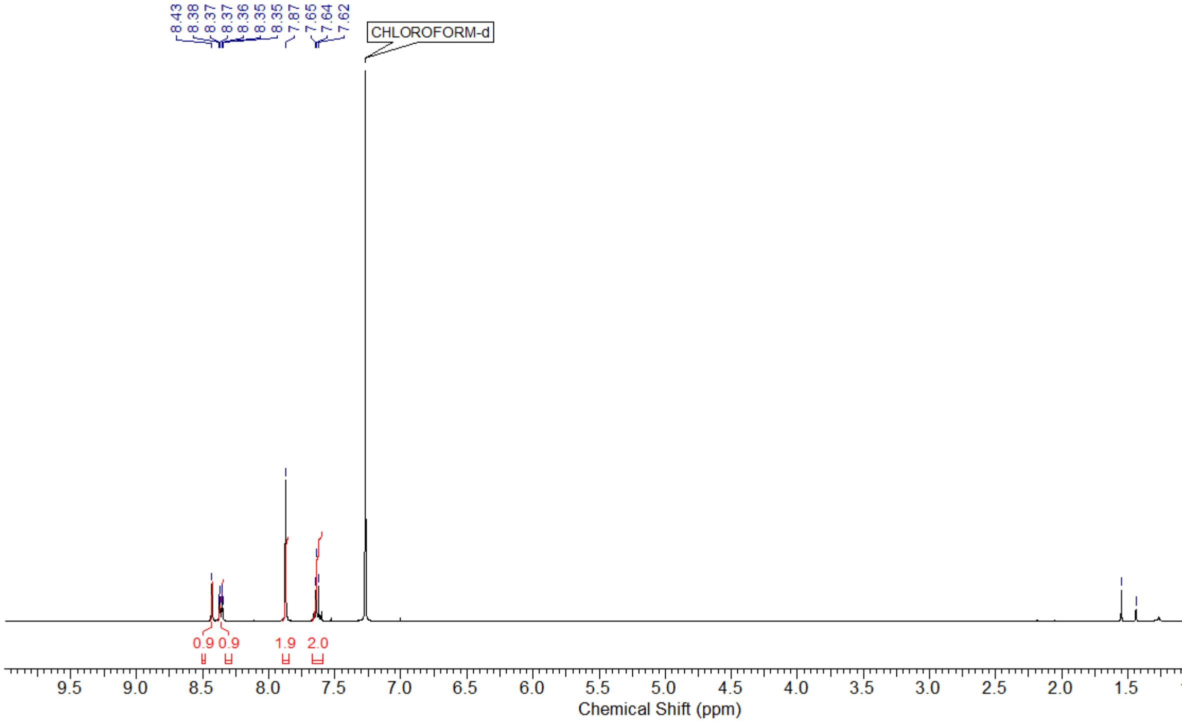

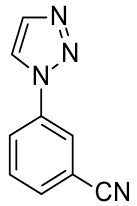

^{13}C NMR (101 MHz, $CDCl_3$):

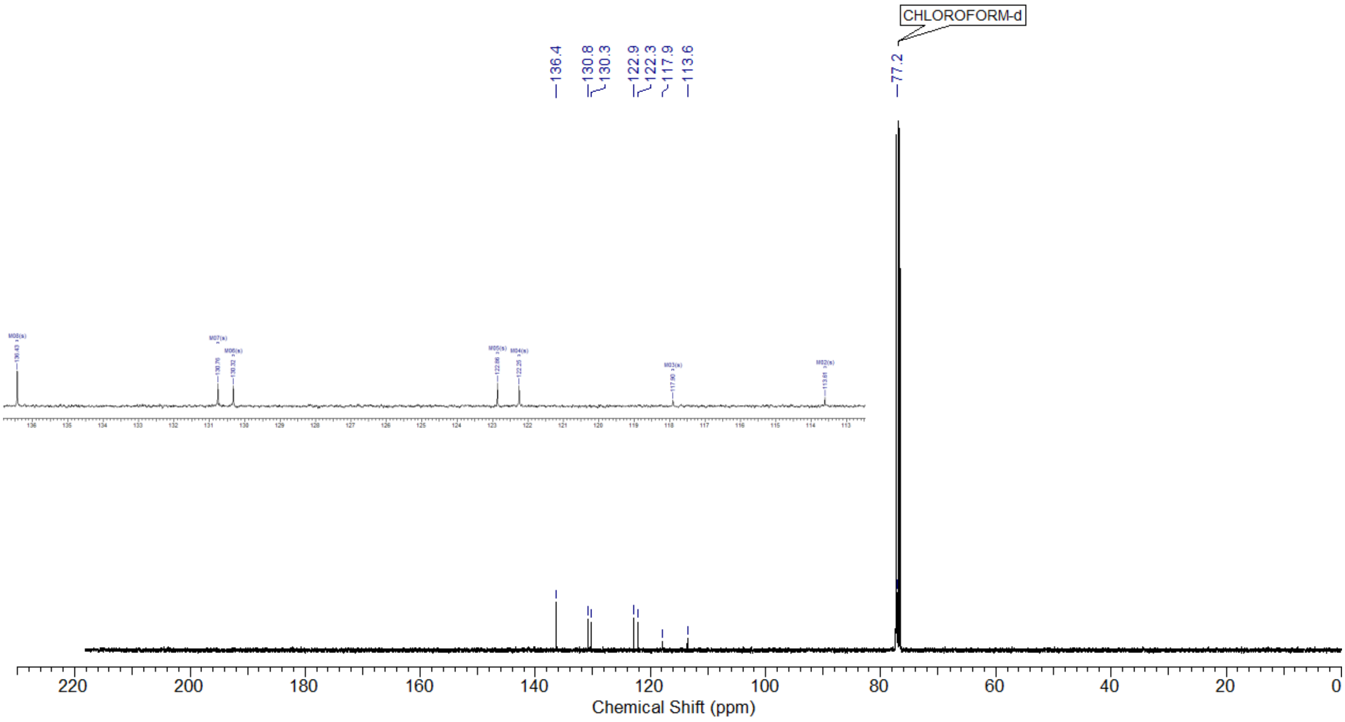

1-(2-Fluorophenyl)-4-methyl-1H-imidazole (**15a**) [CAS 1351990-67-4]

^{1}H NMR (400 MHz, $CDCl_3$):

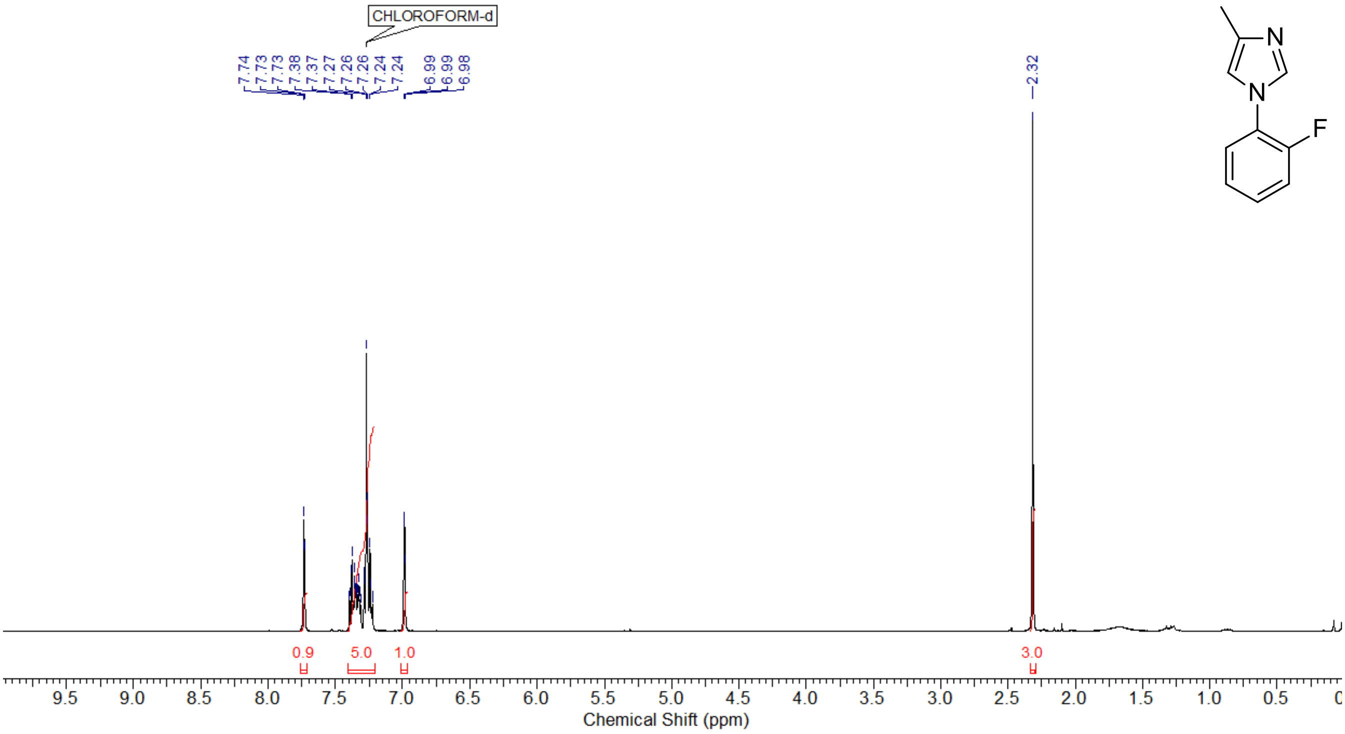

^{13}C NMR (101 MHz, $CDCl_3$):

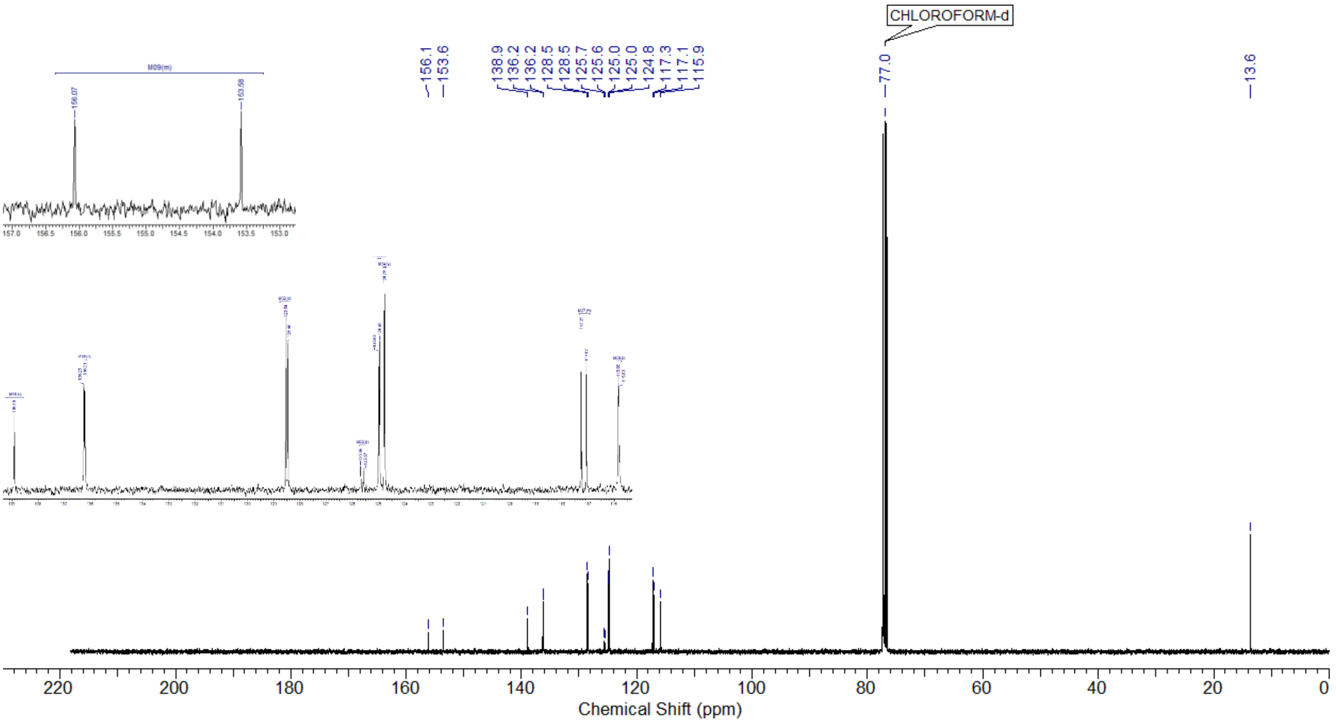

^{19}F NMR (76 MHz, $CDCl_3$, $C_6H_4F_2$):

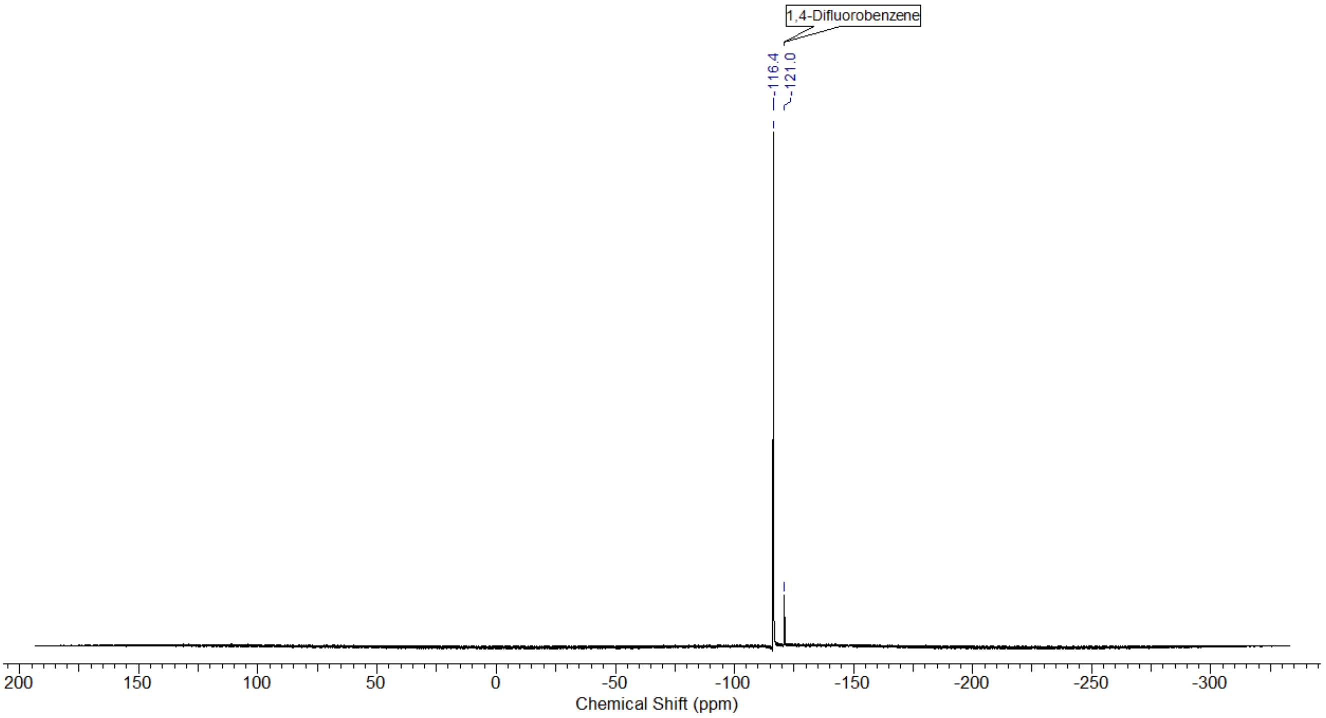

Anisonitrile (1**6a**) [CAS 874-90-8]

^{1}H NMR (300 MHz, $CDCl_3$):

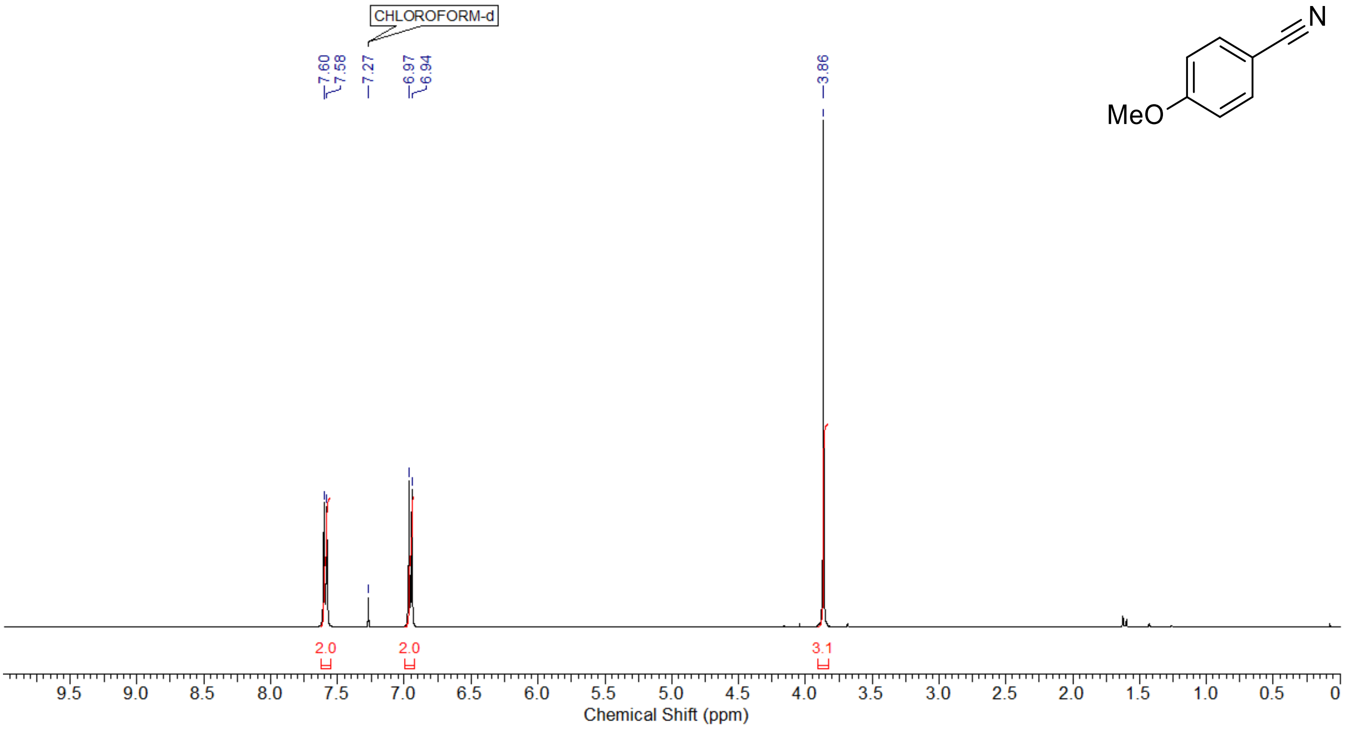

^{13}C NMR (75 MHz, $CDCl_3$):

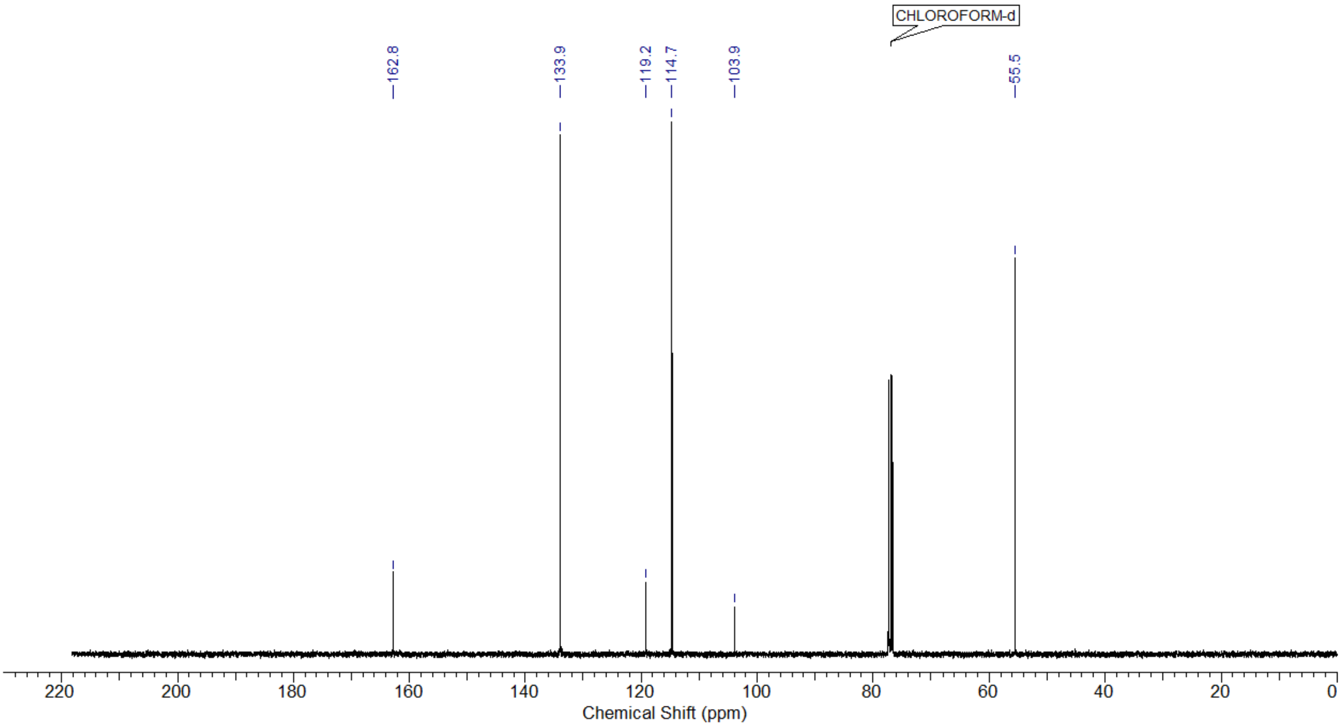

4-(Trifluoromethyl)benzonitrile (**16b**) [CAS 455-18-5]

^{1}H NMR (300 MHz, $CDCl_3$):

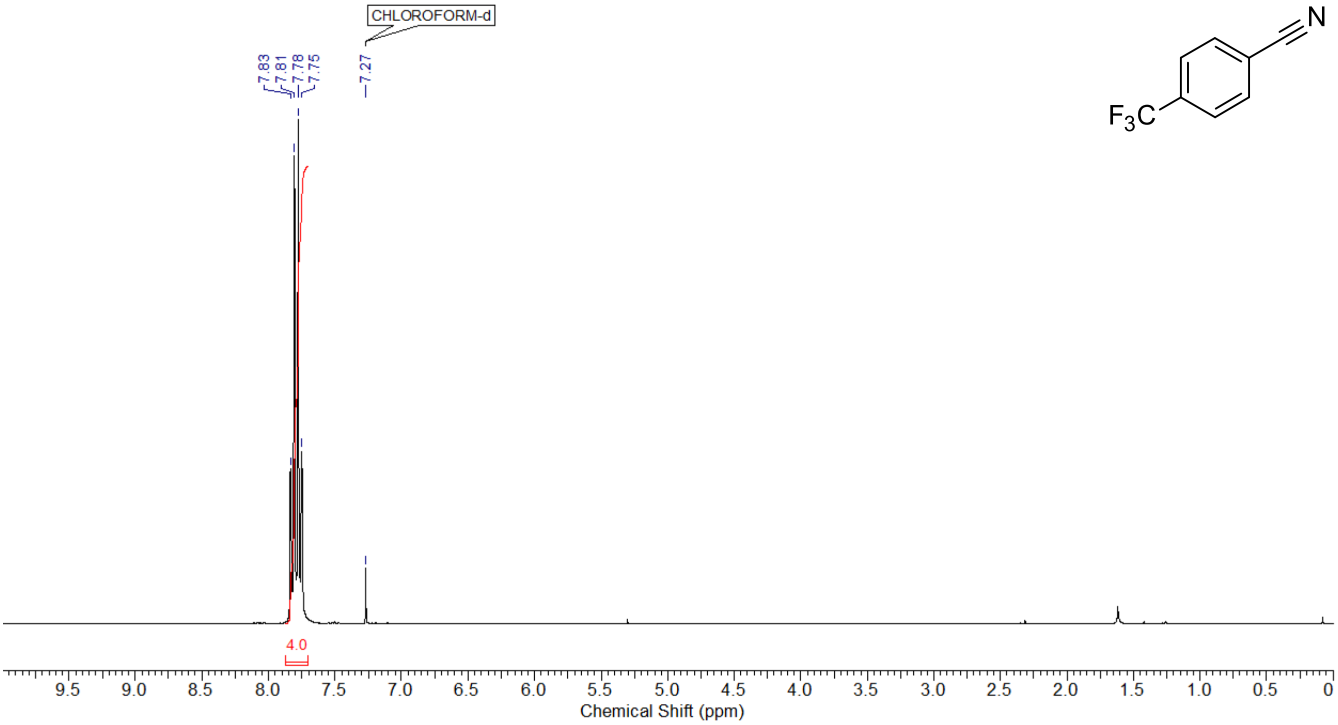

^{13}C NMR (75 MHz, $CDCl_3$):

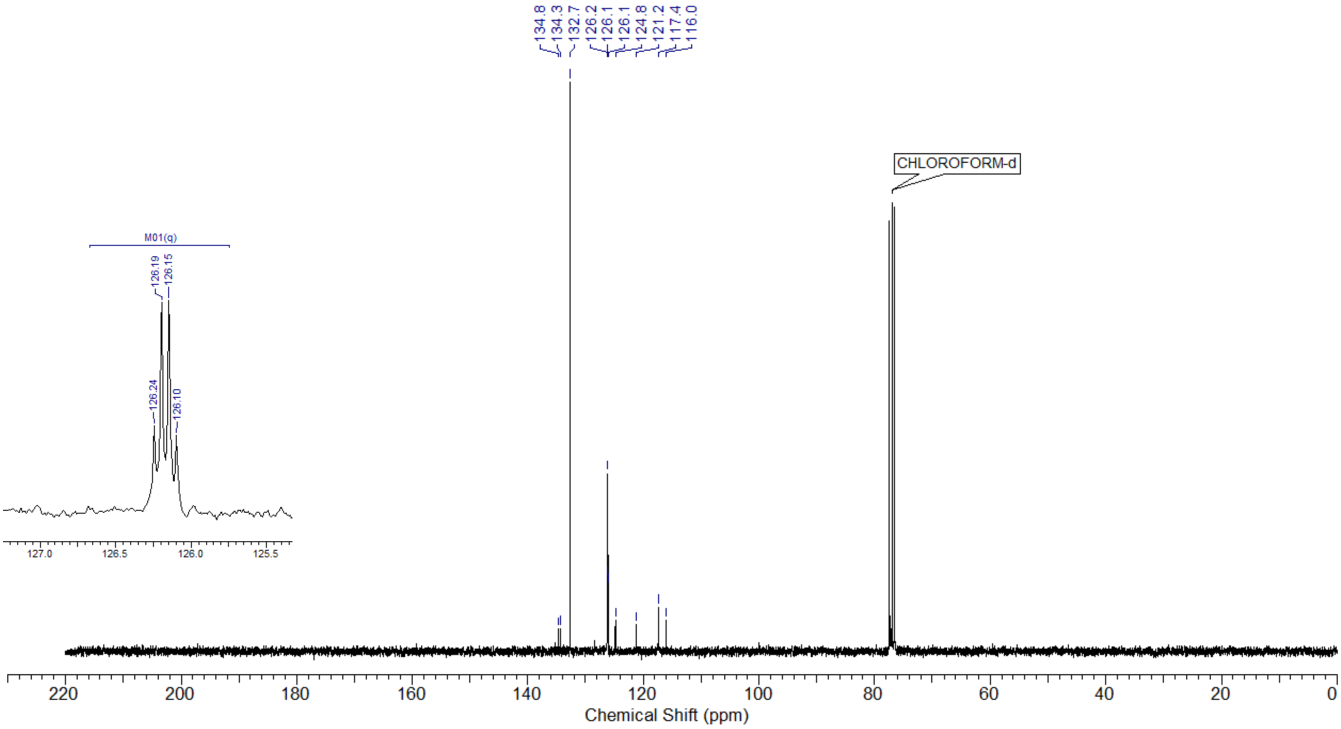

^{19}F NMR (76 MHz, $CDCl_3$, $C_6H_4F_2$):

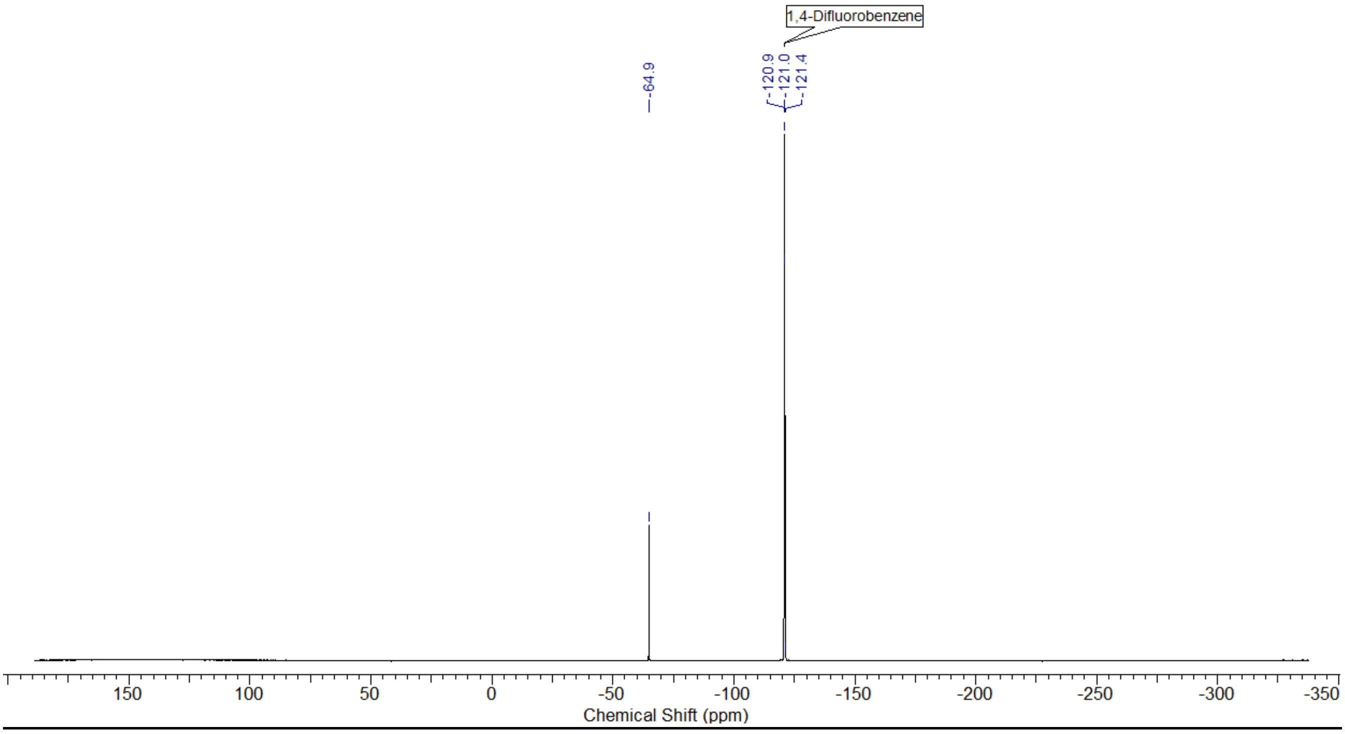

Ethyl 4-cyanobenzoate (**16d**) [CAS 7153-22-2]

^{1}H NMR (300 MHz, $CDCl_3$):

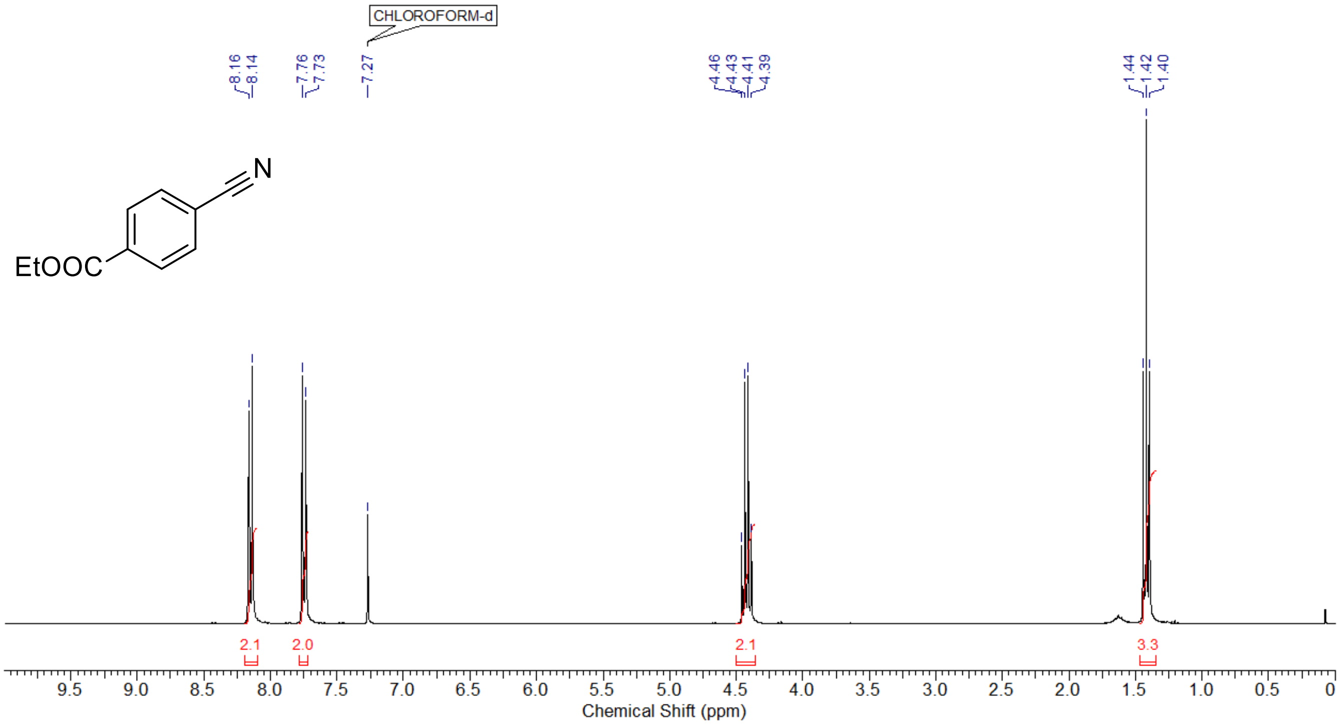

^{13}C NMR (75 MHz, $CDCl_3$):

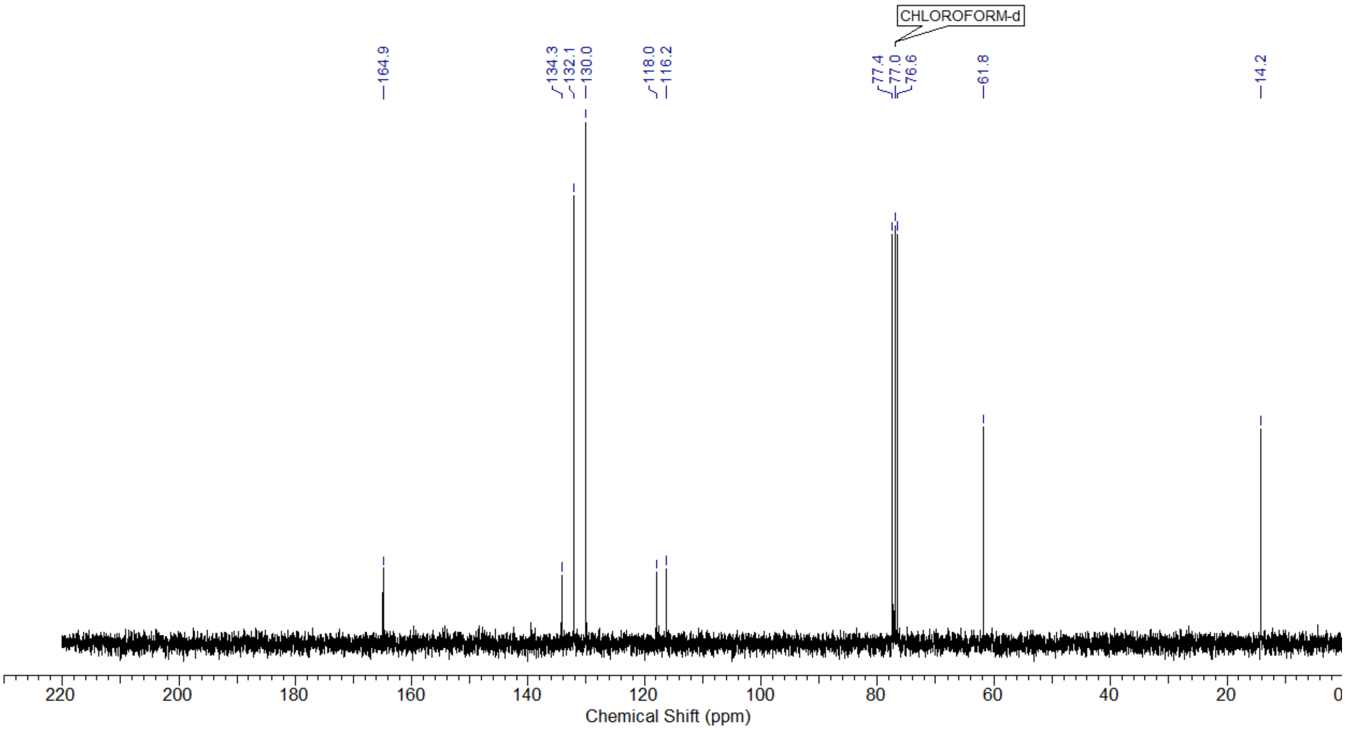

<u>p-Tolunitrile</u> (**16d**) [CAS 104-85-8]

^{1}H NMR (300 MHz, $CDCl_3$):

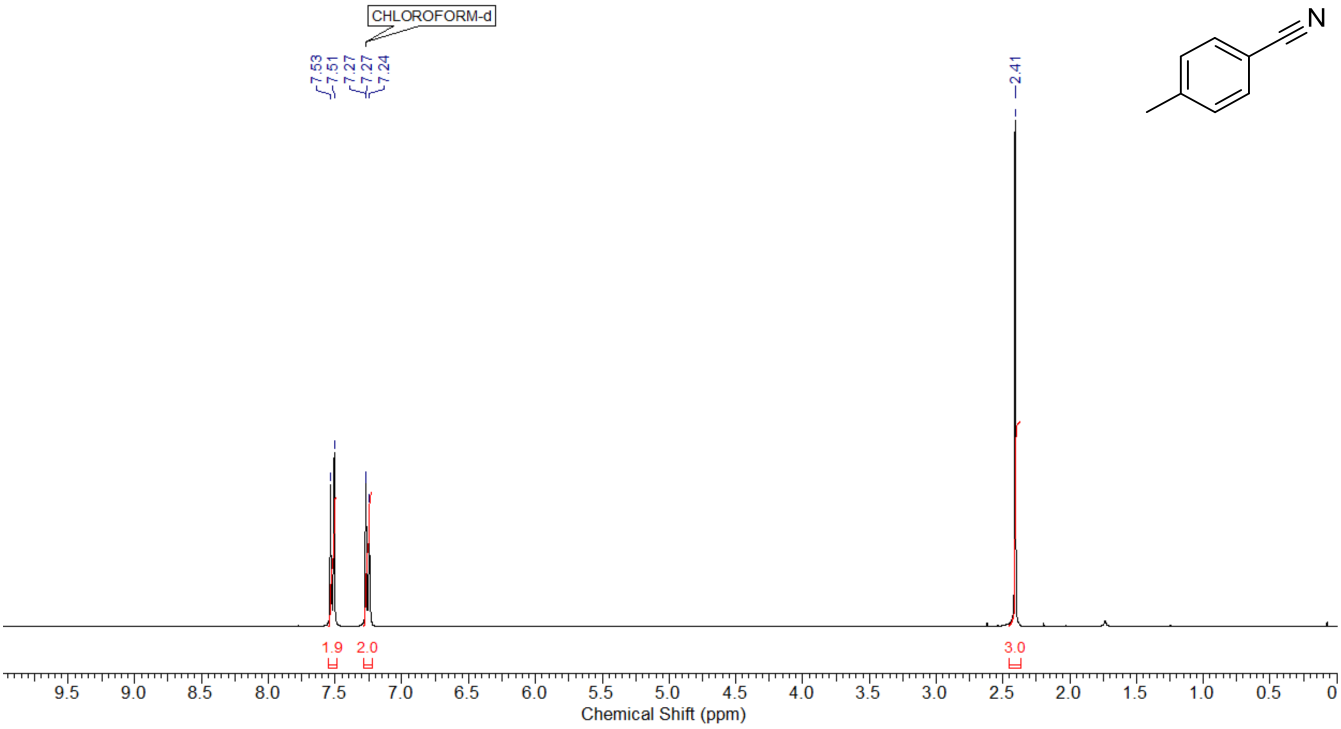

^{13}C NMR (75 MHz, $CDCl_3$):

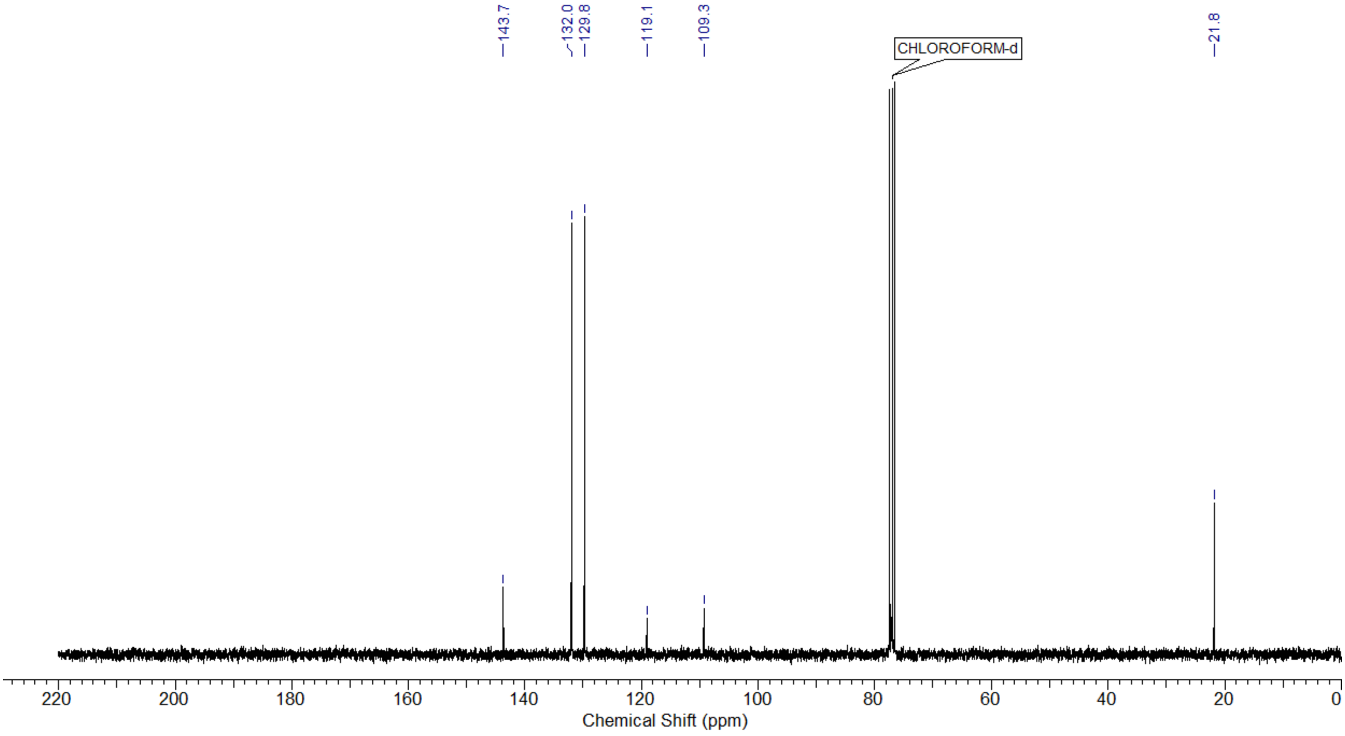

4-Acetylbenzonitril (**16e**) [CAS 1443-80-7]

^{1}H NMR (300 MHz, $CDCl_3$):

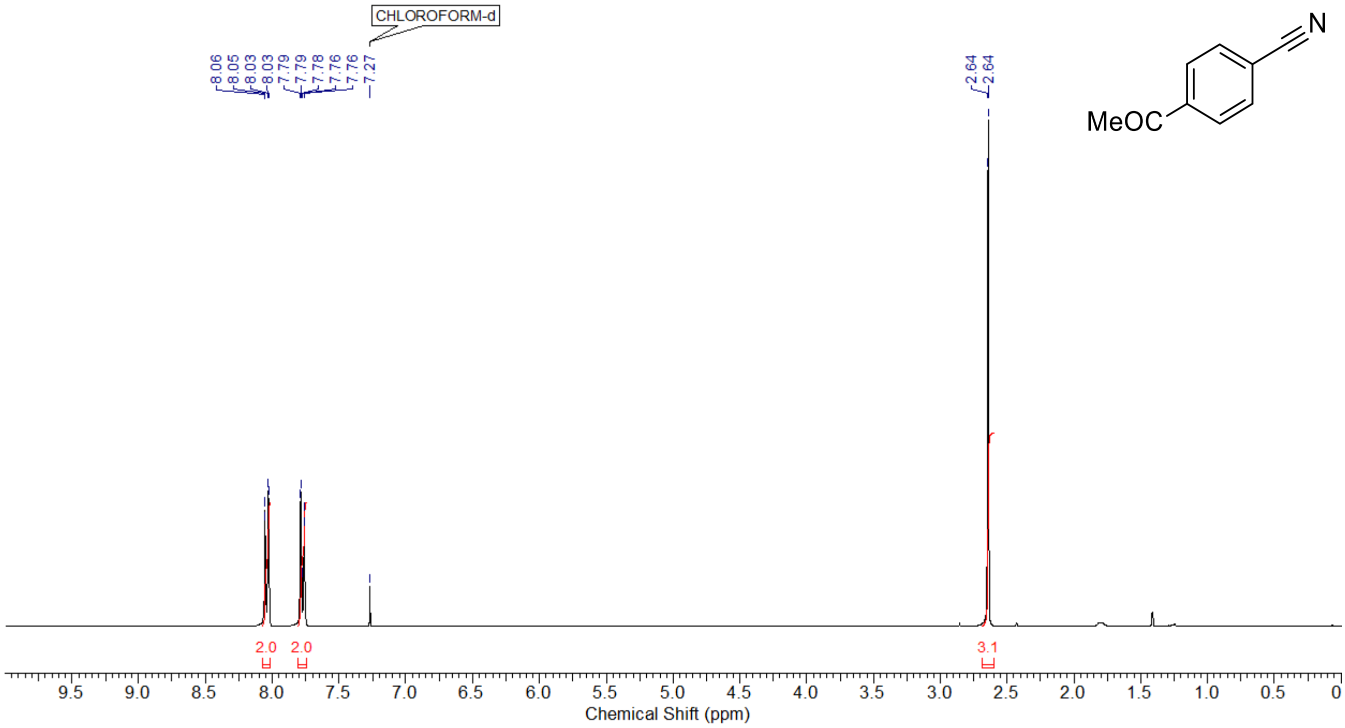

^{13}C NMR (75 MHz, $CDCl_3$):

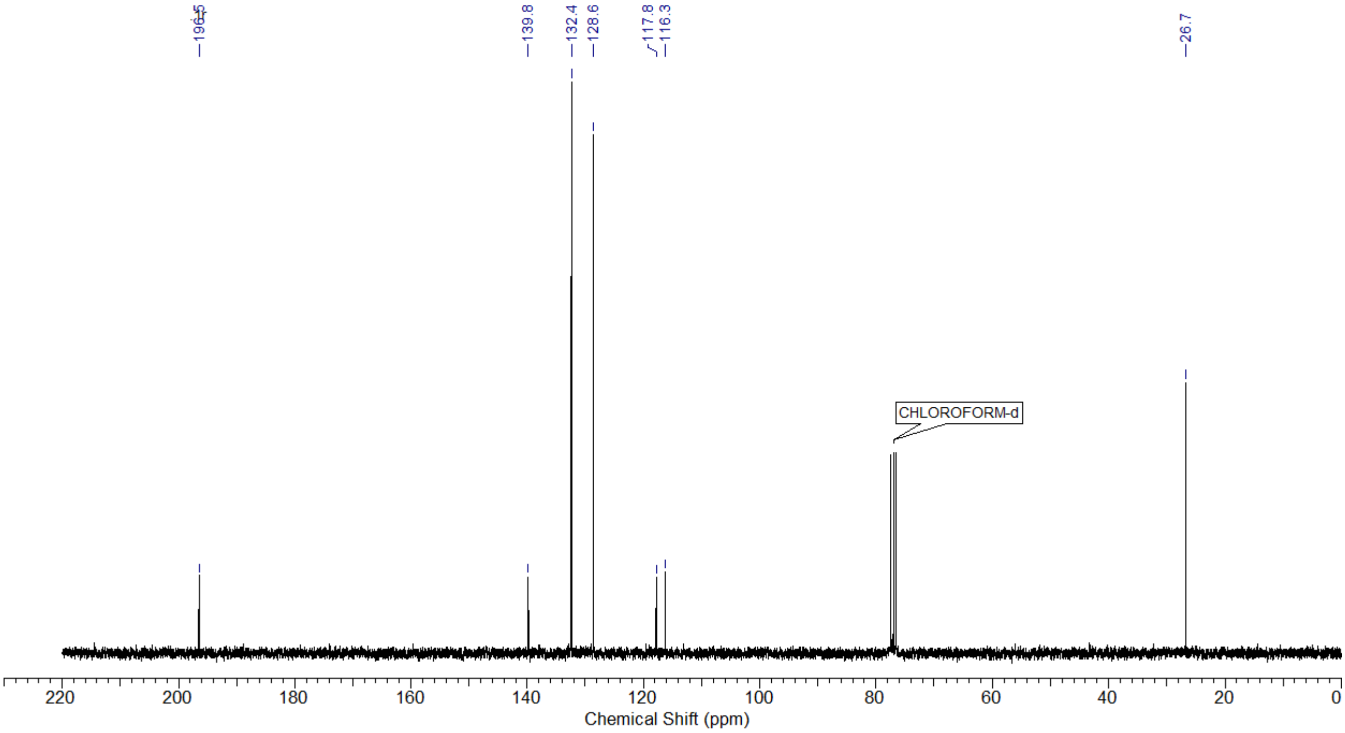

1-Butoxy-4-trifluoromethylbenzene (**18b**) [CAS 944584-50-3]

^1H NMR (300 MHz, $CDCl_3$):

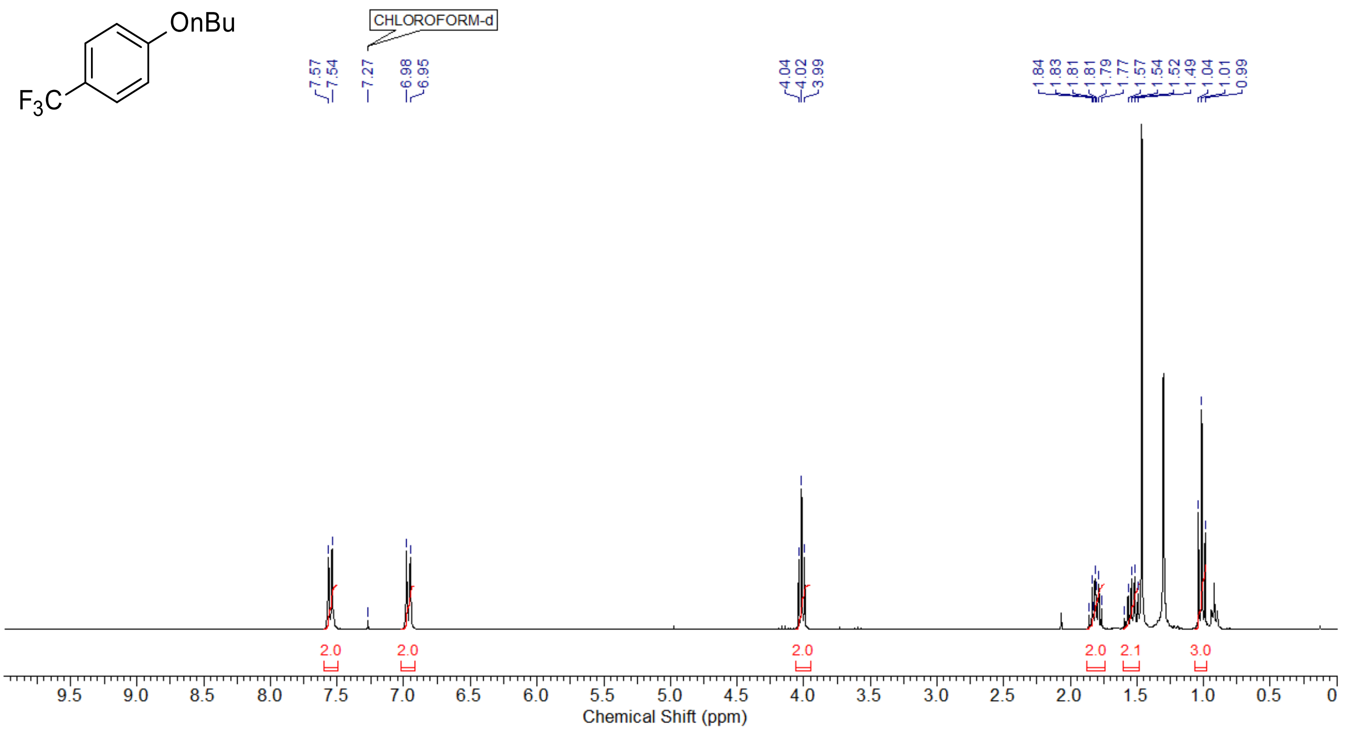

^{13}C NMR (75 MHz, $CDCl_3$):

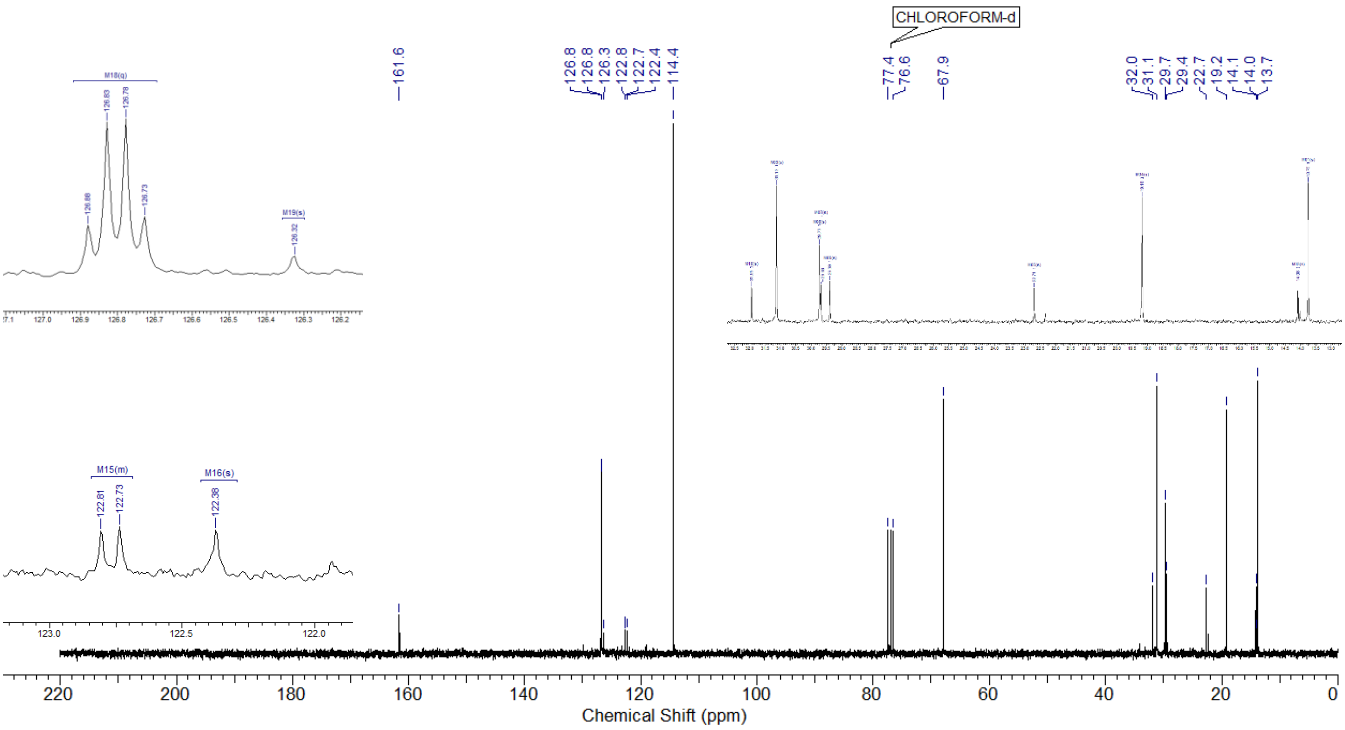

^{19}F NMR (76 MHz, $CDCl_3$, $C_6H_4F_2$):

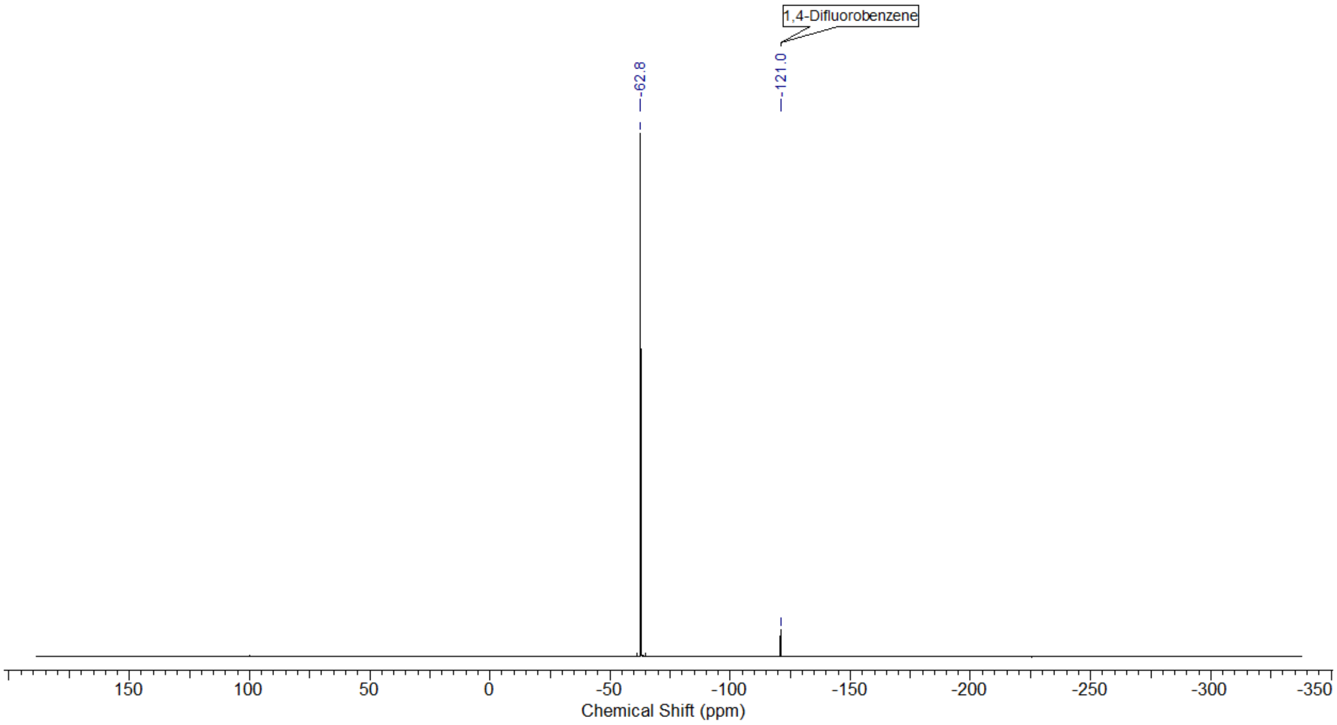

1-Butoxy-4-(methanesulfonyl)benzene (**18c**) [CAS 345222-98-2]

^{1}H NMR (300 MHz, $CDCl_3$):

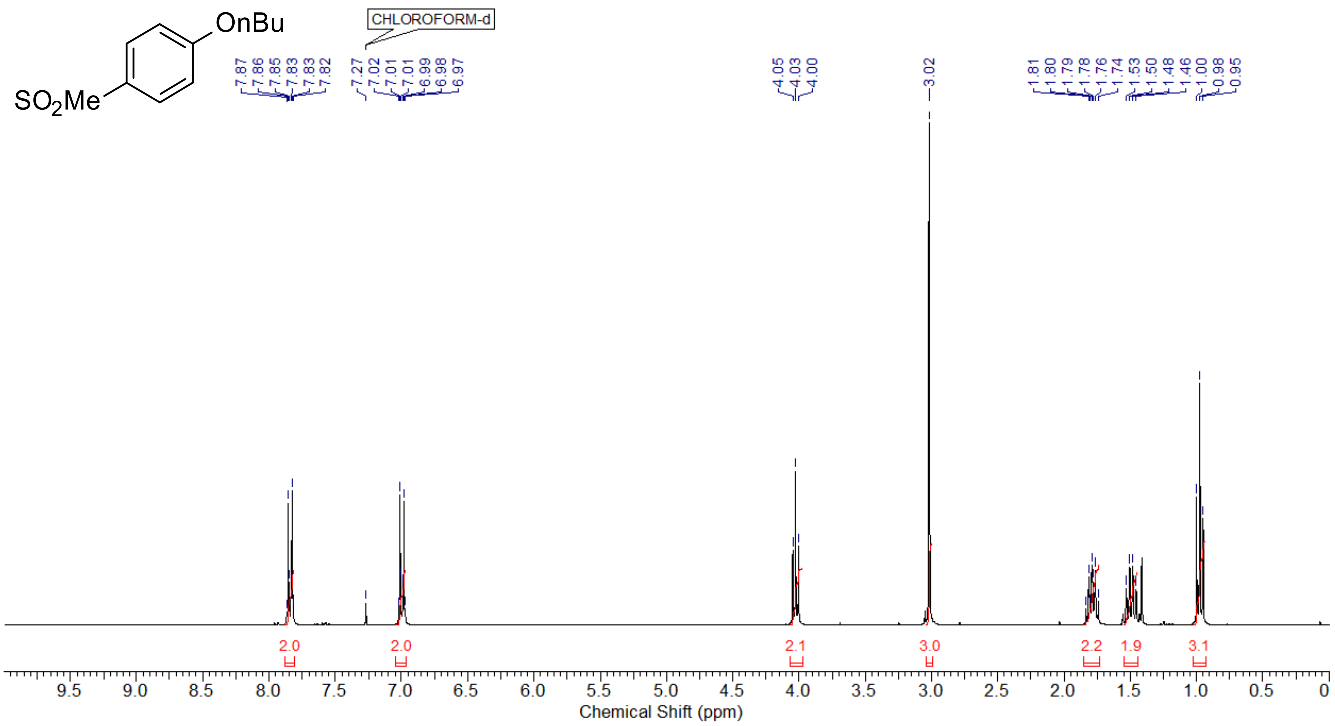

^{13}C NMR (75 MHz, $CDCl_3$):

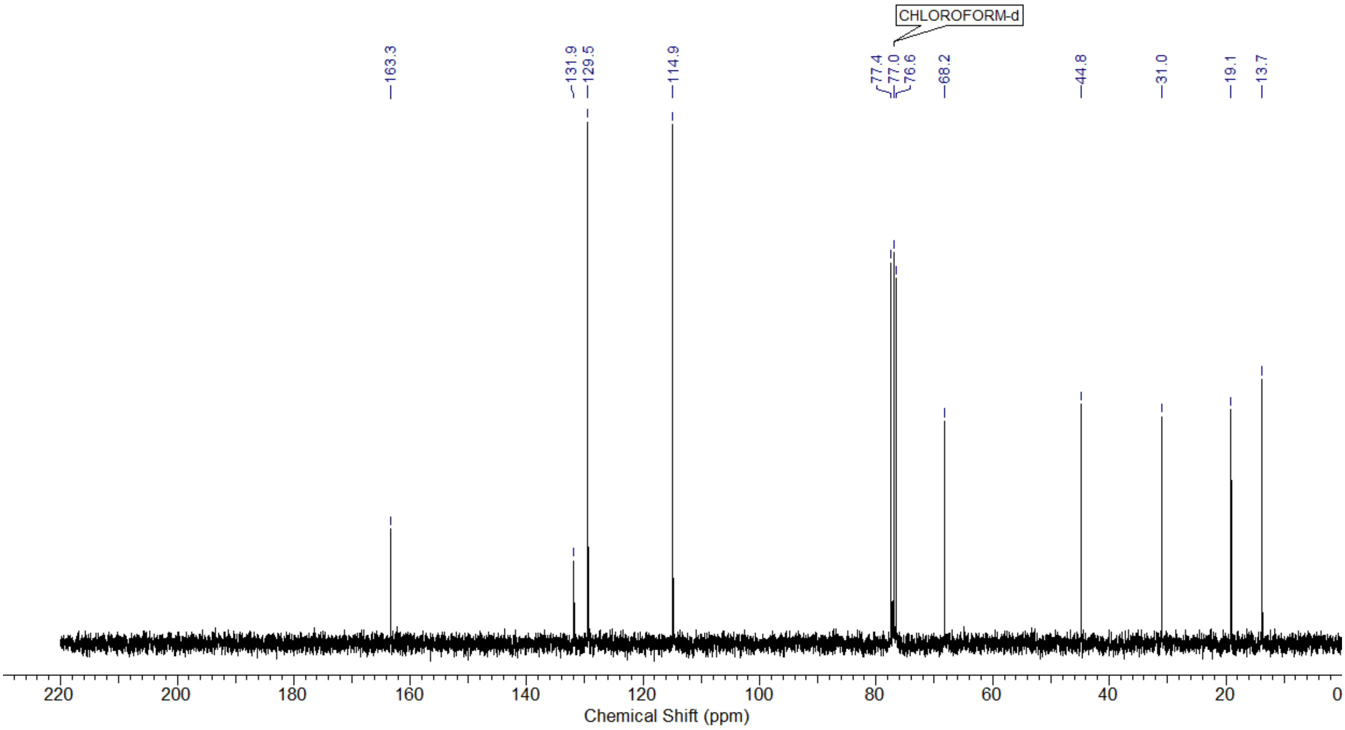

3-Butoxyquinoline (**18d**) [CAS 1239478-93-3]

^{1}H NMR (300 MHz, $CDCl_3$):

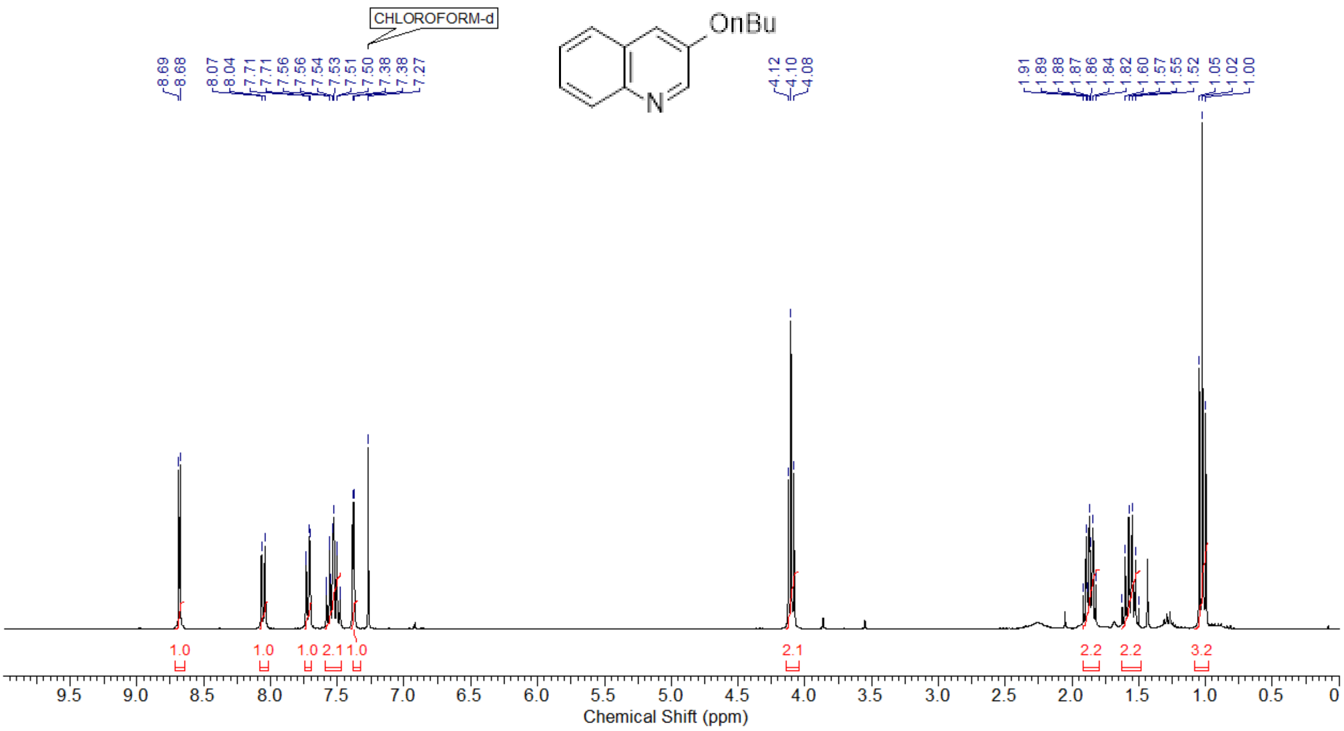

^{13}C NMR (75 MHz, $CDCl_3$):

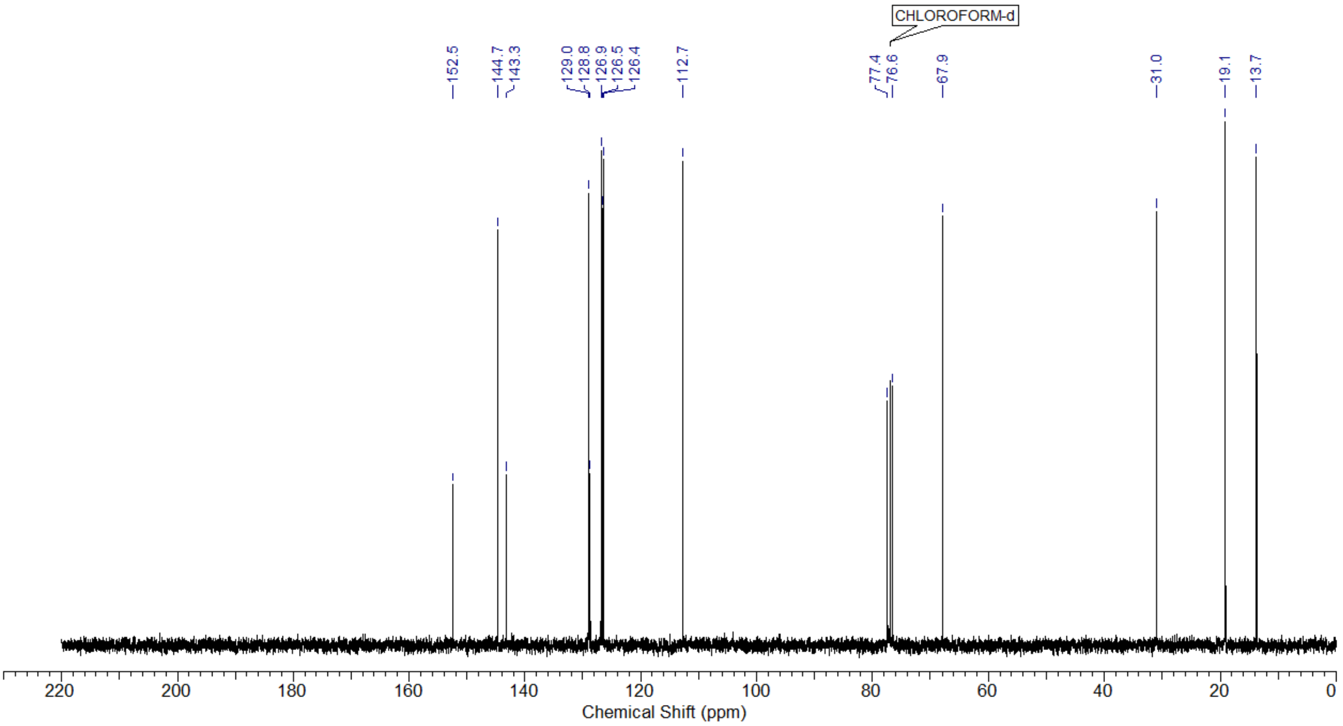

<u>5-Butoxy-2-methylbenzothiazole</u> (**18e**) [CAS 1351403-41-2]

^{1}H NMR (300 MHz, $CDCl_3$):

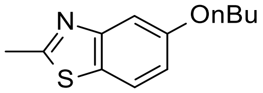

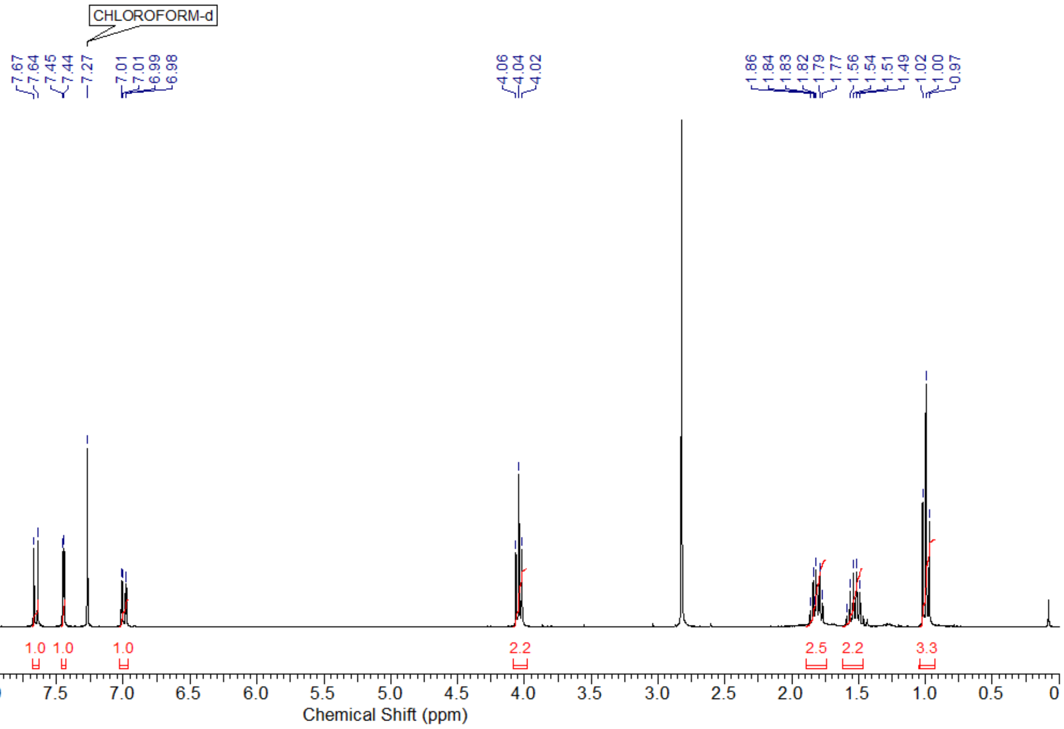

^{13}C NMR (75 MHz, $CDCl_3$):

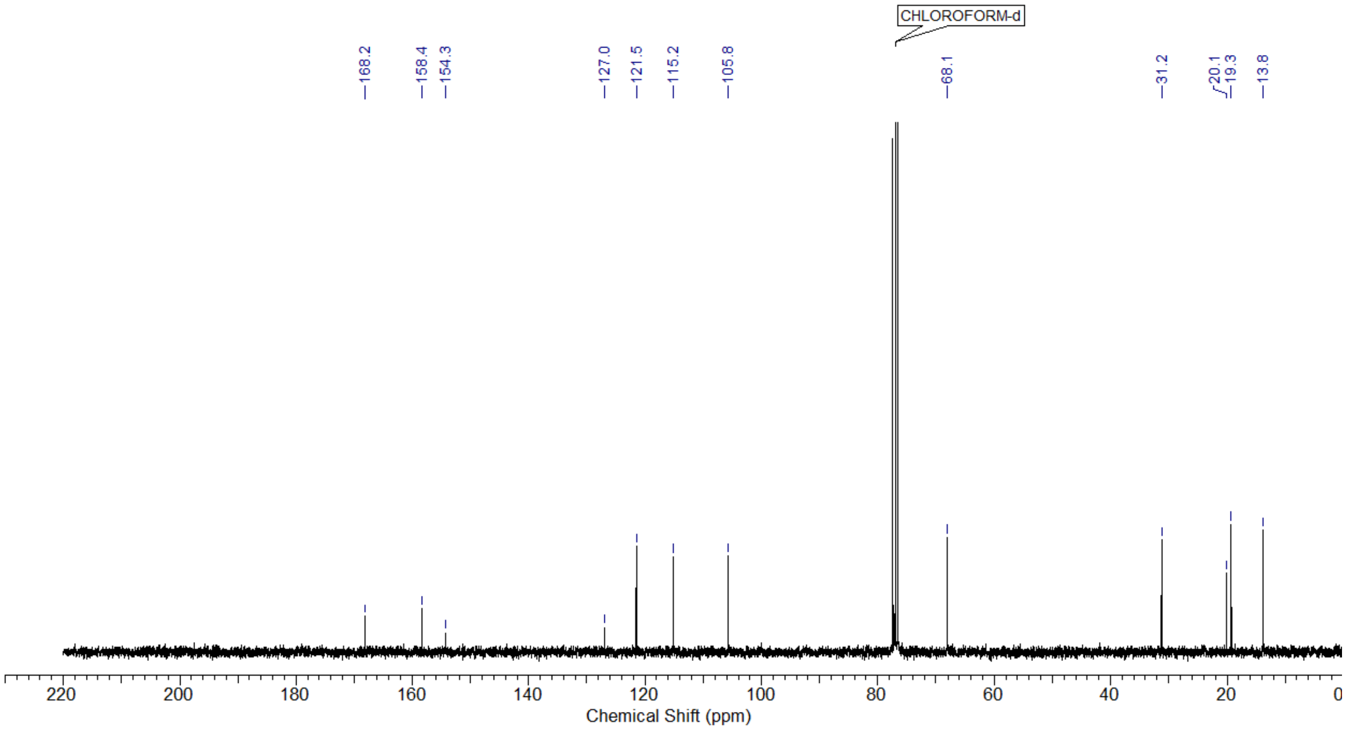

1-Fluoronaphthalene (**19a**) [CAS 321-38-0]

^{1}H NMR (300 MHz, $CDCl_3$):

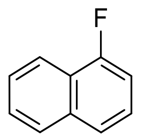

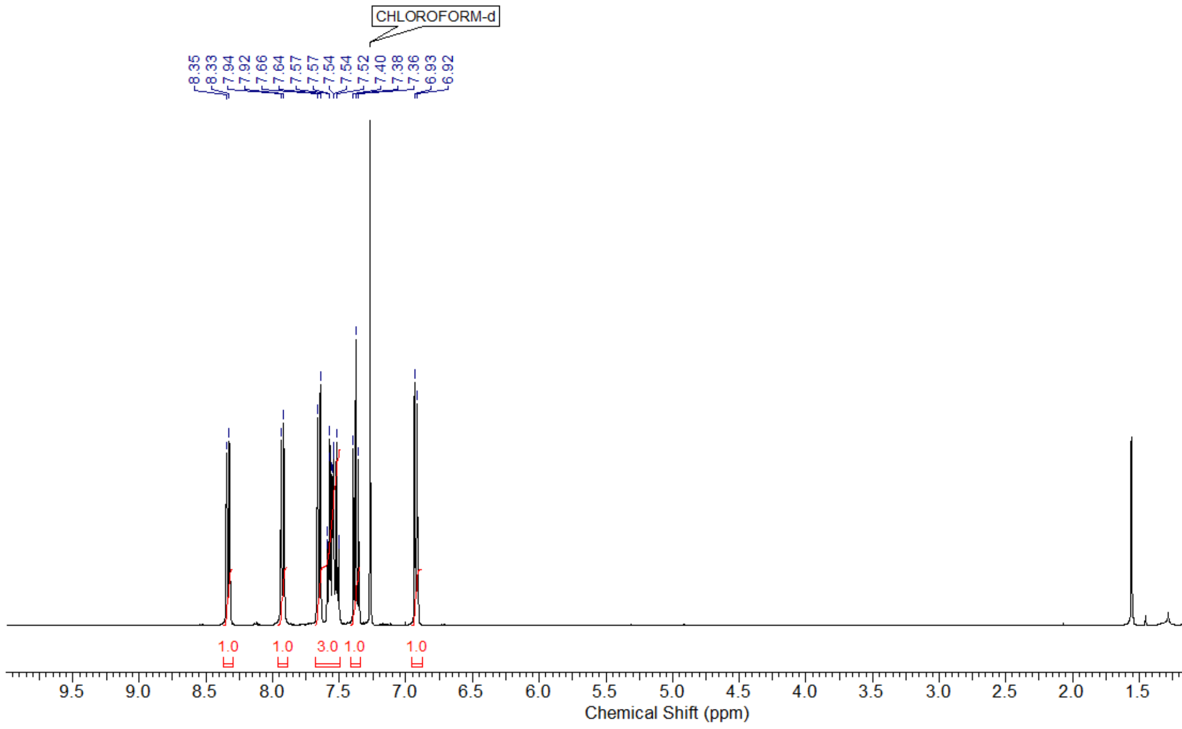

^{13}C NMR (75 MHz, $CDCl_3$):

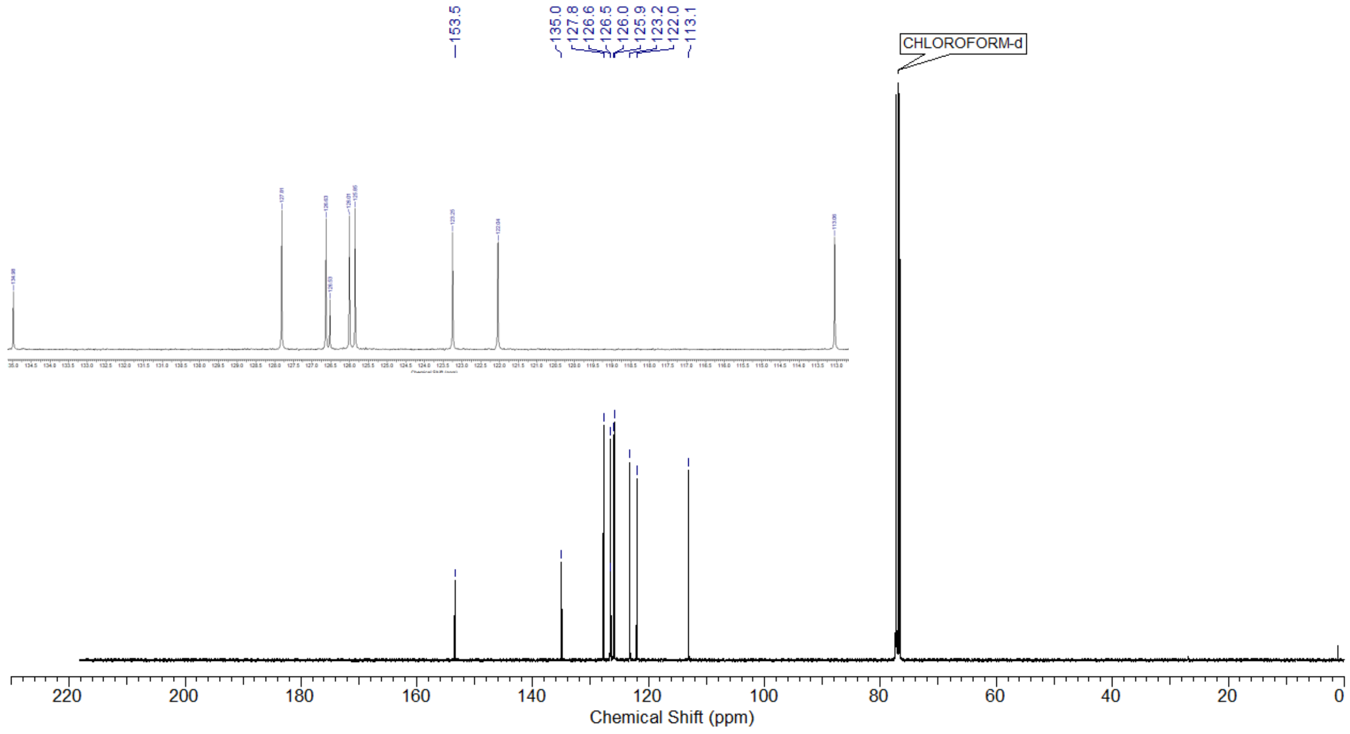

^{19}F NMR (76 MHz, $CDCl_3$, $C_6H_4F_2$):

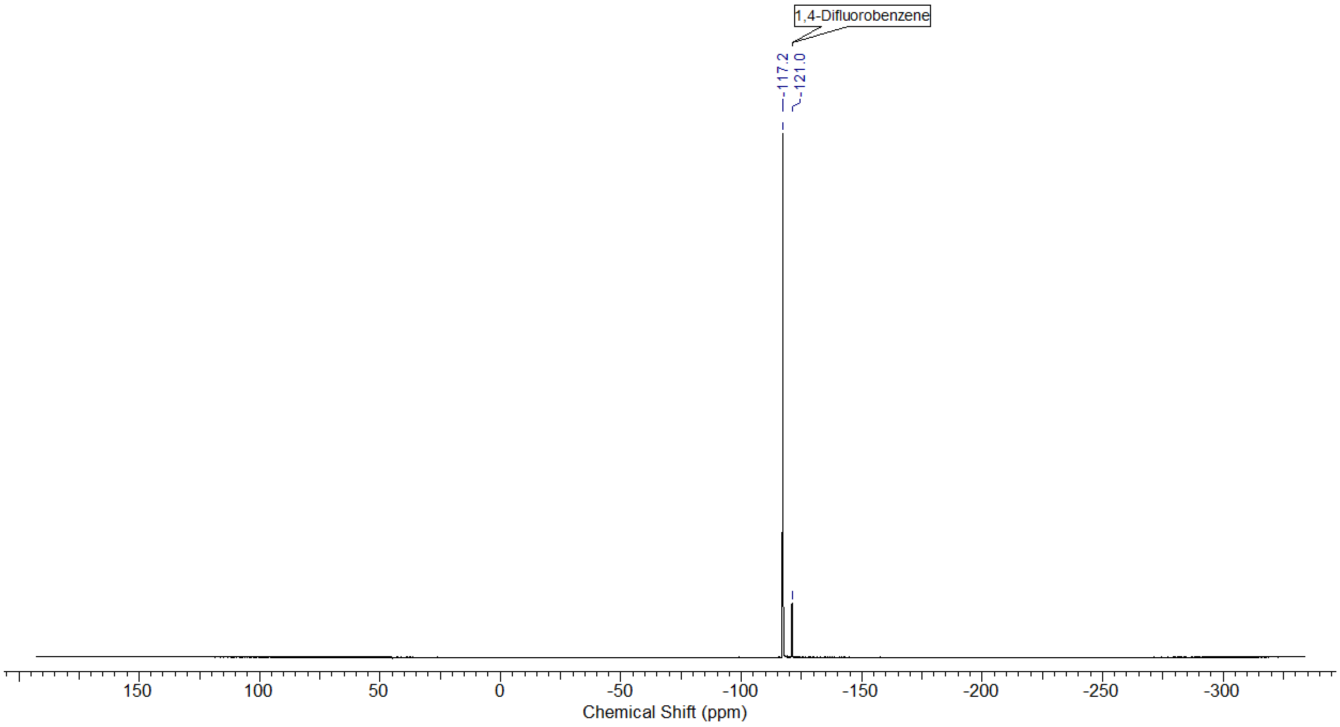

1-Fluor-4-nitrobenzol (**19b**) [CAS 350-46-9]

^{1}H NMR (300 MHz, $CDCl_3$):

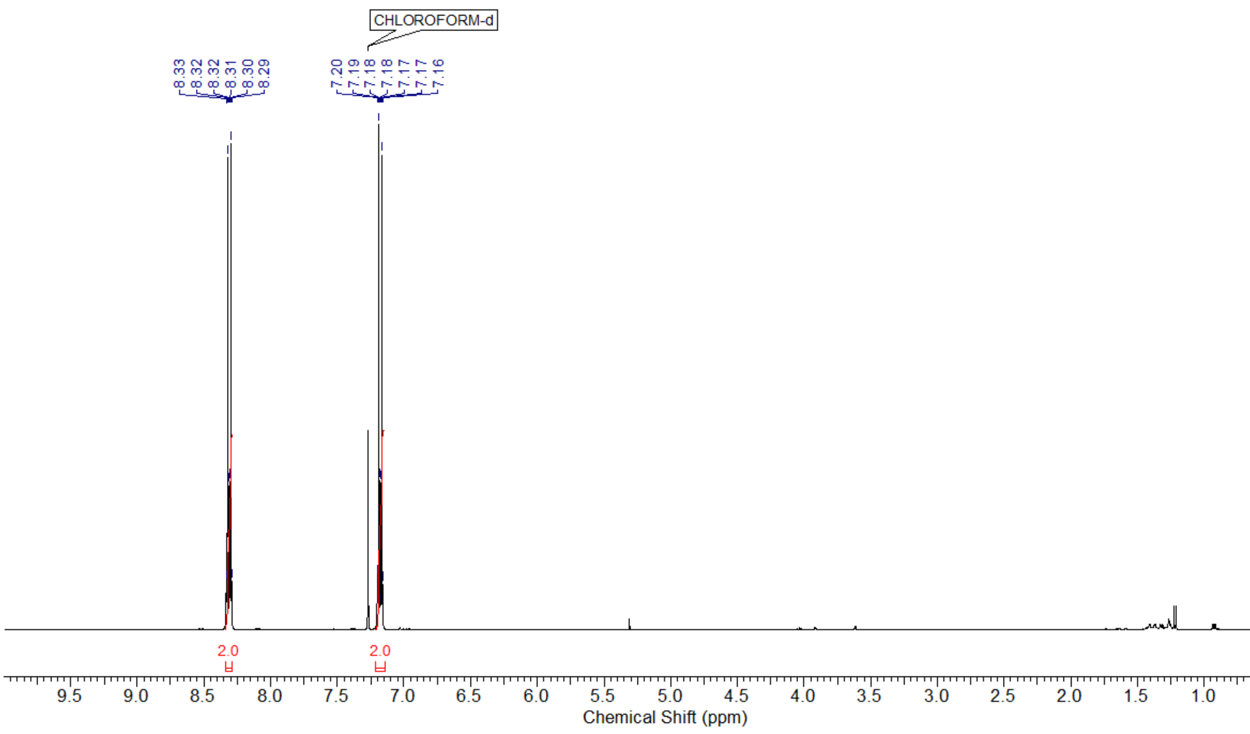

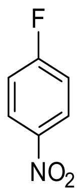

^{13}C NMR (75 MHz, $CDCl_3$):

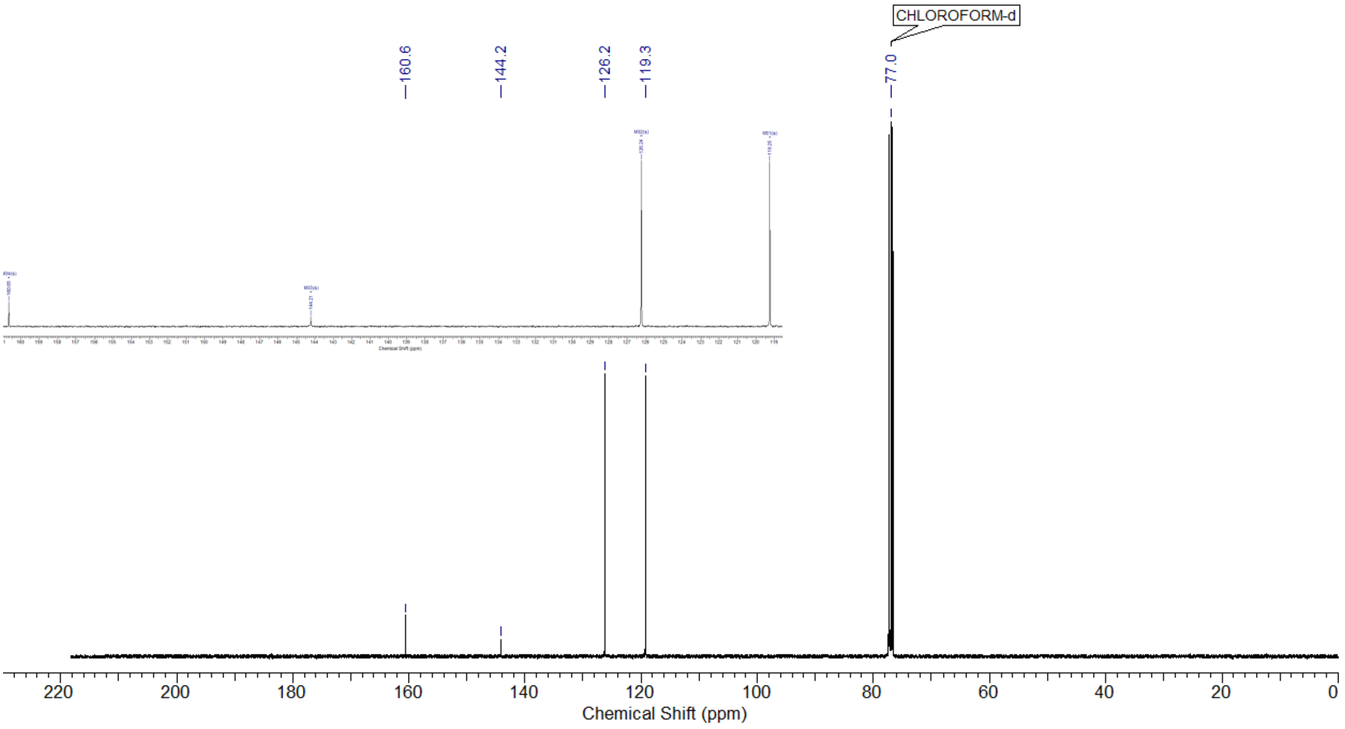

^{19}F NMR (76 MHz, $CDCl_3$, $C_6H_4F_2$):

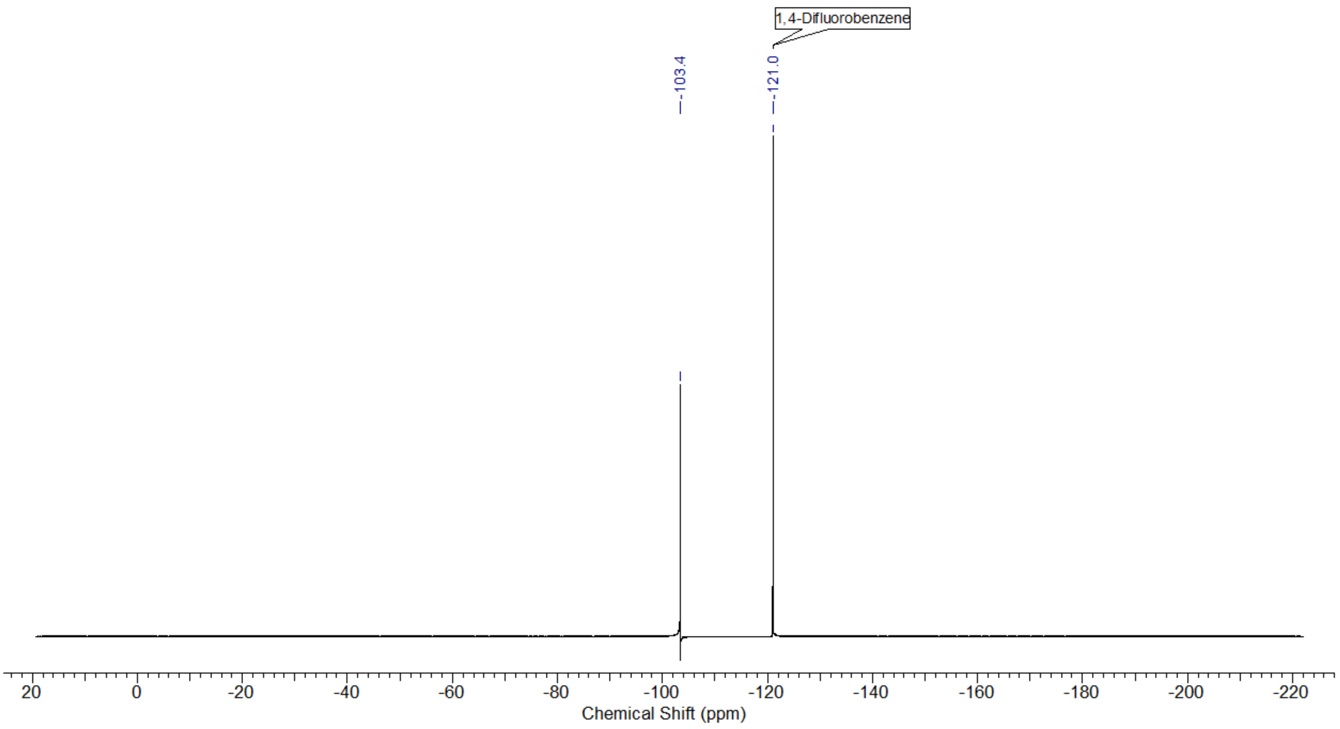

6-Fluoro-2-naphthaldehyde (**19c**) [CAS 721968-77-0]

^{1}H NMR (300 MHz, $CDCl_3$):

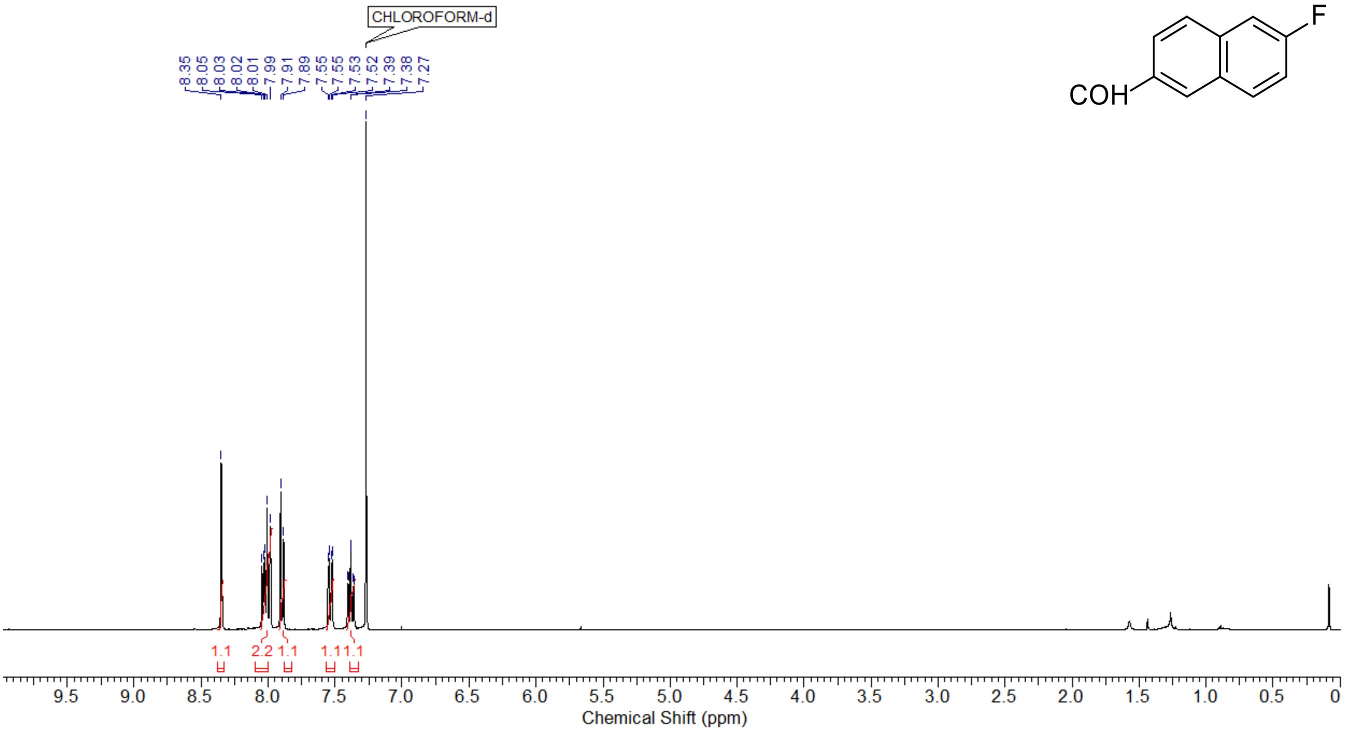

^{13}C NMR (75 MHz, $CDCl_3$):

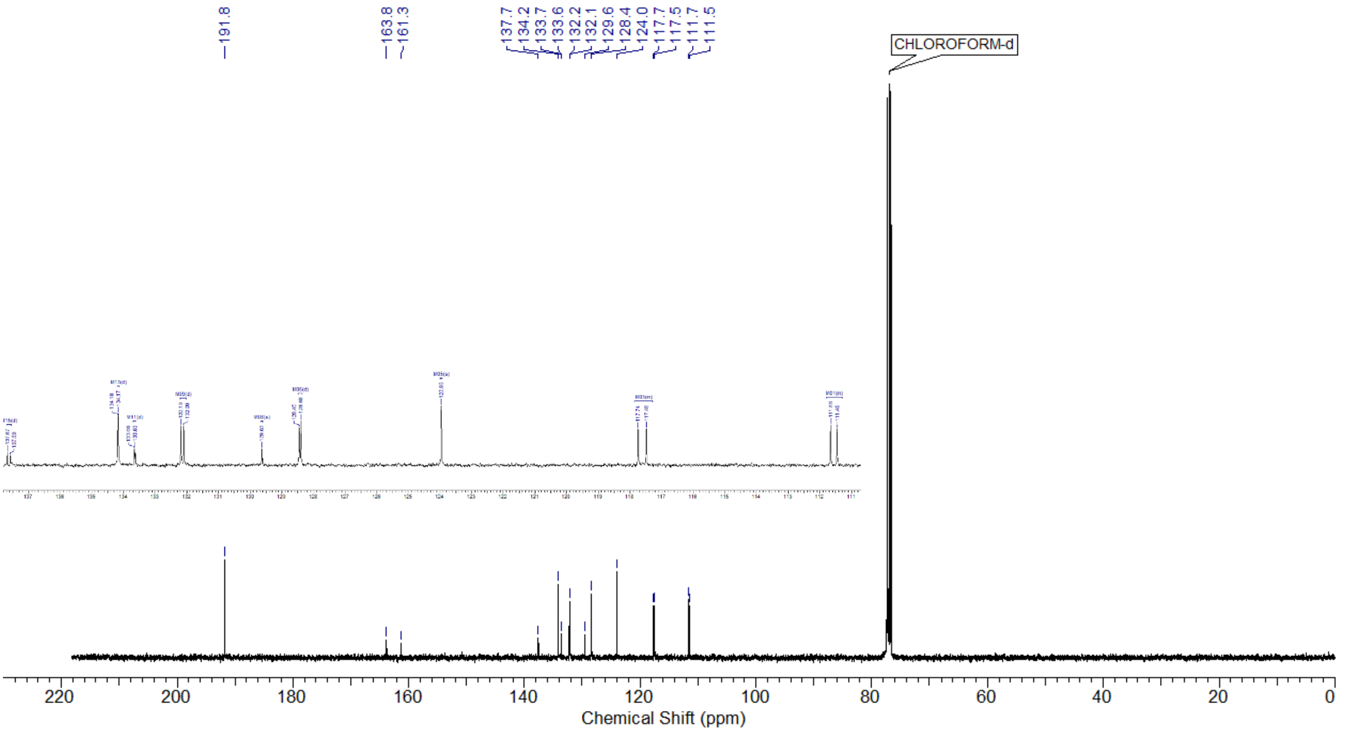

^{19}F NMR (76 MHz, $CDCl_3$, $C_6H_4F_2$):

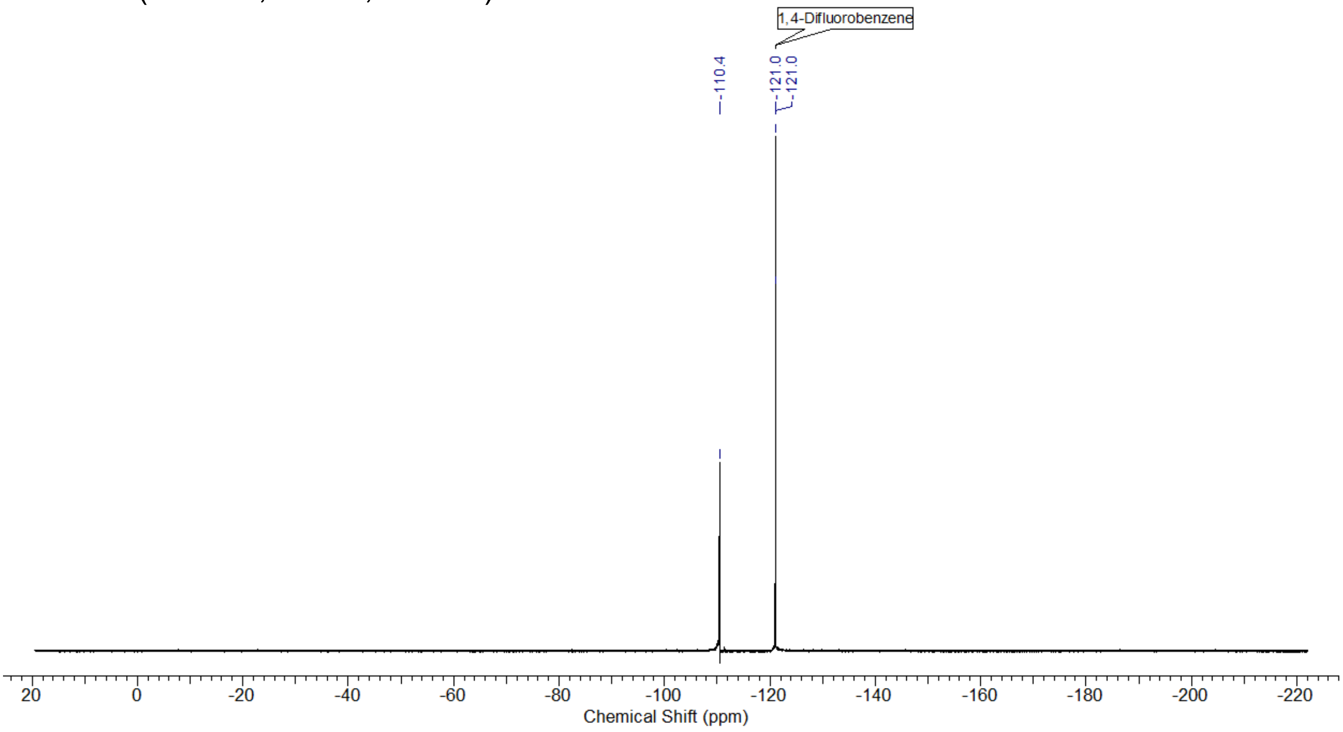

7-Fluoro-2-phenylchromone (**19d**) [CAS 2331212-10-1]

^{1}H NMR (300 MHz, $CDCl_3$):

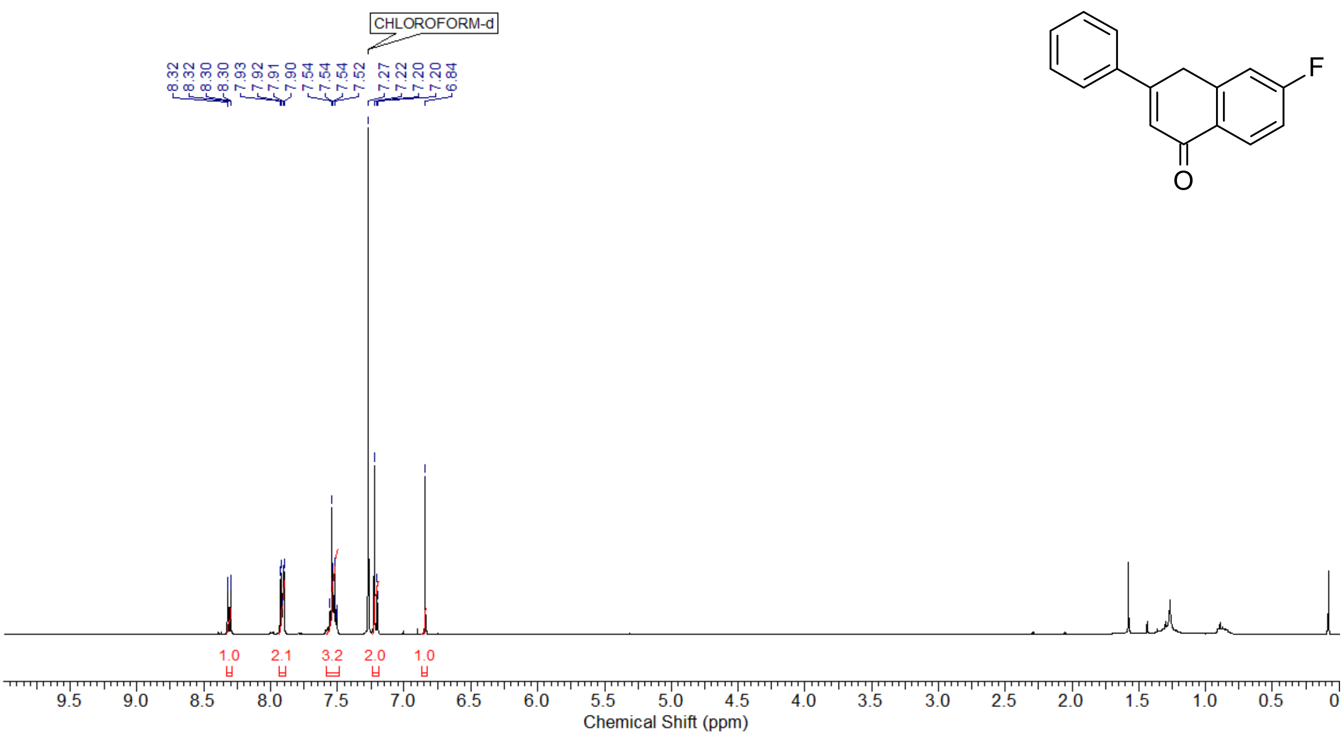

^{13}C NMR (75 MHz, $CDCl_3$):

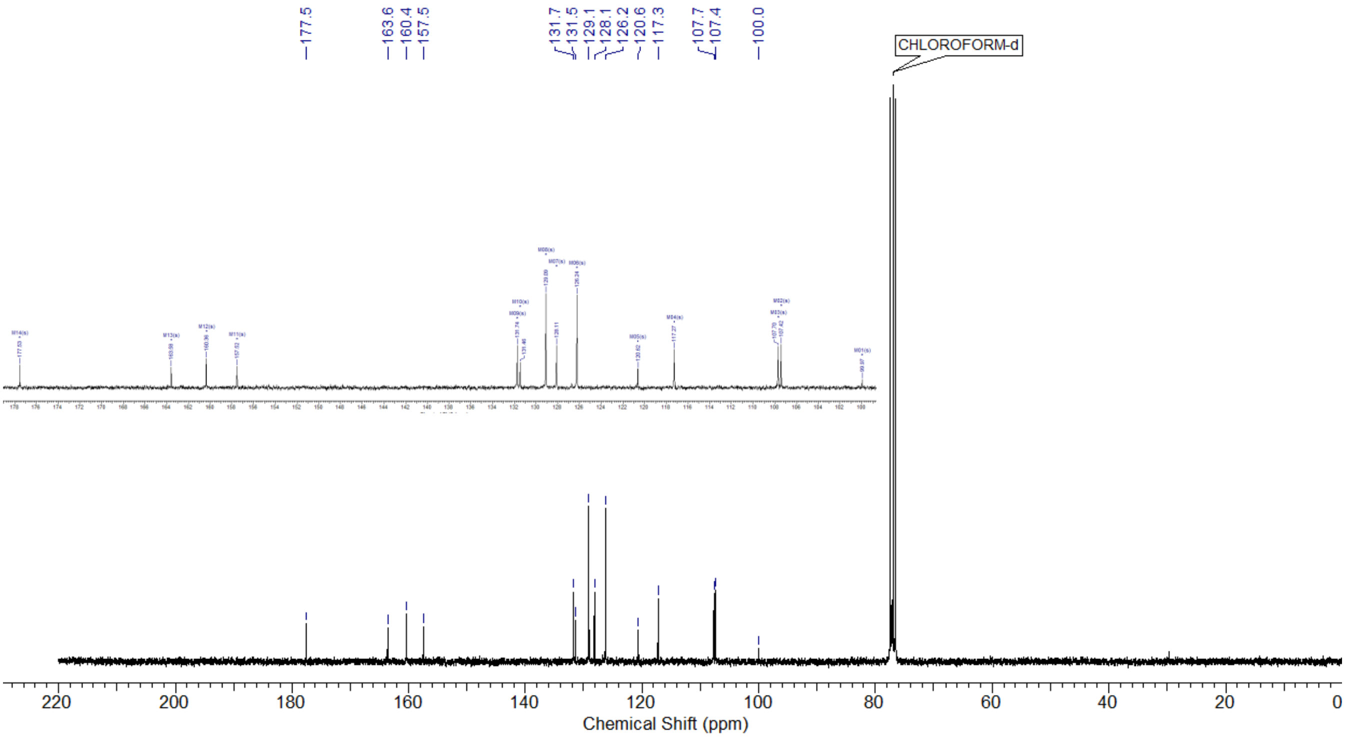

Supplement: Supplementary file 1 — Supporting File 1: anie72355‐sup‐0001‐SuppMat.pdf. [file ANIE-65-e3359287-s001.pdf]
